# Supplementary material for: Site-selective electrooxidation of methylarenes to aromatic acetals
Source: Nat Commun. 2020 Jun 1;11:2706. doi: 10.1038/s41467-020-16519-8 (PMC7264330; doi:10.1038/s41467-020-16519-8)
Supplement: Supplementary file 1 — Supplementary Information [file 41467_2020_16519_MOESM1_ESM.pdf]

Supplementary Information for

Site-selective electrooxidation of methylarenes to aromatic acetals

Peng at al.

Supplementary Methods

General considerations

Anhydrous MeOH was purchased from Thermo Fisher. Other solvents and commercially available reagents were used without purification. Flash column chromatography was performed with silica gel (200–300 mesh). Cyclic voltammograms were recorded on a CHI 760E potentiostat. NMR spectra were recorded on Bruker AV-400, Bruker AV-500, Bruker AV-600 and Bruker AV-850 instruments. Data were reported as chemical shifts in ppm relative to TMS (0.00 ppm) for ^1H and $CDCl_3$ (77.2 ppm) for ^{13}C. The abbreviations used for explaining the multiplicities were as follows: s = singlet, d = doublet, t = triplet, q = quartet, m = multiplet, br = broad. Infrared spectra were recorded on a Nicolet AVATER FTIR330 spectrometer. High resolution mass spectra (ESI) were recorded by the instrumentation center of Department of Chemistry, Xiamen University, on a Micromass QTOF2 Quadruple/Time-of-Flight Tandem mass spectrometer. The reticulated vitreous carbon (100 pores per inch) can be obtained from Goodfellow.

Synthesis and Characterization of New Substrates

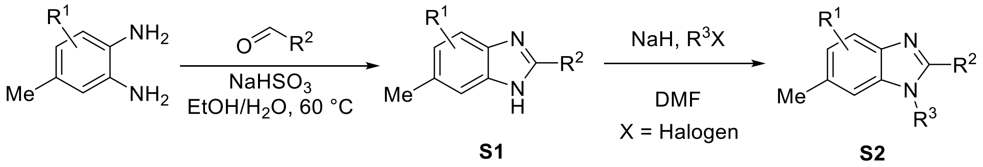

General Procedure A:

Step 1: To a solution of diamine (3.0 mmol, 1.0 equiv) in EtOH (6 mL) and H_2O (2 mL) was added $NaHSO_3$ (7.5 mmol, 2.5 equiv) and aldehyde (3.6 mmol, 1.2 equiv). The reaction was stirred at 60 °C for 8 h and then cooled to RT. The resulting mixture was diluted with H_2O (20 mL) and ethyl acetate (50 mL). The layers were separated. The aqueous phase was extracted twice with ethyl acetate. The combined organic phase was dried over $MgSO_4$, filtered and concentrated under reduced pressure to afford **S1**. The crude product **S1** was used in the next step without purification.

Step 2: **S1** was dissolved in DMF (10 mL) and cooled to 0 °C. NaH (60% dispersion in mineral oil, 4.5 mmol, 1.5 equiv) was added. The reaction mixture was warmed to ambient temperature and stirred for 15 min. R^3X (4.5 mmol, 1.5 equiv) was then added dropwise. After being stirred at RT for 15 min, H_2O (50 mL) was added to quench the reaction. The resulting mixture was extracted with ethyl acetate (50 mL) and the organic layer was washed with H_2O (50 mL). The organic solution was dried over $MgSO_4$, filtered, and concentrated under reduced pressure. The residue was chromatographed through silica gel eluting with ethyl acetate/hexanes to afford **S2**.

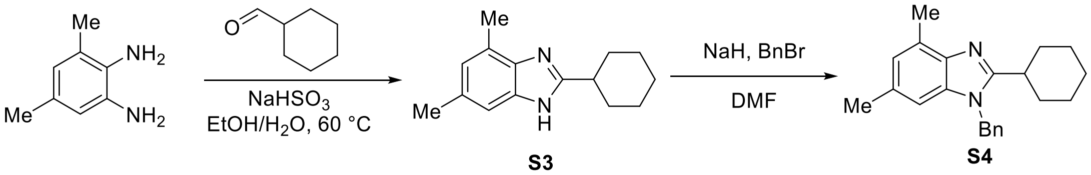

1-Benzyl-2-cyclohexyl-4,6-dimethyl-1*H*-benzo[*d*]imidazole (S4). The title compound was obtained as a white solid in 91% yield (0.33 g) starting from **S3** (0.26 g, 1.1 mmol, 1.0 equiv), BnBr (0.20 mL, 1.7 mmol, 1.5 equiv) by following the General Procedure A (step 2). The structure was confirmed by nuclear Overhauser effect (NOE) experiment. ^{1}H NMR (500 MHz, $CDCl_3$) δ 7.30–7.23 (m, 3H), 7.03–7.00 (m, 2H), 6.85 (s, 1H), 6.79 (s, 1H), 5.29 (s, 2H), 2.80–2.72 (m, 1H), 2.65 (s, 3H), 2.36 (s, 3H), 1.88–1.78 (m, 6H), 1.73–1.67 (m, 1H), 1.37–1.24 (m, 3H); ^{13}C NMR (126 MHz, $CDCl_3$) δ 158.2, 140.4, 136.9, 135.3, 131.9, 129.0, 128.9, 127.8, 126.2, 124.1, 107.1, 46.8, 36.9, 31.9, 26.5, 25.8, 21.9, 17.0; IR (neat, cm^{-1}): 2925, 2852, 1635, 1516, 1451; ESI HRMS m/z $(M+H)^+$ calcd 319.2169, obsd 319.2177.

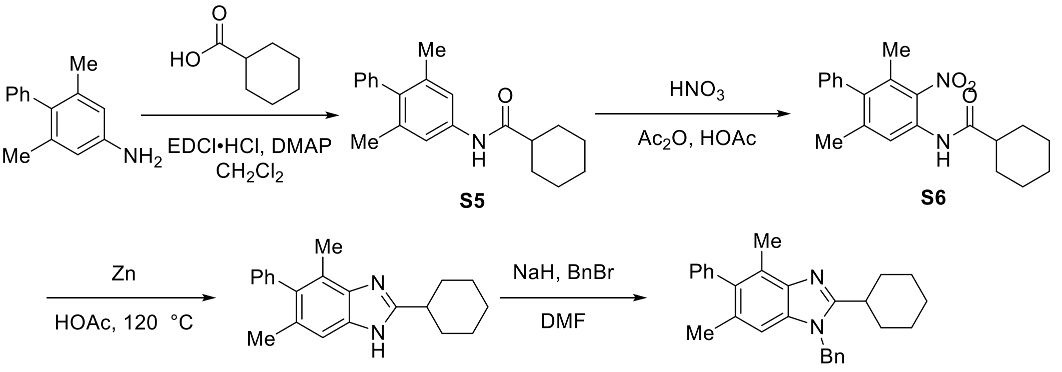

***N*-(2,6-Dimethyl-[1,1'-biphenyl]-4-yl)cyclohexanecarboxamide (S5)**. To a solution of cyclohexanecarboxylic acid (0.62 g, 4.8 mmol, 1.2 equiv) and EDCl•HCl (0.92 g, 4.8 mmol, 1.2 equiv) in CH_2Cl_2 (10 mL) was added DMAP (0.05 g, 0.40 mmol, 0.10 equiv), followed by 2,6-dimethyl-[1,1'-biphenyl]-4-amine (0.80 g, 4.0 mmol, 1.0 equiv). The resulting reaction mixture was stirred at RT for 0.5 h, then diluted with CH_2Cl_2 and quenched with 1 N HCl (20 mL). The layers were separated and the aqueous layer was extracted twice with CH_2Cl_2. The combined organic solution was washed with saturated $NaHCO_3$, dried over $MgSO_4$ and concentrated under reduced pressure. The crude solid was washed with hexane and used in the next step without further purification.

***N*-(2,6-Dimethyl-3-nitro-[1,1'-biphenyl]-4-yl)cyclohexanecarboxamide (S6)**. To a suspension of **S5** (0.70 g, 2.3 mmol, 1.0 equiv) in Ac_2O (4.0 mL) and AcOH (0.4 mL) at 0 °C was added nitric acid (70%, 0.30 mL, 8.0 mmol, 2.0 equiv) dropwise. After addition, the reaction mixture was warmed to RT and stirred for 0.5 h. The reaction mixture was then cooled to 0 °C, diluted with CH_2Cl_2 and basified with aqueous NaOH solution (3.0 M) until

pH 8. The layers were separated. The aqueous phase was extracted with CH_2Cl_2. The combined organic layers were washed with brine, dried over $MgSO_4$ and concentrated under reduced pressure. The crude product **S6** was used directly in the next step without further purification.

2-Cyclohexyl-4,6-dimethyl-5-phenyl-1*H*-benzo[*d*]imidazole (S7). A suspension of **S6** (2.3 mmol, 1.0 equiv) and Zn dust (0.75 g, 12 mmol, 5.0 equiv) in AcOH (6.0 mL) was heated to 120 °C for 0.5 h. The resulting reaction mixture was then cooled to RT, concentrated and basified with aqueous NaOH solution (3.0 N) until pH 10. The aqueous mixture was extracted twice with CH_2Cl_2. The combined organic layers were dried over $MgSO_4$ and concentrated under reduced pressure. The crude product **S7** was used directly in the next step without further purification.

1-Benzyl-2-cyclohexyl-4,6-dimethyl-5-phenyl-1*H*-benzo[*d*]imidazole (S8). The title compound was obtained as a white solid in 66% yield (0.60 g) starting from **S7** (0.70 g, 2.3 mmol, 1.0 equiv), BnBr (0.33 mL, 2.8 mmol, 1.2 equiv) by following the General Procedure A (Step 2). The structure was confirmed by nuclear Overhauser effect (NOE) experiment. ^{1}H NMR (600 MHz, $CDCl_3$) δ 7.43–7.38 (m, 2H), 7.34–7.29 (m, 3H), 7.29–7.25 (m, 1H), 7.18–7.15 (m, 2H), 7.11–7.07 (m, 2H), 6.91 (s, 1H), 5.33 (s, 2H), 2.79 (tt, *J* = 9.7, 5.3 Hz, 1H), 2.38 (s, 3H), 2.06 (s, 3H), 1.90–1.81 (m, 6H), 1.73–1.68 (m, 1H), 1.38–1.27 (m, 3H); ^{13}C NMR (101 MHz, $CDCl_3$) δ 158.4, 141.8, 140.8, 136.9, 136.0, 134.1, 130.6, 130.0, 129.1, 128.4, 127.8, 127.2, 126.5, 126.3, 107.5, 46.8, 36.9, 32.0, 26.5, 25.8, 22.0, 15.1; IR (neat, cm^{-1}): 2923, 2851, 1704, 1631, 1509, 1450; ESI HRMS *m/z* $(M+H)^+$ calcd 395.2482, obsd 395.2500.

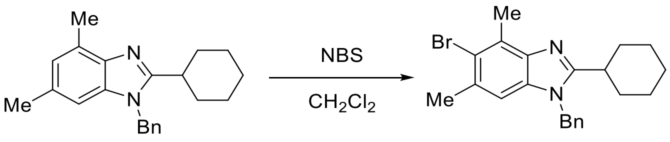

S4 **S9**

1-Benzyl-5-bromo-2-cyclohexyl-4,6-dimethyl-1*H*-benzo[*d*]imidazole (S9). To a solution of **S4** (0.13 g, 0.41 mmol, 1.0 equiv) in CH_2Cl_2 (5 mL) was added NBS (0.085 g, 0.48 mmol, 1.2 equiv). The resulting mixture was stirred at RT for 1 h and concentrated under reduced pressure. The residue was purified by silica gel column chromatography to afford **S9** as a white solid in 80% yield (0.13 g). ^{1}H NMR (400 MHz, $CDCl_3$) δ 7.00 (s, 1H), 3.66 (s, 3H), 2.81 (tt, *J* = 11.7, 3.4 Hz, 1H), 2.72 (s, 3H), 2.52 (s, 3H), 2.01–1.86 (m, 4H), 1.86–1.74 (m, 3H), 1.48–1.33 (m, 3H); ^{13}C NMR (101 MHz, $CDCl_3$) δ 158.9, 141.6, 136.5, 133.8, 131.7, 129.6, 129.1, 127.9, 126.1, 120.7, 108.2, 46.8, 36.8, 31.9, 26.4, 25.8, 24.9, 18.0; IR (neat, cm^{-1}): 2922, 2851, 1646, 1416, 1355, 1129, 994, 729; ESI HRMS *m/z* $(M+H)^+$ calcd

397.1274, obsd 397.1271.

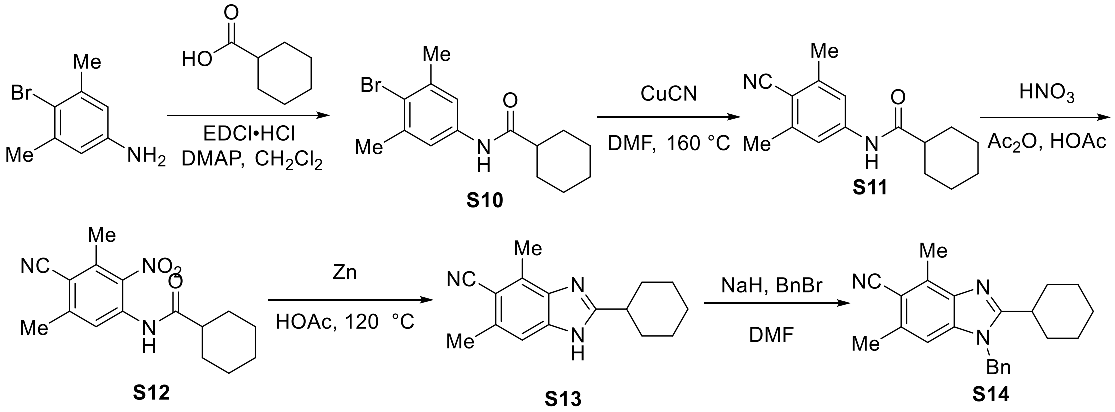

***N*-(4-Bromo-3,5-dimethylphenyl)cyclohexanecarboxamide (S10)**. **S10** was obtained as a white solid in 80% yield (1.0 g) starting from 4-bromo-3,5-dimethylaniline (0.8 g, 4.0 mmol, 1.0 equiv) and cyclohexanecarboxylic acid (0.62 g, 4.8 mmol, 1.2 equiv) by following the procedure described for the synthesis of **S5**. The crude product **S10** was used directly in the next step without further purification.

***N*-(4-Cyano-3,5-dimethylphenyl)cyclohexanecarboxamide (S11)**. A suspension of **S10** (1.0 g, 3.2 mmol, 1.0 equiv) and CuCN (1.4 g, 16 mmol, 5.0 equiv) in DMF (20 mL) was heated to 160 °C for 8 h. The resulting reaction mixture was cooled to RT, filtered and washed with EtOAc. The filtrate was then washed with H_2O, dried over $MgSO_4$ and concentrated under reduced pressure. The crude product **S11** was used directly in the next step without further purification.

1-Benzyl-2-cyclohexyl-4,6-dimethyl-1*H*-benzo[*d*]imidazole-5-carbonitrile (S14). The title compound was obtained as a white solid in 36% yield (0.40 g) for three steps starting from **S11** (0.55 g, 2.1 mmol) by following the procedure described for the synthesis of **S5** to **S8**. The structure was confirmed by nuclear Overhauser effect (NOE) experiment. ^{1}H NMR (600 MHz, $CDCl_3$) δ 7.34–7.27 (m, 3H), 7.02–6.97 (m, 2H), 6.89 (s, 1H), 5.33 (s, 2H), 2.84 (s, 3H), 2.77 (tt, *J* = 11.4, 3.5 Hz, 1H), 2.54 (s, 3H), 1.89–1.76 (m, 6H), 1.75–1.70 (m, 1H), 1.38–1.25 (m, 3H); ^{13}C NMR (151 MHz, $CDCl_3$) δ 160.5, 140.3, 137.5, 135.9, 135.4, 135.1, 129.2, 128.2, 126.0, 118.6, 108.5, 106.5, 47.0, 36.7, 31.8, 26.3, 25.7, 21.4, 15.7; IR (neat, cm^{-1}): 2928, 2852, 2214, 1614, 1516, 1452, 1356, 1264; ESI HRMS *m/z* $(M+H)^+$ calcd 344.2121, obsd 344.2137.

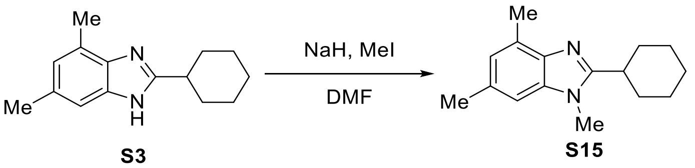

2-Cyclohexyl-1,4,6-trimethyl-1*H*-benzo[*d*]imidazole (S15). The title compound was obtained as a white solid in 87% yield (0.24 g) starting from **S3** (0.26 g, 1.1 mmol, 1.0 equiv) and MeI (0.11 mL, 1.7 mmol, 1.5 equiv) by following the General Procedure A (step 2). The structure was confirmed by nuclear Overhauser effect (NOE) experiment. ^{1}H NMR (500 MHz, $CDCl_3$) δ 6.91 (s, 1H), 6.87 (s, 1H), 3.68 (s, 3H), 2.85 (tt, *J* = 11.7, 3.5 Hz, 1H), 2.64 (s, 3H), 2.46 (s, 3H), 2.02–1.96 (m, 2H), 1.93 (dq, *J* = 13.5, 3.4 Hz, 2H), 1.88–1.75 (m, 3H), 1.48–1.36 (m, 3H); ^{13}C NMR (126 MHz, $CDCl_3$) δ 157.8, 140.2, 135.9, 131.6, 128.8, 123.8, 106.5, 36.8, 31.5, 29.8, 26.5, 25.9, 21.9, 17.0; IR (neat, cm^{-1}): 2929, 1513, 1450, 1358, 1232, 1034, 825, 770; ESI HRMS *m/z* $(M+H)^+$ calcd 243.1856, obsd 243.1854.

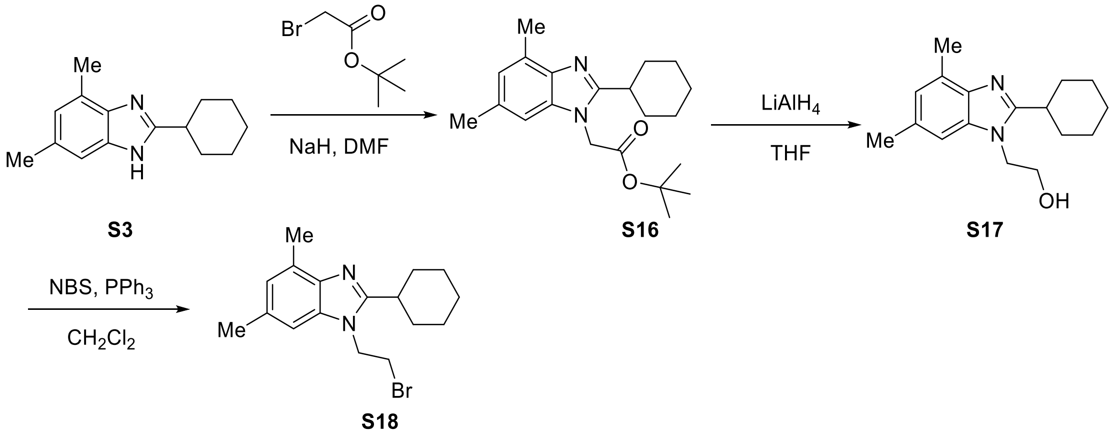

***tert*-Butyl 2-(2-cyclohexyl-4,6-dimethyl-1*H*-benzo[*d*]imidazol-1-yl)acetate (S16)**. **S3** (0.35 g, 1.5 mmol, 1.0 equiv)) was dissolved in DMF (10 mL) and cooled to 0 °C. NaH (60% dispersion in mineral oil, 0.090 g, 2.3 mmol, 1.5 equiv) was added. The reaction mixture was warmed to ambient temperature and stirred for 15 min. *tert*-butyl 2-bromoacetate (0.33 mL, 2.3 mmol, 1.5 equiv) was then added dropwise. After being stirred at RT for 15 min, H_2O (50 mL) was added to quench the reaction. The resulting mixture was extracted with ethyl acetate (50 mL) and the organic layer was washed with H_2O (50 mL). The organic layer was dried over $MgSO_4$, filtered and concentrated under reduced pressure. The residue was used in the next step without further purification.

2-(2-Cyclohexyl-4,6-dimethyl-1*H*-benzo[*d*]imidazol-1-yl)ethan-1-ol (S17). To a solution of **S16** in THF (10 mL) was added $LiAlH_4$ (0.084 g, 2.3 mmol, 1.5 equiv). The reaction mixture was stirred at RT for 0.5 h, diluted with CH_2Cl_2 and quenched sequentially with 0.1 mL of water, 0.1 mL of 15% aqueous NaOH and 0.1 mL of water. The resulting suspension was dried over $MgSO_4$ and filtered. The filtrate was concentrated under reduced pressure. The residue was used in the next step without purification.

2-Cyclohexyl-4,6-dimethyl-1-(prop-2-yn-1-yl)-1*H*-benzo[*d*]imidazole (S18). To a solution of **S17** in CH_2Cl_2 (10 mL) was added PPh_3 (0.47 g, 1.8 mmol, 1.2 equiv) and NBS (0.32 g,

1.8 mmol, 1.2 equiv). The reaction mixture was stirred at RT for 0.5 h and then concentrated under reduced pressure. The residue was chromatographed through silica gel eluting with ethyl acetate/hexanes to give **S18** as a colorless oil in 39% yield (0.20 g) for three steps. The structure was confirmed by nuclear Overhauser effect (NOE) experiment. ^{1}H NMR (500 MHz, $CDCl_3$) δ 6.89 (s, 1H), 6.87 (s, 1H), 4.45 (t, *J* = 7.4 Hz, 2H), 3.58 (t, *J* = 7.4 Hz, 2H), 2.87–2.79 (m, 1H), 2.62 (s, 3H), 2.44 (s, 3H), 1.97–1.81 (m, 6H), 1.81–1.74 (m, 1H), 1.46–1.36 (m, 3H); ^{13}C NMR (126 MHz, $CDCl_3$) δ 157.7, 140.4, 134.3, 132.2, 129.3, 124.4, 106.4, 44.8, 36.7, 32.1, 28.5, 26.5, 25.8, 21.9, 17.0; IR (neat, cm^{-1}): 2928, 2852, 1618, 1448, 1347, 1231, 891, 826; ESI HRMS *m/z* (M+H)$^+$ calcd 335.1117, obsd 335.1126.

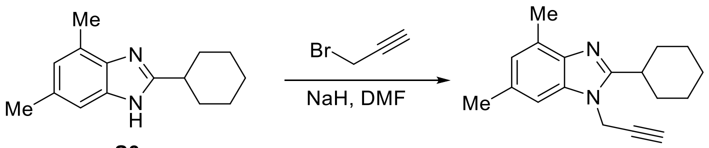

S3 S19

2-Cyclohexyl-4,6-dimethyl-1-(prop-2-yn-1-yl)-1*H*-benzo[*d*]imidazole (S19). The title compound was obtained as a white solid in 71% yield (0.21 g) starting from **S3** (0.26 g, 1.1 mmol, 1.0 equiv), propargyl bromide (0.11 mL, 1.7 mmol, 1.5 equiv) by following the General Procedure A (step 2). The structure was confirmed by nuclear Overhauser effect (NOE) experiment. ^{1}H NMR (500 MHz, $CDCl_3$) δ 7.00 (s, 1H), 6.87 (s, 1H), 4.80 (d, *J* = 2.6 Hz, 2H), 2.84 (tt, *J* = 11.7, 3.5 Hz, 1H), 2.61 (s, 3H), 2.45 (s, 3H), 2.32 (t, *J* = 2.6 Hz, 1H), 2.05–1.98 (m, 2H), 1.93–1.73 (m, 5H), 1.48–1.34 (m, 3H); ^{13}C NMR (126 MHz, $CDCl_3$) δ 157.1, 140.2, 134.6, 132.2, 129.0, 124.3, 106.7, 77.6, 73.2, 36.7, 32.8, 31.7, 26.5, 25.9, 21.9, 16.9; IR (neat, cm^{-1}): 3306, 2928, 2853, 1682, 1620, 1515, 1446, 1346, 1227; ESI HRMS *m/z* (M+H)$^+$ calcd 267.1856, obsd 267.1863.

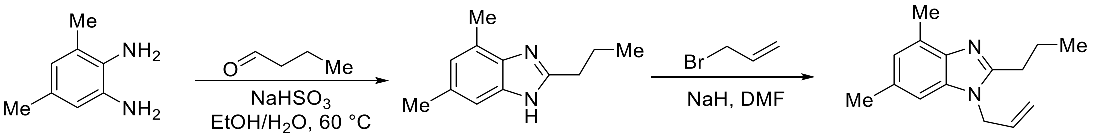

S20 S21

4,6-Dimethyl-2-propyl-1*H*-benzo[*d*]imidazole (S20). The title compound was prepared from 3,5-dimethylbenzene-1,2-diamine (1.5 g, 10 mmol, 1.0 equiv) and butyraldehyde (1.1 mL, 12 mmol, 1.2 equiv) as a white solid in 87% yield (1.6 g) by following the General Procedure A (step 1). ^{1}H NMR (850 MHz, $CDCl_3$) δ 11.20 (brs, 1H), 7.18 (s, 1H), 6.88 (s, 1H), 2.93–2.89 (m, 2H), 2.53 (s, 3H), 2.42 (s, 3H), 1.86 (h, *J* = 7.4 Hz, 2H), 0.95 (t, *J* = 7.4 Hz, 3H); ^{13}C NMR (214 MHz, $CDCl_3$) δ 154.9, 138.3, 136.7, 131.9, 124.7, 124.3, 111.7, 31.4, 22.1, 21.7, 17.4, 14.0; IR (neat, cm^{-1}): 3196, 2968, 2919, 1407, 1218, 997, 841, 735; ESI HRMS *m/z* (M+Na)$^+$ calcd 211.1206, obsd 211.1204.

1-Allyl-4,6-dimethyl-2-propyl-1*H*-benzo[*d*]imidazole (S21). The title compound was prepared from compound **S20** (0.56 g, 3.6 mmol, 1.0 equiv) and allyl bromide (0.31 mL, 3.6 mmol, 1.2 equiv) as a white solid in 63% yield (0.43 g) by following the General Procedure A (step 2). The structure was confirmed by nuclear Overhauser effect (NOE) experiment. ^{1}H NMR (500 MHz, $CDCl_3$) δ 6.86 (s, 2H), 5.90 (ddt, *J* = 17.0, 9.9, 4.8 Hz, 1H), 5.17 (d, *J* = 10.3 Hz, 1H), 4.93 (d, *J* = 17.1 Hz, 1H), 4.63 (dt, *J* = 4.3, 1.9 Hz, 2H), 2.84–2.77 (m, 2H), 2.63 (s, 3H), 2.42 (s, 3H), 1.82 (h, *J* = 7.4 Hz, 2H), 1.03 (t, *J* = 7.4 Hz, 3H); ^{13}C NMR (126 MHz, $CDCl_3$) δ 153.9, 140.0, 135.0, 132.2, 131.7, 128.5, 124.0, 117.0, 107.0, 45.7, 29.6, 22.0, 21.7, 16.8, 14.1; IR (neat, cm^{-1}): 2963, 2930, 1515, 1454, 1326, 1228, 1077, 921, 844, 827; ESI HRMS *m/z* $(M+Na)^+$ calcd 251.1519, obsd 251.1514.

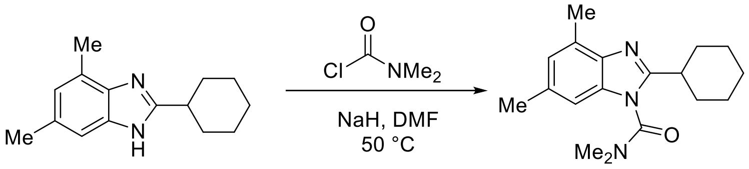

S3 **S22**

2-Cyclohexyl-*N*,*N*,4,6-tetramethyl-1*H*-benzo[*d*]imidazole-1-carboxamide (S22). **S3** (0.26 g, 1.1 mmol, 1.0 equiv) was dissolved in DMF (10 mL) and cooled to 0 °C. NaH (60% dispersion in mineral oil, 0.055 g, 1.4 mmol, 1.2 equiv) was added. The reaction mixture was warmed to ambient temperature and stirred for 15 min. Dimethylcarbamoyl chloride (0.15 mL, 1.6 mmol, 1.5 equiv) was then added dropwise. The reaction mixture was stirred at 50 °C for 0.5 h, quenched with H_2O (50 mL) and extracted with ethyl acetate (50 mL). The organic layer was washed with H_2O (50 mL), dried over $MgSO_4$, filtered and concentrated under reduced pressure. The residue was chromatographed through silica gel eluting with ethyl acetate/hexanes to afford **S22** as a colorless oil in 88% yield (0.30 g). The structure was confirmed by nuclear Overhauser effect (NOE) experiment. ^{1}H NMR (500 MHz, $CDCl_3$) δ 6.88 (s, 1H), 6.81 (s, 1H), 3.24 (brs, 3H), 3.00 (tt, *J* = 11.8, 4.6 Hz, 1H), 2.92 (brs, 3H), 2.61 (s, 3H), 2.42 (s, 3H), 2.16 (s, 1H), 1.91–1.70 (m, 6H), 1.45–1.32 (m, 3H); ^{13}C NMR (126 MHz, $CDCl_3$) δ 157.4, 153.1, 139.8, 133.2, 132.9, 129.4, 125.0, 107.9, 38.6, 37.4, 37.0, 32.2, 31.2, 26.3, 25.9, 21.8, 17.0; IR (neat, cm^{-1}): 2928, 2854, 1698, 1614, 1522, 1450, 1386, 1218, 1149, 835; ESI HRMS *m/z* $(M+Na)^+$ calcd 322.1890, obsd 322.1893.

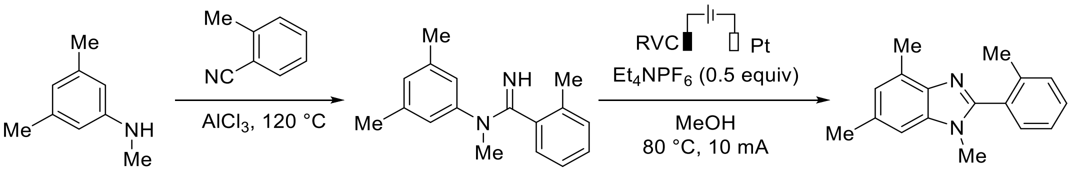

S23 **S24**

N-(3,5-Dimethylphenyl)-N,2-dimethylbenzimidamide (S23). To a pressure tube equipped with a stir bar was added benzonitrile (1.4 mL, 10.0 mmol, 1.0 equiv), $AlCl_3$ (1.5 g, 11.0 mmol, 1.1 equiv) and *N*,3,5-trimethylaniline (1.4 g, 12.0 mmol, 1.2 equiv) under air. The flask was sealed and heated with stirring at 120 °C for 30 min. The reaction mixture was then quenced with ice-water (25 mL) and basified with aqueous NaOH solution (3.0 N) until pH 14. The aqueous layer was extracted with dichloromethane (3 × 30 mL). The combined organic layers were dried over Na_2SO_4 and concentrated under reduced pressure. The residue was purified by silica gel chromatography to give **S23** as a brown oil in 70% yield (1.8 g). ^{1}H NMR (400 MHz, $CDCl_3$) δ 7.18 (dd, J = 7.2, 1.7 Hz, 1H), 7.10–7.02 (m, 2H), 6.99 (dd, J = 7.2, 1.7 Hz, 1H), 6.64 (s, 3H), 3.37 (s, 3H), 2.24 (s, 3H), 2.14 (s, 6H); ^{13}C NMR (101 MHz, $CDCl_3$) δ 167.9, 145.3, 138.8, 138.1, 133.9, 130.0, 128.2, 127.8, 127.0, 125.4, 124.1, 38.9, 21.1, 19.4; IR (neat, cm^{-1}): 3314, 2920, 1580, 1453, 1382, 1132, 1036, 847, 772; ESI HRMS *m/z* $(M+H)^+$ calcd 253.1705, obsd 253.1701.

1,4,6-Trimethyl-2-(*o*-tolyl)-1*H*-benzo[*d*]imidazole (S24). The title compound was was prepared from **S23** (1.2 g, 4.8 mmol) under the standard electrolysis conditions but stopped at half of the theoretical time (3.0 F mol^{-1}). Yield = 25%; White solid; ^{1}H NMR (500 MHz, $CDCl_3$) δ 7.42–7.37 (m, 2H), 7.33 (d, J = 7.6 Hz, 1H), 7.29 (t, J = 7.5 Hz, 1H), 7.03 (s, 1H), 6.97 (s, 1H), 3.56 (s, 3H), 2.68 (s, 3H), 2.51 (s, 3H), 2.24 (s, 3H); ^{13}C NMR (126 MHz, $CDCl_3$) δ 152.7, 140.6, 138.3, 135.6, 132.6, 130.6, 130.6, 130.5, 129.8, 129.6, 125.9, 124.4, 107.0, 30.7, 22.0, 19.9, 16.9; IR (neat, cm^{-1}): 2920, 1460, 1407, 1327, 1218, 1077, 997, 840, 735; ESI HRMS *m/z* $(M+H)^+$ calcd 251.1543, obsd 251.1541.

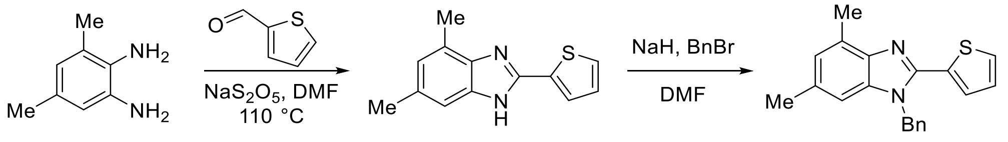

S25 **S26**

4,6-Dimethyl-2-(thiophen-2-yl)-1*H*-benzo[*d*]imidazole (S25). To a solution of 3,5-dimethylbenzene-1,2-diamine (0.27 g, 2.0 mmol, 1.0 equiv) in DMF (6 mL) was added $Na_2S_2O_5$ (0.45 g, 2.4 mmol, 1.2 equiv) and 2-thenaldehyde (0.20 mL, 2.2 mmol, 1.1 equiv). The reaction was stirred at 110 °C for 8 h and then cooled to RT. The reaction mixture was diluted with H_2O (50 mL) and ethyl acetate (50 mL). The layers were separated and organic phase was washed twice with H_2O. The organic layer was dried over $MgSO_4$, filtered and concentrated under reduced pressure to afford **S25**, which was used in the next step without purification.

1-Benzyl-4,6-dimethyl-2-(thiophen-2-yl)-1*H*-benzo[*d*]imidazole (S26). The title compound was obtained as a white solid in 63% yield (0.40 g) from **S25** (2.0 mmol, 1.0 equiv) and BnBr

(0.28 mL, 1.2 mmol, 1.2 equiv) by following the General Procedure A (step 2). The structure was confirmed by nuclear Overhauser effect (NOE) experiment. ^{1}H NMR (500 MHz, $CDCl_3$) δ 7.42 (d, *J* = 5.0 Hz, 1H), 7.34–7.27 (m, 3H), 7.25–7.23 (m, 1H), 7.12–7.07 (m, 2H), 7.04 (dd, *J* = 5.1, 3.6 Hz, 1H), 6.93 (s, 1H), 6.85 (s, 1H), 5.49 (s, 2H), 2.69 (s, 3H), 2.39 (s, 3H); ^{13}C NMR (101 MHz, $CDCl_3$) δ 147.0, 140.8, 136.6, 136.3, 133.3, 132.4, 129.6, 129.2, 128.4, 127.9, 127.8, 126.0, 125.1, 107.4, 48.2, 22.0, 16.8; IR (neat, cm^{-1}): 2918, 1602, 1496, 1452, 1356, 1274, 1232; ESI HRMS *m/z* $(M+H)^+$ calcd 319.1263, obsd 319.1277.

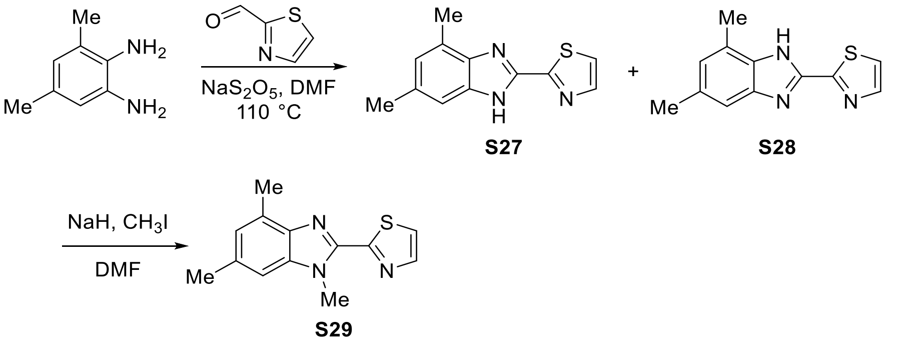

2-(4,6-Dimethyl-1*H*-benzo[*d*]imidazol-2-yl)thiazole (S27) and **2-(5,7-Dimethyl-1*H*-benzo[*d*] imidazol-2-yl)thiazole (S28)**. To a solution of 3,5-dimethylbenzene-1,2-diamine (2.0 g, 15 mmol, 1.0 equiv) in DMF (20 mL) was added $Na_2S_2O_5$ (3.4 g, 18 mmol, 1.2 equiv) and 2-thiazolecarbaldehyde (1.5 mL, 16 mmol, 1.1 equiv). The reaction was stirred at 110 °C for 8 h and then cooled to RT. The resulting mixture was then diluted with H_2O (50 mL) and ethyl acetate (50 mL). The layers were separated and organic phase was washed twice with H_2O. The organic layer was dried over $MgSO_4$, filtered and concentrated under reduced pressure to afford a mixture of **S27** and **S28**, which was used in the next step without purification.

2-(1,4,6-Trimethyl-1*H*-benzo[*d*]imidazol-2-yl)thiazole (S29). The title compound was obtained as a white solid in 47% yield (0.15 g) from **S27** and **S28** (0.30 g, 1.3 mmol, 1.0 equiv) and CH_3I (0.10 mL, 1.6 mmol, 1.2 equiv) by following the General Procedure A (step 2). The structure was confirmed by nuclear Overhauser effect (NOE) experiment. ^{1}H NMR (600 MHz, $CDCl_3$) δ 7.92 (d, *J* = 3.3 Hz, 1H), 7.43 (d, *J* = 3.3 Hz, 1H), 7.01 (s, 1H), 6.94 (s, 1H), 4.23 (s, 3H), 2.66 (s, 3H), 2.47 (s, 3H); ^{13}C NMR (151 MHz, $CDCl_3$) δ 160.6, 144.5, 143.8, 140.6, 137.1, 134.2, 130.0, 125.2, 121.0, 107.3, 32.1, 22.1, 16.8; IR (neat, cm^{-1}): 2921, 1481, 1452, 1319, 1209, 1152, 1014, 837, 777; ESI HRMS *m/z* $(M+Na)^+$ calcd 266.0722, obsd 266.0729.

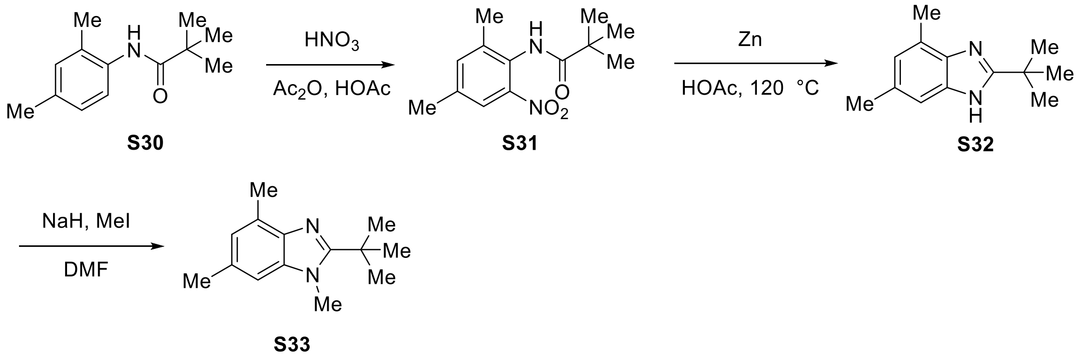

***N*-(2,4-Dimethyl-6-nitrophenyl)pivalamide (S31)**. **S30**[1] (5.0 g, 20.0 mmol, 1.0 equiv) was dissolved in Ac_2O (11.0 mL) and AcOH (1.2 mL). The resulting reaction mixture was cooled to 0 °C and nitric acid (70% aq, 2.6 mL, 2.0 mmol) was slowly added. After complete addition, the reaction mixture was warmed to RT and stirred for 2 h. Ice, aqueous NaOH solution (1.0 M) and ethyl acetate were added to quench the reaction. The layers were separated and the aqueous phase was extracted with ethyl acetate. The combined organic layers were washed with brine, dried over $MgSO_4$ and concentrated under reduced pressure. The residue was chromatographed through silica gel eluting with ethyl acetate/hexanes to give the product **S31** as a white solid (3.56 g, 71% yield). ^{1}H NMR (500 MHz, $CDCl_3$) δ 8.48 (s, 1H), 7.64 (s, 1H), 7.30 (s, 1H), 2.36 (s, 3H), 2.23 (s, 3H), 1.32 (s, 9H); ^{13}C NMR (126 MHz, $CDCl_3$) δ 176.9, 144.7, 138.1, 137.0, 136.5, 128.0, 123.2, 39.7, 27.6, 20.9, 18.9; IR (neat, cm^{-1}): 3294, 2968, 2927, 1659, 1535, 1354, 1178, 863, 766; ESI HRMS *m/z* $(M+Na)^+$ calcd 273.1210, obsd 273.1203.

2-(*tert*-Butyl)-4,6-dimethyl-1*H*-benzo[*d*]imidazole (S32). Zinc dust (4.6 g, 70.0 mmol, 5.0 equiv) was added to a solution of **S31** (3.5 g, 14.0 mmol, 1.0 equiv) in AcOH (30 mL) at RT. The reaction mixture was heated to reflux for 4 h. AcOH was then removed under reduced pressure. The residue was basified with ammonium hydroxide to pH 9. The resulting mixture was extracted with ethyl acetate (3 × 80 mL) and washed with brine. The combined organic solution was dried over anhydrous Na_2SO_4, filtered and evaporated under reduced pressure. The residue was purified by silica gel column chromatography to afford the product **S32** as a white solid (2.3 g, 81% yield). ^{1}H NMR (500 MHz, $CDCl_3$) δ 9.71 (s, 1H), 7.20 (s, 1H), 6.85 (s, 1H), 2.53 (s, 3H), 2.40 (s, 3H), 1.50 (s, 9H); ^{13}C NMR (126 MHz, $CDCl_3$) δ 161.6, 131.8, 124.5, 33.7, 29.8, 21.7, 17.3; IR (neat, cm^{-1}): 3231, 2920, 1407, 1218, 997, 841, 735; ESI HRMS *m/z* $(M+Na)^+$ calcd 225.1362, obsd 225.1362.

2-(*tert*-Butyl)-1,4,6-trimethyl-1*H*-benzo[*d*]imidazole (S33). NaH (0.14 g, 3.6 mmol, 1.2 equiv) was added to a solution of **S32** (0.61 g, 3.0 mmol, 1.0 equiv) in DMF (12 mL) at 0 °C. The resulting mixture was stirred at RT for 30 min. Then MeI (0.23 mL, 3.6 mmol, 1.2 equiv) was added dropwise at –20 °C. The reaction mixture was stirred at RT for another 2 h and

then water (15 mL) was added to quench the reaction. The mixture was extracted with ethyl acetate (3 x 15 mL). The combined organic solution was dried over anhydrous Na_2SO_4, filtrated and evaporated under reduced pressure. The residue was purified by silica gel column chromatography to afford the product **S33** as white solid in 98% yield (0.65 g). The structure was confirmed by nuclear Overhauser effect (NOE) experiment. ^{1}H NMR (500 MHz, $CDCl_3$) δ 6.92 (s, 1H), 6.90 (s, 1H), 3.86 (s, 3H), 2.67 (s, 3H), 2.50 (s, 3H), 1.59 (s, 9H); ^{13}C NMR (126 MHz, $CDCl_3$) δ 159.7, 139.2, 137.4, 131.8, 129.0, 123.8, 106.3, 33.9, 32.4, 29.5, 21.9, 16.6; IR (neat, cm^{-1}): 2921, 1598, 1451, 1363, 1232, 1034, 822, 749, 600; ESI HRMS *m/z* $(M+H)^+$ calcd 239.1519, obsd 239.1516.

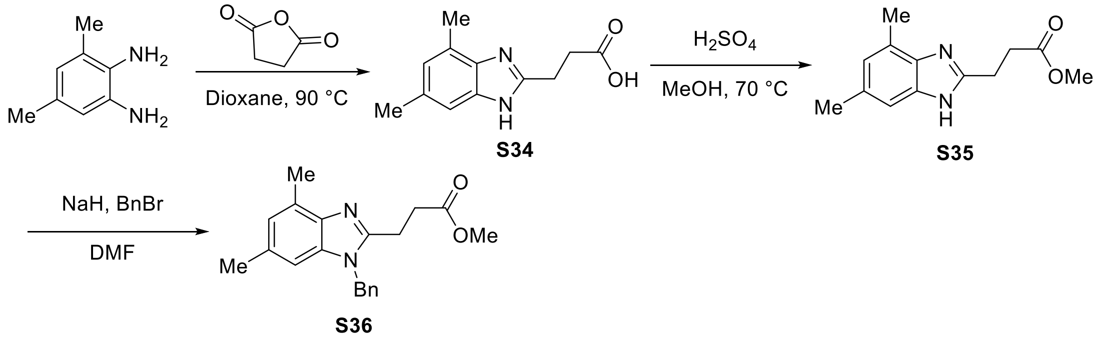

3-(4,6-Dimethyl-1*H*-benzo[*d*]imidazol-2-yl)propanoic acid (S34). A solution of 3,5-dimethylbenzene-1,2-diamine (1.5 g, 11 mmol, 1.0 equiv) and succinic anhydride (1.35 g, 13 mmol, 1.2 equiv) in 1,4-dioxane (20 mL) was heated at 90 °C for 12 h and then cooled to RT. The resulting precipitate was filtered and washed with diethyl ether (50 mL) to give **S34** as a white solid, which was used directly in the next step without further purification.

Methyl 3-(4,6-dimethyl-1*H*-benzo[*d*]imidazol-2-yl)propanoate (S35). To a solution of **S34** (11 mmol, 1.0 equiv) in MeOH (20 mL) was added concentrated H_2SO_4 (1 mL). The reaction mixture was stirred at 70 °C for 3 h and then cooled to RT. The resulting mixture was concentrated under reduced pressure. The residue was diluted with CH_2Cl_2 and washed with saturated $NaHCO_3$. The organic solution was dried over $MgSO_4$, filtered and concentrated under reduced pressure to afford crude **S35**, which was used directly in the next step without further purification.

Methyl 3-(1-benzyl-4,6-dimethyl-1*H*-benzo[*d*]imidazol-2-yl)propanoate (S36). The title compound was obtained as a white solid in 68% yield (2.4 g) from **S35** and BnBr (1.5 mL, 13 mmol, 1.2 equiv) by following the General Procedure A (Step 2). The structure was confirmed by nuclear Overhauser effect (NOE) experiment. ^{1}H NMR (500 MHz, $CDCl_3$) δ 7.31–7.25 (m, 3H), 7.06–7.01 (m, 2H), 6.87 (s, 1H), 6.85 (s, 1H), 5.33 (s, 2H), 3.65 (s, 3H), 3.09 (t, J = 7.5 Hz, 2H), 2.91 (t, J = 7.5 Hz, 2H), 2.62 (s, 3H), 2.39 (s, 3H); ^{13}C NMR (101 MHz, $CDCl_3$) δ 173.2, 152.4, 140.2, 136.5, 135.6, 132.4, 129.1, 128.9, 127.9, 126.3, 124.3,

107.1, 51.9, 46.9, 32.0, 22.7, 21.9, 16.7; IR (neat, cm^{-1}): 2919, 1738, 1602, 1517, 1435, 1363, 1230, 1168; ESI HRMS *m/z* $(M+H)^+$ calcd 323.1754, obsd 323.1765.

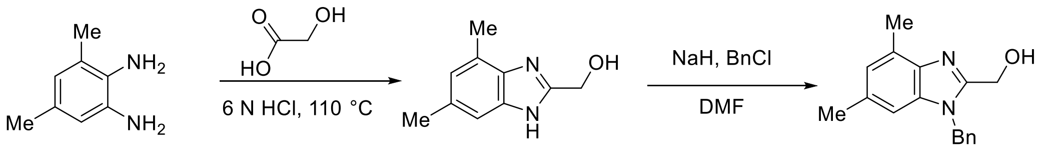

S37 S38

(4,6-Dimethyl-1*H*-benzo[*d*]imidazol-2-yl)methanol (S37). A suspension of 3,5-dimethylbenzene-1,2-diamine (1.7 g, 12 mmol, 1.0 equiv) and glycolic acid (2.7 g, 36 mmol, 3.0 equiv) in 6 N HCl (10 mL) was heated to 110 °C for 8 h. The resulting reaction mixture was cooled to RT and basified with aqueous NaOH solution (3.0 N) until pH 10. The suspension was filtered and washed with Et_2O. The resulting solid was dried to afford **S37** as a white solid, which was used directly in the next step without further purification.

1-(1-Benzyl-4,6-dimethyl-1*H*-benzo[*d*]imidazol-2-yl)ethan-1-ol (S38). The title compound was obtained as a white solid in 73% yield (2.2 g) starting from **S37** (12 mmol, 1.0 equiv) and BnCl (1.5 mL, 13 mmol, 1.1 equiv) by following the General Procedure A (Step 2). The structure was confirmed by nuclear overhauser effect (NOE) experiment. ^1H NMR (500 MHz, $CDCl_3$) δ 7.29–7.20 (m, 3H), 7.10–7.03 (m, 2H), 6.86 (s, 1H), 6.80 (s, 1H), 5.43 (s, 2H), 4.85 (s, 2H), 2.56 (s, 3H), 2.36 (s, 3H); ^{13}C NMR (101 MHz, $CDCl_3$) δ 153.1, 139.1, 136.3, 135.5, 133.3, 129.2, 129.0, 127.9, 126.5, 124.8, 107.6, 57.1, 47.2, 21.9, 16.8; IR (neat, cm^{-1}): 2920, 2851, 1634, 1453, 1217, 1037; ESI HRMS *m/z* $(M+H)^+$ calcd 267.1492, obsd 267.1496.

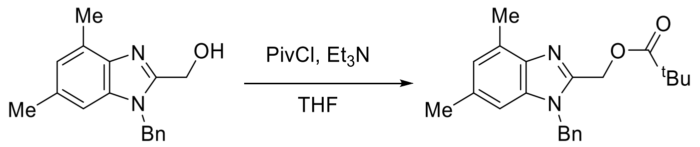

S38 S39

(1-Benzyl-4,6-dimethyl-1*H*-benzo[*d*]imidazol-2-yl)methyl pivalate (S39). To a solution of **S38** (0.20 g, 0.75 mmol, 1.0 equiv) in THF (10 mL) was added pivaloyl chloride (0.11 mL, 0.90 mmol, 1.2 equiv) and Et_3N (0.15 mL, 1.1 mmol, 1.5 equiv). The reaction mixture was stirred at RT for 1 h and the solvent was then removed under revduced pressure. The residue was chromatographed through silica gel eluting with ethyl acetate/hexanes to give **S39** as a white solid in 84% yield (0.22 g). ^1H NMR (500 MHz, $CDCl_3$) δ 7.31–7.24 (m, 3H), 7.05–7.00 (m, 2H), 6.93 (s, 1H), 6.87 (s, 1H), 5.38 (s, 2H), 5.31 (s, 2H), 2.66 (s, 3H), 2.39 (s, 3H), 1.08 (s, 9H); ^{13}C NMR (101 MHz, $CDCl_3$) δ 177.9, 147.7, 140.1, 136.1, 135.8, 133.8, 129.9, 129.1, 128.0, 126.2, 125.0, 107.6, 59.1, 47.3, 39.0, 27.2, 22.0, 16.8; IR (neat, cm^{-1}): 2918, 1727, 1601, 1539, 1452, 1423, 1276, 1141; ESI HRMS *m/z* $(M+H)^+$ calcd 351.2067, obsd 351.2071.

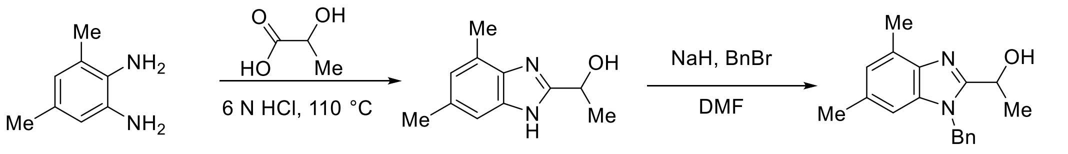

S40 **S41**

1-(1-Benzyl-4,6-dimethyl-1*H*-benzo[*d*]imidazol-2-yl)ethan-1-ol (S41). The title compound was obtained as a white solid in 73% yield (1.0 g) for two steps from 3,5-dimethylbenzene-1,2-diamine (0.68 g, 5.0 mmol, 1.0 equiv), lactic acid (1.4 g, 15 mmol, 3.0 equiv) and BnBr (0.71 mL, 6.0 mmol, 1.2 equiv) by following the procedure described for the synthesis of **S38**. The structure was confirmed by nuclear Overhauser effect (NOE) experiment. ^{1}H NMR (500 MHz, $CDCl_3$) δ 7.24–7.20 (m, 3H), 6.98–6.92 (m, 2H), 6.87 (s, 1H), 6.68 (s, 1H), 5.42, 5.28 (ABq, J_{AB} = 16.9 Hz, 2H), 5.11 (q, J = 6.7 Hz, 1H), 4.73 (brs, 1H), 2.61 (s, 3H), 2.35 (s, 3H), 1.53 (d, J = 6.7 Hz, 3H); ^{13}C NMR (101 MHz, $CDCl_3$) δ 155.8, 136.5, 136.0, 133.0, 129.2, 129.0, 127.7, 126.1, 124.7, 107.6, 64.3, 47.1, 22.8, 21.9, 16.8; IR (neat, cm^{-1}): 3206, 2979, 2920, 1603, 1425, 1318, 1230, 1112, 1028, 827, 733; ESI HRMS *m/z* $(M+H)^+$ calcd 281.1648, obsd 281.1654.

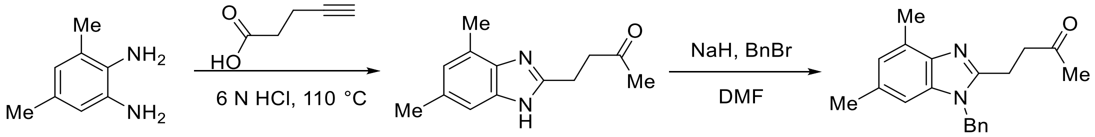

S42 **S43**

4-(1-Benzyl-4,6-dimethyl-1*H*-benzo[*d*]imidazol-2-yl)butan-2-one (S43). The title compound was obtained as a white solid in 49% yield (0.30 g) for two steps from 3,5-dimethylbenzene-1,2-diamine (0.27 g, 2.0 mmol, 1.0 equiv), 4-pentynoic acid (0.59 g, 6.0 mmol, 3.0 equiv) and BnBr (0.28 mL, 2.4 mmol, 1.2 equiv) by following the procedure described for the synthesis of **S38**. The structure was confirmed by nuclear Overhauser effect (NOE) experiment. ^{1}H NMR (500 MHz, $CDCl_3$) δ 7.32–7.22 (m, 3H), 7.07–7.01 (m, 2H), 6.87 (s, 1H), 6.86 (s, 1H), 5.32 (s, 2H), 3.06–3.01 (m, 4H), 2.62 (s, 3H), 2.39 (s, 3H), 2.17 (s, 3H); ^{13}C NMR (126 MHz, $CDCl_3$) δ 207.5, 153.0, 140.1, 136.5, 135.6, 132.3, 129.1, 128.7, 127.9, 126.3, 124.3, 107.1, 46.9, 41.0, 30.2, 21.8, 21.4, 16.7; IR (neat, cm^{-1}): 2917, 2850, 1714, 1646, 1539, 1516, 1453, 1417; ESI HRMS *m/z* $(M+H)^+$ calcd 307.1805, obsd 307.1806.

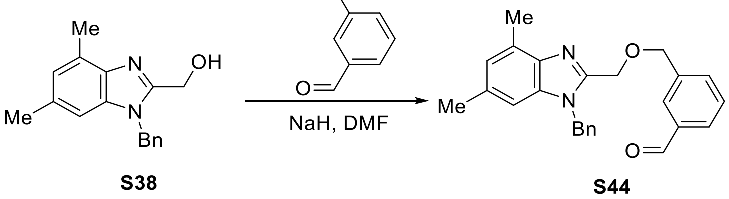

S38 **S44**

3-(((1-Benzyl-4,6-dimethyl-1*H*-benzo[*d*]imidazol-2-yl)methoxy)methyl)benzaldehyde

(S44). To a solution of **S38** (0.20 g, 0.75 mmol, 1.0 equiv) in DMF (10 mL) was added NaH (60% dispersion in mineral oil, 0.045 g, 1.1 mmol, 1.5 equiv). The reaction mixture was stirred at RT for 15 min before addition of 3-(bromomethyl)benzaldehyde (0.18 g, 0.90 mmol, 1.2 equiv). The reaction mixture was stirred at RT for 15 min and quenched with H_2O (50 mL). The resulting mixture was extracted with ethyl acetate (50 mL) and the organic layer was washed with H_2O (50 mL). The organic solution was dried over $MgSO_4$, filtered, and concentrated under reduced pressure. The residue was chromatographed through silica gel eluting with ethyl acetate/hexanes to afford **S44** as a white solid in 76% yield (0.22 g). ^{1}H NMR (400 MHz, $CDCl_3$) δ 9.91 (s, 1H), 7.78–7.74 (m, 1H), 7.71–7.69 (m, 1H), 7.48–7.41 (m, 2H), 7.27–7.22 (m, 3H), 7.07–7.02 (m, 2H), 6.92 (s, 1H), 6.88 (s, 1H), 5.43 (s, 2H), 4.85 (s, 2H), 4.60 (s, 2H), 2.66 (s, 3H), 2.40 (s, 3H); ^{13}C NMR (101 MHz, $CDCl_3$) δ 192.2, 149.1, 139.9, 138.8, 136.7, 136.5, 136.0, 133.7, 133.6, 129.8, 129.2, 129.1, 129.0 (2s), 127.8, 126.4, 124.8, 107.5, 71.8, 65.8, 47.4, 22.0, 16.9; IR (neat, cm^{-1}): 2923, 2853, 1698, 1605, 1453, 1356, 1229, 1142, 1076; ESI HRMS *m/z* $(M+Na)^+$ calcd 407.1730, obsd 407.1731.

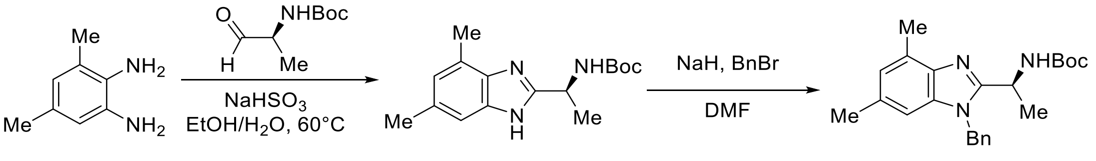

***tert*-Butyl (*S*)-(1-(1-benzyl-4,6-dimethyl-1*H*-benzo[*d*]imidazol-2-yl)ethyl)carbamate (S46)**. The title compound was obtained as a white solid in 53% yield (0.30 g) for two steps from 3,5-dimethylbenzene-1,2-diamine (0.27 g, 2.0 mmol, 1.0 equiv), Boc-*L*-alaninal (0.42 g, 2.4 mmol, 1.2 equiv) and BnBr (0.28 mL, 2.4 mmol, 1.2 equiv) by following the General Procedure A. The structure was confirmed by nuclear Overhauser effect (NOE) experiment. ^{1}H NMR (600 MHz, $CDCl_3$) δ 7.30–7.23 (m, 3H), 7.08–7.02 (m, 2H), 6.89 (s, 1H), 6.86 (s, 1H), 5.62 (d, J = 8.4 Hz, 1H), 5.45, 5.37 (ABq, J_{AB} = 16.9 Hz, 2H), 5.10–5.04 (m, 1H), 2.63 (s, 3H), 2.38 (s, 3H), 1.48 (d, J = 6.8 Hz, 3H), 1.38 (s, 9H); ^{13}C NMR (101 MHz, $CDCl_3$) δ 155.2, 154.5, 140.0, 136.5, 135.5, 132.9, 129.3, 129.0, 127.8, 126.3, 124.6, 107.5, 79.7, 47.0, 43.2, 28.5, 21.9, 21.3, 16.8; IR (neat, cm^{-1}): 3348, 2920, 1678, 1524, 1453, 1332, 1250, 1170, 1061; ESI HRMS *m/z* $(M+H)^+$ calcd 381.2333, obsd 380.2343.

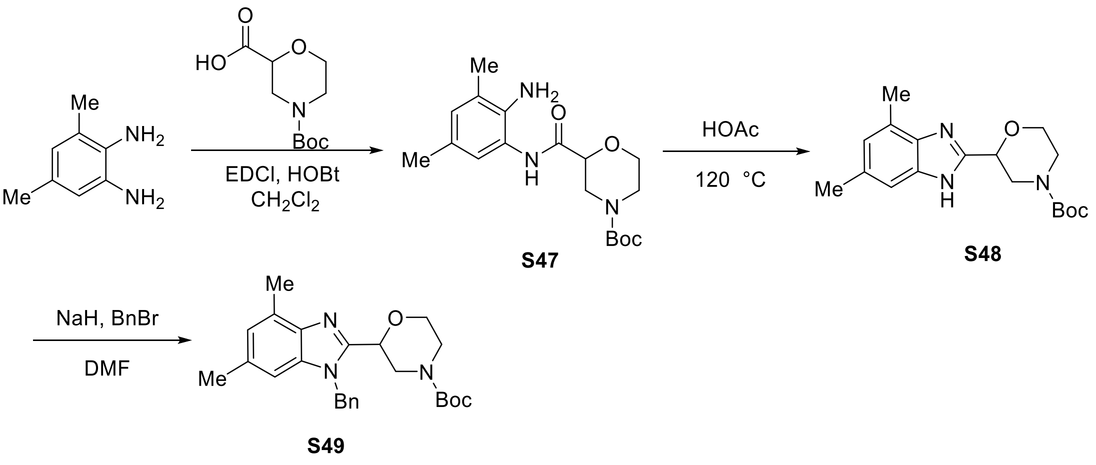

***tert*-Butyl 2-((2-amino-3,5-dimethylphenyl)carbamoyl)morpholine-4-carboxylate (S47)**. To a solution of 4-Boc-2-morpholinecarboxylic acid (0.46 g, 2.0 mmol, 1.0 equiv) and EDCl•HCl (0.42 g, 2.2 mmol, 1.1 equiv) in CH_2Cl_2 (10 mL) was added HOBt (0.30 g, 2.2 mmol, 1.1 equiv), followed by 3,5-dimethylbenzene-1,2-diamine (0.27 g, 2.0 mmol, 1.0 equiv). The resulting reaction mixture was stirred at RT for 0.5 h, diluted with CH_2Cl_2 and saturated $NaHCO_3$. The layers were separated and the aqueous layer was extracted twice with CH_2Cl_2. The combined organic solution was dried over $MgSO_4$ and concentrated under reduced pressure to afford **S47** as a white solid, which was used directly in the next step without further purification.

***tert*-Butyl 2-(4,6-dimethyl-1*H*-benzo[*d*]imidazol-2-yl)morpholine-4-carboxylate (S48)**. The crude **S47** was dissolved in HOAc (6 mL) and heated at 120 °C for 0.5 h. The resulting reaction mixture was cooled to RT, concentrated and basified with aqueous NaOH solution (3.0 N) until pH 10. The aqueous mixture was then extracted twice with CH_2Cl_2. The combined organic layers were dried over $MgSO_4$ and concentrated under reduced pressure. The crude product **S48** was used directly in the next step without further purification.

***tert*-Butyl 2-(1-benzyl-4,6-dimethyl-1*H*-benzo[*d*]imidazol-2-yl)morpholine-4-carboxylate (S49)**. The title compound was obtained as a white solid in 47% yield (0.40 g) from **S48** and BnBr (0.28 mL, 2.4 mmol, 1.2 equiv) by following the General Procedure A (Step 2). The structure was confirmed by nuclear Overhauser effect (NOE) experiment. ^{1}H NMR (600 MHz, $CDCl_3$) δ 7.32–7.25 (m, 3H), 7.10–7.06 (m, 2H), 6.89 (s, 1H), 6.84 (s, 1H), 5.47, 5.42 (ABq, J_{AB} = 16.4 Hz, 2H), 4.62 (dd, *J* = 10.5, 2.9 Hz, 1H), 4.24 (brs, 1H), 4.03–3.85 (m, 2H), 3.61 (t, *J* = 10.9 Hz, 1H), 3.52 (t, *J* = 12.3 Hz, 1H), 3.05 (brs, 1H), 2.64 (s, 3H), 2.37 (s, 3H), 1.45 (s, 9H); ^{13}C NMR (151 MHz, $CDCl_3$) δ 154.8, 149.2, 140.0, 136.6, 135.7, 133.3, 129.9, 129.0, 127.8, 126.5, 124.7, 107.4, 80.4, 71.5, 66.9, 47.5, 47.0, 42.9, 28.5, 22.0, 16.8; IR (neat, cm^{-1}): 2920, 1698, 1453, 1416, 1365, 1251, 1168, 1125, 1087; ESI HRMS *m/z* $(M+H)^+$ calcd

422.2438, obsd 422.2447.

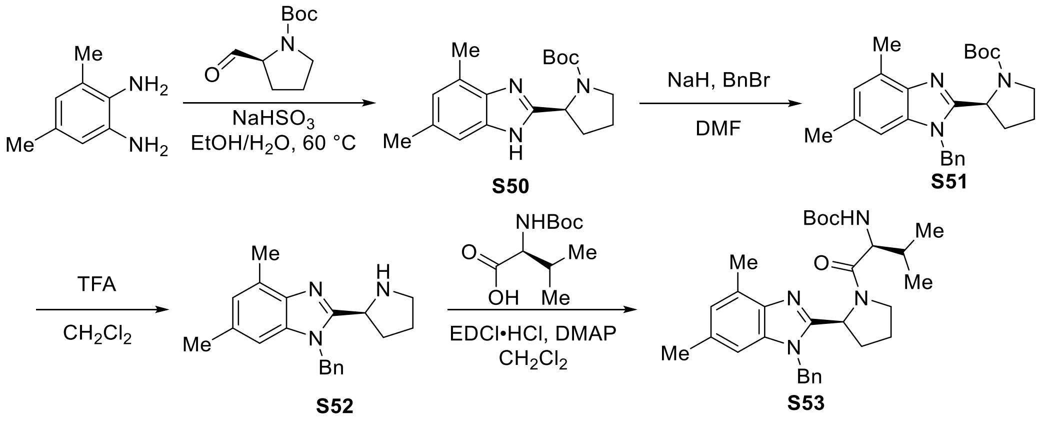

***tert*-Butyl (*S*)-2-(1-benzyl-4,6-dimethyl-1*H*-benzo[*d*]imidazol-2-yl)pyrrolidine-1-carboxyl -ate (S51)**. The title compound was obtained as a 3:2 mixture of rotary isomers in 69% yield (0.84 g) for two steps from 3,5-dimethylbenzene-1,2-diamine (0.68 g, 5.0 mmol, 1.0 equiv), *N*-Boc-*L*-valine (1.3 g, 6.0 mmol, 1.2 equiv) and BnBr (0.71 mL, 6.0 mmol, 1.2 equiv) by following the General Procedure A. The structure was confirmed by nuclear Overhauser effect (NOE) experiment. ^{1}H NMR (500 MHz, $CDCl_3$) δ 7.31–7.21 (m, 3H), 7.09–7.02 (m, 2H), 6.92–6.81 (m, 2H), 5.74 (d, *J* = 17.2 Hz, 0.4H), 5.49–5.25 (m, 1.6H), 5.08–4.91 (m, 1H), 3.77–3.44 (m, 2H), 2.87 (s, 0.4H), 2.61 (s, 3H), 2.38 (s, 3H), 2.47–2.34 (m, 0.6 H), 2.14–1.94 (m, 2H), 1.91–1.78 (m, 1H), 1.39 (s, 4H), 1.15 (s, 5H); ^{13}C NMR (126 MHz, $CDCl_3$) δ 154.8, 154.4, 140.3, 140.0, 137.4, 136.7, 135.5, 132.3, 132.2, 129.3, 129.2, 129.1, 128.9, 128.5, 127.9, 127.5, 127.0, 126.3, 125.4, 124.3, 124.1, 107.2, 106.8, 79.8, 79.6, 54.6, 53.2, 47.1, 47.0, 33.3, 28.6, 28.3, 24.7, 23.7, 21.9, 16.8; IR (neat, cm^{-1}): 2975, 2926, 1694, 1514, 1455, 1394, 1164, 826, 740; ESI HRMS *m/z* $(M+H)^+$ calcd 406.2489, obsd 406.2499.

(*S*)-1-Benzyl-4,6-dimethyl-2-(pyrrolidin-2-yl)-1*H*-benzo[*d*]imidazole (S52). A solution of **S51** (0.13 g, 4.0 mmol, 1.0 equiv) in CH_2Cl_2 (10 mL) and TFA (2 mL) was stirred at RT for 2 h and concentrated under reduced pressure. The residue was diluted with CH_2Cl_2 and washed with saturated $NaHCO_3$. The organic solution was dried over $MgSO_4$, filtered and concentrated under reduced pressure to afford **S52** as a white solid, which was used directly in the next step without further purification.

((*S*)-1-((*S*)-2-(1-Benzyl-4,6-dimethyl-1*H*-benzo[*d*]imidazol-2-yl)pyrrolidin-1-yl)-3-methyl -1-oxobutan-2-yl)carbamate (S53). The title compound was obtained as a white solid in 34% yield (0.68 g) starting from **S52** (0.61 g, 2.0 mmol, 1.0 equiv) and *N*-Boc-*L*-valine (0.52 g, 2.4 mmol, 1.2 equiv) by following the the procedure described for the synthesis of **S5**. ^{1}H NMR (500 MHz, $CDCl_3$) δ 7.33–7.22 (m, 3H), 7.14–7.08 (m, 2H), 6.88 (s, 1H), 6.84 (s, 1H), 5.61 (d, *J* = 17.0 Hz, 1H), 5.41 (d, *J* = 17.0 Hz, 1H), 5.19 (d, *J* = 9.3 Hz, 1H), 5.14 (dd, *J* = 7.5, 5.4

Hz, 1H), 4.32 (dd, *J* = 9.4, 6.3 Hz, 1H), 3.99–3.91 (m, 1H), 3.91–3.82 (m, 1H), 2.54 (s, 3H), 2.38 (s, 3H), 2.27 (d, *J* = 17.5 Hz, 1H), 2.06–1.87 (m, 4H), 1.42 (s, 9H), 0.98 (d, *J* = 6.8 Hz, 3H), 0.80 (d, *J* = 6.7 Hz, 3H); ^{13}C NMR (101 MHz, $CDCl_3$) δ 171.1, 156.0, 154.1, 140.3, 137.3, 135.3, 132.3, 129.2, 129.0, 127.8, 126.4, 124.1, 107.1, 79.4, 57.1, 53.3, 47.6, 47.0, 31.6, 31.5, 28.5, 25.2, 21.9, 19.4, 17.7, 16.6; ESI HRMS *m/z* $(M+H)^+$ calcd 505.3173, obsd 505.3190.

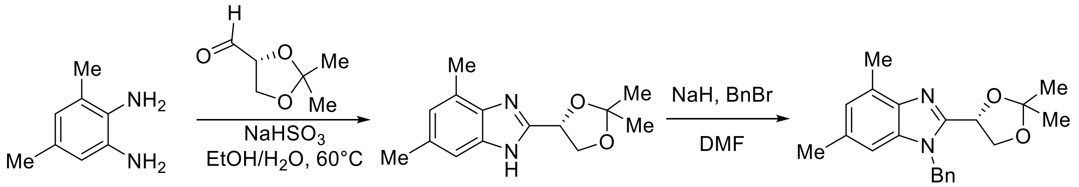

S54 S55

(*S*)-1-Benzyl-2-(2,2-dimethyl-1,3-dioxolan-4-yl)-4,6-dimethyl-1*H*-benzo[*d*]imidazole (S55). The title compound was obtained as a white solid in 95% yield (0.64 g) for two steps from 3,5-dimethylbenzene-1,2-diamine (0.27 g, 2.0 mmol, 1.0 equiv), (*R*)-glyceraldehyde acetonide (0.31 g, 2.4 mmol, 1.2 equiv) and BnBr (0.28 mL, 2.4 mmol, 1.2 equiv) by following the General Procedure A. The structure was confirmed by nuclear Overhauser effect (NOE) experiment. ^{1}H NMR (500 MHz, $CDCl_3$) δ 7.30–7.20 (m, 3H), 7.11–7.04 (m, 2H), 6.88 (s, 1H), 6.83 (s, 1H), 5.55, 5.43 (ABq, J_{AB} = 16.5 Hz, 2H), 5.29 (t, *J* = 6.9 Hz, 1H), 4.67 (t, *J* = 7.6 Hz, 1H), 4.37 (t, *J* = 7.6 Hz, 1H), 2.63 (s, 3H), 2.36 (s, 3H), 1.42 (s, 3H), 1.35 (s, 3H); ^{13}C NMR (151 MHz, $CDCl_3$) δ 150.0, 139.8, 136.7, 136.2, 133.2, 129.7, 128.8, 127.7, 126.5, 124.6, 110.6, 107.4, 71.6, 67.7, 47.4, 26.2, 25.4, 21.9, 16.7; IR (neat, cm^{-1}): 2988, 2924, 1619, 1515, 1452, 1424, 1369, 1210, 1151, 1050, 839; ESI HRMS *m/z* $(M+H)^+$ calcd 337.1911, obsd 337.1915.

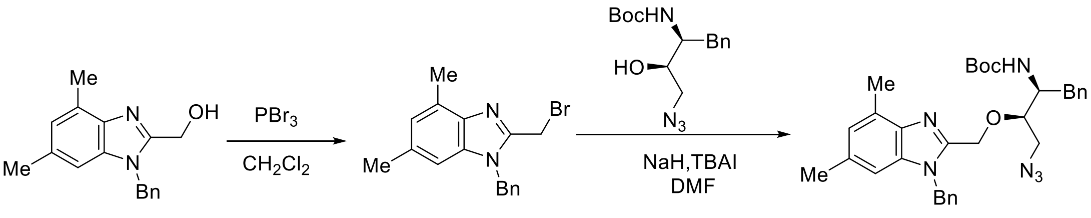

S38 S56 S57

1-Benzyl-2-(bromomethyl)-4,6-dimethyl-1*H*-benzo[*d*]imidazole (S56). To a solution of **S38** (0.30 g, 1.1 mmol, 1.0 equiv) in CH_2Cl_2 (10 mL) was added PBr_3 (0.11 mL, 1.2 mmol, 1.1 equiv). The reaction mixture was stirred at RT for 0.5 h and quenced with with saturated $NaHCO_3$. The layers were separated and the aqueous layer was extracted twice with CH_2Cl_2. The combined organic layers were dried over $MgSO_4$ and concentrated under reduced pressure to afford **S56** as a white solid in 89% yield (0.33 g), which was used directly in the next step without further purification.

***tert*-Butyl ((2*S*,3*R*)-4-azido-3-((1-benzyl-4,6-dimethyl-1*H*-benzo[*d*]imidazol-2-yl)methoxy)-1-phenylbutan-2-yl)carbamate (S57)**. To a solution of *tert*-butyl ((2*S*,3*R*)-4-azido-3-hydroxy-1-phenylbutan-2-yl)carbamate (0.15 g, 0.50 mmol, 1.0 equiv) in DMF (5 mL) at 0 °C was added NaH (60% dispersion in mineral oil, 0.024 g, 0.60 mmol, 1.2 equiv). The reaction mixture was warmed to ambient temperature and stirred for 15 min. **S56** (0.25 g, 0.75 mmol, 1.5 equiv) was then added, followed by TBAI (0.018 g, 0.049 mmol, 0.10 equiv). The reaction mixture was stirred at RT for 12 h, quenched with H_2O (50 mL), and extracted with ethyl acetate (50 mL). The organic layer was washed with H_2O (50 mL), dried over $MgSO_4$, filtered and concentrated under reduced pressure. The residue was chromatographed through silica gel eluting with ethyl acetate/hexanes to afford **S57** as a white solid in 54% yield (0.15 g). ^{1}H NMR (500 MHz, $CDCl_3$) δ 7.33–7.26 (m, 3H), 7.23–7.14 (m, 3H), 7.10–7.06 (m, 2H), 7.04–7.00 (m, 2H), 6.94 (s, 1H), 6.90 (s, 1H), 5.52–5.43 (m, 2H), 5.38–5.28 (m, 1H), 4.99 (d, J = 12.7 Hz, 1H), 4.85 (d, J = 12.7 Hz, 1H), 3.96 (t, J = 8.1 Hz, 1H), 3.76 (s, 1H), 3.42–3.29 (m, 2H), 2.74 (dd, J = 14.1, 5.0 Hz, 1H), 2.67 (s, 3H), 2.49 (dd, J = 14.1, 9.6 Hz, 1H), 2.40 (s, 3H), 1.30 (s, 9H); ^{13}C NMR (126 MHz, $CDCl_3$) δ 155.6, 149.4, 139.8, 137.8, 136.5, 135.8, 133.7, 129.8, 129.4, 129.1, 128.5, 128.0, 126.5, 126.4, 124.9, 107.6, 81.5, 79.5, 65.9, 53.3, 52.3, 47.3, 36.0, 28.4, 22.0, 16.9; IR (neat, cm^{-1}): 3419, 2924, 2101, 1706, 1497, 1454, 1365, 1248, 1169, 1048, 699; ESI HRMS *m/z* (M+Na)$^+$ calcd 577.2898, obsd 577.2911.

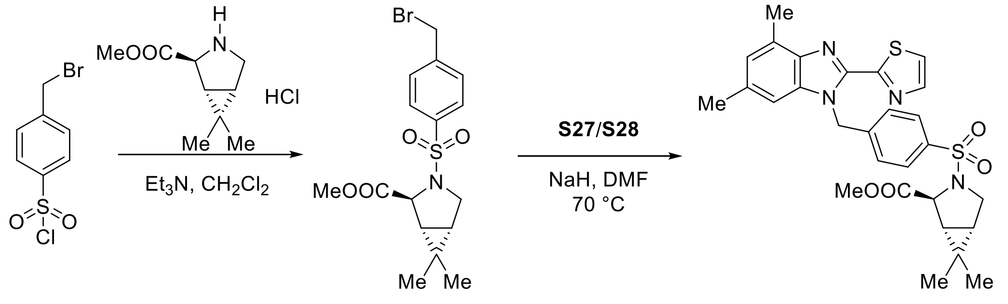

S58 **S59**

Methyl (1*R*,2*S*,5*S*)-3-((4-(bromomethyl)phenyl)sulfonyl)-6,6-dimethyl-3-azabicyclo[3.1.0]hexane-2-carboxylate (S58). To a solution of methyl (1*R*,2*S*,5*S*)-6,6-dimethyl-3-azabicyclo[3.1.0]hexane-2-carboxylate hydrochloride (0.41 g, 2.0 mmol, 1.0 equiv) in CH_2Cl_2 (10 mL) was 4-bromomethylbenzenesulfonyl chloride (0.64 g, 2.4 mmol, 1.2 equiv) and Et_3N (0.67 mL, 4.8 mmol, 2.4 equiv). The reaction mixture was stirred at RT for 1 h, diluted with CH_2Cl_2 and washed with saturated $NaHCO_3$. The layers were separated and the aqueous layer was extracted twice with CH_2Cl_2. The combined organic layers were dried over $MgSO_4$ and concentrated under reduced pressure to afford **S58** as a white solid, which was used directly in the next step without further purification.

methyl (1*R*,2*S*,5*S*)-3-((4-((4,6-dimethyl-2-(thiazol-2-yl)-1*H*-benzo[*d*]imidazol-1-yl)methyl)

phenyl)sulfonyl)-6,6-dimethyl-3-azabicyclo[3.1.0]hexane-2-carboxylate (S59). **S27** and **S28** (0.20 g, 0.87 mmol, 1.0 equiv) was dissolved in DMF (10 mL) and cooled to 0 °C. NaH (60% dispersion in mineral oil, 0.052 g, 1.3 mmol, 1.5 equiv) was added. The reaction mixture was warmed to ambient temperature and stirred for 15 min. **S58** (0.40 g, 1.0 mmol, 1.1 equiv) was then added. After being stirred at 70 °C for 2 h, H_2O (50 mL) was added to quench the reaction. The resulting mixture was extracted with ethyl acetate (50 mL). The organic layer was washed with H_2O (50 mL), dried over $MgSO_4$, filtered and concentrated under reduced pressure. The residue was chromatographed through silica gel eluting with ethyl acetate/hexanes to afford **S59** as a white solid in 67% yield (0.32 g). The structure was confirmed by nuclear Overhauser effect (NOE) experiment. ^{1}H NMR (600 MHz, $CDCl_3$) δ 7.83 (d, J = 3.2 Hz, 1H), 7.73–7.69 (m, 2H), 7.44 (d, J = 3.2 Hz, 1H), 7.26 (d, J = 8.4 Hz, 2H), 6.95 (s, 1H), 6.85 (s, 1H), 6.16, 6.13 (ABq, J_{AB} = 16.8 Hz, 2H), 4.22 (s, 1H), 3.57 (dd, J = 9.3, 5.2 Hz, 1H), 3.50 (s, 3H), 3.37 (d, J = 9.2 Hz, 1H), 2.68 (s, 3H), 2.40 (s, 3H), 1.39 (dd, J = 7.5, 5.1 Hz, 1H), 1.34 (d, J = 7.5 Hz, 1H), 0.95 (s, 3H), 0.73 (s, 3H); ^{13}C NMR (151 MHz, $CDCl_3$) δ 172.3, 160.0, 144.1, 143.9, 142.3, 140.7, 138.6, 136.3, 134.8, 130.4, 127.7, 127.3, 125.8, 121.4, 107.5, 61.1, 52.3, 48.1, 47.8, 32.6, 27.8, 26.6, 22.1, 20.2, 16.7, 12.2; IR (neat, cm^{-1}): 2951, 2923, 1743, 1600, 1451, 1345, 1061, 734, 687; ESI HRMS m/z (M+Na)$^+$ calcd 573.1601, obsd 573.1612.

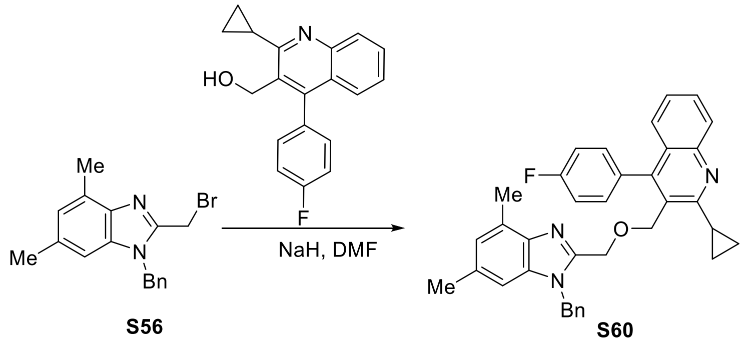

S56 **S60**

3-(((1-Benzyl-4,6-dimethyl-1*H*-benzo[*d*]imidazol-2-yl)methoxy)methyl)-2-cyclopropyl-4-(4-fluorophenyl)quinolone (S60). Cyclopropyl-4-(4-fluorophenyl)-3-quinolinemethanol (0.15 g, 0.50 mmol, 1.1 equiv) was dissolved in DMF (5 mL) and cooled to 0 °C. NaH (60% dispersion in mineral oil, 0.022 g, 0.55 mmol, 1.2 equiv) was added. The reaction mixture was warmed to ambient temperature and stirred for 15 min. **S56** (0.15 g, 0.46 mmol, 1.0 equiv) was then added. After being stirred at RT for 15 min, H_2O (50 mL) was added to quench the reaction. The resulting mixture was extracted with ethyl acetate (50 mL) and the organic layer was washed with H_2O (50 mL). The organic solution was dried over $MgSO_4$, filtered and concentrated under reduced pressure. The residue was chromatographed through silica gel eluting with ethyl acetate/hexanes to afford **S60** as a white solid in 96% yield (0.24 g). ^{1}H

NMR (500 MHz, $CDCl_3$) δ 7.96 (d, *J* = 8.4 Hz, 1H), 7.60 (ddd, *J* = 8.4, 6.7, 1.6 Hz, 1H), 7.31–7.27 (m, 1H), 7.24–7.21 (m, 1H), 7.19–7.11 (m, 3H), 7.10–7.06 (m, 2H), 6.99–6.94 (m, 2H), 6.93–6.87 (m, 3H), 6.86 (s, 1H), 5.33 (s, 2H), 4.65 (s, 2H), 4.62 (s, 2H), 2.61 (s, 3H), 2.45–2.41 (m, 1H), 2.40 (s, 3H), 1.34–1.28 (m, 2H), 0.98–0.92 (m, 2H); ^{13}C NMR (126 MHz, $CDCl_3$) δ 162.6 (d, $J_{C\text{-}F}$ = 247.8 Hz), 162.9, 149.1, 147.7, 147.4, 139.8, 136.4, 135.9, 133.5, 132.3 (d, $J_{C\text{-}F}$ = 3.5 Hz), 131.4 (d, $J_{C\text{-}F}$ = 7.9 Hz), 129.7, 129.4, 129.0, 128.9, 127.8, 126.6, 126.3, 126.2, 125.5, 124.8, 115.4 (d, $J_{C\text{-}F}$ = 21.8 Hz), 107.3, 67.3, 65.5, 47.2, 22.0, 16.8, 14.6, 10.3; ^{19}F NMR (471 MHz, $CDCl_3$) δ −113.4–−113.6 (m, 1F); IR (neat, cm^{-1}): 2922, 2855, 1605, 1513, 1453, 1421, 1225, 1072, 1028, 840, 730; ESI HRMS *m/z* $(M+H)^+$ calcd 542.2602, obsd 542.2613.

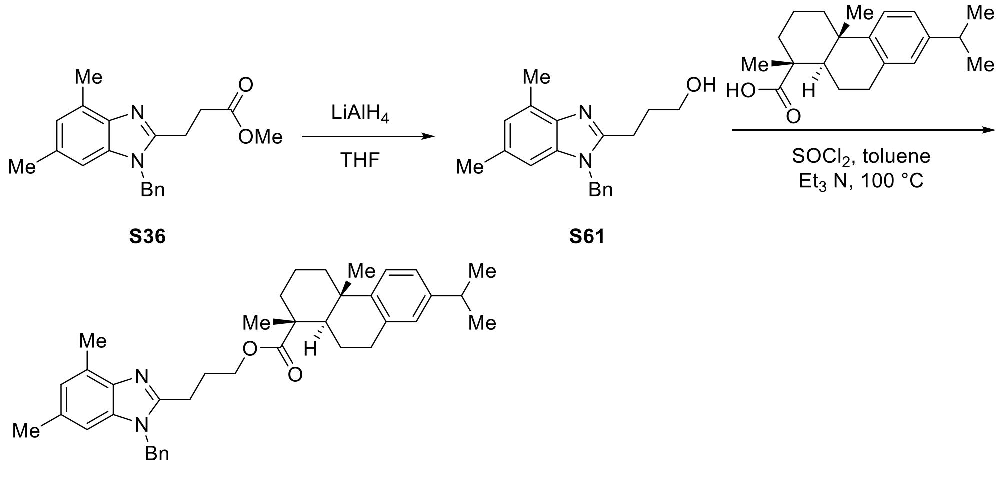

3-(1-Benzyl-4,6-dimethyl-1*H*-benzo[*d*]imidazol-2-yl)propan-1-ol (S61). The title compound was obtained as a colorless oil in 82% yield (0.24 g) from **S36** (0.30 g, 1.3 mmol, 1.0 equiv) by following the procedure described for the synthesis of **S17**. The crude **S61** was used directly in the next step without further purification.

3-(1-Benzyl-4,6-dimethyl-1*H*-benzo[*d*]imidazol-2-yl)propyl (1*R*,4a*S*,10a*R*)-7-isopropyl-1,4a-dimethyl-1,2,3,4,4a,9,10,10a-octahydrophenanthrene-1-carboxylate (S62). To a solution of dehydroabietic acid (0.60 g, 2.0 mmol, 3.0 equiv) in toluene (10 mL) was added $SOCl_2$ (0.43 mL, 6.0 mmol, 9 equiv) and DMF (two drops). The reaction mixture was heated to 100 °C for 2 h and cooled to RT. The solvent and excess $SOCl_2$ were removed under reduced pressure. The residue was dissolved in toluene (10 mL). **S61** (0.20 g, 0.68 mmol, 1.0 equiv) and Et_3N (0.28 mL, 2.0 mmol, 1.0 equiv) were added. The reaction mixture was heated to 100 °C for 12 h and cooled to RT. The solvent was removed under reduced pressure. The residue was chromatographed through silica gel eluting with ethyl acetate/hexanes to afford **S62** as a white solid in 41% yield (0.16 g). ^1H NMR (500 MHz, $CDCl_3$) δ 7.25–7.20 (m, 3H), 7.15 (d, *J* = 8.2 Hz, 1H), 7.00 (dd, *J* = 8.1, 2.0 Hz, 1H), 6.95–6.91 (m, 2H), 6.87 (s, 1H), 6.86

(s, 1H), 6.80 (s, 1H), 5.22 (s, 2H), 4.14 (t, *J* = 6.2 Hz, 2H), 2.89–2.79 (m, 5H), 2.62 (s, 3H), 2.38 (s, 3H), 2.29–2.23 (m, 1H), 2.19–2.09 (m, 3H), 1.82–1.74 (m, 1H), 1.73–1.67 (m, 1H), 1.67–1.60 (m, 2H), 1.58–1.53 (m, 1H), 1.39–1.33 (m, 2H), 1.23 (s, 3H), 1.22 (s, 3H), 1.21 (s, 3H), 1.19 (s, 3H); ^{13}C NMR (151 MHz, $CDCl_3$) δ 178.5, 153.0, 147.0, 145.9, 140.1, 136.3, 135.5, 134.7, 132.3, 129.1, 128.8, 127.9, 127.0, 126.0, 124.4, 124.3, 124.1, 107.0, 64.1, 47.8, 46.9, 45.0, 38.0, 37.0, 36.7, 33.6, 30.3, 27.3, 25.3, 24.5, 24.1, 21.9, 18.7, 16.8, 16.6; IR (neat, cm^{-1}): 2925, 1722, 1603, 1454, 1385, 1245, 1173, 1124; ESI HRMS *m/z* $(M+H)^+$ calcd 577.3789, obsd 577.3809.

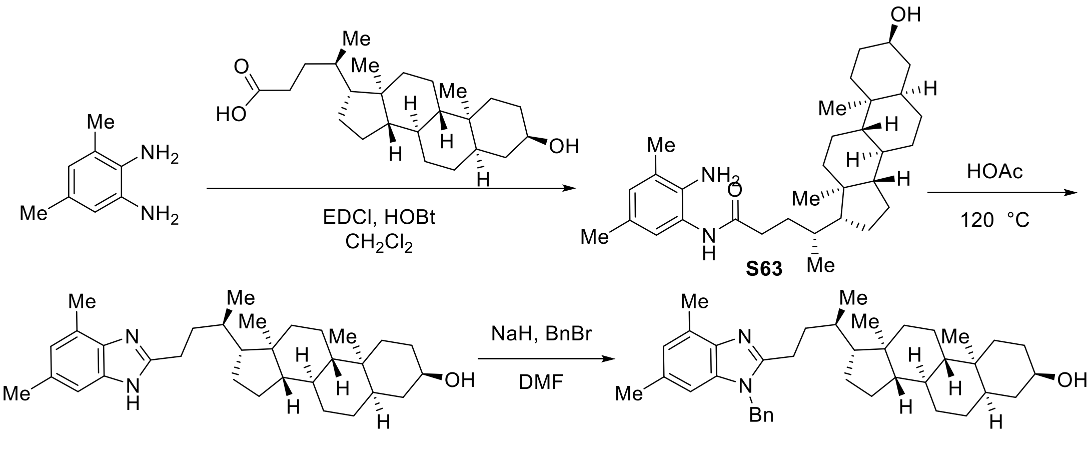

S63 **S64** **S65**

(3*R*,5*R*,8*R*,9*S*,10*S*,13*R*,14*S*,17*R*)-17-((*R*)-4-(1-Benzyl-4,6-dimethyl-1*H*-benzo[*d*]imidazol-2-yl)butan-2-yl)-10,13-dimethylhexadecahydro-1*H*-cyclopenta[*a*]phenanthren-3-ol (S65). The title compound was obtained as a white solid in 19% yield (0.21 g) for three steps from 3,5-dimethylbenzene-1,2-diamine (0.27 g, 2.0 mol, 1.0 equiv), lithocholic acid (0.75 g, 2.0 mol, 1.0 equiv) and BnBr (0.28 mL, 2.4 mmol, 1.2 equiv) by following the procedure described for the synthesis of **S49**. The structure was confirmed by nuclear Overhauser effect (NOE) experiment. ^{1}H NMR (500 MHz, $CDCl_3$) δ 7.35–7.23 (m, 3H), 7.09–6.99 (m, 2H), 6.87 (s, 1H), 6.82 (s, 1H), 5.28 (s, 2H), 3.68–3.56 (m, 1H), 2.96–2.85 (m, 1H), 2.73–2.66 (m, 1H), 2.64 (s, 3H), 2.38 (s, 3H), 1.94 (d, *J* = 12.2 Hz, 1H), 1.88–1.57 (m, 9H), 1.54–1.47 (m, 2H), 1.47–1.32 (m, 8H), 1.27–1.18 (m, 2H), 1.17–1.05 (m, 4H), 1.02–0.98 (m, 1H), 0.95 (d, *J* = 6.1 Hz, 3H), 0.91 (s, 3H), 0.61 (s, 3H); ^{13}C NMR (126 MHz, $CDCl_3$) δ 154.9, 140.1, 136.5, 135.5, 132.1, 129.1, 128.7, 127.8, 126.2, 124.3, 107.1, 71.9, 56.6, 56.2, 47.0, 42.9, 42.3, 40.6, 40.3, 36.6, 36.2, 36.0, 35.5, 34.8, 34.7, 30.7, 28.3, 27.4, 26.6, 25.1, 24.4, 23.5, 21.9, 21.0, 18.6, 17.0, 12.2; IR (neat, cm^{-1}): 3456, 2922, 1456, 1384, 1261, 1029, 842, 750; ESI HRMS *m/z* $(M+H)^+$ calcd 567.4309, obsd 567.4329.

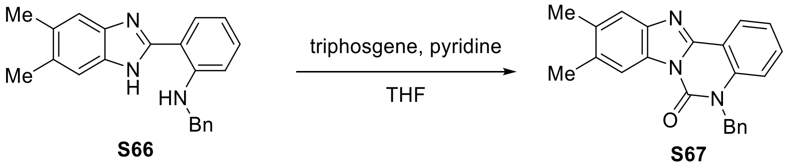

5-Benzyl-9,10-dimethylbenzo[4,5]imidazo[1,2-*c*]quinazolin-6(5*H*)-one (S67). To a solution of triphosgene (0.46 mmol, 1.0 equiv) in anhydrous THF (5 mL) at 0 °C was added dropwise pyridine (2.5 mL). The reaction mixture was stirred for 15 min at this temperature and then treated with **S66**[2] (0.15 g, 0.46 mmol, 1.0 equiv). The resulting reaction mixture was warmed to RT and stirred for 6 h. The reaction was carefully quenched by addition of water (10 mL) and then extracted with Et_2O (3 x 10 mL). The combined organic solution was dried over Na_2SO_4, filtrated and evaporated under reduced pressure. The residue was purified by silica gel column chromatography to afford the product **S67** as a white solid in 54% yield (0.087 g). ^{1}H NMR (500 MHz, $CDCl_3$) δ 8.52 (d, *J* = 7.8 Hz, 1H), 8.27 (s, 1H), 7.66 (s, 1H), 7.47 (t, *J* = 7.8 Hz, 1H), 7.36–7.30 (m, 5H), 7.29–7.24 (m, 2H), 5.56 (s, 2H), 2.45 (s, 3H), 2.44 (s, 3H); ^{13}C NMR (126 MHz, $CDCl_3$) δ 147.7, 146.2, 142.6, 137.3, 135.8, 134.9, 133.8, 132.0, 129.8, 129.2, 127.9, 126.7, 125.7, 123.9, 119.7, 115.7, 115.4, 114.0, 47.2, 20.7 (2s); IR (neat, cm^{-1}): 2916, 1687, 1590, 1480, 1383, 1196, 1076, 733; ESI HRMS *m/z* $(M+H)^+$ calcd 354.1601, obsd 354.1601.

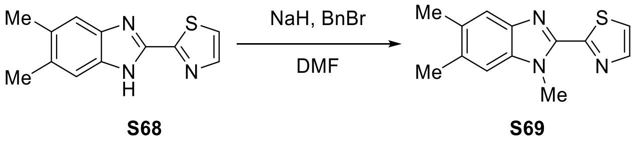

2-(1,5,6-Trimethyl-1*H*-benzo[*d*]imidazol-2-yl)thiazole (S69). The title compound was obtained as a white solid in 72% yield (0.70 g) starting from **S68**[3] (0.92 g, 4.0 mmol, 1.0 equiv) and CH_3I (0.37 mL, 6.0 mmol, 1.5 equiv) by following the General Procedure A (step 2). ^{1}H NMR (500 MHz, $CDCl_3$) δ 7.91 (d, *J* = 3.2 Hz, 1H), 7.56 (s, 1H), 7.41 (d, *J* = 3.3 Hz, 1H), 7.13 (s, 1H), 4.21 (s, 3H), 2.38 (s, 3H), 2.36 (s, 3H); ^{13}C NMR (126 MHz, $CDCl_3$) δ 160.4, 144.8, 143.8, 141.4, 135.7, 133.6, 132.2, 121.0, 120.1, 110.1, 32.1, 20.9, 20.5; IR (neat, cm^{-1}): 3027, 2920, 2854, 1615, 1540, 1450, 1336, 1230, 1101, 827, 731; ESI HRMS *m/z* $(M+Na)^+$ calcd 266.0722, obsd 266.0729.

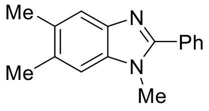

S70

1,5,6-Trimethyl-2-phenyl-1*H*-benzo[*d*]imidazole (S70). The title compound is known in the literature[4].

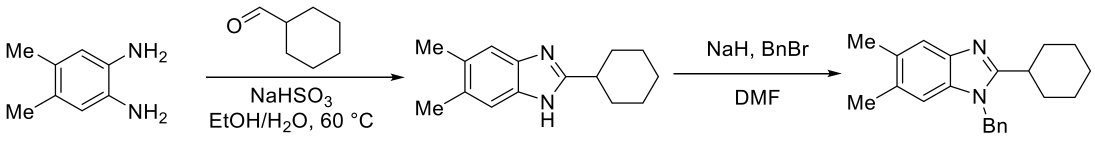

S71 **S72**

1-Benzyl-2-cyclohexyl-5,6-dimethyl-1*H*-benzo[*d*]imidazole (S72). The title compound was obtained as a white solid in 47% yield (0.30 g) for two steps from 4,5-dimethylbenzene-1,2-diamine (0.27 g, 2.0 mmol, 1.0 equiv), cyclohexanecarboxaldehyde (0.29 mL, 2.4 mmol, 1.2 equiv) and BnBr (0.28 mL, 2.4 mmol, 1.2 equiv) by following the General Procedure A. ^{1}H NMR (400 MHz, $CDCl_3$) δ 7.54 (s, 1H), 7.32–7.24 (m, 3H), 7.05–7.00 (m, 2H), 6.93 (s, 1H), 5.30 (s, 2H), 2.79–2.69 (m, 1H), 2.34 (s, 3H), 2.30 (s, 3H), 1.89–1.78 (m, 6H), 1.74–1.69 (m, 1H), 1.41–1.21 (m, 3H); ^{13}C NMR (101 MHz, $CDCl_3$) δ 158.7, 141.5, 136.8, 133.8, 131.2, 130.7, 129.1, 127.8, 126.2, 119.7, 110.0, 46.7, 36.6, 32.1, 26.5, 25.9, 20.6, 20.4; IR (neat, cm^{-1}): 2927, 2853, 1448, 1355, 1137, 1020, 840, 728; ESI HRMS *m/z* $(M+H)^+$ calcd 319.2169, obsd 319.2176.

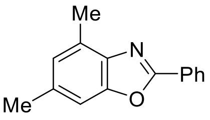

S73

4,6-Dimethyl-2-phenylbenzo[*d*]oxazole (S73). The title compound is known in the literature[5].

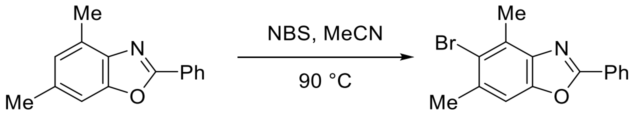

S73 **S74**

5-Bromo-4,6-dimethyl-2-phenylbenzo[*d*]oxazole (S73). A solution of **S73** (0.089 g, 0.40 mmol, 1.0 equiv) and NBS (85.4 mg, 0.48 mmol, 1.2 equiv) in MeCN (8.0 mL) was heated to reflux for 24 h. The reaction mixture was cooled to RT and concentrated under reduced pressure. The residue was chromatographed through silica gel eluting with ethyl acetate/hexanes to give **S74** as a white solid in 96% yield (0.12 g). The structure was confirmed by nuclear Overhauser effect (NOE) experiment. ^{1}H NMR (500 MHz, $CDCl_3$) δ 8.26–8.21 (m, 2H), 7.54–7.49 (m, 3H), 7.33 (s, 1H), 2.73 (s, 3H), 2.55 (s, 3H); ^{13}C NMR (126 MHz, $CDCl_3$) δ 162.7, 149.4, 140.8, 135.1, 131.6, 130.8, 129.0, 127.7, 127.4, 122.9, 109.4, 25.0, 18.1; IR (neat, cm^{-1}): 2919, 1613, 1556, 1446, 1385, 1263, 1165, 1051, 839, 700, 685; ESI HRMS *m/z* $(M+H)^+$ calcd 302.0175, 304.0155, obsd 302.0181, 304.0160.

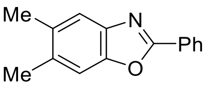

S75

5,6-Dimethyl-2-phenylbenzo[*d*]oxazole (S75). The title compound is known in the literature[6].

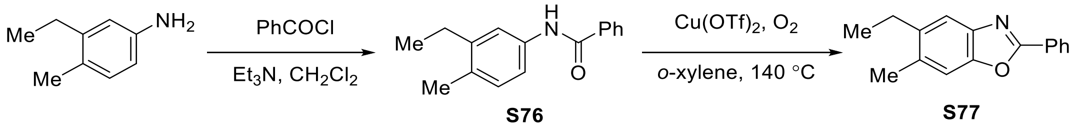

***N*-(3-Ethyl-4-methylphenyl)benzamide (S76)**. To a solution of 3-ethyl-4-methylaniline (0.17 g, 1.2 mmol, 1.0 equiv) in CH_2Cl_2 (10 mL) was added PhCOCl (0.21 g, 1.5 mmol, 1.2 equiv), followed by Et_3N (0.26 mL, 1.9 mmol, 1.5 equiv). The reaction mixture was stirred at RT for 0.5 h, diluted with EtOAc and washed with saturated $NaHCO_3$. The layers were separated and the aqueous layer was extracted twice with EtOAc. The combined organic solution was dried over $MgSO_4$, filtered and concentrated under reduced pressure to afford **S76** as a white solid in 98% (0.29 g), which was used directly in the next step without further purification.

5-Ethyl-6-methyl-2-phenylbenzo[*d*]oxazole (S77). A suspension of **S76** (0.29 g, 1.2 mmol, 1.0 equiv) and $Cu(OTf)_2$ (0.13 g, 0.36 mmol, 0.30 equiv) in *o*-xylene (5 mL) was heated to 140 °C for 24 h under under oxygen atmosphere (balloon). The resulting mixture was then cooled to RT and concentrated under reduced pressure. The residue was chromatographed through silica gel eluting with ethyl acetate/hexanes to afford **S77** as a white solid in 29% yield (0.083 g). ^{1}H NMR (400 MHz, $CDCl_3$) δ 8.26–8.20 (m, 2H), 7.56 (s, 1H), 7.53–7.48 (m, 3H), 7.34 (s, 1H), 2.72 (q, *J* = 7.5 Hz, 2H), 2.42 (s, 3H), 1.29 (t, *J* = 7.5 Hz, 3H); ^{13}C NMR (101 MHz, $CDCl_3$) δ 162.5, 149.4, 140.6, 139.3, 133.8, 131.2, 128.9, 127.6, 127.5, 118.5, 111.3, 26.4, 20.1, 14.7; IR (neat, cm^{-1}): 2921, 2849, 1635, 1553, 1458, 1384, 1270, 1117, 1052, 103, 988, 870, 704; ESI HRMS *m/z* $(M+H)^+$ calcd 238.1226, obsd 238.1228.

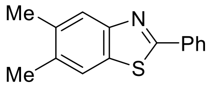

S78

5,6-Dimethyl-2-phenylbenzo[*d*]thiazole (S78). The title compound is known in the literature[5].

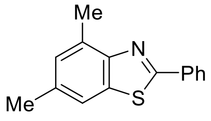

S79

4,6-Dimethyl-2-phenylbenzo[*d*]thiazole (S79). The title compound is known in the literature[7].

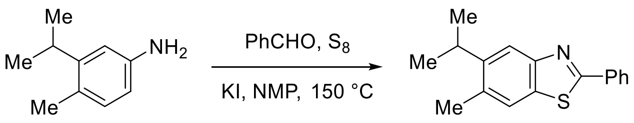

S80

5-Isopropyl-6-methyl-2-phenylbenzo[*d*]thiazole (S80). A 25 mL oven-dried reaction vessel was charged with 3-isopropyl-4-methylaniline (0.40 g, 2.6 mmol, 1.0 equiv), benzaldehyde (0.55 g, 5.2 mmol, 2.0 equiv), S_8 (0.42 g, 13 mmol, 5.0 equiv), KI (0.086 g, 0.50 mmol, 1.0 equiv) and NMP (6.5 mL). The reaction vessel was purged with oxygen and stirred at 150 °C for 24 h. After cooling to RT, the reaction mixture was concentrated under reduced pressure. The residue was purified by silica gel column chromatography to afford the product **S80** as a yellow oil in 35% yield (0.24 g). ^{1}H NMR (500 MHz, $CDCl_3$) δ 8.10–8.07 (m, 2H), 7.99 (s, 1H), 7.65 (s, 1H), 7.50–7.47 (m, 3H), 3.24 (hept, J = 6.9 Hz, 1H), 2.48 (s, 3H), 1.33 (d, J = 6.9 Hz, 6H); ^{13}C NMR (126 MHz, $CDCl_3$) δ 167.2, 153.5, 146.4, 134.0, 133.6, 132.3, 130.8, 129.1, 127.5, 122.3, 119.3, 29.7, 23.7, 20.0; IR (neat, cm^{-1}): 2924, 1481, 1442, 1267, 1074, 1018, 762, 689; ESI HRMS *m/z* $(M+H)^+$ calcd 268.1154, obsd 268.1158.

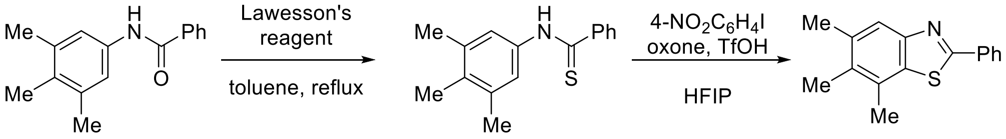

S81 **S82** **S83**

6-(Dimethoxymethyl)-2,5-dimethylbenzo[*d*]thiazole (S82). A mixture of **S81**[8] (2.4 g, 10.0 mmol, 1.0 equiv) and Lawesson's reagent (2.4 g, 6.0 mmol, 0.6 equiv) in toluene (15 mL) was refluxed at 130 °C for 5 h. The solvent was then removed under reduced pressure. The residue was purified by silica gel column chromatography to afford the product **S82** as a pale yellow solid in 85% yield (2.2 g). ^{1}H NMR (500 MHz, $CDCl_3$) δ 8.90 (s, 1H), 7.84 (d, J = 7.5 Hz, 2H), 7.49 (t, J = 7.4 Hz, 1H), 7.42 (t, J = 7.6 Hz, 2H), 7.37 (s, 2H), 2.32 (s, 6H), 2.18 (s, 3H); ^{13}C NMR (126 MHz, $CDCl_3$) δ 198.4, 143.4, 137.5, 136.2, 134.5, 131.3, 128.8, 126.8, 123.1, 20.9, 15.4; IR (neat, cm^{-1}): 3199, 2918, 1593, 1519, 1447, 1355, 1228, 1077, 724, 693; ESI HRMS *m/z* $(M+Na)^+$ calcd 278.0974, obsd 278.0971.

5,6,7-Trimethyl-2-phenylbenzo[*d*]thiazole (S83). Oxone (0.61 g, 7.5 mmol, 1.5 equiv) was added to a stirred solution of **S82** (1.3 g, 5.0 mmol, 1.0 equiv), 4-nitroiodobenzene (0.25 g, 1.0 mmol, 0.2 equiv) and triflic acid (1.3 mL, 15 mmol, 3.0 equiv) in HFIP (20 mL) at RT. The resulting reaction mixture was stirred at RT for 12 h. Saturated $Na_2S_2O_3$ (5 mL) and saturated $NaHCO_3$ (5 mL) was then added to quenched the reaction. The resulting mixture was extracted with ethyl acetate (3 × 15 mL) and washed with brine. The combined organic solution was dried over Na_2SO_4, filtered and evaporated under reduced pressure. The residue was purified by silica gel column chromatography to afford **S83** as a white solid in 82% yield

(1.0 g). ^{1}H NMR (500 MHz, $CDCl_3$) δ 8.12–8.06 (m, 2H), 7.75 (s, 1H), 7.52–7.42 (m, 3H), 2.52 (s, 3H), 2.43 (s, 3H), 2.29 (s, 3H); ^{13}C NMR (126 MHz, $CDCl_3$) δ 166.6, 151.8, 135.9, 134.4, 134.2, 132.3, 130.6, 129.0, 129.0, 127.5, 121.6, 21.4, 20.2, 15.7; IR (neat, cm^{-1}): 2919, 1597, 1478, 1443, 1299, 1084, 761, 687; ESI HRMS *m/z* $(M+Na)^+$ calcd 276.0817, obsd 276.0810.

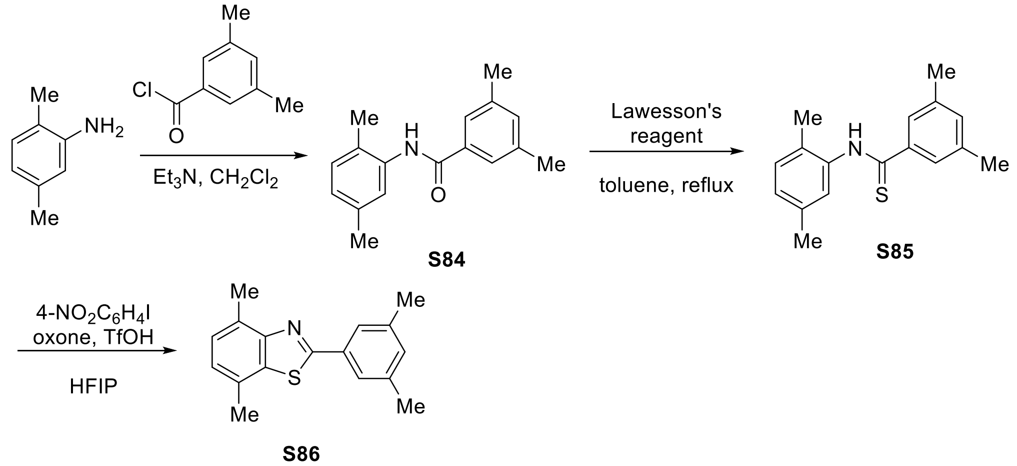

***N*-(2,5-Dimethylphenyl)-3,5-dimethylbenzamide (S84)**. To a solution of 2,5-dimethylaniline (2.5 mL, 20 mmol, 1.0 equiv) and Et_3N (4.2 mL, 30 mmol, 1.5 equiv) in CH_2Cl_2 (80 mL) was added dropwise 3,5-dimethylbenzoyl chloride (3.5 mL, 24 mmol, 1.2 equiv) under argon at 0 °C. The resulting reaction mixture was stirred at RT for 3 h and then concentrated under reduced pressure. The residue was diluted with ethyl acetate and filtered. The filtrate was washed with brine and then dried over $MgSO_4$. The solvent was removed under reduced pressure to afford crude **S84**, which was used in the next step without further purification.

***N*-(2,5-Dimethylphenyl)-3,5-dimethylbenzothioamide (S85)**. A mixture of **S84** (2.5 g, 10.0 mmol, 1.0 equiv) and Lawesson's reagent (2.4 g, 6.0 mmol, 0.6 equiv) in toluene (15 mL) was refluxed at 130 °C for 5 h. The solvent was then removed under reduced pressure. The residue was purified by silica gel column chromatography to afford the product **S85** as a white solid in 90% yield (2.4 g). ^{1}H NMR (850 MHz, $CDCl_3$) δ 8.79 (s, 1H), 7.51 (s, 2H), 7.31 (s, 1H), 7.20 (d, J = 7.8 Hz, 1H), 7.16 (s, 1H), 7.10 (d, J = 7.8 Hz, 1H), 2.39 (s, 6H), 2.36 (s, 3H), 2.28 (s, 3H); ^{13}C NMR (214 MHz, $CDCl_3$) δ 200.1, 142.4, 138.5, 137.5, 136.7, 133.1, 131.3, 130.9, 129.1, 127.2, 124.7, 21.4, 21.1, 17.7; IR (neat, cm^{-1}): 3211, 2920, 1676, 1593, 1498, 1293, 1028, 829, 760, 701; ESI HRMS *m/z* $(M+Na)^+$ calcd 292.1130, obsd 292.1128.

***N*-(2,3-Dimethylphenyl)-3,5-dimethylbenzamide (S86)**. Oxone (0.61 g, 7.5 mmol, 1.5 equiv) was added to a stirred solution of **S85** (1.3 g, 5.0 mmol, 1.0 equiv), 4-nitroiodobenzene (0.25 g, 1.0 mmol, 0.2 equiv) and triflic acid (1.3 mL, 15 mmol, 3.0 equiv) in HFIP (20 mL) at RT.

The resulting reaction mixture was stirred at RT for 12 h. Saturated $Na_2S_2O_3$ (5 mL) and saturated $NaHCO_3$ (5 mL) were then added to quenched the reaction. The resulting mixture was extracted with ethyl acetate (3 × 15 mL) and washed with brine. The combined organic solution was dried over Na_2SO_4, filtered and evaporated under reduced pressure. The residue was purified by silica gel column chromatography to afford **S86** as a white solid in 71% yield (0.95 g). ^{1}H NMR (600 MHz, $CDCl_3$) δ 7.77 (s, 2H), 7.21 (d, *J* = 7.3 Hz, 1H), 7.12 (s, 1H), 7.08 (d, *J* = 7.3 Hz, 1H), 2.80 (s, 3H), 2.56 (s, 3H), 2.43 (s, 6H); ^{13}C NMR (151 MHz, $CDCl_3$) δ 166.9, 153.3, 138.7, 135.5, 134.1, 132.6, 130.6, 128.9, 127.0, 125.4, 125.3, 21.5, 21.4, 18.3; IR (neat, cm^{-1}): 2919, 1434, 1312, 1132, 1041, 848, 792, 686; ESI HRMS *m/z* $(M+Na)^+$ calcd 290.0974, obsd 290.0974.

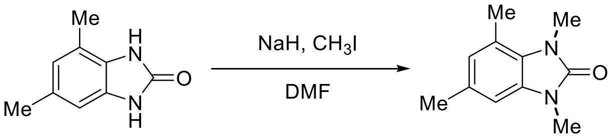

S87

1,3,4,6-Tetramethyl-1,3-dihydro-2*H*-benzo[*d*]imidazol-2-one (S87). The title compound was obtained as a white solid in 64% yield (0.24 g) from 4,6-dimethyl-1,3-dihydro-2*H*-benzo[*d*]imidazol-2-one[9] (0.41 g, 2.5 mmol, 1.0 equiv), NaH (60% dispersion in mineral oil, 0.25 g, 6.3 mmol, 2.5 equiv) and CH_3I (0.39 mL, 6.3 mmol, 2.5 equiv) by following the General Procedure A (step 2). ^{1}H NMR (500 MHz, $CDCl_3$) δ 6.63 (s, 1H), 6.61 (s, 1H), 3.62 (s, 3H), 3.35 (s, 3H), 2.55 (s, 3H), 2.35 (s, 3H); ^{13}C NMR (101 MHz, $CDCl_3$) δ 155.1, 130.8, 130.5, 125.9, 125.1, 119.1, 106.1, 29.9, 27.2, 21.3, 18.1; IR (neat, cm^{-1}): 2934, 1705, 1625, 1459, 1378, 1262, 1098, 1004, 883, 738; ESI HRMS *m/z* $(M+Na)^+$ calcd 213.0998, obsd 213.1004.

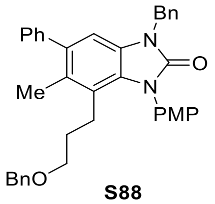

S88

1-Benzyl-4-(3-(benzyloxy)propyl)-3-(4-methoxyphenyl)-5-methyl-6-phenyl-1,3-dihydro-2*H*-benzo[*d*]imidazol-2-one (S88). The title compound is known in the literature[10].

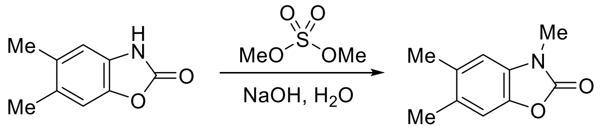

S89 **S90**

3,5,6-Trimethylbenzo[*d*]oxazol-2(3*H*)-one (S90). To a solution of **S89**[11] (0.65 g, 4.0 mmol, 1.0 equiv) and sodium hydroxide (0.18 g, 4.4 mmol, 1.1 equiv) in 10 mL water was added dimethyl sulfate (0.45 mL, 4.8 mmol, 1.2 equiv). The reaction mixture was heated until complete consumption of **S89** (monitored by TLC). The reaction mixture was then cooled to RT and extracted with ethyl acetate (3 x 10 mL). The combined organic solution was washed with brine, dried over anhydrous Na_2SO_4, filtered and concentrated under reduced pressure. The residue was purified by silica gel column chromatography to afford the product **S90** as a yellow solid in 69% yield (0.46 g). ^{1}H NMR (400 MHz, $CDCl_3$) δ 6.87 (s, 1H), 6.67 (s, 1H), 3.29 (s, 3H), 2.24 (s, 3H), 2.21 (s, 3H); ^{13}C NMR (101 MHz, $CDCl_3$) δ 155.0, 140.8, 132.0, 130.6, 129.5, 110.7, 109.1, 28.0, 19.9, 19.8; IR (neat, cm^{-1}): 3442, 1635, 1054, 1033, 1014; ESI HRMS *m/z* (M+Na)$^+$ calcd 200.0682, obsd 200.0678.

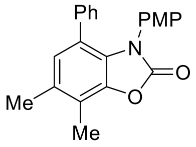

S91

3-(4-Methoxyphenyl)-6,7-dimethyl-4-phenylbenzo[*d*]oxazol-2(3*H*)-one (S91). The title compound is known in the literature[10].

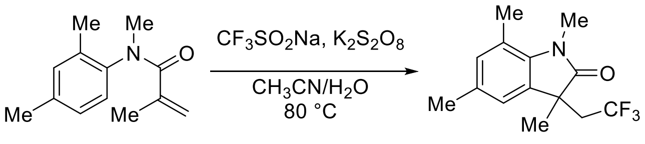

S92

1,3,5,7-Tetramethyl-3-(2,2,2-trifluoroethyl)indolin-2-one (S92). A solution of $K_2S_2O_8$ (0.54 g, 2.0 mmol, 2.0 equiv), *N*-(3,5-dimethylphenyl)-*N*-methylmethacrylamide[12] (0.20 g, 1.0 mmol, 1.0 equiv) and CF_3SO_2Na (0.46 g, 3.0 mmol, 3.0 equiv) in CH_3CN (8 mL) and H_2O (2 mL) was heated to 80 °C for 12 h. The resulting mixture was cooled to RT and concentrated under reduced pressure. The residue was chromatographed through silica gel eluting with ethyl acetate/hexanes to afford **S92** as a white solid in 55% yield (0.15 g). ^{1}H NMR (500 MHz, $CDCl_3$) δ 6.89 (s, 1H), 6.84 (s, 1H), 3.48 (s, 3H), 2.87–2.75 (m, 1H), 2.65–2.55 (m, 1H), 2.54 (s, 3H), 2.29 (s, 3H), 1.36 (s, 3H); ^{13}C NMR (101 MHz, $CDCl_3$) δ 179.3, 138.3, 132.8, 132.1, 131.9, 125.4 (q, $J_{C\text{-}F}$ = 278.2 Hz), 122.2, 119.8, 43.9, 41.0 (q, $J_{C\text{-}F}$ = 27.9 Hz), 29.9, 25.7, 20.9, 19.0; ^{19}F NMR (471 MHz, $CDCl_3$) δ −61.9 (t, J = 10.6 Hz); IR (neat, cm^{-1}): 2923, 2853, 1710, 1457, 1362, 1262, 1184, 1149, 1071; ESI HRMS *m/z* (M+H)$^+$ calcd 272.1257, obsd 272.1263.

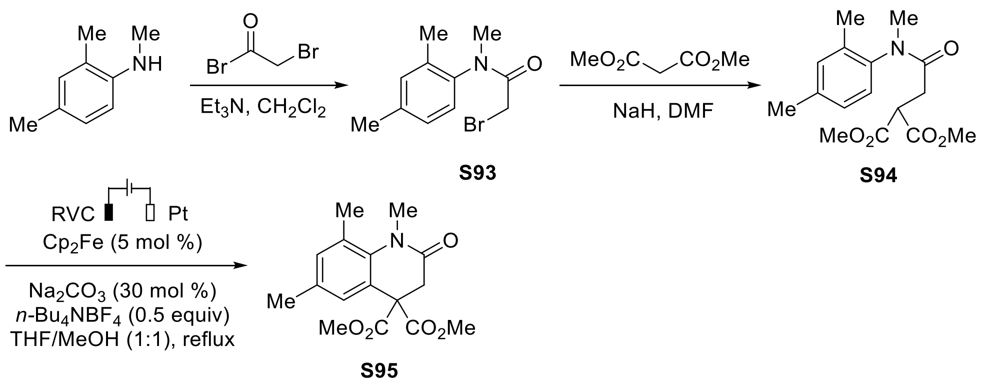

2-Bromo-*N*-(2,4-dimethylphenyl)-N-methylacetamide (S93). To a solution of *N*,2,4-trimethylbenzenamine (1.1 g, 8.0 mmol, 1.0 equiv) in CH_2Cl_2 (30 mL) was added bromoacetyl bromide (0.84 mL, 9.6 mmol, 1.2 equiv), followed by Et_3N (1.7 mL, 12 mmol, 1.5 equiv). The reaction mixture was stirred at RT for 0.5 h, diluted with EtOAc and washed with saturated $NaHCO_3$. The layers were separated and the aqueous layer was extracted twice with EtOAc. The combined organic solution was dried over $MgSO_4$ and concentrated under reduced pressure to afford **S93** as a white solid, which was used directly in the next step without further purification.

Dimethyl 2-(2-((2,4-dimethylphenyl)(methyl)amino)-2-oxoethyl)malonate (S94). To a solution of dimethyl malonate (1.8 mL, 16 mmol, 2.0 equiv) in DMF (30 mL) was added NaH (60% dispersion in mineral oil, 0.64 g, 16 mmol, 2.0 equiv). After being stirred at RT for 0.5 h, the crude **S93** (8.0 mmol, 1.0 equiv) was added and stirred at RT for 0.5 h. H_2O (100 mL) was added to quench the reaction. The resulting mixture was extracted with ethyl acetate (100 mL). The organic layer was washed with H_2O (100 mL), dried over $MgSO_4$, filtered and concentrated under reduced pressure. The residue was chromatographed through silica gel eluting with ethyl acetate/hexanes to afford **S94** as a light yellow oil in 77% yield (1.9 g). ^1H NMR (400 MHz, $CDCl_3$) δ 7.09 (s, 1H), 7.04–6.98 (m, 2H), 3.95 (dd, J = 8.0, 6.7 Hz, 1H), 3.68 (s, 3H), 3.67 (s, 3H), 3.12 (s, 3H), 2.59 (dd, J = 16.9, 6.7 Hz, 1H), 2.40 (dd, J = 16.9, 8.0 Hz, 1H), 2.31 (s, 3H), 2.20 (s, 3H); ^{13}C NMR (101 MHz, $CDCl_3$) δ 169.9, 169.7, 169.6, 139.2, 138.6, 135.3, 132.4, 128.4, 127.8, 52.7, 52.7, 47.8, 36.1, 33.6, 21.1, 17.2; IR (neat, cm^{-1}): 3002, 2954, 1792, 1652, 1505, 1435, 1348, 1113, 1037, 970, 827, 582; ESI HRMS *m/z* $(M+Na)^+$ calcd 330.1312, obsd 330.1318.

Dimethyl 1,6,8-trimethyl-2-oxo-2,3-dihydroquinoline-4,4(1*H*)-dicarboxylate (S95). The title compound was obtained by following the literature[13]: A 50 mL three–necked round bottom flask was charged with Cp_2Fe (0.019 g, 0.10 mmol, 0.05 equiv), **S94** (0.61 g, 2.0 mmol, 1.0 equiv), *n*-Bu_4NBF_4 (0.33 g, 1.0 mmol, 0.50 equiv) and Na_2CO_3 (0.064 g, 0.60 mmol, 0.3 equiv). The flask was equipped with a reticulated vitreous carbon (100 PPI) anode

(1 cm x 1 cm x 1 cm) and a platinum plate (1 cm x 1 cm) cathode. The flask was flushed with argon. THF (10 mL) and MeOH (10 mL) were added. The constant current (20 mA) electrolysis was carried out at reflux until complete consumption of **S94** (monitored by TLC or ^{1}H NMR). The solvent was removed under reduced pressure. The residue was chromatographed through silica gel eluting with AcOEt/hexane to **S95** as a white solid in 61% yield (0.38 g). ^{1}H NMR (400 MHz, $CDCl_3$) δ 6.99–6.96 (m, 1H), 6.80–6.76 (m, 1H), 3.79 (s, 6H), 3.23 (s, 3H), 3.12 (s, 2H), 2.30 (s, 3H), 2.27 (s, 3H); ^{13}C NMR (101 MHz, $CDCl_3$) δ 169.7, 169.6, 138.3, 134.3, 133.6, 128.4, 127.6, 124.8, 58.0, 53.5, 39.7, 36.1, 20.9, 20.7; IR (neat, cm^{-1}): 2954, 1738, 1683, 1481, 1435, 1359, 1265, 1251, 1167, 1143, 1056; ESI HRMS *m/z* $(M+Na)^+$ calcd 328.1155, obsd 328.1164.

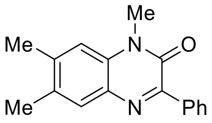

S96

1,6,7-Trimethyl-3-phenylquinoxalin-2(1*H*)-one (S96). The title compound is known in the literature[14].

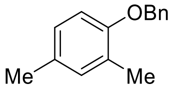

S97

1-(Benzyloxy)-2,4-dimethylbenzene (S97). The title compound is known in the literature[15].

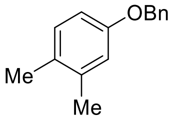

S98

4-(Benzyloxy)-1,2-dimethylbenzene (S98). The title compound is known in the literature[16].

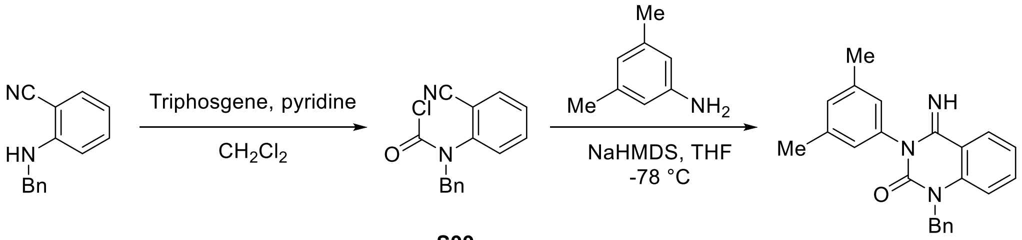

S99 **S100**

Benzyl(2-cyanophenyl)carbamic chloride (S99). To a solution of triphosgene (7.4 g, 25 mmol, 0.5 equiv) in CH_2Cl_2 (200 mL) was added dropwise pyridine (15 mL, 187 mmol, 3.75 equiv) at 0 °C. After being stirred for 15 min at 0 °C, 2-(benzylamino)benzonitrile[17] (6.0 g, 50 mmol, 1.0 equiv) was added. The reaction mixture was warmed to RT, stirred for 6 h and then

carefully quenched by addition of water (100 mL). The resulting mixture was extracted with Et_2O (3 x 200 mL). The combined organic solution was washed with brine, dried over Na_2SO_4, filtered and concentrated to give **S99**. The crude carbamic chloride was used in the next step without purification.

1-Benzyl-3,4-dihydro-4-imino-3-(3,5-dimethylphenyl)quinazolin-2(1*H*)-one (S100). NaHMDS (2.0 M in THF solution, 30 mL, 60 mmol, 1.2 equiv) was added dropwise to the solution of 3,5-dimethylaniline (6.0 g, 50 mmol, 1.0 equiv) in dry THF (200 mL) at –78 °C. After being stirred for 30 min at this temperature, the crude **S99** obtained above (50 mmol, 1.0 equiv) was added. The reaction mixture was stirred at –78 °C for 1 h and then warmed to RT for 1 h. Water (100 mL) was added to quench the reaction. The mixture was extracted with ethyl acetate (3 x 100 mL). The combined organic solution was dried over Na_2SO_4, filtrated and evaporated under reduced pressure. The residue was purified by silica gel column chromatography to afford **S100** as a white solid in 73% yield (13 g). ^{1}H NMR (500 MHz, $CDCl_3$) δ 8.34 (s, 1H), 7.41 (ddd, *J* = 8.7, 7.3, 1.7 Hz, 1H), 7.34–7.28 (m, 4H), 7.26–7.21 (m, 1H), 7.15–7.11 (m, 1H), 7.10 (s, 1H), 7.05 (d, *J* = 8.4 Hz, 1H), 6.93 (s, 2H), 6.72 (br, 1H), 5.30 (s, 2H), 2.36 (s, 6H); ^{13}C NMR (126 MHz, $CDCl_3$) δ 154.3, 150.8, 140.3, 138.6, 136.4, 135.1, 133.3, 131.2, 128.9, 127.7, 127.6, 126.7 (2s), 123.0, 117.1, 114.5, 47.2, 21.4; IR (neat, cm^{-1}): 3302, 2918, 1684, 1478, 1305, 1165, 1028, 830, 753; ESI HRMS *m/z* $(M+H)^+$ calcd 356.1757, obsd 356.1751.

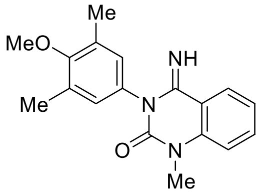

S101

3,4-Dihydro-4-imino-3-(4-methoxy-3,5-dimethylphenyl)-1-methylquinazolin-2(1*H*)-one (S101). The title compound was prepared as a white solid in 86% yield (1.1 g) from 2-(methylamino)benzonitrile[18] (0.53 g, 4.0 mmol, 1.0 equiv) and 4-methoxy-3,5-dimethylaniline (0.61 g, 4.0 mmol, 1.0 equiv) by following the procedure described for the synthesis of **S100**. ^{1}H NMR (500 MHz, $CDCl_3$) δ 8.36 (s, 1H), 7.58 (t, *J* = 7.7 Hz, 1H), 7.21 (t, *J* = 7.7 Hz, 1H), 7.14 (d, *J* = 8.4 Hz, 1H), 6.92 (s, 2H), 6.65 (br, 1H), 3.78 (s, 3H), 3.54 (s, 3H), 2.32 (s, 6H); ^{13}C NMR (126 MHz, $CDCl_3$) δ 157.6, 154.5, 150.6, 139.2, 133.4, 133.3, 130.5, 129.3, 127.7, 123.0, 116.9, 113.6, 59.8, 30.6, 16.3; IR (neat, cm^{-1}): 3302, 2920, 1687, 1622, 1482, 1302, 1225, 1126, 1009, 752; ESI HRMS *m/z* $(M+H)^+$ calcd 310.1550, obsd 310.1543.

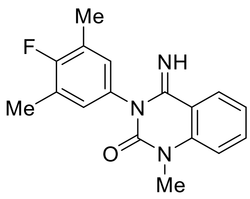

S102

3-(4-Fluoro-3,5-dimethylphenyl)-3,4-dihydro-4-imino-1-methylquinazolin-2(1*H*)-one (S102). The title compound was prepared as a white solid in 67% yield (1.0 g) from 2-(methylamino)benzonitrile (1.3 g, 10 mmol, 2.0 equiv) and 4-fluoro-3,5-dimethylaniline (0.70 g, 5.0 mmol, 1.0 equiv) by following the procedure described for the synthesis of **S100**. ^{1}H NMR (500 MHz, $CDCl_3$) δ 8.32 (s, 1H), 7.59 (ddd, *J* = 8.6, 7.3, 1.6 Hz, 1H), 7.25–7.18 (m, 1H), 7.15 (d, *J* = 8.3 Hz, 1H), 6.93 (d, *J* = 6.2 Hz, 2H), 6.65 (br, 1H), 3.54 (s, 3H), 2.30 (2s, 6H); ^{13}C NMR (126 MHz, $CDCl_3$) δ 160.0 (d, $J_{C\text{-}F}$ = 246.2 Hz), 154.5, 150.5 , 139.2, 133.5, 130.3, 129.5 (d, $J_{C\text{-}F}$ = 5.6 Hz), 127.6, 126.9 (d, $J_{C\text{-}F}$ = 19.6 Hz), 123.1, 116.8, 113.7, 30.7, 14.9 (d, $J_{C\text{-}F}$ = 4.1 Hz); ^{19}F NMR (471 MHz, $CDCl_3$) δ −119.8–−119.9 (m, 1F); IR (neat, cm^{-1}): 3300, 2925, 1686, 1621, 1481, 1302, 1125, 1041, 751; ESI HRMS *m/z* (M+H)$^{+}$ calcd 298.1350, obsd 298.1344.

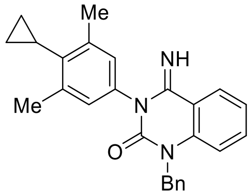

S103

1-Benzyl-3-(4-cyclopropyl-3,5-dimethylphenyl)-3,4-dihydro-4-iminoquinazolin-2(1*H*)-one (S103). The title compound was prepared as a white solid in 40% yield (1.3 g) starting from 2-(benzylamino)benzonitrile (1.7 g, 8.0 mmol, 1.0 equiv) and 4-cyclopropyl-3,5-dimethylaniline (1.3 g, 8.0 mmol, 1.0 equiv) by following the procedure described for the synthesis of **S100**. ^{1}H NMR (500 MHz, $CDCl_3$) δ 8.39 (s, 1H), 7.44 (ddd, *J* = 8.7, 7.3, 1.6 Hz, 1H), 7.36–7.31 (m, 4H), 7.29–7.23 (m, 1H), 7.16 (t, *J* = 7.8 Hz, 1H), 7.08 (d, *J* = 8.4 Hz, 1H), 6.94 (s, 2H), 6.74 (s, 1H), 5.32 (s, 2H), 2.47 (s, 6H), 1.73 (ddd, *J* = 14.5, 8.4, 6.0 Hz, 1H), 1.09–1.02 (m, 2H), 0.59 (td, *J* = 6.0, 4.3 Hz, 2H); ^{13}C NMR (126 MHz, $CDCl_3$) δ 154.4, 150.9, 141.5, 138.7, 136.5, 133.4, 132.7, 129.0, 128.1, 127.8, 127.6, 126.9, 123.1, 117.2, 114.6, 47.4, 20.9, 12.2, 8.2; IR (neat, cm^{-1}): 3302, 2922, 1685, 1495, 1304, 1183, 1026, 873, 765; ESI HRMS *m/z* (M+H)$^{+}$ calcd 396.2070, obsd 396.2065.

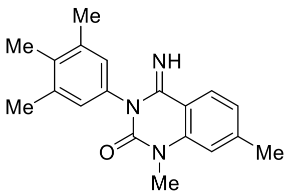

S104

3,4-Dihydro-4-imino-1,7-dimethyl-3-(3,4,5-trimethylphenyl)quinazolin-2(1*H*)-one (S104). The title compound was prepared as a white solid in 44% yield (0.81 g) starting from 4-methyl-2-(methylamino)benzonitrile[19] (0.88 g, 6.0 mmol, 1.0 equiv) and 3,4,5-trimethylaniline (1.0 mL, 7.0 mmol, 1.2 equiv) by following the procedure described for the synthesis of **S100**. ^{1}H NMR (500 MHz, $CDCl_3$) δ 8.19 (d, *J* = 8.0 Hz, 1H), 7.02 (dd, *J* = 8.0, 1.4 Hz, 1H), 6.94 (s, 1H), 6.90 (s, 2H), 3.53 (s, 3H), 2.46 (s, 3H), 2.31 (s, 6H), 2.22 (s, 3H); ^{13}C NMR (126 MHz, $CDCl_3$) δ 154.9, 150.8, 144.2, 139.3, 138.7, 136.6, 132.3, 127.8, 127.5, 124.1, 114.4, 114.0, 30.6, 22.2, 20.8, 15.4; IR (neat, cm^{-1}): 3303, 2920, 1687, 1474, 1386, 1299, 1180, 1077, 826, 746; ESI HRMS *m/z* $(M+H)^+$ calcd 308.1757, obsd 308.1756.

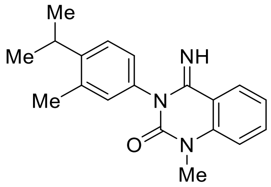

S105

3,4-Dihydro-4-imino-3-(4-isopropyl-3-methylphenyl)-1-methylquinazolin-2(1*H*)-one (S105). The title compound was prepared as a pale yellow oil in 70% yield (1.1 g) from 2-(methylamino)benzonitrile (0.66 g, 5.0 mmol, 1.0 equiv) and 4-isopropyl-3-methylaniline (0.68 g, 5.0 mmol, 1.0 equiv) by following the procedure described for the synthesis of **S100**. ^{1}H NMR (850 MHz, $CDCl_3$) δ 8.35 (s, 1H), 7.58 (ddd, *J* = 8.7, 7.3, 1.6 Hz, 1H), 7.41 (d, *J* = 8.1 Hz, 1H), 7.23–7.19 (m, 1H), 7.15 (d, *J* = 8.4 Hz, 1H), 7.07 (dd, *J* = 8.1, 2.4 Hz, 1H), 7.02 (d, *J* = 2.4 Hz, 1H), 6.65 (br, 1H), 3.54 (s, 3H), 3.18 (hept, *J* = 6.9 Hz, 1H), 2.37 (s, 3H), 1.27 (d, *J* = 7.5 Hz, 6H); ^{13}C NMR (214 MHz, $CDCl_3$) δ 154.5, 150.5, 148.2, 139.3, 137.6, 133.4, 132.4, 130.5, 127.6, 126.9, 126.7, 123.0, 116.9, 113.6, 30.6, 29.4, 23.2, 19.5; IR (neat, cm^{-1}): 3304, 2927, 1686, 1620, 1482, 1394, 1309, 1167, 1088, 752; ESI HRMS *m/z* $(M+H)^+$ calcd 308.1757, obsd 308.1758.

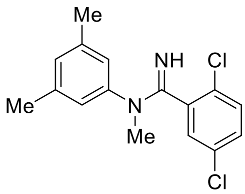

S106

2,5-Dichloro-*N*-(3,5-dimethylphenyl)-*N*-methylbenzimidamide (S106). The title

compound was prepared as a brown solid in 70% yield (1.1 g) from *N*,3,5-trimethylaniline (0.68 g, 5.0 mmol, 1.0 equiv) and 2,5-dichlorobenzonitrile (1.0 g, 6.0 mmol, 1.2 equiv) by following the procedure described for the synthesis of **S23**. ^{1}H NMR (500 MHz, $CDCl_3$) δ 7.22 (d, *J* = 2.5 Hz, 1H), 7.15 (d, *J* = 8.6 Hz, 1H), 7.08 (dd, *J* = 8.6, 2.5 Hz, 1H), 6.73 (s, 2H), 6.71 (s, 1H), 3.34 (s, 3H), 2.18 (s, 6H); ^{13}C NMR (126 MHz, $CDCl_3$) δ 163.6, 144.7, 139.2, 138.7, 132.5, 130.8, 129.6, 129.5, 129.4, 128.0, 124.4, 39.0, 21.2; IR (neat, cm^{-1}): 3318, 2919, 1581, 1434, 1367, 1113, 1034, 814, 704; ESI HRMS *m/z* $(M+Na)^+$ calcd 329.0588, obsd 329.0585.

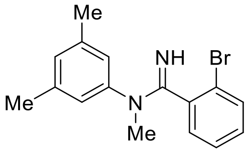

S107

2-Bromo-*N*-(3,5-dimethylphenyl)-*N*-methylbenzimidamide (S107). The title compound was prepared as a white solid in 80% yield (1.3 g) from *N*,3,5-trimethylaniline (0.68 g, 5.0 mmol, 1.0 equiv) and 2-bromobenzonitrile (1.1 g, 6.0 mmol, 1.2 equiv) by following the procedure described for the synthesis of **S23**. ^{1}H NMR (500 MHz, $CDCl_3$) δ 7.43 (d, *J* = 8.0 Hz, 1H), 7.24 (d, *J* = 7.4 Hz, 1H), 7.18 (t, *J* = 7.5 Hz, 1H), 7.07 (t, *J* = 7.7 Hz, 1H), 6.77 (s, 2H), 6.71 (s, 1H), 6.35 (brs, 1H), 3.41 (s, 3H), 2.18 (s, 6H); ^{13}C NMR (126 MHz, $CDCl_3$) δ 165.8, 144.6, 138.7, 132.9, 130.1, 129.8, 128.1, 127.2, 124.4, 120.7, 39.7, 21.2; IR (neat, cm^{-1}): 3316, 2918, 1580, 1477, 1384, 1180, 1053, 848, 761; ESI HRMS *m/z* $(M+H)^+$ calcd 317.0653, 319.0627, obsd 317.0648, 319.0628.

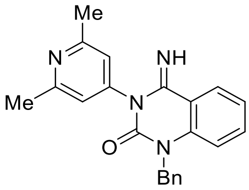

S108

1-Benzyl-3-(2,6-dimethylpyridin-4-yl)-4-imino-3,4-dihydroquinazolin-2(1H)-one (S108). The title compound is known in the literature[20].

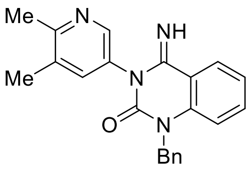

S109

1-Benzyl-3,4-dihydro-4-imino-3-(5,6-dimethylpyridin-3-yl)quinazolin-2(1*H*)-one (S109). The title compound was prepared as a white solid in 42% yield (0.76 g) from

2-(benzylamino)benzonitrile (0.79 g, 6.0 mmol, 1.2 equiv) and 5,6-dimethylpyridin-3-amine (0.61 g, 5.0 mmol, 1.0 equiv) by following the procedure described for the synthesis of **S100**. ^{1}H NMR (500 MHz, $CDCl_3$) δ 8.37 (brs, 1H), 8.32 (d, *J* = 2.4 Hz, 1H), 7.46–7.40 (m, 2H), 7.34–7.28 (m, 4H), 7.27–7.22 (m, 1H), 7.18–7.13 (m, 1H), 7.08 (d, *J* = 8.4 Hz, 1H), 6.69 (s, 1H), 5.30 (s, 2H), 2.57 (s, 3H), 2.34 (s, 3H); ^{13}C NMR (126 MHz, $CDCl_3$) δ 158.4, 155.0, 150.7, 146.7, 138.4, 137.9, 136.0, 133.6, 133.2, 130.3, 129.0, 127.6, 127.5, 126.7, 123.3, 116.4, 114.8, 47.4, 22.5, 19.3; IR (neat, cm^{-1}): 3308, 2924, 1686, 1622, 1478, 1305, 1162, 1024, 752, 729; ESI HRMS *m/z* $(M+H)^+$ calcd 357.1710, obsd 357.1705.

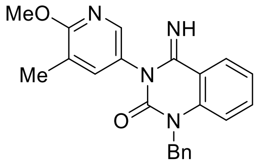

S110

1-Benzyl-3,4-dihydro-4-imino-3-(6-methoxy-5-methylpyridin-3-yl)quinazolin-2(1*H*)-one (S110). The title compound was prepared as a white solid in 45% yield (0.84 g) from 2-(benzylamino)benzonitrile (0.79 g, 6.0 mmol, 1.2 equiv) and 6-methoxy-5-methylpyridin-3-amine (0.69 g, 5.0 mmol, 1.0 equiv) by following the procedure described for the synthesis of **S100**. ^{1}H NMR (500 MHz, $CDCl_3$) δ 8.32 (brs, 1H), 8.00 (dd, *J* = 2.6, 0.9 Hz, 1H), 7.46 (ddd, *J* = 8.7, 7.3, 1.6 Hz, 1H), 7.37 (dd, *J* = 2.6, 0.9 Hz, 1H), 7.36–7.34 (m, 1H), 7.33–7.30 (m, 3H), 7.29–7.24 (m, 1H), 7.21–7.14 (m, 1H), 7.09 (d, *J* = 8.4 Hz, 1H), 5.32 (s, 2H), 4.03 (s, 3H), 2.25 (s, 3H); ^{13}C NMR (126 MHz, $CDCl_3$) δ 162.7, 154.8, 151.0, 144.5, 139.1, 138.5, 136.2, 133.6, 129.0, 127.7, 126.8, 125.5, 123.3, 122.9, 116.7, 114.8, 54.1, 47.5, 16.2; IR (neat, cm^{-1}): 3302, 2922, 1693, 1556, 1494, 1180, 1077, 857, 751; ESI HRMS *m/z* $(M+H)^+$ calcd 373.1659, obsd 373.1653.

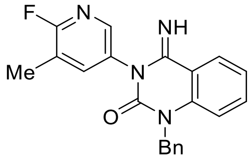

S111

1-Benzyl-3-(6-fluoro-5-methylpyridin-3-yl)-4-imino-3,4-dihydroquinazolin-2(1*H*)-one (S111). The title compound is known in the literature[20].

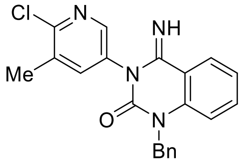

S112

1-Benzyl-3-(6-chloro-5-methylpyridin-3-yl)-3,4-dihydro-4-iminoquinazolin-2(1*H*)-one (S112). The title compound was prepared as a white solid in 54% yield (1.0 g) from

2-(benzylamino)benzonitrile (0.79 g, 6.0 mmol, 1.2 equiv) and 6-chloro-5-methylpyridin-3-amine (0.71 g, 5.0 mmol, 1.0 equiv) by following the procedure described for the synthesis of **S100**. ^{1}H NMR (500 MHz, $CDCl_3$) δ 8.24 (d, *J* = 2.6 Hz, 1H), 8.16 (brs, 1H), 7.59 (d, *J* = 2.6 Hz, 1H), 7.46 (ddd, *J* = 8.7, 7.3, 1.6 Hz, 1H), 7.35–7.28 (m, 4H), 7.29–7.24 (m, 1H), 7.20–7.16 (m, 1H), 7.11 (d, *J* = 8.4 Hz, 1H), 5.31 (s, 2H), 2.42 (s, 3H); ^{13}C NMR (126 MHz, $CDCl_3$) δ 151.7, 150.5, 147.4, 140.3, 138.1, 135.8, 134.1, 133.8, 131.9, 129.0, 127.7, 127.1, 126.6, 123.4, 116.0, 115.0, 47.3, 19.8; IR (neat, cm^{-1}): 3312, 2918, 1681, 1621, 1478, 1404, 1306, 1195, 1132, 1077, 734; ESI HRMS *m/z* $(M+H)^+$ calcd 377.1164, obsd 377.1151.

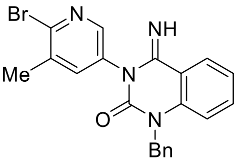

S113

1-Benzyl-3-(6-bromo-5-methylpyridin-3-yl)-3,4-dihydro-4-iminoquinazolin-2(1*H*)-one (S113). The title compound was prepared as a white solid in 76% yield (1.6 g) from 2-(benzylamino)benzonitrile (0.79 g, 6.0 mmol, 1.2 equiv) and 6-bromo-5-methylpyridin-3-amine (0.93 g, 5.0 mmol, 1.0 equiv) by following the procedure described for the synthesis of **S100**. ^{1}H NMR (500 MHz, $CDCl_3$) δ 8.23 (d, *J* = 2.6 Hz, 1H), 8.15 (s, 1H), 7.55 (d, *J* = 2.6 Hz, 1H), 7.48 (ddd, *J* = 8.6, 7.3, 1.6 Hz, 1H), 7.37–7.32 (m, 2H), 7.32–7.26 (m, 3H), 7.20 (t, *J* = 7.4 Hz, 1H), 7.11 (d, *J* = 8.5 Hz, 1H), 5.32 (s, 2H), 2.46 (s, 3H); ^{13}C NMR (126 MHz, $CDCl_3$) δ 155.3, 150.6, 147.8, 144.9, 139.8, 138.3, 136.9, 135.9, 133.9, 132.5, 129.1, 127.8, 127.2, 126.7, 123.5, 116.1, 115.1, 47.5, 22.3; IR (neat, cm^{-1}): 3312, 2921, 1685, 1621, 1478, 1400, 1305, 1161, 1055, 721; ESI HRMS *m/z* $(M+Na)^+$ calcd 443.0478, 445.0457, obsd 443.0480, 445.0459.

Procedures for the Electrochemical Oxidation

Milligram scale reaction: A 10 mL three-necked round-bottomed flask was charged with the substrate (0.20 mmol, 1.0 equiv) and Et_4NPF_6 (0.10 mmol, 0.5 equiv). The flask was then equipped with a condenser, a reticulated vitreous carbon (100 PPI, ~65 cm^2 cm^{-3}, 1.2 cm x 1.0 cm x 0.8 cm) anode and a platinum plate (1.0 cm x 1.0 cm) cathode, and flushed with argon (Supplementary Figure 1a). MeOH (9.0 mL) was added. The electrolysis was carried out at 80 °C (oil bath temperature) using a constant current of 10 mA until complete consumption of the substrate (monitored by TLC or ^{1}H NMR). The reaction mixture was cooled to RT and concentrated under reduced pressure. The residue was chromatographed through silica gel eluting with ethyl acetate/hexanes with 1% triethylamine to give the desired product.

The gram scale synthesis of **56** was conducted in a 1000-mL beaker-type cell using two reticulated vitreous carbon (100 PPI, 5.0 cm x 5.0 cm x 1.2 cm) anodes, a platinum plate (5.0 cm x 5.0 cm x 0.1 mm) cathode and a constant current of 500 mA. The three electrodes were placed in parallel with the Pt plate cathode sandwiched between two RVC anodes (Supplementary Figure 1b). 10 g of **S100** (28.2 mmol) and 900 mL of MeOH were employed. The electrolysis was carried out at 80 °C (oil bath temperature). The reaction mixture was refluxed for 12.5 h (electricity = 8.3 F mol^{-1}) and isolated 8.3 g of **56** (71% yield).

The 60 mmol scale reaction of **75** was conducted in a 2.0-L beaker-type cell with two pieces of RVC (5.0 cm x 9.0 cm x 1.2 cm) as the anode, a Pt plate cathode (5 cm x 5 cm x 0.1 mm) and a constant current of 1,000 mA. The three electrodes were placed in parallel with the Pt plate cathode sandwiched between two RVC anodes. The electrolytic cell was linked with a 2.0-L glass bottle as a reservoir for reaction mixture. The continuous circulation of solution between the cell and the reservoir was achieved by a peristaltic pump through Teflon catheter with a flow rate 22 mL min^{-1} (Supplementary Figure 1c). The reaction mixture consisted **75** (27.0 g, 60.0 mmol), Et_4NPF_6 (6.5 g, 30 mmol) and MeOH (2.7 L). Each glass bottle contained 1.37 L MeOH before electrolysis. The reaction time was 12.5 h (electricity = 4.1 F mol^{-1}) and isolated 14.2 g of **76** (46% yield). The structure was confirmed by nuclear Overhauser effect (NOE) experiment.

a

b

c

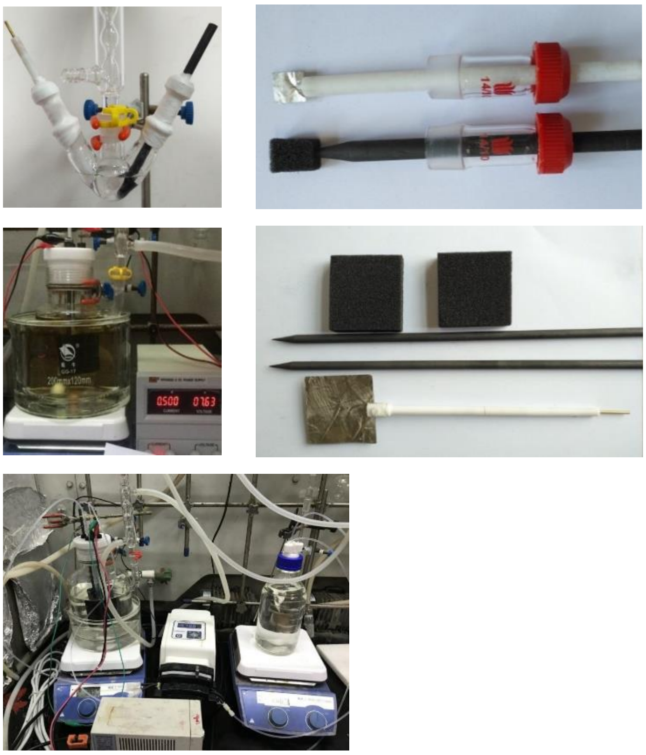

Supplementary Figure 1. Electrolysis setup. **a**, electrolysis setup for milligram scale reactions. **b**, electrolysis setup for gram scale reactions. **c**, Electrolysis setup for decagram reaction of **75**.

Characterization Data for the Electrolysis Products

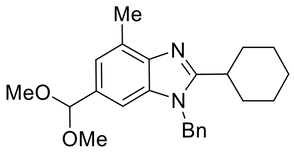

1-Benzyl-2-cyclohexyl-6-(dimethoxymethyl)-4-methyl-1*H*-benzo[*d*]imidazole (2). The structure was confirmed by nuclear Overhauser effect (NOE) experiment. Yield = 72%, electricity = 4.3 F mol^{-1}; White solid; ^{1}H NMR (500 MHz, $CDCl_3$) δ 7.30–7.24 (m, 3H), 7.15 (s, 1H), 7.12 (s, 1H), 7.03–6.99 (m, 2H), 5.41 (s, 1H), 5.35 (s, 2H), 3.29 (s, 6H), 2.81–2.73 (m, 1H), 2.69 (s, 3H), 1.87–1.81 (m, 6H), 1.73–1.68 (m, 1H), 1.39–1.26 (m, 3H); ^{13}C NMR (126 MHz, $CDCl_3$) δ 159.4, 142.5, 136.7, 134.9, 132.2, 129.2, 129.0, 127.8, 126.2, 121.3, 105.5, 103.9, 52.9, 46.9, 36.9, 31.9, 26.5, 25.7, 17.2; IR (neat, cm^{-1}): 2937, 1686, 1608, 1497, 1140, 843, 737; ESI HRMS *m/z* $(M+H)^+$ calcd 379.2380, obsd 379.2386.

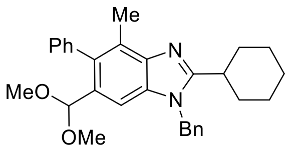

1-Benzyl-2-cyclohexyl-6-(dimethoxymethyl)-4-methyl-5-phenyl-1*H*-benzo[*d*]imidazole (3). The structure was confirmed by nuclear Overhauser effect (NOE) experiment. Yield = 67%, electricity = 4.1 F mol^{-1}; Colorless oil; ^{1}H NMR (850 MHz, $CDCl_3$) δ 7.45–7.38 (m, 3H), 7.38–7.34 (m, 1H), 7.32–7.25 (m, 3H), 7.24–7.20 (m, 2H), 7.11–7.06 (m, 2H), 5.39 (s, 2H), 4.90 (s, 1H), 3.12 (s, 6H), 2.84–2.76 (m, 1H), 2.38 (s, 3H), 1.88–1.81 (m, 6H), 1.74–1.68 (m, 1H), 1.39–1.28 (m, 3H); ^{13}C NMR (101 MHz, $CDCl_3$) δ 159.9, 142.5, 140.0, 136.8, 135.1, 134.1, 130.9, 130.4, 129.0, 128.0, 127.8, 127.5, 126.9, 126.4, 104.4, 103.3, 54.1, 46.9, 37.0, 31.9, 26.5, 25.8, 15.0; IR (neat, cm^{-1}): 2927, 2853, 1507, 1451, 1396, 1072, 704; ESI HRMS *m/z* $(M+H)^+$ calcd 455.2693, obsd 455.2699.

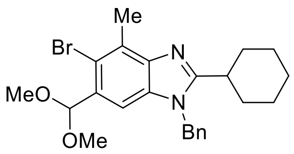

1-Benzyl-5-bromo-2-cyclohexyl-6-(dimethoxymethyl)-4-methyl-1*H*-benzo[*d*]imidazole (4). The structure was confirmed by nuclear Overhauser effect (NOE) experiment. Yield = 55%, electricity = 4.4 F mol^{-1}; Colorless oil; ^{1}H NMR (600 MHz, $CDCl_3$) δ 7.33 (s, 1H), 7.29–7.24 (m, 3H), 7.01–6.98 (m, 2H), 5.66 (s, 1H), 5.34 (s, 2H), 3.33 (s, 6H), 2.80–2.73 (m, 1H), 2.76 (s, 3H), 1.90–1.77 (m, 7H), 1.37–1.26 (m, 3H); ^{13}C NMR (151 MHz, $CDCl_3$) δ 160.3, 143.5, 136.3, 133.7, 130.5, 130.1, 129.1, 128.0, 126.2, 118.3, 106.7, 104.4, 54.4, 47.0,

36.9, 31.8, 26.4, 25.8, 17.8; IR (neat, cm^{-1}): 2930, 2853, 1507, 1450, 1075, 996, 733; ESI HRMS *m/z* $(M+H)^+$ calcd 457.1485, obsd 457.1501.

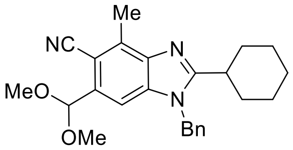

1-Benzyl-2-cyclohexyl-6-(dimethoxymethyl)-4-methyl-1*H*-benzo[*d*]imidazole-5-carbonitrile (5). The structure was confirmed by nuclear Overhauser effect (NOE) experiment. Yield = 70%, electricity = 4.6 F mol^{-1}; White solid; ^1H NMR (600 MHz, $CDCl_3$) δ 7.37 (s, 1H), 7.33–7.27 (m, 3H), 7.01–6.97 (m, 2H), 5.66 (s, 1H), 5.38 (s, 2H), 3.37 (s, 6H), 2.89 (s, 3H), 2.78 (tt, *J* = 9.6, 4.7 Hz, 1H), 1.87–1.79 (m, 6H), 1.75–1.70 (m, 1H), 1.37–1.28 (m, 3H); ^{13}C NMR (101 MHz, $CDCl_3$) δ 161.6, 142.1, 137.1, 135.8, 135.6, 129.2, 128.2, 126.2, 117.6, 106.3, 104.7, 102.6, 54.5, 47.1, 36.9, 31.8, 26.3, 25.7, 15.6; IR (neat, cm^{-1}): 2932, 2856, 2218, 1611, 1448, 1053, 858, 727; ESI HRMS *m/z* $(M+Na)^+$ calcd 426.2152, obsd 426.2156.

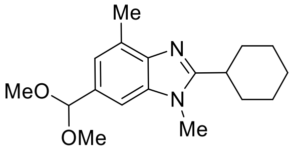

2-Cyclohexyl-6-(dimethoxymethyl)-1,4-dimethyl-1*H*-benzo[*d*]imidazole (6). The structure was confirmed by nuclear Overhauser effect (NOE) experiment. Yield = 63%, electricity = 4.4 F mol^{-1}; Colorless oil; ^1H NMR (500 MHz, $CDCl_3$) δ 7.25 (s, 1H), 7.09 (s, 1H), 5.48 (s, 1H), 3.73 (s, 3H), 3.35 (s, 6H), 2.86 (tt, *J* = 11.8, 3.5 Hz, 1H), 2.66 (s, 3H), 1.99 (dd, *J* = 13.0, 3.5 Hz, 2H), 1.95–1.89 (m, 2H), 1.85 (td, *J* = 12.3, 3.2 Hz, 2H), 1.80–1.75 (m, 1H), 1.49–1.37 (m, 3H); ^{13}C NMR (151 MHz, $CDCl_3$) δ 159.0, 142.3, 135.5, 132.0, 129.0, 121.1, 104.8, 104.1, 53.0, 36.8, 31.4, 29.9, 26.5, 25.9, 17.1; IR (neat, cm^{-1}): 2929, 1688, 1510, 1450, 1343, 1194, 1134, 1053, 867, 842; ESI HRMS *m/z* $(M+Na)^+$ calcd 325.1886, obsd 325.1883.

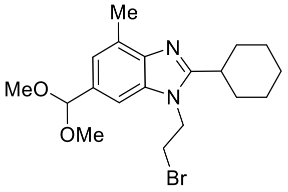

1-(2-Bromoethyl)-2-cyclohexyl-6-(dimethoxymethyl)-4-methyl-1*H*-benzo[*d*]imidazole (7). The structure was confirmed by nuclear Overhauser effect (NOE) experiment. Yield = 78%, electricity = 5.4 F mol^{-1}; Colorless oil; ^1H NMR (500 MHz, $CDCl_3$) δ 7.24 (s, 1H), 7.11 (s, 1H), 5.47 (s, 1H), 4.51 (t, *J* = 7.3 Hz, 2H), 3.61 (t, *J* = 7.3 Hz, 2H), 3.35 (s, 6H), 2.90–2.82 (m, 1H), 2.66 (s, 3H), 1.98–1.85 (m, 6H), 1.81–1.74 (m, 1H), 1.47–1.38 (m, 3H); ^{13}C NMR (126 MHz, $CDCl_3$) δ 158.9, 142.5, 134.0, 132.6, 129.5, 121.7, 104.6, 103.9, 53.0, 44.9, 36.7, 32.1,

28.7, 26.5, 25.8, 17.1; IR (neat, cm^{-1}): 2929, 1687, 1449, 1424, 1139, 1134, 1052; ESI HRMS *m/z* $(M+H)^+$ calcd 395.1329, obsd 395.1347.

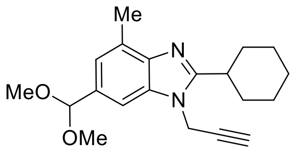

2-Cyclohexyl-6-(dimethoxymethyl)-4-methyl-1-(prop-2-yn-1-yl)-1*H*-benzo[*d*]imidazole (8). The structure was confirmed by nuclear Overhauser effect (NOE) experiment. Yield = 63%, electricity = 4.7 F mol^{-1}; White solid; ^1H NMR (500 MHz, $CDCl_3$) δ 7.35 (s, 1H), 7.12 (s, 1H), 5.49 (s, 1H), 4.86 (d, *J* = 2.6 Hz, 2H), 3.35 (s, 6H), 2.89 (tt, *J* = 11.7, 3.5 Hz, 1H), 2.66 (s, 3H), 2.34 (t, *J* = 2.5 Hz, 1H), 2.06–2.00 (m, 2H), 1.94–1.89 (m, 2H), 1.89–1.80 (m, 2H), 1.80–1.74 (m, 1H), 1.49–1.36 (m, 3H); ^{13}C NMR (126 MHz, $CDCl_3$) δ 158.4, 142.3, 134.2, 132.5, 129.3, 121.6, 105.2, 103.9, 77.4, 73.4, 52.9, 36.8, 32.9, 31.7, 26.5, 25.8, 17.1; IR (neat, cm^{-1}): 2932, 1688, 1510, 1447, 1346, 1141, 978, 843; ESI HRMS *m/z* $(M+H)^+$ calcd 327.2067, obsd 327.2079.

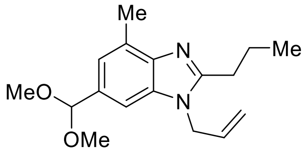

1-Allyl-6-(dimethoxymethyl)-4-methyl-2-propyl-1*H*-benzo[*d*]imidazole (9). The structure was confirmed by nuclear Overhauser effect (NOE) experiment. Yield = 60%, electricity = 5.0 F mol^{-1}; White solid; ^1H NMR (500 MHz, $CDCl_3$) δ 7.21 (s, 1H), 7.11 (s, 1H), 5.93 (ddt, *J* = 17.3, 9.9, 4.8 Hz, 1H), 5.46 (s, 1H), 5.19 (d, *J* = 10.4 Hz, 1H), 4.95 (d, *J* = 17.1 Hz, 1H), 4.73 (dt, *J* = 3.8, 1.8 Hz, 2H), 3.34 (s, 6H), 2.87–2.81 (m, 2H), 2.66 (s, 3H), 1.87–1.82 (m, 2H), 1.04 (t, *J* = 7.3 Hz, 3H); ^{13}C NMR (151 MHz, $CDCl_3$) δ 155.3, 142.3, 134.8, 132.2, 128.9, 121.3, 117.3, 105.4, 104.0, 53.0, 45.9, 29.8, 22.1, 17.0, 14.2; IR (neat, cm^{-1}): 2931, 1687, 1514, 1452, 1359, 1192, 1075, 988, 848; ESI HRMS *m/z* $(M+Na)^+$ calcd 311.1730, obsd 311.1727.

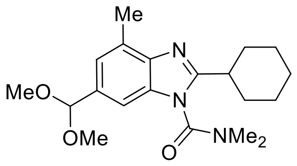

2-Cyclohexyl-6-(dimethoxymethyl)-*N*,*N*,4-trimethyl-1*H*-benzo[*d*]imidazole-1-carboxamide (10). The structure was confirmed by nuclear Overhauser effect (NOE) experiment of the regioselective isomers. Yield = 72%, electricity = 4.7 F mol^{-1}; Colorless oil; Isolated as a 10:1 mixture of regioselective isomers and only the major isomer was shown: ^1H NMR (500 MHz, $CDCl_3$) δ 7.13 (s, 2H), 5.43 (s, 1H), 3.31 (s, 6H), 3.23 (brs, 3H), 3.00 (tt, *J* = 11.7, 3.6 Hz,

1H), 2.83 (brs, 3H), 2.64 (s, 3H), 2.22–2.08 (m, 1H), 1.91–1.68 (m, 6H), 1.44–1.30 (m, 3H); ^{13}C NMR (126 MHz, $CDCl_3$) δ 158.7, 152.8, 142.0, 133.5, 132.5, 129.7, 122.3, 106.2, 103.7, 53.0, 38.7, 37.4, 37.0, 32.2, 31.0, 26.3, 25.9, 17.1; IR (neat, cm^{-1}): 2931, 1698, 1519, 1451, 1385, 1275, 1057; ESI HRMS *m/z* $(M+H)^+$ calcd 360.2282, obsd 360.2283.

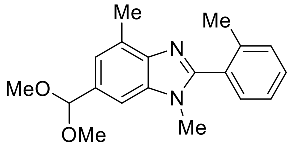

6-(Dimethoxymethyl)-1,4-dimethyl-2-(*o*-tolyl)-1*H*-benzo[*d*]imidazole (11). The structure was confirmed by nuclear Overhauser effect (NOE) experiment. Yield = 54%, electricity = 5.0 F mol^{-1}; White solid; ^{1}H NMR (850 MHz, $CDCl_3$) δ 7.42–7.36 (m, 3H), 7.33 (d, *J* = 7.6 Hz, 1H), 7.30 (t, *J* = 7.4 Hz, 1H), 7.20 (s, 1H), 5.52 (s, 1H), 3.60 (s, 3H), 3.40 (s, 6H), 2.71 (s, 3H), 2.24 (s, 3H); ^{13}C NMR (214 MHz, $CDCl_3$) δ 153.8, 142.7, 138.3, 135.3, 132.9, 130.5 (2s), 130.3, 130.0, 129.8, 125.9, 121.6, 105.5, 104.1, 53.1, 30.9, 19.9, 17.0; IR (neat, cm^{-1}): 2920, 1670, 1473, 1353, 1262, 1120, 1083, 790; ESI HRMS *m/z* $(M+H)^+$ calcd 311.1760, obsd 311.1762.

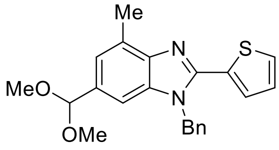

1-Benzyl-6-(dimethoxymethyl)-4-methyl-2-(thiophen-2-yl)-1*H*-benzo[*d*]imidazole (**12**). The structure was confirmed by nuclear Overhauser effect (NOE) experiment. Yield = 50%, electricity = 8.3 F mol^{-1}; Colorless oil; ^{1}H NMR (500 MHz, $CDCl_3$) δ 7.46 (dd, *J* = 5.1, 1.2 Hz, 1H), 7.35–7.29 (m, 3H), 7.28–7.26 (m, 1H), 7.22–7.19 (m, 2H), 7.10–7.05 (m, 3H), 5.57 (s, 2H), 5.43 (s, 1H), 3.31 (s, 6H), 2.74 (s, 3H); ^{13}C NMR (151 MHz, $CDCl_3$) δ 148.1, 142.8, 136.5, 136.0, 133.5, 132.2, 130.0, 129.2, 128.7, 128.2, 127.9 (2s), 126.0, 122.2, 106.0, 103.8, 53.0, 48.3, 17.0; IR (neat, cm^{-1}): 2925, 1688, 1607, 1454, 1359, 1277, 1125, 854, 734; ESI HRMS *m/z* $(M+H)^+$ calcd 379.1475, obsd 379.1478.

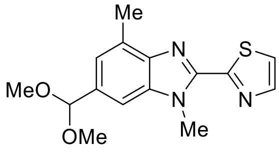

2-(6-(Dimethoxymethyl)-1,4-dimethyl-1*H*-benzo[*d*]imidazol-2-yl)thiazole (**13**). The structure was confirmed by nuclear Overhauser effect (NOE) experiment. Yield = 74%, electricity = 5.2 F mol^{-1}; Colorless oil; ^{1}H NMR (600 MHz, $CDCl_3$) δ 7.96 (d, *J* = 3.2 Hz, 1H), 7.47 (d, *J* = 3.2 Hz, 1H), 7.40 (s, 1H), 7.19 (s, 1H), 5.51 (s, 1H), 4.30 (s, 3H), 3.38 (s, 6H), 2.71 (s, 3H); ^{13}C NMR (151 MHz, $CDCl_3$) δ 160.3, 145.5, 143.9, 142.6, 136.8, 134.3, 130.3,

122.2, 121.4, 105.9, 103.8, 53.1, 32.3, 16.9; IR (neat, cm^{-1}): 2922, 1688, 1609, 1541, 1451, 1354; ESI HRMS *m/z* $(M+H)^+$ calcd 304.1114, obsd 304.1124.

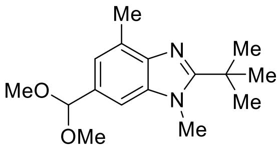

2-(*tert*-Butyl)-6-(dimethoxymethyl)-1,4-dimethyl-1*H*-benzo[*d*]imidazole (14). The structure was confirmed by nuclear Overhauser effect (NOE) experiment. Yield = 56%, electricity = 5.3 F mol^{-1}; Colorless oil; ^1H NMR (600 MHz, $CDCl_3$) δ 7.24 (s, 1H), 7.10 (s, 1H), 5.49 (s, 1H), 3.89 (s, 3H), 3.36 (s, 6H), 2.66 (s, 3H), 1.56 (s, 9H); ^{13}C NMR (151 MHz, $CDCl_3$) δ 161.0, 141.3, 137.1, 132.2, 129.3, 121.1, 104.6, 104.1, 53.0, 34.1, 32.6, 29.5, 16.8; IR (neat, cm^{-1}): 2930, 1693, 1504, 1464, 1337, 1270, 1138, 1053, 852, 752; ESI HRMS *m/z* $(M+Na)^+$ calcd 299.1730, obsd 299.1730.

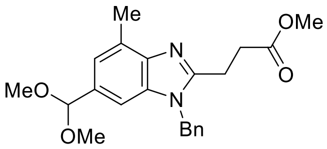

Methyl 3-(1-benzyl-6-(dimethoxymethyl)-4-methyl-1*H*-benzo[*d*]imidazol-2-yl)propanoate (15). The structure was confirmed by nuclear Overhauser effect (NOE) experiment. Yield = 57%, electricity = 4.7 F mol^{-1}; White solid; ^1H NMR (500 MHz, $CDCl_3$) δ 7.30–7.25 (m, 3H), 7.22 (s, 1H), 7.13 (s, 1H), 7.06–7.01 (m, 2H), 5.43 (s, 1H), 5.39 (s, 2H), 3.66 (s, 3H), 3.30 (s, 6H), 3.10 (t, J = 7.4 Hz, 2H), 2.93 (t, J = 7.4 Hz, 2H), 2.66 (s, 3H); ^{13}C NMR (101 MHz, $CDCl_3$) δ 173.2, 153.7, 142.3, 136.3, 135.3, 132.7, 129.2, 129.1, 128.0, 126.3, 121.5, 105.5, 103.9, 53.0, 51.9, 47.0, 31.8, 22.8, 16.9; IR (neat, cm^{-1}): 2932, 2857, 1737, 1687, 1508, 1436, 1363, 1120, 737; ESI HRMS *m/z* $(M+Na)^+$ calcd 405.1785, obsd 405.1805.

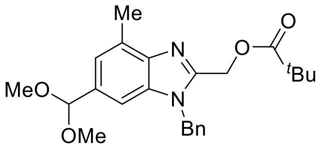

(1-Benzyl-6-(dimethoxymethyl)-4-methyl-1*H*-benzo[*d*]imidazol-2-yl)methyl pivalate (**16**). The structure was confirmed by nuclear Overhauser effect (NOE) experiment. Yield = 60%, electricity = 5.2 F mol^{-1}; Colorless oil; ^1H NMR (500 MHz, $CDCl_3$) δ 7.30–7.25 (m, 3H), 7.23 (s, 1H), 7.20 (s, 1H), 7.04–7.00 (m, 2H), 5.44 (s, 2H), 5.43 (s, 1H), 5.31 (s, 2H), 3.30 (s, 6H), 2.71 (s, 3H), 1.09 (s, 9H); ^{13}C NMR (101 MHz, $CDCl_3$) δ 177.9, 148.9, 142.1, 136.0, 135.5, 134.0, 130.3, 129.1, 128.0, 126.2, 122.0, 106.2, 103.7, 59.0, 53.0, 47.4, 39.0, 27.2, 16.9; IR (neat, cm^{-1}): 2930, 1733, 1691, 1480, 1453, 1276, 1139, 1054; ESI HRMS *m/z* $(M+Na)^+$ calcd 433.2098, obsd 433.2100.

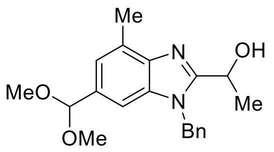

1-(1-Benzyl-6-(dimethoxymethyl)-4-methyl-1*H*-benzo[*d*]imidazol-2-yl)ethan-1-ol (**17**). The structure was confirmed by nuclear Overhauser effect (NOE) experiment. Yield = 53%, electricity = 4.1 F mol^{-1}; White solid; ^{1}H NMR (500 MHz, $CDCl_3$) δ 7.25–7.21 (m, 3H), 7.14 (s, 1H), 7.09 (s, 1H), 6.99–6.95 (m, 2H), 5.47, 5.37 (ABq, J_{AB} = 16.8 Hz, 2H), 5.39 (s, 1H), 5.11 (q, *J* = 6.7 Hz, 1H), 4.50 (brs, 1H), 3.27 (s, 6H), 2.64 (s, 3H), 1.54 (d, *J* = 6.6 Hz, 3H); ^{13}C NMR (101 MHz, $CDCl_3$) δ 157.0, 141.4, 136.3, 135.6, 133.3, 129.7, 129.0, 127.9, 126.2, 121.8, 106.1, 103.8, 64.1, 52.9, 47.3, 22.9, 16.9; IR (neat, cm^{-1}): 3340, 2928, 1689, 1454, 1359, 843, 734; ESI HRMS *m/z* $(M+H)^+$ calcd 341.1860, obsd 341.1872.

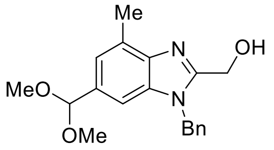

(1-Benzyl-6-(dimethoxymethyl)-4-methyl-1*H*-benzo[*d*]imidazol-2-yl)methanol (**18**). The structure was confirmed by nuclear Overhauser effect (NOE) experiment. Yield = 61%, electricity = 6.2 F mol^{-1}; White solid; ^{1}H NMR (500 MHz, $CDCl_3$) δ 7.32–7.22 (m, 3H), 7.20 (s, 1H), 7.13 (s, 1H), 7.11–7.01 (m, 2H), 5.69 (brs, 1H), 5.48 (s, 2H), 5.40 (s, 1H), 4.86 (s, 2H), 3.29 (s, 6H), 2.61 (s, 3H); ^{13}C NMR (151 MHz, $CDCl_3$) δ 154.1, 141.2, 136.0, 135.2, 133.6, 129.6, 129.1, 128.0, 126.5, 121.9, 106.1, 103.7, 57.2, 53.0, 47.3, 16.9; IR (neat, cm^{-1}): 2917, 1603, 1455, 1365, 1137, 1053, 971, 699; ESI HRMS *m/z* $(M+Na)^+$ calcd 349.1523, obsd 349.1534.

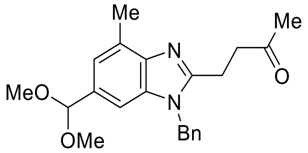

4-(1-Benzyl-6-(dimethoxymethyl)-4-methyl-1*H*-benzo[*d*]imidazol-2-yl)butan-2-one (**19**). The structure was confirmed by nuclear Overhauser effect (NOE) experiment. Yield = 61%, electricity = 8.5 F mol^{-1}; White solid; ^{1}H NMR (500 MHz, $CDCl_3$) δ 7.30–7.24 (m, 3H), 7.22 (s, 1H), 7.13 (s, 1H), 7.05–7.02 (m, 2H), 5.43 (s, 1H), 5.38 (s, 2H), 3.30 (s, 6H), 3.06–3.02 (m, 4H), 2.66 (s, 3H), 2.18 (s, 3H); ^{13}C NMR (101 MHz, $CDCl_3$) δ 207.5, 154.2, 142.2, 136.4, 135.3, 132.6, 129.1, 129.0, 127.9, 126.4, 121.4, 105.5, 103.9, 53.0, 47.0, 40.9, 30.2, 21.5, 16.9; IR (neat, cm^{-1}): 2925, 1716, 1687, 1609, 1455, 1361, 1120, 736; ESI HRMS *m/z* $(M+H)^+$ calcd 367.2016, obsd 367.2023.

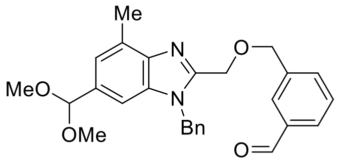

3-(((1-Benzyl-6-(dimethoxymethyl)-4-methyl-1*H*-benzo[*d*]imidazol-2-yl)methoxy)methyl)benzaldehyde (**20**). The structure was confirmed by nuclear Overhauser effect (NOE) experiment. Yield = 50%, electricity = 4.7 F mol^{-1}; Coroless oil; ^{1}H NMR (500 MHz, $CDCl_3$) δ 9.93 (s, 1H), 7.79–7.75 (m, 1H), 7.72 (s, 1H), 7.49–7.42 (m, 2H), 7.26–7.22 (m, 4H), 7.18 (s, 1H), 7.05–7.02 (m, 2H), 5.49 (s, 2H), 5.43 (s, 1H), 4.85 (s, 2H), 4.61 (s, 2H), 3.31 (s, 6H), 2.71 (s, 3H); ^{13}C NMR (151 MHz, $CDCl_3$) δ 192.2, 150.2, 141.9, 138.7, 136.6, 136.3, 135.6, 133.8, 133.7, 130.1, 129.2, 129.1, 129.0, 129.0, 127.9, 126.4, 121.8, 106.1, 103.7, 72.0, 65.7, 53.0, 47.5, 17.0; IR (neat, cm^{-1}): 2922, 1748, 1698, 1622, 1506, 1453, 1361, 1140, 1076; ESI HRMS *m/z* (M+Na)$^+$ calcd 467.1941, obsd 467.1946.

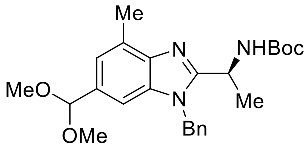

***tert*-Butyl (*S*)-(1-(1-benzyl-6-(dimethoxymethyl)-4-methyl-1*H*-benzo[*d*]imidazol-2-yl)ethyl)carbamate** (**21**). The structure was confirmed by nuclear Overhauser effect (NOE) experiment. Yield = 62%, electricity = 4.7 F mol^{-1}; White solid; ^{1}H NMR (500 MHz, $CDCl_3$) δ 7.30–7.23 (m, 3H), 7.22 (s, 1H), 7.15 (s, 1H), 7.07–7.02 (m, 2H), 5.57 (d, *J* = 8.7 Hz, 1H), 5.53–5.44 (m, 2H), 5.43 (s, 1H), 5.13–5.03 (m, 1H), 3.29 (d, *J* = 2.0 Hz, 6H), 2.67 (s, 3H), 1.47 (d, *J* = 6.8 Hz, 3H), 1.39 (s, 9H); ^{13}C NMR (151 MHz, $CDCl_3$) δ 155.8, 155.2, 142.0, 136.4, 135.0, 133.1, 129.6, 129.0, 127.9, 126.4, 121.7, 106.1, 103.7, 79.8, 52.9, 52.9, 47.0, 43.1, 28.5, 21.2, 17.0; IR (neat, cm^{-1}): 3348, 2929, 1679, 1525, 1453, 1171, 1060; ESI HRMS *m/z* (M+H)$^+$ calcd 440.2544, obsd 440.2561.

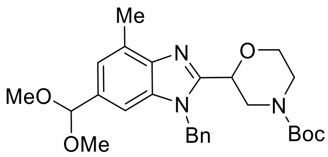

***t*ert-Butyl 2-(1-benzyl-6-(dimethoxymethyl)-4-methyl-1*H*-benzo[*d*]imidazol-2-yl)morpholine-4-carboxylate** (**22**). The structure was confirmed by nuclear Overhauser effect (NOE) experiment. Yield = 63%, electricity = 4.4 F mol^{-1}; Colorless oil; ^{1}H NMR (600 MHz, $CDCl_3$) δ 7.31–7.25 (m, 3H), 7.21 (s, 1H), 7.15 (s, 1H), 7.10–7.06 (m, 2H), 5.55–5.47 (m, 2H), 5.42 (s, 1H), 4.61 (dd, *J* = 10.5, 2.9 Hz, 1H), 4.24 (brs, 1H), 4.02–3.90 (m, 2H), 3.61 (t, *J* = 10.9 Hz, 1H), 3.57–3.49 (m, 1H), 3.28 (2s, 6H), 3.06 (brs, 1H), 2.68 (s, 3H), 1.45 (s, 9H); ^{13}C NMR

(151 MHz, $CDCl_3$) δ 154.7, 150.3, 142.0, 136.5, 135.4, 133.5, 130.3, 129.0, 127.9, 126.6, 121.7, 106.0, 103.7, 80.5, 71.5, 67.0, 52.9, 52.9, 47.6, 46.9, 42.9, 28.5, 16.9; IR (neat, cm^{-1}): 2975, 2928, 1697, 1416, 1365, 1168, 865, 733; ESI HRMS *m/z* $(M+H)^+$ calcd 482.2649, obsd 482.2651.

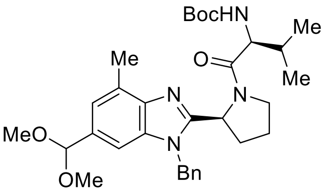

***tert*-Butyl ((*S*)-1-((*S*)-2-(1-benzyl-6-(dimethoxymethyl)-4-methyl-1*H*-benzo[*d*]imidazol-2-yl)pyrrolidin-1-yl)-3-methyl-1-oxobutan-2-yl)carbamate** (**23**). The structure was confirmed by nuclear Overhauser effect (NOE) experiment. Yield = 65%, electricity = 4.7 F mol^{-1}; Colorless oil; ^1H NMR (600 MHz, $CDCl_3$) δ 7.32–7.28 (m, 2H), 7.27–7.25 (m, 1H), 7.24 (s, 1H), 7.11 (s, 1H), 7.10–7.09 (m, 2H), 5.63, 5.49 (ABq, J_{AB} = 17.0 Hz, 2H), 5.42 (s, 1H), 5.17 (d, *J* = 9.3 Hz, 1H), 5.13 (dd, *J* = 7.9, 5.1 Hz, 1H), 4.32 (dd, *J* = 9.4, 6.3 Hz, 1H), 3.98–3.93 (m, 1H), 3.90–3.85 (m, 1H), 3.31 (s, 3H), 3.30 (s, 3H), 2.58 (s, 3H), 2.40–2.32 (m, 1H), 2.06–1.99 (m, 1H), 1.97–1.88 (m, 3H), 1.42 (s, 9H), 0.98 (d, *J* = 6.8 Hz, 3H), 0.81 (d, *J* = 6.7 Hz, 3H); ^{13}C NMR (126 MHz, $CDCl_3$) δ 171.2, 156.0, 155.3, 142.4, 137.1, 135.0, 132.6, 129.5, 129.0, 127.9, 126.5, 121.2, 105.6, 104.0, 79.5, 57.2, 53.4, 53.0, 52.9, 47.6, 47.1, 31.6, 31.4, 28.5, 25.2, 19.4, 17.7, 16.7; IR (neat, cm^{-1}): 3303, 2973, 1706, 1639, 1498, 1365, 1174, 736, 700; ESI HRMS *m/z* $(M+Na)^+$ calcd 587.3204, obsd 587.3232.

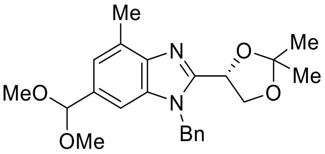

(*S*)-1-Benzyl-6-(dimethoxymethyl)-2-(2,2-dimethyl-1,3-dioxolan-4-yl)-4-methyl-1*H*-benzo[*d*]imidazole (**24**). The structure was confirmed by nuclear Overhauser effect (NOE) experiment. Yield = 63%, electricity = 4.4 F mol^{-1}; White solid; ^1H NMR (500 MHz, $CDCl_3$) δ 7.30–7.24 (m, 3H), 7.20 (s, 1H), 7.15 (s, 1H), 7.10–7.06 (m, 2H), 5.61, 5.51 (ABq, J_{AB} = 16.5 Hz, 2H), 5.41 (s, 1H), 5.29 (t, *J* = 6.9 Hz, 1H), 4.69 (dd, *J* = 8.4, 6.8 Hz, 1H), 4.38 (dd, *J* = 8.4, 6.8 Hz, 1H), 3.29 (s, 6H), 2.67 (s, 3H), 1.43 (s, 3H), 1.36 (s, 3H); ^{13}C NMR (126 MHz, $CDCl_3$) δ 151.2, 141.9, 136.6, 135.9, 133.5, 130.2, 128.9, 127.8, 126.6, 121.7, 110.8, 106.1, 103.8, 71.6, 67.8, 53.0, 47.5, 26.2, 25.5, 16.8; IR (neat, cm^{-1}): 2993, 2924, 1687, 1454, 1371, 1121, 844; ESI HRMS *m/z* $(M+Na)^+$ calcd 419.1941, obsd 419.1961.

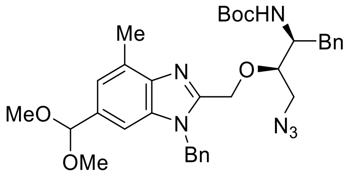

***t*ert-Butyl ((2*S*,3*R*)-4-azido-3-((1-benzyl-6-(dimethoxymethyl)-4-methyl-1*H*-benzo[*d*]imidazol-2-yl)methoxy)-1-phenylbutan-2-yl)carbamate** (**25**). The structure was confirmed by nuclear Overhauser effect (NOE) experiment. Yield = 56%, electricity = 4.4 F mol^{-1}; Colorless oil; ^{1}H NMR (600 MHz, $CDCl_3$) δ 7.31–7.26 (m, 4H), 7.22–7.18 (m, 3H), 7.18–7.14 (m, 1H), 7.11–7.05 (m, 2H), 7.05–7.00 (m, 2H), 5.53 (s, 2H), 5.44 (s, 1H), 5.35 (d, *J* = 9.1 Hz, 1H), 5.00 (d, *J* = 12.8 Hz, 1H), 4.85 (d, *J* = 12.8 Hz, 1H), 4.02–3.91 (m, 1H), 3.79–3.72 (m, 1H), 3.40–3.34 (m, 2H), 3.31 (s, 6H), 2.75 (dd, *J* = 14.8, 5.0 Hz, 1H), 2.71 (s, 3H), 2.51 (dd, *J* = 14.1, 9.7 Hz, 1H), 1.30 (s, 9H); ^{13}C NMR (126 MHz, $CDCl_3$) δ 155.6, 150.5, 141.8, 137.8, 136.3, 135.4, 133.8, 130.2, 129.3, 129.1, 128.5, 128.0, 126.5, 126.4, 121.9, 106.2, 103.7, 81.6, 79.5, 65.9, 53.3, 53.0, 52.9, 52.3, 47.4, 36.1, 28.4, 17.0; IR (neat, cm^{-1}): 3376, 2934, 2101, 1705, 1601, 1497, 1054, 830, 737; ESI HRMS *m/z* $(M+Na)^+$ calcd 637.3109, obsd 637.3121.

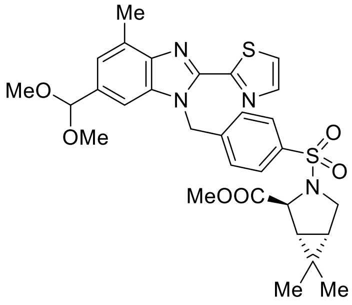

Methyl (1*R*,2*S*,5*S*)-3-((4-((6-(dimethoxymethyl)-4-methyl-2-(thiazol-2-yl)-1*H*-benzo[*d*]imidazol-1-yl)methyl)phenyl)sulfonyl)-6,6-dimethyl-3-azabicyclo[3.1.0]hexane-2-carboxylate (**26**). The structure was confirmed by nuclear Overhauser effect (NOE) experiment. Yield = 60%, electricity = 5.7 F mol^{-1}; Colorless oil; ^{1}H NMR (500 MHz, $CDCl_3$) δ 7.85 (d, *J* = 3.3 Hz, 1H), 7.75–7.66 (m, 2H), 7.46 (d, *J* = 3.3 Hz, 1H), 7.29–7.25 (m, 2H), 7.24 (s, 1H), 7.22 (s, 1H), 6.19 (s, 2H), 5.43 (s, 1H), 4.22 (s, 1H), 3.56 (dd, *J* = 9.2, 5.3 Hz, 1H), 3.49 (s, 3H), 3.38 (d, *J* = 9.3 Hz, 1H), 3.32 (s, 6H), 2.73 (s, 3H), 1.39 (dd, *J* = 7.5, 5.0 Hz, 1H), 1.33 (d, *J* = 7.5 Hz, 1H), 0.95 (s, 3H), 0.75 (s, 3H); ^{13}C NMR (151 MHz, $CDCl_3$) δ 172.2, 159.7, 145.1, 144.0, 142.7, 142.1, 138.6, 136.0, 134.9, 130.7, 127.7, 127.3, 122.7, 121.7, 106.1, 103.5, 61.1, 53.0 (2s), 52.3, 48.1, 47.7, 32.6, 27.8, 26.6, 20.1, 16.9, 12.2; IR (neat, cm^{-1}): 2931, 1743, 1690, 1600, 1450, 1164, 878, 734; ESI HRMS *m/z* $(M+Na)^+$ calcd 633.1812, obsd 633.1829.

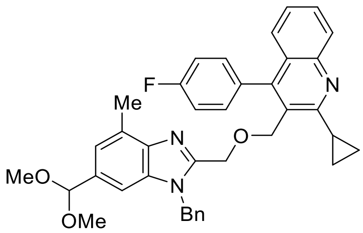

3-(((1-Benzyl-6-(dimethoxymethyl)-4-methyl-1*H*-benzo[*d*]imidazol-2-yl)methoxy)methyl)-2-cyclopropyl-4-(4-fluorophenyl)quinoline (**27**). The structure was confirmed by nuclear Overhauser effect (NOE) experiment. Yield = 55%, electricity = 6.4 F mol^{-1}; Colorless oil; ^{1}H NMR (500 MHz, $CDCl_3$) δ 7.96 (dd, *J* = 8.4, 1.2 Hz, 1H), 7.61 (ddd, *J* = 8.4, 6.7, 1.5 Hz, 1H), 7.32–7.28 (m, 1H), 7.25–7.22 (m, 2H), 7.18 (s, 1H), 7.16–7.12 (m, 2H), 7.12–7.06 (m, 3H), 7.00–6.95 (m, 2H), 6.89–6.85 (m, 2H), 5.45 (s, 1H), 5.40 (s, 2H), 4.66 (s, 2H), 4.62 (s, 2H), 3.31 (s, 6H), 2.65 (s, 3H), 2.45–2.39 (m, 1H), 1.34–1.30 (m, 2H), 0.98–0.92 (m, 2H); ^{13}C NMR (126 MHz, $CDCl_3$) δ 162.9, 162.6 (d, $J_{C\text{-}F}$ = 247.9 Hz), 150.3, 147.7, 147.4, 141.9, 136.2, 135.5, 133.7, 132.3 (d, $J_{C\text{-}F}$ = 3.5 Hz), 131.4 (d, $J_{C\text{-}F}$ = 7.9 Hz), 130.1, 129.4, 129.1, 128.9, 127.9, 126.6, 126.5, 126.4, 126.1, 125.6, 121.8, 115.4 (d, $J_{C\text{-}F}$ = 21.6 Hz), 105.9, 103.7, 67.4, 65.5, 52.9, 47.2, 17.0, 14.5, 10.3; ^{19}F NMR (471 MHz, $CDCl_3$) δ −113.5; IR (neat, cm^{-1}): 2926, 1690, 1514, 1494, 1454, 1222, 1074, 1054, 841, 765; ESI HRMS *m/z* $(M+Na)^+$ calcd 624.2633, obsd 624.2660.

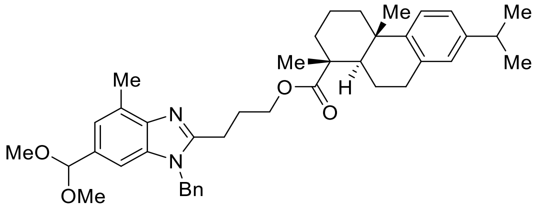

3-(1-Benzyl-6-(dimethoxymethyl)-4-methyl-1*H*-benzo[*d*]imidazol-2-yl)propyl (1*R*,4a*S*,10a*R*)-7-isopropyl-1,4a-dimethyl-1,2,3,4,4a,9,10,10a-octahydrophenanthrene-1-carboxylate (**28**). The structure was confirmed by nuclear Overhauser effect (NOE) experiment. Yield = 46%, electricity = 4.1 F mol^{-1}; Colorless oil; ^{1}H NMR (600 MHz, $CDCl_3$) δ 7.25–7.21 (m, 3H), 7.18 (s, 1H), 7.16 (d, *J* = 8.1 Hz, 1H), 7.14 (s, 1H), 7.01 (dd, *J* = 8.2, 2.1 Hz, 1H), 6.93 (dd, *J* = 7.1, 2.4 Hz, 2H), 6.86 (s, 1H), 5.43 (s, 1H), 5.29 (s, 2H), 4.14 (t, *J* = 6.2 Hz, 2H), 3.30 (s, 6H), 2.90–2.85 (m, 2H), 2.84–2.80 (m, 3H), 2.67 (s, 3H), 2.30–2.25 (m, 1H), 2.20–2.11 (m, 3H), 1.83–1.76 (m, 1H), 1.75–1.69 (m, 1H), 1.68–1.62 (m, 2H), 1.59–1.53 (m, 1H), 1.45–1.39 (m, 1H), 1.38–1.34 (m, 1H), 1.23 (s, 3H), 1.22 (s, 3H), 1.22 (s, 3H), 1.19 (s, 3H); ^{13}C NMR (151 MHz, $CDCl_3$) δ 178.5, 154.2, 147.0, 145.9, 142.3, 136.2, 135.2, 134.7, 132.7, 129.2, 129.1, 128.0, 127.0, 126.1, 124.4, 124.1, 121.5, 105.5, 103.9, 64.1, 52.9, 47.8, 47.0, 45.1, 38.1, 37.1, 36.8, 33.6, 30.3, 27.2, 25.3, 24.6, 24.2, 21.9, 18.7, 17.0, 16.7; IR (neat,

cm^{-1}): 2956, 1721, 1685, 1611, 1459, 1175, 1119; ESI HRMS *m/z* $(M+H)^+$ calcd 638.4073, obsd 638.4071.

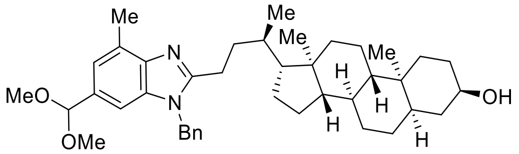

(3*R*,5*R*,8*R*,9*S*,10*S*,13*R*,14*S*,17*R*)-17-((*R*)-4-(1-Benzyl-6-(dimethoxymethyl)-4-methyl-1*H*-benzo[*d*]imidazol-2-yl)butan-2-yl)-10,13-dimethylhexadecahydro-1*H*-cyclopenta[*a*]phenanthren-3-ol (**29**). The structure was confirmed by nuclear Overhauser effect (NOE) experiment. Yield = 52%, electricity = 4.1 F mol^{-1}; White solid; ^{1}H NMR (500 MHz, $CDCl_3$) δ 7.31–7.25 (m, 3H), 7.17 (s, 1H), 7.13 (s, 1H), 7.04–6.99 (m, 2H), 5.42 (s, 1H), 5.33 (s, 2H), 3.67–3.56 (m, 1H), 3.30 (s, 6H), 2.95–2.88 (m, 1H), 2.73–2.69 (m, 1H), 2.69 (d, *J* = 3.0 Hz, 3H), 1.98–1.91 (m, 1H), 1.84 (dt, *J* = 13.3, 6.7 Hz, 3H), 1.81–1.62 (m, 6H), 1.54–1.47 (m, 2H), 1.45–1.31 (m, 7H), 1.27–1.21 (m, 2H), 1.16–1.05 (m, 4H), 1.03–0.98 (m, 2H), 0.95 (d, *J* = 5.9 Hz, 3H), 0.91 (s, 3H), 0.61 (s, 3H); ^{13}C NMR (126 MHz, $CDCl_3$) δ 156.2, 142.3, 136.4, 135.2, 132.4, 129.1, 129.0, 127.9, 126.3, 121.4, 105.5, 104.0, 72.0, 56.6, 56.1, 53.0, 47.1, 42.9, 42.3, 40.6, 40.3, 36.6, 36.2, 36.0, 35.5, 34.7 (2s), 30.7, 28.3, 27.4, 26.6, 25.1, 24.4, 23.5, 21.0, 18.6, 17.1, 12.2; IR (neat, cm^{-1}): 3361, 2923, 1685, 1459, 1260, 1077, 841; ESI HRMS *m/z* $(M+H)^+$ calcd 627.4520, obsd 627.4533.

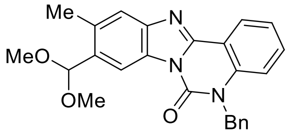

5-Benzyl-9-(dimethoxymethyl)-10-methylbenzo[4,5]imidazo[1,2-*c*]quinazolin-6(5*H*)-one (30). The structure was confirmed by nuclear Overhauser effect (NOE) experiment. Yield = 77%, electricity = 5.9 F mol^{-1}; White solid; ^{1}H NMR (500 MHz, $CDCl_3$) δ 8.64 (s, 1H), 8.49 (d, *J* = 7.8 Hz, 1H), 7.63 (s, 1H), 7.44 (t, *J* = 7.8 Hz, 1H), 7.30–7.25 (m, 5H), 7.23–7.19 (m, 2H), 5.56 (s, 1H), 5.52 (s, 2H), 3.29 (s, 6H), 2.51 (s, 3H); ^{13}C NMR (126 MHz, $CDCl_3$) δ 147.5, 144.1, 137.6, 135.7, 134.6, 132.8, 132.4, 129.5, 129.2, 127.9, 126.7, 125.9, 124.0, 120.8, 115.5, 114.3, 113.8, 101.9, 53.0, 47.3, 19.9; IR (neat, cm^{-1}): 2924, 1698, 1591, 1482, 1354, 1114, 1051, 749, 699; ESI HRMS *m/z* $(M+Na)^+$ calcd 436.1632, obsd 436.1635.

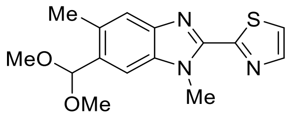

2-(6-(Dimethoxymethyl)-1,5-dimethyl-1*H*-benzo[*d*]imidazol-2-yl)thiazole (**31**). The structure was confirmed by nuclear Overhauser effect (NOE) experiment. Yield = 66%, electricity = 5.5 F mol^{-1}; White solid; ^{1}H NMR (500 MHz, $CDCl_3$) δ 7.96 (d, *J* = 3.2 Hz, 1H),

7.67 (s, 1H), 7.60 (s, 1H), 7.47 (d, *J* = 3.2 Hz, 1H), 5.60 (s, 1H), 4.32 (s, 3H), 3.37 (s, 6H), 2.50 (s, 3H); ^{13}C NMR (101 MHz, $CDCl_3$) δ 160.2, 146.0, 144.0, 142.9, 135.3, 132.6, 131.6, 121.4, 121.2, 108.4, 101.8, 53.2, 32.3, 19.6; IR (neat, cm^{-1}): 2924, 1678, 1614, 1571, 1449, 1333, 1180, 1007; ESI HRMS *m/z* $(M+H)^+$ calcd 304.1114, obsd 304.1127.

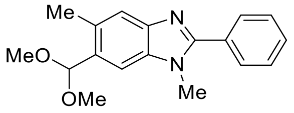

6-(Dimethoxymethyl)-1,5-dimethyl-2-phenyl-1*H*-benzo[*d*]imidazole (32). The structure was confirmed by nuclear Overhauser effect (NOE) experiment. Yield = 67%, electricity = 8.0 F mol^{-1}; White solid; Isolated as a 10:1 mixture of isomers, only the major isomer was shown: ^{1}H NMR (500 MHz, $CDCl_3$) δ 7.77–7.74 (m, 2H), 7.64 (s, 1H), 7.59 (s, 1H), 7.53–7.49 (m, 3H), 5.62 (s, 1H), 3.87 (s, 3H), 3.37 (s, 6H), 2.50 (s, 3H); ^{13}C NMR (126 MHz, $CDCl_3$) δ 154.4, 143.1, 135.0, 131.2, 130.9, 130.4, 129.8, 129.6, 128.8, 121.0, 108.1, 101.9, 53.1, 32.0, 19.5; IR (neat, cm^{-1}): 2920, 1681, 1614, 1469, 1353, 1180, 1132, 777, 702; ESI HRMS *m/z* $(M+Na)^+$ calcd 319.1422, obsd 319.1419.

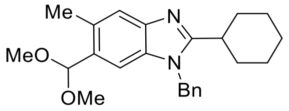

1-Benzyl-2-cyclohexyl-6-(dimethoxymethyl)-5-methyl-1*H*-benzo[*d*]imidazole (33). The structure was confirmed by nuclear Overhauser effect (NOE) experiment. Yield = 69%, electricity = 4.4 F mol^{-1}; Colorless oil; Isolated as a 2.5:1 mixture of regioselective isomers and a major product, only the major isomer was shown: ^{1}H NMR (600 MHz, $CDCl_3$) δ 7.56 (s, 1H), 7.43 (s, 1H), 7.31–7.24 (m, 3H), 7.03–7.00 (m, 2H), 5.51 (s, 1H), 5.36 (s, 2H), 3.27 (s, 6H), 2.77–2.71 (m, 1H), 2.46 (s, 3H), 1.86–1.77 (m, 6H), 1.74–1.69 (m, 1H), 1.35–1.24 (m, 3H); ^{13}C NMR (151 MHz, $CDCl_3$) δ 160.1, 142.9, 136.7, 133.4, 130.4, 130.3, 129.0, 127.9, 126.2, 120.7, 107.9, 102.1, 53.2, 46.8, 36.7, 32.0, 26.5, 25.9, 19.4; IR (neat, cm^{-1}): 2931, 2853, 1686, 1505, 1452, 1192, 1052, 858, 700; ESI HRMS *m/z* $(M+H)^+$ calcd 379.2380, obsd 379.2392.

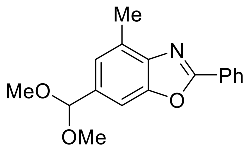

6-(Dimethoxymethyl)-4-methyl-2-phenylbenzo[*d*]oxazole (34). The structure was confirmed by nuclear Overhauser effect (NOE) experiment. Yield = 60%, electricity = 5.0 F mol^{-1}; White solid; ^{1}H NMR (500 MHz, $CDCl_3$) δ 8.30–8.22 (m, 2H), 7.55–7.49 (m, 4H), 7.26 (s, 1H), 5.48 (s, 1H), 3.37 (s, 6H), 2.68 (s, 3H); ^{13}C NMR (214 MHz, $CDCl_3$) δ 163.1, 150.7, 141.8, 135.5, 131.5, 130.3, 129.0, 127.8, 127.5, 123.9, 106.6, 103.2, 53.0, 16.8; IR

(neat, cm^{-1}): 2935, 1615, 1448, 1356, 1266, 1140, 1057, 872, 767, 706; ESI HRMS *m/z* (M+Na)$^+$ calcd 306.1101, obsd 306.1091.

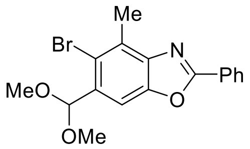

5-Bromo-6-(dimethoxymethyl)-4-methyl-2-phenylbenzo[*d*]oxazole (35). The structure was confirmed by nuclear Overhauser effect (NOE) experiment. Yield = 56%, electricity = 8.0 F mol^{-1}; White solid; ^{1}H NMR (850 MHz, $CDCl_3$) δ 8.28–8.24 (m, 2H), 7.74 (s, 1H), 7.56–7.51 (m, 3H), 5.72 (s, 1H), 3.44 (s, 6H), 2.75 (s, 3H); ^{13}C NMR (214 MHz, $CDCl_3$) δ 163.9, 149.4, 143.0, 134.0, 131.9, 131.4, 129.1, 127.9, 127.1, 120.6, 107.9, 103.8, 54.3, 17.9; IR (neat, cm^{-1}): 2920, 1556, 1447, 1362, 1300, 1109, 1057, 705, 688; ESI HRMS *m/z* (M+Na)$^+$ calcd 384.0206, obsd 384.0206.

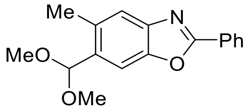

6-(Dimethoxymethyl)-5-methyl-2-phenylbenzo[*d*]oxazole (36). The structure was confirmed by nuclear Overhauser effect (NOE) experiment. Yield = 67%, electricity = 5.5 F mol^{-1}; White solid; ^{1}H NMR (500 MHz, $CDCl_3$) δ 8.26–8.21 (m, 2H), 7.82 (s, 1H), 7.55 (s, 1H), 7.53–7.49 (m, 3H), 5.56 (s, 1H), 3.35 (s, 6H), 2.48 (s, 3H); ^{13}C NMR (126 MHz, $CDCl_3$) δ 163.8, 149.4, 142.2, 133.6, 133.2, 131.6, 129.0, 127.7, 127.4, 121.2, 109.1, 101.3, 53.1, 19.4; IR (neat, cm^{-1}): 2932, 1695, 1552, 1447, 1273, 1120, 1052, 865, 704; ESI HRMS *m/z* (M+Na)$^+$ calcd 306.1101, obsd 306.1101.

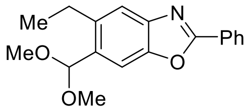

6-(Dimethoxymethyl)-5-ethyl-2-phenylbenzo[*d*]oxazole (**37**). Yield = 70%, electricity = 4.7 F mol^{-1}; White solid; ^{1}H NMR (600 MHz, $CDCl_3$) δ 8.26–8.23 (m, 2H), 7.83 (s, 1H), 7.61 (s, 1H), 7.53–7.50 (m, 3H), 5.62 (s, 1H), 3.36 (s, 6H), 2.85 (q, *J* = 7.5 Hz, 2H), 1.30 (t, *J* = 7.6 Hz, 3H); ^{13}C NMR (151 MHz, $CDCl_3$) δ 163.8, 149.2, 142.4, 139.4, 133.1, 131.6, 129.0, 127.7, 127.4, 119.5, 109.1, 101.1, 53.2, 25.2, 15.7.IR (neat, cm^{-1}): 2916, 1730, 1635, 1464, 1271, 705; ESI HRMS *m/z* (M+Na)$^+$ calcd 320.1257, obsd 320.1255.

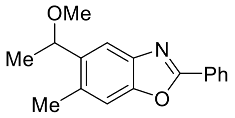

5-(1-Methoxyethyl)-6-methyl-2-phenylbenzo[*d*]oxazole (**37'**). Yield = 7%, electricity = 4.7 F mol^{-1}; White solid; ^{1}H NMR (500 MHz, $CDCl_3$) δ 8.27–8.21 (m, 2H), 7.82 (s, 1H),

7.55–7.50 (m, 3H), 7.36 (s, 1H), 4.64 (q, *J* = 6.4 Hz, 1H), 3.27 (s, 3H), 2.47 (s, 3H), 1.46 (d, *J* = 6.4 Hz, 3H); ^{13}C NMR (151 MHz, $CDCl_3$) δ 163.0, 150.1, 141.0, 139.0, 133.1, 131.5, 129.1, 127.7, 127.5, 116.7, 111.9, 76.3, 56.6, 23.1, 19.9.

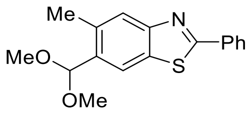

6-(Dimethoxymethyl)-5-methyl-2-phenylbenzo[*d*]thiazole (38). The structure was confirmed by conversion to aldehyde **39**, which was characterized by X-ray crystal structural analysis. Yield = 75%, electricity = 6.8 F mol^{-1}; White solid; ^{1}H NMR (500 MHz, $CDCl_3$) δ 8.11 (s, 1H), 8.09–8.06 (m, 2H), 7.87 (s, 1H), 7.50–7.45 (m, 3H), 5.58 (s, 1H), 3.36 (s, 6H), 2.52 (s, 3H); ^{13}C NMR (126 MHz, $CDCl_3$) δ 169.0, 154.4, 135.2, 133.9, 133.7, 132.4, 131.0, 129.1, 127.7, 124.6, 119.9, 101.4, 53.0, 19.4; IR (neat, cm^{-1}): 2930, 1695, 1480, 1448, 1352, 1195, 1104, 1054, 764, 688; ESI HRMS *m/z* (M+Na)$^+$ calcd 322.0872, obsd 322.0869.

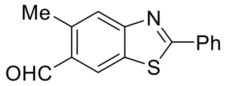

5-Methyl-2-phenylbenzo[*d*]thiazole-6-carbaldehyde (39). After electrolysis under the standard conditions, the reaction mixture was cooled to RT and 2 N HCl (2 mL) was then added. After being stirred at RT for 30 min, the reaction mixture was concentrated under reduced pressure. The residue was chromatographed through silica gel eluting with ethyl acetate/hexanes to give **39**. Yield = 57%, two steps; White solid; ^{1}H NMR (500 MHz, $CDCl_3$) δ 10.34 (s, 1H), 8.33 (s, 1H), 8.12–8.06 (m, 2H), 7.90 (s, 1H), 7.55–7.47 (m, 3H), 2.80 (s, 3H); ^{13}C NMR (126 MHz, $CDCl_3$) δ 191.6, 172.8, 157.7, 138.7, 133.3, 133.1, 131.9, 131.7, 129.3, 128.0, 126.2, 125.7, 20.1; IR (neat, cm^{-1}): 2918, 1678, 1592, 1475, 1180, 1132, 1076, 765; ESI HRMS *m/z* (M+H)$^+$ calcd 254.0634, obsd 254.0639. This compound was further characterized by X-ray structural analysis.

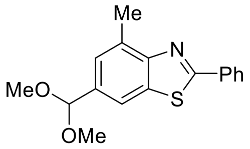

6-(Dimethoxymethyl)-4-methyl-2-phenylbenzo[*d*]thiazole (40). The structure was confirmed by nuclear Overhauser effect (NOE) experiment. Yield = 63%, electricity = 6.3 F mol^{-1}; White solid; ^{1}H NMR (500 MHz, $CDCl_3$) δ 8.13–8.09 (m, 2H), 7.85 (s, 1H), 7.50–7.47 (m, 3H), 7.37 (s, 1H), 5.49 (s, 1H), 3.38 (s, 6H), 2.82 (s, 3H); ^{13}C NMR (126 MHz, $CDCl_3$) δ 167.4, 153.8, 135.4, 135.1, 134.1, 133.3, 130.9, 129.1, 127.7, 125.7, 117.5, 103.2, 53.0, 18.6; IR (neat, cm^{-1}): 2929, 1694, 1480, 1445, 1347, 1134, 1055, 865, 763, 688; ESI HRMS *m/z* (M+Na)$^+$ calcd 322.0872, obsd 322.0864.

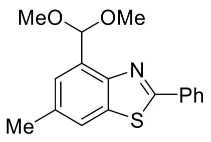

4-(Dimethoxymethyl)-6-methyl-2-phenylbenzo[*d*]thiazole (40'). Yield = 12%, electricity = 6.3 F mol^{-1}; White solid; ^{1}H NMR (850 MHz, $CDCl_3$) δ 8.14–8.09 (m, 2H), 7.68 (s, 1H), 7.52 (s, 1H), 7.50–7.47 (m, 3H), 6.31 (s, 1H), 3.51 (s, 6H), 2.51 (s, 3H); ^{13}C NMR (214 MHz, $CDCl_3$) δ 167.0, 150.4, 135.7, 135.4, 134.0, 132.1, 130.9, 129.1, 127.8, 125.0, 121.7, 101.5, 54.6, 21.9; IR (neat, cm^{-1}): 2922, 1693, 1480, 1272, 1099, 861, 761, 684; ESI HRMS *m/z* $(M+Na)^+$ calcd 322.0872, obsd 322.0861.

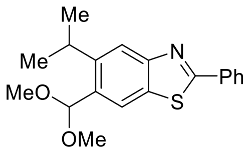

6-(Dimethoxymethyl)-5-isopropyl-2-phenylbenzo[*d*]thiazole (41). Yield = 73%, electricity = 5.0 F mol^{-1}; White solid; ^{1}H NMR (600 MHz, $CDCl_3$) δ 8.13 (s, 1H), 8.11–8.08 (m, 2H), 8.06 (s, 1H), 7.50–7.46 (m, 3H), 5.70 (s, 1H), 3.44 (hept, *J* = 6.9 Hz, 1H), 3.36 (s, 6H), 1.34 (d, *J* = 6.9 Hz, 6H); ^{13}C NMR (151 MHz, $CDCl_3$) δ 169.1, 154.9, 146.6, 133.9, 132.4, 132.2, 131.1, 129.2, 127.7, 120.3, 120.0, 101.0, 53.1, 28.5, 24.6; IR (neat, cm^{-1}): 2931, 1513, 1481, 1443, 1352, 1194, 1102, 1052, 763, 688; ESI HRMS *m/z* $(M+H)^+$ calcd 328.1366, obsd 328.1367.

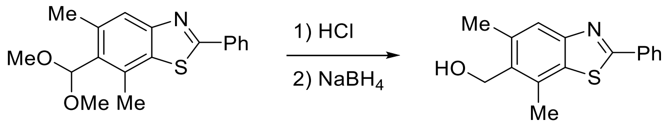

(5,7-Dimethyl-2-phenylbenzo[*d*]thiazol-6-yl)methanol (43a). Compounds **42** and **43** cannot be separated by column chromatography. For characterization purpose, compound **43** was converted to **43a** and then separated from **42**. After electrolysis (electricity = 5.0 F mol^{-1}), The crude mixture of **42** and **43** were treated with 2 N HCl (2 mL) and stirred at RT for 30 min. The reaction mixture was then concentrated under reduced pressure. The residue was dissolved in THF (5.0 mL) band treated with $NaBH_4$ (15.1 mg, 0.4 mmol) at 0 °C. The reaction mixture was warmed to RT and stireed for 30 min. Water (2.0 mL) was added to quench the reaction. The mixture was extracted with ethyl acetate (3 x 5 mL). The combined organic solution was dried over anhydrous Na_2SO_4, filtered and evaporated under reduced pressure. The residue was purified by silica gel column chromatography to afford the product **43a** as a white solid solid (10 mg, 19% yield) and product **42** as a white solid (30 mg, 53% yield). For product **43a**, the structure was confirmed by nuclear Overhauser effect (NOE)

experiment. ^{1}H NMR (600 MHz, $CDCl_3$) δ 8.11–8.06 (m, 2H), 7.74 (s, 1H), 7.51–7.47 (m, 3H), 4.86 (s, 2H), 2.66 (s, 3H), 2.58 (s, 3H), 1.56 (s, 1H); ^{13}C NMR (151 MHz, $CDCl_3$) δ 168.4, 153.5, 136.6, 134.8, 133.9, 133.7, 131.1, 131.0, 129.2, 127.6, 122.5, 59.4, 20.4, 19.4; IR (neat, cm^{-1}): 3363, 2921, 1478, 1444, 1378, 1247, 1165, 1032, 763, 688; ESI HRMS *m/z* $(M+H)^+$ calcd 270.0947, obsd 270.0950.

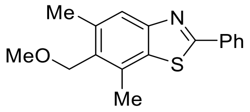

6-(Methoxymethyl)-5,7-dimethyl-2-phenylbenzo[*d*]thiazole (42). The structure was confirmed by nuclear Overhauser effect (NOE) experiment. ^{1}H NMR (600 MHz, $CDCl_3$) δ 8.12–8.07 (m, 2H), 7.77 (s, 1H), 7.51–7.47 (m, 3H), 4.60 (s, 2H), 3.45 (s, 3H), 2.65 (s, 3H), 2.56 (s, 3H); ^{13}C NMR (126 MHz, $CDCl_3$) δ 168.2, 153.6, 137.1, 134.7, 134.0, 131.5, 131.4, 131.0, 129.1, 127.6, 122.3, 68.7, 58.5, 20.4, 19.5; IR (neat, cm^{-1}): 2923, 1682, 1478, 1375, 1167, 1092, 862, 763, 688; ESI HRMS *m/z* $(M+Na)^+$ calcd 306.0923, obsd 306.0917.

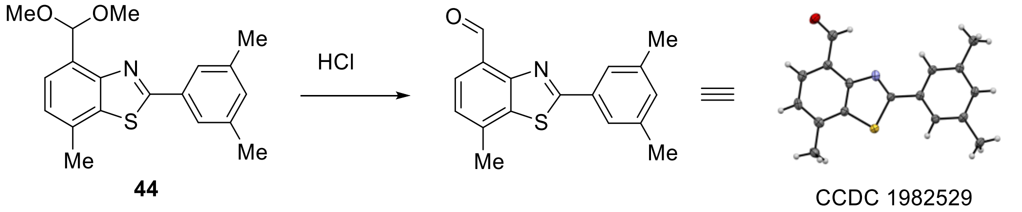

4-(Dimethoxymethyl)-2-(3,5-dimethylphenyl)-7-methylbenzo[*d*]thiazole (44). The structure was confirmed by hydrolyzing to the corresponding aldehyde and characterized by X-ray structural analysis. Yield = 57%, electricity = 8.4 F mol^{-1}; White solid; ^{1}H NMR (850 MHz, $CDCl_3$) δ 7.76 (s, 2H), 7.62 (d, J = 7.5 Hz, 1H), 7.21 (d, J = 7.5 Hz, 1H), 7.12 (s, 1H), 6.35 (s, 1H), 3.49 (s, 6H), 2.59 (s, 3H), 2.42 (s, 6H); ^{13}C NMR (151 MHz, $CDCl_3$) δ 168.0, 152.0, 138.7, 135.8, 133.8, 132.8, 132.0, 129.8, 125.6, 125.1, 124.0, 101.3, 54.1, 21.6, 21.4; IR (neat, cm^{-1}): 2927, 1605, 1504, 1443, 1307, 1191, 1105, 1057, 810, 691; ESI HRMS *m/z* $(M+Na)^+$ calcd 350.1185, obsd 350.1179.

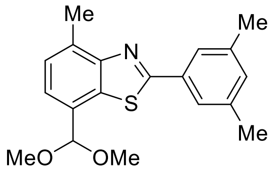

7-(Dimethoxymethyl)-2-(3,5-dimethylphenyl)-4-methylbenzo[*d*]thiazole (44'). Yield = 10%; White solid; ^{1}H NMR (850 MHz, $CDCl_3$) δ 7.77 (s, 2H), 7.37 (d, J = 7.5 Hz, 1H), 7.29 (d, J = 7.5 Hz, 1H), 7.11 (s, 1H), 5.60 (s, 1H), 3.39 (s, 6H), 2.82 (s, 3H), 2.41 (s, 6H); ^{13}C NMR (214 MHz, $CDCl_3$) δ 168.7, 154.4, 138.7, 134.0, 133.7, 132.6, 132.2, 129.8, 126.7,

125.5, 123.6, 102.7, 53.0, 21.5, 18.5; IR (neat, cm^{-1}): 2922, 1682, 1504, 1446, 1352, 1191, 1112, 1056, 850, 808, 691; ESI HRMS *m/z* $(M+Na)^+$ calcd 350.1185, obsd 350.1182.

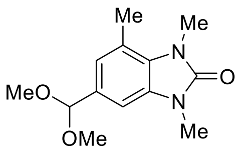

6-(Dimethoxymethyl)-1,3,4-trimethyl-1,3-dihydro-2*H*-benzo[*d*]imidazol-2-one (**45**). The structure was confirmed by nuclear Overhauser effect (NOE) experiment. Yield = 84%, electricity = 4.5 F mol^{-1}; Colorless oil; ^1H NMR (500 MHz, $CDCl_3$) δ 6.94 (s, 1H), 6.91 (s, 1H), 5.36 (s, 1H), 3.66 (s, 3H), 3.41 (s, 3H), 3.35 (s, 6H), 2.62 (s, 3H); ^{13}C NMR (101 MHz, $CDCl_3$) δ 155.3, 131.5, 130.4, 128.2, 123.1, 119.1, 103.9, 103.6, 53.0, 30.0, 27.4, 18.3; IR (neat, cm^{-1}): 2926, 1709, 1683, 1599, 1471, 1094, 974, 747; ESI HRMS *m/z* $(M+H)^+$ calcd 251.1390, obsd 251.1385.

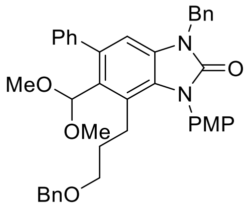

1-Benzyl-4-(3-(benzyloxy)propyl)-5-(dimethoxymethyl)-3-(4-methoxyphenyl)-6-phenyl-1,3-dihydro-2*H*-benzo[*d*]imidazol-2-one (46). Yield = 67%, electricity = 9.9 F mol^{-1}; Light yellow oil; ^1H NMR (500 MHz, $CDCl_3$) δ 7.43–7.23 (m, 17H), 6.99–6.92 (m, 2H), 6.71 (s, 1H), 5.08 (s, 2H), 5.06 (s, 1H), 4.35 (s, 2H), 3.81 (s, 3H), 3.18 (s, 6H), 2.92 (t, *J* = 7.1 Hz, 2H), 2.82–2.67 (m, 2H), 1.63–1.54 (m, 2H); ^{13}C NMR (126 MHz, $CDCl_3$) δ 159.9, 155.7, 142.1, 139.1, 137.2, 136.3, 130.7, 129.9, 129.7, 129.0, 128.9, 128.6, 128.5, 128.4, 128.2, 127.9, 127.8, 127.7, 127.5, 127.2, 125.7, 114.5, 107.5, 106.8, 72.3, 70.5, 55.7, 45.1, 31.0, 23.9; IR (neat, cm^{-1}): 3060, 2960, 2930, 1716, 1513, 1250, 701; ESI HRMS *m/z* $(M+Na)^+$ calcd 651.2829, obsd 651.2820.

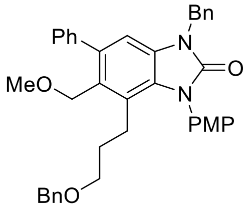

1-Benzyl-4-(3-(benzyloxy)propyl)-5-(methoxymethyl)-3-(4-methoxyphenyl)-6-phenyl-1,3-dihydro-2*H*-benzo[*d*]imidazol-2-one (46'). Yield = 74%, electricity = 4.1 F mol^{-1}; Light yellow solid; ^1H NMR (500 MHz, $CDCl_3$) δ 7.44–7.27 (m, 17H), 6.99–6.96 (m, 2H), 6.80 (s, 1H), 5.10 (s, 2H), 4.38 (s, 2H), 4.18 (s, 2H), 3.83 (s, 3H), 3.22 (s, 3H), 3.02 (t, *J* = 6.4 Hz, 2H), 2.59–2.50 (m, 2H), 1.62–1.56 (m, 2H); ^{13}C NMR (126 MHz, $CDCl_3$) δ 160.0, 155.5,

142.0, 138.7, 138.3, 136.2, 130.7, 129.9, 129.7, 129.0, 128.9, 128.4, 128.0 (2s), 127.8, 127.7 (2s), 127.6, 127.5, 127.1, 125.7, 114.7, 108.1, 72.6, 70.0, 69.1, 58.0, 55.7, 45.1, 31.5, 23.6; IR (neat, cm^{-1}): 3060, 2926, 1715, 1513, 1250, 700; ESI HRMS *m/z* $(M+Na)^+$ calcd 621.2724, obsd 621.2703.

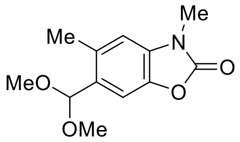

6-(Dimethoxymethyl)-3,5-dimethylbenzo[*d*]oxazol-2(3*H*)-one (47). The structure was confirmed by nuclear Overhauser effect (NOE) experiment. Yield = 86%, electricity = 4.4 F mol^{-1}; Colorless oil; ^1H NMR (400 MHz, $CDCl_3$) δ 7.43 (s, 1H), 6.75 (s, 1H), 5.46 (s, 1H), 3.38 (s, 3H), 3.30 (s, 6H), 2.40 (s, 3H); ^{13}C NMR (101 MHz, $CDCl_3$) δ 155.3, 140.9, 132.6, 131.6, 130.7, 109.9, 108.7, 101.0, 52.9, 28.2, 19.2; IR (neat, cm^{-1}): 2936, 1780, 1620, 1497, 1285, 1098, 1053, 881, 750; ESI HRMS *m/z* $(M+Na)^+$ calcd 260.0893, obsd 260.0890.

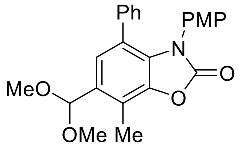

6-(Dimethoxymethyl)-3-(4-methoxyphenyl)-7-methyl-4-phenylbenzo[*d*]oxazol-2(3*H*)-one (48). The structure was confirmed by nuclear Overhauser effect (NOE) experiment. Yield = 75%, electricity = 6.0 F mol^{-1}; Pale yellow oil; ^1H NMR (500 MHz, $CDCl_3$) δ 7.34 (s, 1H), 7.10–7.04 (m, 1H), 7.01–6.97 (m, 2H), 6.93–6.90 (m, 2H), 6.89–6.85 (m, 2H), 6.59–6.55 (m, 2H), 5.50 (s, 1H), 3.71 (s, 3H), 3.36 (s, 6H), 2.49 (s, 3H); ^{13}C NMR (126 MHz, $CDCl_3$) δ 159.2, 154.6, 142.3, 135.9, 131.1, 129.2, 127.9 (2s), 127.6, 127.0, 126.7, 124.9, 122.7, 118.6, 114.0, 102.0, 55.7, 53.5, 10.9; IR (neat, cm^{-1}): 2918, 2849, 1958, 1778, 1658, 1250, 1076, 948; ESI HRMS *m/z* $(M+Na)^+$ calcd 428.1468, obsd 428.1464.

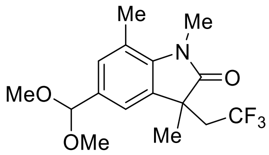

5-(Dimethoxymethyl)-1,3,7-trimethyl-3-(2,2,2-trifluoroethyl)indolin-2-one (49). The structure was confirmed by nuclear Overhauser effect (NOE) experiment. Yield = 84%, electricity = 4.7 F mol^{-1}; White solid; ^1H NMR (500 MHz, $CDCl_3$) δ 7.18 (s, 1H), 7.12 (s, 1H), 5.35 (s, 1H), 3.51 (s, 3H), 3.31 (s, 3H), 3.29 (s, 3H), 2.90–2.81 (m, 1H), 2.68–2.61 (m, 1H), 2.60 (s, 3H), 1.38 (s, 3H); ^{13}C NMR (101 MHz, $CDCl_3$) δ 179.5, 140.9, 132.7, 131.7, 130.9, 125.3 (q, $J_{C\text{-}F}$ = 278.4 Hz), 119.9, 102.9, 52.7, 52.6, 43.9 (q, $J_{C\text{-}F}$ = 2.0 Hz), 41.1 (q, $J_{C\text{-}F}$ = 28.2 Hz), 29.9, 25.7, 19.2; ^{19}F NMR (471 MHz, $CDCl_3$) δ −62.1 (t, J = 10.5); IR (neat,

cm^{-1}): 2975, 1717, 1683, 1594, 1457, 1342, 1074, 952, 894, 616; ESI HRMS *m/z* $(M+Na)^+$ calcd 354.1287, obsd 354.1292.

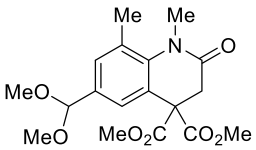

Dimethyl 6-(dimethoxymethyl)-1,8-dimethyl-2-oxo-2,3-dihydroquinoline-4,4(1*H*)-dicarboxylate (**50**). The structure was confirmed by nuclear Overhauser effect (NOE) experiment. Yield = 96%, electricity = 5.2 F mol^{-1}; Colorless oil; ^{1}H NMR (500 MHz, $CDCl_3$) δ 7.28 (d, *J* = 1.9 Hz, 1H), 7.10 (d, *J* = 1.9 Hz, 1H), 5.33 (s, 1H), 3.81 (s, 6H), 3.33 (s, 6H), 3.27 (s, 3H), 3.16 (s, 2H), 2.37 (s, 3H); ^{13}C NMR (126 MHz, $CDCl_3$) δ 169.7, 169.6, 140.8, 134.5, 131.2, 128.5, 127.6, 123.0, 102.5, 57.9, 53.6, 53.6, 52.9, 39.6, 36.1, 21.1; IR (neat, cm^{-1}): 2925, 1709, 1683, 1600, 1471, 1378, 1263,1202, 1094; ESI HRMS *m/z* $(M+Na)^+$ calcd 388.1367, obsd 388.1374.

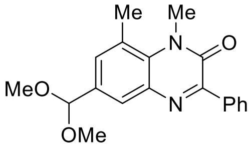

6-(Dimethoxymethyl)-1,8-dimethyl-3-phenylquinoxalin-2(1*H*)-one (**51**). The structure was confirmed by nuclear Overhauser effect (NOE) experiment. Yield = 55%, electricity = 8.3 F mol^{-1}; White solid; ^{1}H NMR (500 MHz, $CDCl_3$) δ 8.35–8.27 (m, 2H), 8.12 (s, 1H), 7.50–7.44 (m, 3H), 7.11 (s, 1H), 5.52 (s, 1H), 3.74 (s, 3H), 3.35 (s, 6H), 2.54 (s, 3H); ^{13}C NMR (101 MHz, $CDCl_3$) δ 155.0, 153.4, 139.8, 136.3, 133.2, 132.1, 131.2, 130.3, 129.6, 129.1, 128.2, 115.3, 101.2, 53.0, 29.4, 19.9; IR (neat, cm^{-1}): 2930, 1651, 1618, 1572, 1460, 1278, 1054, 806, 692; ESI HRMS *m/z* $(M+Na)^+$ calcd 347.1366, obsd 347.2370.

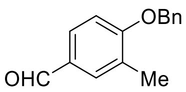

4-(Benzyloxy)-3-methylbenzaldehyde (52). After electrolysis under the standard conditions (Electricity = 3.9 F mol^{-1}), the reaction mixture was cooled to RT and 2 N HCl (2 mL) was then added. After being stirred at RT for 30 min, the reaction mixture was concentrated under reduced pressure. The residue was chromatographed through silica gel eluting with ethyl acetate/hexanes to give **52**. Yield = 67%, two steps; Pale yelleow solid; ^{1}H NMR (500 MHz, $CDCl_3$) δ 9.86 (s, 1H), 7.72 (dd, *J* = 2.1, 1.0 Hz, 1H), 7.69 (dd, *J* = 8.3, 2.1 Hz, 1H), 7.47–7.38 (m, 4H), 7.38–7.33 (m, 1H), 6.99 (d, *J* = 8.3 Hz, 1H), 5.18 (s, 2H), 2.34 (s, 3H); ^{13}C NMR (126 MHz, $CDCl_3$) δ 191.2, 162.1, 136.5, 131.8, 130.7, 129.8, 128.8, 128.3, 128.1, 127.2, 111.1, 70.2, 16.5. The title compound is known in the literature[21].

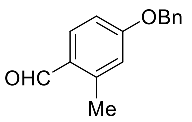

4-(Benzyloxy)-2-methylbenzaldehyde (53). After electrolysis under the standard conditions (Electricity = 4.7 F mol^{-1}), the reaction mixture was cooled to RT and 2 N HCl (2 mL) was then added. After being stirred at RT for 30 min, the reaction mixture was concentrated under reduced pressure. The residue was chromatographed through silica gel eluting with ethyl acetate/hexanes to give **53**. Yield = 43%, two steps; Light yellow oil; ^{1}H NMR (500 MHz, $CDCl_3$) δ 10.11 (s, 1H), 7.75 (d, *J* = 8.5 Hz, 1H), 7.44–7.37 (m, 4H), 7.36–7.33 (m, 1H), 6.91 (dd, *J* = 8.5, 2.5 Hz, 1H), 6.83 (d, *J* = 2.5 Hz, 1H), 5.12 (s, 2H), 2.64 (s, 3H); ^{13}C NMR (126 MHz, $CDCl_3$) δ 191.3, 162.9, 143.5, 136.3, 134.9, 128.9, 128.4, 128.3, 127.6, 118.0, 112.4, 70.3, 20.1. The title compound is known in the literature[22].

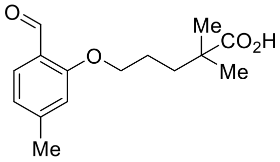

5-(2-Formyl-5-methylphenoxy)-2,2-dimethylpentanoic acid (54). After electrolysis under the standard conditions (Electricity = 4.2 F mol^{-1}), the reaction mixture was cooled to RT and 2 N HCl (2 mL) was then added. After being stirred at RT for 30 min, the reaction mixture was concentrated under reduced pressure. The residue was chromatographed through silica gel eluting with ethyl acetate/hexanes to give **54**. Yield = 61%, two steps; Light yellow oil; ^{1}H NMR (500 MHz, $CDCl_3$) δ 10.43 (s, 1H), 7.72 (d, *J* = 7.8 Hz, 1H), 6.82 (d, *J* = 7.8 Hz, 1H), 6.75 (s, 1H), 4.05 (t, *J* = 6.1 Hz, 2H), 2.38 (s, 3H), 1.89–1.83 (m, 2H), 1.78–1.73 (m, 2H), 1.26 (s, 6H). The title compound is known in the literature[23].

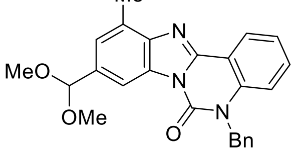

5-Benzyl-9-(dimethoxymethyl)-11-methylbenzo[4,5]imidazo[1,2-*c*]quinazolin-6(5*H*)-one (56). The structure was confirmed by nuclear Overhauser effect (NOE) experiment. Yield = 80%, electricity = 8.0 F mol^{-1} (71% yield for 10-gram scale, 8.1 F mol^{-1}); White solid; ^{1}H NMR (500 MHz, $CDCl_3$) δ 8.61 (dd, *J* = 7.8, 1.6 Hz, 1H), 8.46 (s, 1H), 7.51 (ddd, *J* = 8.7, 7.3, 1.6 Hz, 1H), 7.45 (s, 1H), 7.36–7.32 (m, 5H), 7.30–7.27 (m, 2H), 5.58 (s, 2H), 5.53 (s, 1H), 3.39 (s, 6H), 2.79 (s, 3H); ^{13}C NMR (126 MHz, $CDCl_3$) δ 147.7, 146.8, 143.6, 137.4, 135.8, 134.7, 132.3, 131.0, 129.6, 129.2, 127.9, 126.7, 126.0, 124.6, 123.9, 115.5, 114.0, 111.9, 103.9, 53.2, 47.3, 17.1; IR (neat, cm^{-1}): 2939, 1702, 1518, 1480, 1357, 1133, 749; ESI HRMS

m/z (M+Na)$^+$ calcd 436.1632, obsd 436.1627.

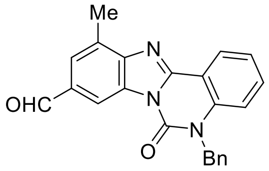

5-Benzyl-11-methyl-6-oxo-5,6-dihydrobenzo[4,5]imidazo[1,2-*c*]quinazoline-9-carbaldehyde (57). After electrolysis under the standard conditions, the reaction mixture was cooled to RT and 2 N HCl (2 mL) was then added. After being stirred at RT for 30 min, the reaction mixture was concentrated under reduced pressure. The residue was chromatographed through silica gel eluting with ethyl acetate/hexanes to give **57**. Yield = 77%, two steps; White solid; ^{1}H NMR (500 MHz, $CDCl_3$) δ 10.04 (s, 1H), 8.74 (s, 1H), 8.56 (dd, *J* = 7.9, 1.5 Hz, 1H), 7.82 (s, 1H), 7.55 (ddd, *J* = 8.7, 7.3, 1.5 Hz, 1H), 7.38–7.31 (m, 6H), 7.30–7.28 (m, 1H), 5.57 (s, 2H), 2.77 (s, 3H); ^{13}C NMR (126 MHz, $CDCl_3$) δ 191.8, 149.0, 147.9, 147.3, 137.8, 135.4, 133.1, 132.7, 131.1, 130.6, 129.3, 128.1, 126.7, 126.3, 125.7, 124.2, 117.2, 115.6, 113.4, 47.4, 16.9; IR (neat, cm^{-1}): 3362, 2919, 1699, 1481, 1355, 1116, 748; ESI HRMS m/z (M+Na)$^+$ calcd 390.1218, obsd 390.1218.

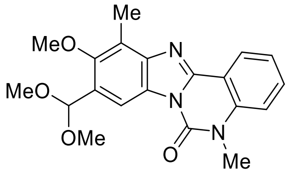

9-(Dimethoxymethyl)-10-methoxy-5,11-dimethylbenzo[4,5]imidazo[1,2-*c*]quinazolin-6(5*H*)-one (58). The structure was confirmed by nuclear Overhauser effect (NOE) experiment. Yield = 86%, electricity = 6.5 F mol^{-1}; White solid; ^{1}H NMR (500 MHz, $CDCl_3$) δ 8.56 (dd, *J* = 7.8, 1.6 Hz, 1H), 8.50 (s, 1H), 7.62 (ddd, *J* = 8.6, 7.3, 1.6 Hz, 1H), 7.37 (t, *J* = 7.5 Hz, 1H), 7.32 (d, *J* = 8.3 Hz, 1H), 5.81 (s, 1H), 3.89 (s, 3H), 3.78 (s, 3H), 3.41 (s, 6H), 2.71 (s, 3H); ^{13}C NMR (126 MHz, $CDCl_3$) δ 154.5, 147.2, 147.1, 144.7, 138.1, 132.3, 128.2, 127.4, 125.9, 123.8, 122.0, 114.5, 113.6, 111.7, 100.1, 62.0, 53.4, 30.6, 10.9; IR (neat, cm^{-1}): 2921, 1698, 1612, 1301, 1112, 747; ESI HRMS m/z (M+Na)$^+$ calcd 390.1424, obsd 390.1416.

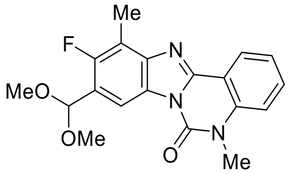

9-(Dimethoxymethyl)-10-fluoro-5,11-dimethylbenzo[4,5]imidazo[1,2-*c*]quinazolin-6(5*H*)-one (59). The structure was confirmed by nuclear Overhauser effect (NOE) experiment. Yield = 84%, electricity = 6.5 F mol^{-1}; White solid; ^{1}H NMR (500 MHz, $CDCl_3$) δ 8.49 (d, *J* = 7.7

Hz, 1H), 8.45 (d, J = 6.1 Hz, 1H), 7.58 (t, J = 7.8 Hz, 1H), 7.33 (t, J = 7.6 Hz, 1H), 7.27 (d, J = 8.2 Hz, 1H), 5.74 (s, 1H), 3.74 (s, 3H), 3.42 (s, 6H), 2.63 (s, 3H); ^{13}C NMR (126 MHz, $CDCl_3$) δ 156.8 (d, $J_{C\text{-}F}$ = 242.6 Hz), 147.7, 146.9, 144.3 (d, $J_{C\text{-}F}$ = 9.8 Hz), 138.0, 132.4, 126.8, 125.8, 123.8, 122.2 (d, $J_{C\text{-}F}$ = 16.5 Hz), 115.5 (d, $J_{C\text{-}F}$ = 20.9 Hz), 114.5, 113.3, 111.6 (d, $J_{C\text{-}F}$ = 5.0 Hz), 99.3 (d, $J_{C\text{-}F}$ = 4.4 Hz), 53.5, 30.5, 9.5 (d, $J_{C\text{-}F}$ = 4.4 Hz); ^{19}F NMR (471 MHz, $CDCl_3$) δ −127.1 (d, J = 9.4 Hz); IR (neat, cm^{-1}): 2929, 1705, 1590, 1483, 1306, 1127, 1076, 749; ESI HRMS *m/z* $(M+Na)^+$ calcd 378.1224, obsd 378.1217.

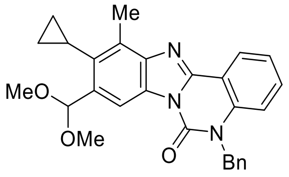

5-Benzyl-10-cyclopropyl-9-(dimethoxymethyl)-11-methylbenzo[4,5]imidazo[1,2-*c*]quinazolin-6(5*H*)-one (60). The structure was confirmed by nuclear Overhauser effect (NOE) experiment. Yield = 70%, electricity = 8.0 F mol^{-1}; White solid; ^{1}H NMR (500 MHz, $CDCl_3$) δ 8.63 (s, 1H), 8.61 (dd, J = 7.8, 1.6 Hz, 1H), 7.49 (ddd, J = 8.7, 7.2, 1.6 Hz, 1H), 7.37–7.32 (m, 5H), 7.31–7.27 (m, 2H), 6.16 (s, 1H), 5.58 (s, 2H), 3.42 (s, 6H), 2.91 (s, 3H), 2.03–1.98 (m, 1H), 1.20–1.15 (m, 2H), 0.77–0.72 (m, 2H); ^{13}C NMR (126 MHz, $CDCl_3$) δ 147.5, 146.7, 143.9, 137.5, 136.3, 135.9, 135.5, 132.1, 130.6, 129.4, 129.1, 127.8, 126.7, 126.0, 123.8, 115.4, 114.0, 111.2, 101.7, 53.7, 47.1, 15.1, 11.7, 8.4; IR (neat, cm^{-1}): 2921, 1651, 1530, 1462, 1195, 1042, 857, 751; ESI HRMS *m/z* $(M+H)^+$ calcd 454.2125, obsd 454.2115.

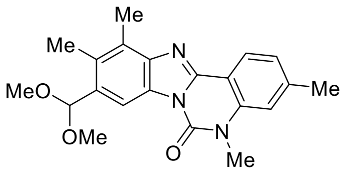

9-(Dimethoxymethyl)-3,5,10,11-tetramethylbenzo[4,5]imidazo[1,2-*c*]quinazolin-6(5*H*)-one (61). The structure was confirmed by nuclear Overhauser effect (NOE) experiment. Yield = 73%, electricity = 8.1 F mol^{-1}; White solid; ^{1}H NMR (500 MHz, $CDCl_3$) δ 8.48 (s, 1H), 8.39 (d, J = 8.0 Hz, 1H), 7.13 (d, J = 8.0 Hz, 1H), 7.04 (s, 1H), 5.61 (s, 1H), 3.73 (s, 3H), 3.37 (s, 6H), 2.69 (s, 3H), 2.45 (s, 3H), 2.43 (s, 3H); ^{13}C NMR (126 MHz, $CDCl_3$) δ 147.3, 146.7, 143.6, 143.0, 138.0, 132.2, 132.0, 128.6, 127.9, 125.6, 124.8, 114.8, 111.5, 111.2, 102.9, 53.3, 30.4, 22.4, 15.0, 14.0; IR (neat, cm^{-1}): 2919, 1703, 1586, 1469, 1377, 1083, 816, 752; ESI HRMS *m/z* $(M+H)^+$ calcd 366.1812, obsd 366.1814.

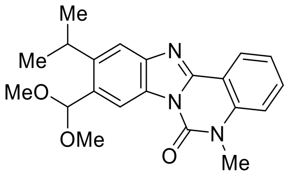

9-(Dimethoxymethyl)-10-isopropyl-5-methylbenzo[4,5]imidazo[1,2-*c*]quinazolin-6(5*H*)-one (62). The structure was confirmed by nuclear Overhauser effect (NOE) experiment. Yield = 51%, electricity = 7.2 F mol^{-1}; White solid; ^{1}H NMR (850 MHz, $CDCl_3$) δ 8.65 (s, 1H), 8.51 (dd, *J* = 7.8, 1.6 Hz, 1H), 7.86 (s, 1H), 7.61 (ddd, *J* = 8.5, 7.2, 1.6 Hz, 1H), 7.37 (t, *J* = 7.5 Hz, 1H), 7.32 (d, *J* = 8.4 Hz, 1H), 5.73 (s, 1H), 3.77 (s, 3H), 3.52 (p, *J* = 7.0 Hz, 1H), 3.36 (s, 6H), 1.33 (d, *J* = 7.0 Hz, 6H); ^{13}C NMR (214 MHz, $CDCl_3$) δ 147.5, 147.1, 145.9, 144.3, 138.1, 132.4, 131.4, 129.1, 125.7, 123.9, 116.4, 114.6, 114.1, 113.5, 101.7, 53.1, 30.6, 28.6, 24.8; IR (neat, cm^{-1}): 2922, 1704, 1593, 1483, 1352, 1047, 742; ESI HRMS *m/z* $(M+Na)^+$ calcd 388.1632, obsd 388.1634.

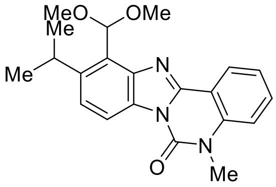

11-(Dimethoxymethyl)-10-isopropyl-5-methylbenzo[4,5]imidazo[1,2-*c*]quinazolin-6(5*H*)-one (62'). Yield = 34%, electricity = 7.2 F mol^{-1}; White solid; ^{1}H NMR (850 MHz, $CDCl_3$) δ 8.63 (dd, *J* = 7.7, 1.6 Hz, 1H), 8.39 (d, *J* = 8.4 Hz, 1H), 7.66 (ddd, *J* = 8.6, 7.2, 1.6 Hz, 1H), 7.48 (d, *J* = 8.4 Hz, 1H), 7.41 (t, *J* = 7.2 Hz, 1H), 7.37 (d, *J* = 8.4 Hz, 1H), 6.49 (s, 1H), 4.03 (h, *J* = 6.9 Hz, 1H), 3.81 (s, 3H), 3.53 (s, 6H), 1.30 (d, *J* = 6.9 Hz, 6H); ^{13}C NMR (214 MHz, $CDCl_3$) δ 147.3, 146.4, 146.2, 143.6, 138.1, 132.2, 128.9, 126.2, 125.7, 123.8, 123.5, 115.6, 114.6, 113.9, 103.6, 56.1, 30.6, 29.1, 24.8; IR (neat, cm^{-1}): 2919, 1699, 1590, 1483, 1359, 1071, 736; ESI HRMS *m/z* $(M+Na)^+$ calcd 388.1632, obsd 388.1633.

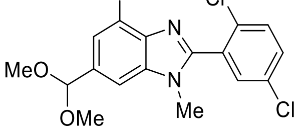

2-(2,5-Dichlorophenyl)-6-(dimethoxymethyl)-1,4-dimethyl-1*H*-benzo[*d*]imidazole (63). The structure was confirmed by nuclear Overhauser effect (NOE) experiment. Yield = 54%, electricity = 11 F mol^{-1}; White solid; ^{1}H NMR (500 MHz, $CDCl_3$) δ 7.60 (dd, *J* = 2.2, 0.7 Hz, 1H), 7.46–7.43 (m, 2H), 7.40 (s, 1H), 7.21 (s, 1H), 5.52 (s, 1H), 3.67 (s, 3H), 3.39 (s, 6H), 2.70 (s, 3H); ^{13}C NMR (126 MHz, $CDCl_3$) δ 150.1, 142.5, 135.4, 133.7, 133.3, 132.9, 132.7, 131.8, 131.5, 130.9, 130.2, 122.0, 105.7, 103.9, 53.1, 31.1, 17.0; IR (neat, cm^{-1}): 2921, 1692, 1450, 1254, 1132, 1097, 877; ESI HRMS *m/z* $(M+Na)^+$ calcd 387.0643, obsd 387.0636.

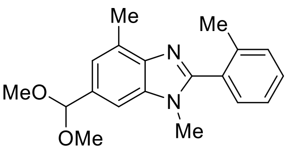

6-(Dimethoxymethyl)-1,4-dimethyl-2-(*o*-tolyl)-1*H*-benzo[*d*]imidazole (64). The structure was confirmed by nuclear Overhauser effect (NOE) experiment. Yield = 53%, electricity = 10 F mol^{-1}; White solid; ^{1}H NMR (850 MHz, $CDCl_3$) δ 7.42–7.36 (m, 3H), 7.33 (d, *J* = 7.6 Hz, 1H), 7.30 (t, *J* = 7.4 Hz, 1H), 7.20 (s, 1H), 5.52 (s, 1H), 3.60 (s, 3H), 3.40 (s, 6H), 2.71 (s, 3H), 2.24 (s, 3H); ^{13}C NMR (214 MHz, $CDCl_3$) δ 153.8, 142.7, 138.3, 135.3, 132.9, 130.5 (2s), 130.3, 130.0, 129.8, 125.9, 121.6, 105.5, 104.1, 53.1, 30.9, 19.9, 17.0; IR (neat, cm^{-1}): 2920, 1670, 1473, 1353, 1262, 1120, 1083, 790; ESI HRMS *m/z* $(M+H)^+$ calcd 311.1760, obsd 311.1762.

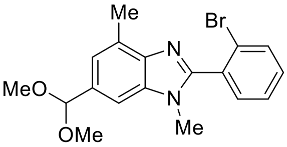

2-(2-Bromophenyl)-6-(dimethoxymethyl)-1,4-dimethyl-1*H*-benzo[*d*]imidazole (65). The structure was confirmed by nuclear Overhauser effect (NOE) experiment. Yield = 51%, electricity = 10 F mol^{-1}; White solid; ^{1}H NMR (500 MHz, $CDCl_3$) δ 7.70 (d, *J* = 7.9 Hz, 1H), 7.54 (dd, *J* = 7.6, 1.8 Hz, 1H), 7.45 (d, *J* = 7.6 Hz, 1H), 7.41–7.35 (m, 2H), 7.21 (s, 1H), 5.52 (s, 1H), 3.64 (s, 3H), 3.39 (s, 6H), 2.71 (s, 3H); ^{13}C NMR (126 MHz, $CDCl_3$) δ 152.6, 142.4, 135.2, 133.3, 132.9, 132.7, 132.5, 131.6, 130.0, 127.7, 124.2, 121.8, 105.7, 104.0, 53.1, 31.1, 17.0; IR (neat, cm^{-1}): 2920, 1687, 1452, 1344, 1263, 1083, 763; ESI HRMS *m/z* $(M+H)^+$ calcd 375.0708, 377.0682, obsd 375.0711, 377.0692.

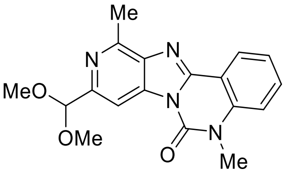

9-(Dimethoxymethyl)-5,11-dimethylpyrido[3',4':4,5]imidazo[1,2-*c*]quinazolin-6(5*H*)-one (66). The structure was confirmed by nuclear Overhauser effect (NOE) experiment. Yield = 62%, electricity = 10 F mol^{-1}; White solid; ^{1}H NMR (500 MHz, $CDCl_3$) δ 8.62 (dd, *J* = 7.9, 1.6 Hz, 1H), 8.46 (s, 1H), 7.72 (ddd, *J* = 8.7, 7.3, 1.6 Hz, 1H), 7.45 (t, *J* = 7.6 Hz, 1H), 7.40 (d, *J* = 8.4 Hz, 1H), 5.57 (s, 1H), 3.82 (s, 3H), 3.45 (s, 6H), 3.02 (s, 3H); ^{13}C NMR (214 MHz, $CDCl_3$) δ 151.3, 151.2, 147.5, 146.9, 139.3, 138.4, 136.5, 133.1, 126.3, 124.3, 114.8, 113.5, 106.3, 104.4, 53.8, 30.8, 20.4; IR (neat, cm^{-1}): 2925, 1703, 1620, 1482, 1372, 1142, 1076, 750; ESI HRMS *m/z* $(M+Na)^+$ calcd 361.1271, obsd 361.1263.

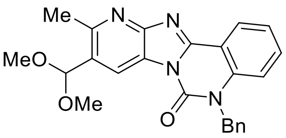

5-Benzyl-9-(dimethoxymethyl)-10-methylpyrido[2',3':4,5]imidazo[1,2-*c*]quinazolin-6(5*H*)-one (67). The structure was confirmed by nuclear Overhauser effect (NOE) experiment. Yield = 80%, electricity = 8.5 F mol^{-1}; White solid; ^{1}H NMR (500 MHz, $CDCl_3$) δ 8.91 (s, 1H), 8.66 (dd, *J* = 7.9, 1.6 Hz, 1H), 7.57 (ddd, *J* = 8.7, 7.3, 1.6 Hz, 1H), 7.40 (t, *J* = 7.5 Hz, 1H), 7.36–7.29 (m, 6H), 5.67 (s, 1H), 5.60 (s, 2H), 3.36 (s, 6H), 2.79 (s, 3H); ^{13}C NMR (214 MHz, $CDCl_3$) δ 156.0, 155.7, 149.2, 147.1, 137.7, 135.4, 133.1, 129.2, 128.0, 127.8, 126.8, 126.6, 124.4, 122.6, 122.2, 115.6, 113.4, 100.8, 52.9, 47.4, 22.9; IR (neat, cm^{-1}): 2921, 1704, 1616, 1541, 1396, 1110, 1083, 752; ESI HRMS *m/z* $(M+H)^+$ calcd 415.1765, obsd 415.1762.

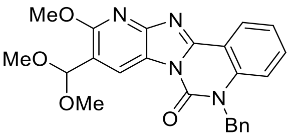

5-Benzyl-9-(dimethoxymethyl)-10-methoxypyrido[2',3':4,5]imidazo[1,2-*c*]quinazolin-6(5*H*)-one (68). The structure was confirmed by nuclear Overhauser effect (NOE) experiment. Yield = 86%, electricity = 8.1 F mol^{-1}; White solid; ^{1}H NMR (500 MHz, $CDCl_3$) δ 8.87 (s, 1H), 8.61 (dd, *J* = 7.8, 1.6 Hz, 1H), 7.54 (ddd, *J* = 8.7, 7.3, 1.6 Hz, 1H), 7.37 (t, *J* = 7.8 Hz, 1H), 7.34–7.27 (m, 6H), 5.74 (s, 1H), 5.59 (s, 2H), 4.16 (s, 3H), 3.39 (s, 6H); ^{13}C NMR (126 MHz, $CDCl_3$) δ 160.9, 154.2, 148.2, 146.9, 137.4, 135.5, 132.8, 129.2, 128.0, 126.7, 126.4, 124.6, 124.3, 118.8, 117.6, 115.6, 113.4, 99.0, 54.6, 53.4, 47.4; IR (neat, cm^{-1}): 2926, 1703, 1615, 1588, 1397, 1213, 1032, 751; ESI HRMS *m/z* $(M+Na)^+$ calcd 453.1533, obsd 453.1524.

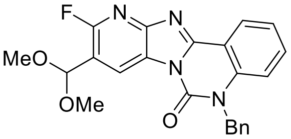

5-Benzyl-9-(dimethoxymethyl)-10-fluoropyrido[2',3':4,5]imidazo[1,2-*c*]quinazolin-6(5*H*)-one (69). The structure was confirmed by nuclear Overhauser effect (NOE) experiment. Yield = 79%, electricity = 15 F mol^{-1}; White solid; ^{1}H NMR (500 MHz, $CDCl_3$) δ 9.03 (d, *J* = 8.4 Hz, 1H), 8.62 (dd, *J* = 7.9, 1.6 Hz, 1H), 7.59 (ddd, *J* = 8.7, 7.3, 1.6 Hz, 1H), 7.40 (t, *J* = 7.4 Hz, 1H), 7.36–7.29 (m, 6H), 5.72 (s, 1H), 5.60 (s, 2H), 3.42 (s, 6H); ^{13}C NMR (214 MHz, $CDCl_3$) δ 159.5 (d, $J_{C\text{-}F}$ = 242.3 Hz), 153.9 (d, $J_{C\text{-}F}$ = 20.3 Hz), 150.2, 146.7, 137.7, 135.2, 133.6, 129.3, 128.2, 126.9, 126.7, 126.5 (d, $J_{C\text{-}F}$ = 5.9 Hz), 124.6, 122.2 (d, $J_{C\text{-}F}$ = 3.1 Hz), 116.5 (d, $J_{C\text{-}F}$ = 29.8 Hz), 115.7, 113.0, 98.5 (d, $J_{C\text{-}F}$ = 1.6 Hz), 53.7, 47.5; ^{19}F NMR (471 MHz, $CDCl_3$) δ −72.0; IR (neat, cm^{-1}): 2924, 1707, 1549, 1401, 1336, 1122, 750; ESI HRMS *m/z*

$(M+H)^+$ calcd 419.1514, obsd 419.1513.

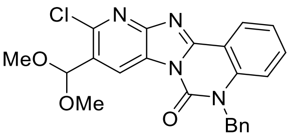

5-Benzyl-10-chloro-9-(dimethoxymethyl)pyrido[2',3':4,5]imidazo[1,2-*c*]quinazolin-6(5*H*)-one (70). The structure was confirmed by nuclear Overhauser effect (NOE) experiment. Yield = 54%, electricity = 20 F mol^{-1}; White solid; ^1H NMR (600 MHz, $CDCl_3$) δ 8.99 (s, 1H), 8.62 (dd, *J* = 7.9, 1.6 Hz, 1H), 7.60 (ddd, *J* = 8.7, 7.2, 1.6 Hz, 1H), 7.41 (t, *J* = 7.4 Hz, 1H), 7.37–7.26 (m, 6H), 5.78 (s, 1H), 5.60 (s, 2H), 3.43 (s, 6H); ^{13}C NMR (151 MHz, $CDCl_3$) δ 155.7, 150.5, 147.9, 146.8, 137.9, 135.2, 133.8, 129.3, 128.2, 127.8, 127.1, 126.7, 124.7, 124.6, 123.2, 115.7, 113.0, 100.8, 54.0, 47.5; IR (neat, cm^{-1}): 2926, 1703, 1615, 1481, 1400, 1289, 1076, 755; ESI HRMS *m/z* $(M+Na)^+$ calcd 457.1038, obsd 457.1028.

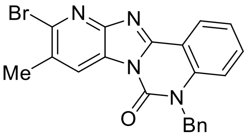

5-Benzyl-10-bromo-9-methylpyrido[2',3':4,5]imidazo[1,2-*c*]quinazolin-6(5*H*)-one (71). The structure was confirmed by nuclear Overhauser effect (NOE) experiment. Yield = 66%, electricity = 8.2 F mol^{-1}; White solid; ^1H NMR (500 MHz, $CDCl_3$) δ 8.60 (d, *J* = 7.8 Hz, 1H), 8.58 (s, 1H), 7.58 (t, *J* = 7.8 Hz, 1H), 7.39 (t, *J* = 7.6 Hz, 1H), 7.37–7.28 (m, 6H), 5.59 (s, 2H), 2.59 (s, 3H); ^{13}C NMR (214 MHz, $CDCl_3$) δ 154.5, 149.1, 147.0, 142.0, 137.7, 135.2, 133.4, 130.5, 129.3, 128.2, 126.9, 126.7, 125.3, 124.6, 123.7, 115.7, 113.2, 47.5, 23.0; IR (neat, cm^{-1}): 2922, 1704, 1614, 1481, 1398, 1196, 1131, 1076, 750; ESI HRMS *m/z* $(M+H)^+$ calcd 419.0502, 421.0482 obsd 419.0500, 421.0481.

Synthesis of Telmisartan

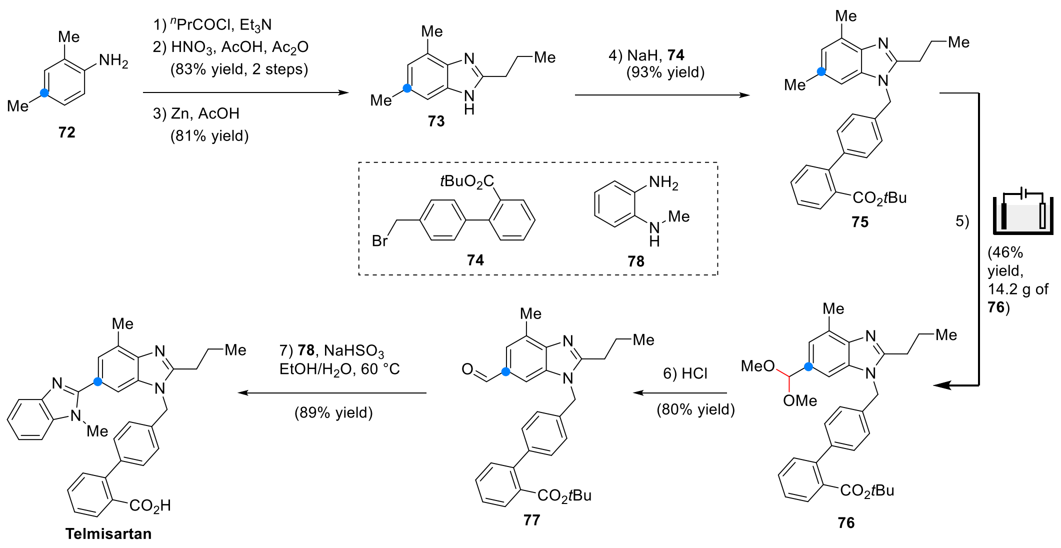

4,6-Dimethyl-2-propyl-1*H*-benzo[*d*]imidazole (73). The title compound was prepared as a white solid (15.0 g, 80.0 mmol) for three steps starting from 2,4-dimethylaniline (14.8 mL, 120 mmol) by following the procedure described for the synthesis of **S32**.

***tert*-Butyl-4'-((4,6-dimethyl-2-propyl-1*H*-benzo[*d*]imidazol-1-yl)methyl)-[1,1'-biphenyl]-2-carboxylate (75)**. The title compound was prepared from **73** (13 g, 71 mmol, 1.0 equiv) and **74** (30 g, 85 mmol, 1.2 equiv) as a white solid in 93% yield (30 g) by following the General Procedure A (step 2). The structure was confirmed by nuclear Overhauser effect (NOE) experiment. ^{1}H NMR (850 MHz, $CDCl_3$) δ 7.76 (dd, *J* = 7.7, 1.4 Hz, 1H), 7.44 (td, *J* = 7.5, 1.5 Hz, 1H), 7.36 (td, *J* = 7.6, 1.3 Hz, 1H), 7.27–7.22 (m, 3H), 7.04 (d, *J* = 8.1 Hz, 2H), 6.87 (s, 1H), 6.82 (s, 1H), 5.33 (s, 2H), 2.87–2.83 (m, 2H), 2.65 (s, 3H), 2.37 (s, 3H), 1.84–1.79 (m, 2H), 1.21 (s, 9H), 1.01 (t, *J* = 7.4 Hz, 3H); ^{13}C NMR (214 MHz, $CDCl_3$) δ 168.0, 154.2, 141.6, 141.4, 140.3, 135.3 (2s), 133.0, 132.0, 130.8, 130.6, 129.8, 129.3, 128.8, 127.4, 125.7, 124.3, 107.2, 81.4, 46.8, 30.0, 27.7, 22.2, 21.9, 16.9, 14.2; IR (neat, cm^{-1}): 2929, 1708, 1446, 1302, 1129, 1047, 848, 762; ESI HRMS *m/z* $(M+Na)^+$ calcd 477.2512, obsd 477.2509.

***tert*-Butyl-4'-((6-(dimethoxymethyl)-4-methyl-2-propyl-1*H*-benzo[*d*]imidazol-1-yl)methyl)-[1,1'-biphenyl]-2-carboxylate (76)**. ^{1}H NMR (500 MHz, $CDCl_3$) δ 7.76 (d, *J* = 7.6 Hz, 1H), 7.46 (t, *J* = 7.5 Hz, 1H), 7.37 (t, *J* = 7.6 Hz, 1H), 7.30–7.20 (m, 4H), 7.14 (s, 1H), 7.04 (d, *J* = 7.9 Hz, 2H), 5.43 (s, 1H), 5.40 (s, 2H), 3.31 (s, 6H), 2.88–2.83 (m, 2H), 2.70 (s, 3H), 1.83 (h, *J* = 7.5 Hz, 2H), 1.23 (s, 9H), 1.02 (t, *J* = 7.3 Hz, 3H); ^{13}C NMR (126 MHz, $CDCl_3$) δ 167.9, 155.4, 142.3, 141.6, 141.4, 135.1 (2s), 132.9, 132.5, 130.8, 130.6, 129.8, 129.3, 129.1, 127.4, 125.7, 121.4, 105.5, 103.9, 81.4, 52.9, 46.8, 30.0, 27.7, 22.0, 17.0, 14.2; IR (neat, cm^{-1}): 2921, 1709, 1599, 1517, 1367, 1302, 1100, 1005, 848, 757, 734; ESI HRMS *m/z* $(M+Na)^+$ calcd 537.2724, obsd 537.2718.

4'-((6-Formyl-4-methyl-2-propyl-1*H*-benzo[*d*]imidazol-1-yl)methyl)-[1,1'-biphenyl]-2-carboxylic acid (77). To a solution of **76** (2.0 g, 3.8 mmol, 1.0 equiv) in 1,4-dioxane (30 mL) was added concentrated hydrochloric acid (10 mL). The resulting reaction mixture was heated to reflux for 3 h. 1,4-Dioxane was then evaporated under reduced pressure. Water (30 mL) was added and acidified using AcOH. The resulting solution was extracted with CH_2Cl_2 (5 x 20 mL). The combined organic solution was dried over anhydrous Na_2SO_4, filtered, and concentrated under reduced pressure to afford **77** as a white solid in 80% yield (1.4 g). The crude product was used in the next step without further purification.

Telmisartan. To a solution of **S78** (0.037 g, 0.30 mmol, 1.0 equiv) in EtOH (6 mL) and H_2O (2 mL) was added $NaHSO_3$ (0.078 g, 0.75 mmol, 2.5 equiv) and **77** (0.17 g, 0.36 mmol, 1.2 equiv). The reaction was stirred at 60 °C for 8 h and then cooled to RT. The solvent was

removed under reduced pressure and the residue was chromatographed through silica gel eluting with MeOH/CH_2Cl_2 to give **telmisartan** as a white solid in 89% yield (0.14 g). ^{1}H NMR (600 MHz, $CDCl_3$) δ 12.59 (brs, 1H), 8.33 (d, *J* = 8.0 Hz, 1H), 8.02 (d, *J* = 7.7 Hz, 1H), 7.49 (t, *J* = 7.4 Hz, 1H), 7.43 (t, *J* = 7.5 Hz, 1H), 7.37 (d, *J* = 7.5 Hz, 1H), 7.35–7.31 (m, 3H), 7.31–7.27 (m, 2H), 7.14 (d, *J* = 7.7 Hz, 2H), 7.05 (s, 1H), 6.99 (s, 1H), 5.36 (s, 2H), 3.68 (s, 3H), 3.08 (t, *J* = 7.9 Hz, 2H), 2.68 (s, 3H), 1.95 (q, *J* = 7.6 Hz, 2H), 1.12 (t, *J* = 7.3 Hz, 3H); ^{13}C NMR (151 MHz, $CDCl_3$) δ 171.3, 156.6, 154.0, 143.4, 142.8, 141.8, 141.1, 135.7, 134.6, 134.0, 133.9, 130.6, 130.3, 129.4, 129.1, 129.0, 127.5, 127.1, 123.8, 123.3, 122.0, 119.7, 111.2, 109.6, 48.7, 31.9, 30.0, 22.5, 17.1, 14.2; IR (neat, cm^{-1}): 2930, 1722, 1681, 1597, 1452, 1248, 1131, 1006, 743; ESI HRMS *m/z* $(M+H)^+$ calcd 515.2442, obsd 515.2446. The spectra data matched the reported literature[24].

Bromination of benzoxazole 81

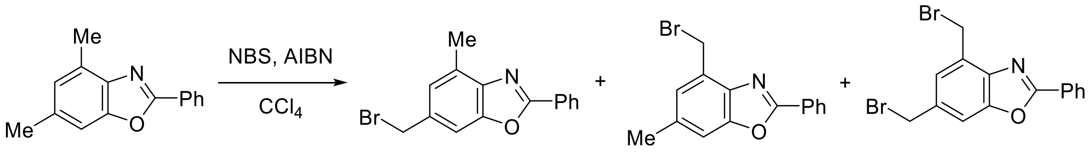

81 **82** **83** **84**

A solution of **81** (0.022 g, 0.10 mmol, 1.0 equiv), NBS (0.020 g, 0.11 mmol, 1.1 equiv) and AIBN (0.0016 g, 0.010 mmol, 0.1 equiv) in CCl_4 (2.0 mL) was heated to reflux for 6 h. The reaction mixture was then cooled to RT and concentrated under reduced pressure. The residue was chromatographed through silica gel eluting with ethyl acetate/hexanes to give the product. The ratio of **82**:**83**:**84** = 1:3:0.8 was determined by ^{1}H NMR analysis of crude reaction mixture.

6-(Bromomethyl)-4-methyl-2-phenylbenzo[*d*]oxazole (82). The structure was confirmed by nuclear Overhauser effect (NOE) experiment. Yield = 50% (15 mg); White solid; ^{1}H NMR (850 MHz, $CDCl_3$) δ 8.27–8.23 (m, 2H), 7.55–7.50 (m, 3H), 7.45 (s, 1H), 7.20 (s, 1H), 4.62 (s, 2H), 2.66 (s, 3H); ^{13}C NMR (214 MHz, $CDCl_3$) δ 163.4, 150.6, 141.9, 134.8, 131.7, 131.0, 129.1, 127.8, 127.3, 126.6, 108.8, 34.2, 16.7; IR (neat, cm^{-1}): 2917, 1614, 1553, 1470, 1447, 1269, 1133, 1077, 763, 703; ESI HRMS m/z $(M+Na)^+$ calcd 323.9994, 325.9974, obsd 323.9996, 325.9975.

4-(Bromomethyl)-6-methyl-2-phenylbenzo[*d*]oxazole (83). The structure was confirmed by nuclear Overhauser effect (NOE) experiment. Yield = 17% (5.0 mg); White solid; ^{1}H NMR (850 MHz, $CDCl_3$) δ 8.27–8.24 (m, 2H), 7.54–7.50 (m, 3H), 7.33 (s, 1H), 7.23 (s, 1H), 4.91 (s, 2H), 2.50 (s, 3H); ^{13}C NMR (214 MHz, $CDCl_3$) δ 162.9, 151.3, 139.0, 135.8, 131.6, 129.1, 129.0, 127.9, 127.4, 126.7, 111.2, 28.1, 22.0; IR (neat, cm^{-1}): 2919, 1632, 1469, 1267, 1196,

1047, 773, 703; ESI HRMS m/z $(M+Na)^+$ calcd 323.9994, 325.9974, obsd 323.9996, 325.9977.

4,6-Bis(bromomethyl)-2-phenylbenzo[*d*]oxazole (84). Yield = 10% (3.8 mg); White solid; ^{1}H NMR (850 MHz, $CDCl_3$) δ 8.30–8.26 (m, 2H), 7.58–7.52 (m, 4H), 7.45 (d, *J* = 1.5 Hz, 1H), 4.91 (s, 2H), 4.63 (s, 2H); ^{13}C NMR (214 MHz, $CDCl_3$) δ 164.3, 151.0, 141.3, 135.4, 132.1, 130.0, 129.1, 128.1, 127.0, 126.7, 111.5, 33.5, 27.4; IR (neat, cm^{-1}): 2920, 1617, 1555, 1449, 1269, 1209, 1051, 1023, 704, 688; ESI HRMS *m/z* $(M+Na)^+$ calcd 401.9100, 403.9079, obsd 401.9104, 403.9083.

DFT calculations

Calculations were performed using the GAUSSIAN 09 program package[25]. The B3LYP functional[26-28] and the aug-cc-Pvdz[29] basis sets were employed. The CPCM(UAHF)[30, 31] implicit solvation model was used to account for the solvation effect (solvent CH_3OH used). All the calculations for the radical intermediates are spin polarized. Optimized structures were checked by calculating vibrational frequencies to ensure the absence of imaginary frequencies. NPA charges were analyzed with the Multiwfn code[32].

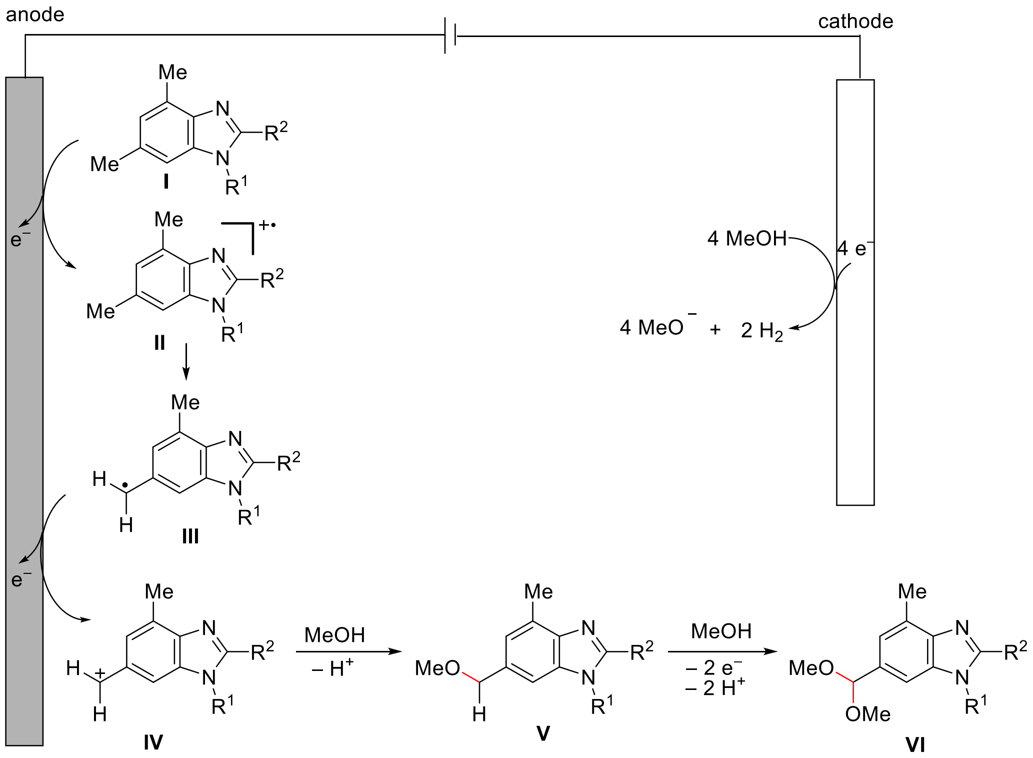

Supplementary Figure 2. Proposed mechanism. The methylarene is oxidized directly on the anode to generate a radical cation **II**, which is deprotonated and further oxidized to give a cation **IV**. The trapping of **IV** by MeOH affords monomethoxylated **V**, which undergoes a second oxidation to afford the final product **VI**. While the oxidation potentials of **V** and **I** are close, **VI** is oxidized at higher potential than **V** and **I**, preventing its further oxidation to an orthoester.

NMR Spectra

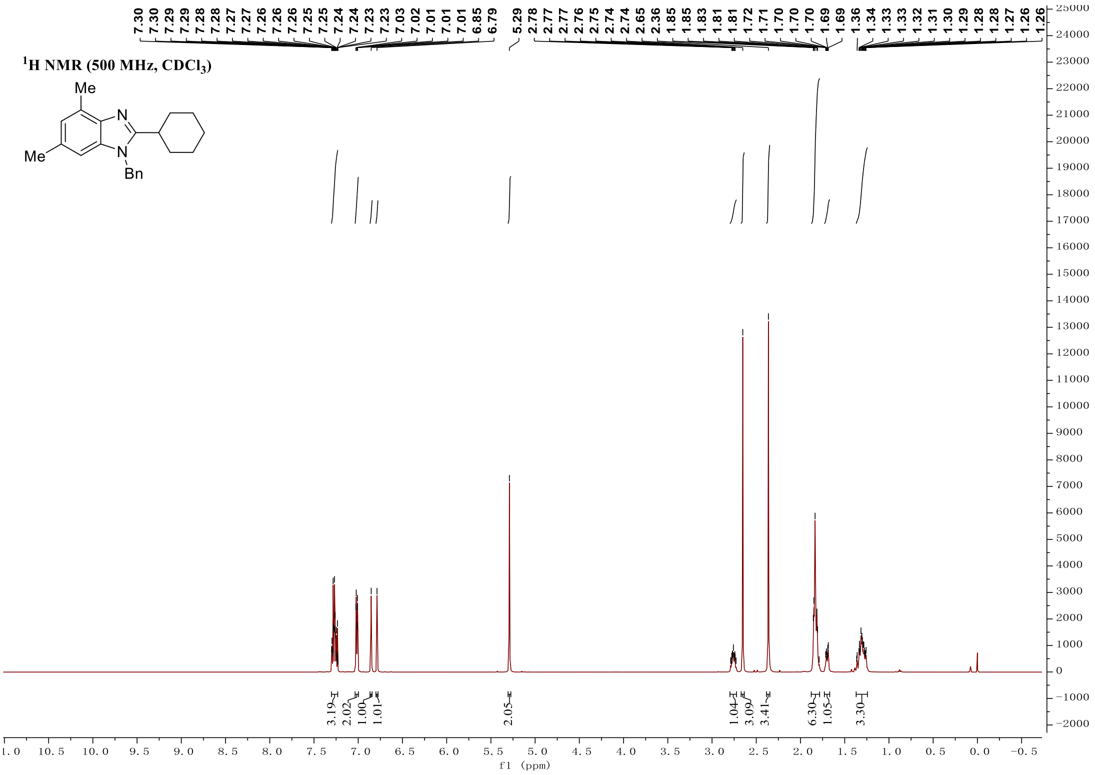

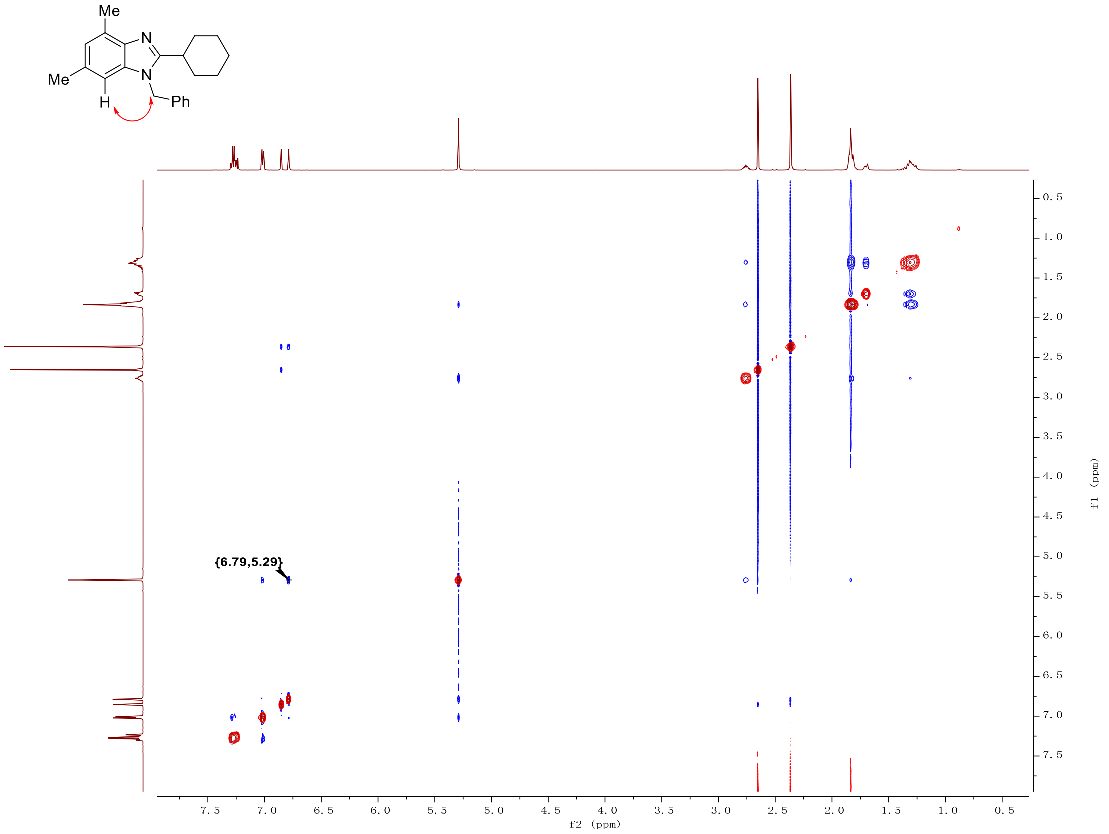

Supplementary Figure 3. ^{1}**H NMR and 2D NOESY spectra of compound S4.**

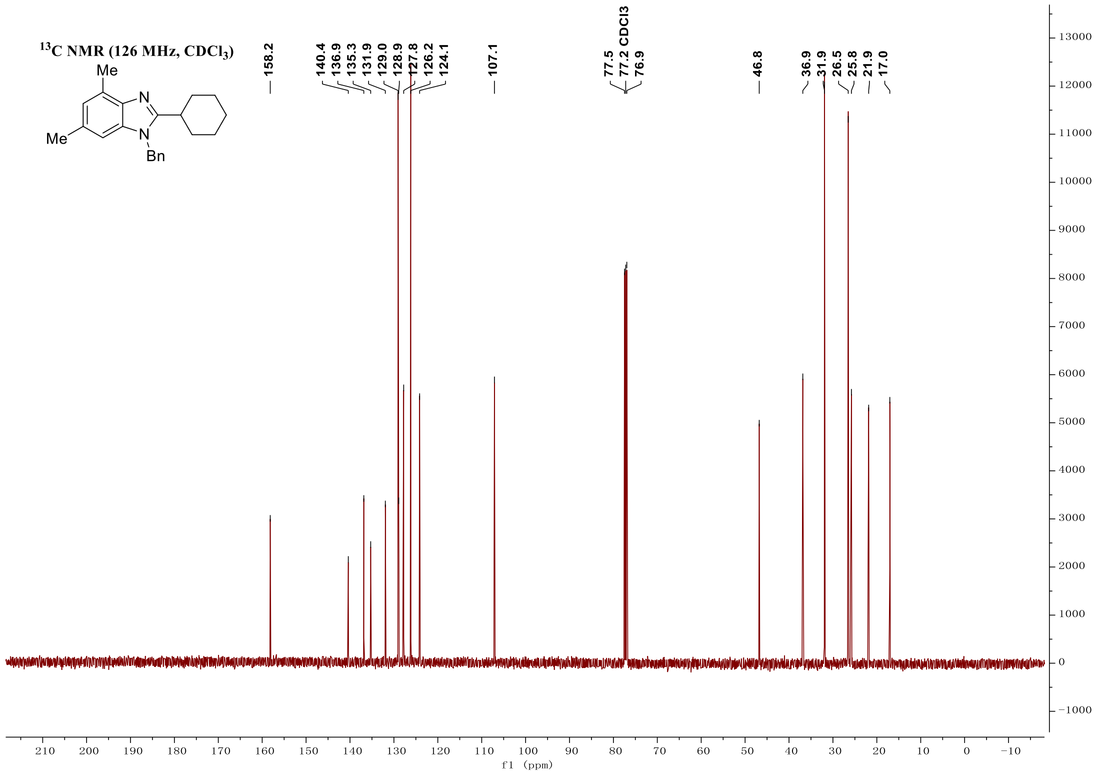

Supplementary Figure 4. ^{13}C NMR spectrum of compound S4.

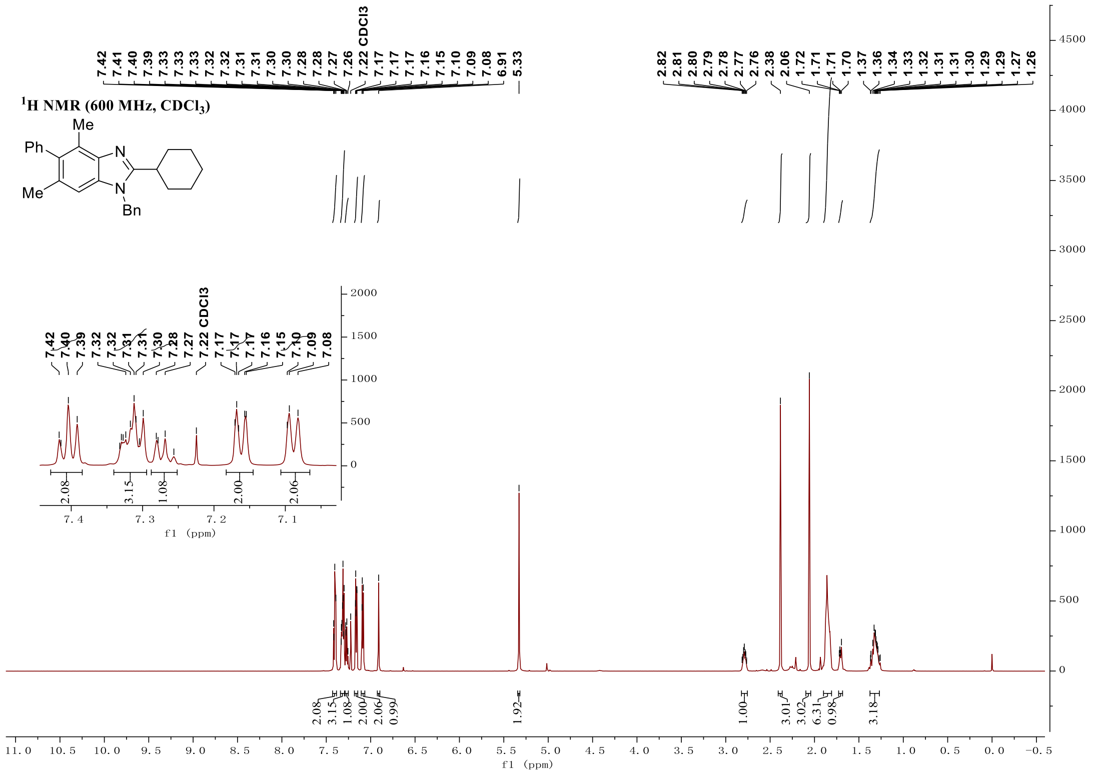

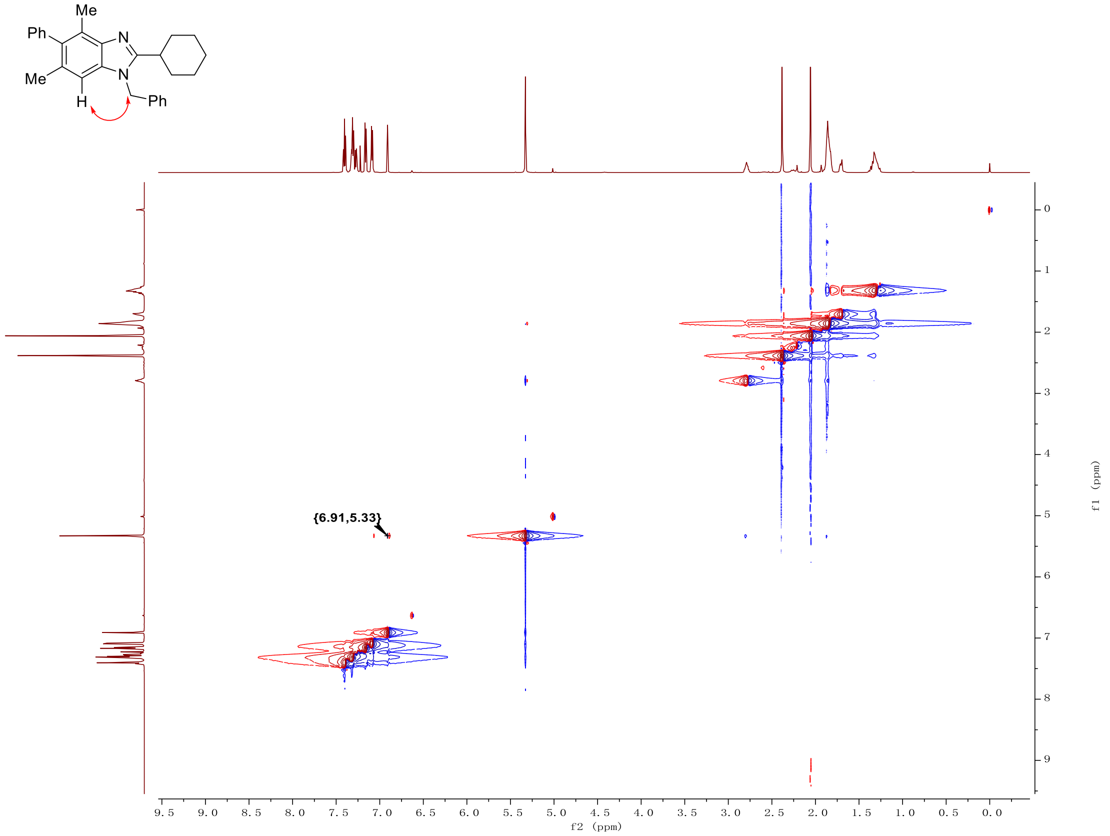

Supplementary Figure 5. ^{1}H NMR and 2D NOESY spectra of compound S8.

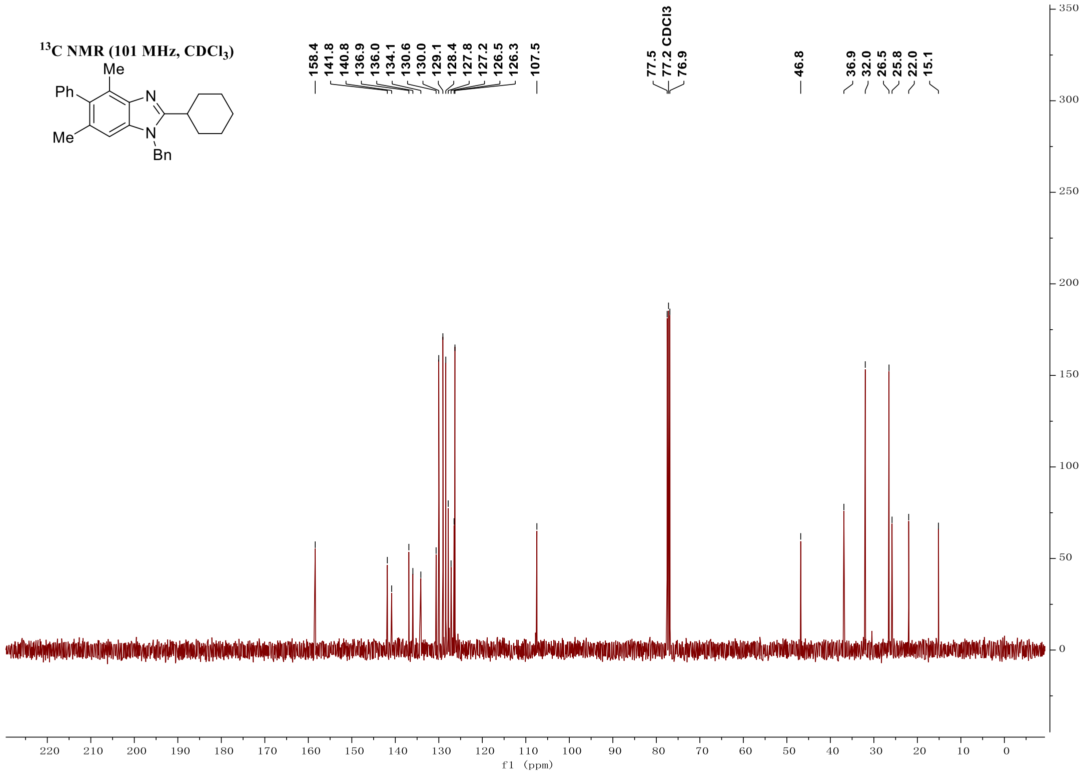

Supplementary Figure 6. ^{13}C NMR spectrum of compound S8.

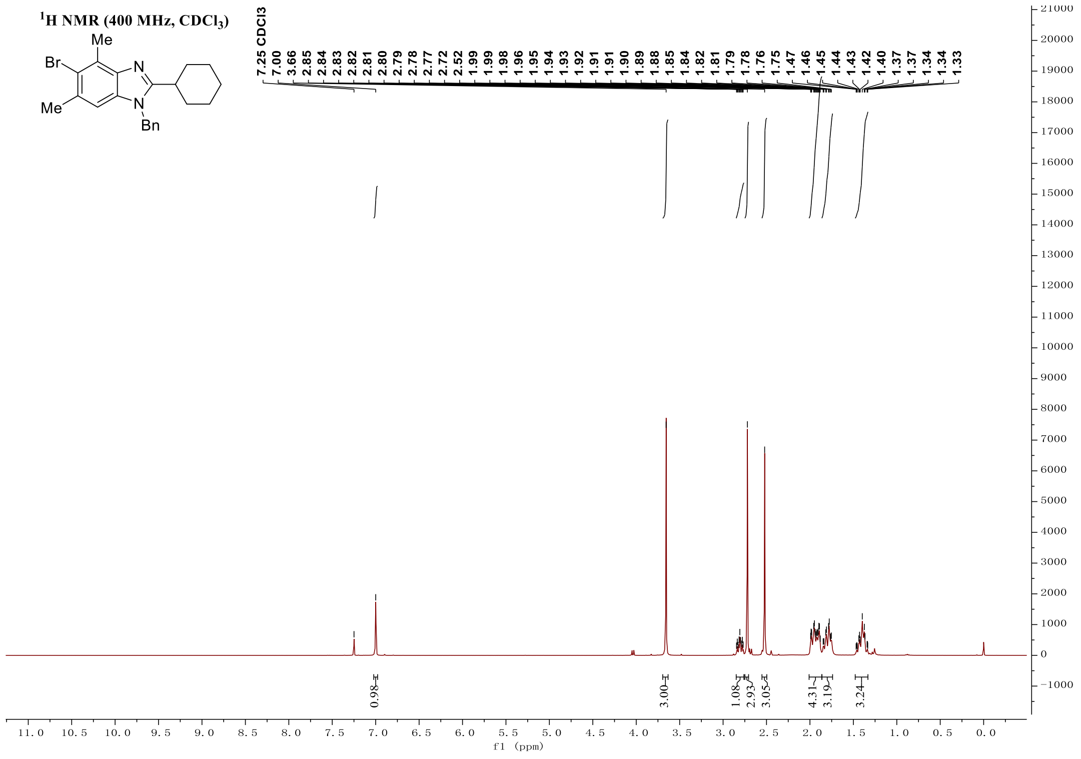

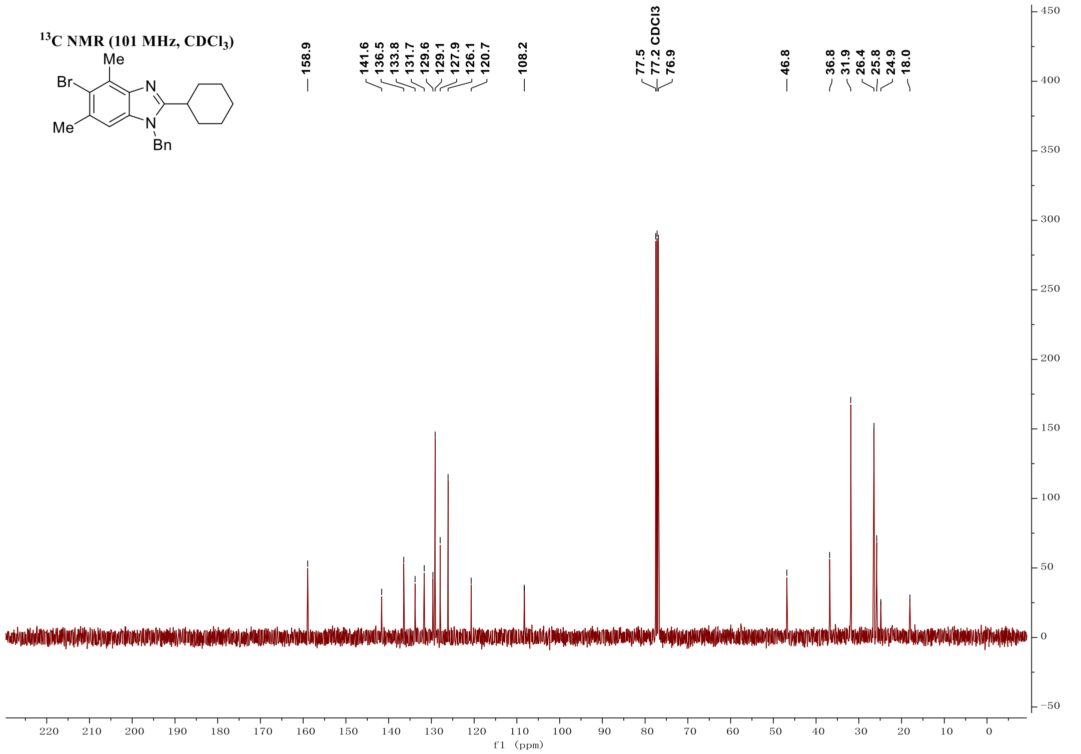

Supplementary Figure 7. ^{1}H NMR and ^{13}C NMR spectra of compound S9.

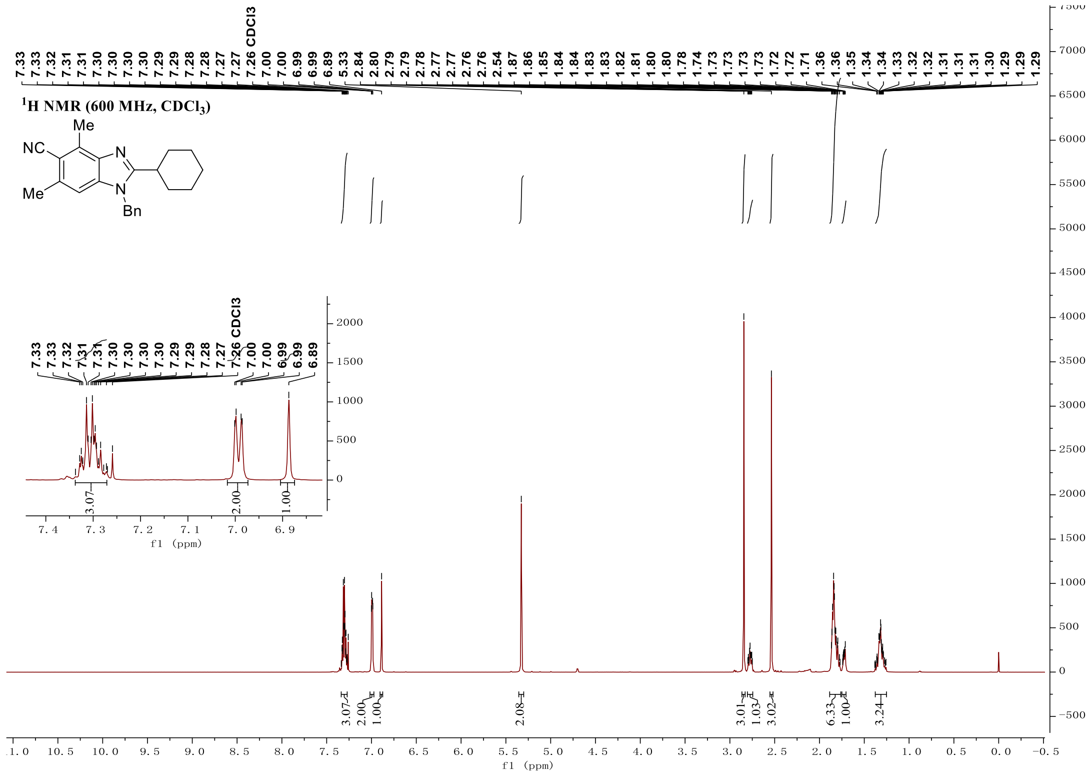

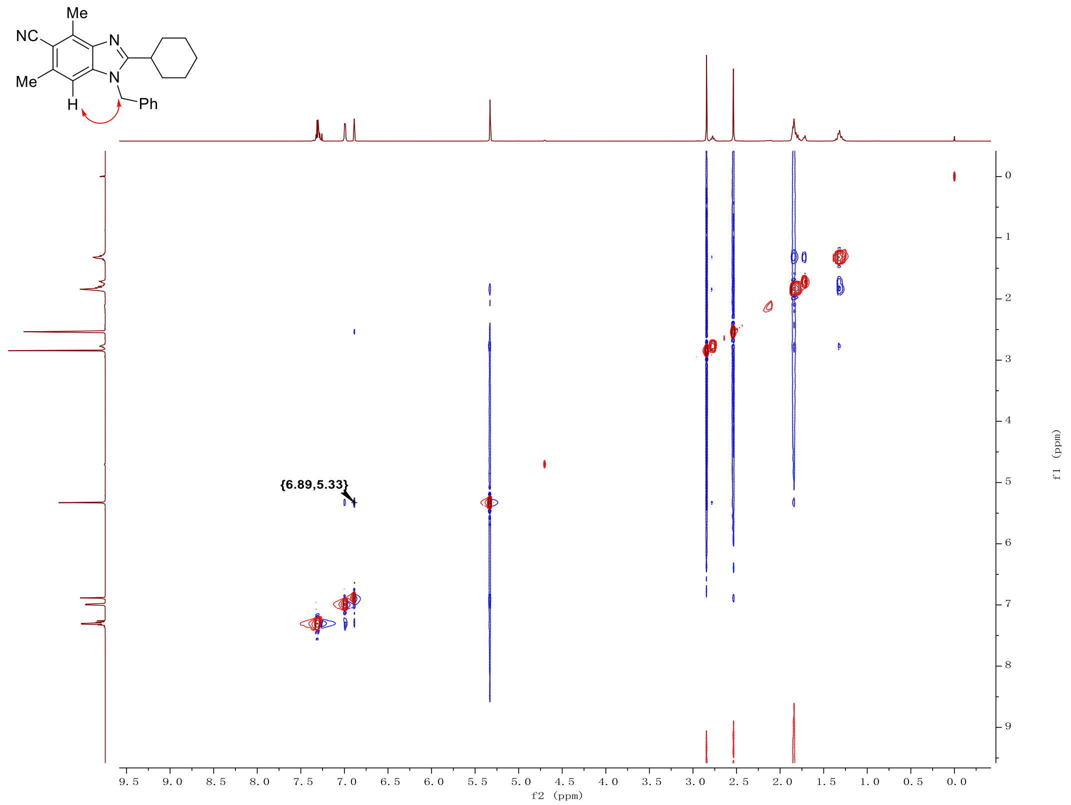

Supplementary Figure 8. ^{1}H NMR and 2D NOESY spectra of compound S14.

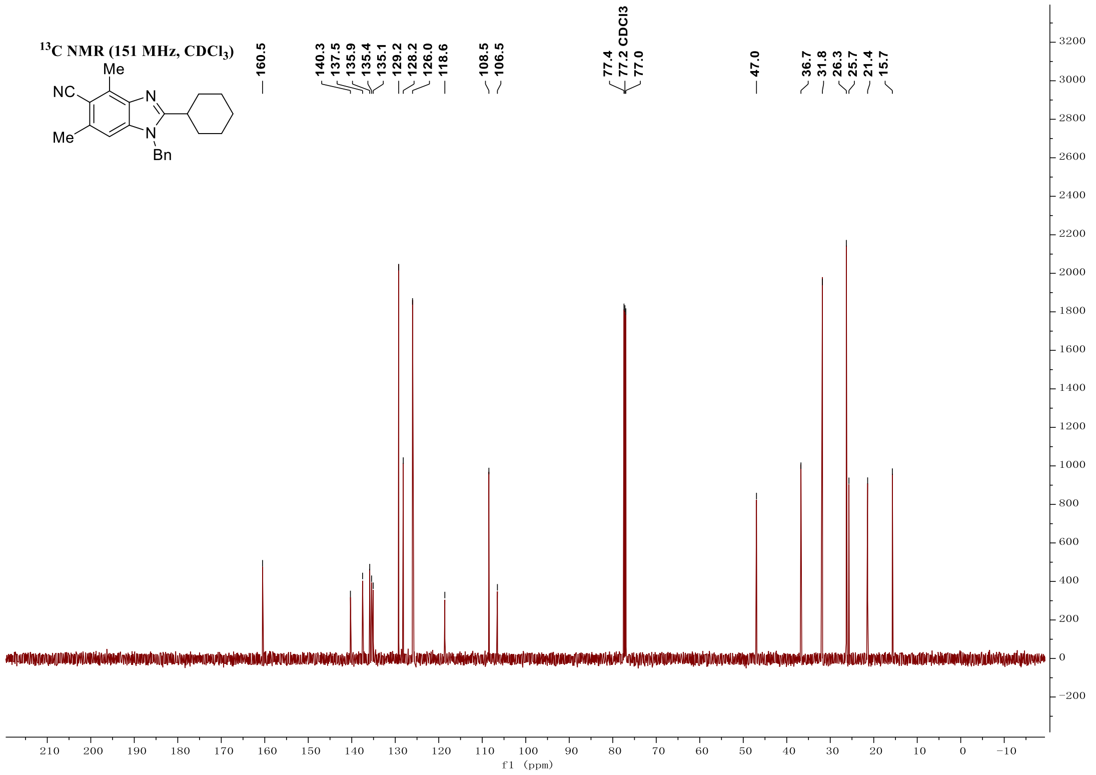

Supplementary Figure 9. ^{13}C NMR spectrum of compound S14.

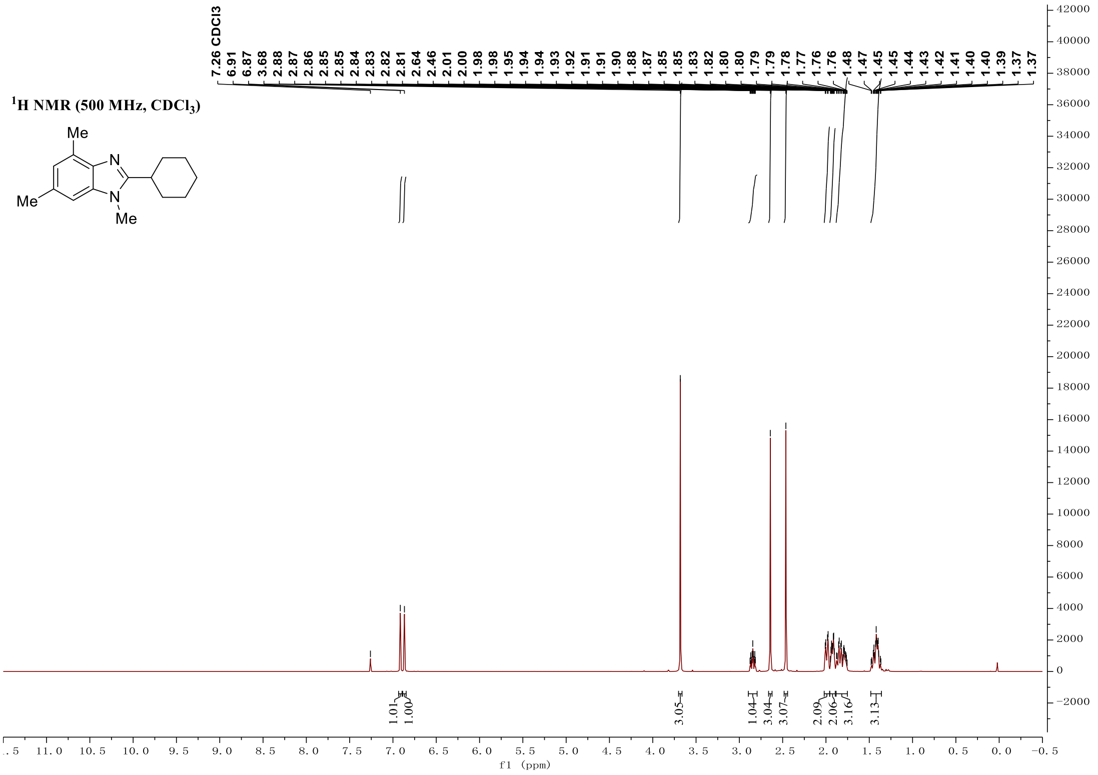

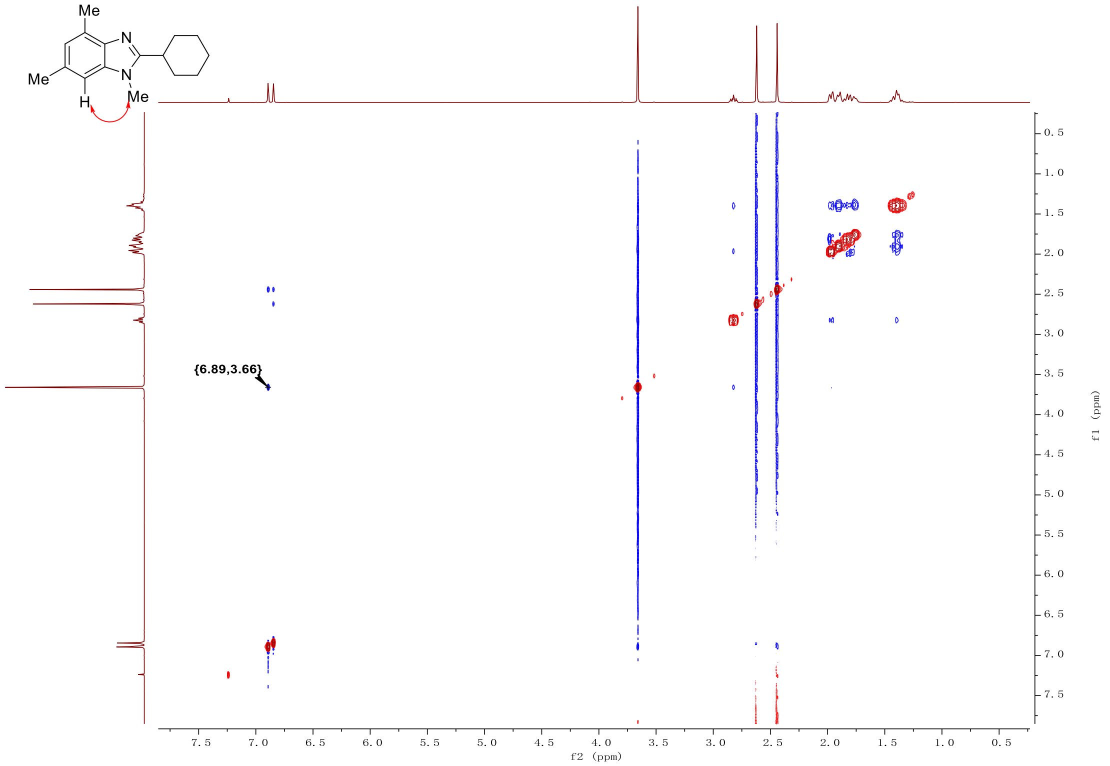

Supplementary Figure 10. ^{1}H NMR and 2D NOESY spectra of compound S15.

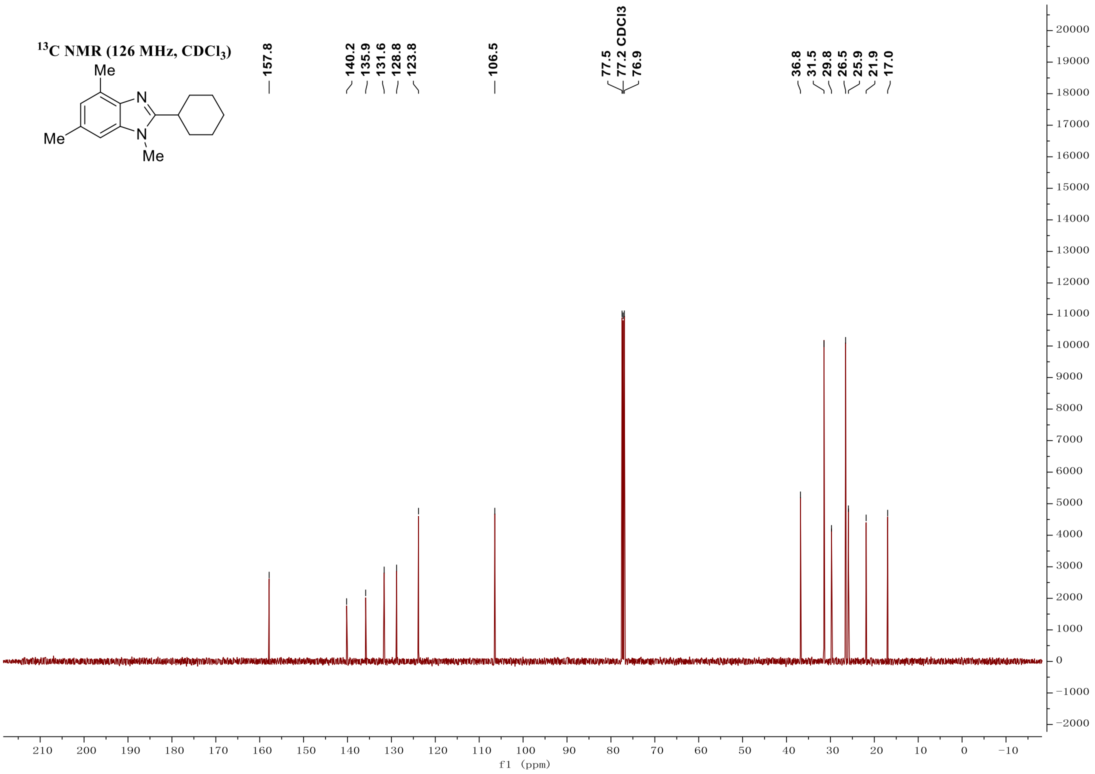

Supplementary Figure 11. ^{13}C NMR spectrum of compound S15.

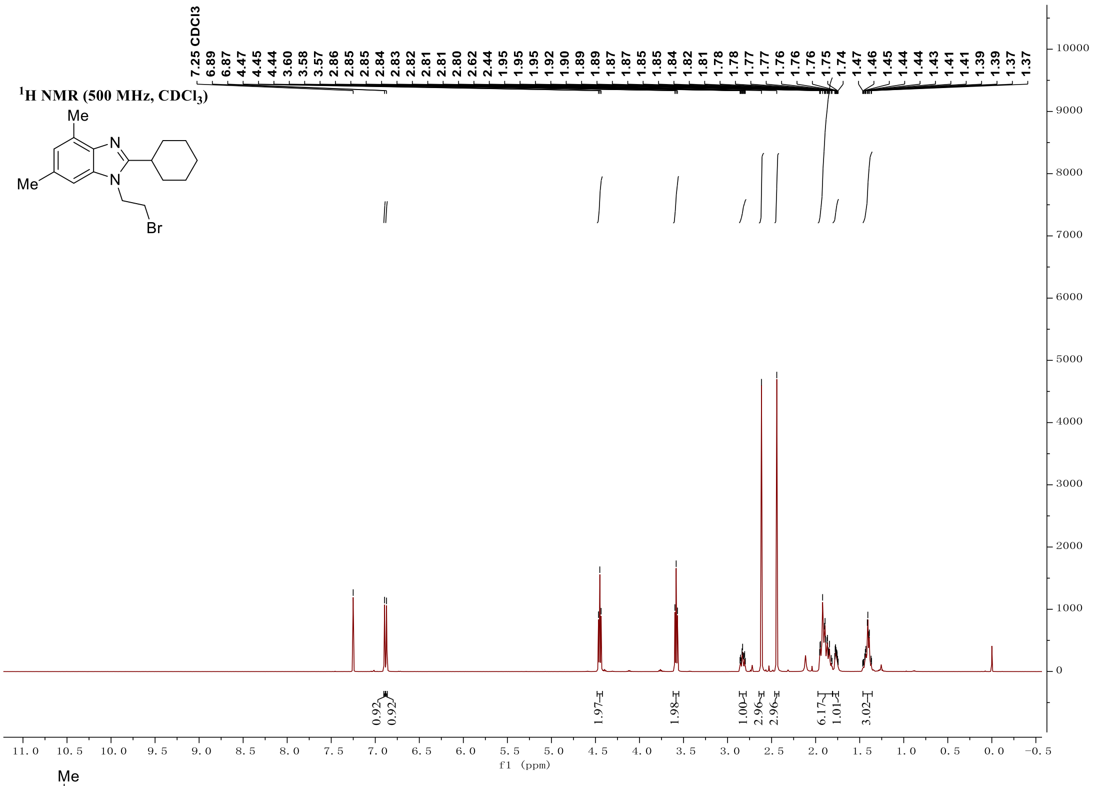

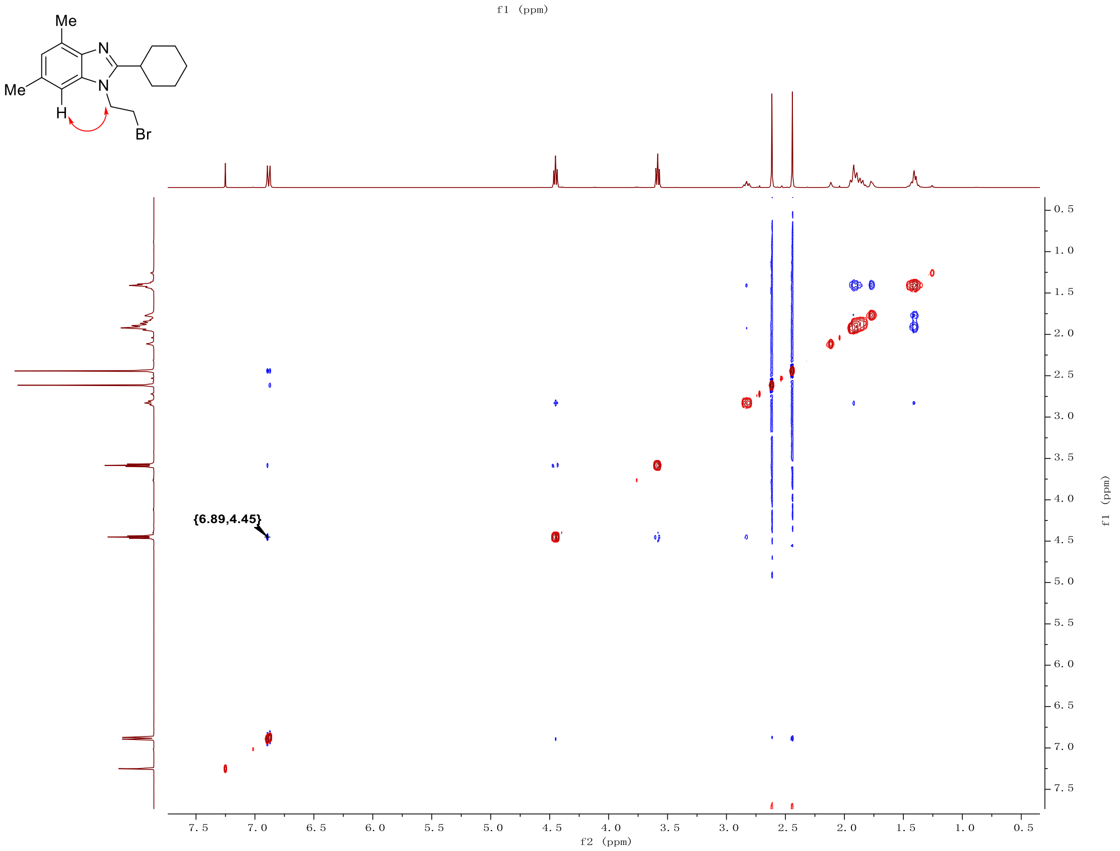

Supplementary Figure 12. ^{1}H NMR and 2D NOESY spectra of compound S18.

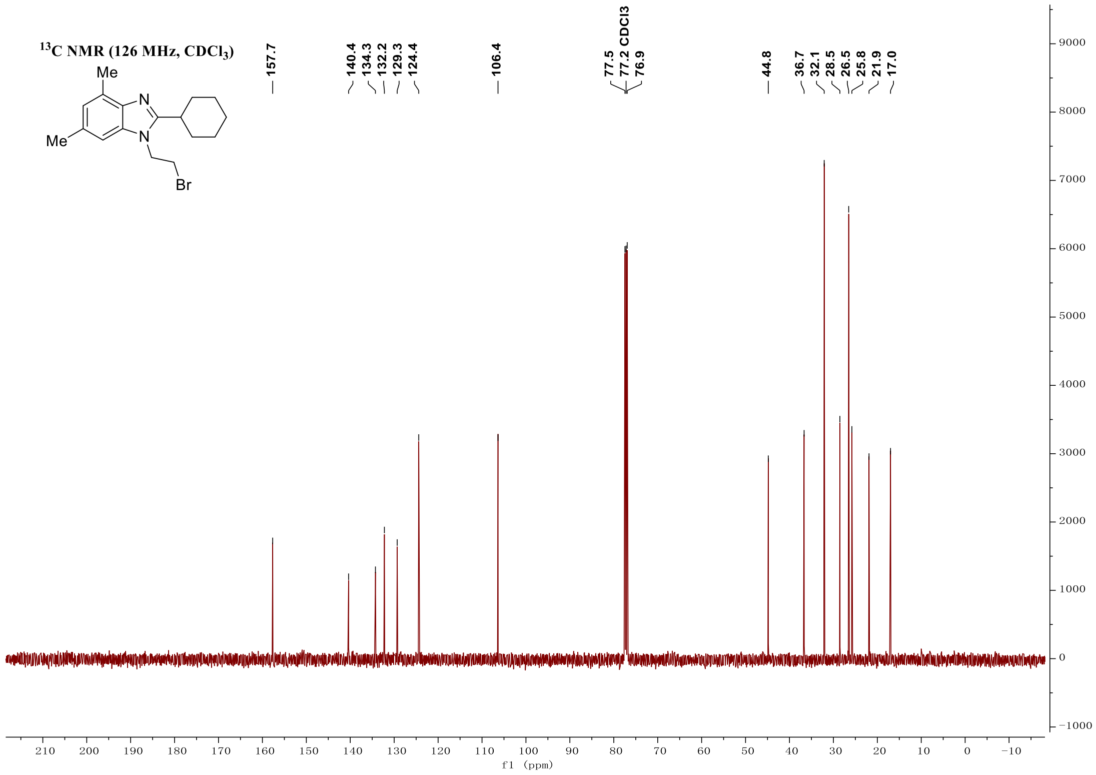

Supplementary Figure 13. ^{13}C NMR spectrum of compound S18.

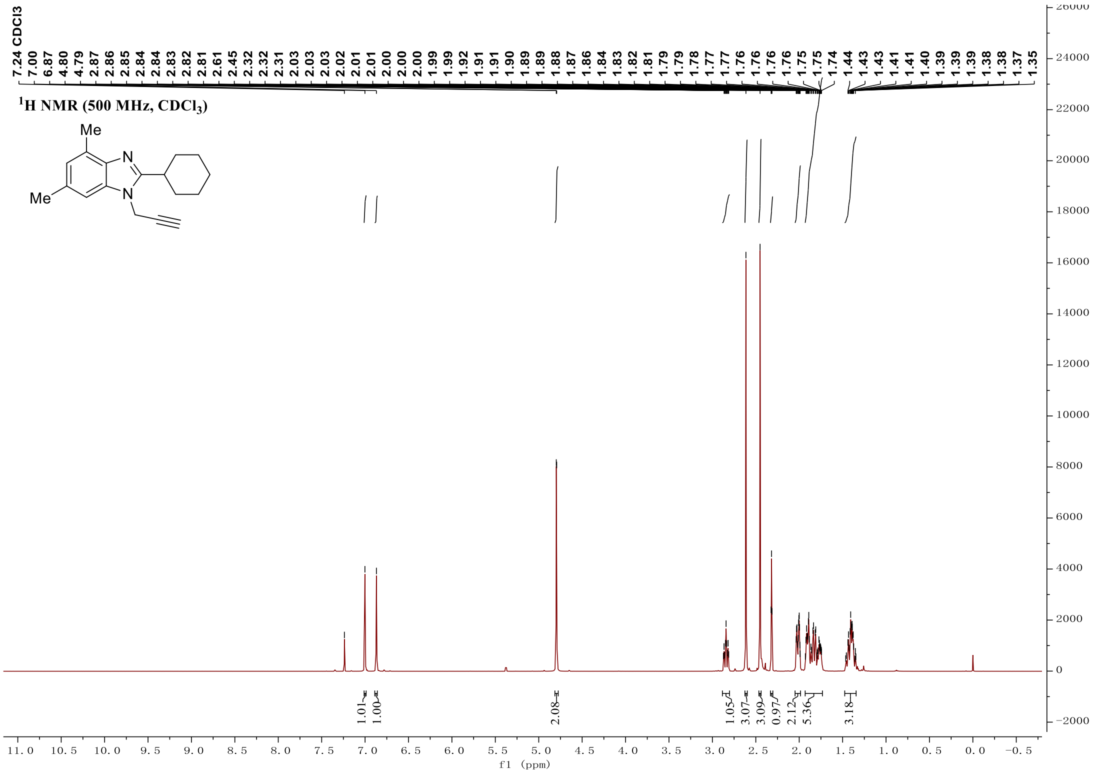

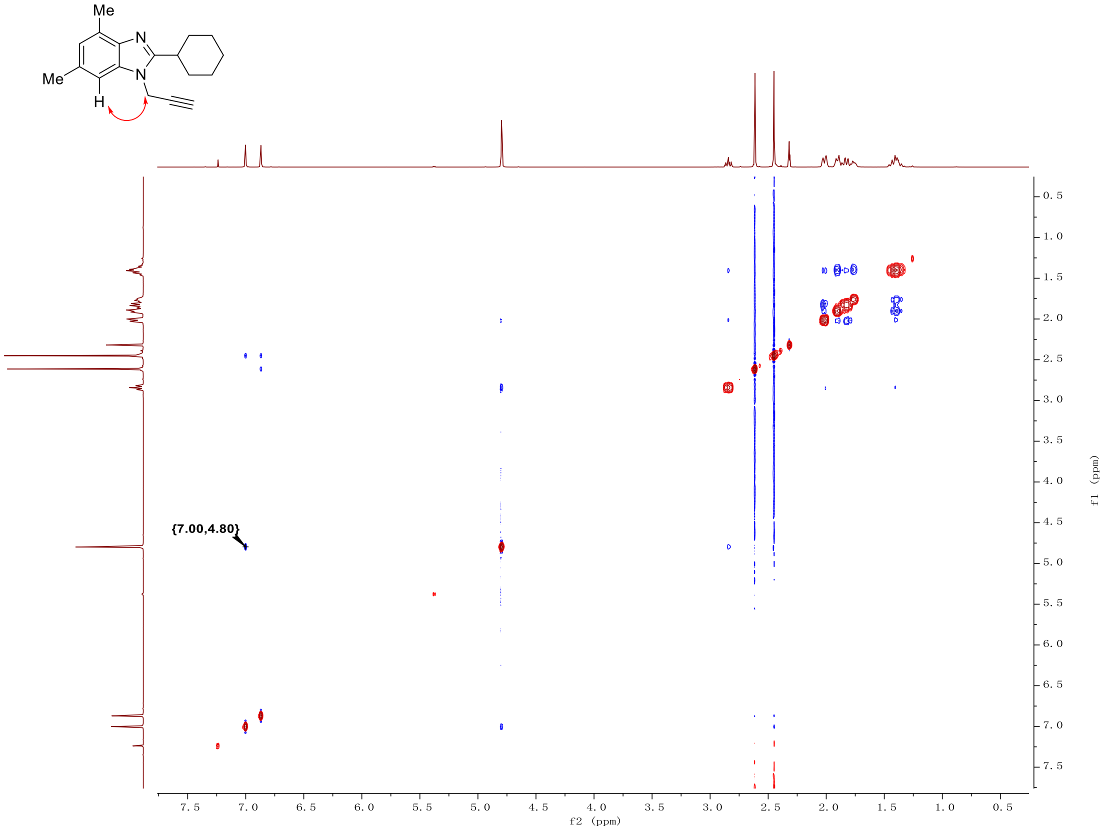

Supplementary Figure 14. ^{1}H NMR and 2D NOESY spectra of compound S19.

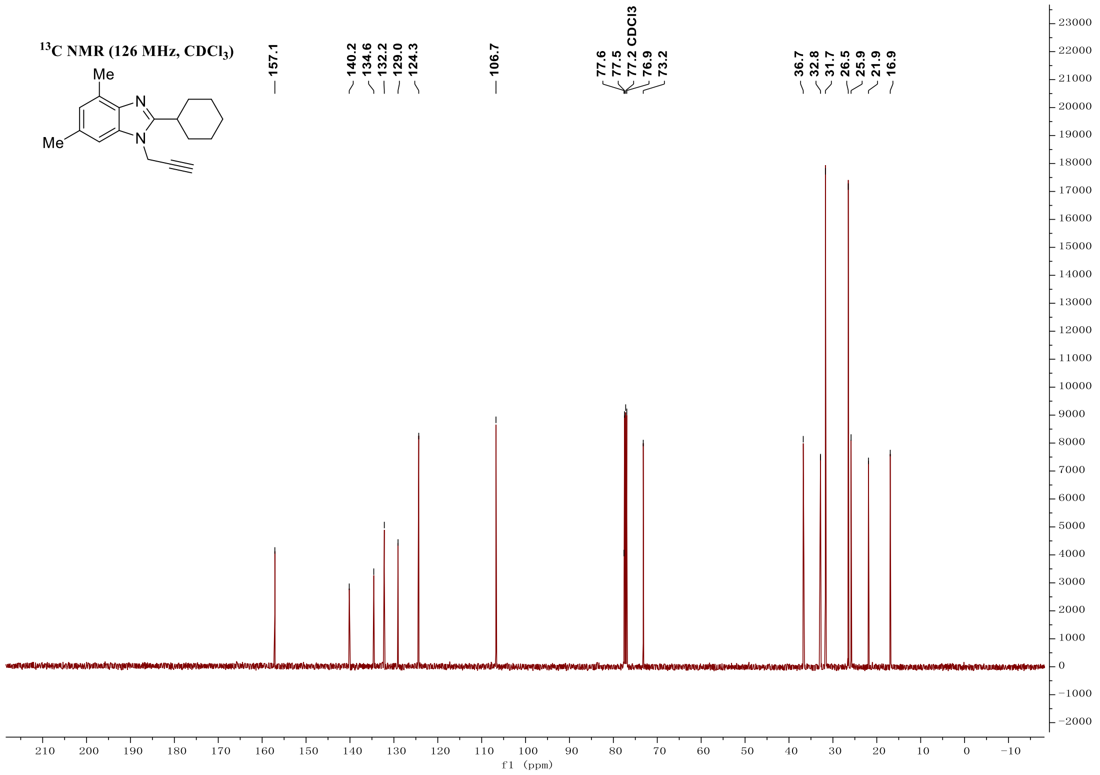

Supplementary Figure 15. ^{13}C NMR spectrum of compound S19.

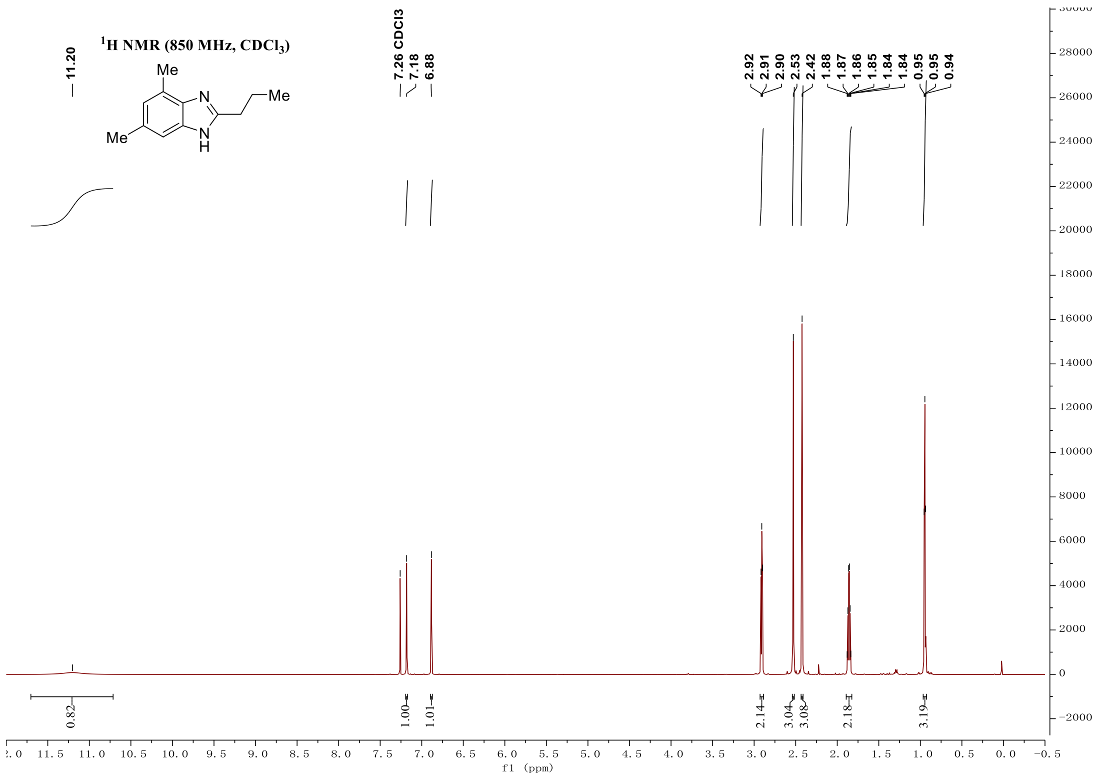

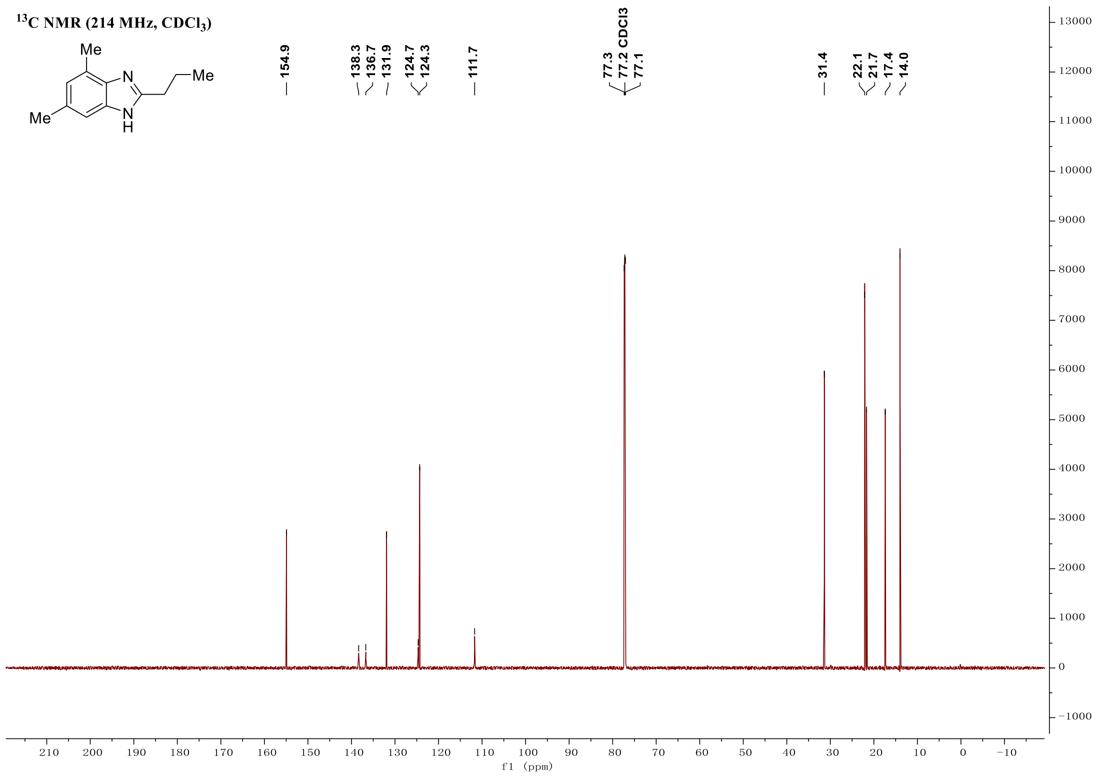

Supplementary Figure 16. ^{1}H **NMR and** ^{13}C **NMR spectra of compound S20.**

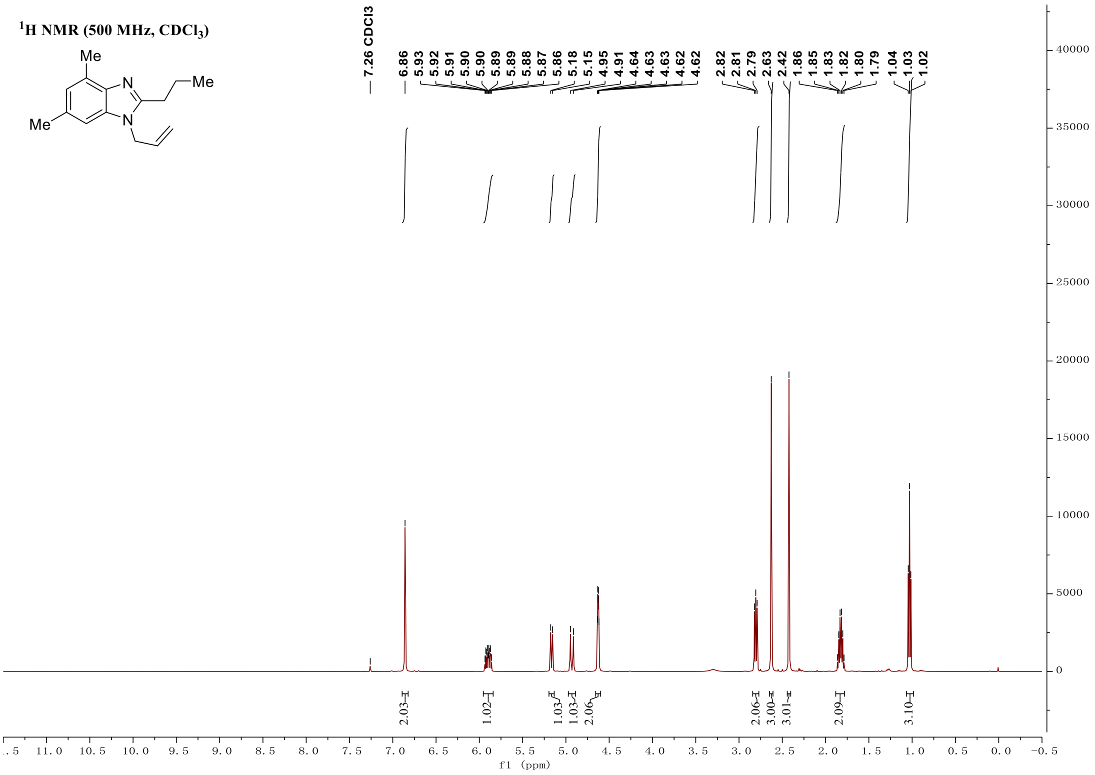

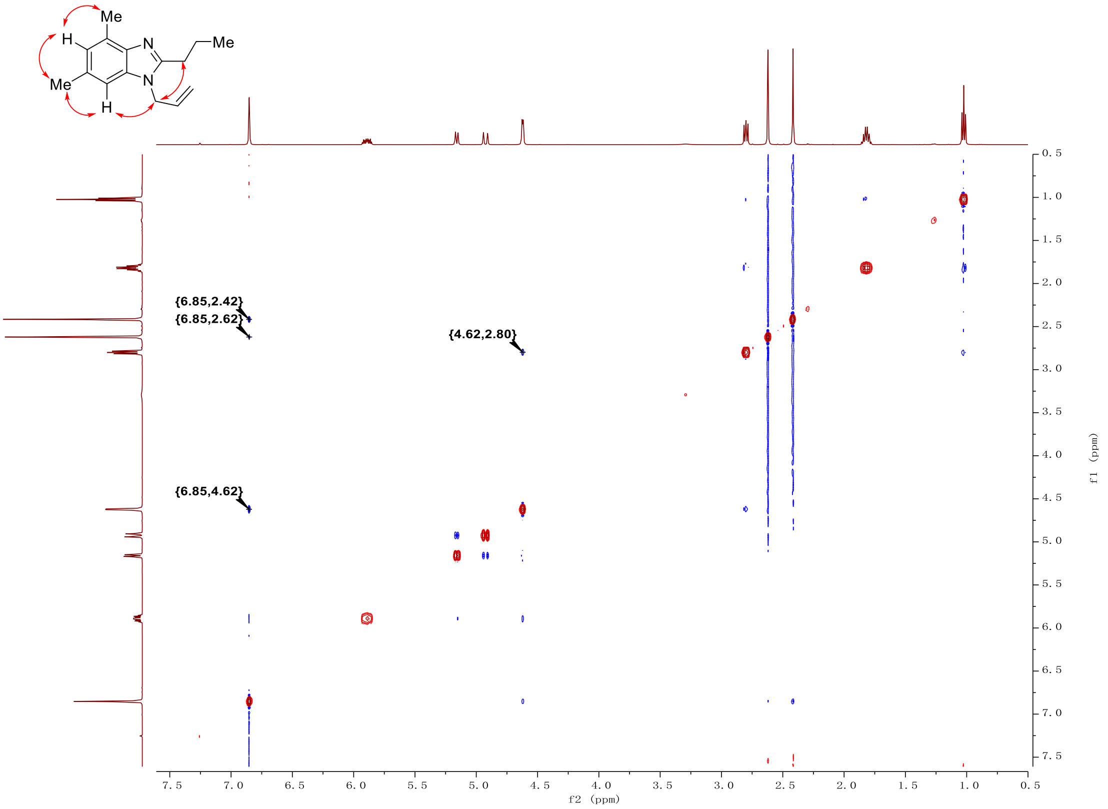

Supplementary Figure 17. ^{1}H NMR and 2D NOESY spectra of compound S21.

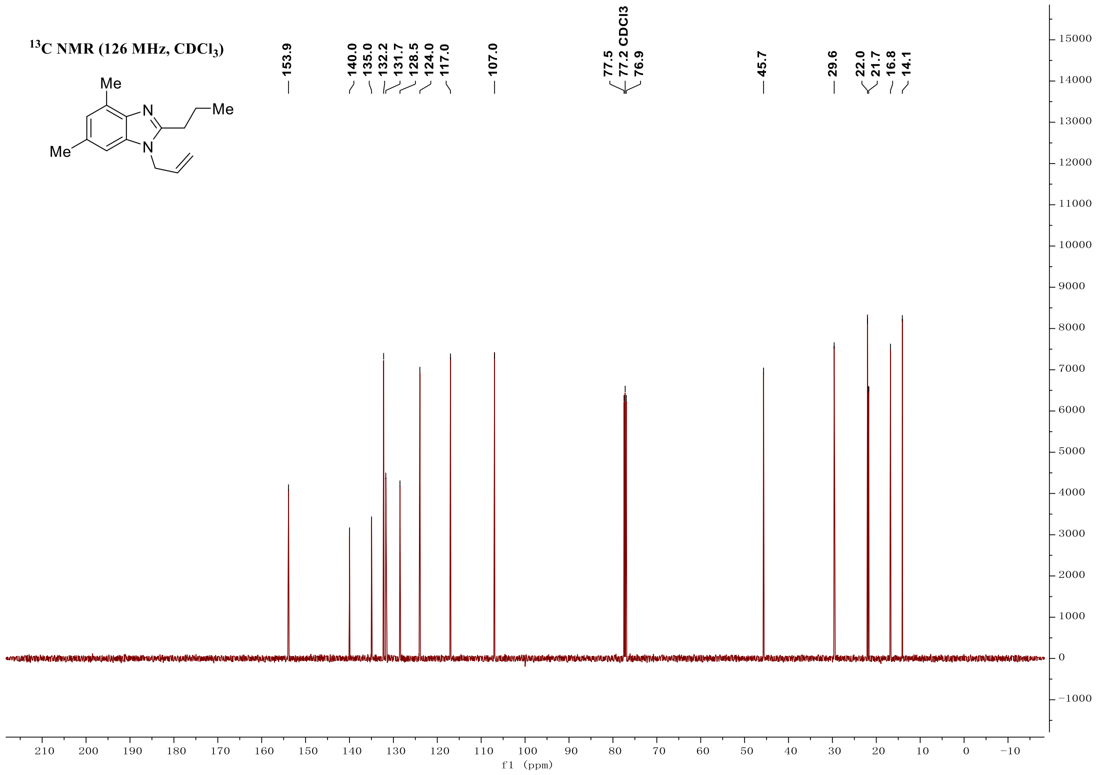

Supplementary Figure 18. ^{13}C NMR spectrum of compound S21.

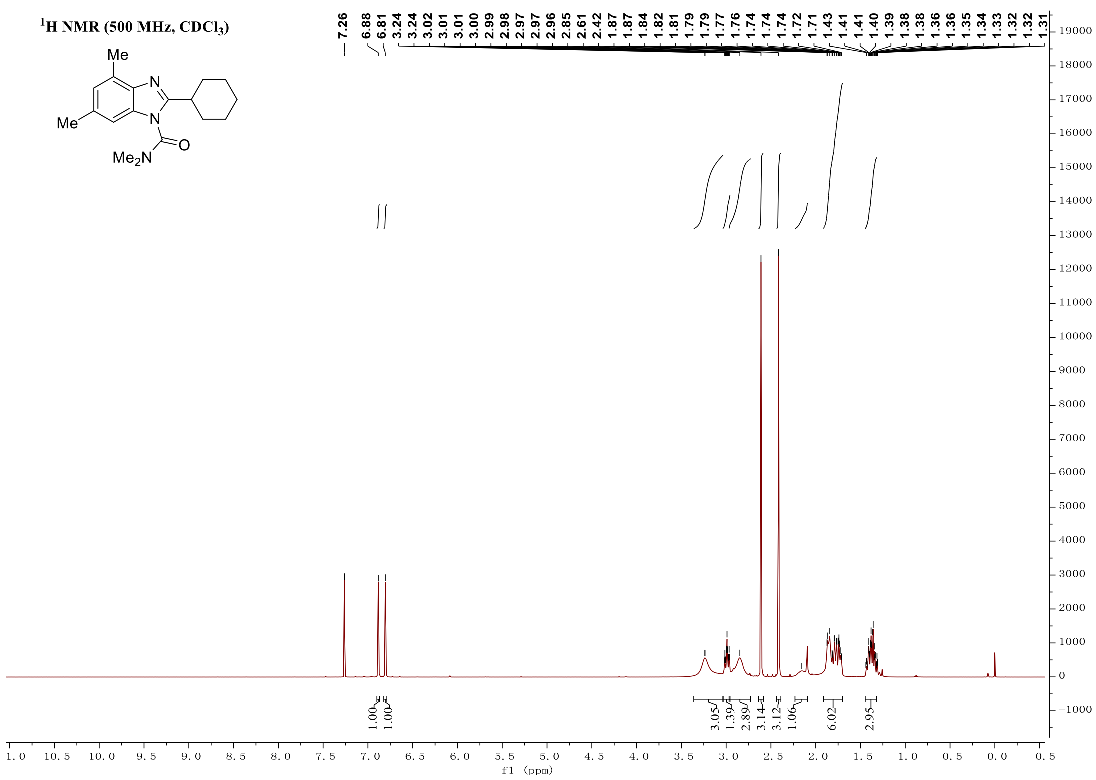

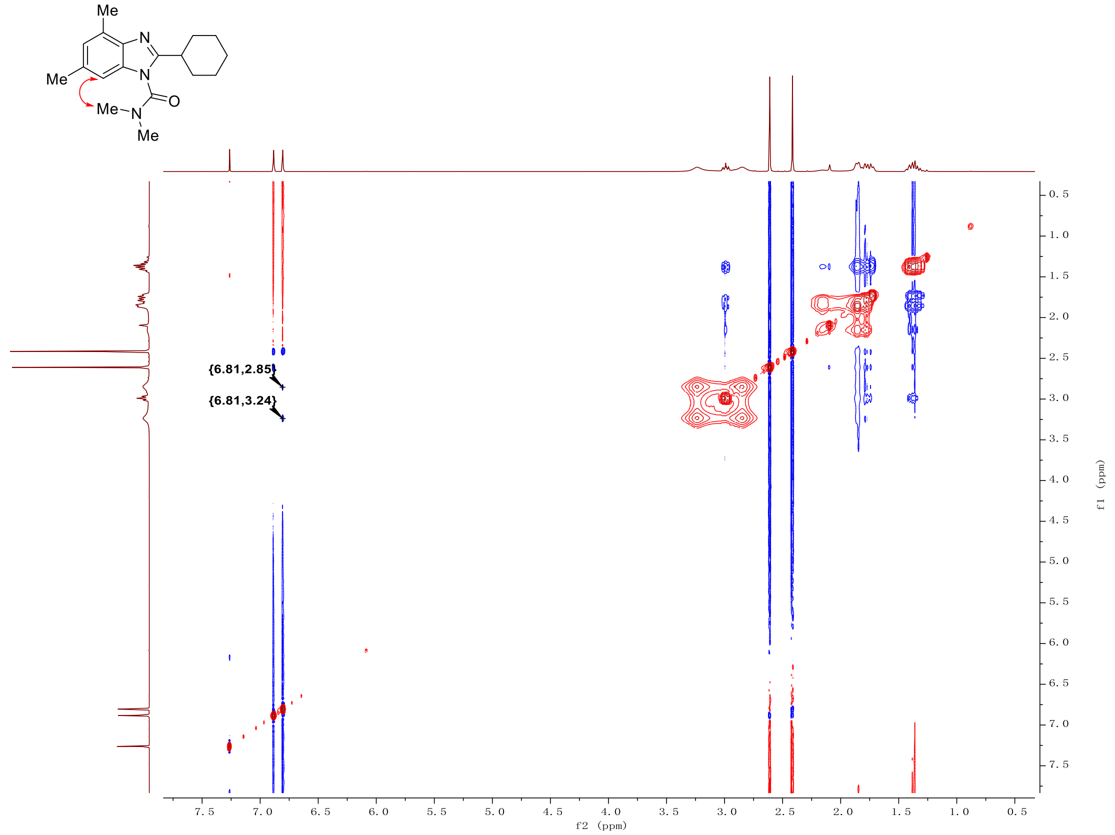

Supplementary Figure 19. ^{1}H NMR and 2D NOESY spectra of compound S22.

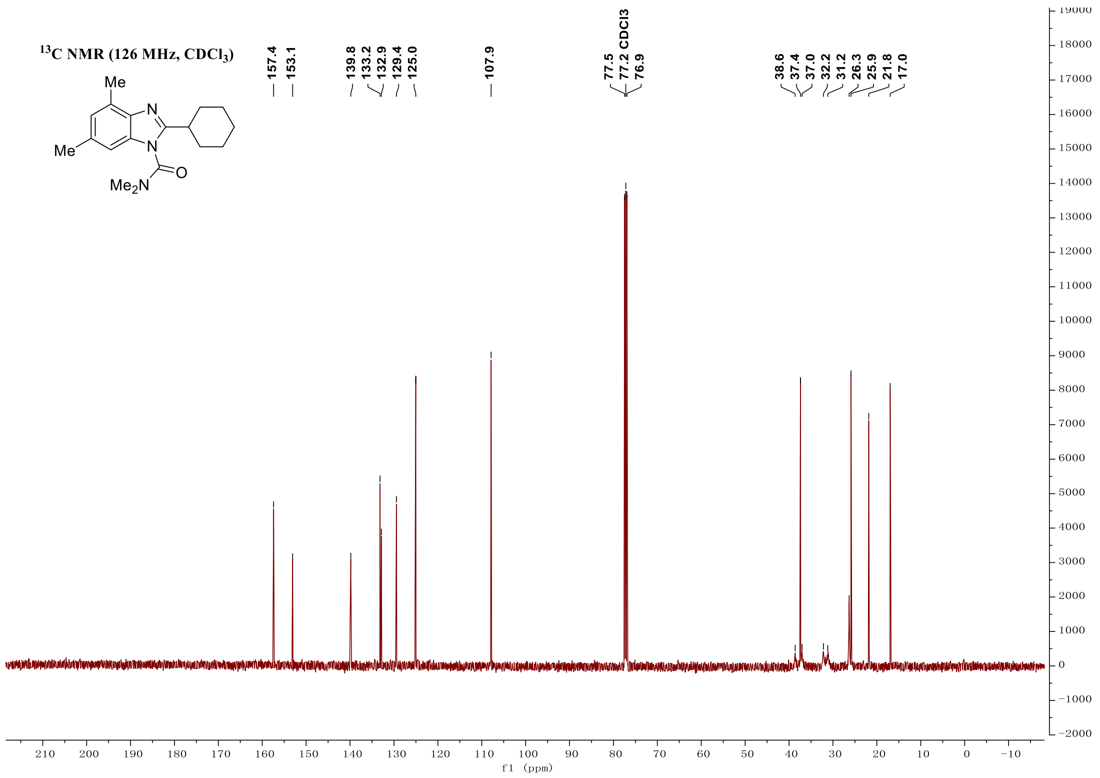

Supplementary Figure 20. ^{13}C NMR spectrum of compound S22.

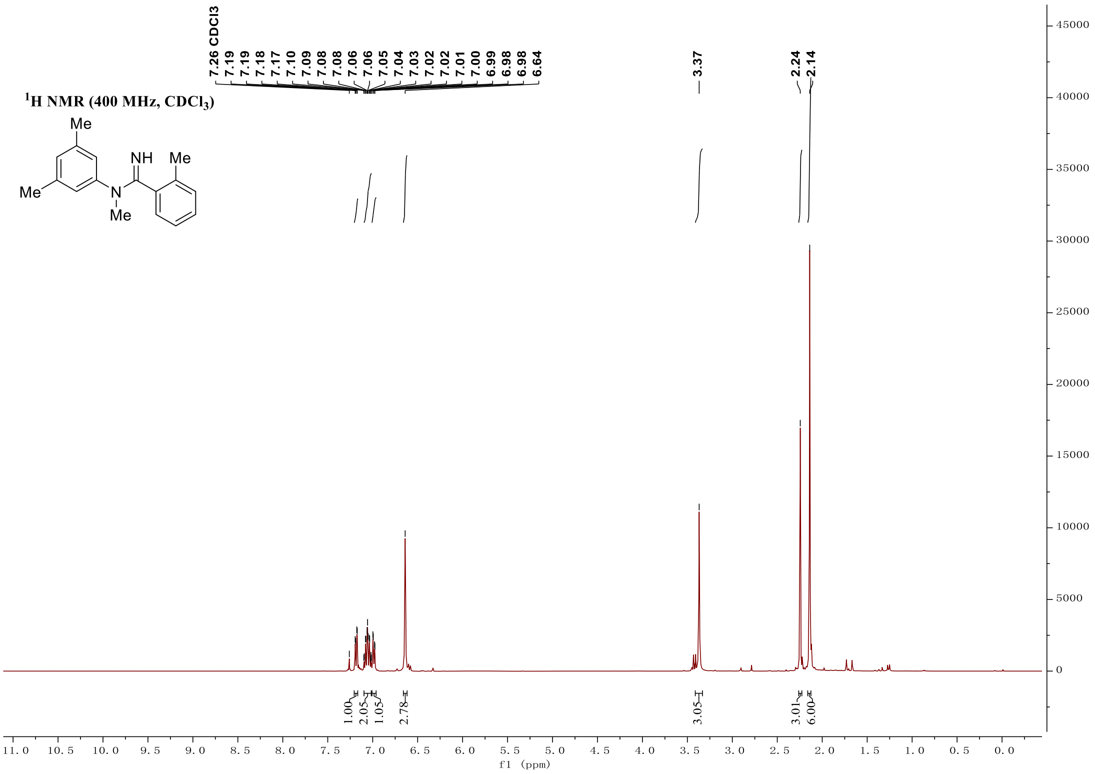

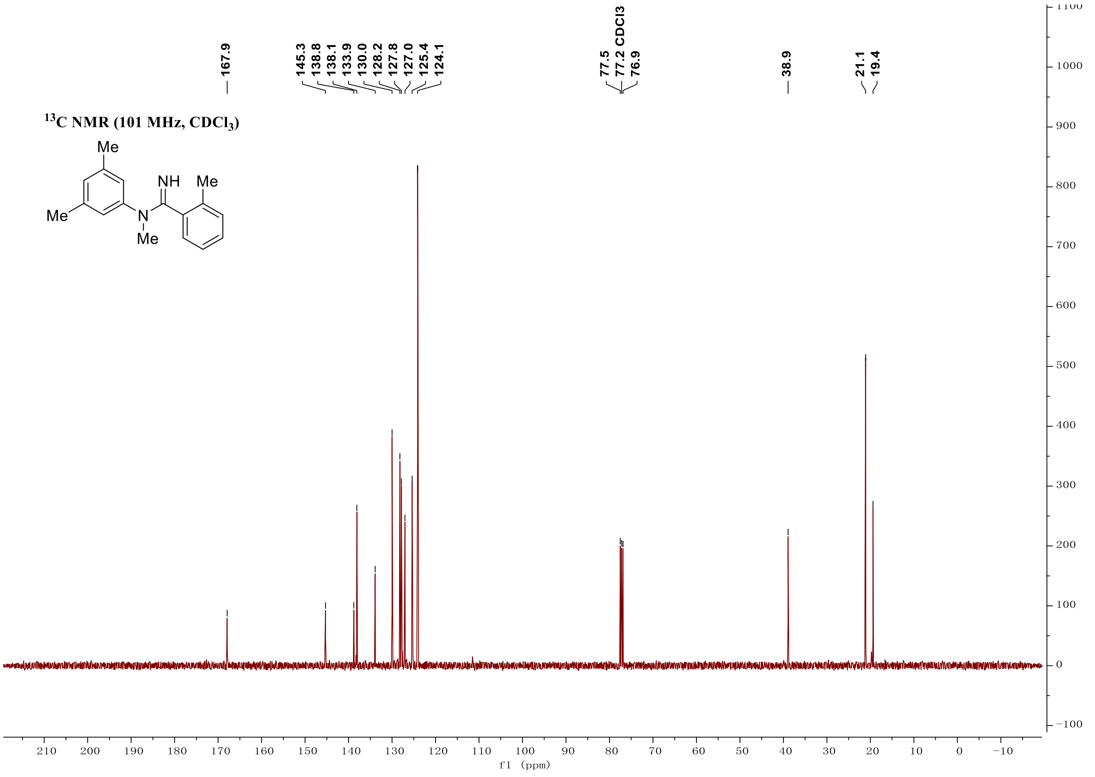

Supplementary Figure 21. ^{1}H NMR and ^{13}C NMR spectra of compound S23.

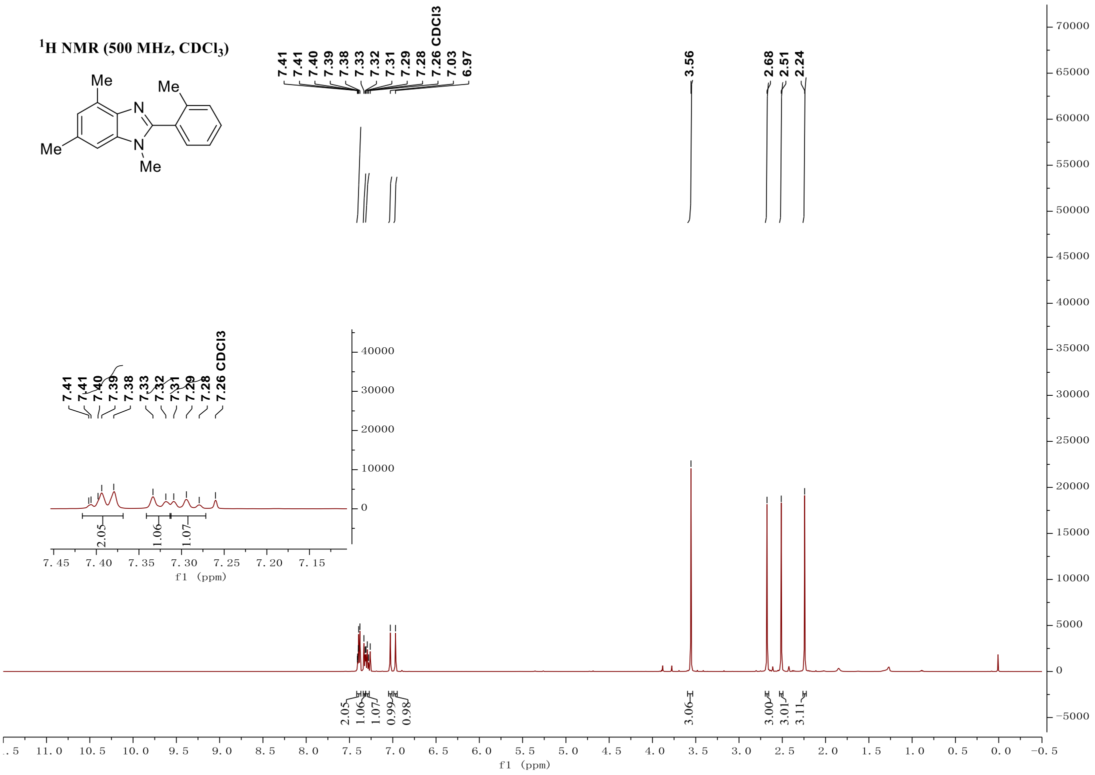

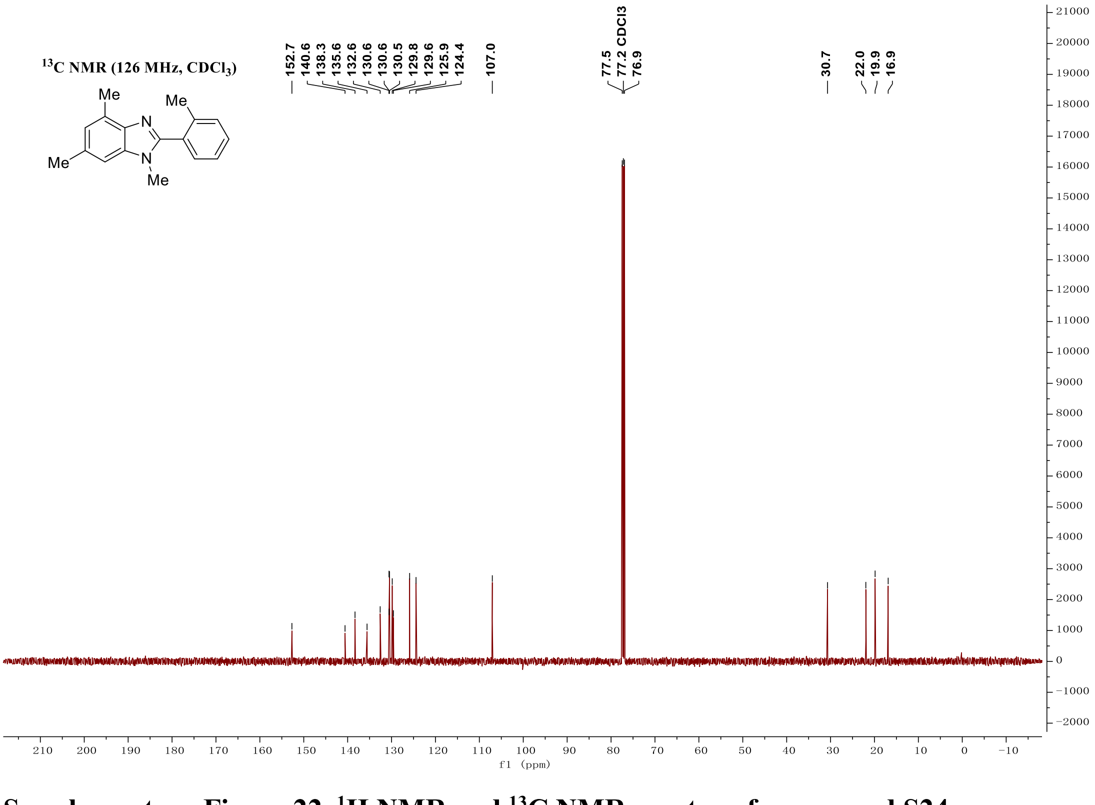

Supplementary Figure 22. ^{1}H NMR and ^{13}C NMR spectra of compound S24.

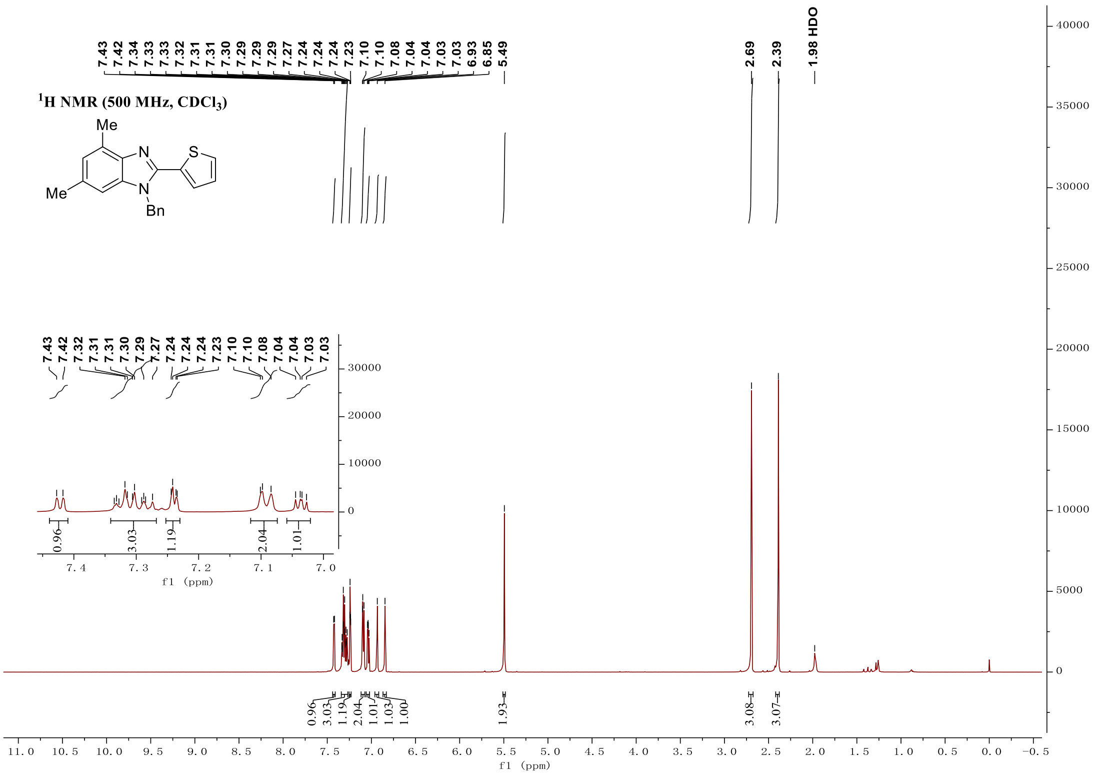

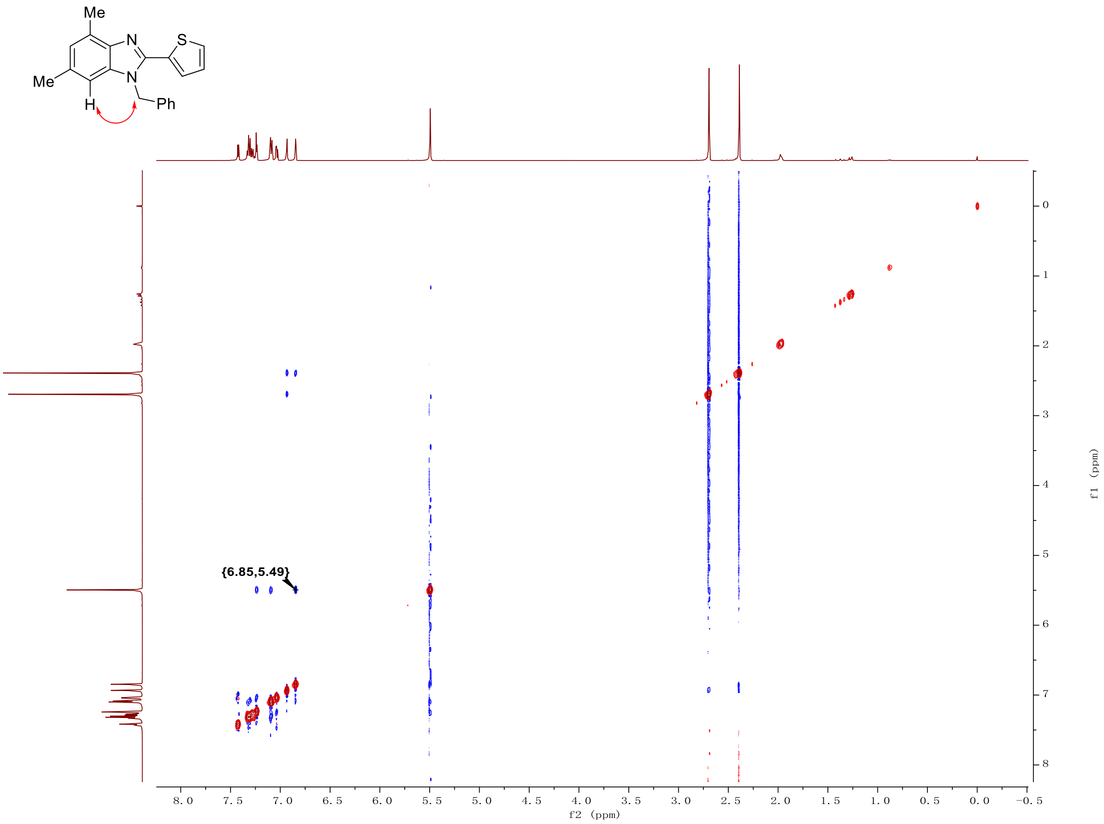

Supplementary Figure 23. ^{1}H NMR and 2D NOESY spectra of compound S26.

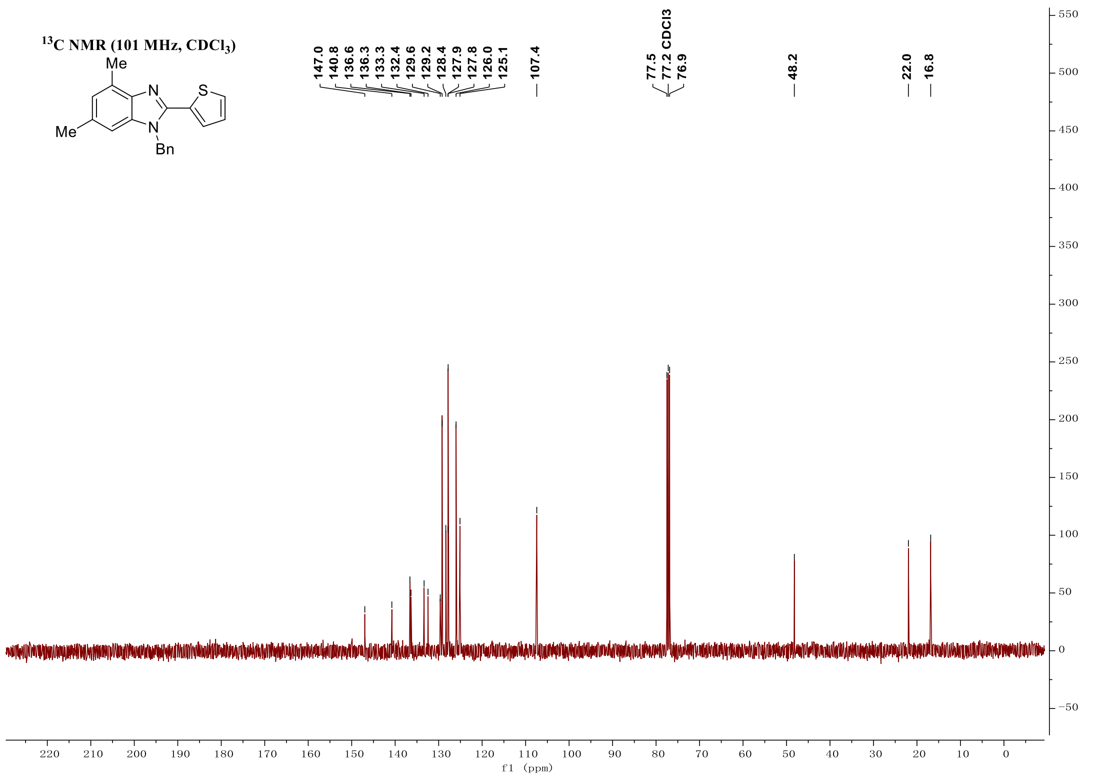

Supplementary Figure 24. ^{13}C NMR spectrum of compound S26.

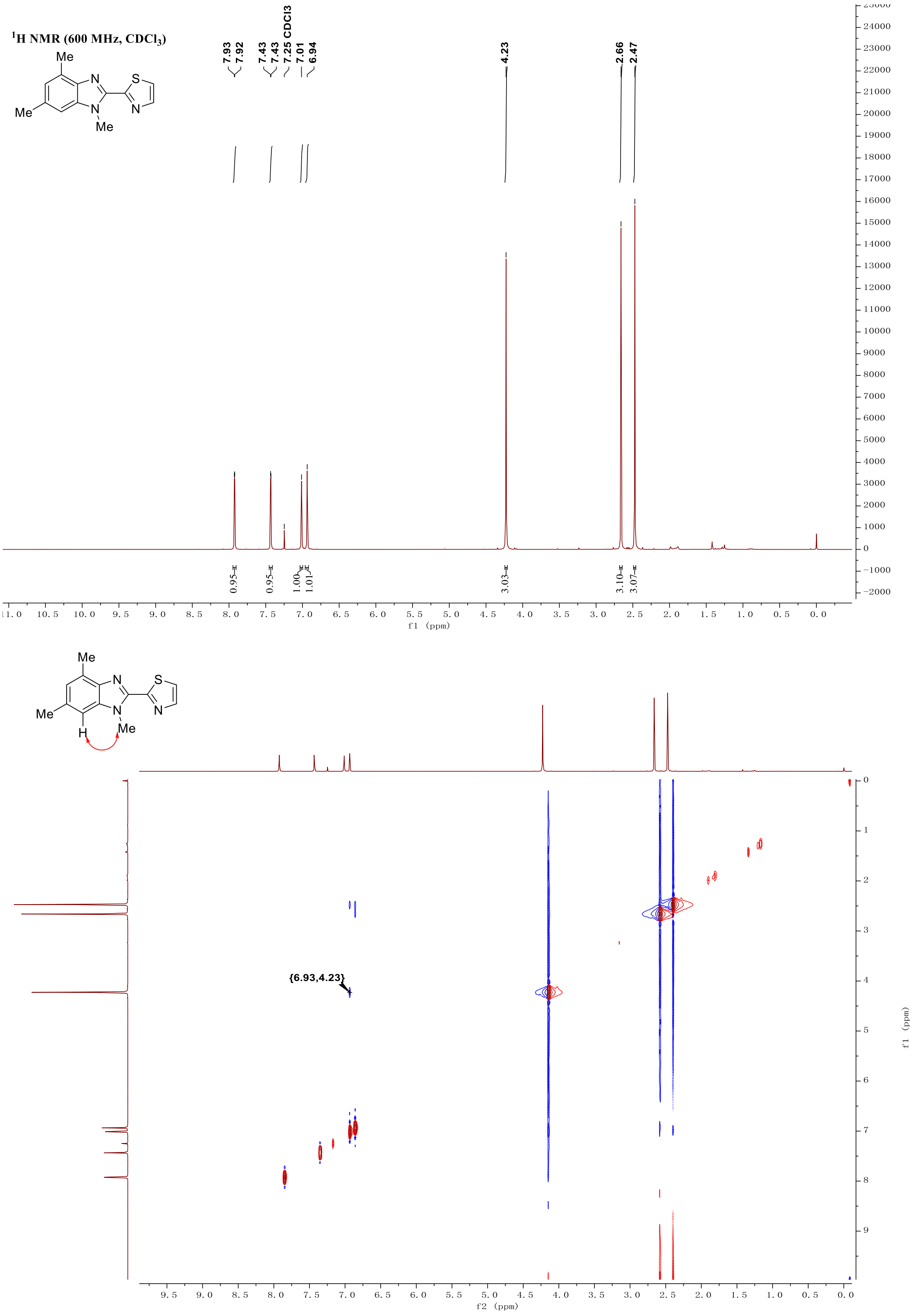

Supplementary Figure 25. ^{1}H NMR and 2D NOESY spectra of compound S29.

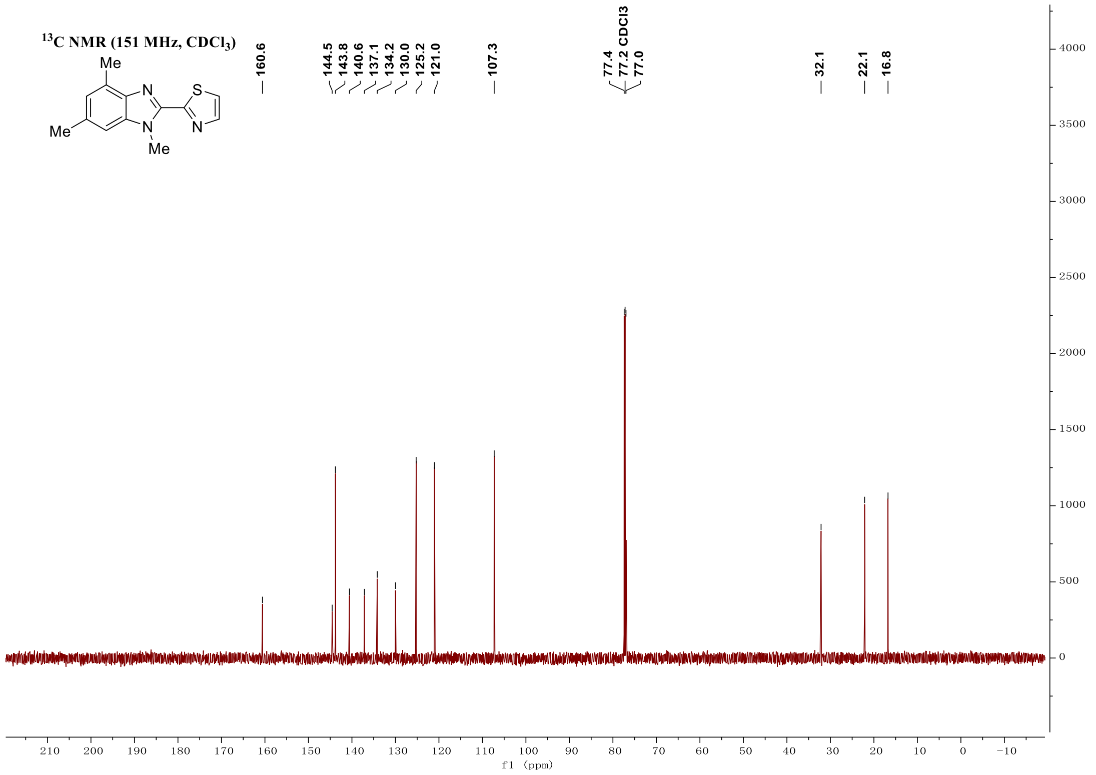

Supplementary Figure 26. ^{13}C NMR spectrum of compound S29.

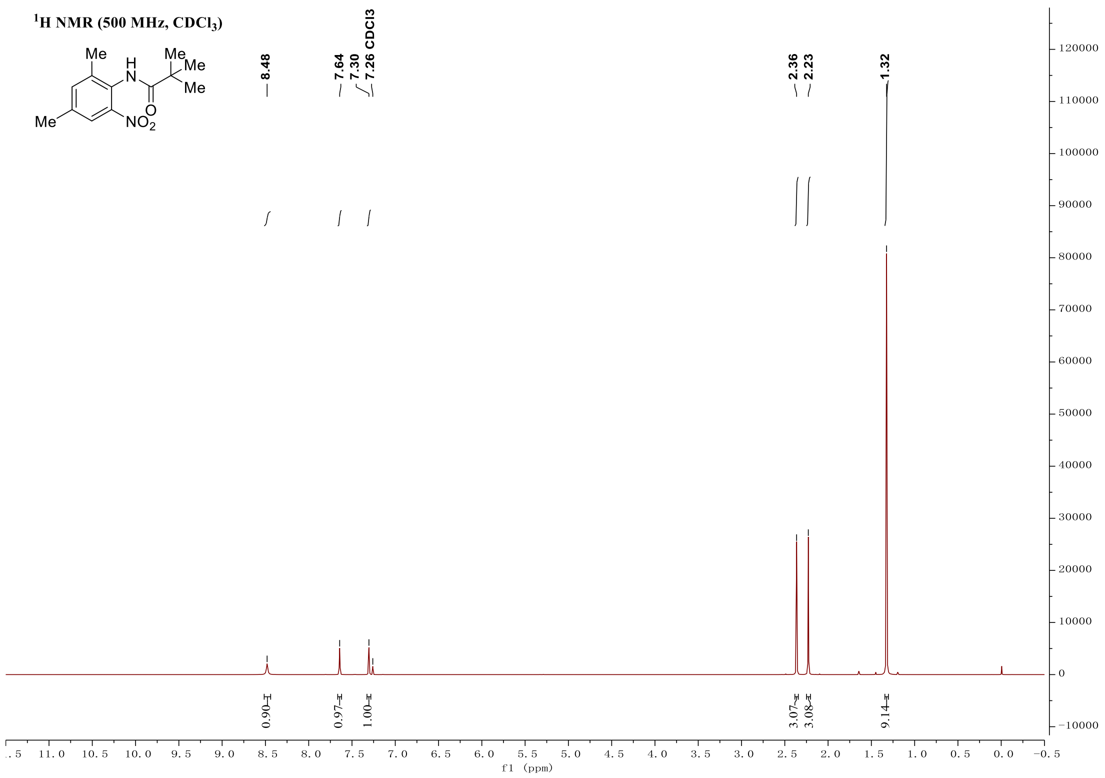

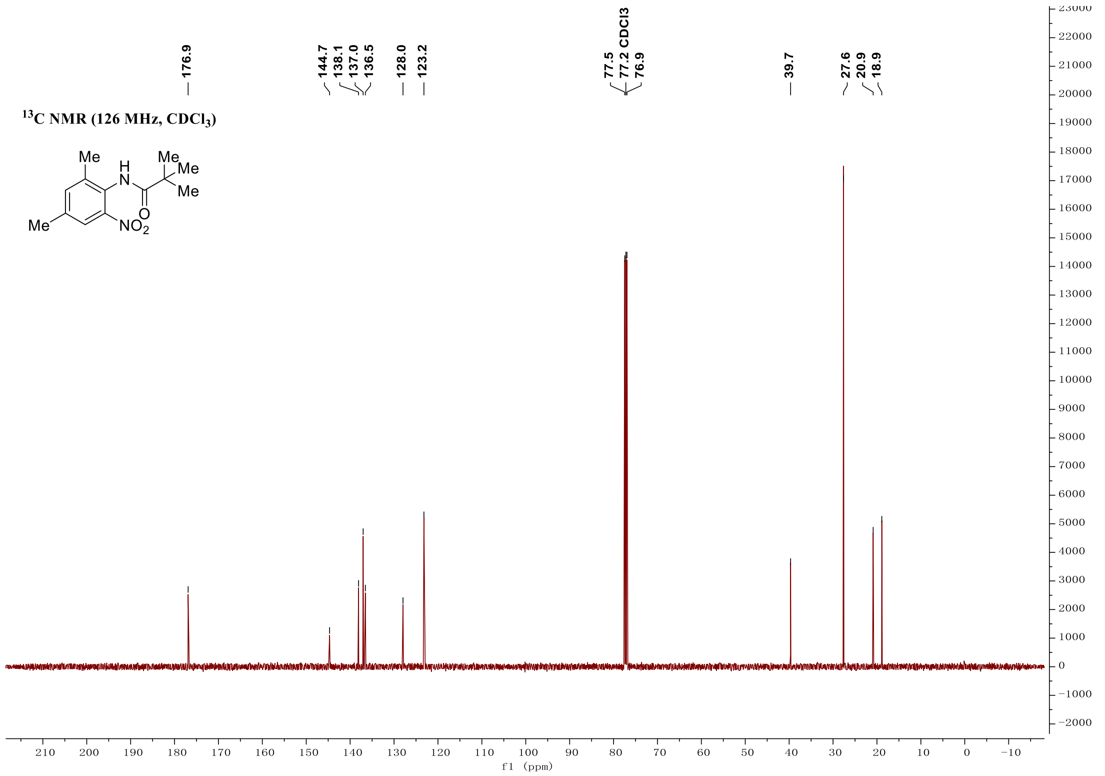

Supplementary Figure 27. ^{1}H NMR and ^{13}C NMR spectra of compound S31.

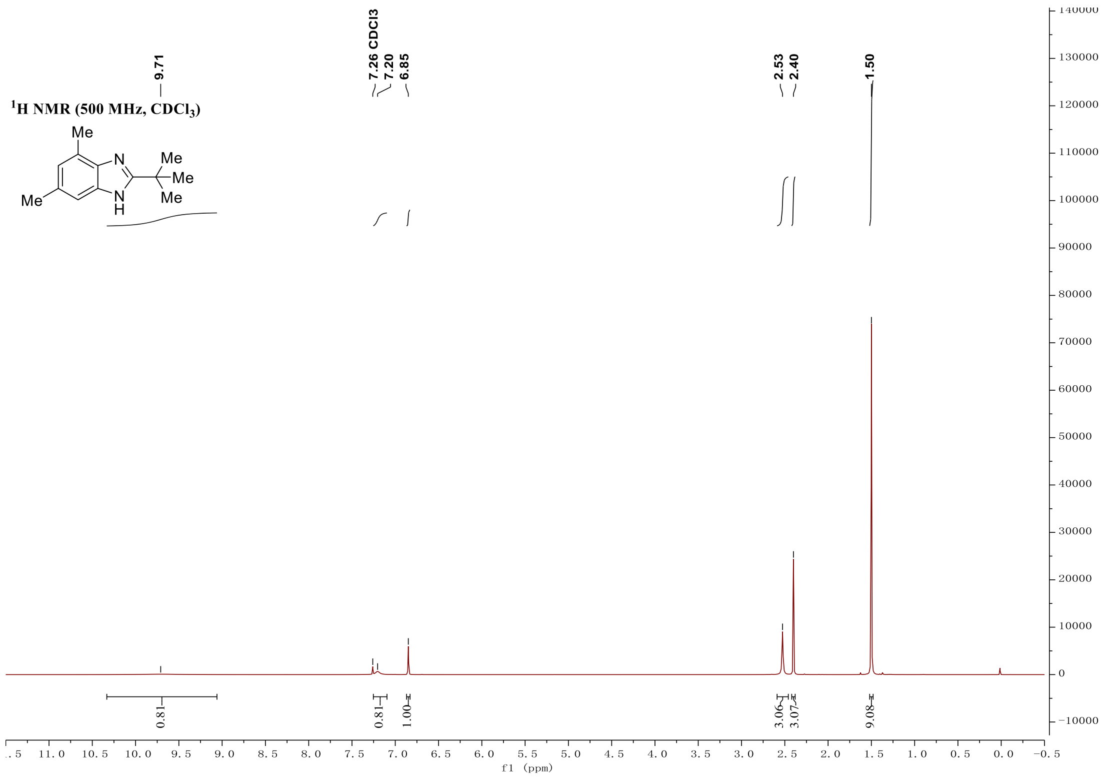

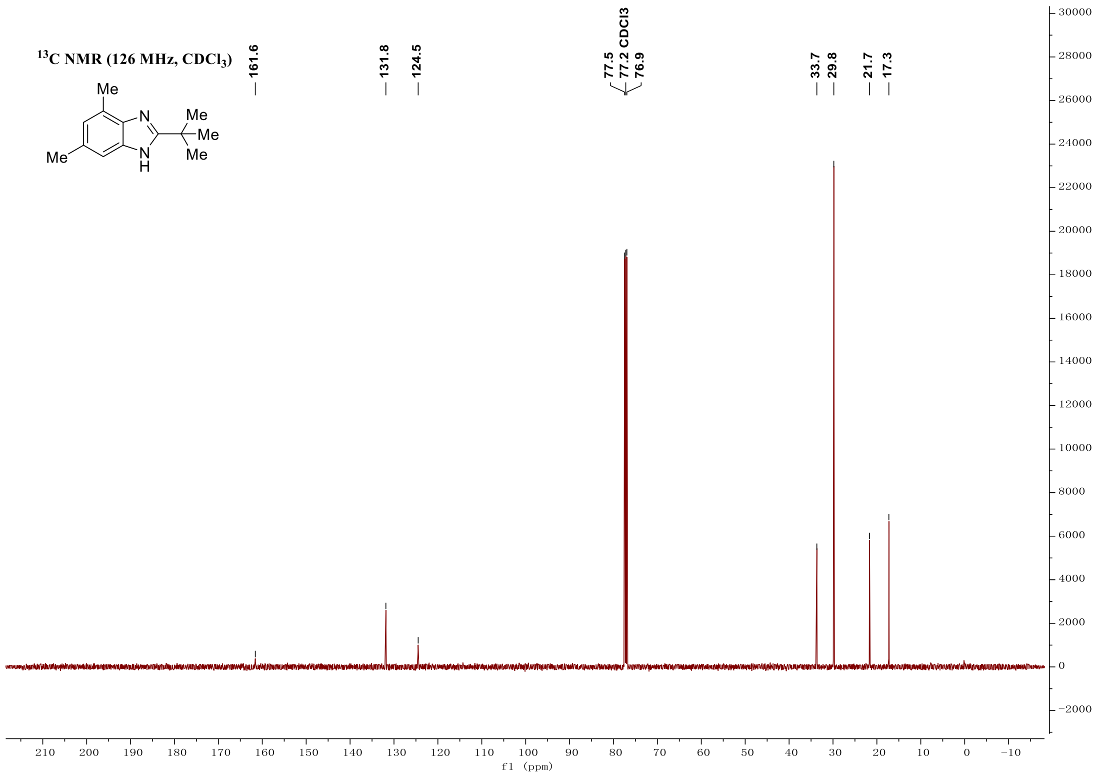

Supplementary Figure 28. ^{1}H NMR and ^{13}C NMR spectra of compound S32.

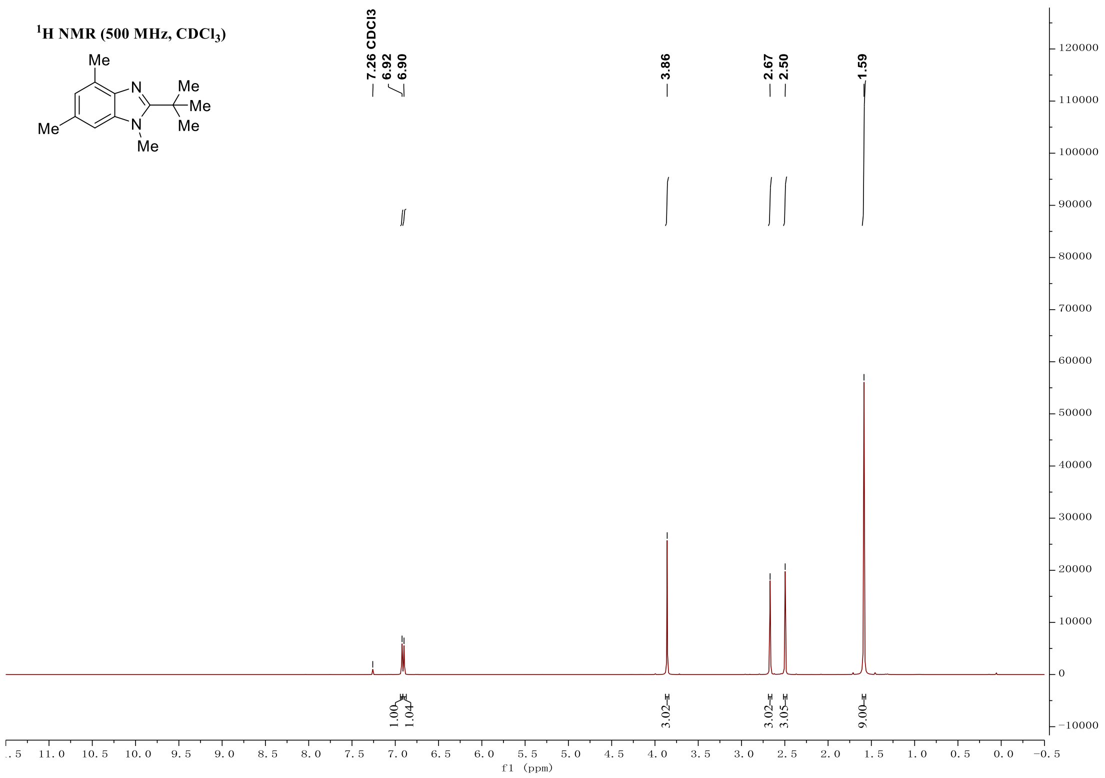

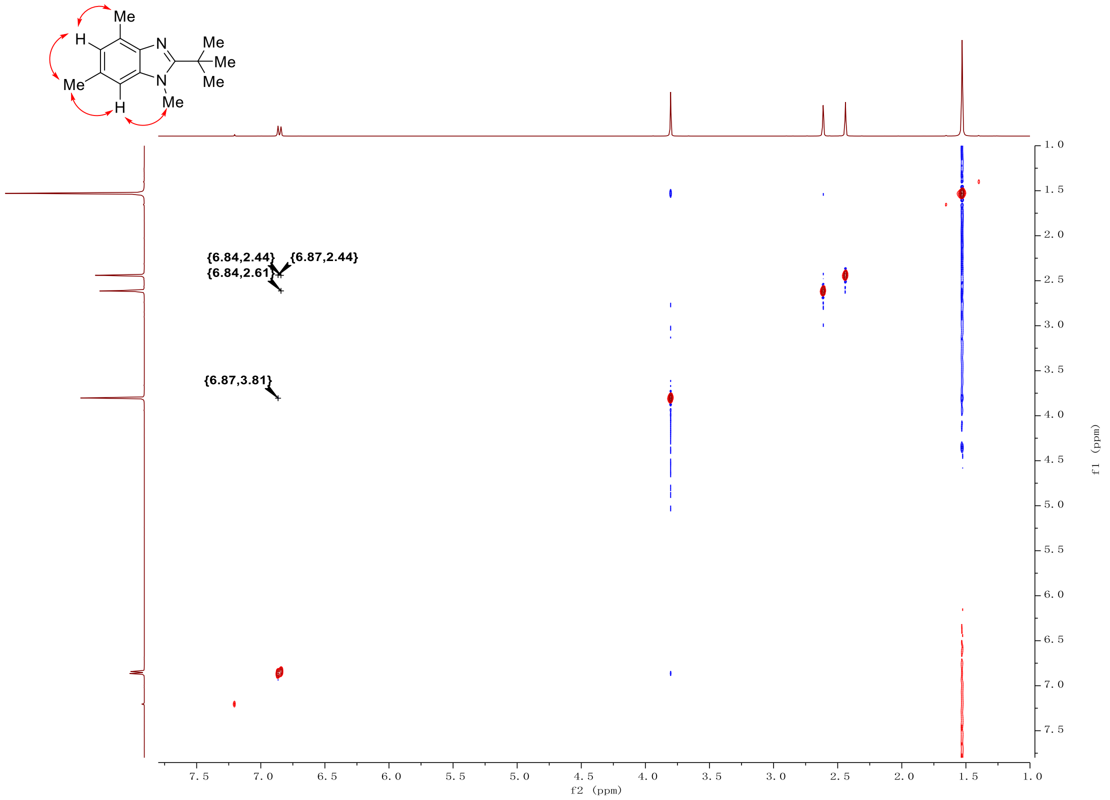

Supplementary Figure 29. ¹H NMR and 2D NOESY spectra of compound S33.

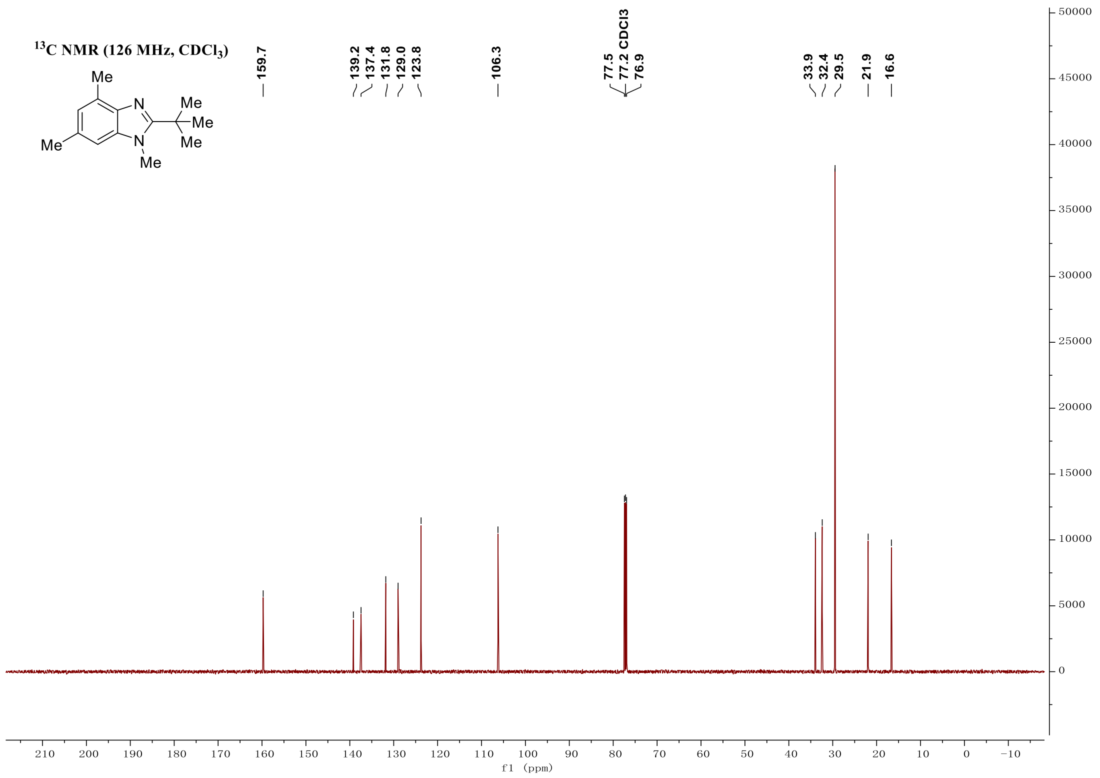

Supplementary Figure 30. ^{13}C NMR spectrum of compound S33.

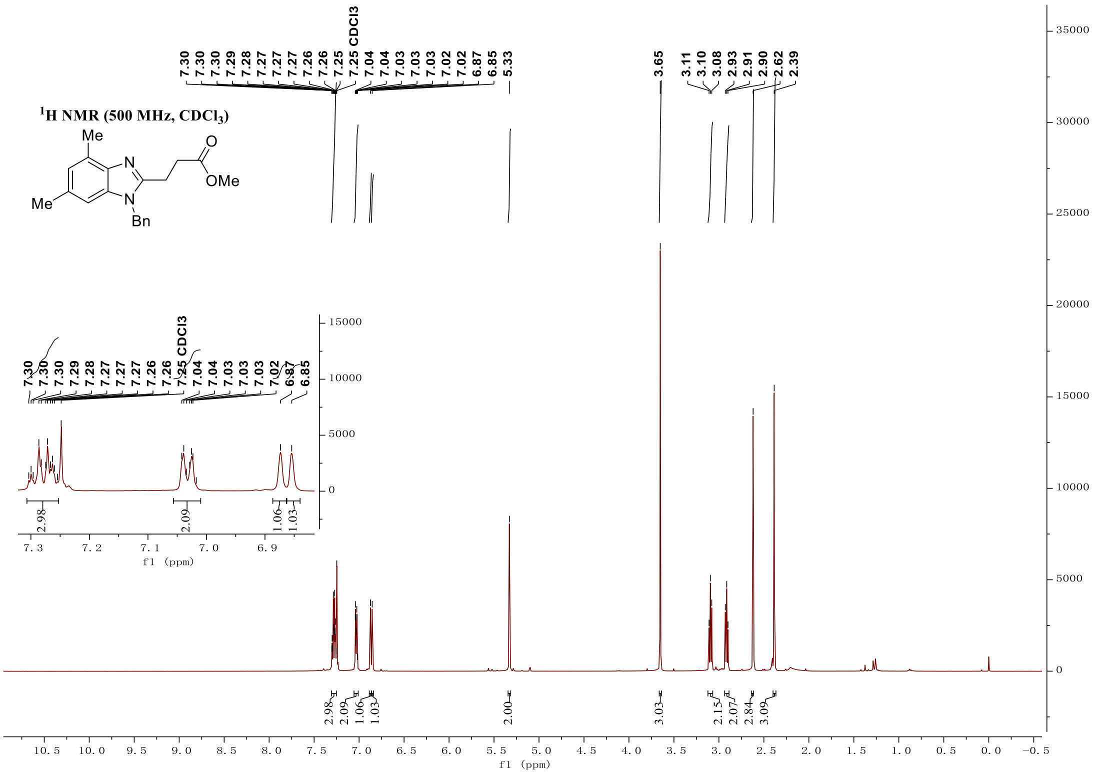

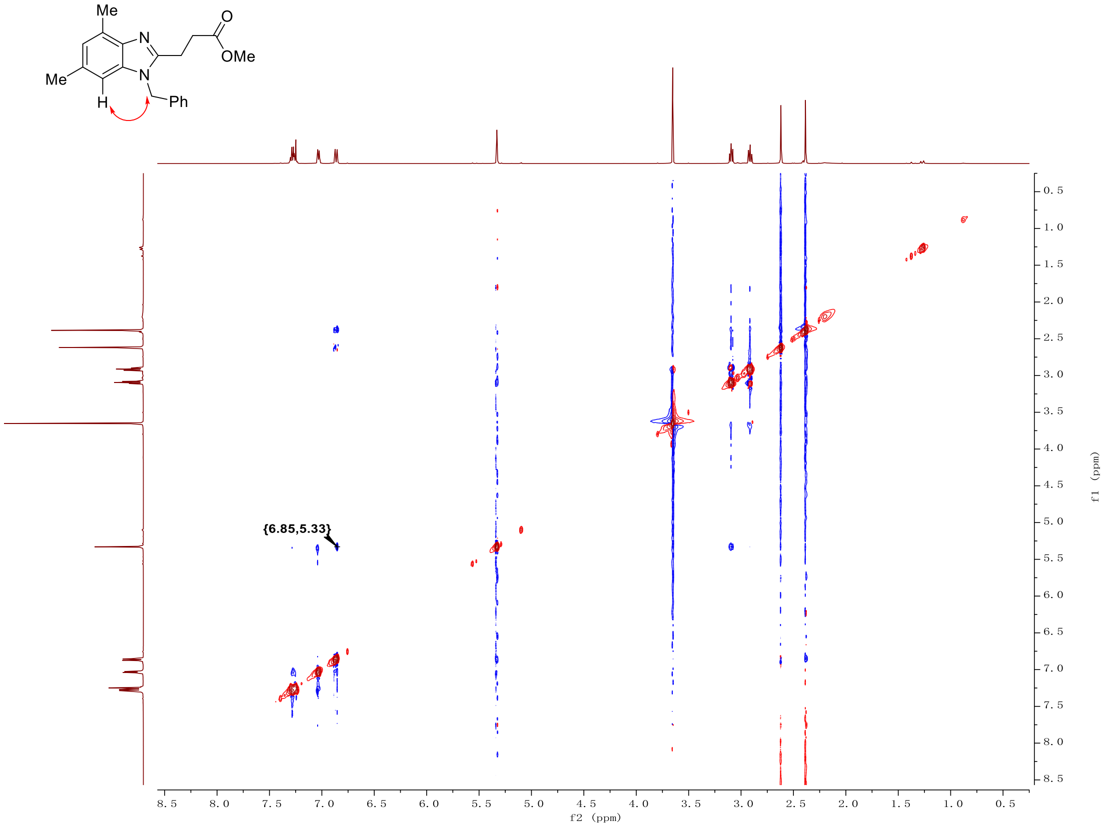

Supplementary Figure 31. ^{1}H NMR and 2D NOESY spectra of compound S36.

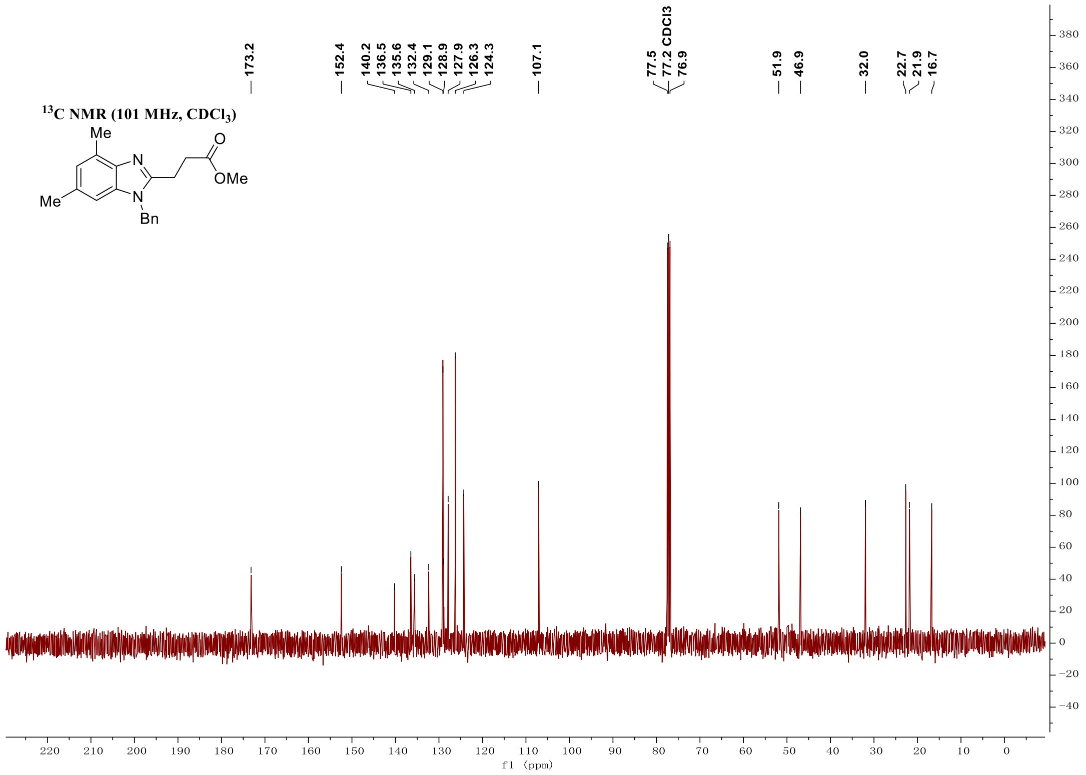

Supplementary Figure 32. ^{13}C NMR spectrum of compound S36.

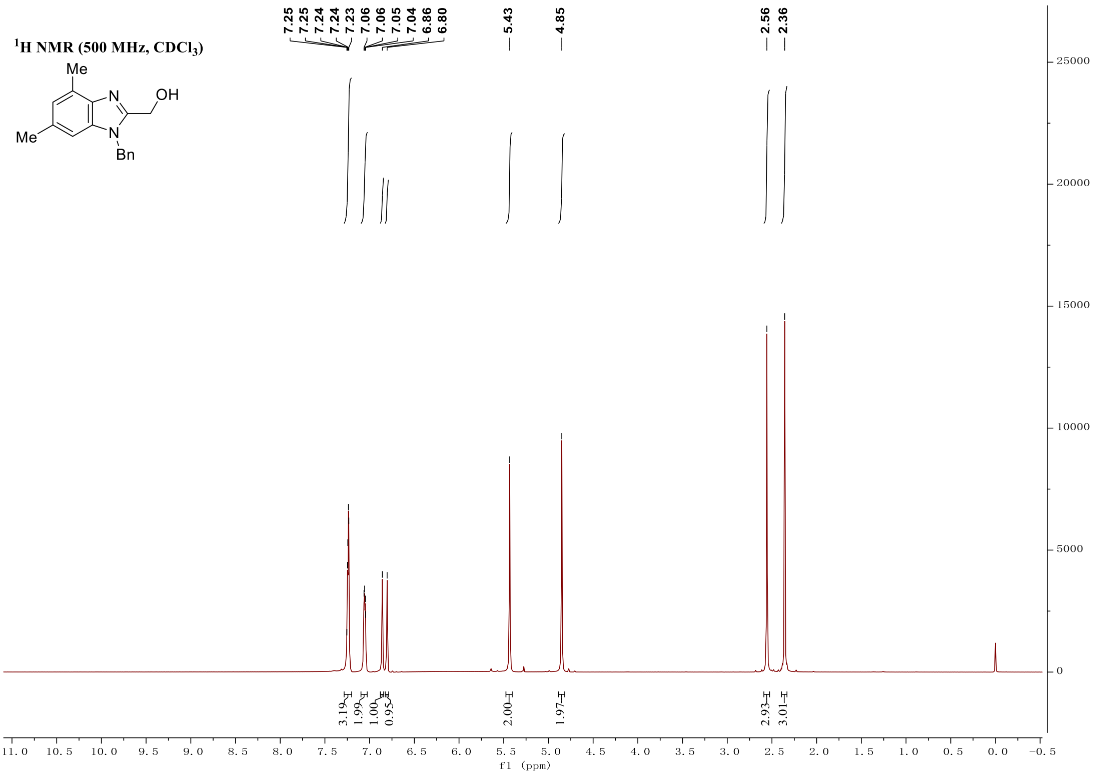

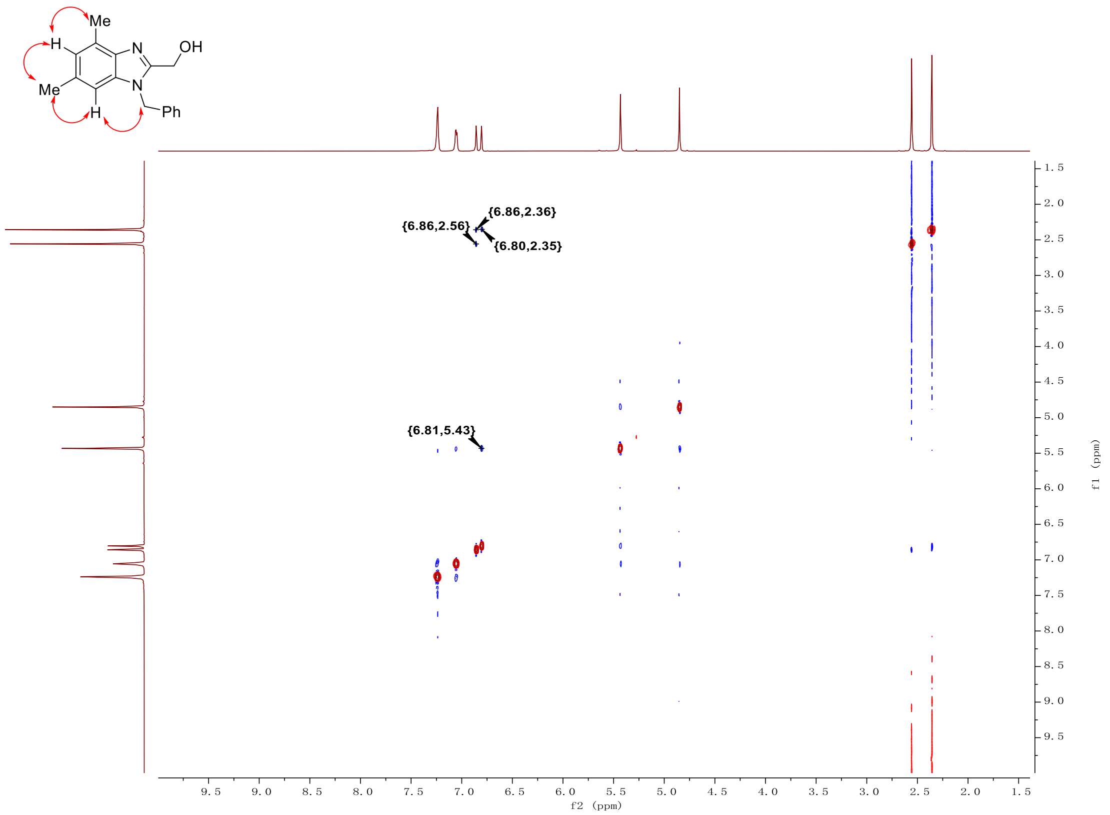

Supplementary Figure 33. ^{1}H NMR and 2D NOESY spectra of compound S38.

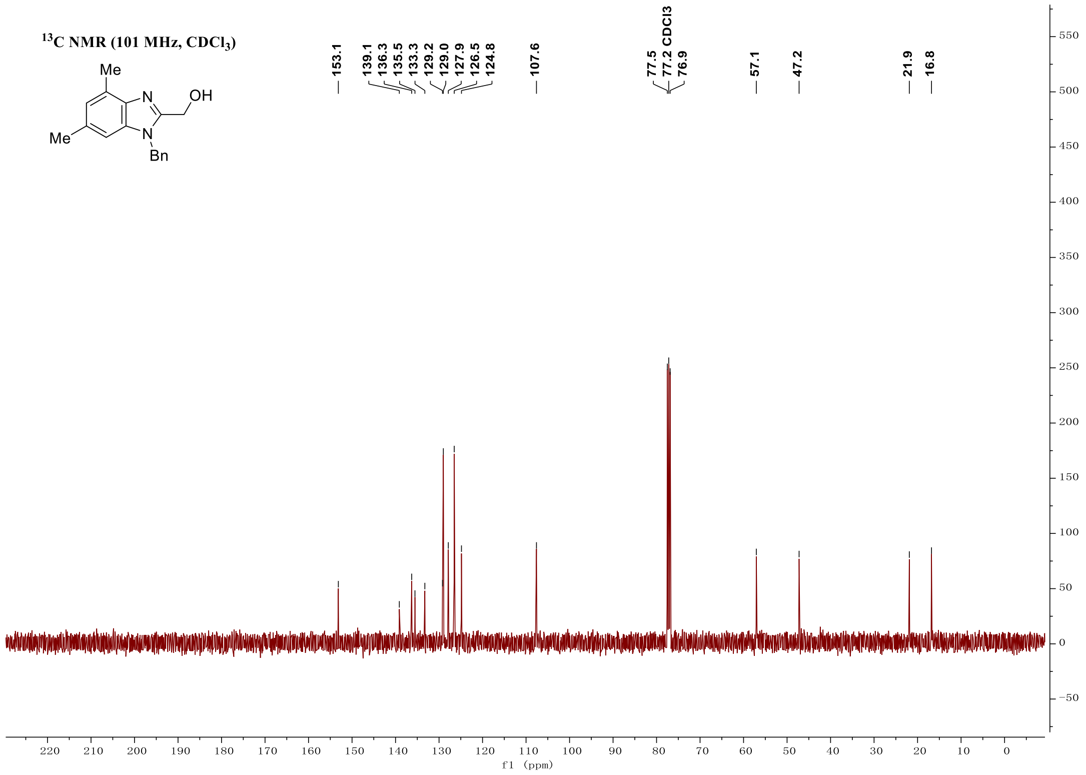

Supplementary Figure 34. ^{13}C NMR spectrum of compound S38.

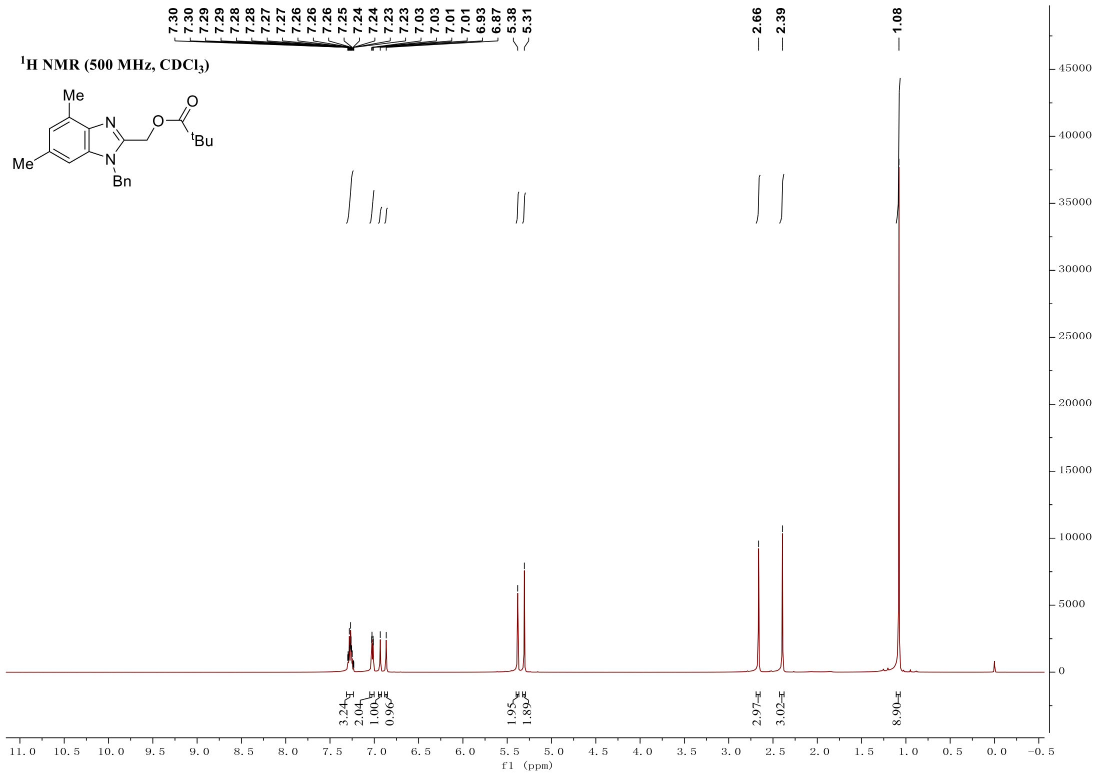

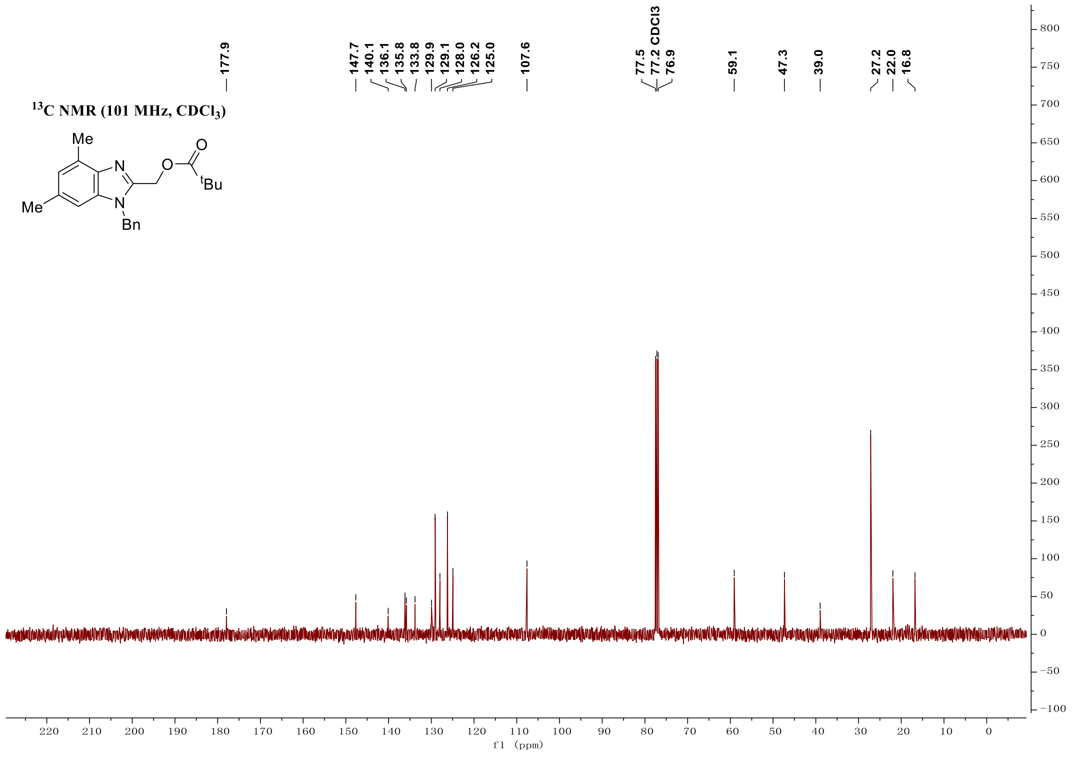

Supplementary Figure 35. ^{1}H NMR and ^{13}C NMR spectra of compound S39.

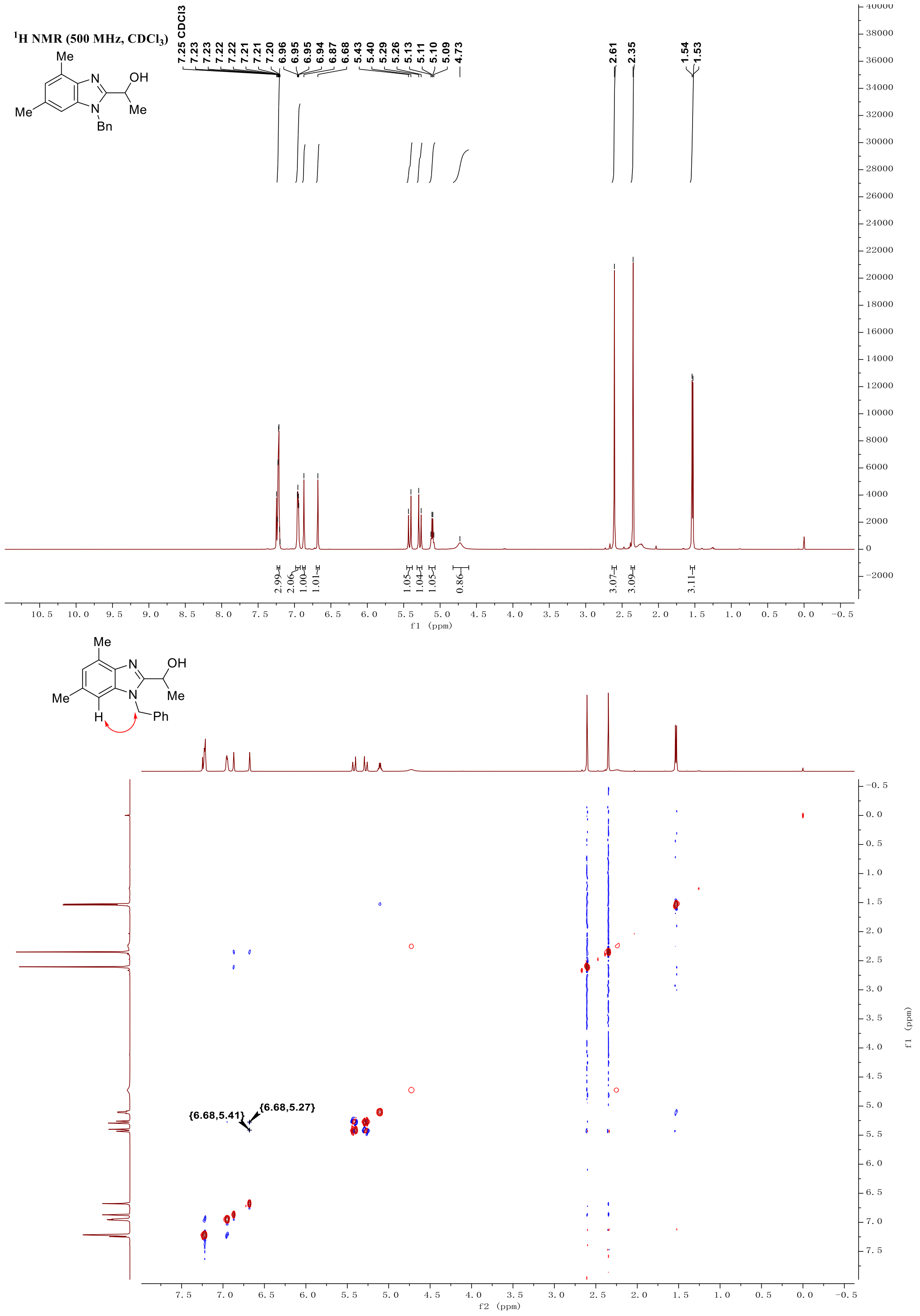

Supplementary Figure 36. ^{1}H NMR and 2D NOESY spectra of compound S41.

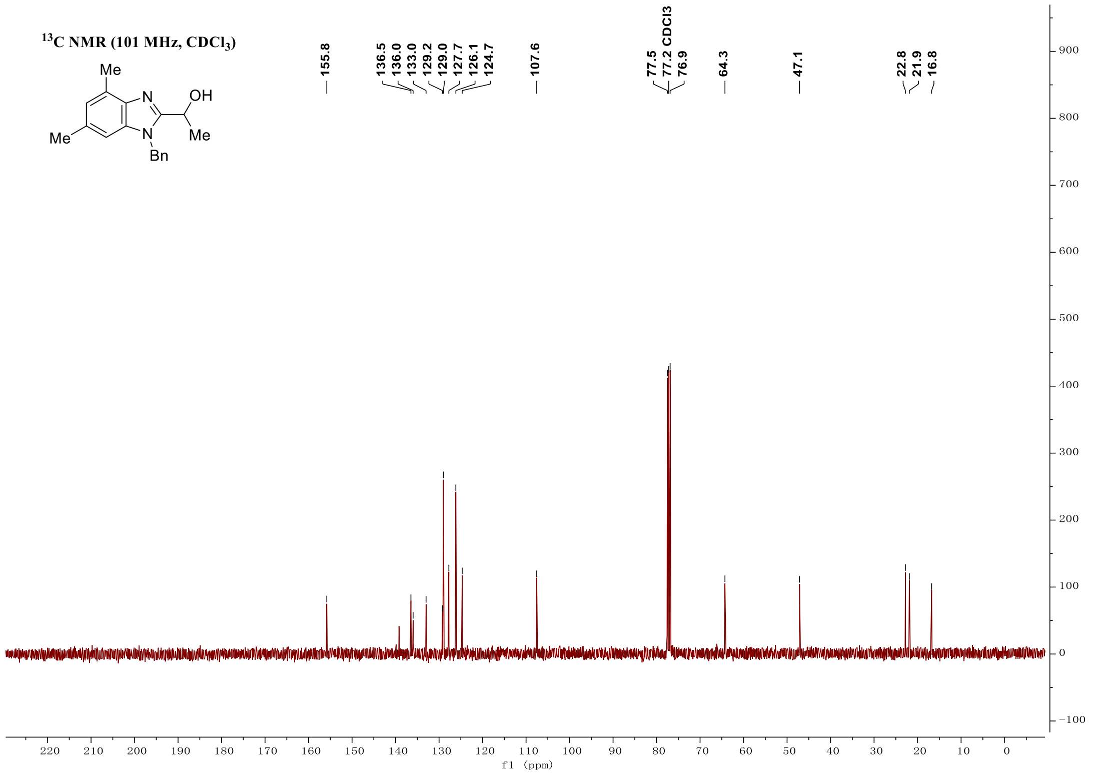

Supplementary Figure 37. ^{13}C NMR spectrum of compound S41.

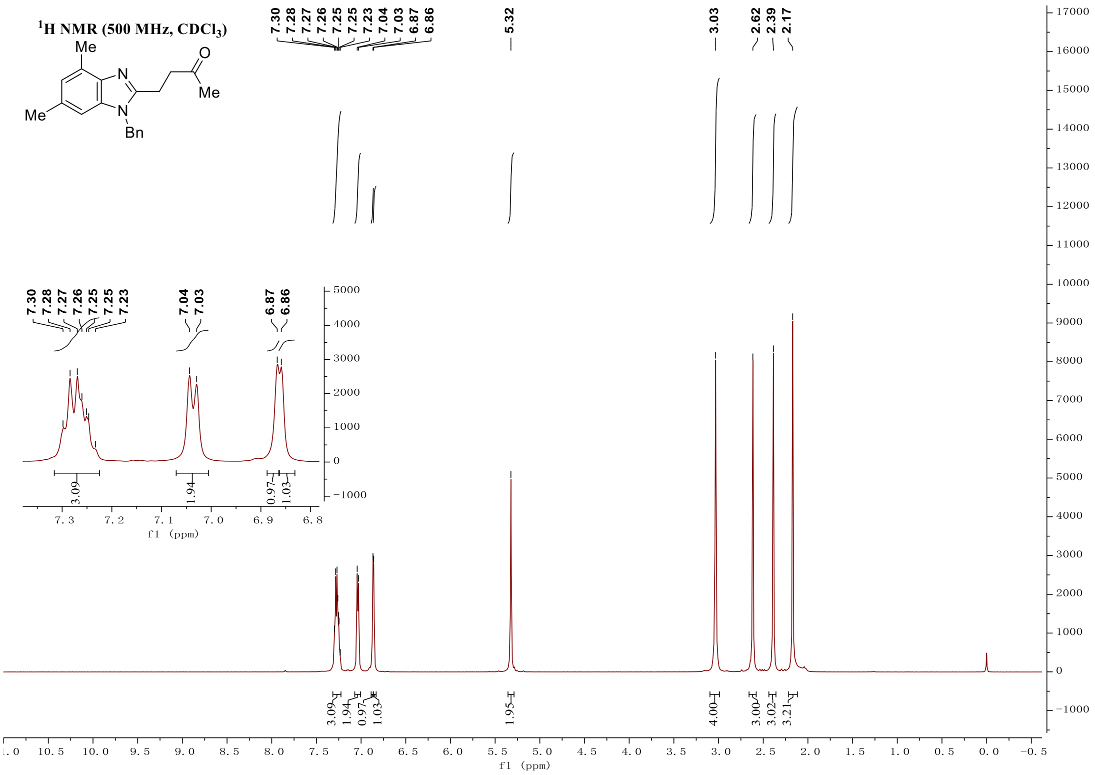

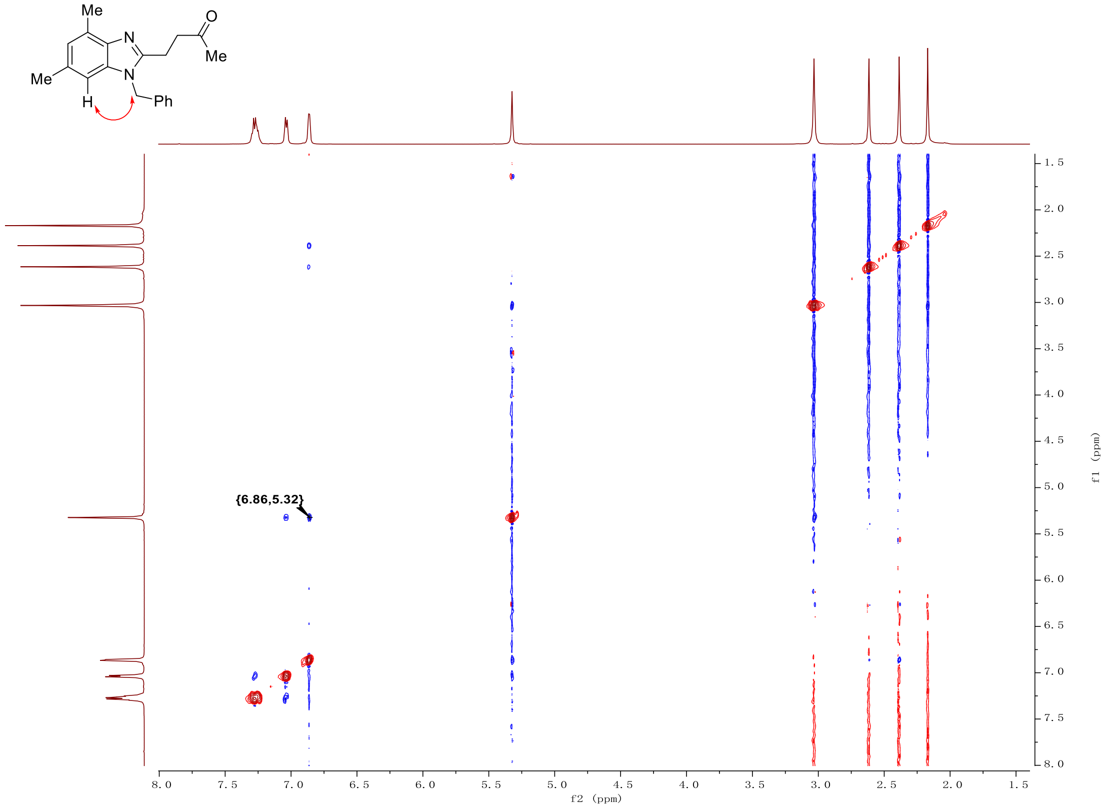

Supplementary Figure 38. ^{1}H NMR and 2D NOESY spectra of compound S43.

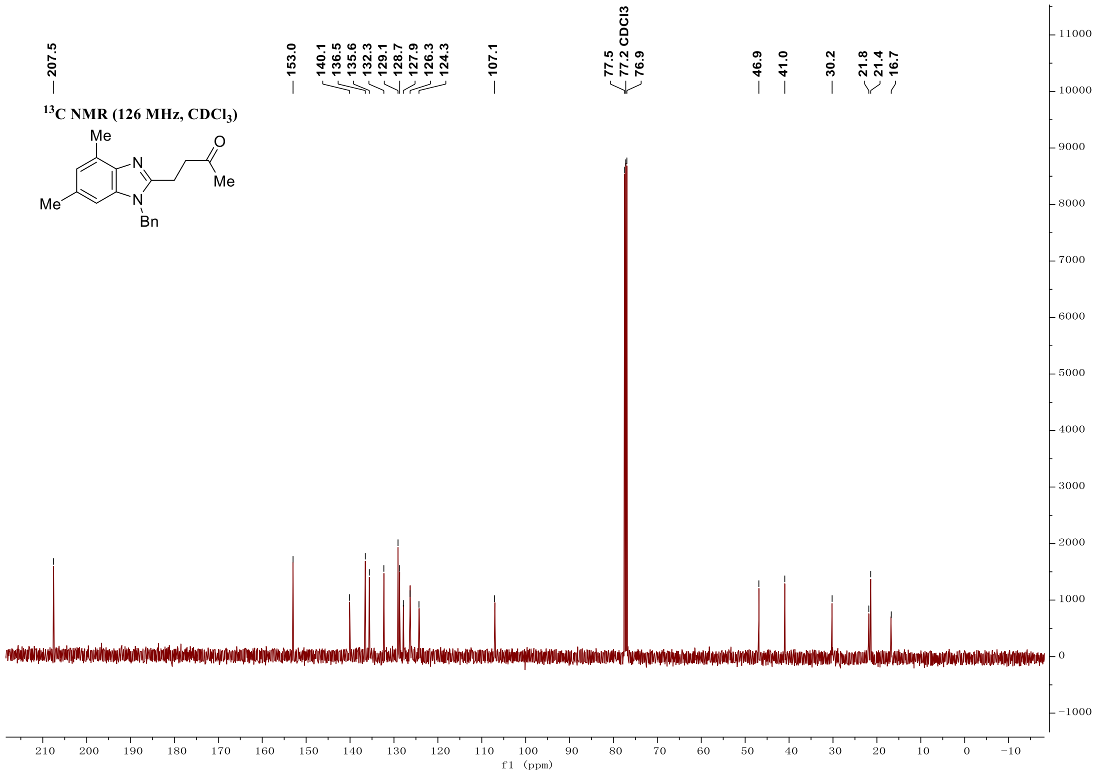

Supplementary Figure 39. ^{13}C NMR spectrum of compound S43.

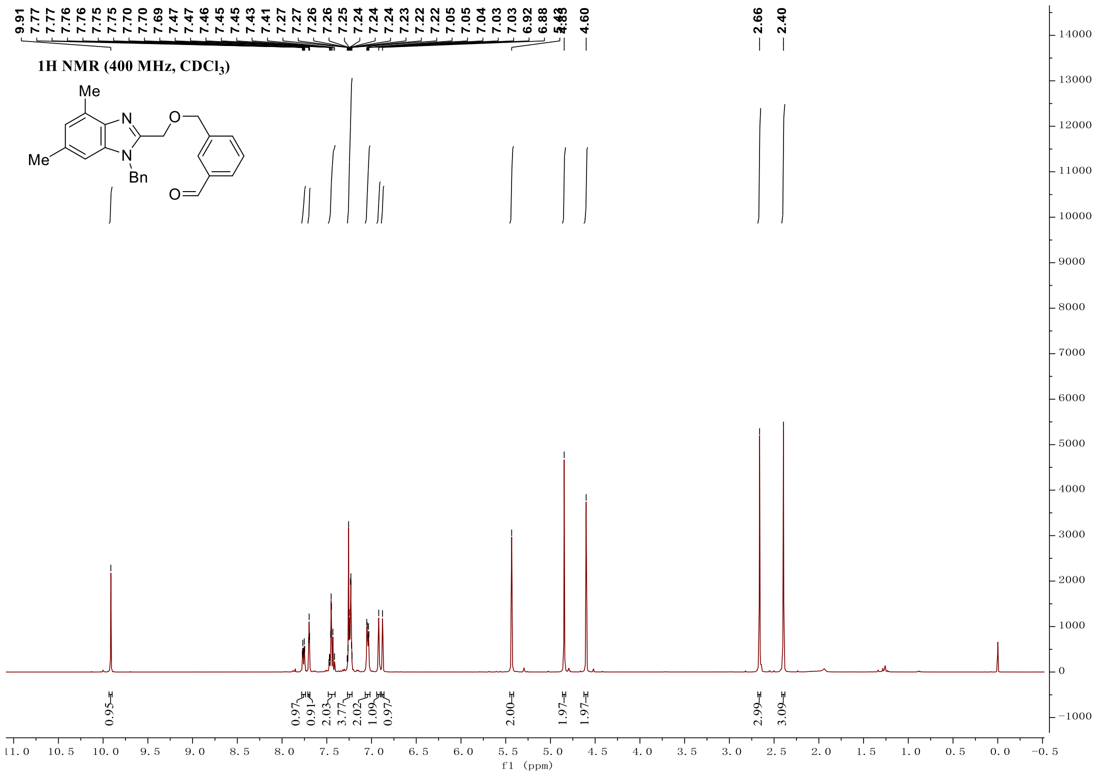

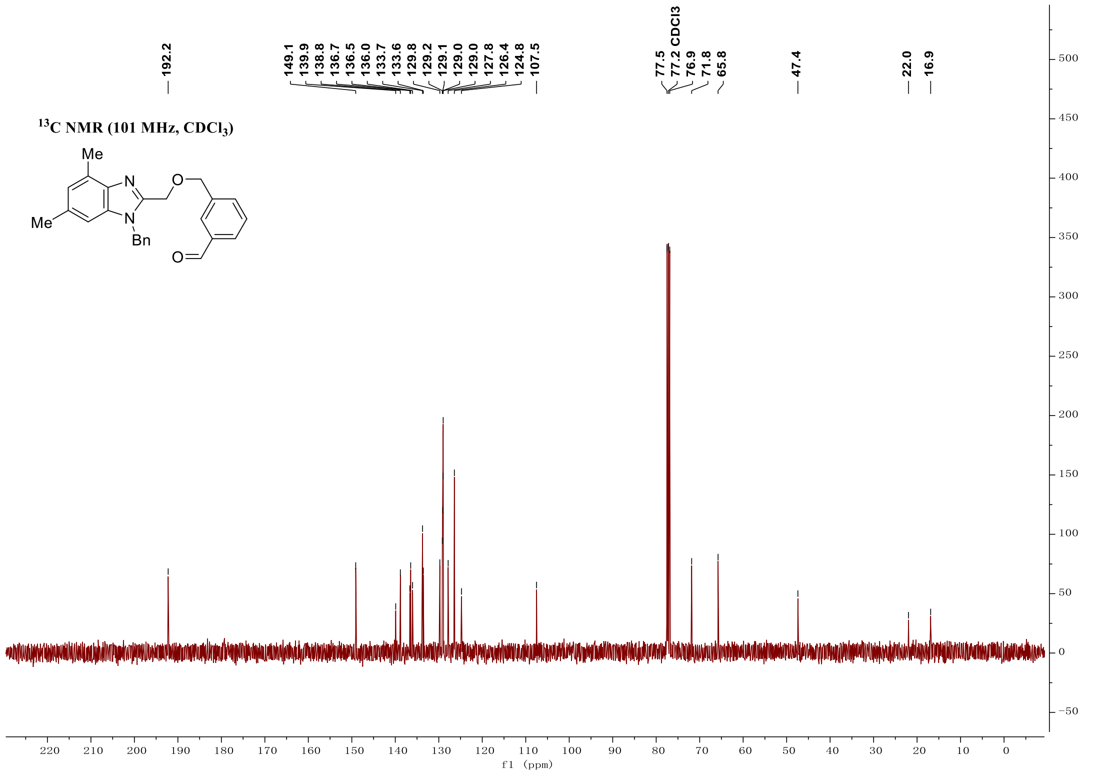

Supplementary Figure 40. **^{1}H NMR and ^{13}C NMR spectra of compound S44.**

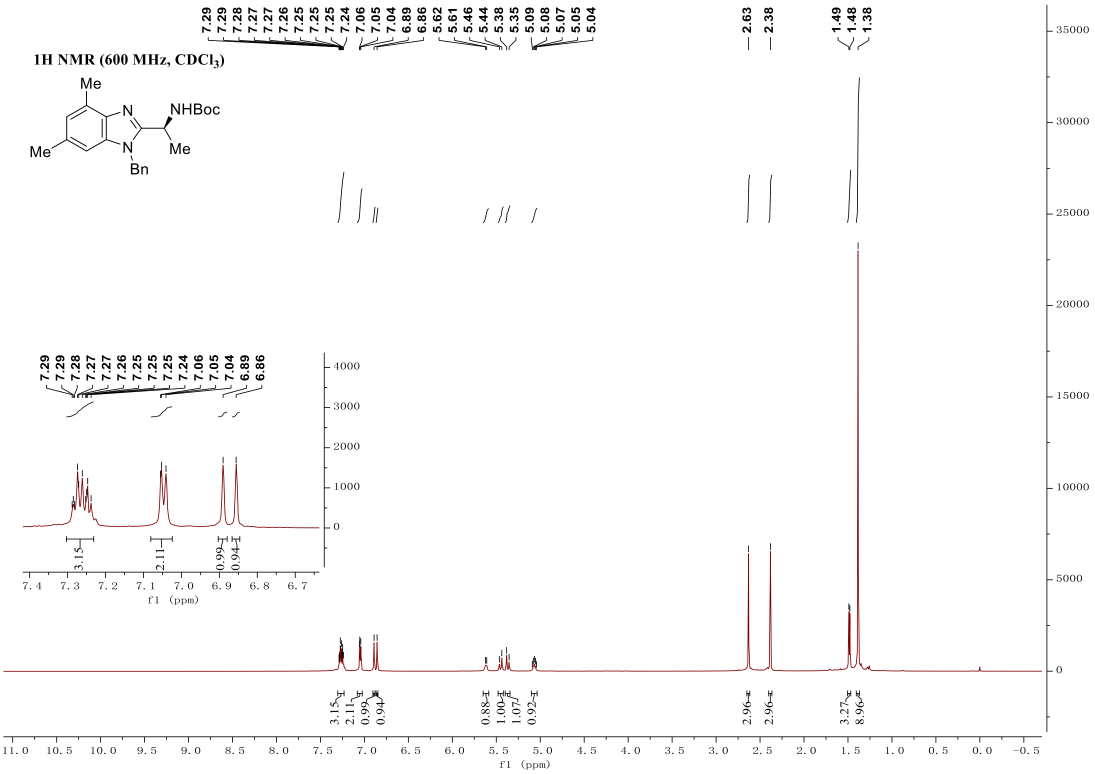

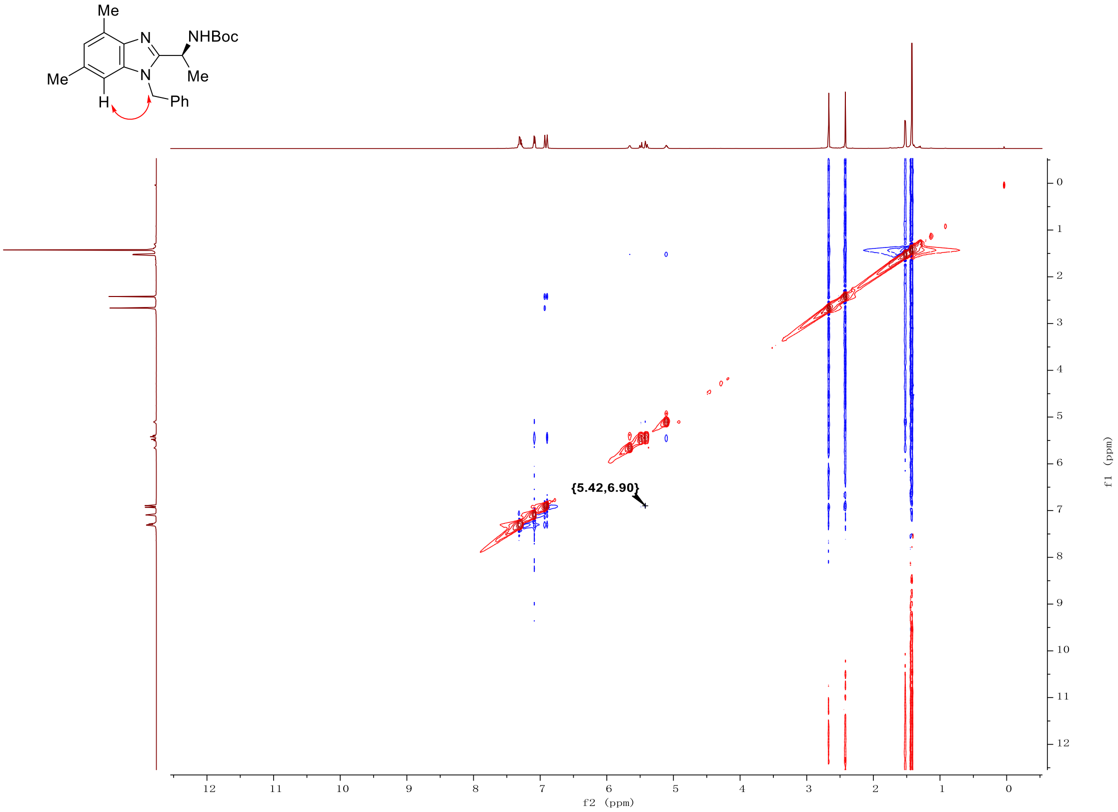

Supplementary Figure 41. ¹H NMR and 2D NOESY spectra of compound S46.

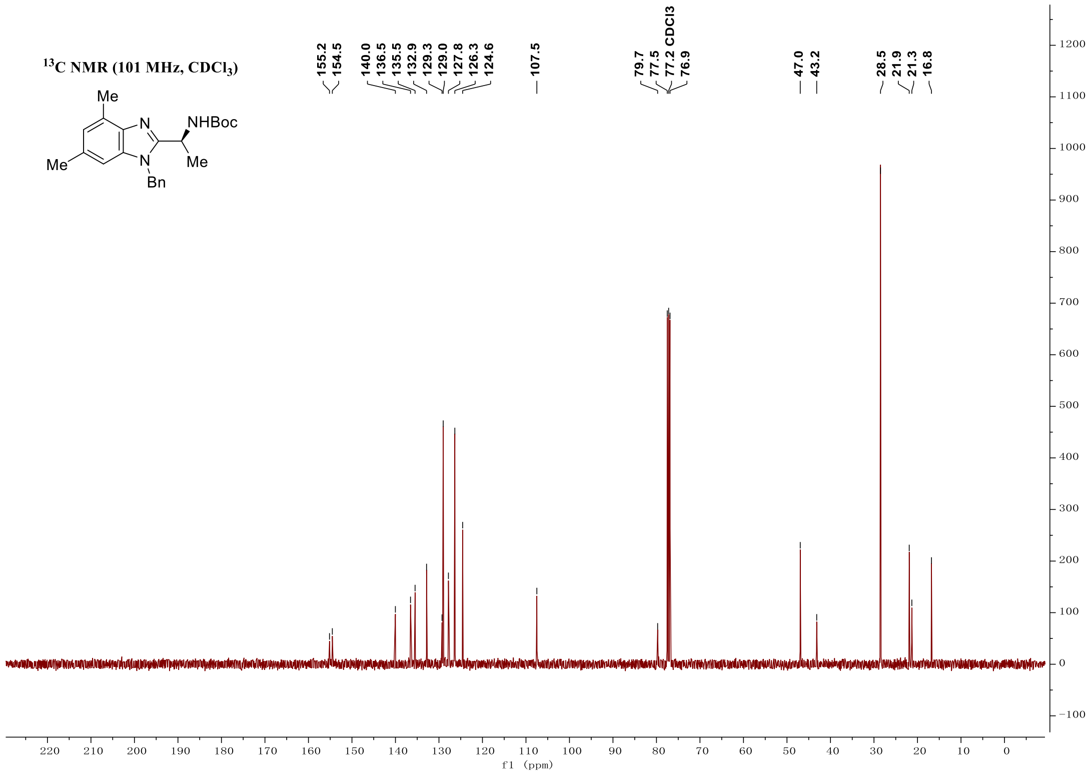

Supplementary Figure 42. ^{13}**C NMR spectrum of compound S46.**

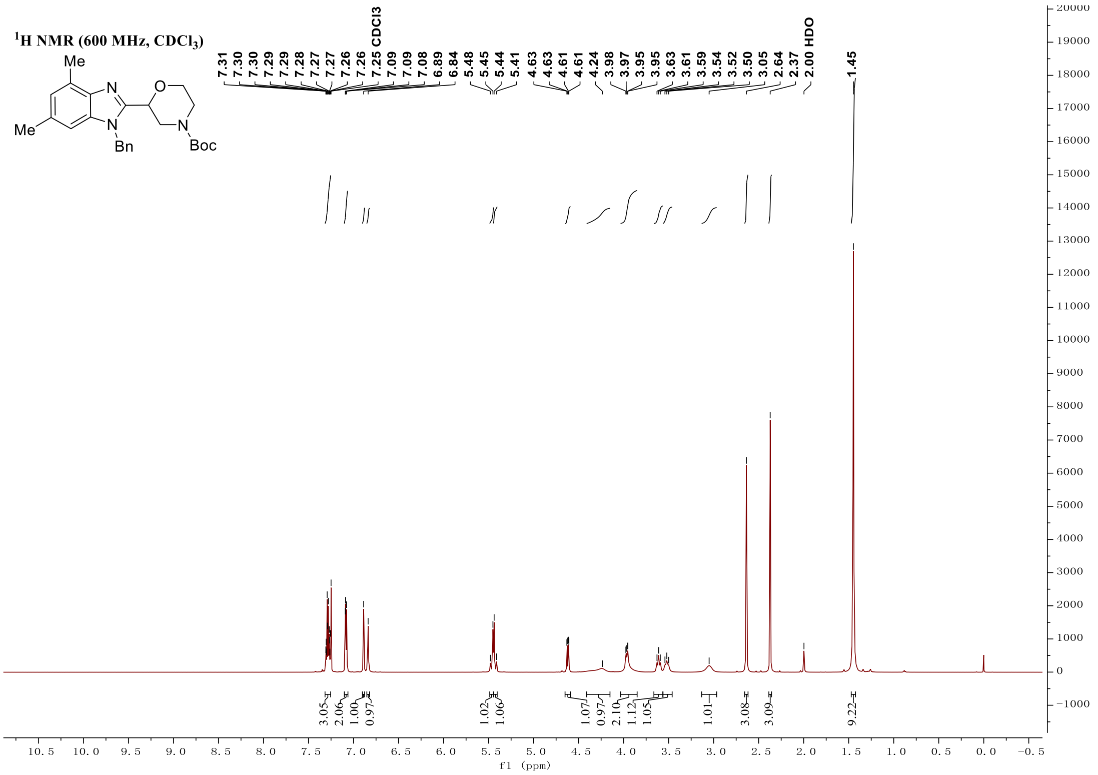

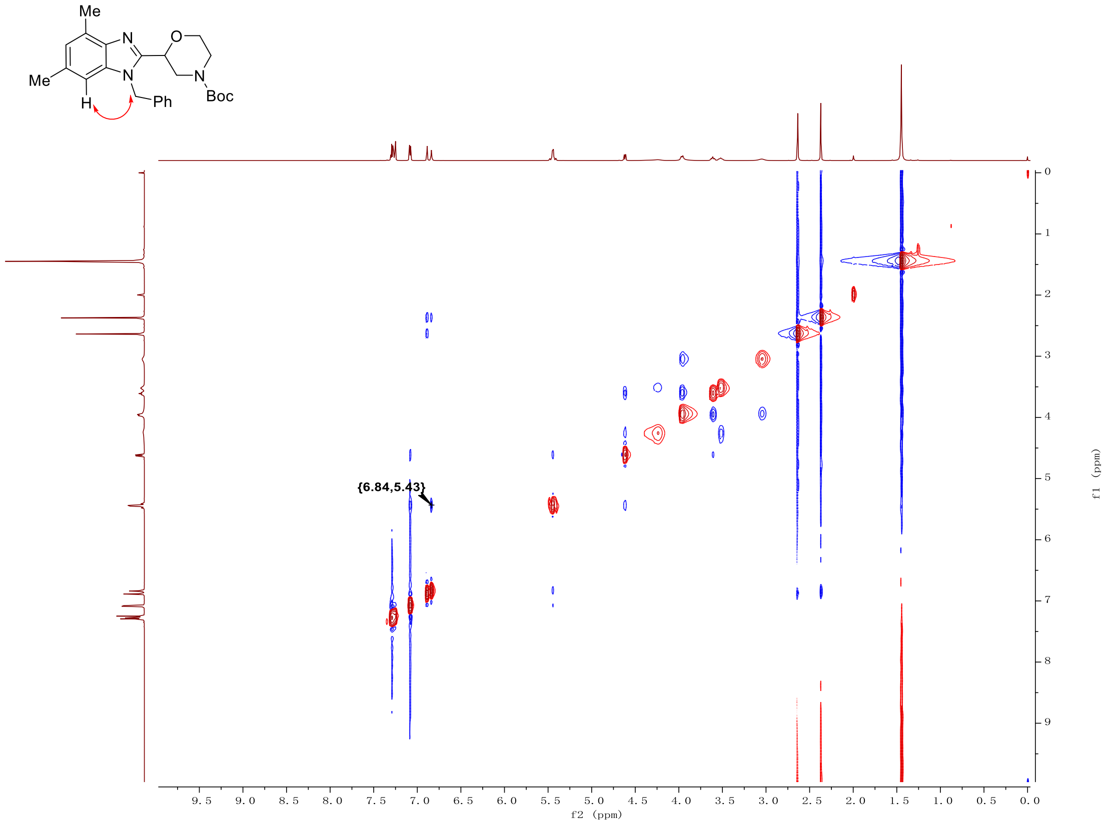

Supplementary Figure 43. ¹H NMR and 2D NOESY spectra of compound S49.

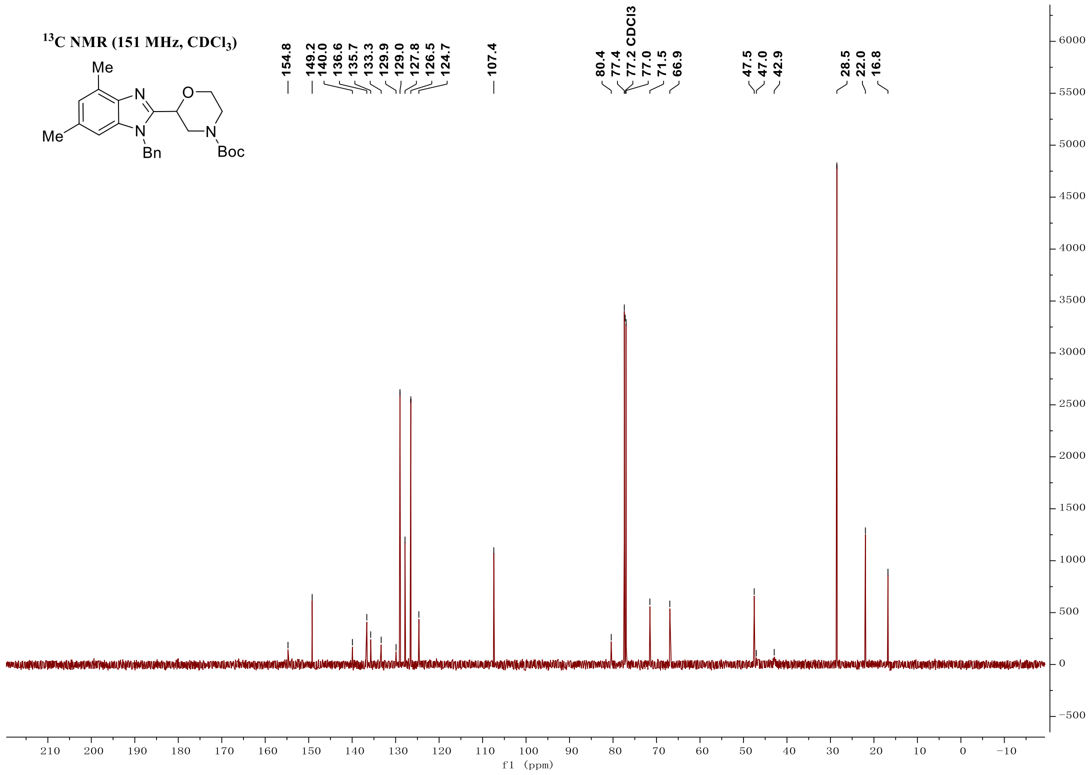

Supplementary Figure 44. ^{13}C NMR spectrum of compound S49.

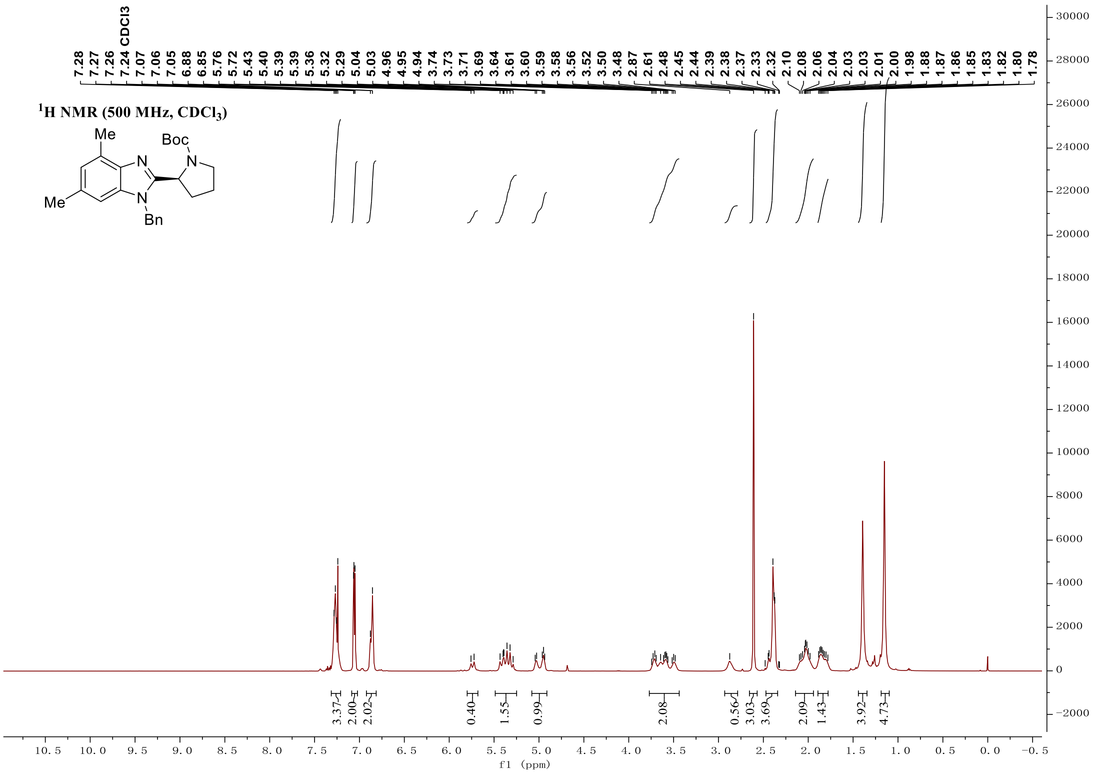

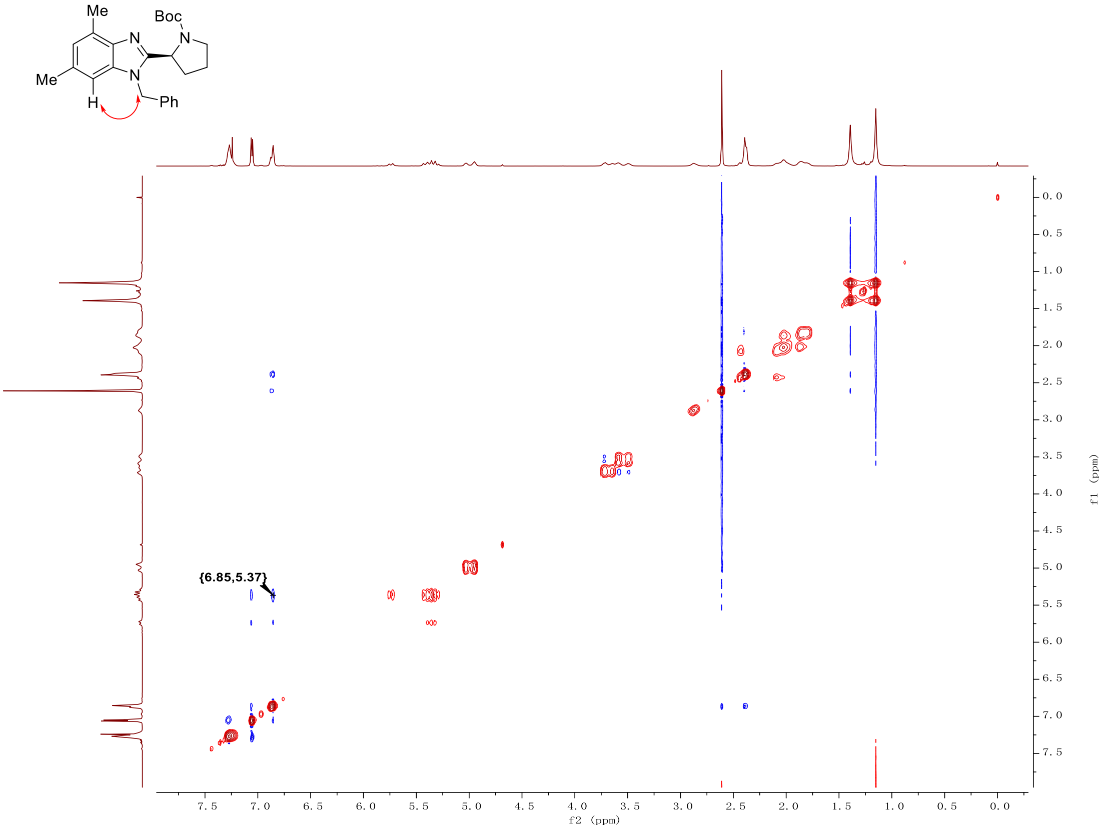

Supplementary Figure 45. ^{1}H NMR and 2D NOESY spectra of compound S51.

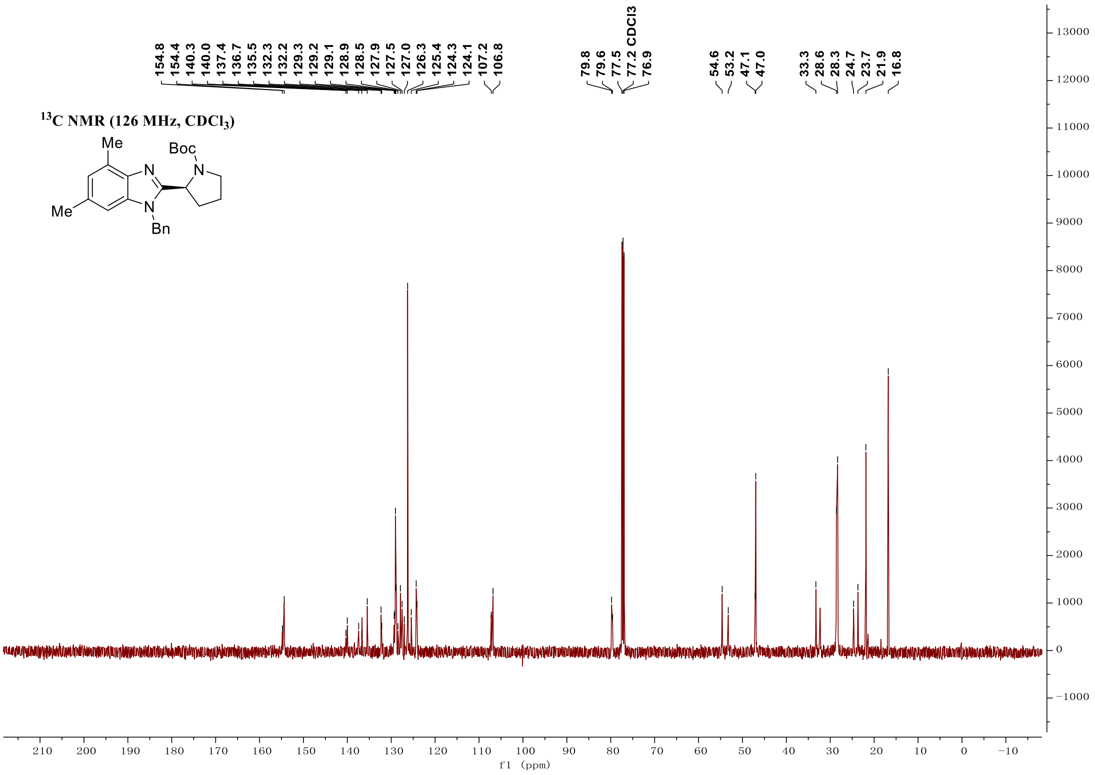

Supplementary Figure 46. ^{13}C NMR spectrum of compound S51.

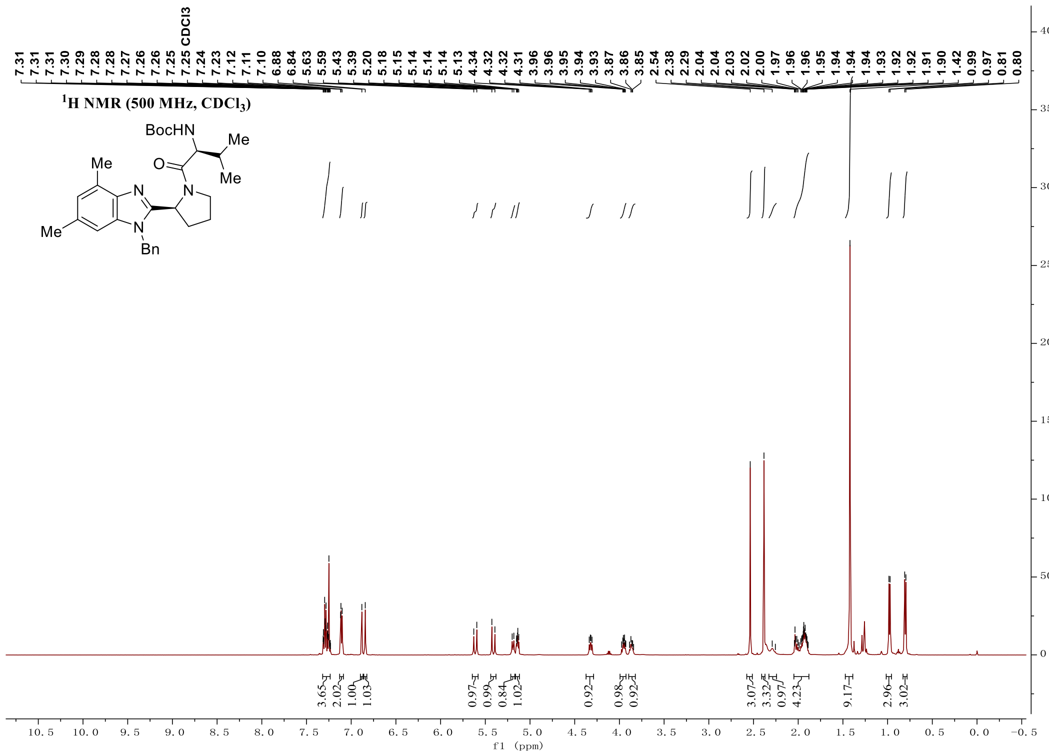

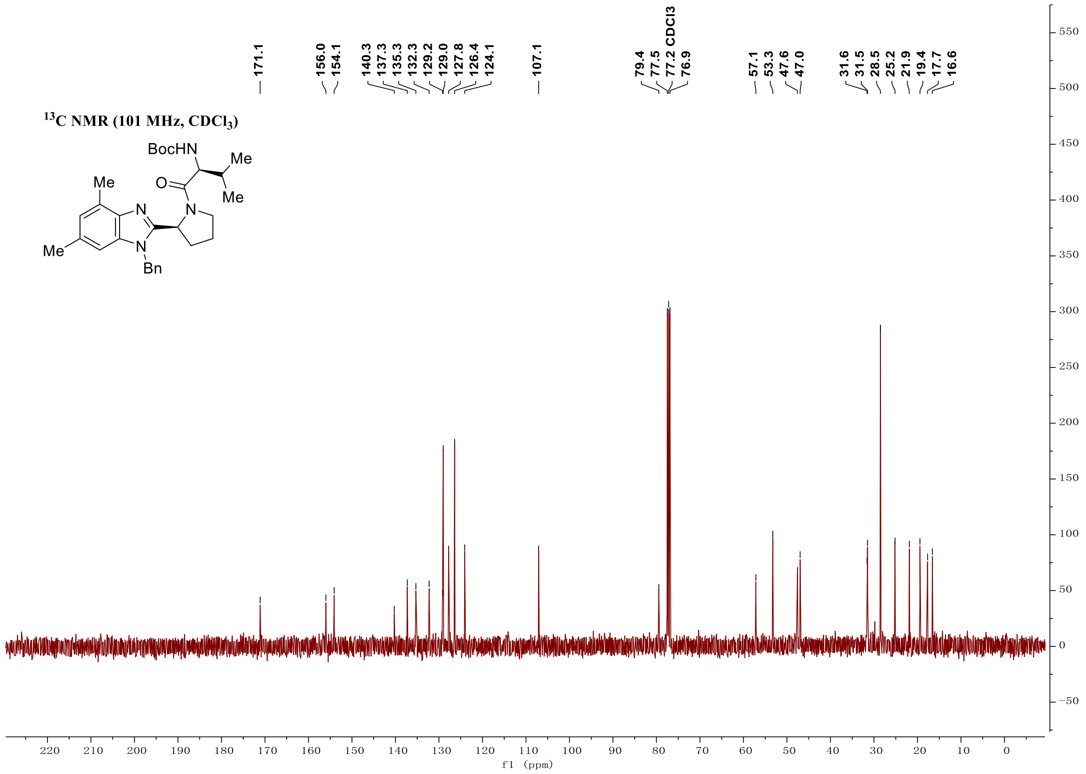

Supplementary Figure 47. ^{1}H NMR and ^{13}C NMR spectra of compound S53.

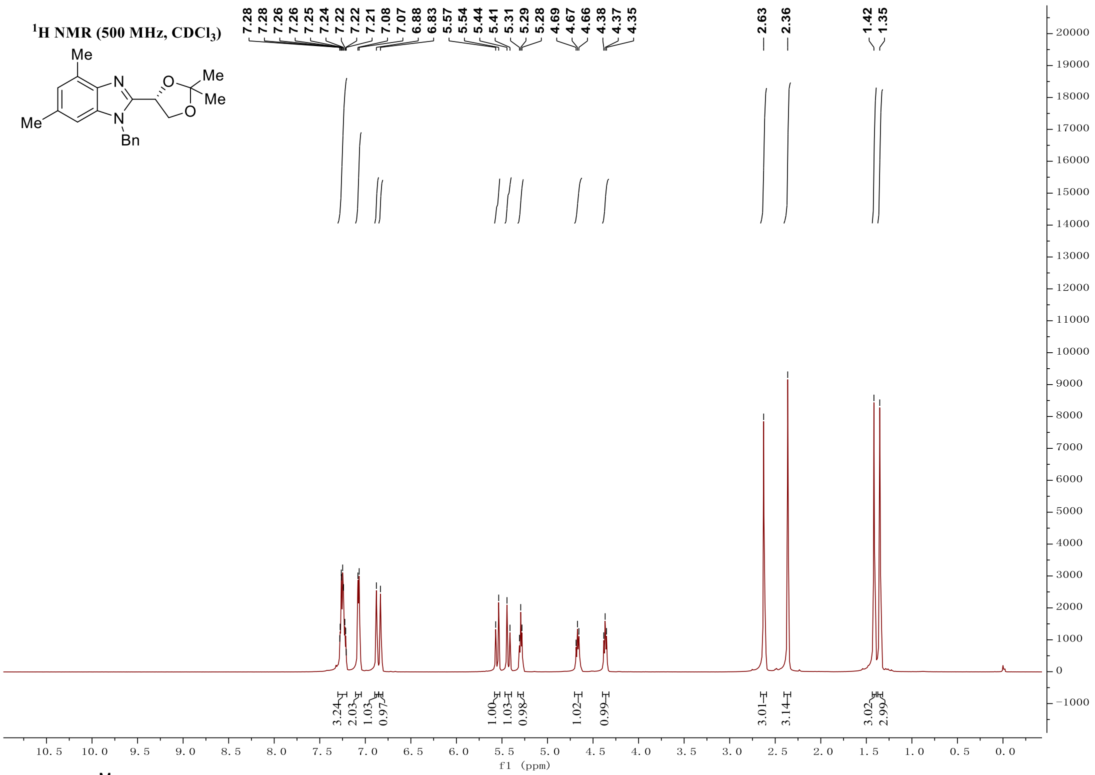

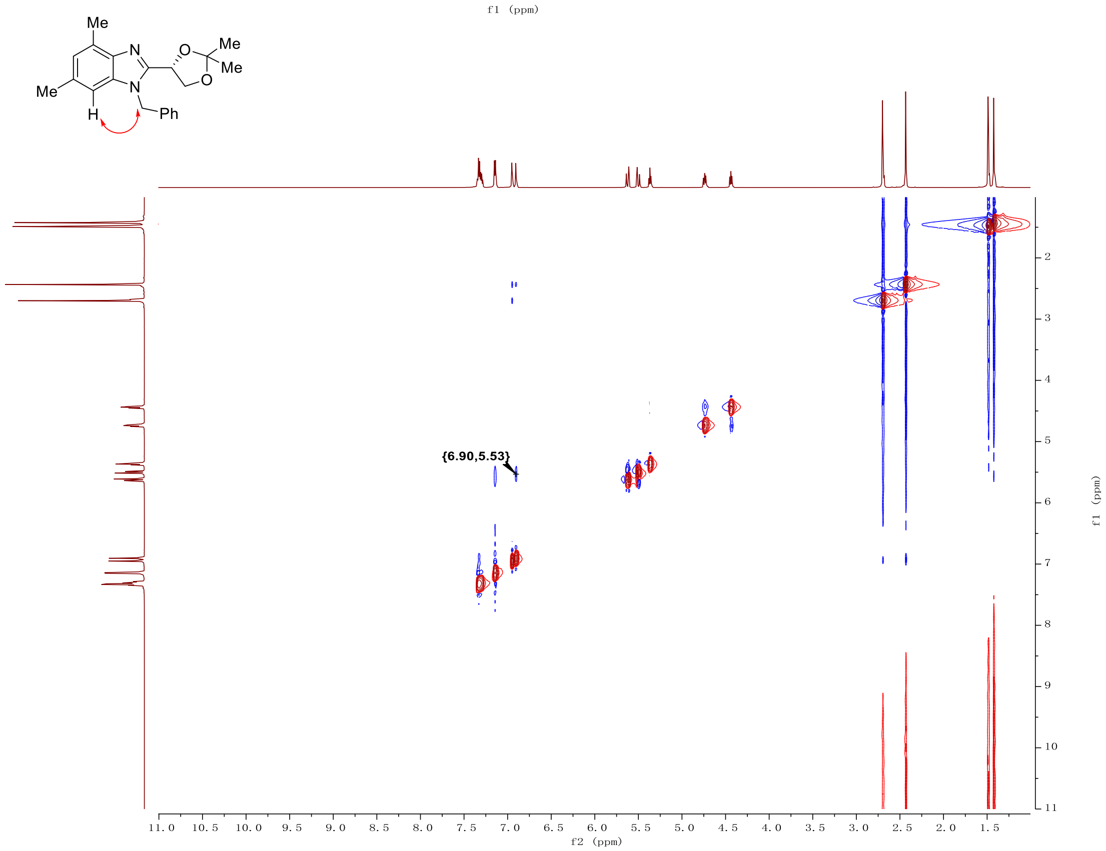

Supplementary Figure 48. ^{1}H NMR and 2D NOESY spectra of compound S55.

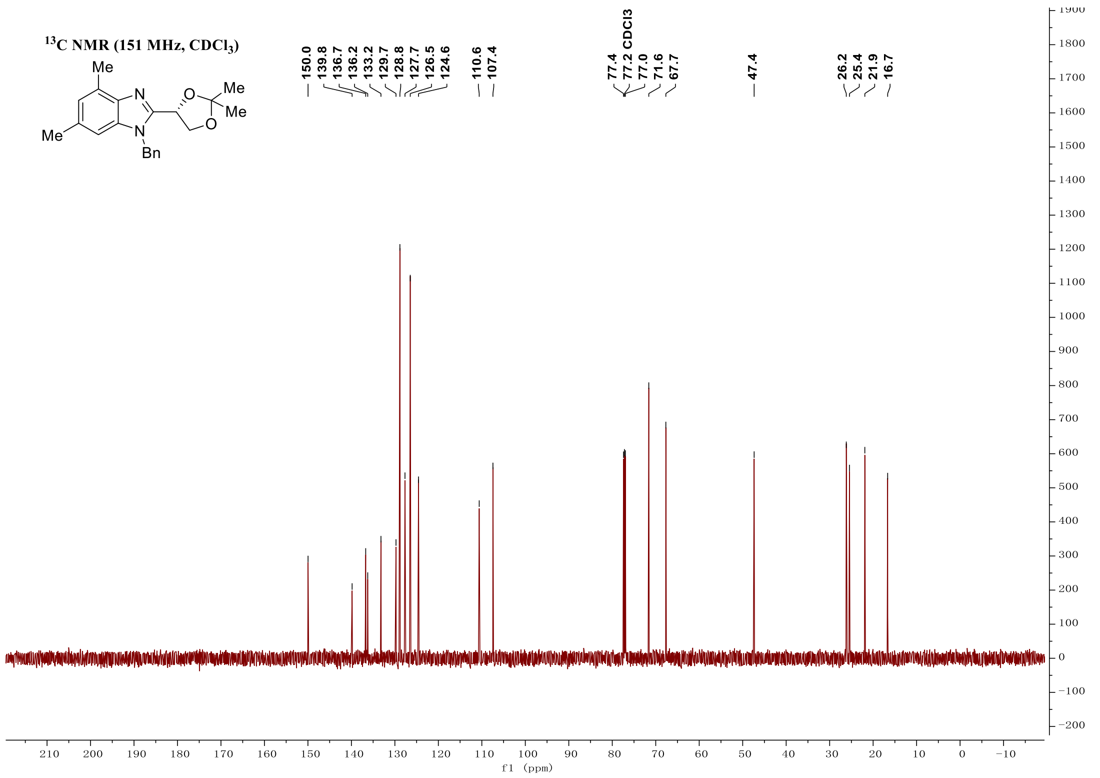

Supplementary Figure 49. ^{13}C NMR spectrum of compound S55.

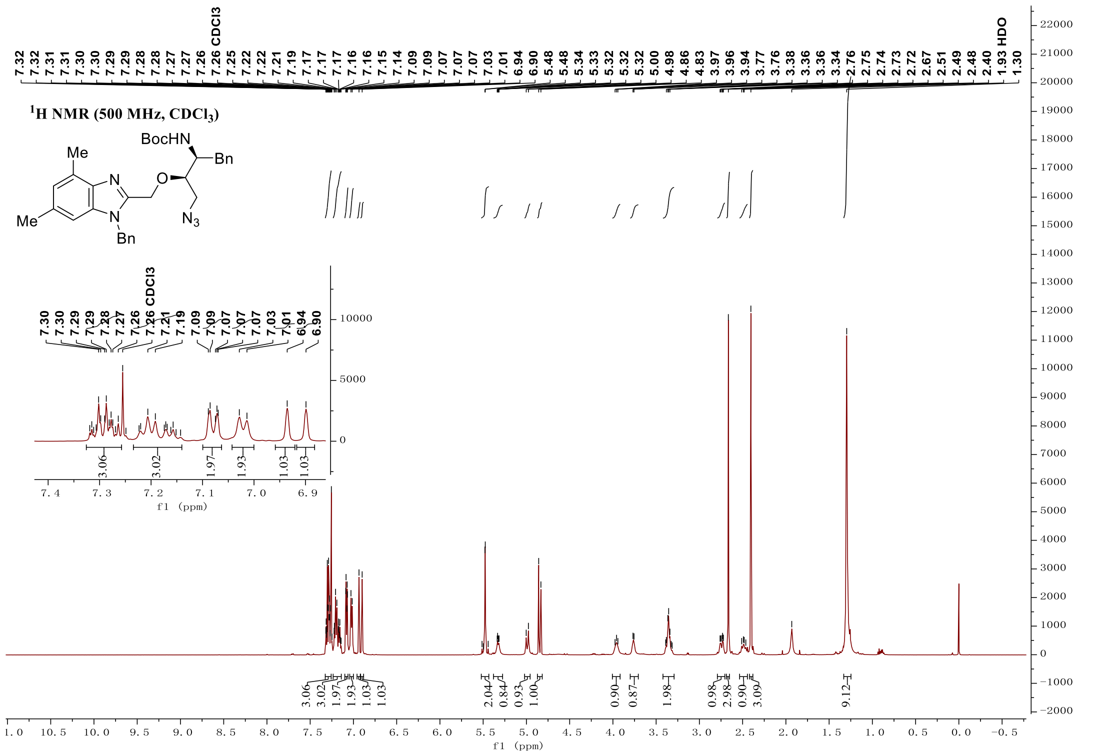

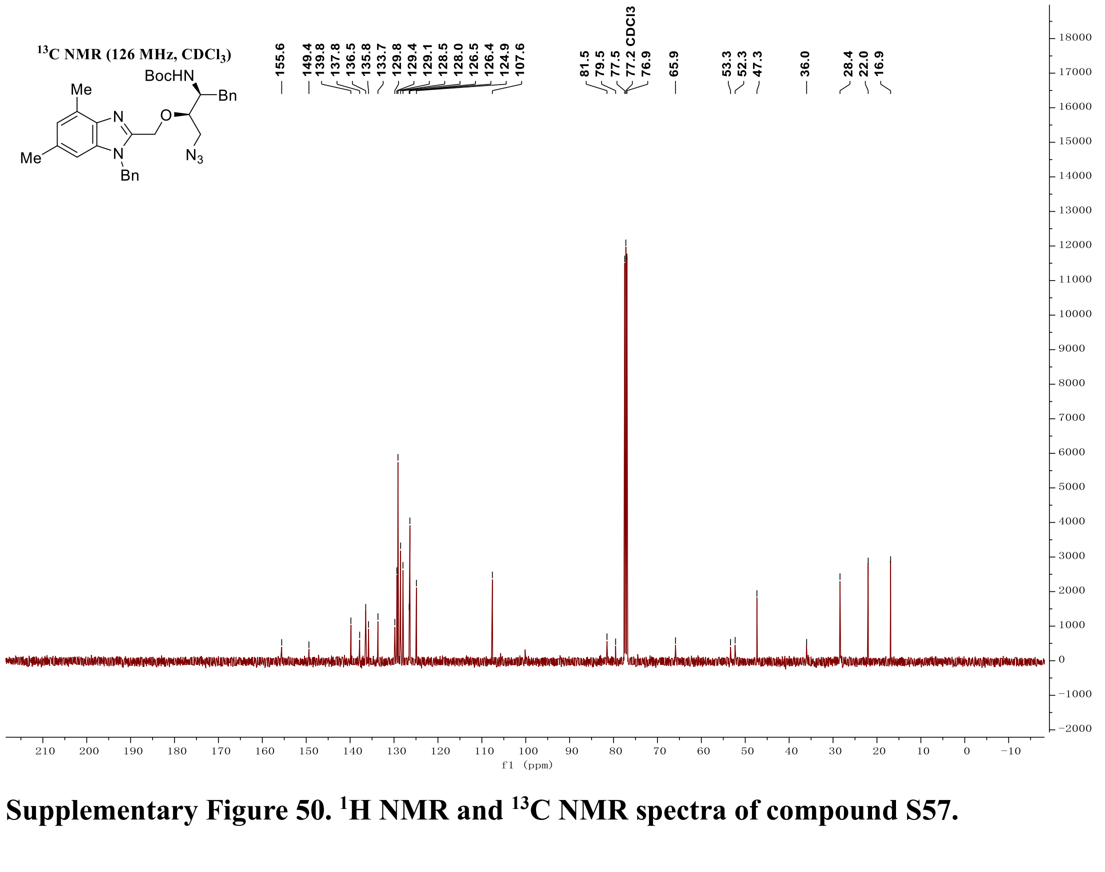

Supplementary Figure 50. ^{1}H NMR and ^{13}C NMR spectra of compound S57.

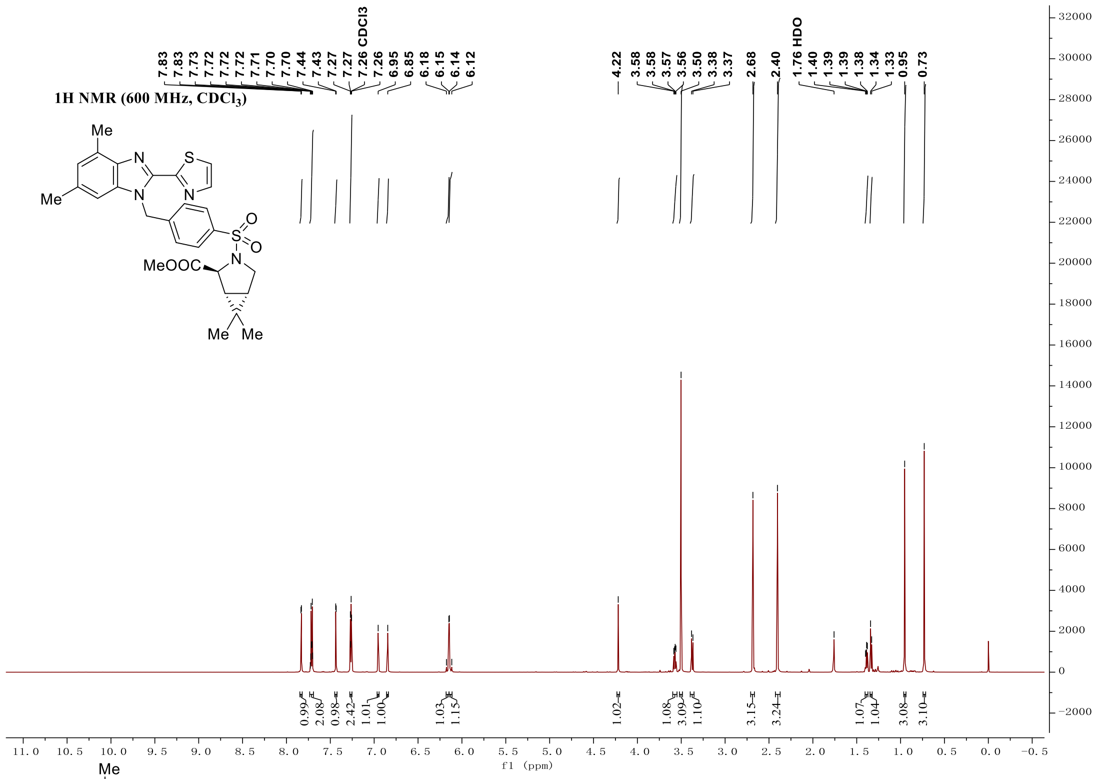

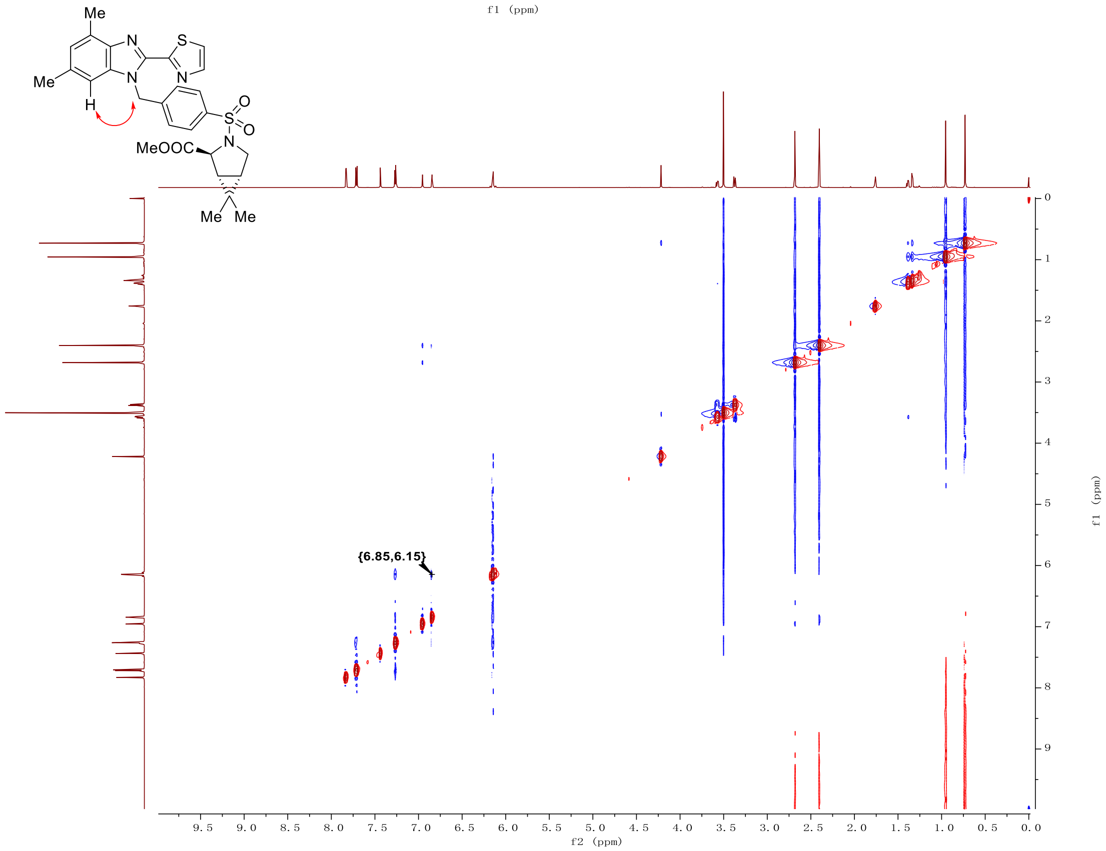

Supplementary Figure 51. ^{1}H NMR and 2D NOESY spectra of compound S59.

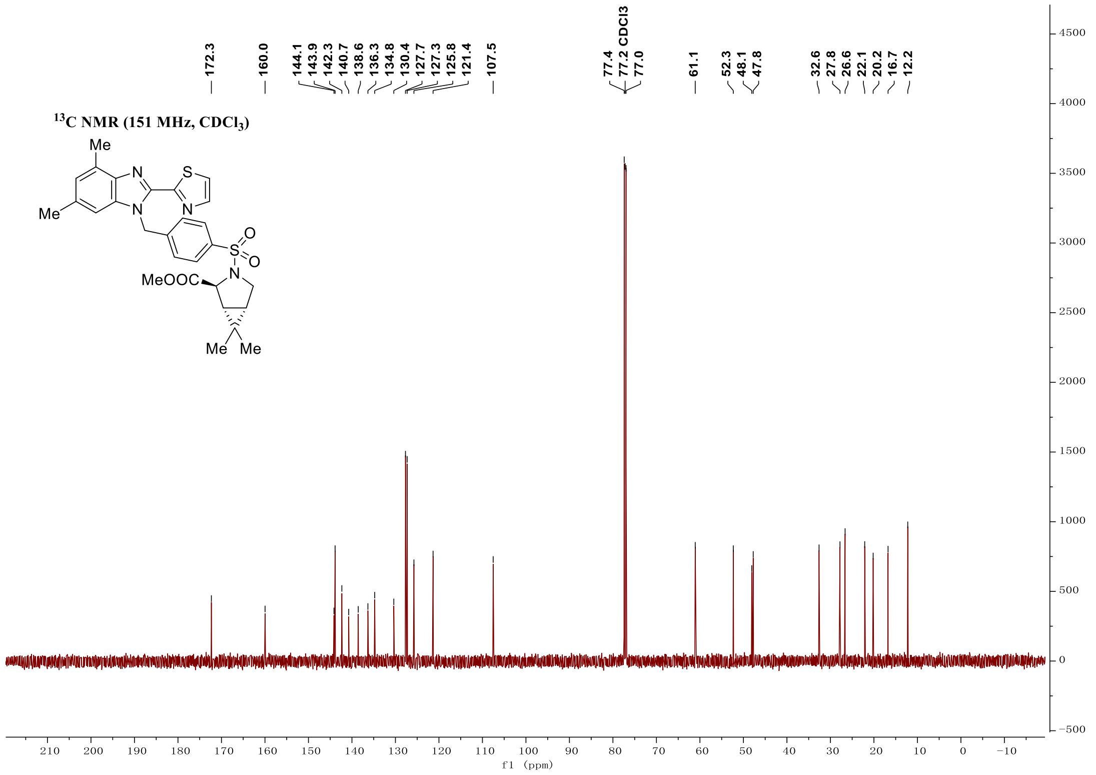

Supplementary Figure 52. ^{13}C NMR spectrum of compound S59.

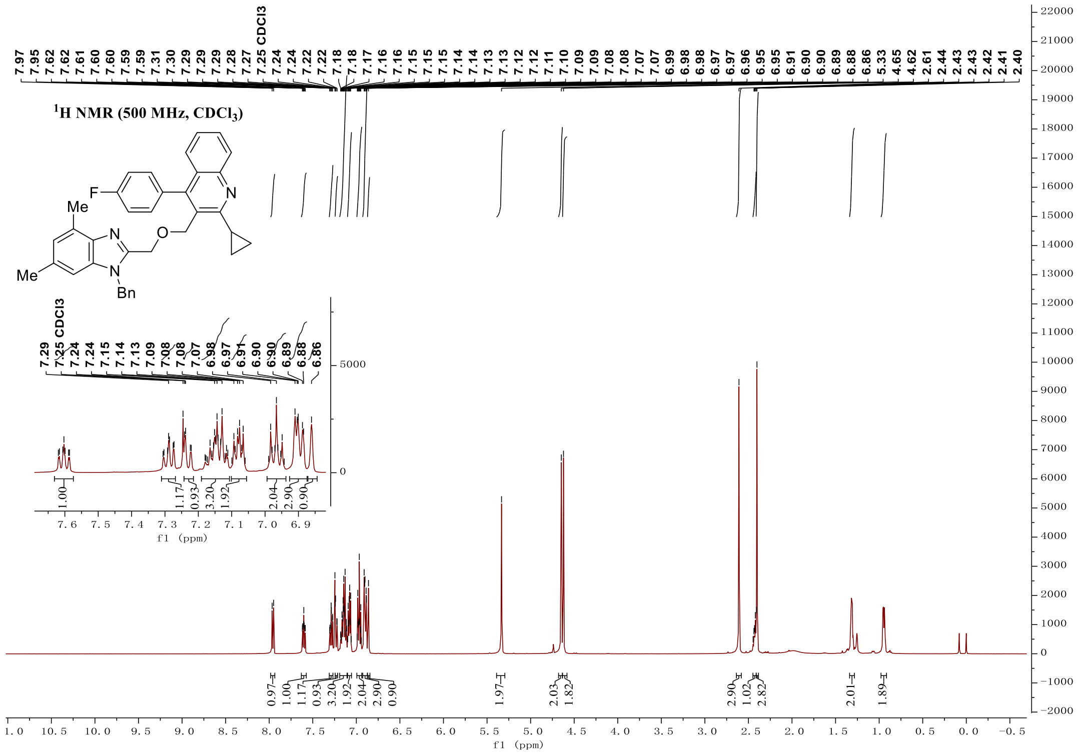

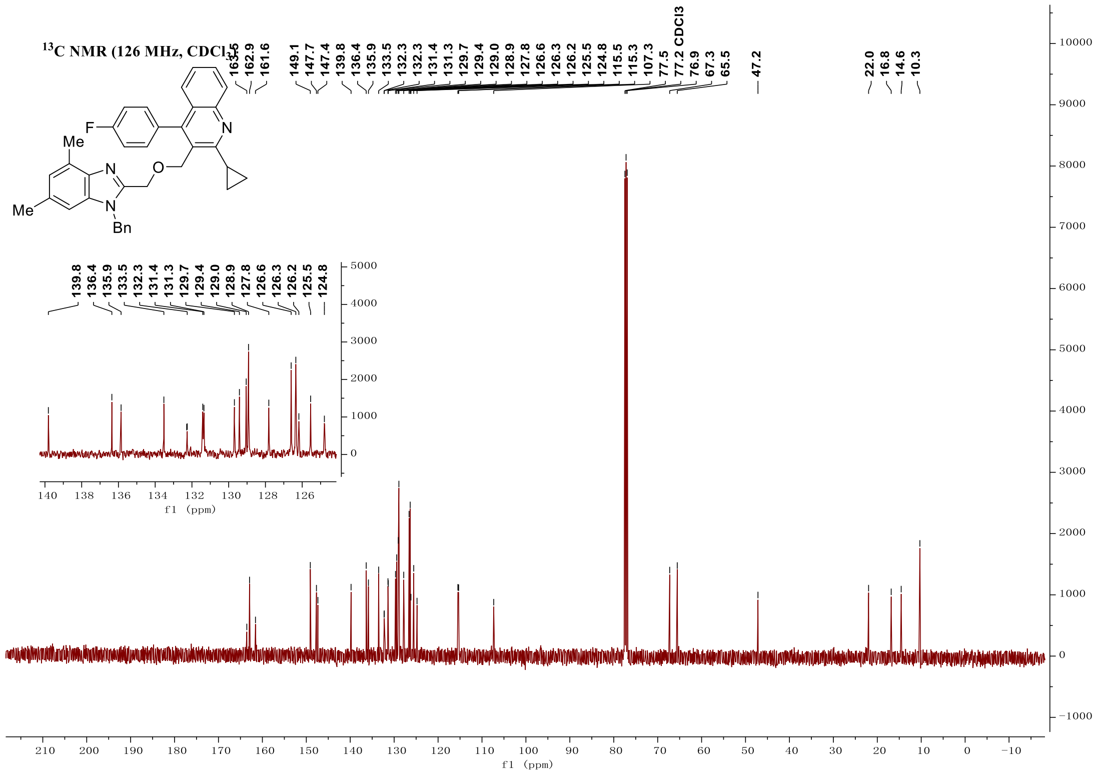

Supplementary Figure 53. ^{1}H NMR and ^{13}C NMR spectra of compound S60.

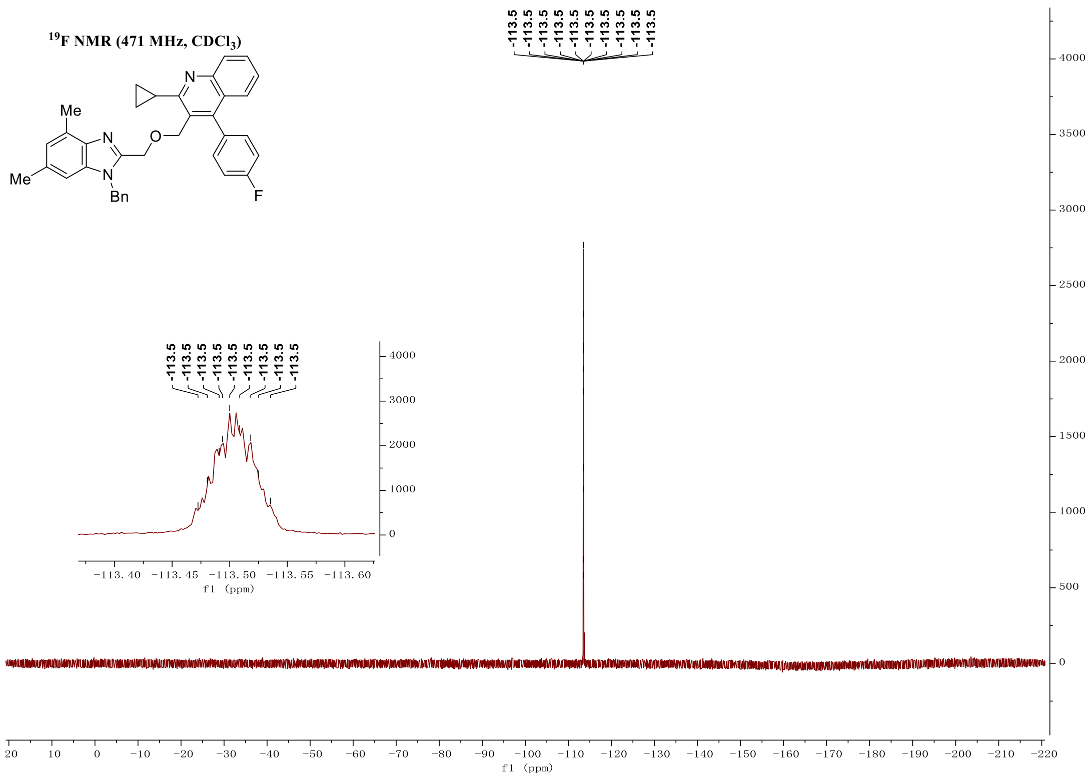

Supplementary Figure 54. ^{19}F NMR spectra of compound S60.

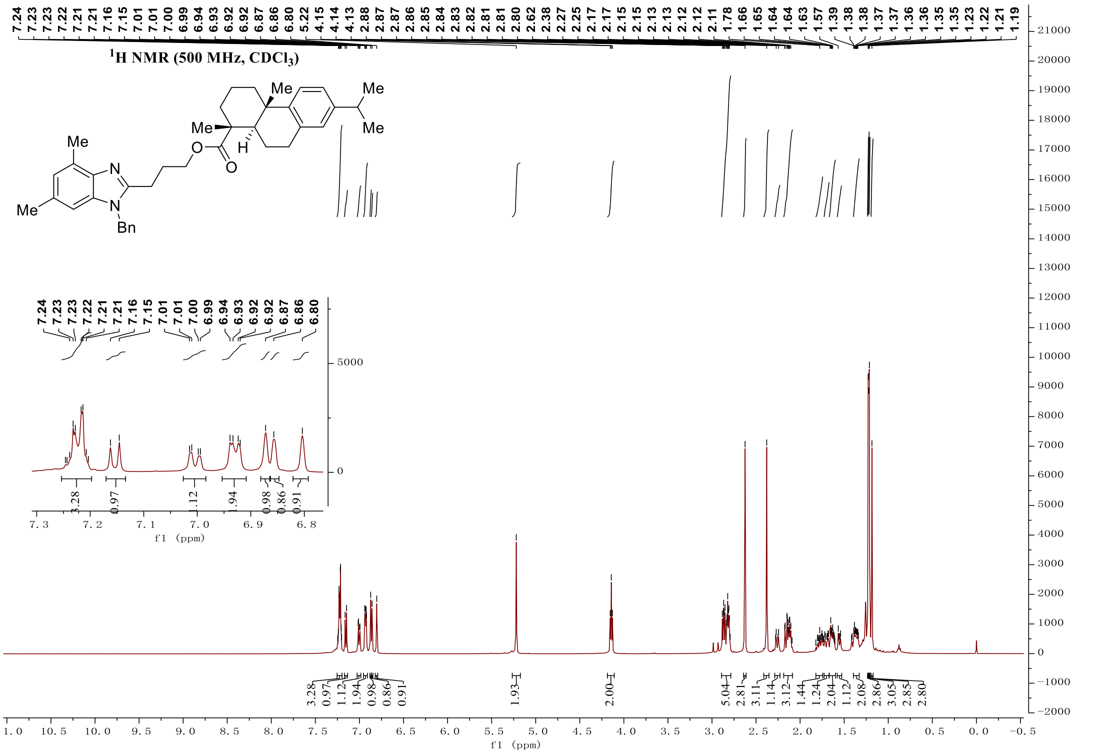

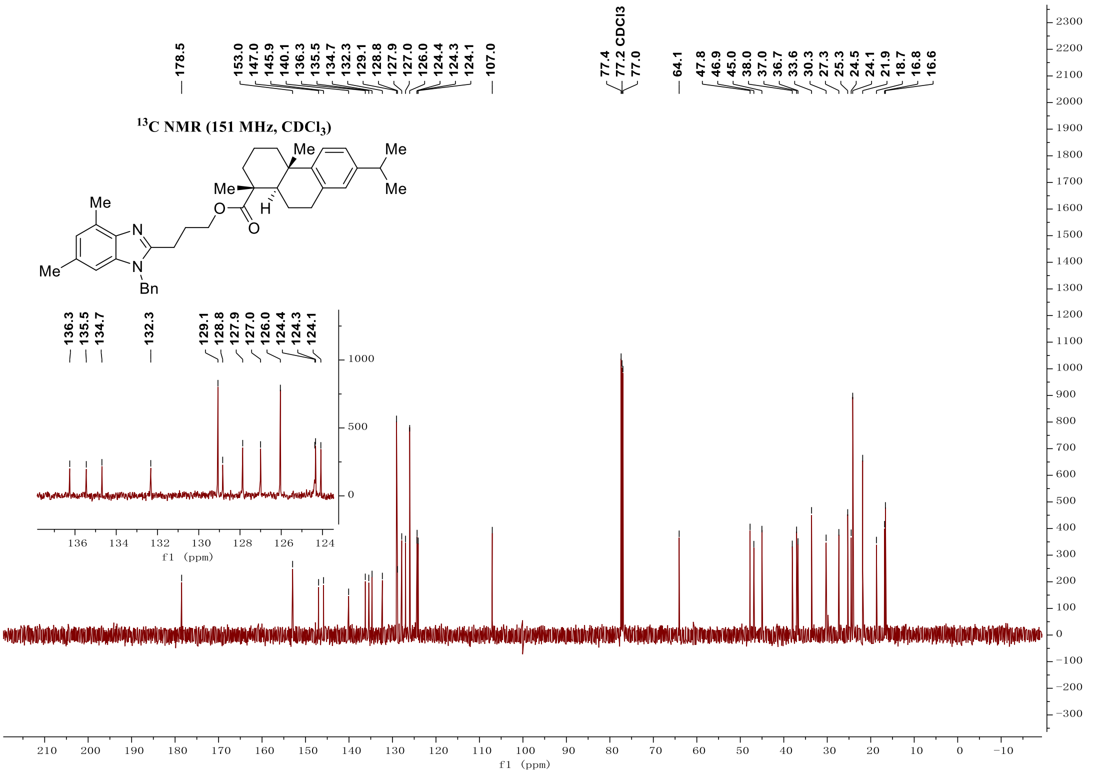

Supplementary Figure 55. ^{1}H NMR and ^{13}C NMR spectra of compound S62.

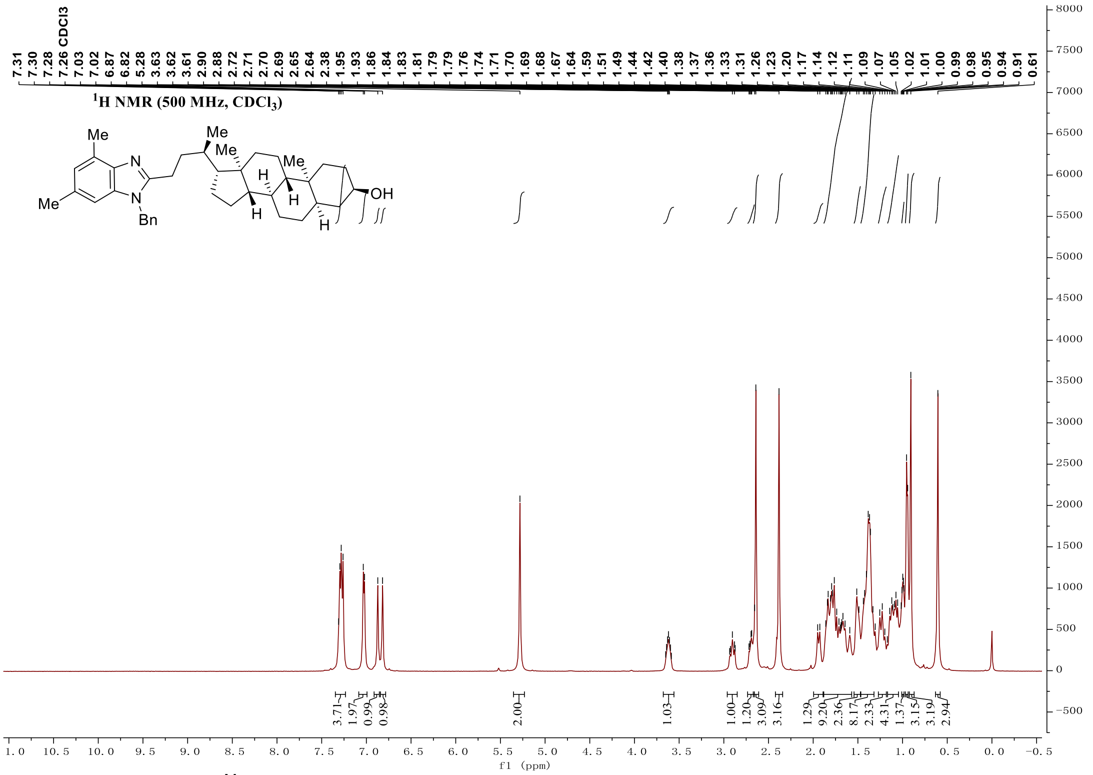

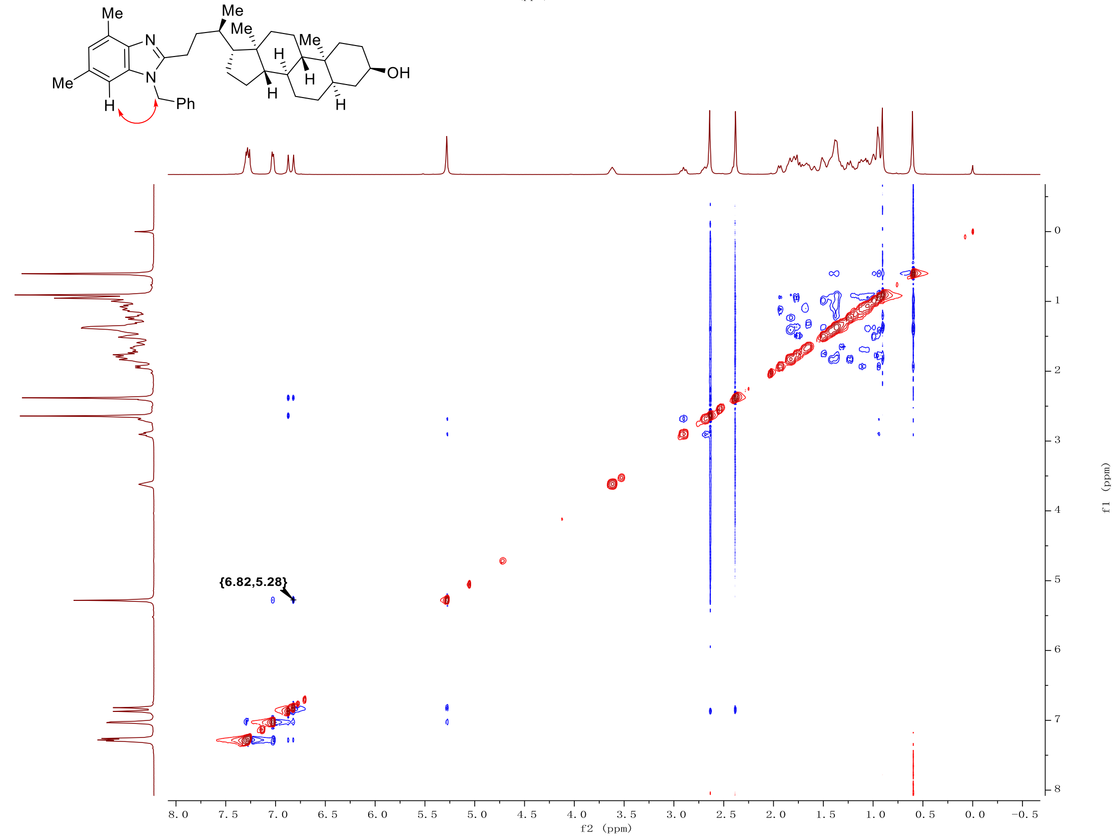

Supplementary Figure 56. ¹H NMR and 2D NOESY spectra of compound S65.

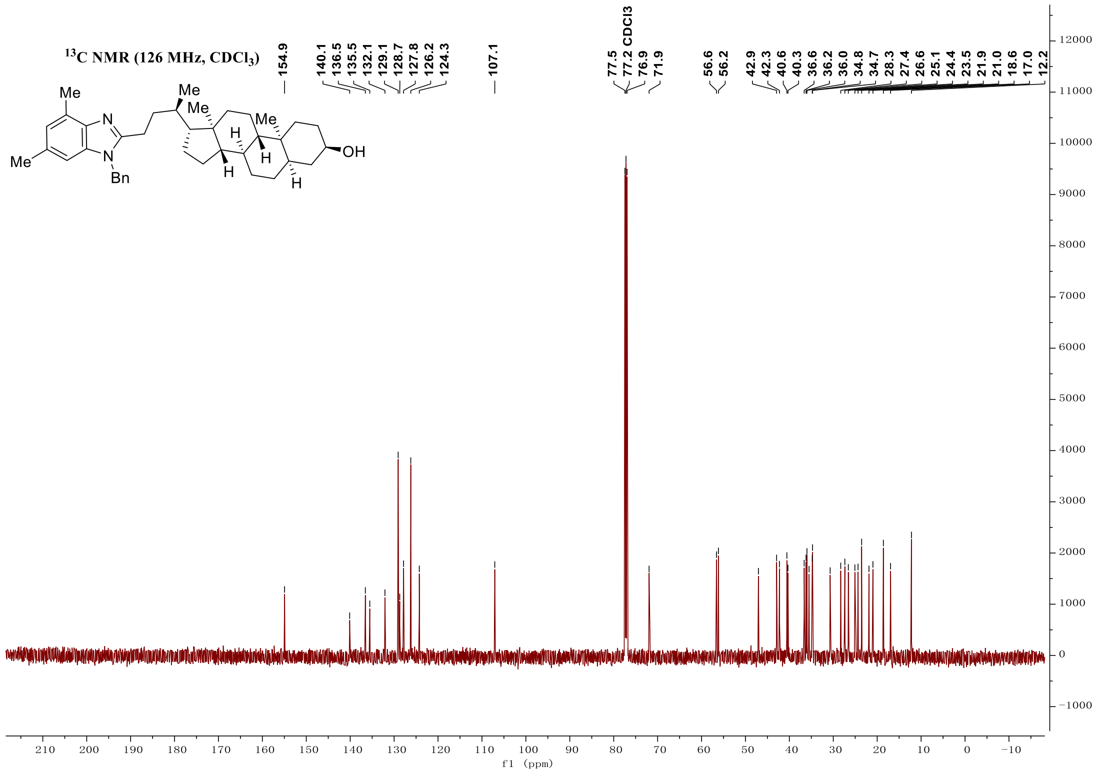

Supplementary Figure 57. ^{13}C NMR spectrum of compound S65.

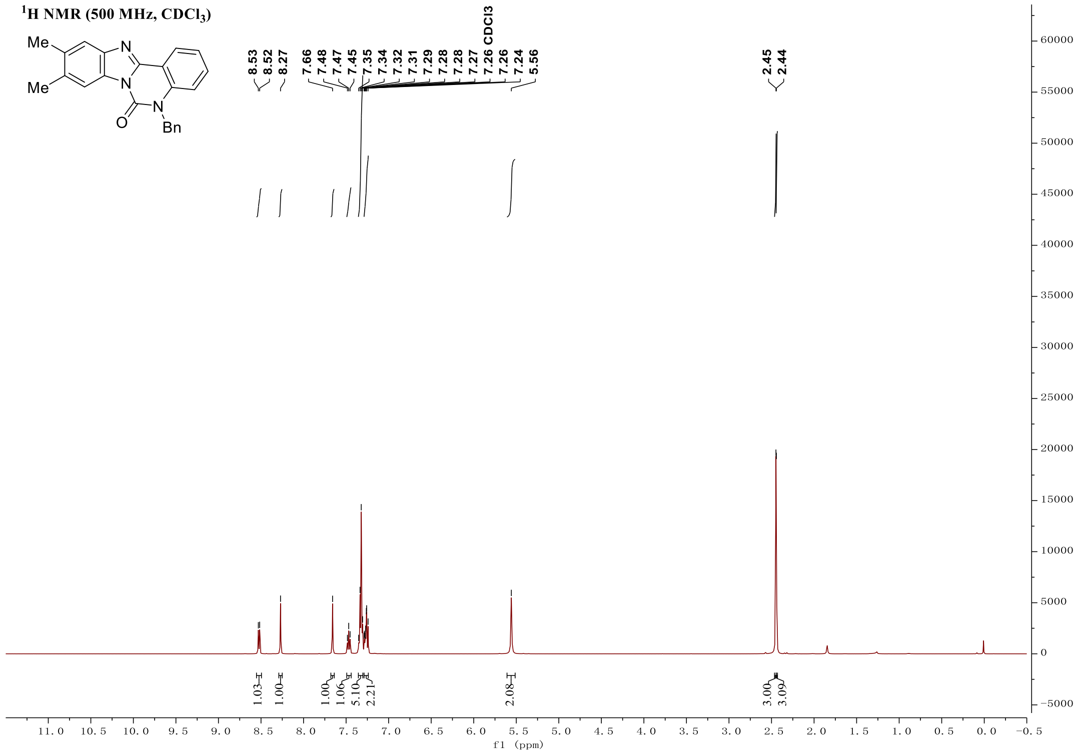

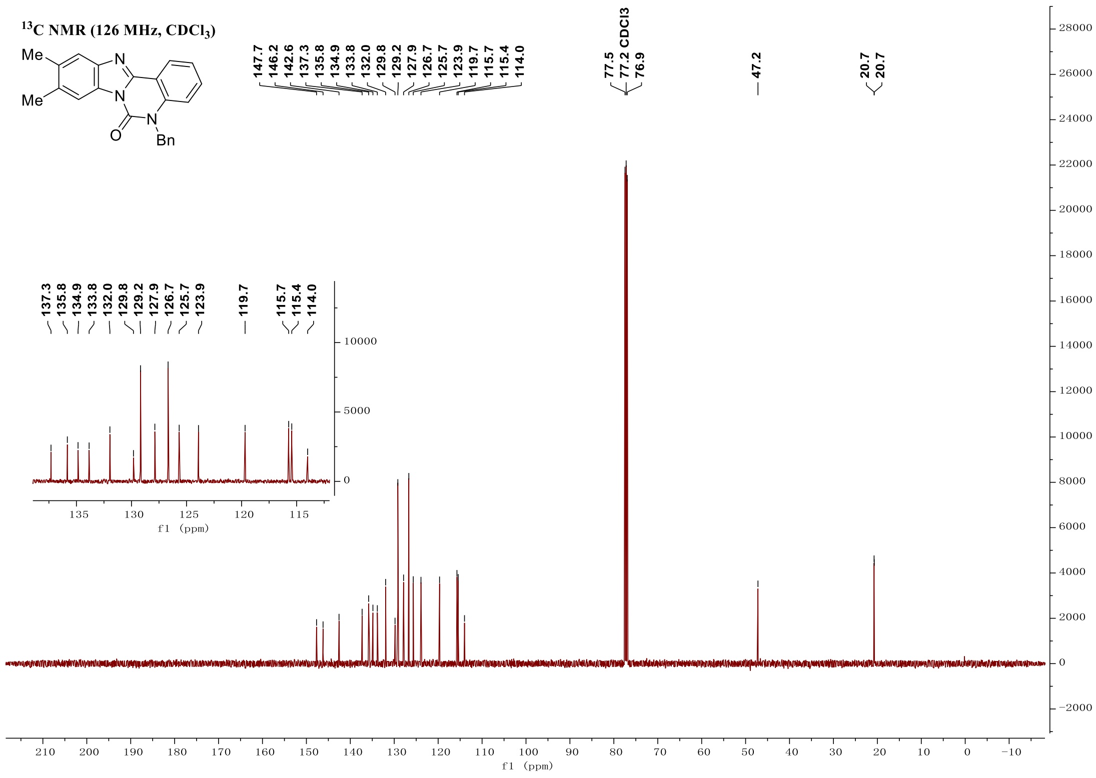

Supplementary Figure 58. **^{1}H NMR and ^{13}C NMR spectra of compound S67.**

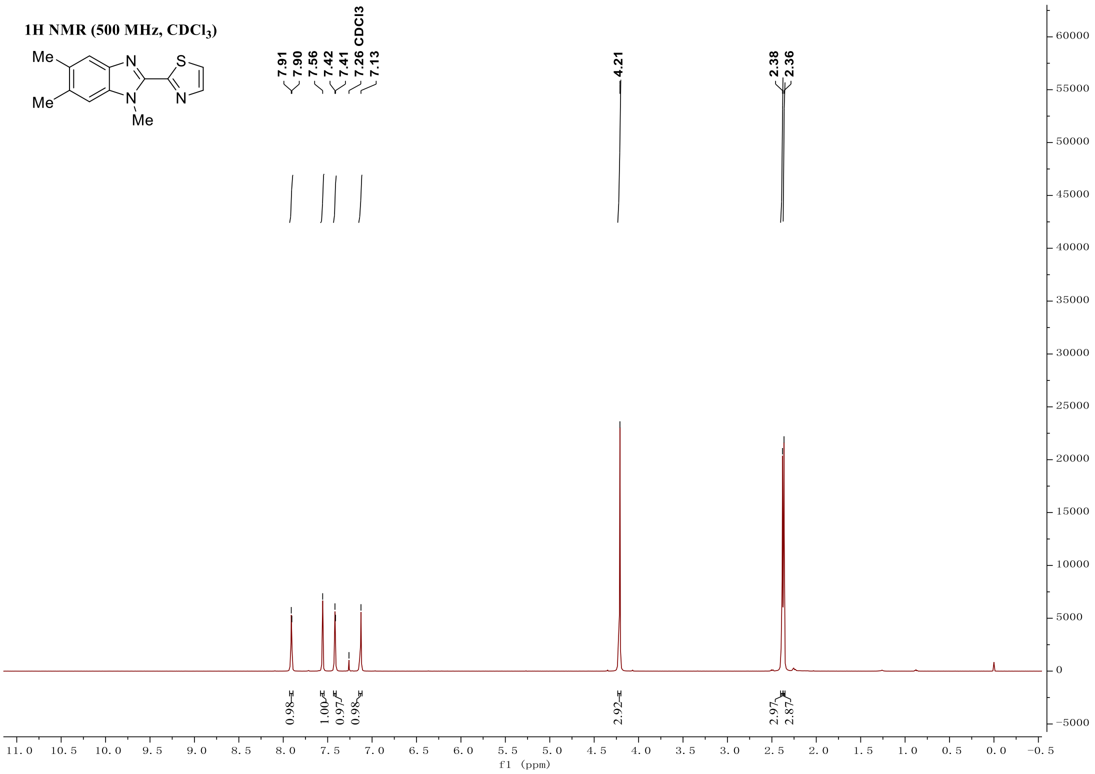

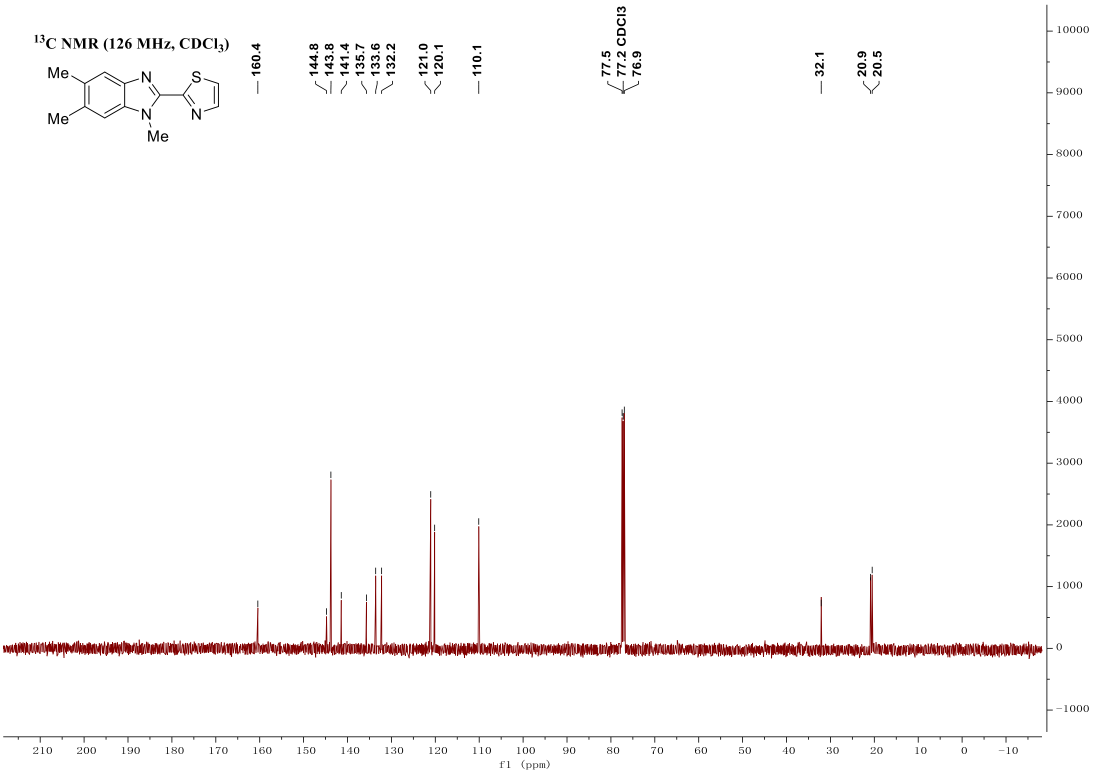

Supplementary Figure 59. ^{1}H NMR and ^{13}C NMR spectra of compound S69.

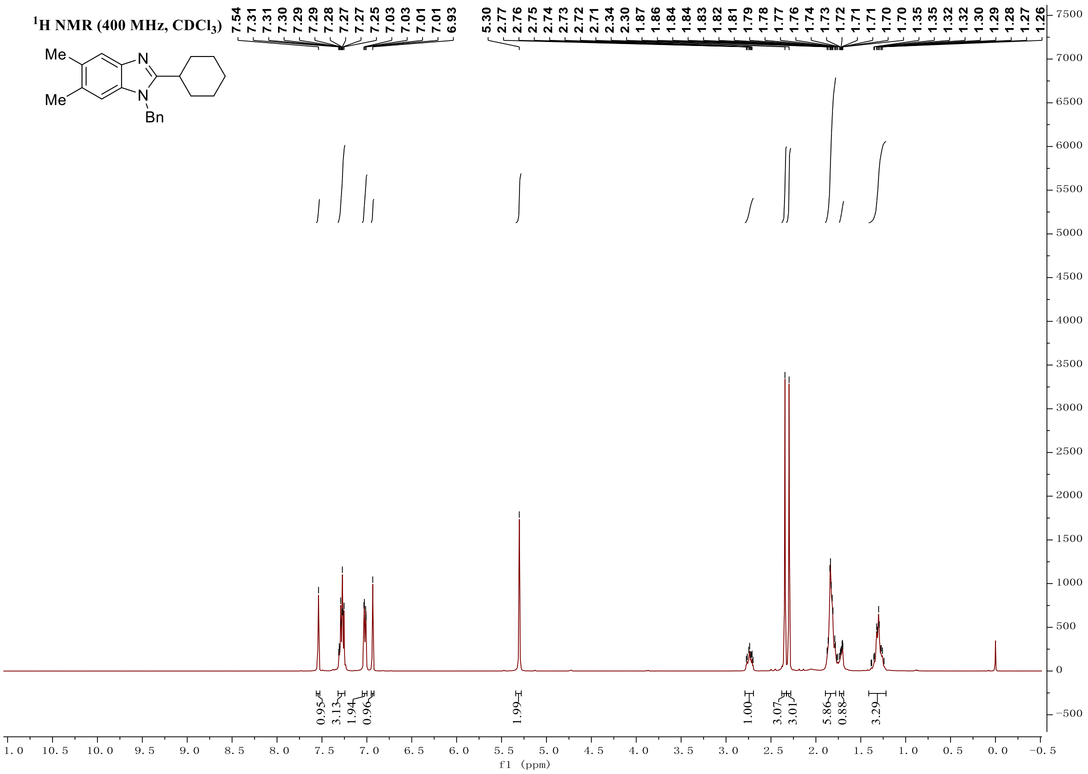

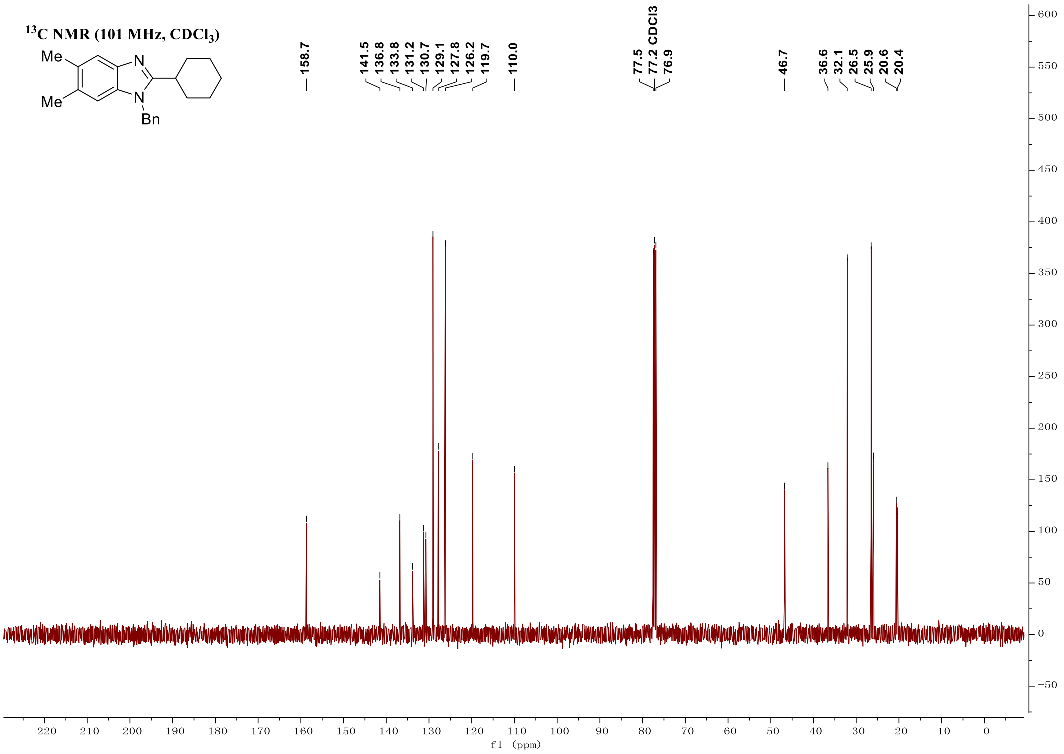

Supplementary Figure 60. ^{1}H NMR and ^{13}C NMR spectra of compound S72.

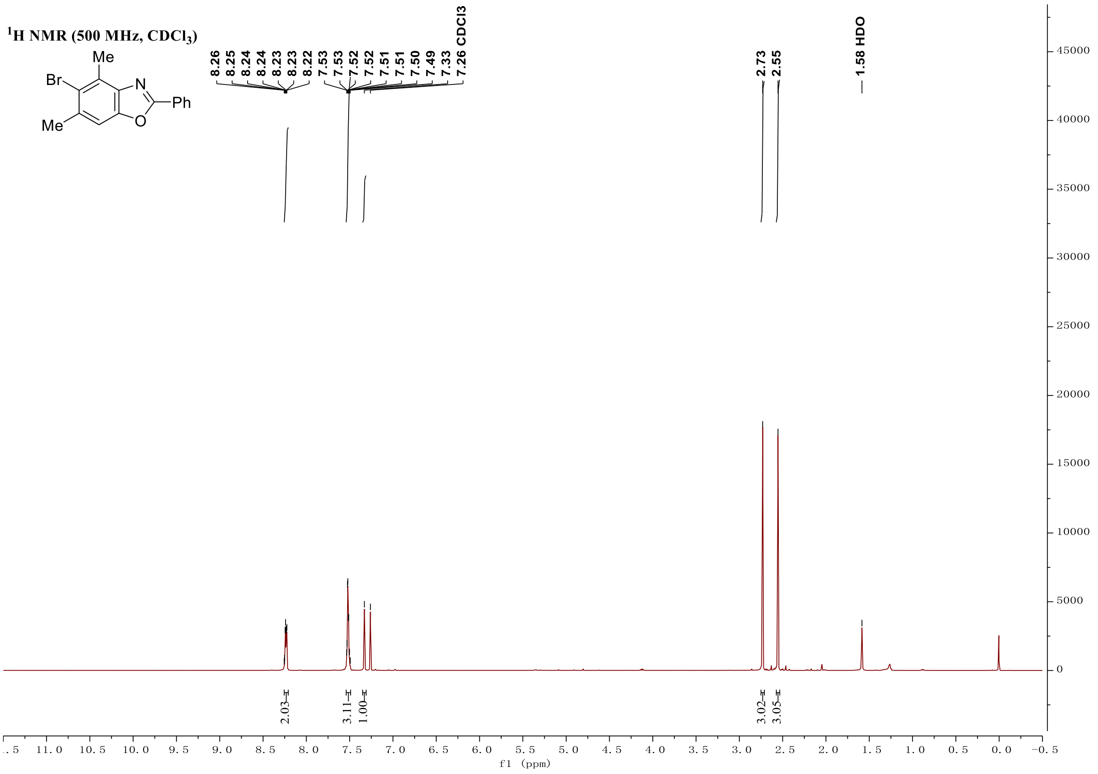

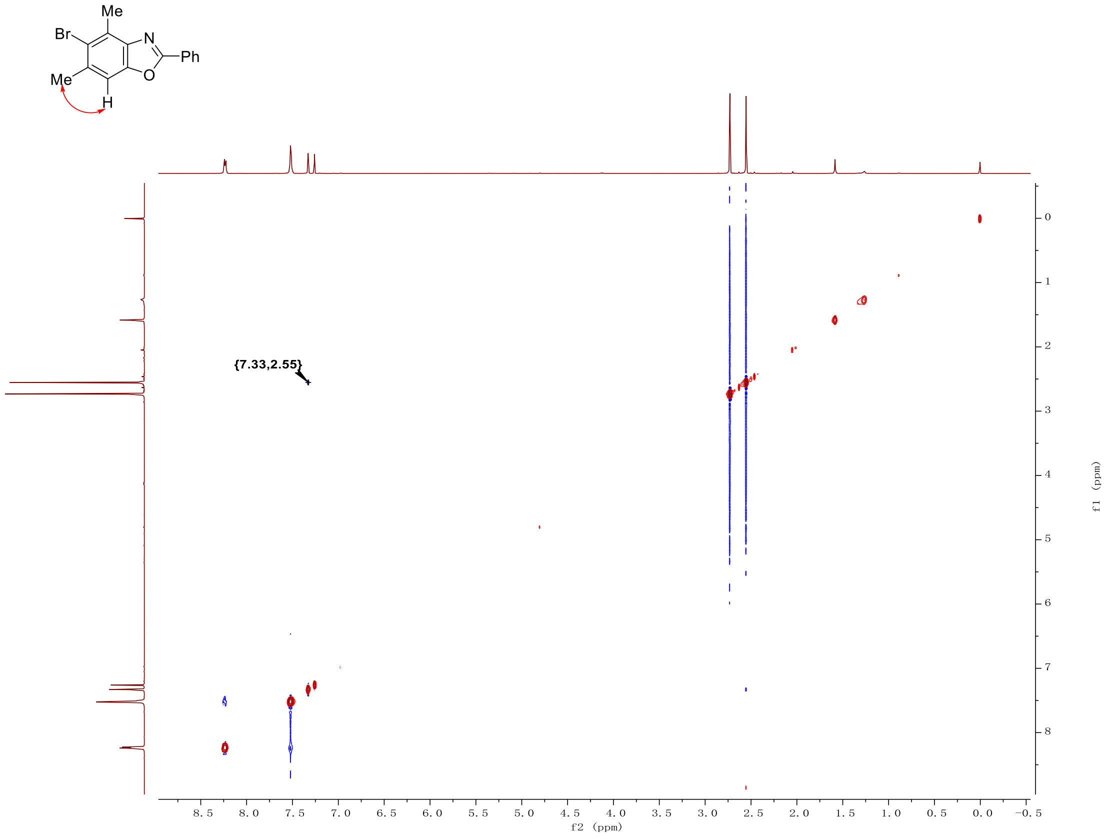

Supplementary Figure 61. **^{1}H NMR and 2D NOESY spectra of compound S74.**

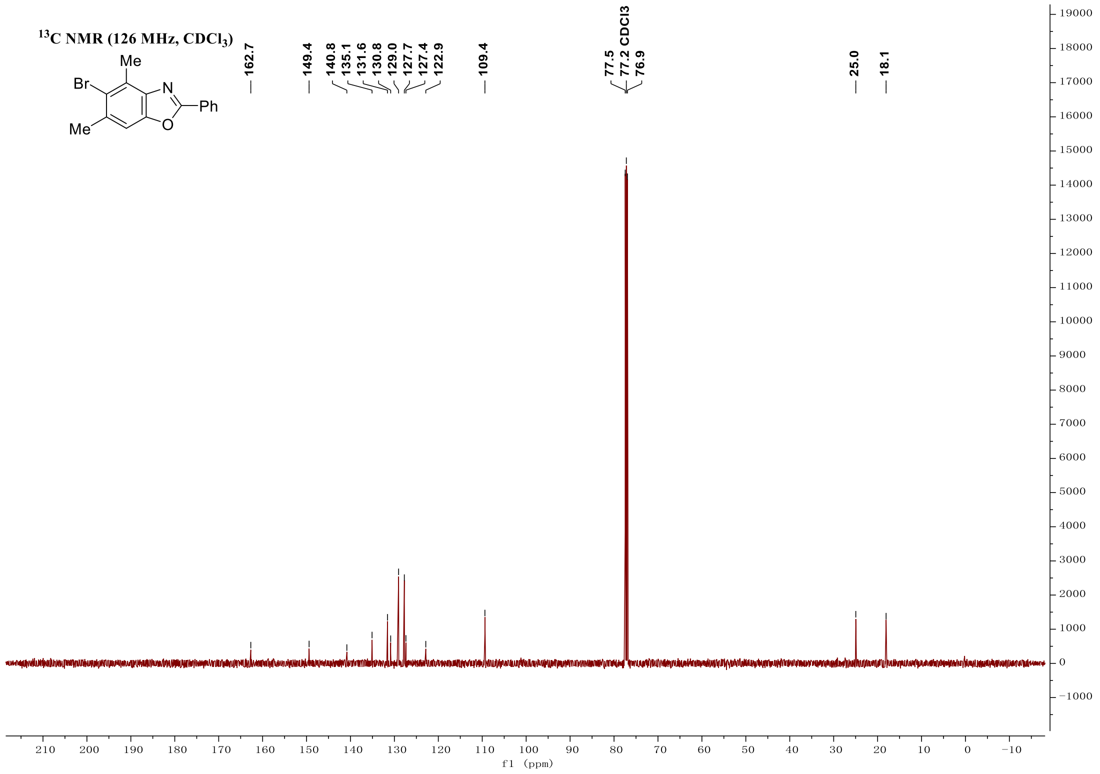

Supplementary Figure 62. ^{13}C NMR spectrum of compound S74.

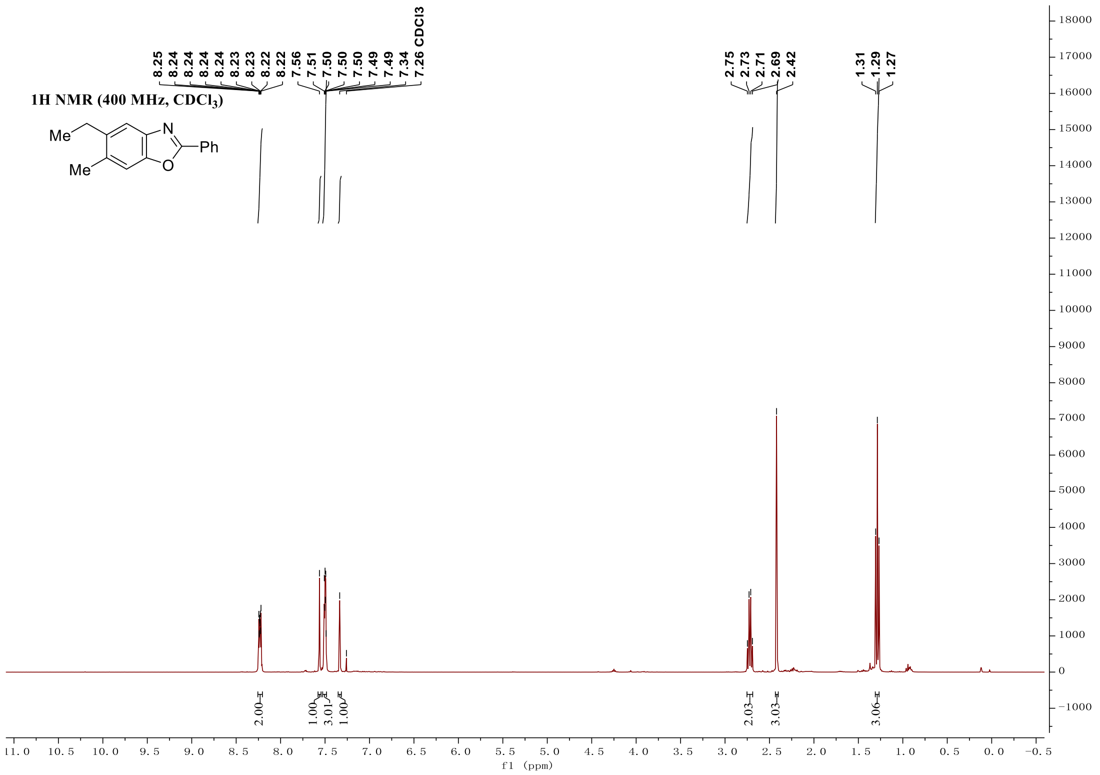

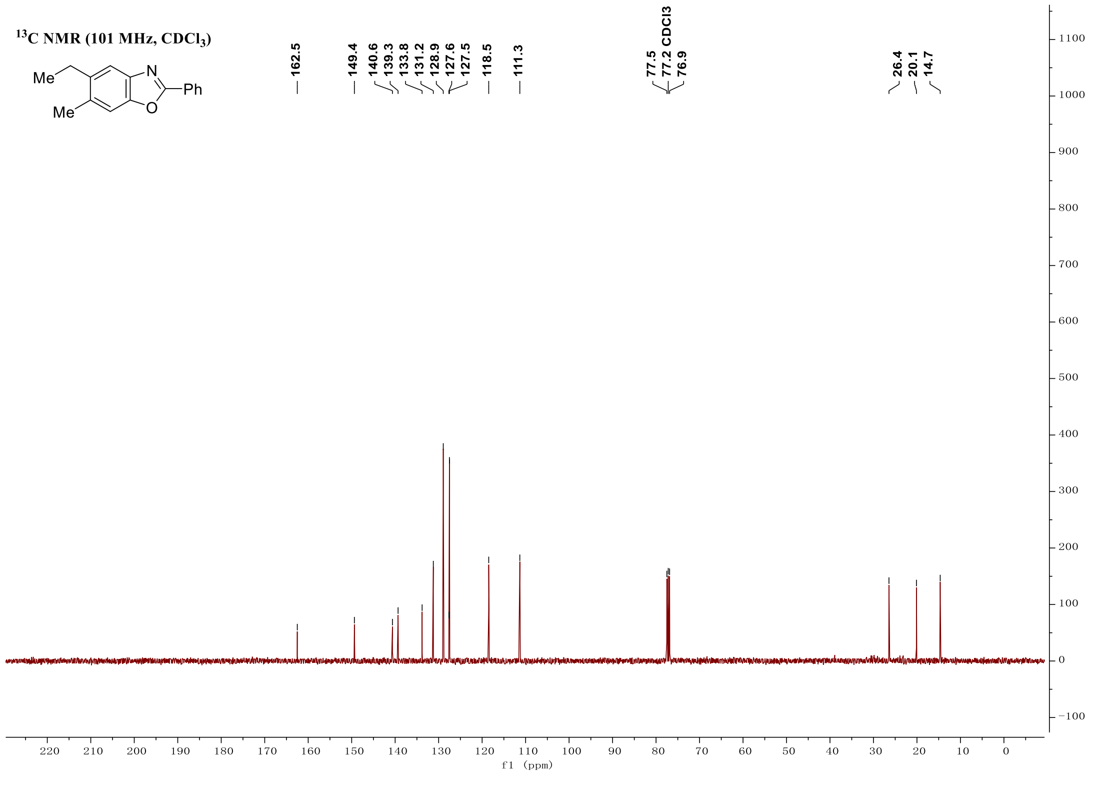

Supplementary Figure 63. ^{1}H NMR and ^{13}C NMR spectra of compound S77.

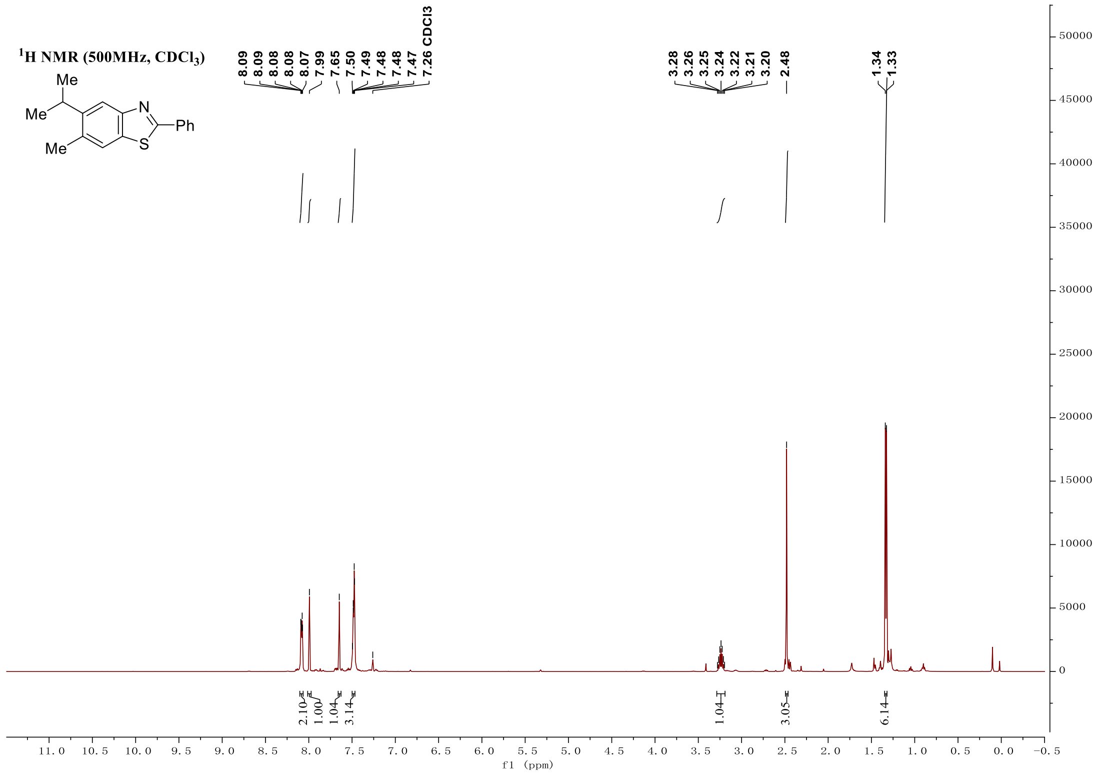

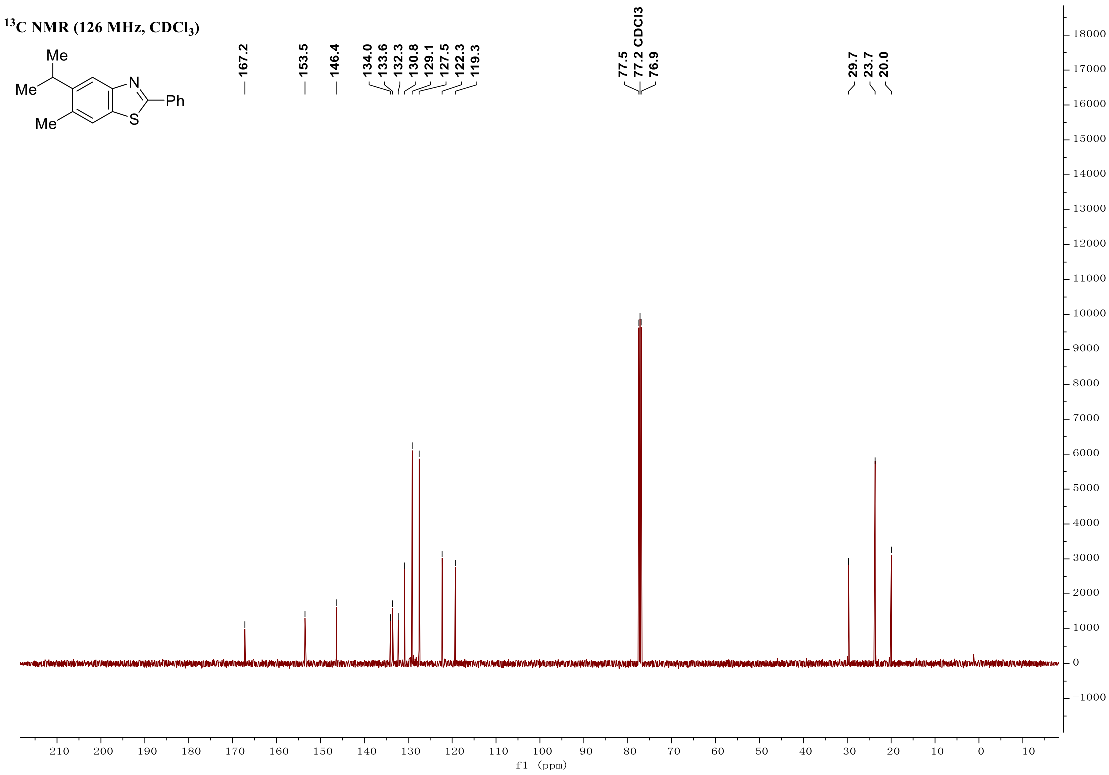

Supplementary Figure 64. ^{1}H NMR and ^{13}C NMR spectra of compound S80.

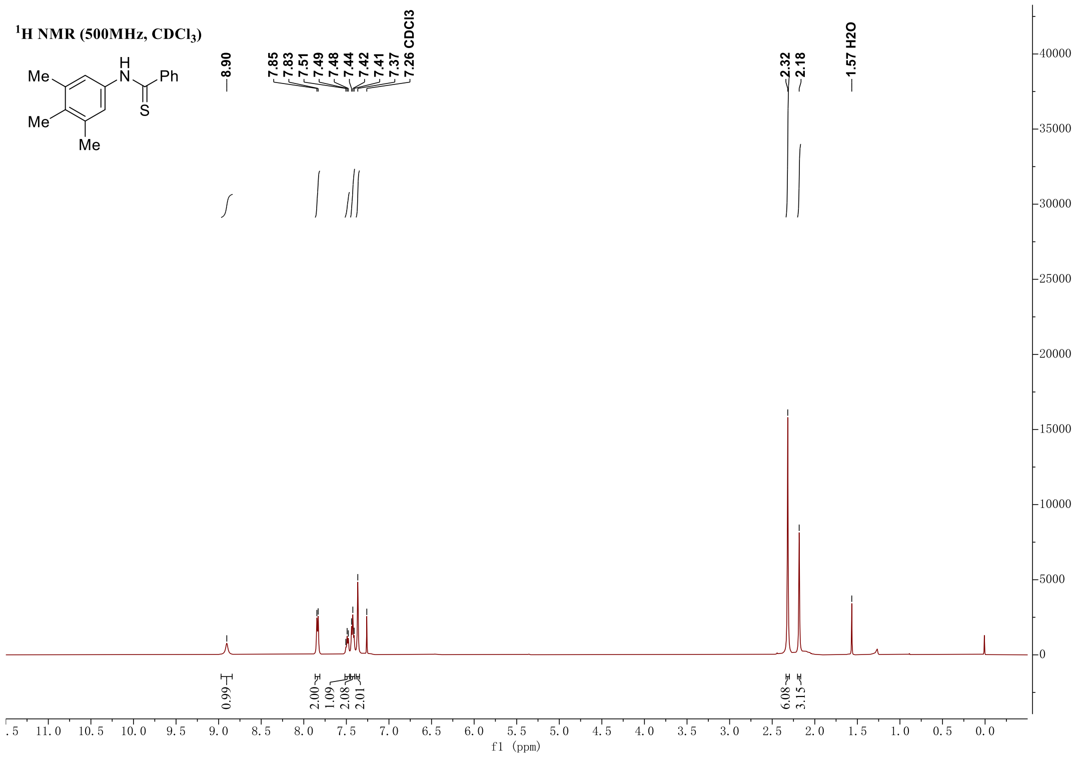

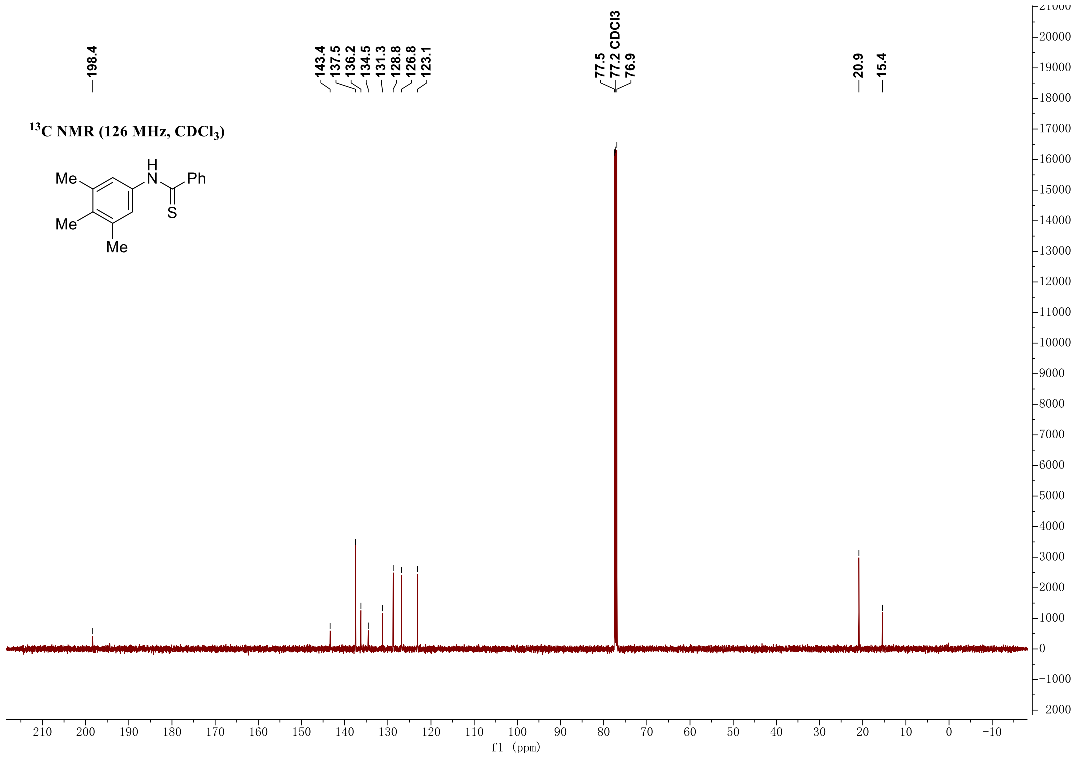

Supplementary Figure 65. ^{1}H NMR and ^{13}C NMR spectra of compound S82.

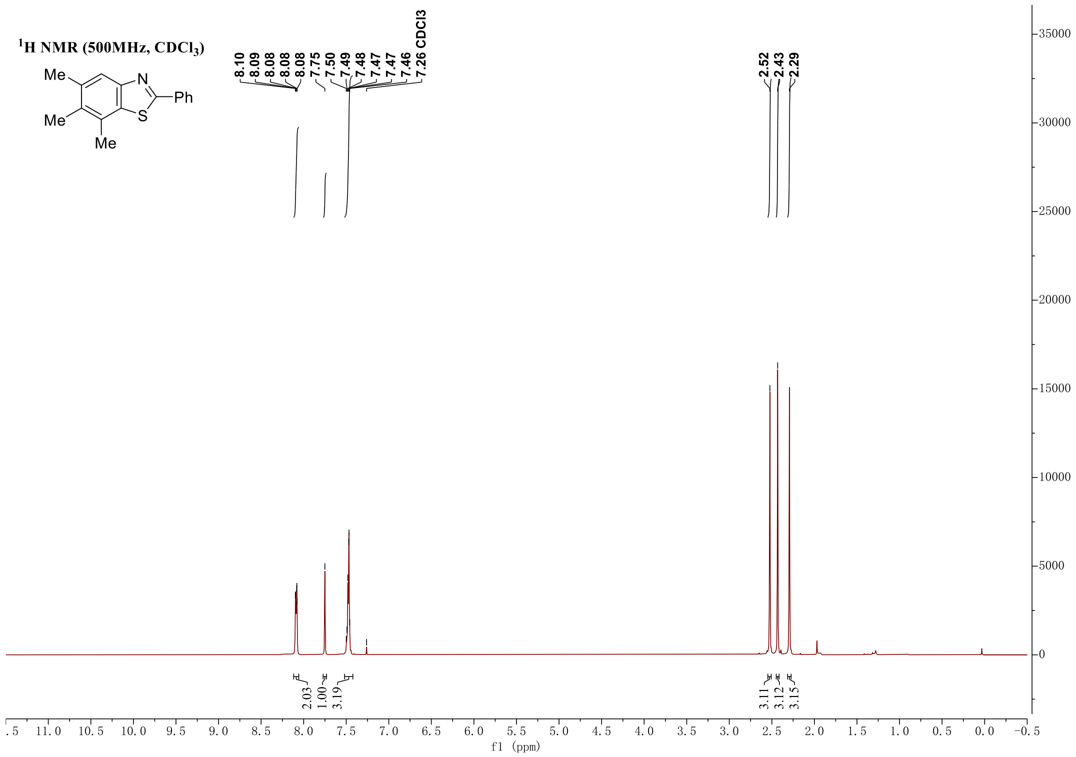

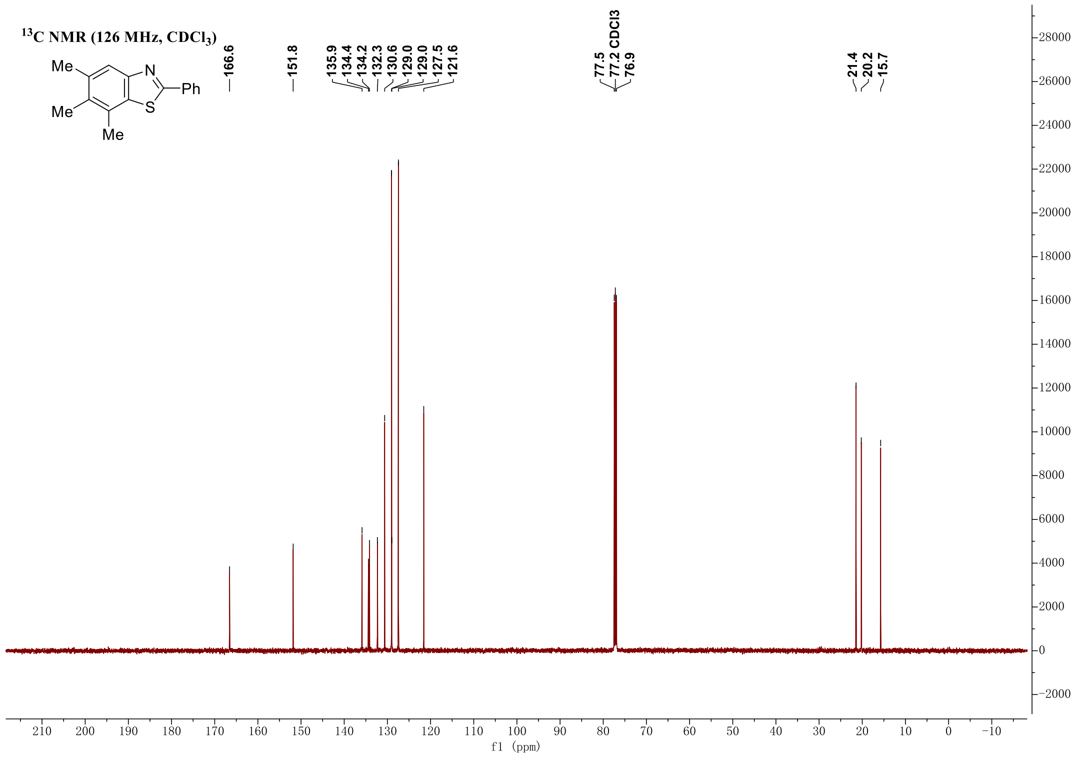

Supplementary Figure 66. ^{1}H NMR and ^{13}C NMR spectra of compound S83.

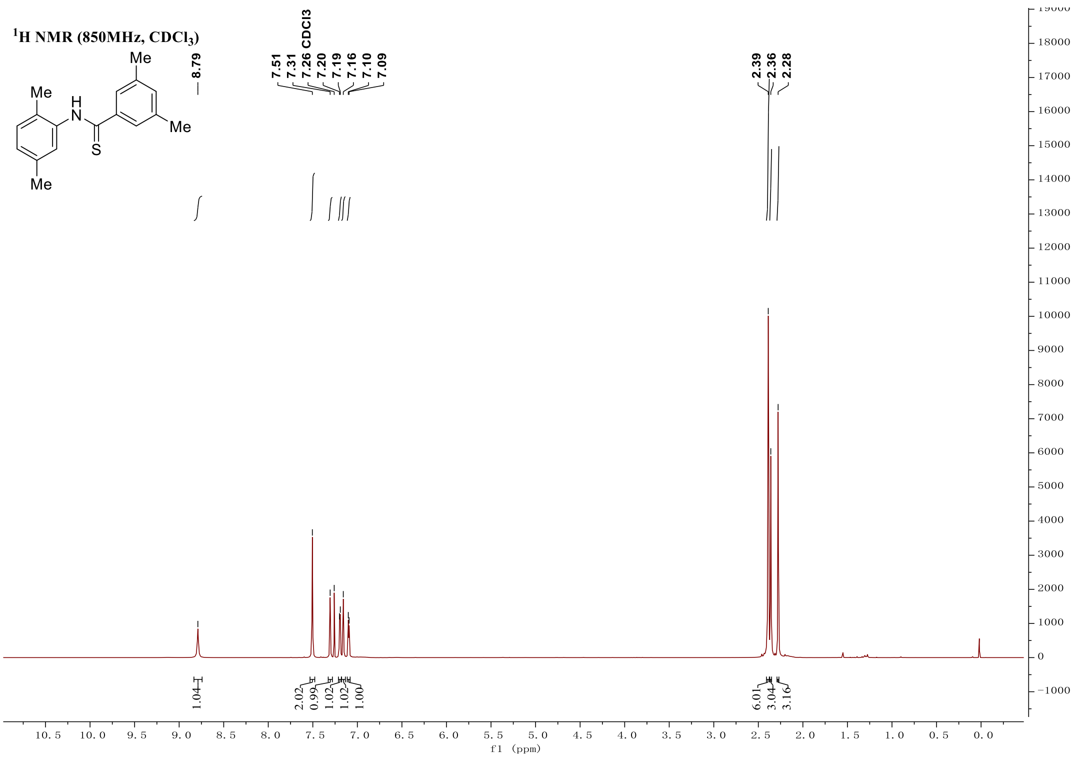

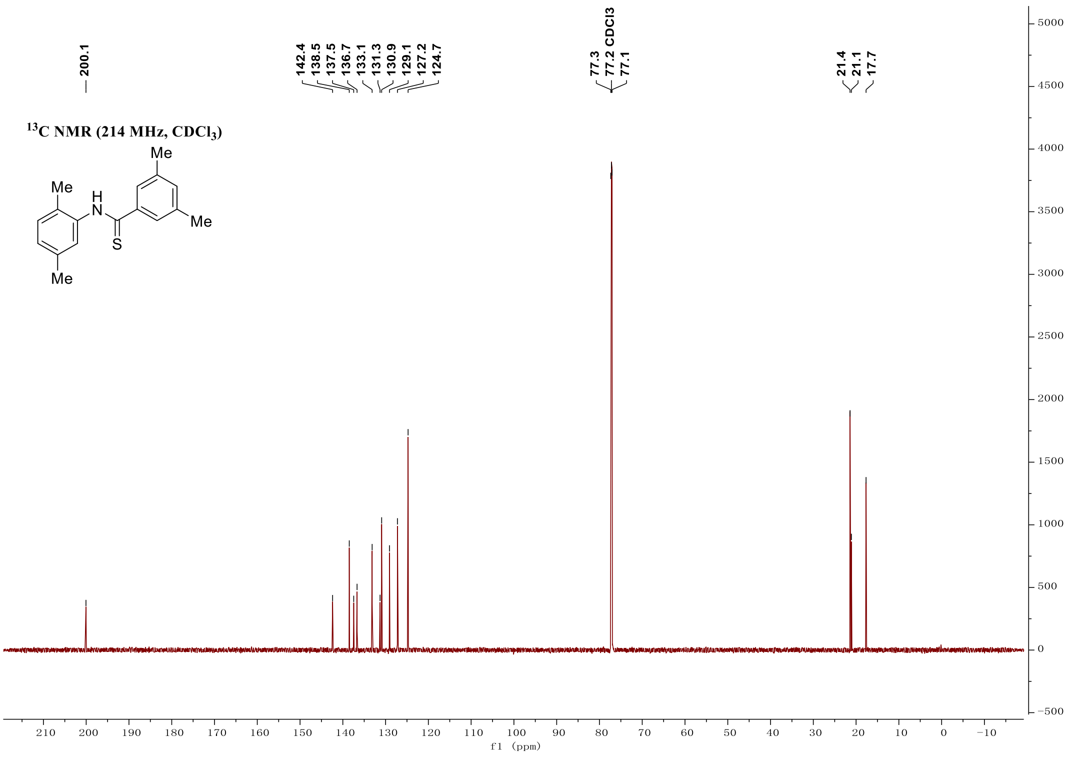

Supplementary Figure 67. ^{1}H NMR and ^{13}C NMR spectra of compound S85.

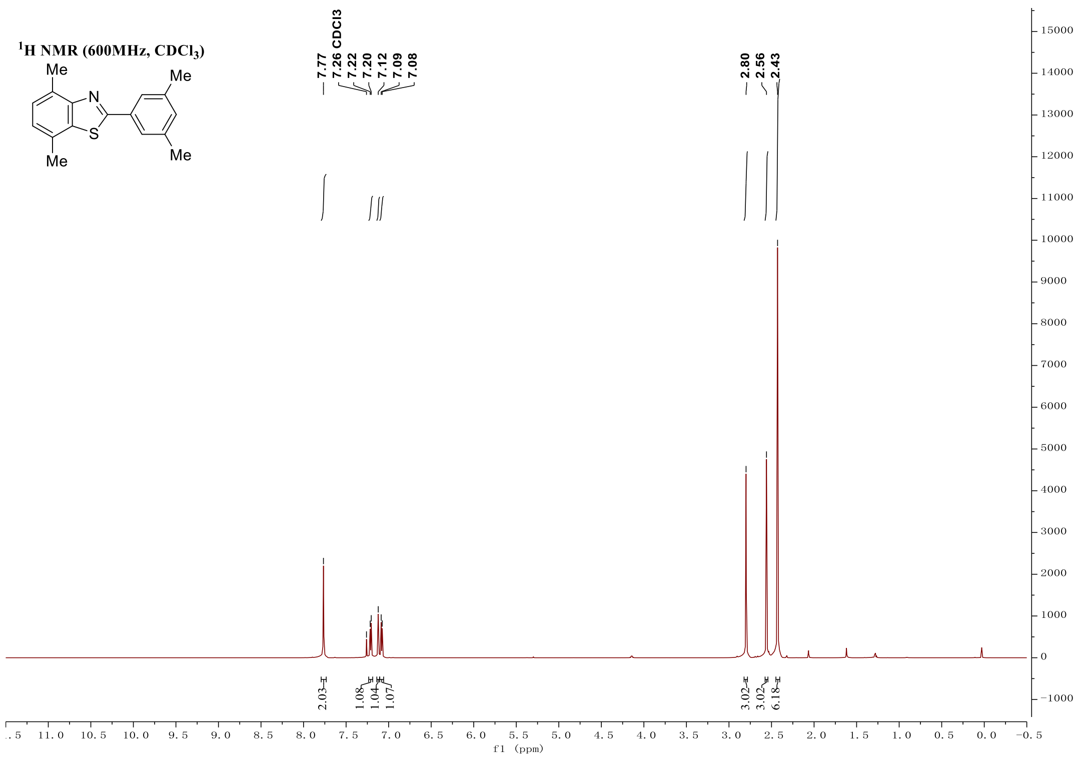

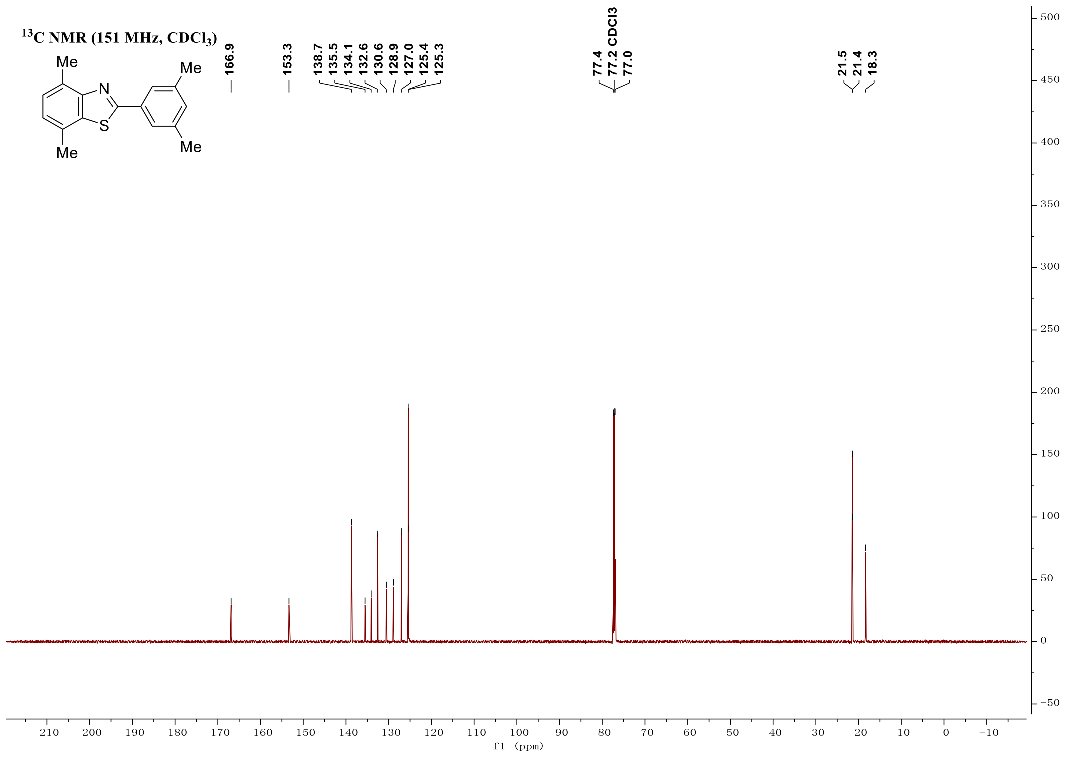

Supplementary Figure 68. ^{1}H NMR and ^{13}C NMR spectra of compound S86.

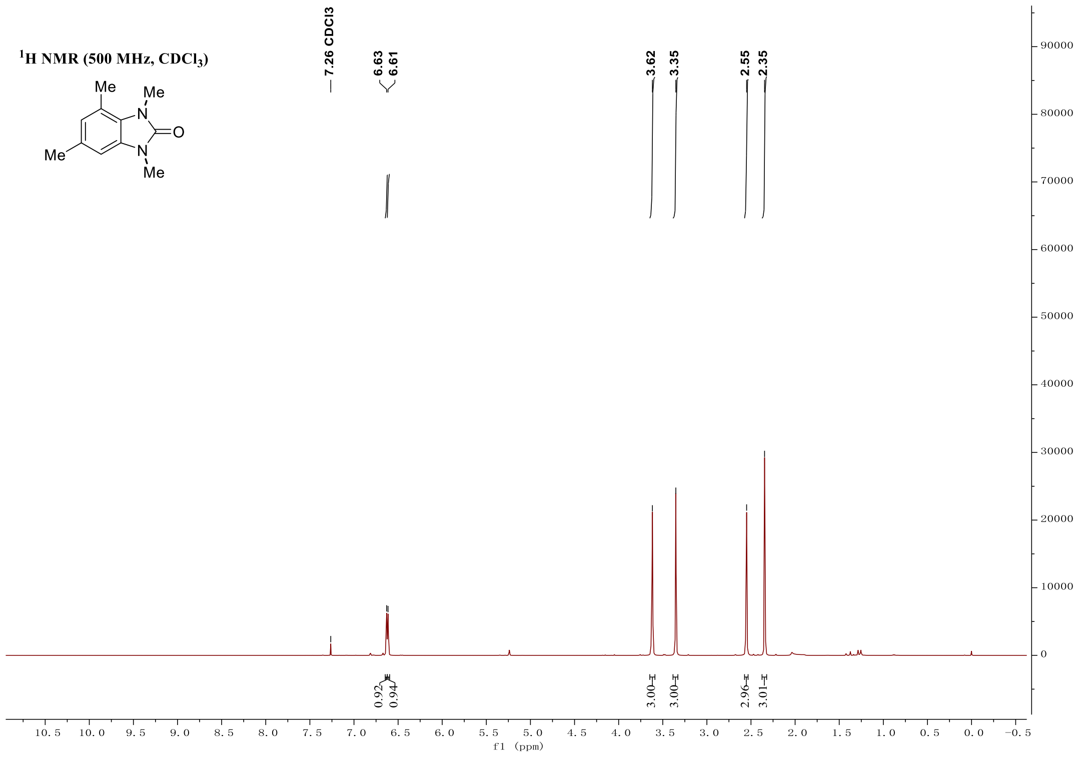

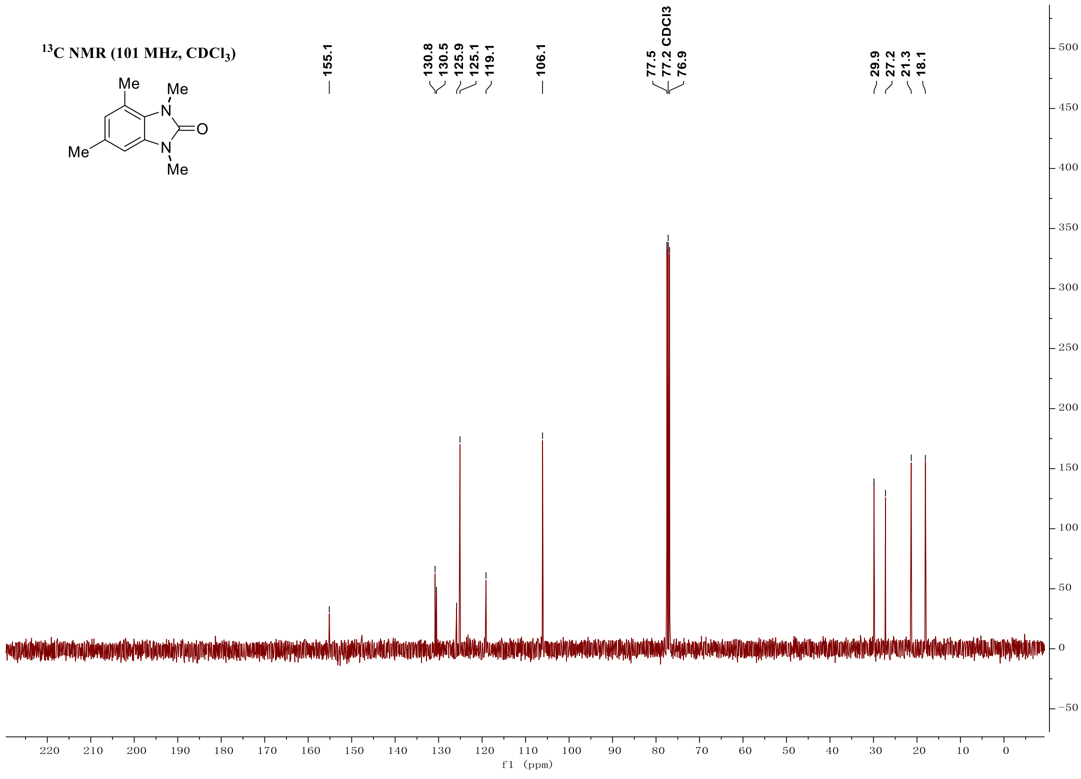

Supplementary Figure 69. ^{1}H NMR and ^{13}C NMR spectra of compound S87.

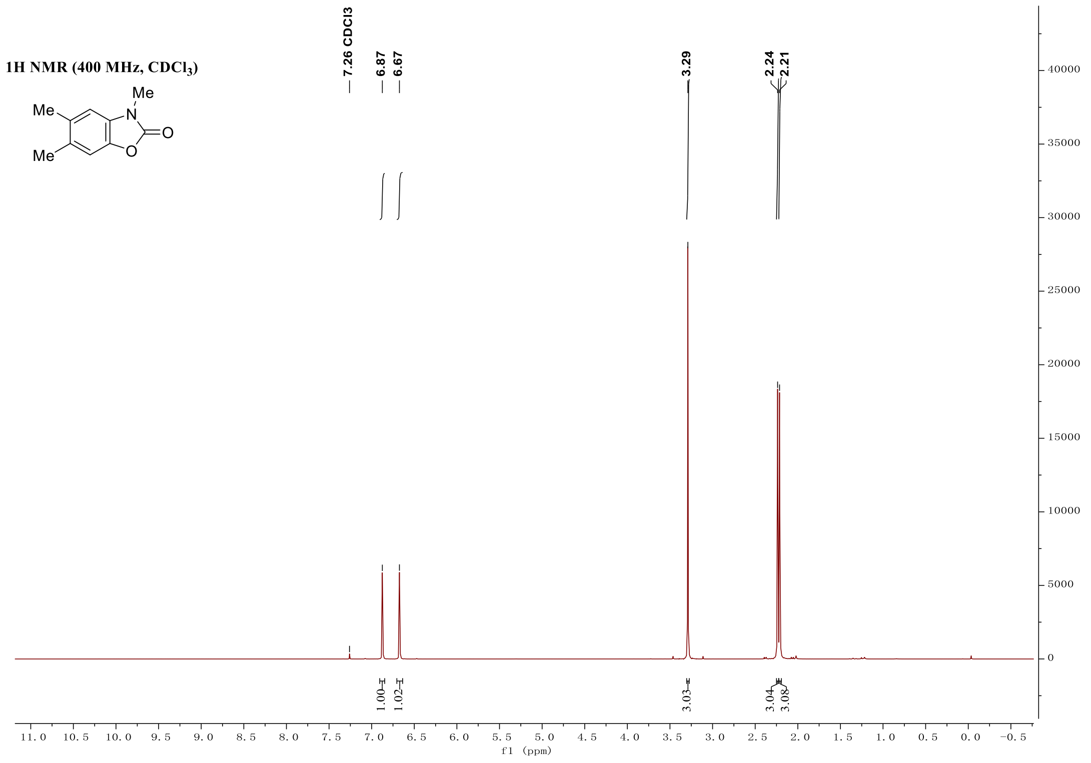

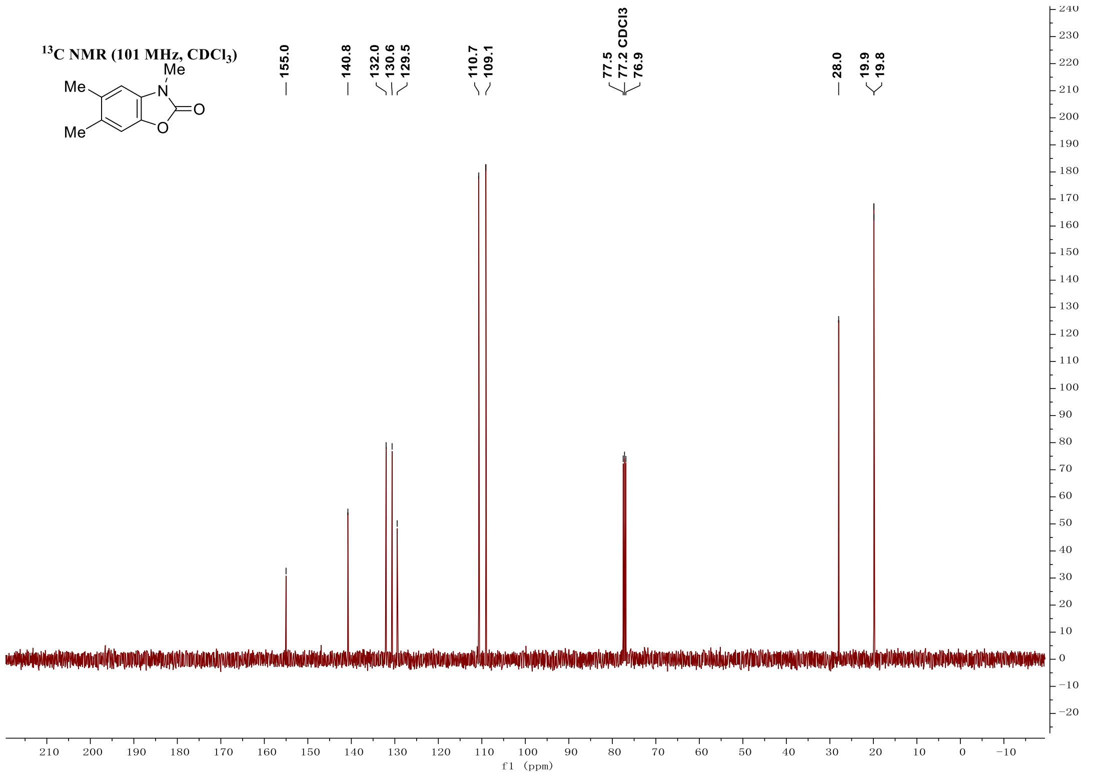

Supplementary Figure 70. ^{1}H NMR and ^{13}C NMR spectra of compound S90.

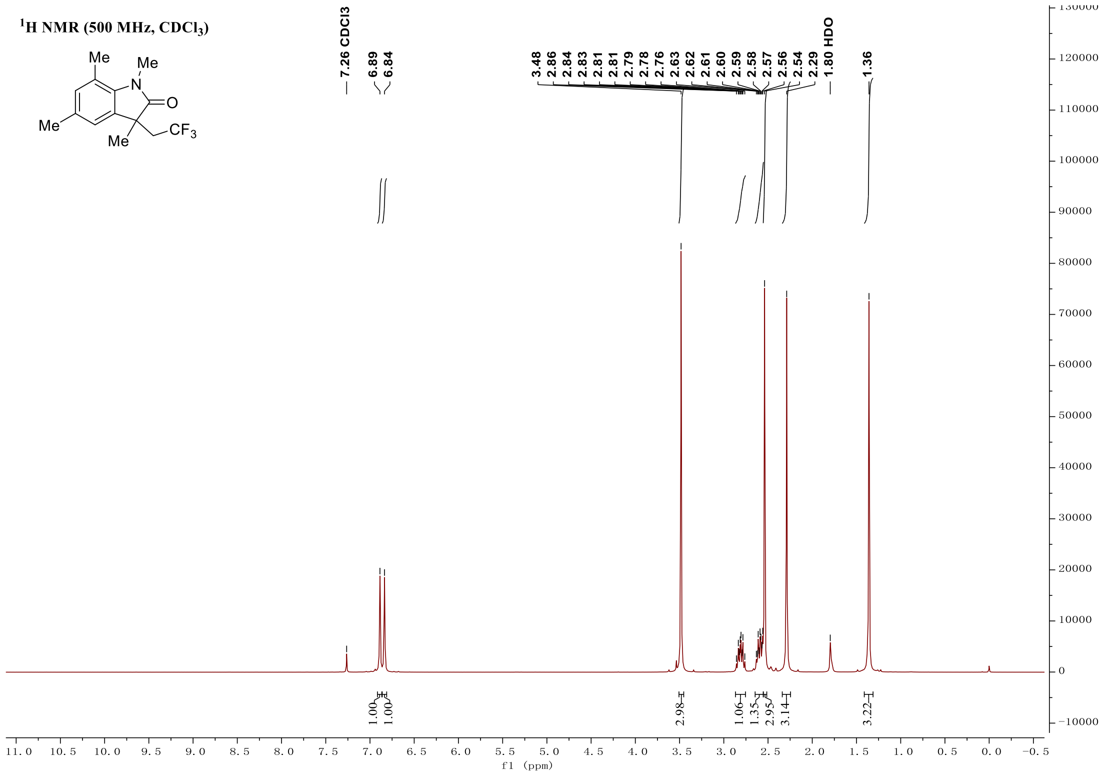

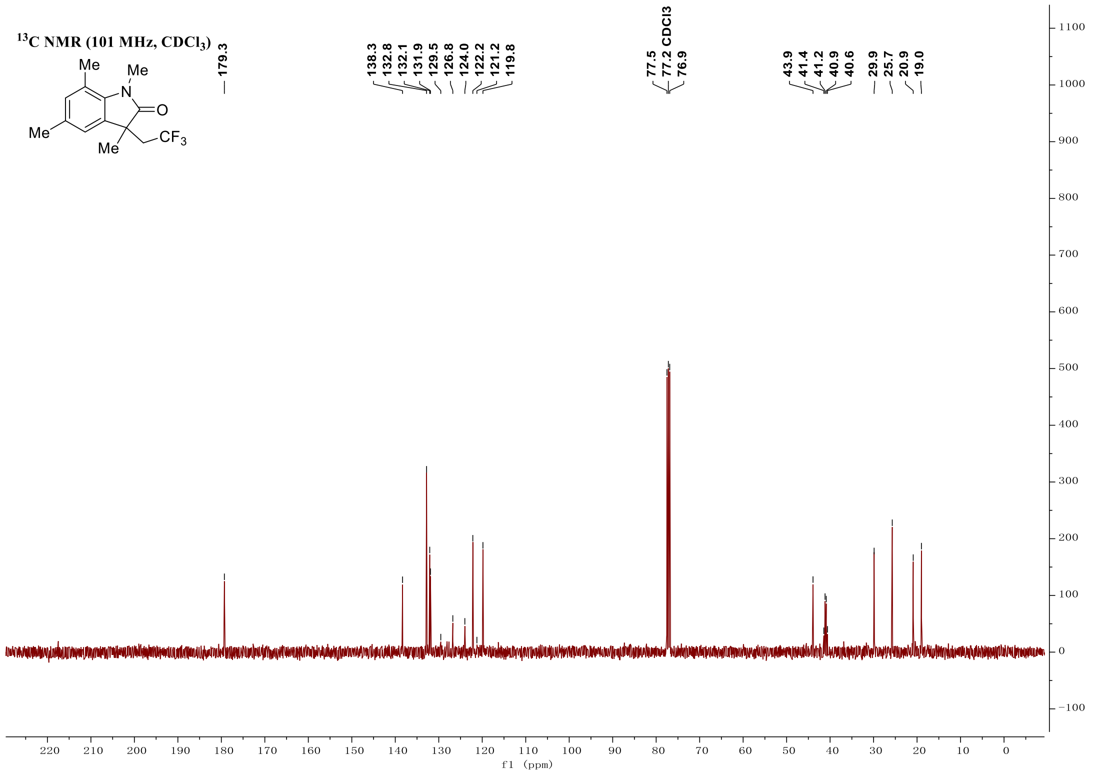

Supplementary Figure 71. ^{1}H NMR and ^{13}C NMR spectra of compound S92.

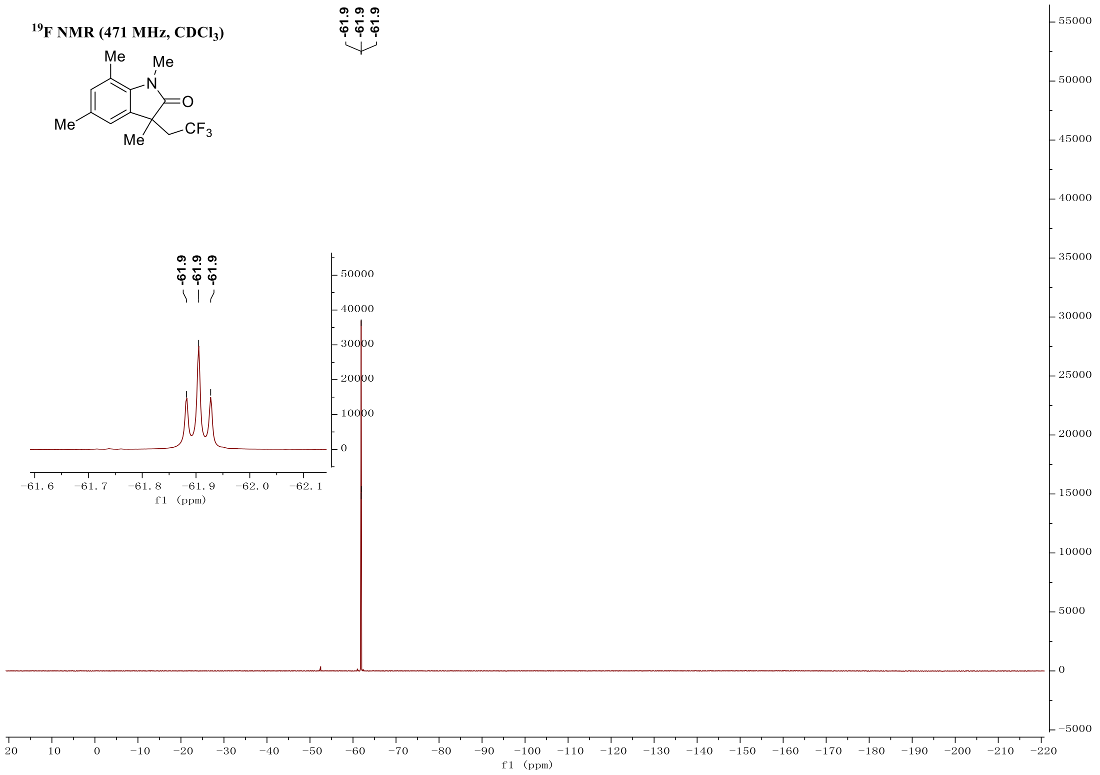

Supplementary Figure 72. ^{19}F NMR spectrum of compound S92.

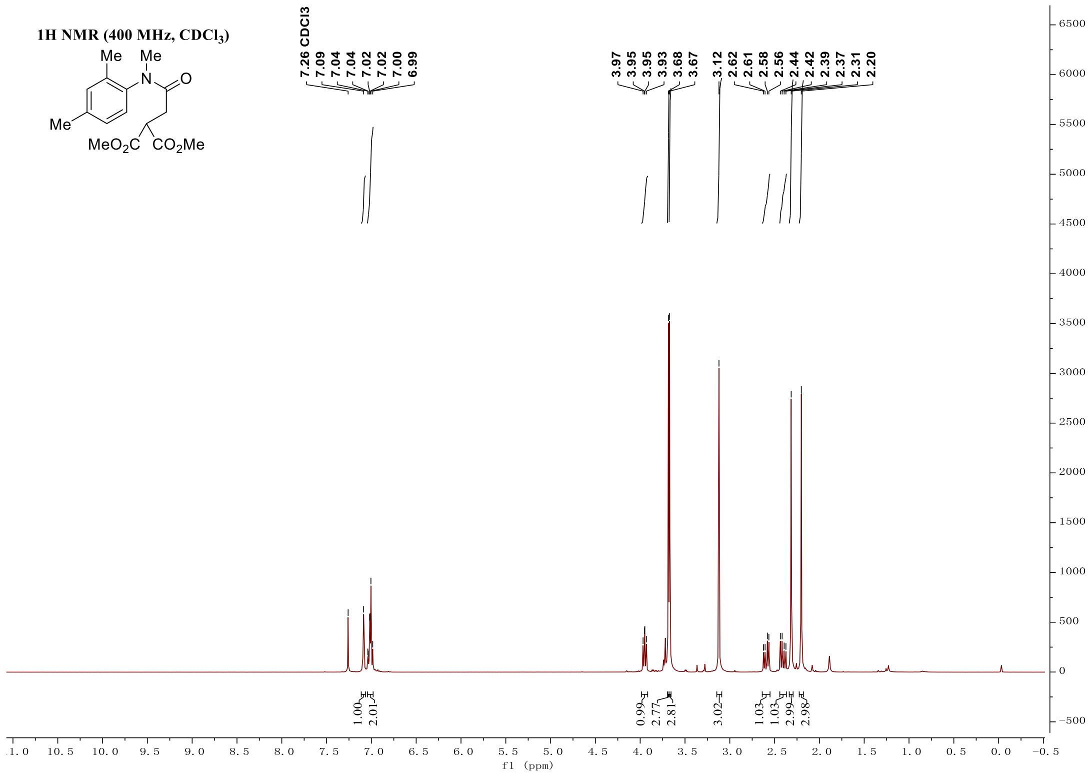

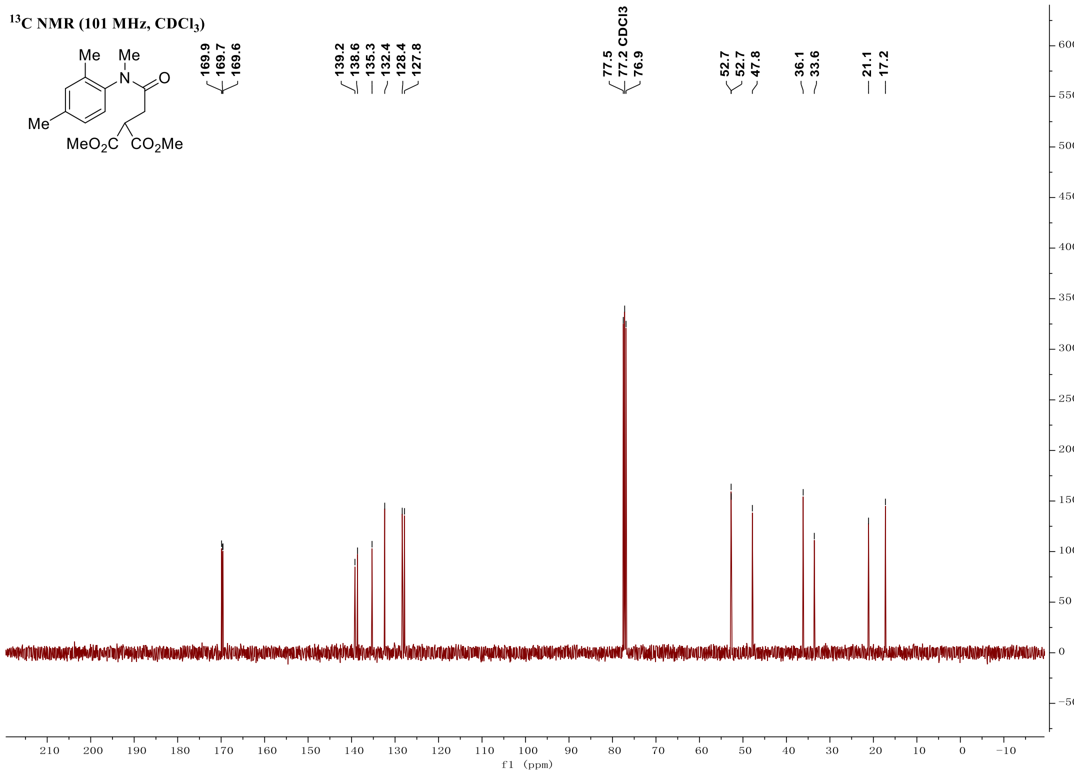

Supplementary Figure 73. ^{1}H NMR and ^{13}C NMR spectra of compound S94.

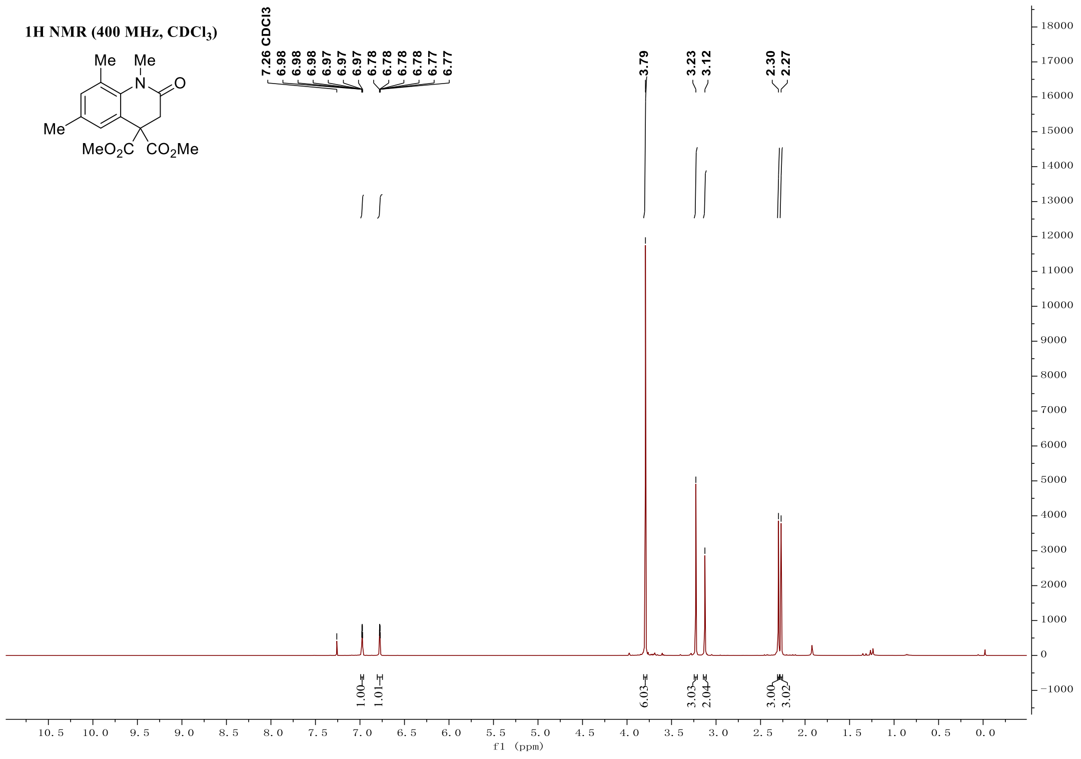

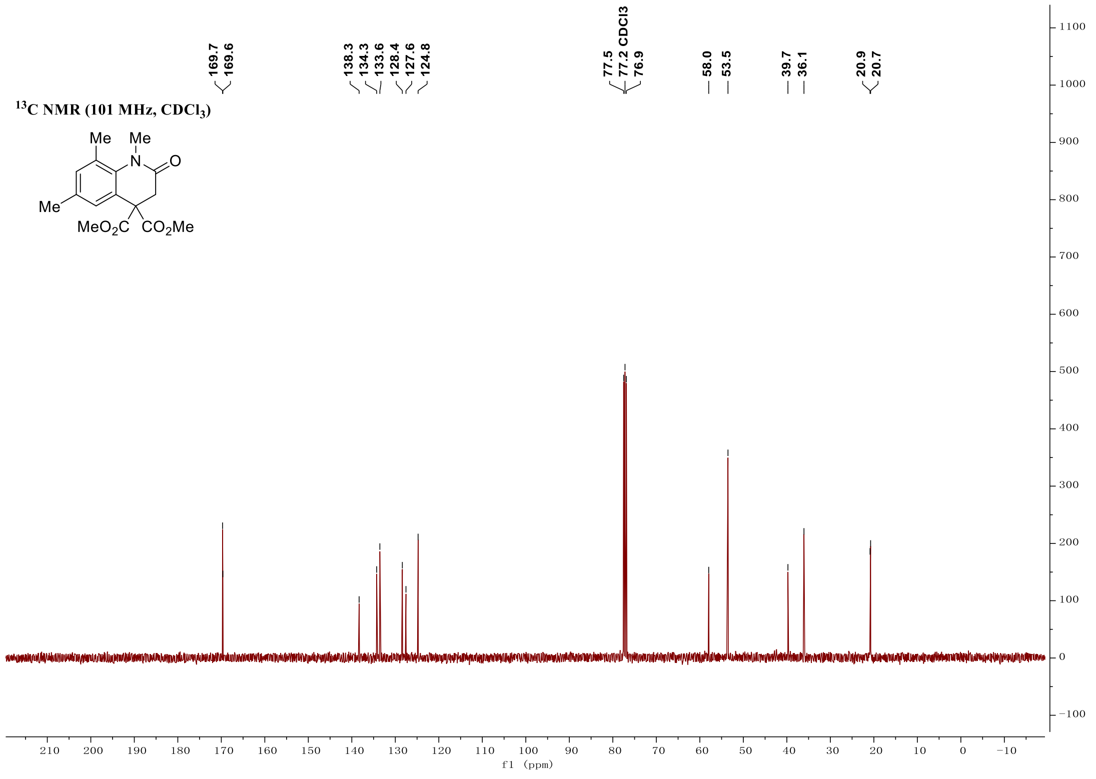

Supplementary Figure 74. ^{1}H NMR and ^{13}C NMR spectra of compound S95.

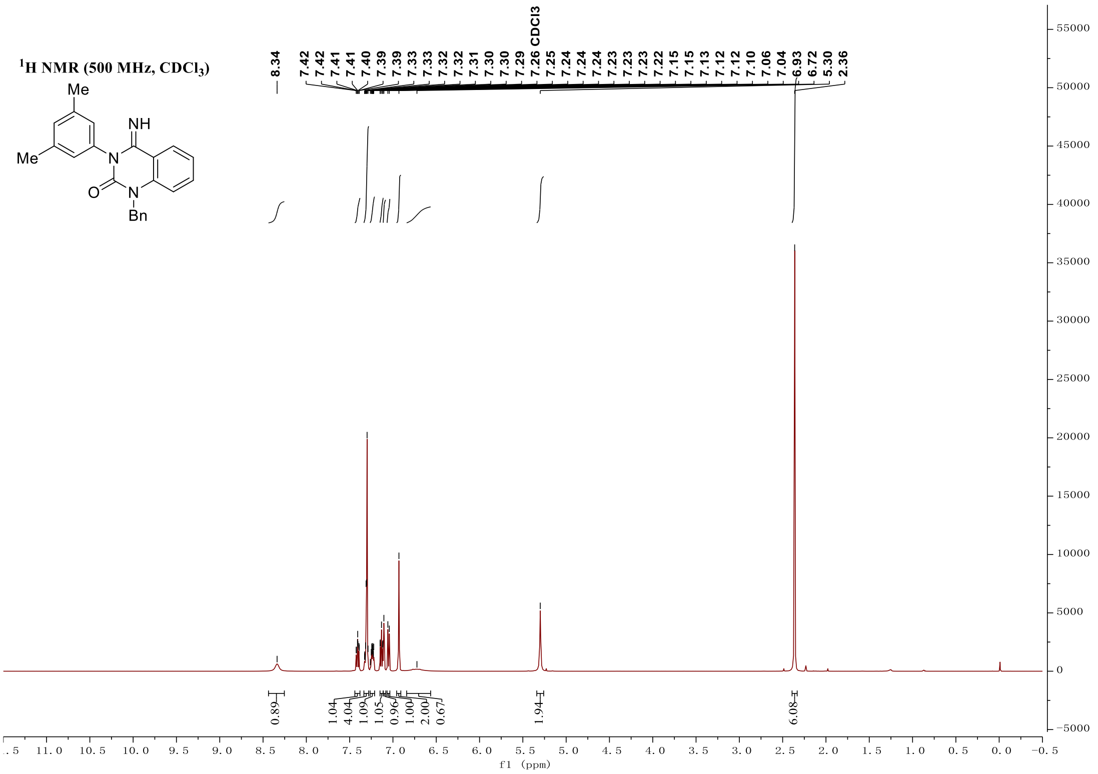

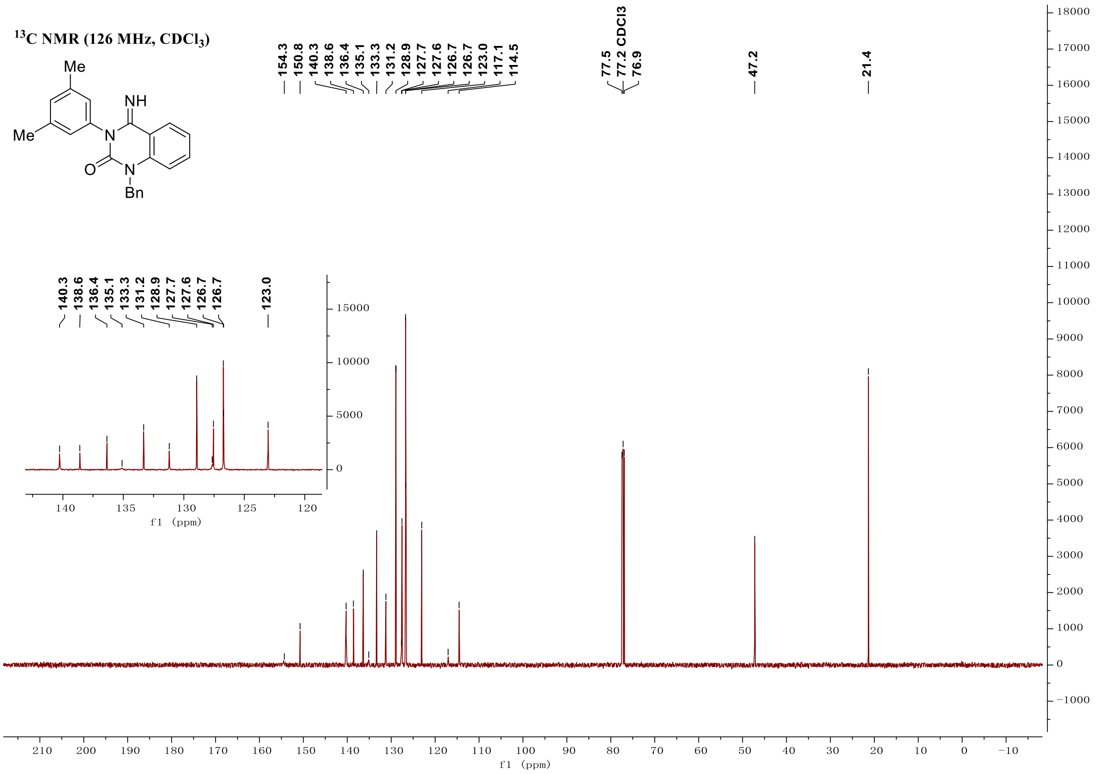

Supplementary Figure 75. ^{1}H NMR and ^{13}C NMR spectra of compound S100.

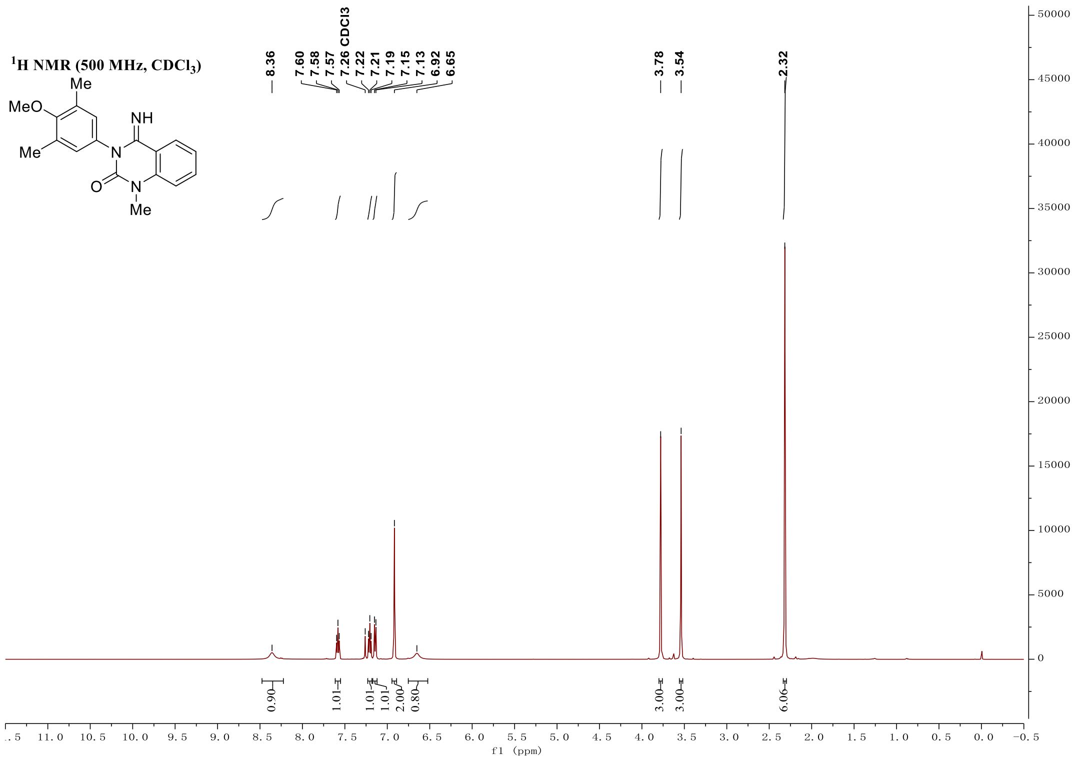

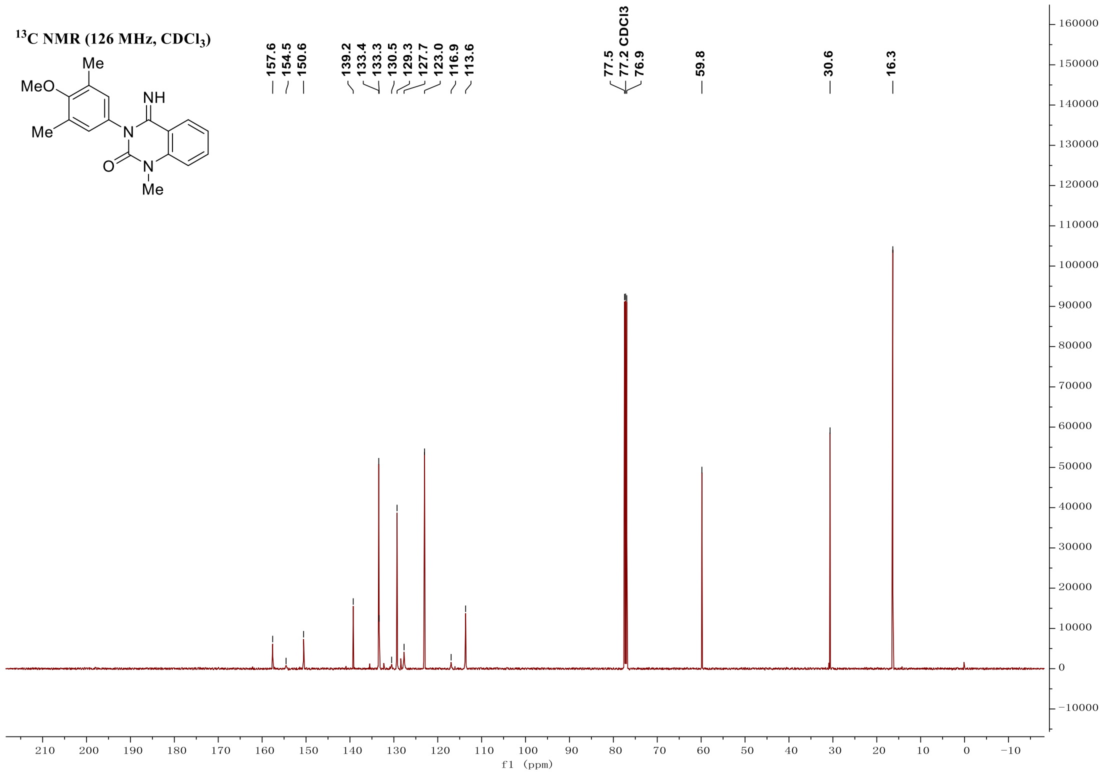

Supplementary Figure 76. ^{1}H **NMR and** ^{13}C **NMR spectra of compound S101.**

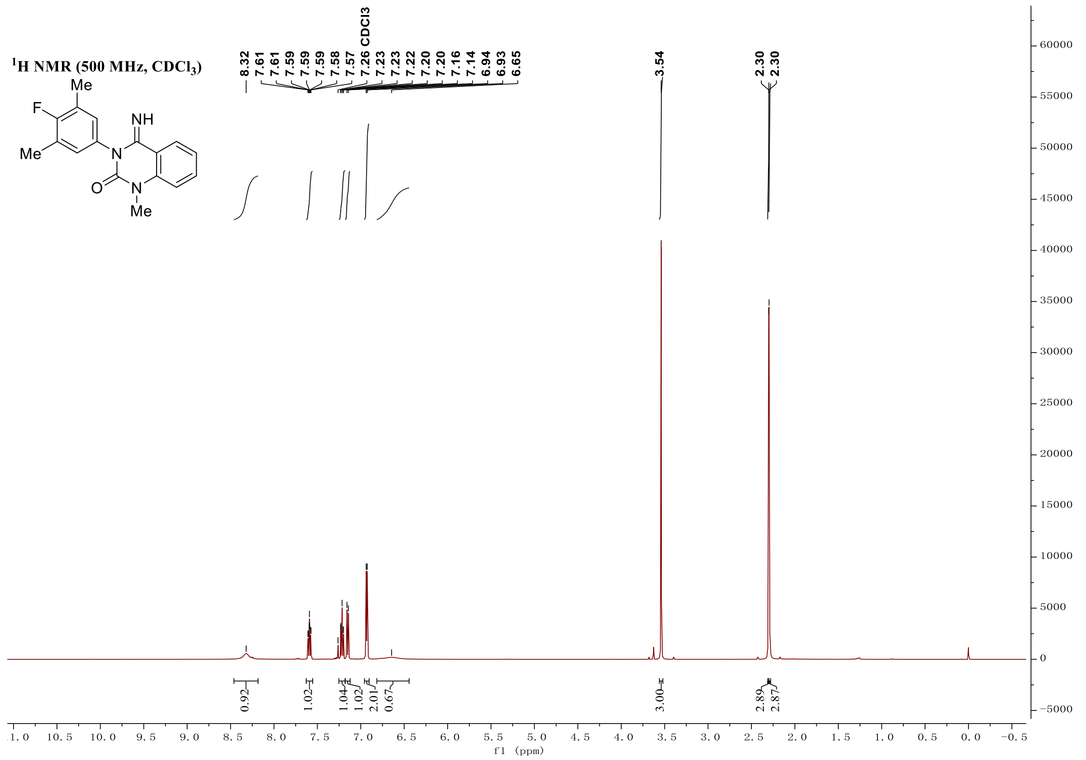

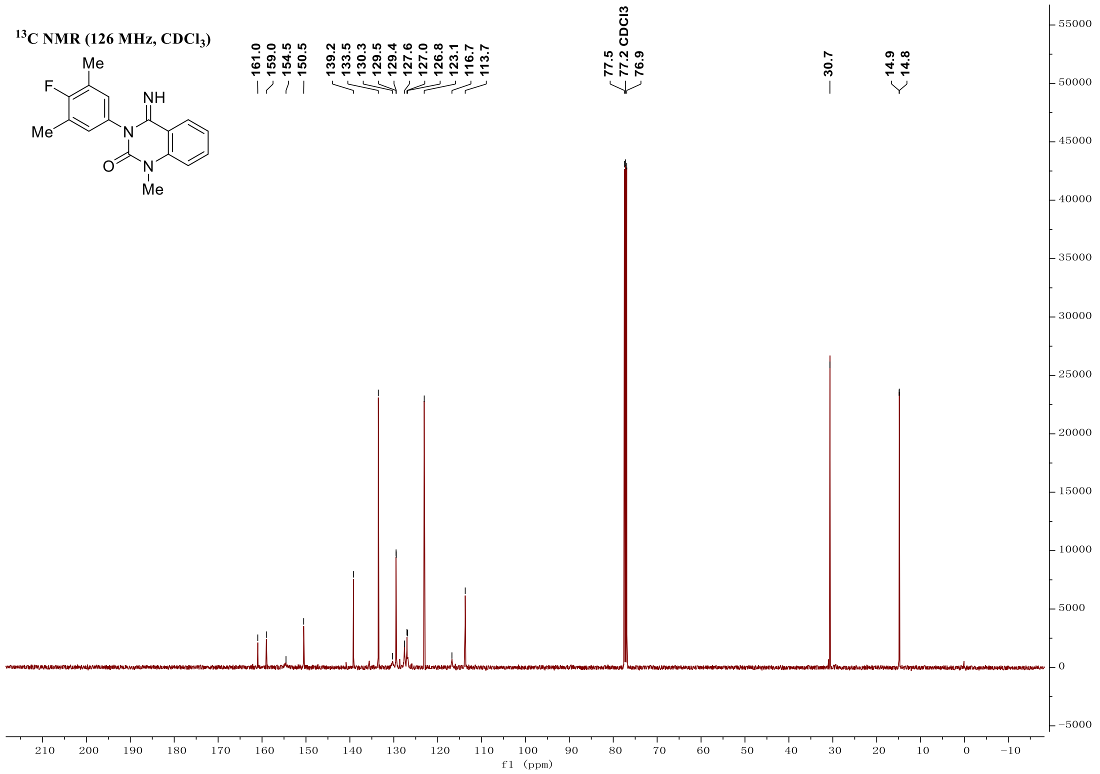

Supplementary Figure 77. ^{1}H NMR and ^{13}C NMR spectra of compound S102.

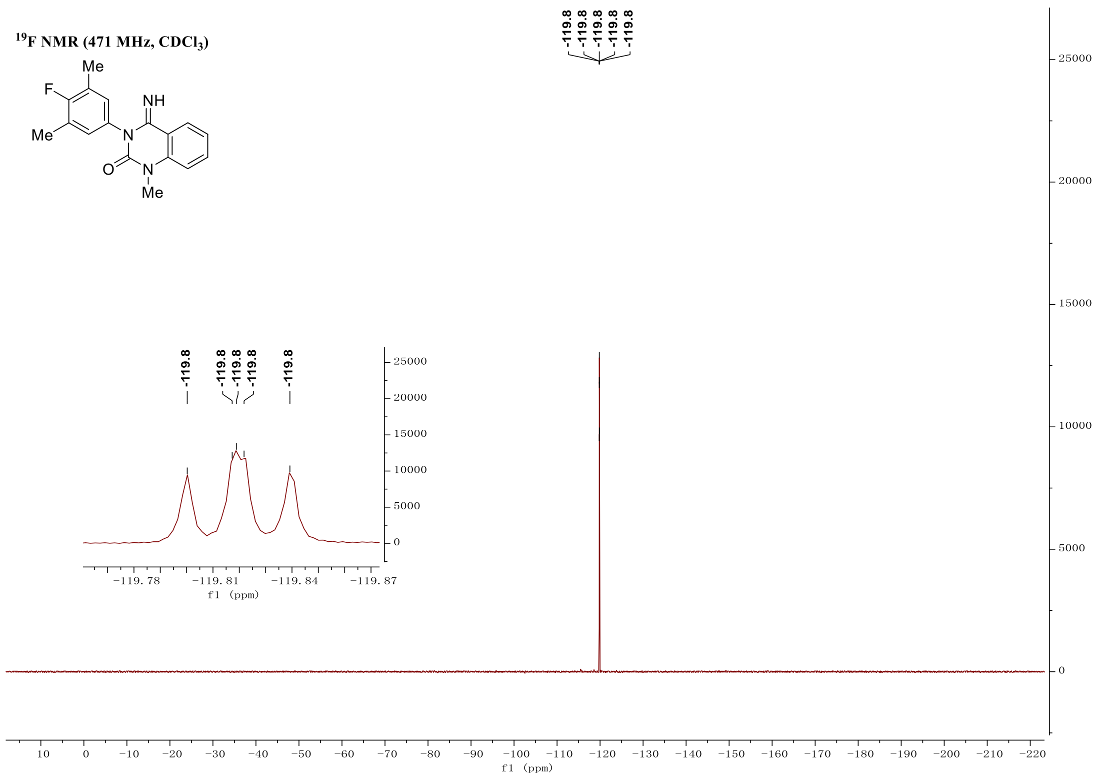

Supplementary Figure 78. ^{19}F NMR spectrum of compound S102.

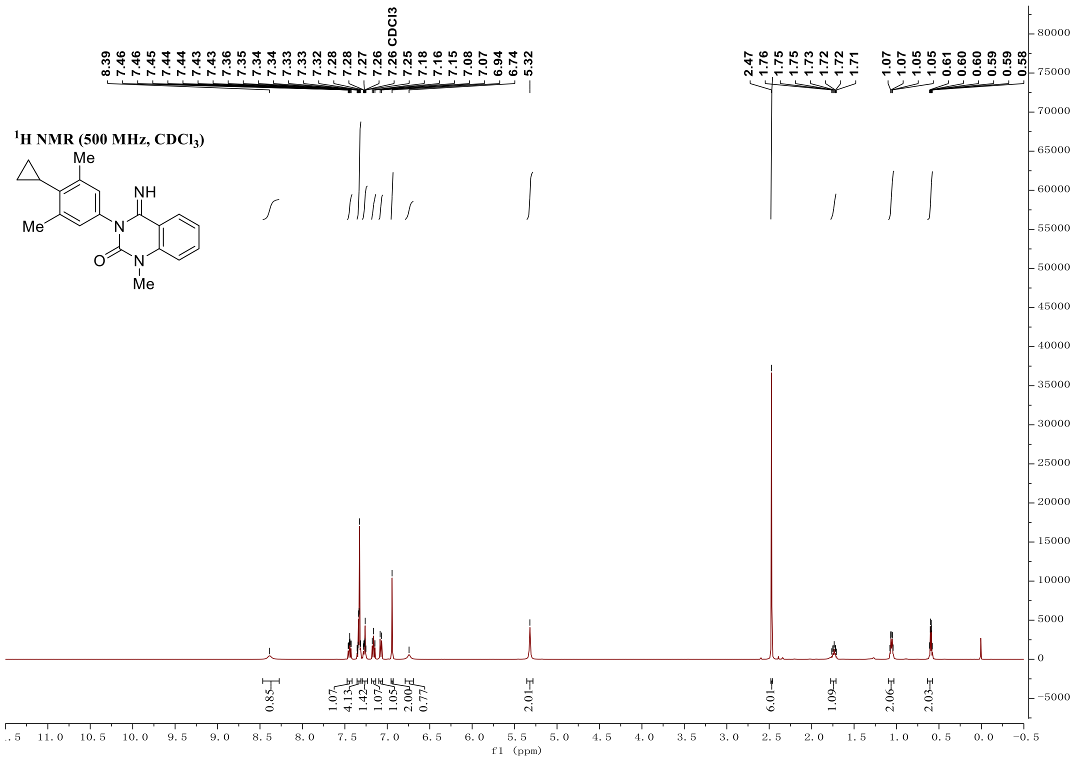

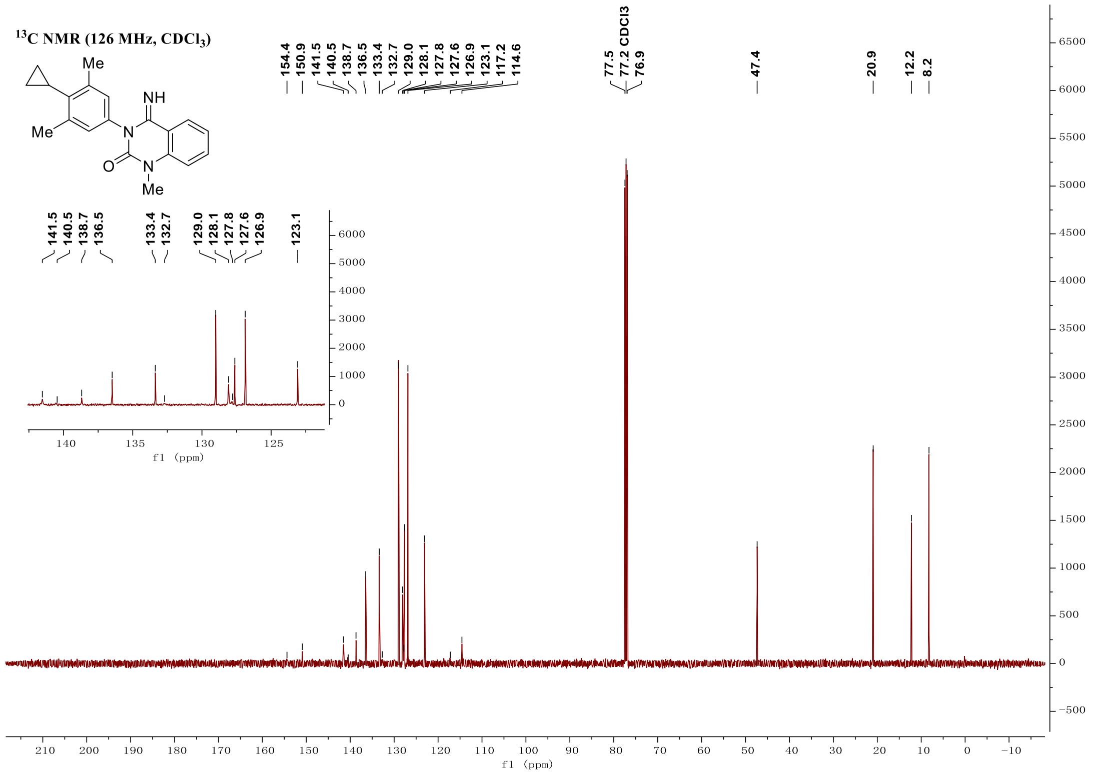

Supplementary Figure 79. ^{1}H NMR and ^{13}C NMR spectra of compound S103.

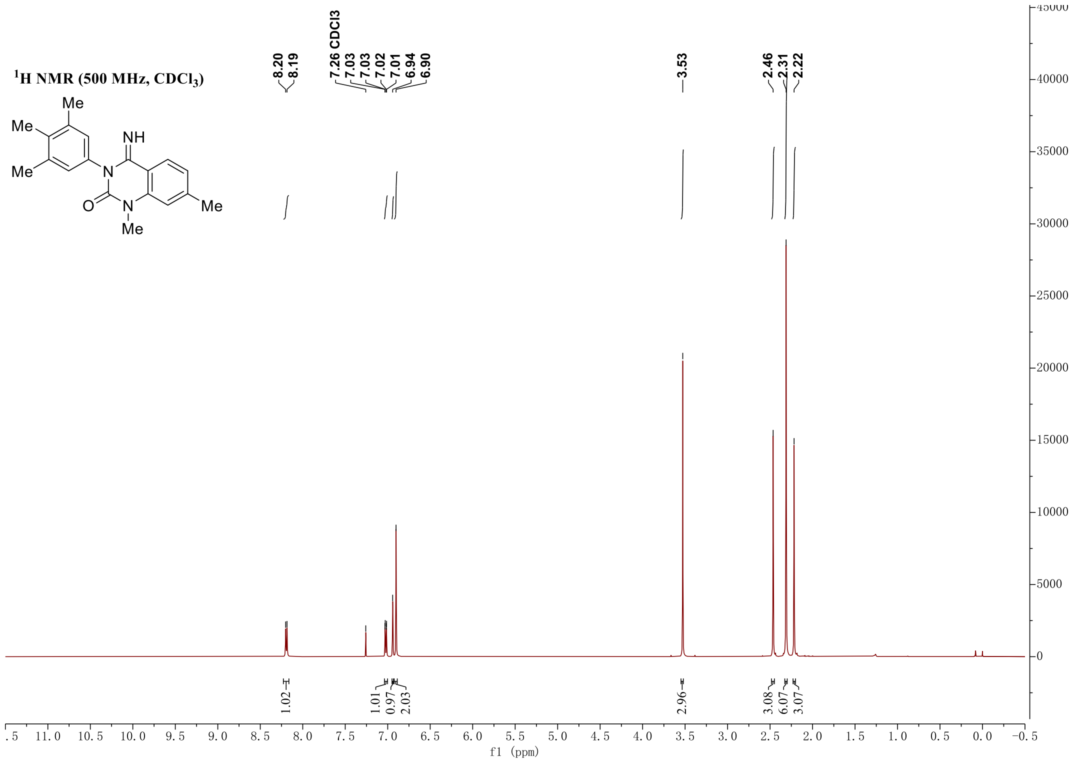

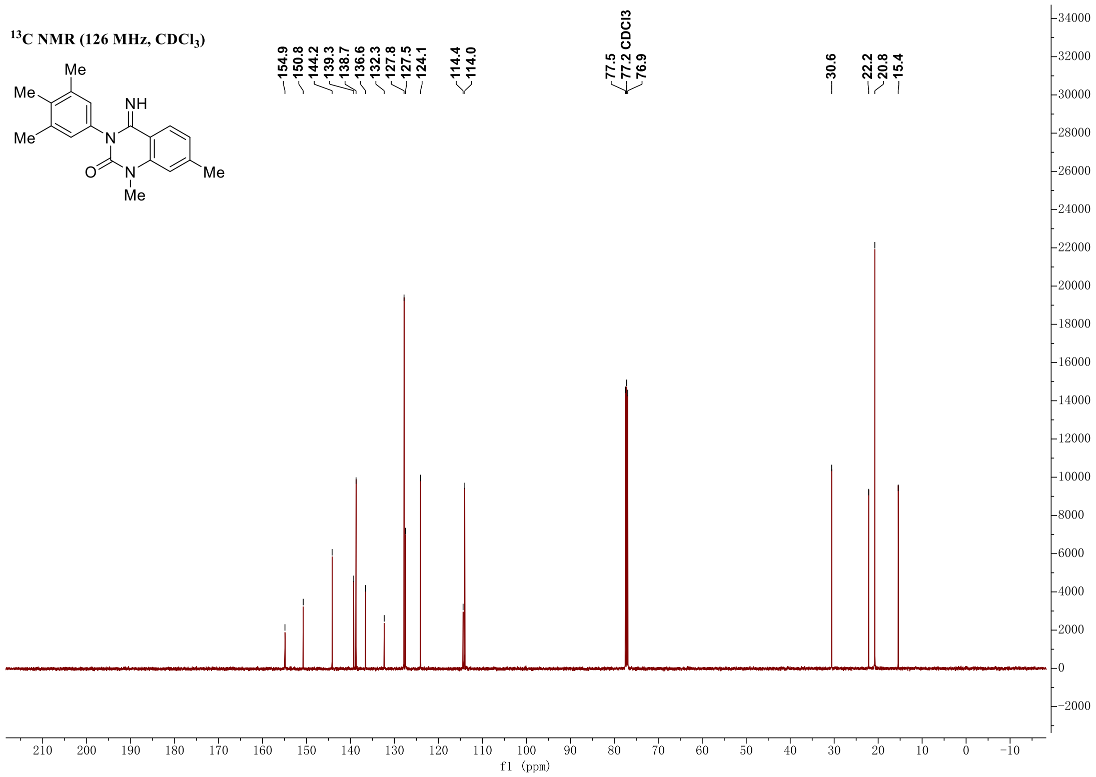

Supplementary Figure 80. ^{1}H NMR and ^{13}C NMR spectra of compound S104.

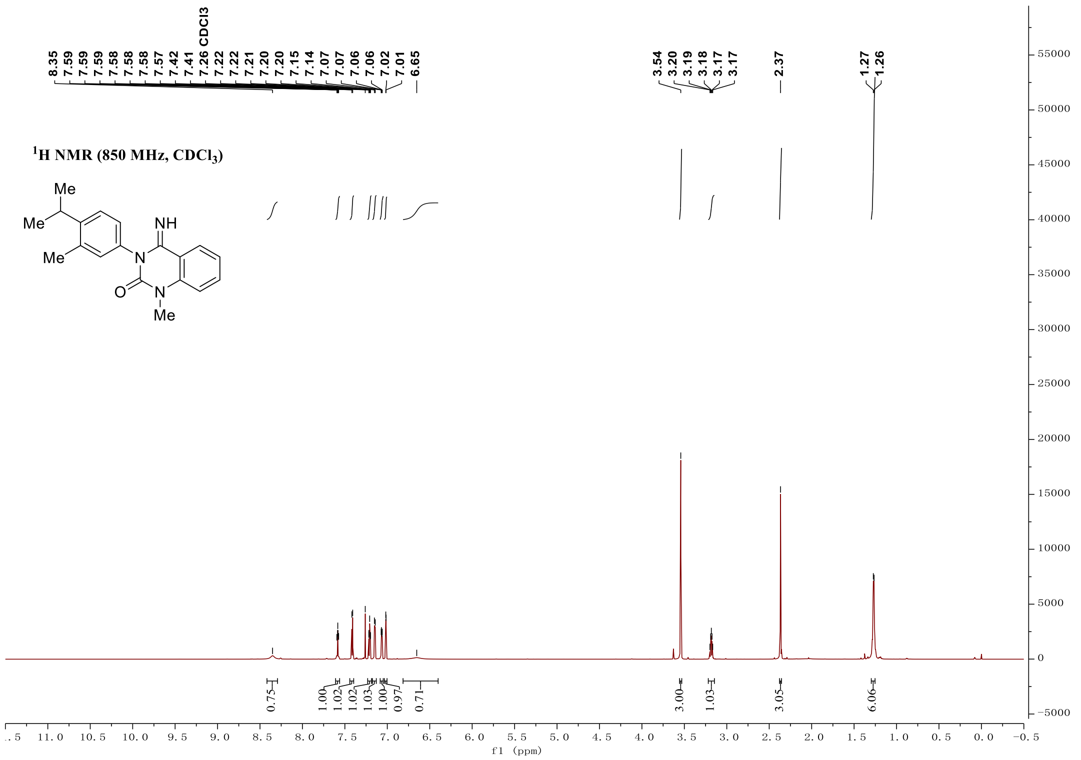

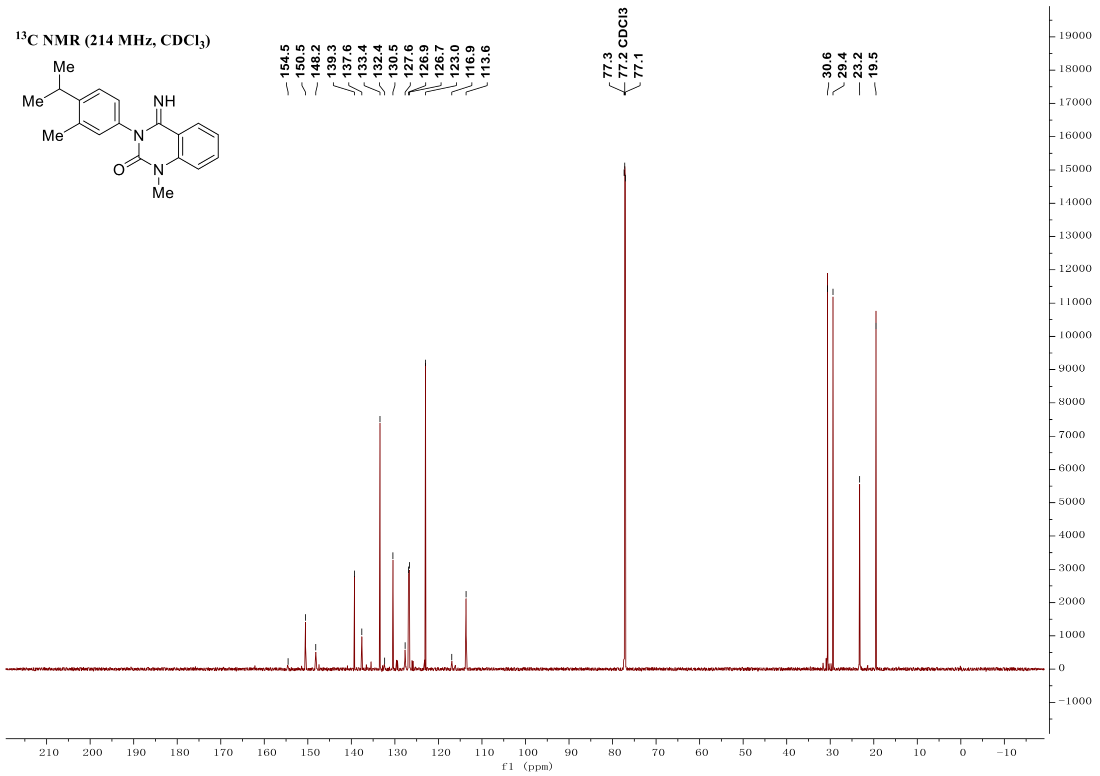

Supplementary Figure 81. ^{1}H NMR and ^{13}C NMR spectra of compound S105.

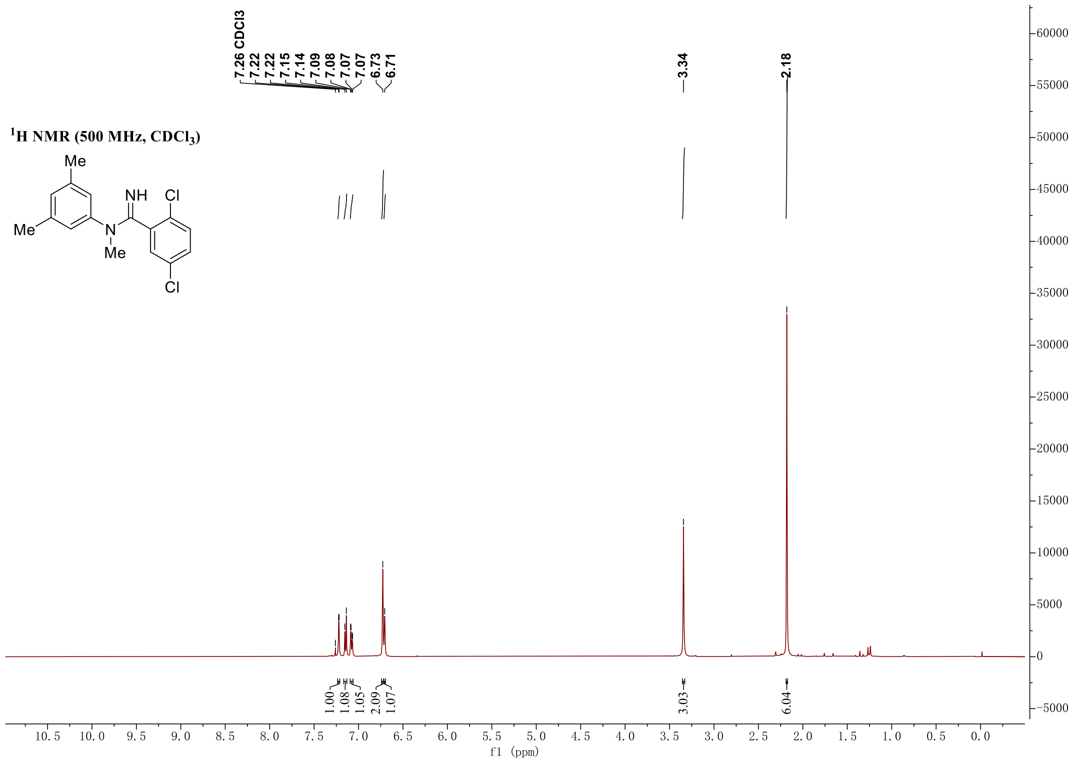

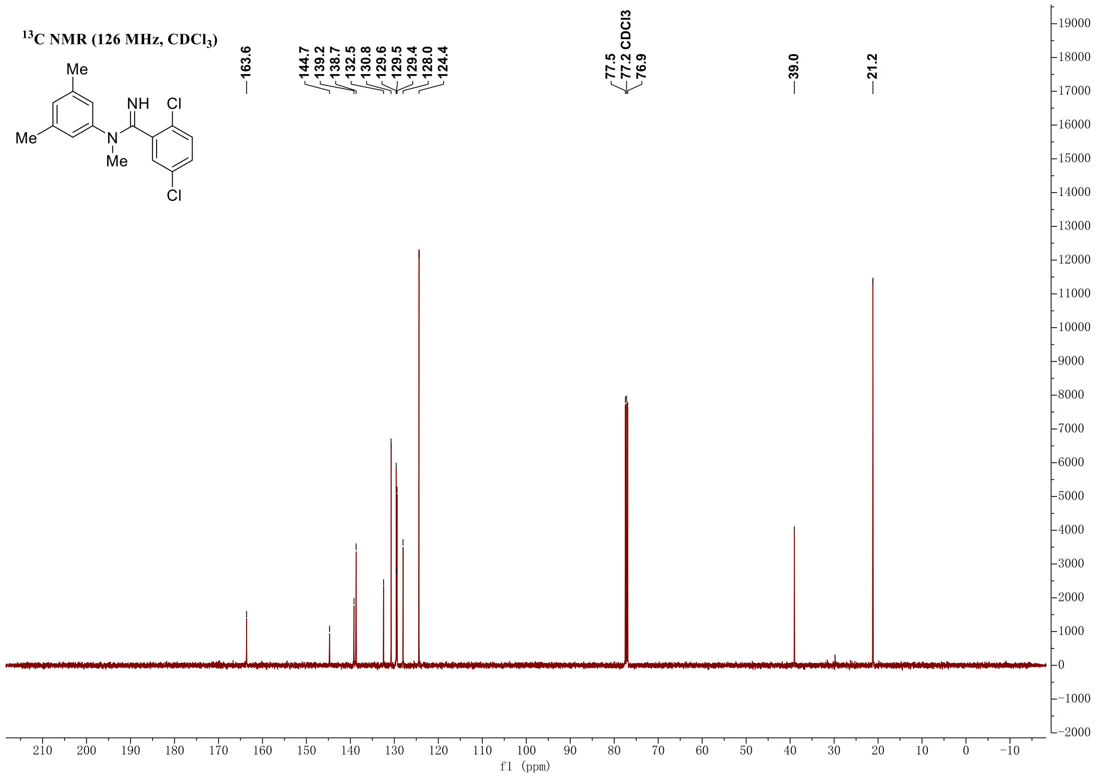

Supplementary Figure 82. ^{1}H NMR and ^{13}C NMR spectra of compound S106.

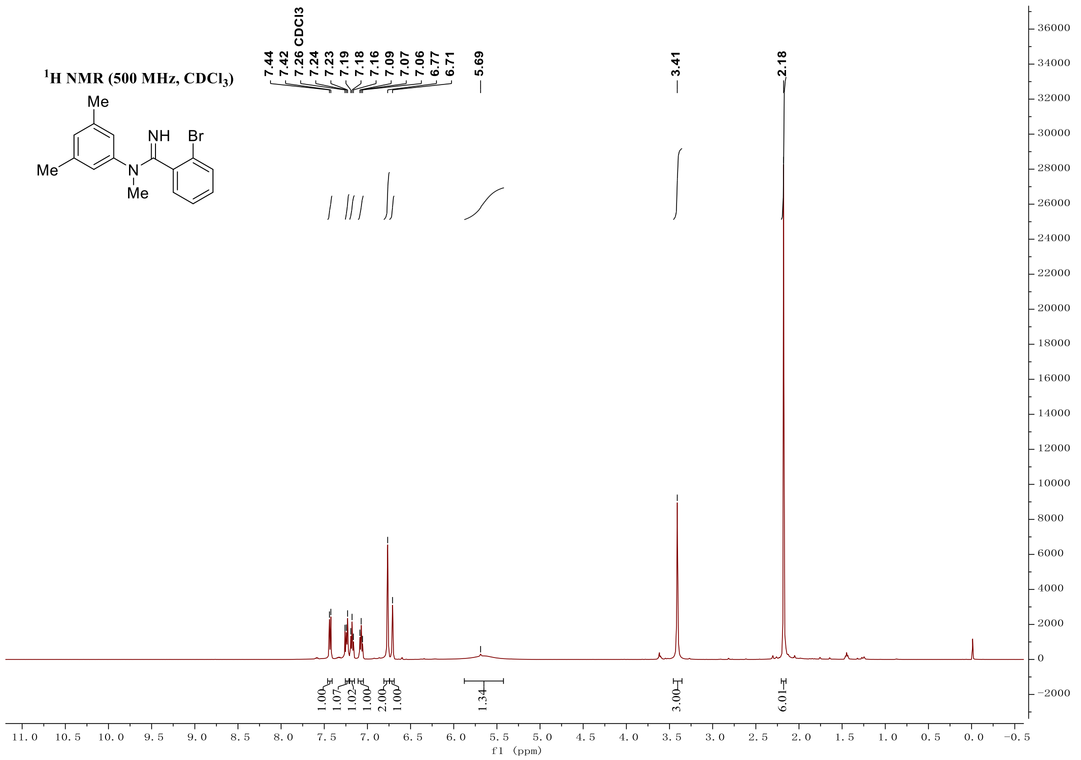

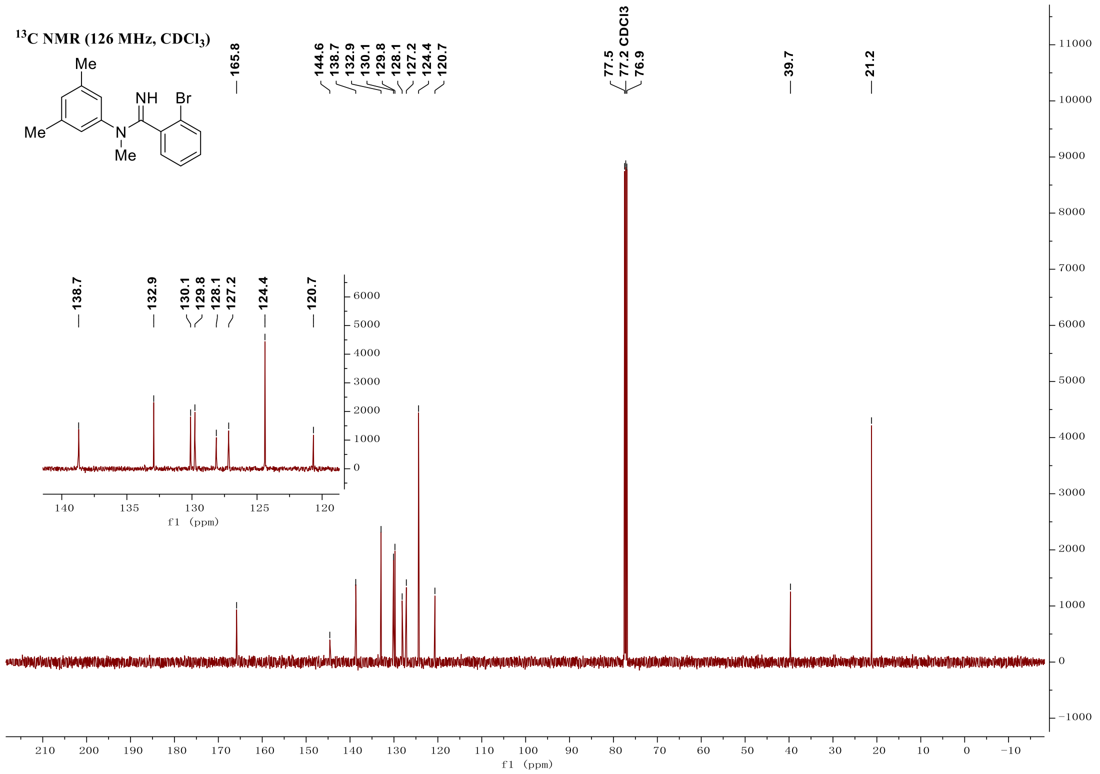

Supplementary Figure 83. ^{1}H NMR and ^{13}C NMR spectra of compound S107.

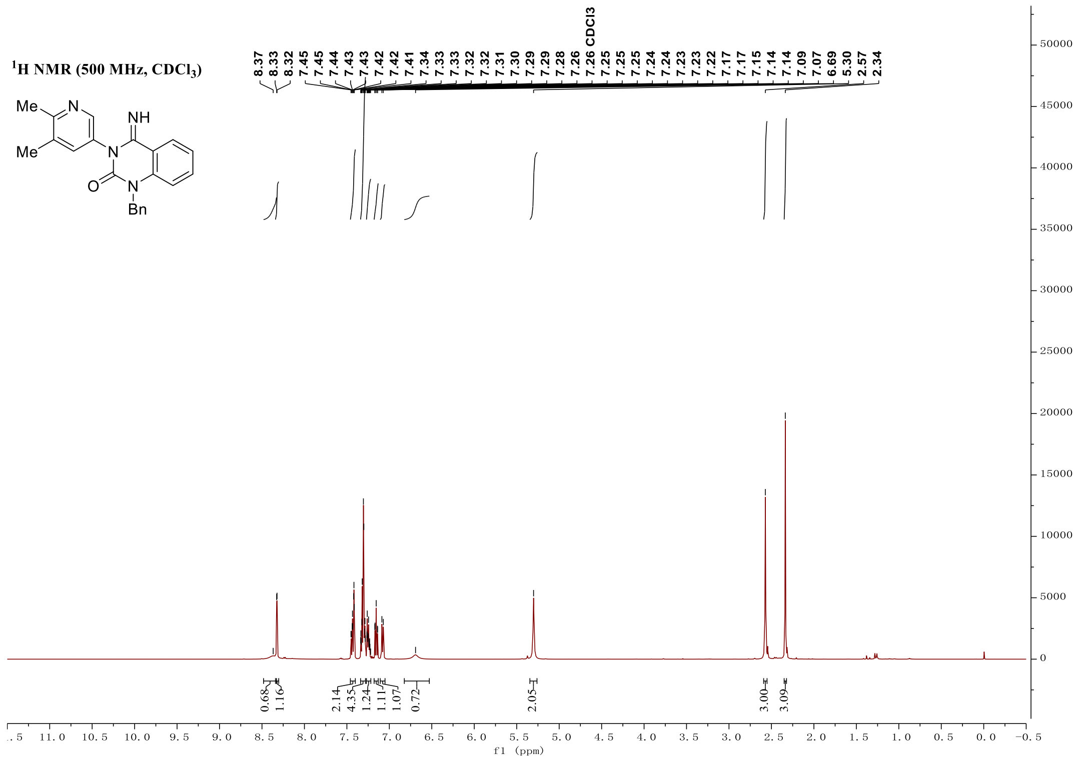

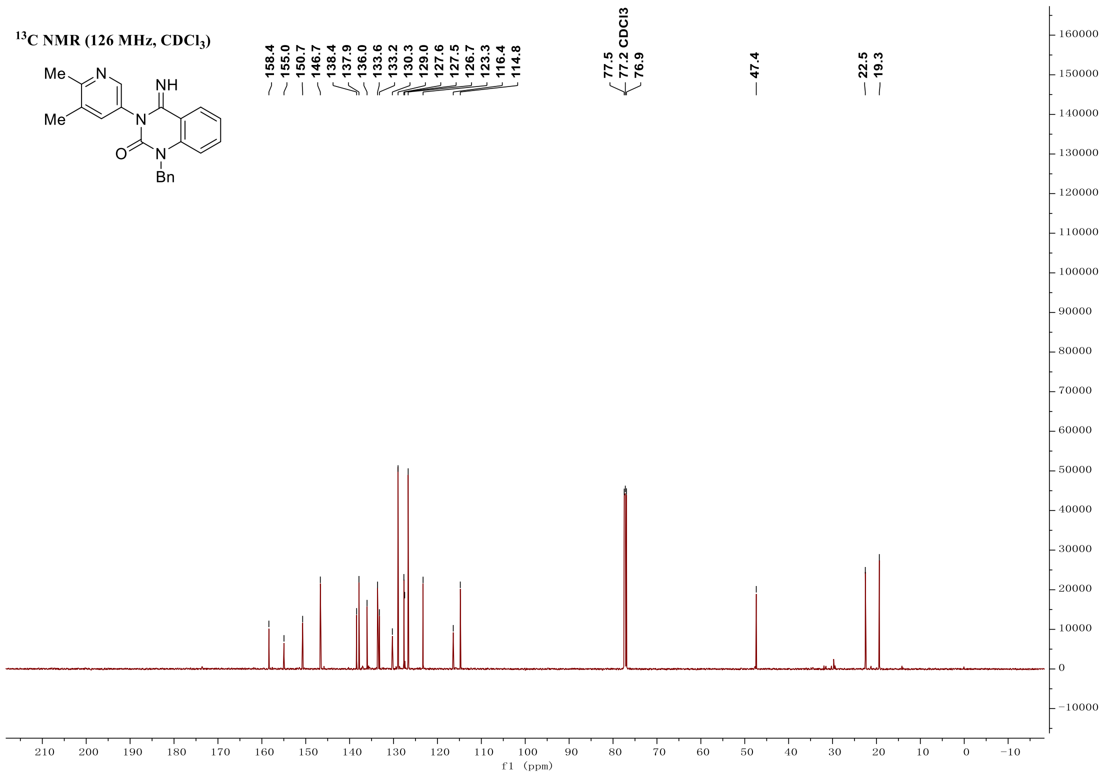

Supplementary Figure 84. ^{1}H NMR and ^{13}C NMR spectra of compound S109.

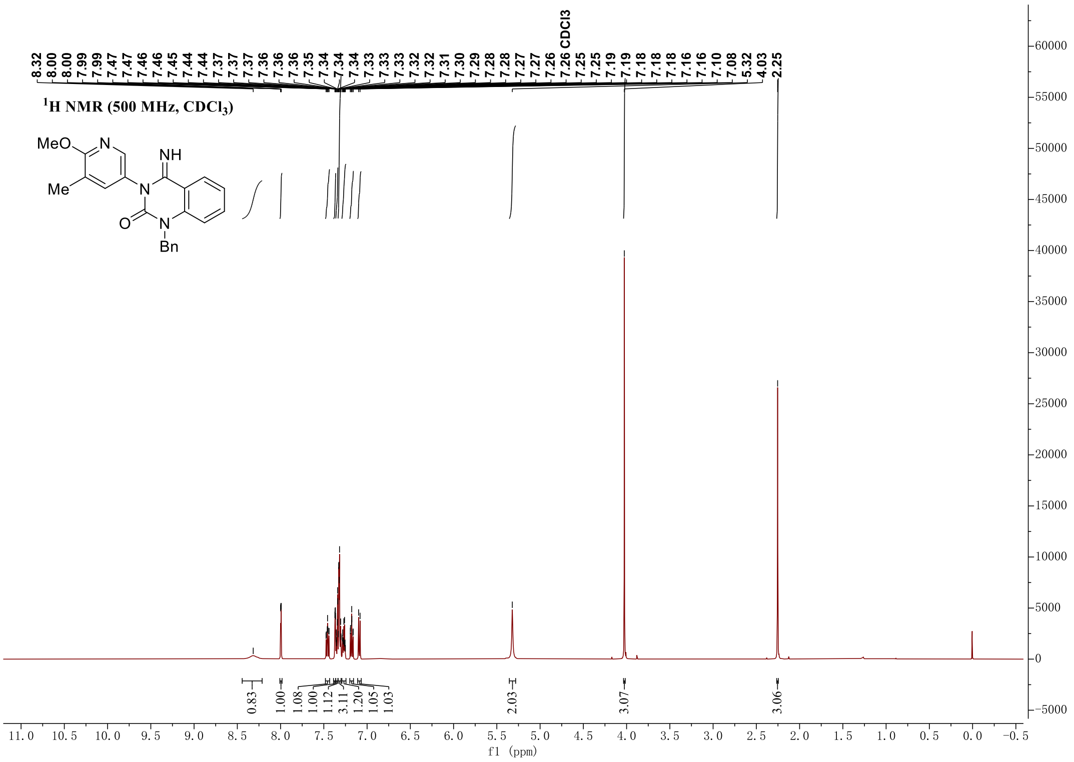

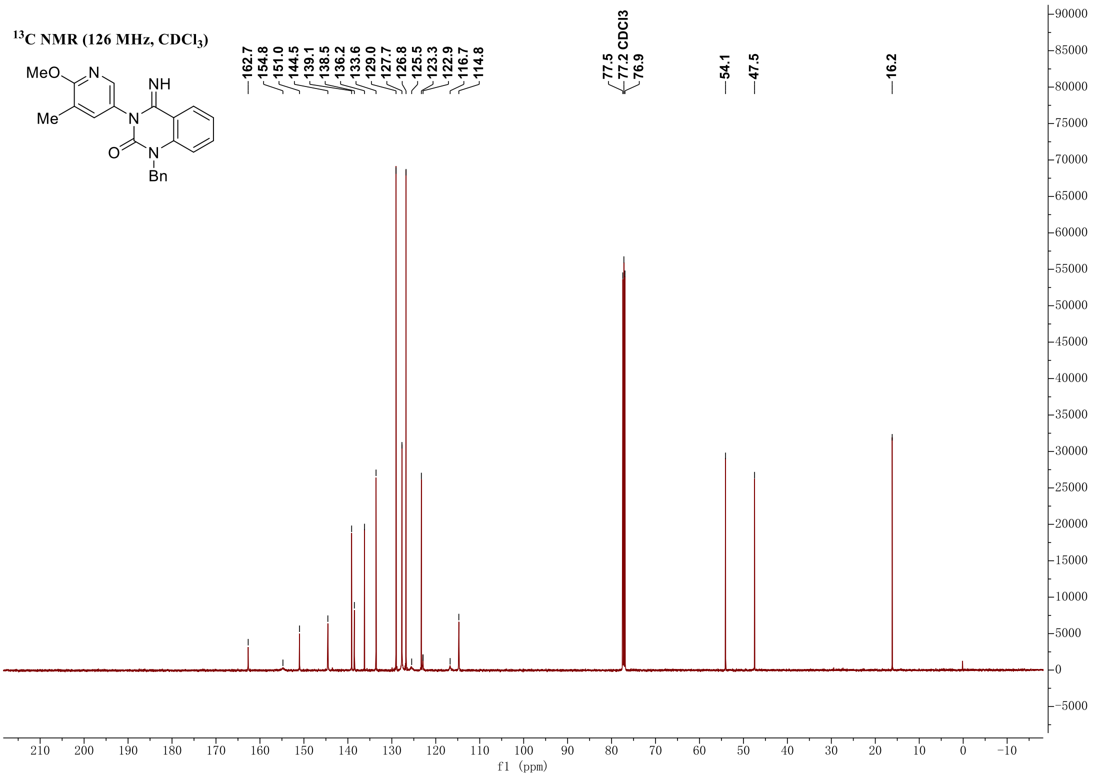

Supplementary Figure 85. ^{1}H NMR and ^{13}C NMR spectra of compound S110.

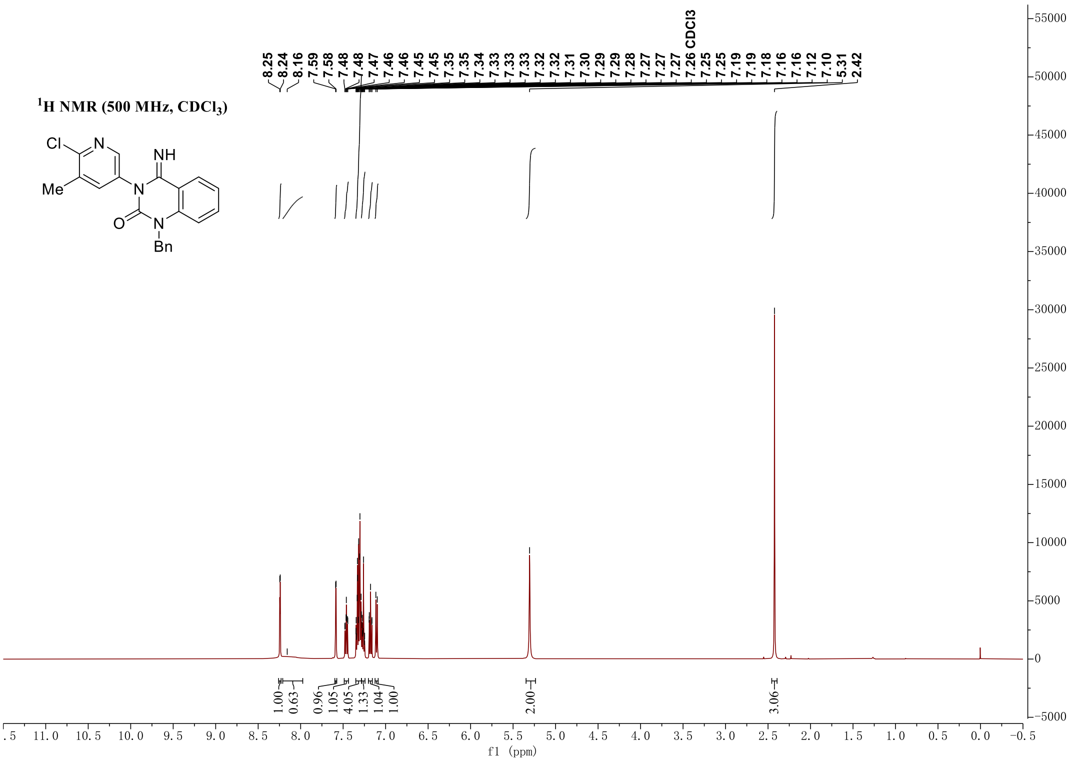

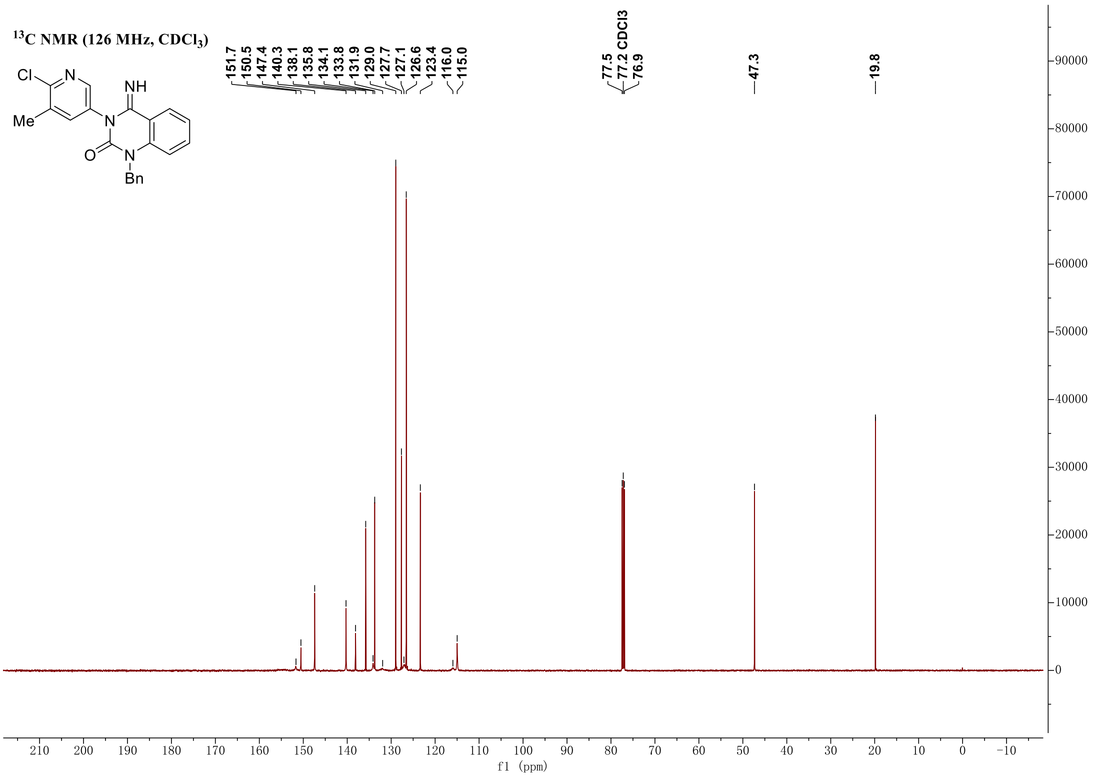

Supplementary Figure 86. ^{1}H NMR and ^{13}C NMR spectra of compound S112.

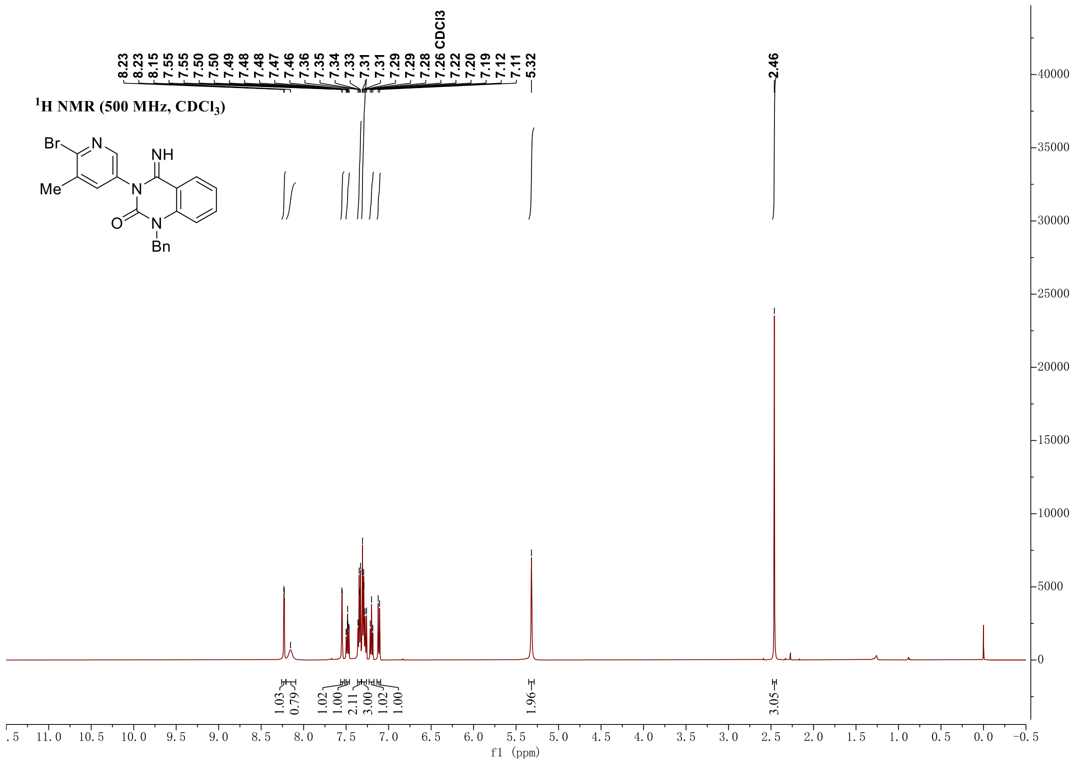

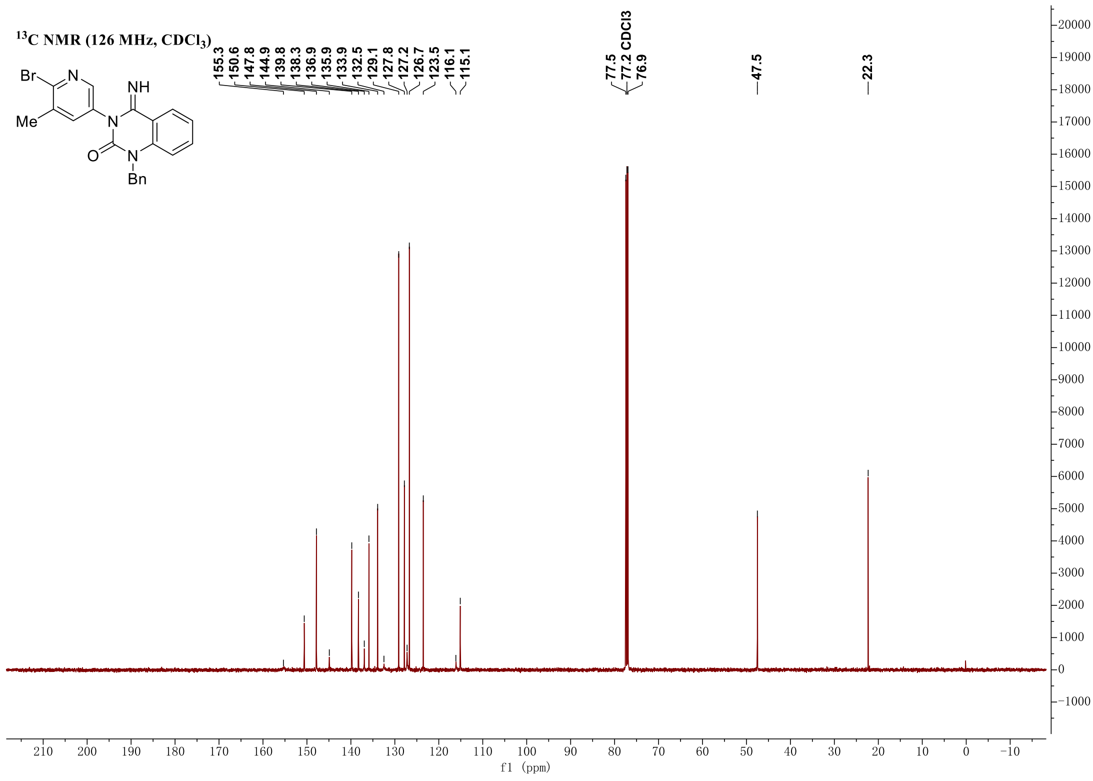

Supplementary Figure 87. ^{1}H NMR and ^{13}C NMR spectra of compound S113.

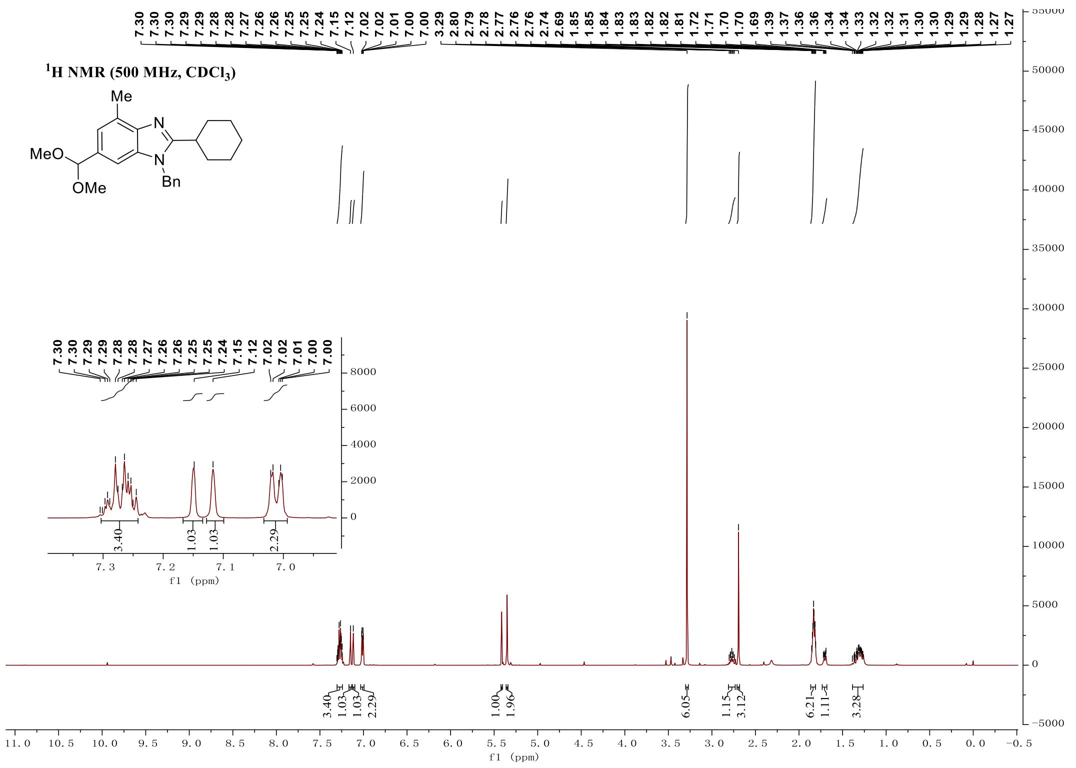

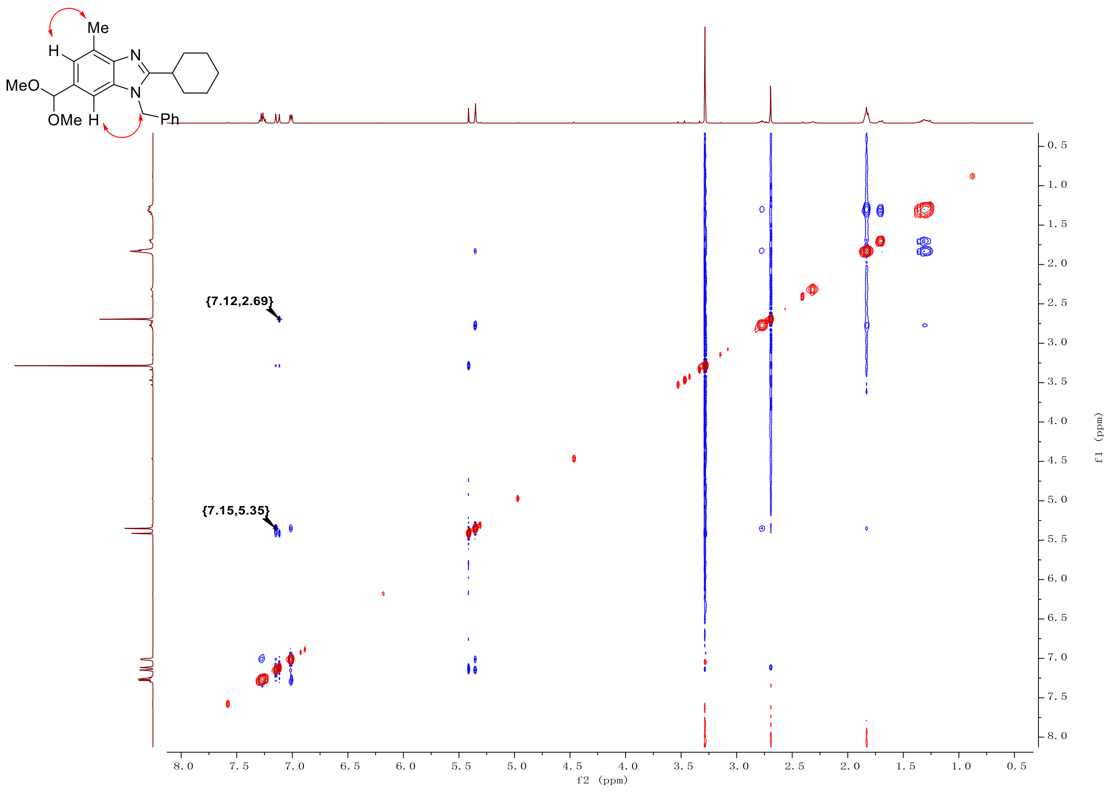

Supplementary Figure 88. **¹H NMR and 2D NOESY spectra of compound 2.**

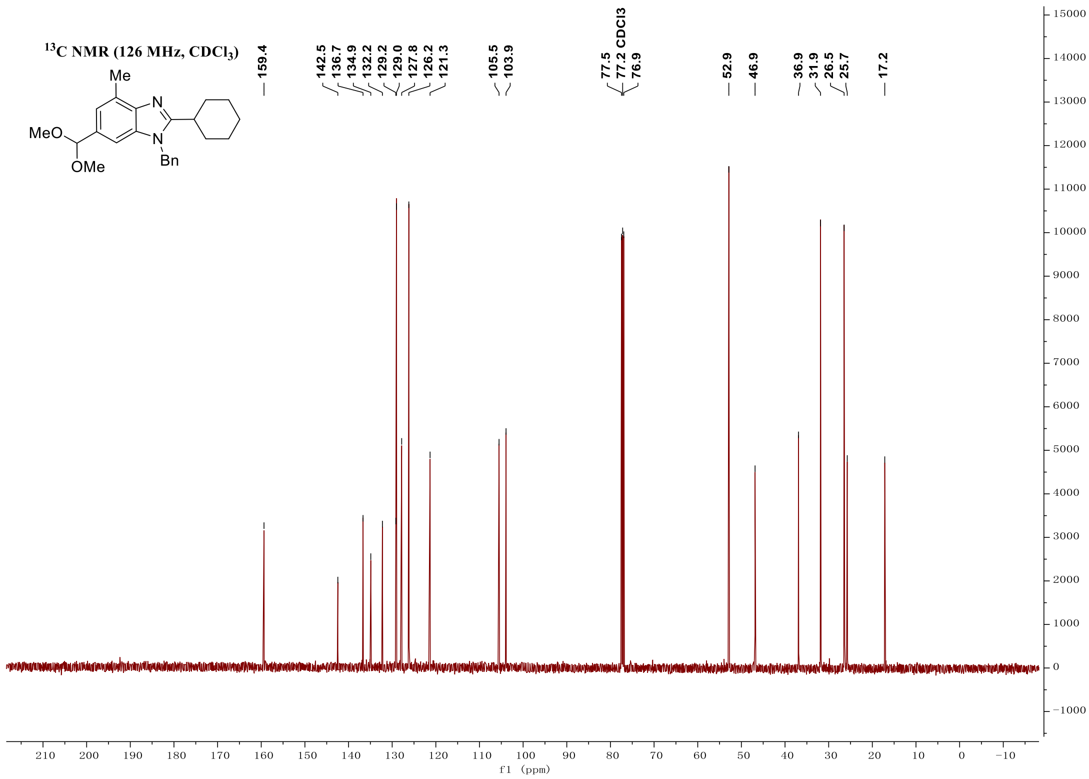

Supplementary Figure 89. ^{13}C NMR spectrum of compound 2.

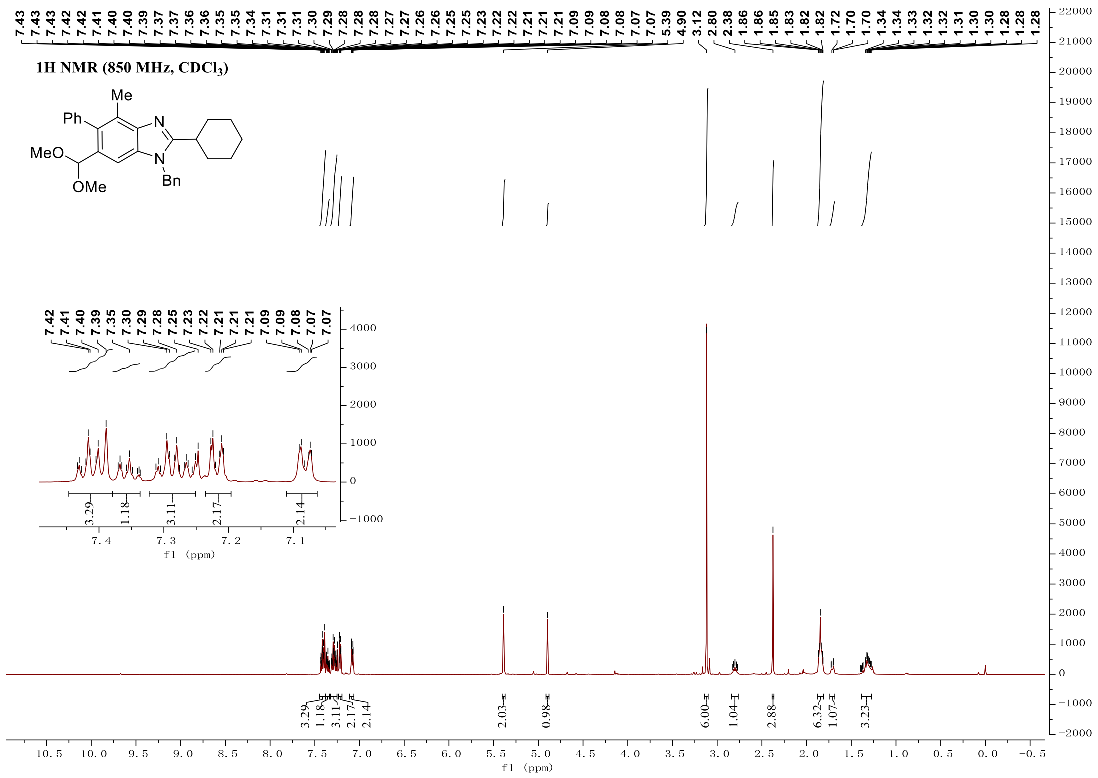

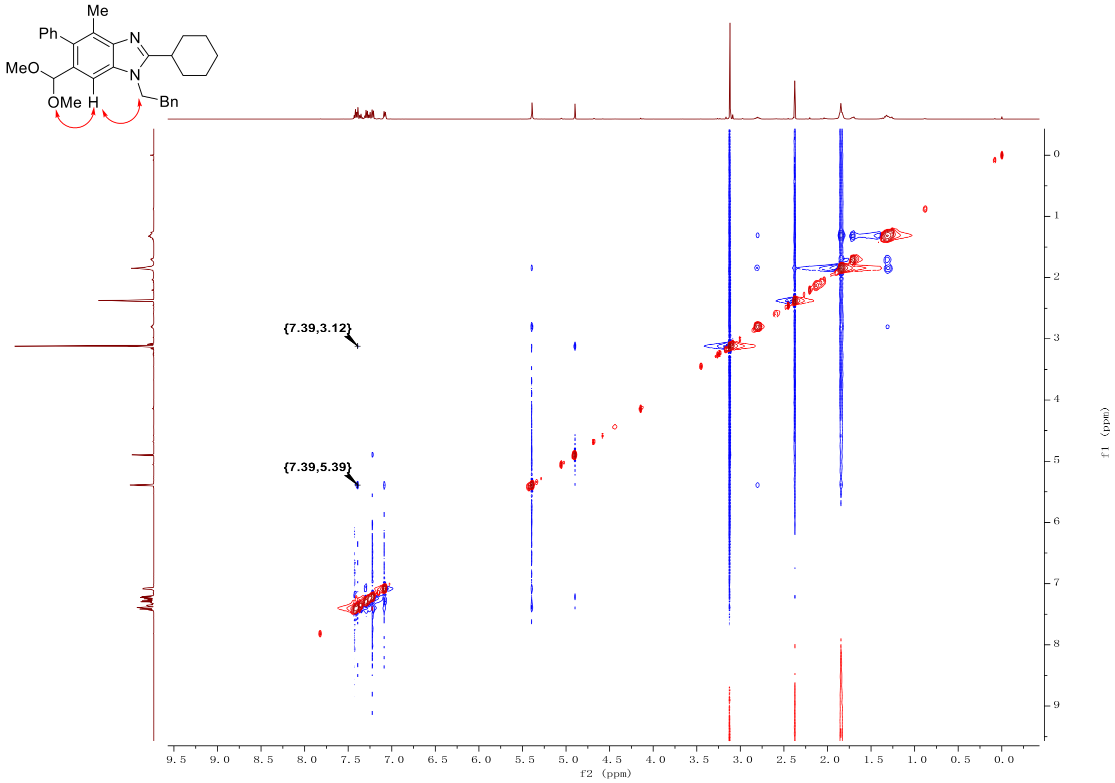

Supplementary Figure 90. ^{1}H NMR and 2D NOESY spectra of compound 3.

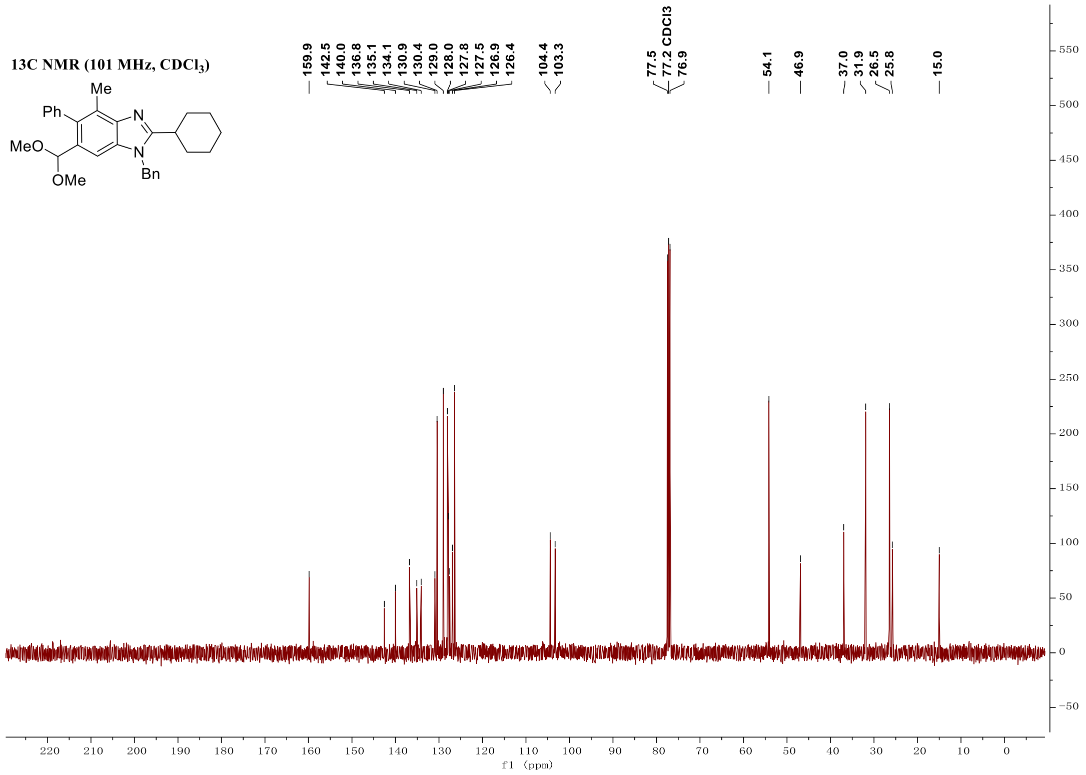

Supplementary Figure 91. ^{13}C NMR spectrum of compound 3.

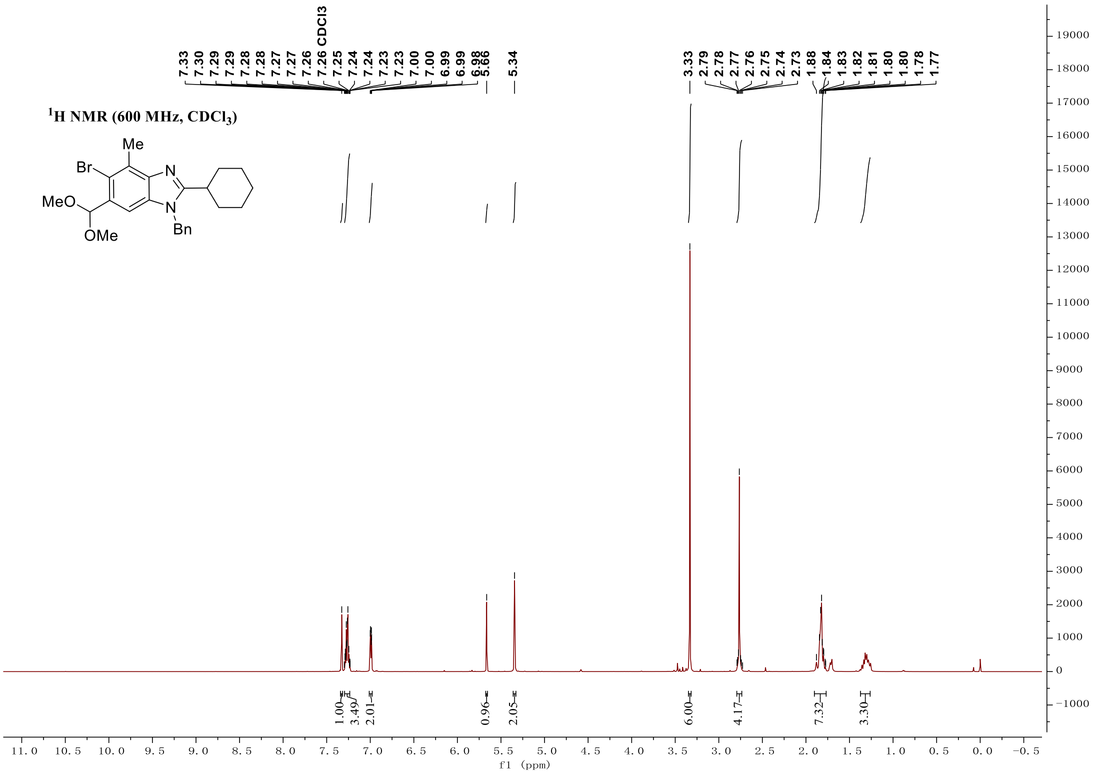

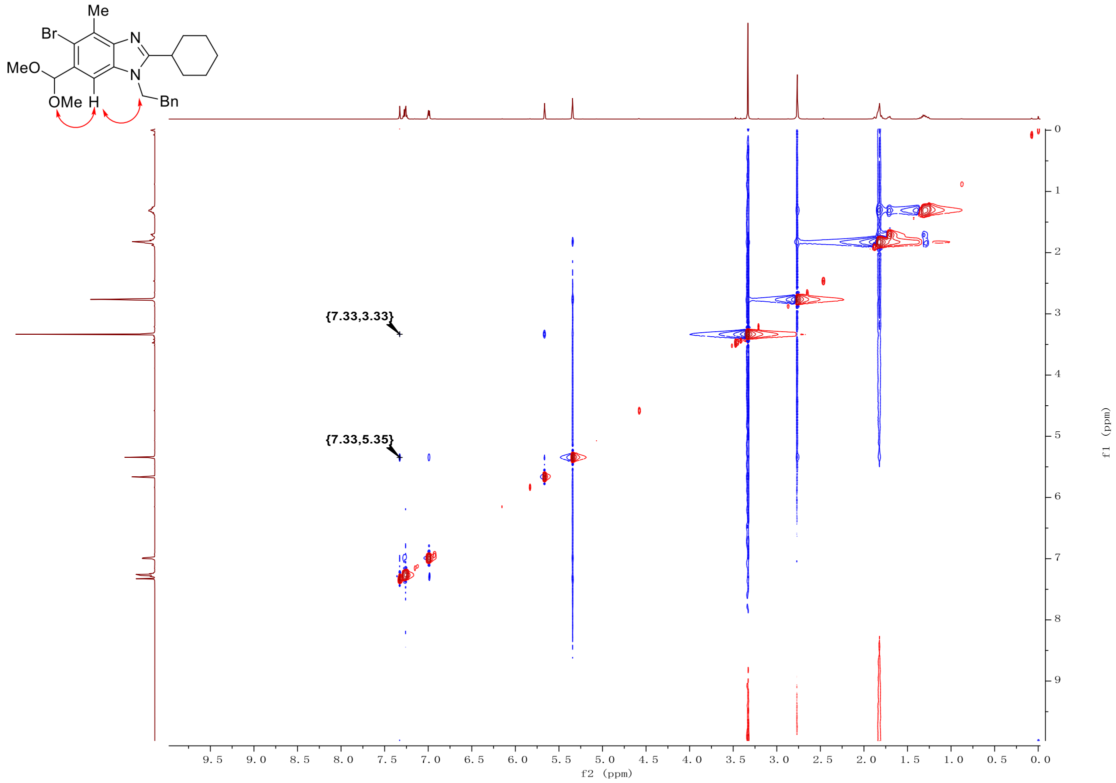

Supplementary Figure 92. ^{1}H NMR and 2D NOESY spectra of compound 4.

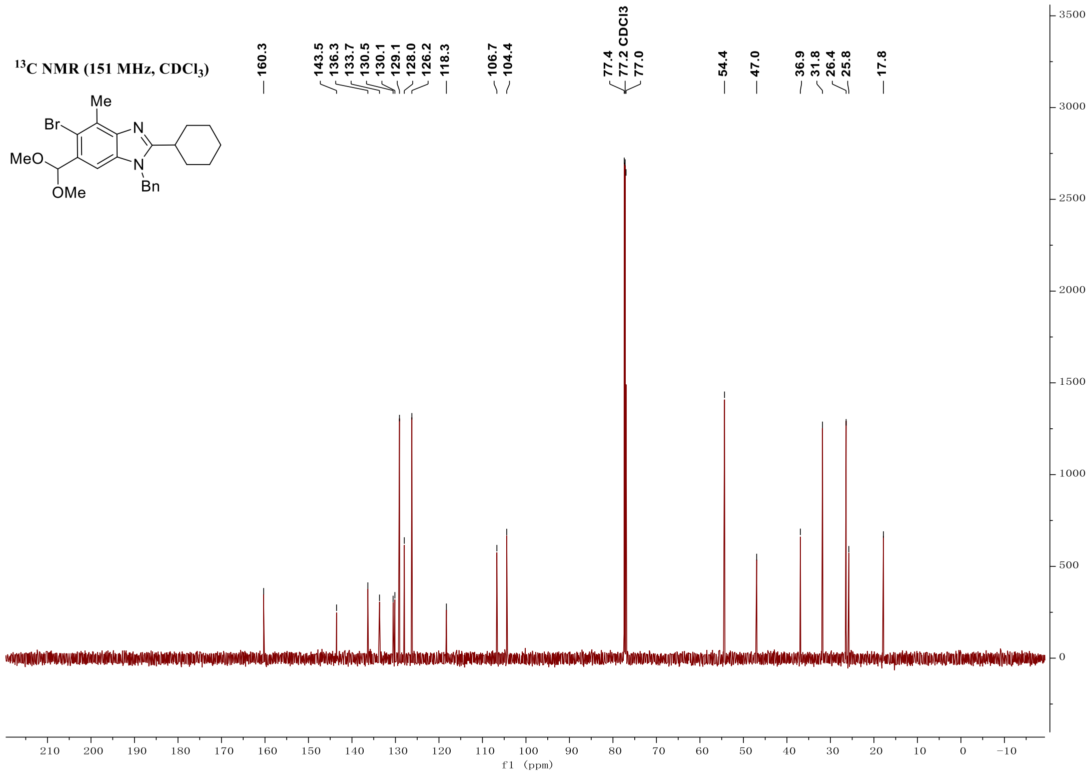

Supplementary Figure 93. ^{13}C NMR spectrum of compound 4.

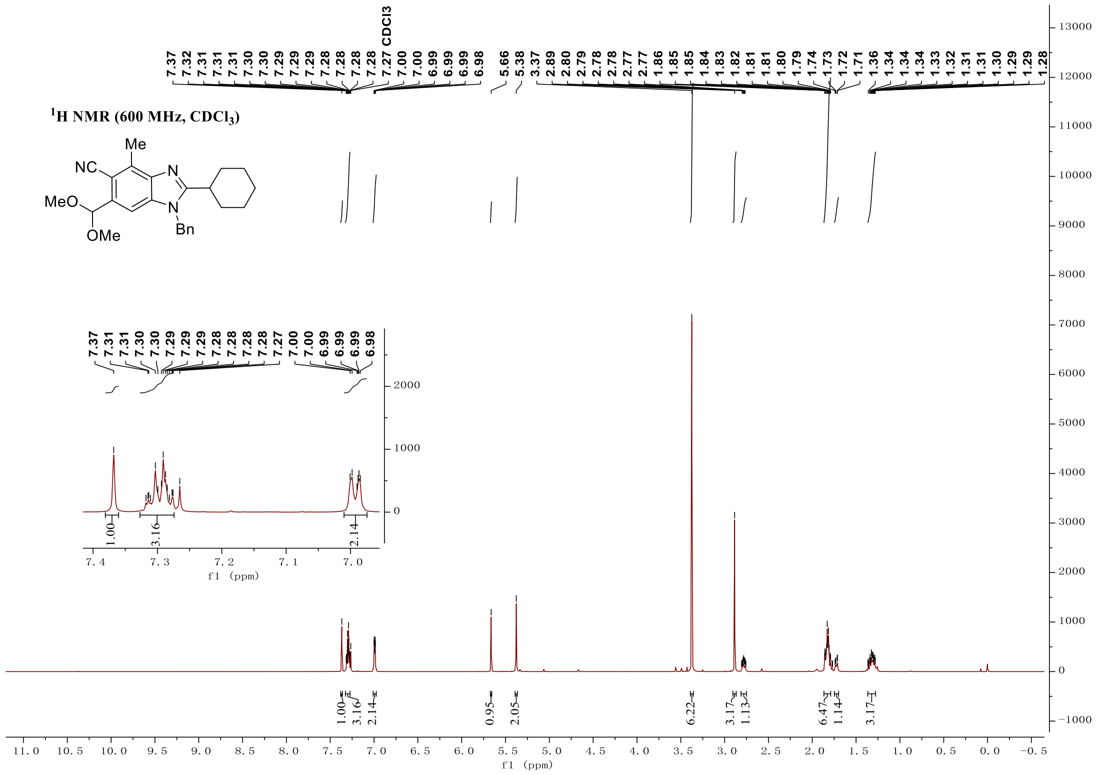

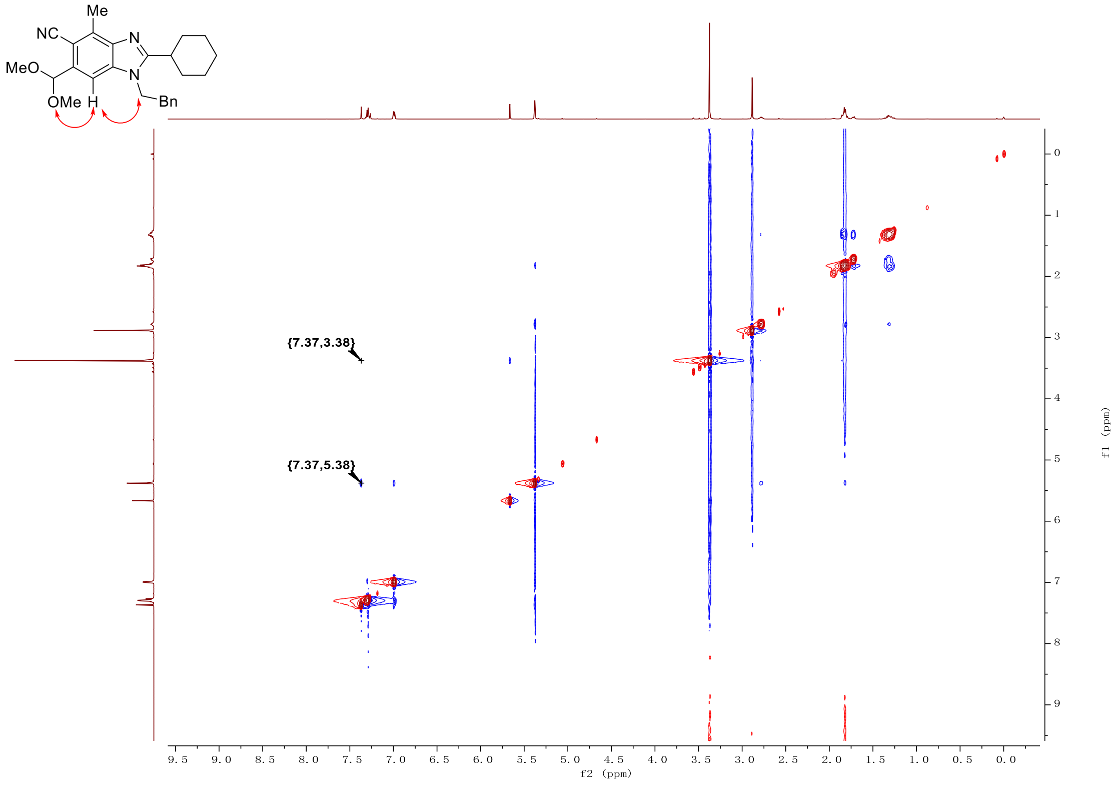

Supplementary Figure 94. ^{1}H NMR and 2D NOESY spectra of compound 5.

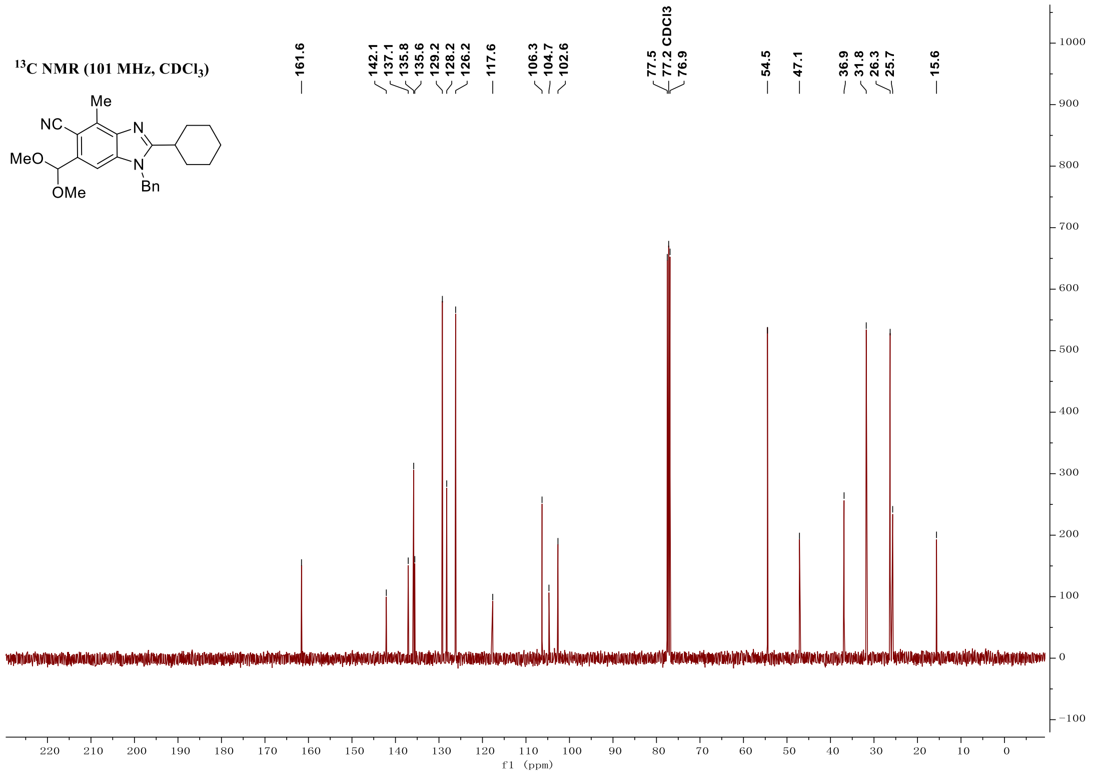

Supplementary Figure 95. ^{13}C NMR spectrum of compound 5.

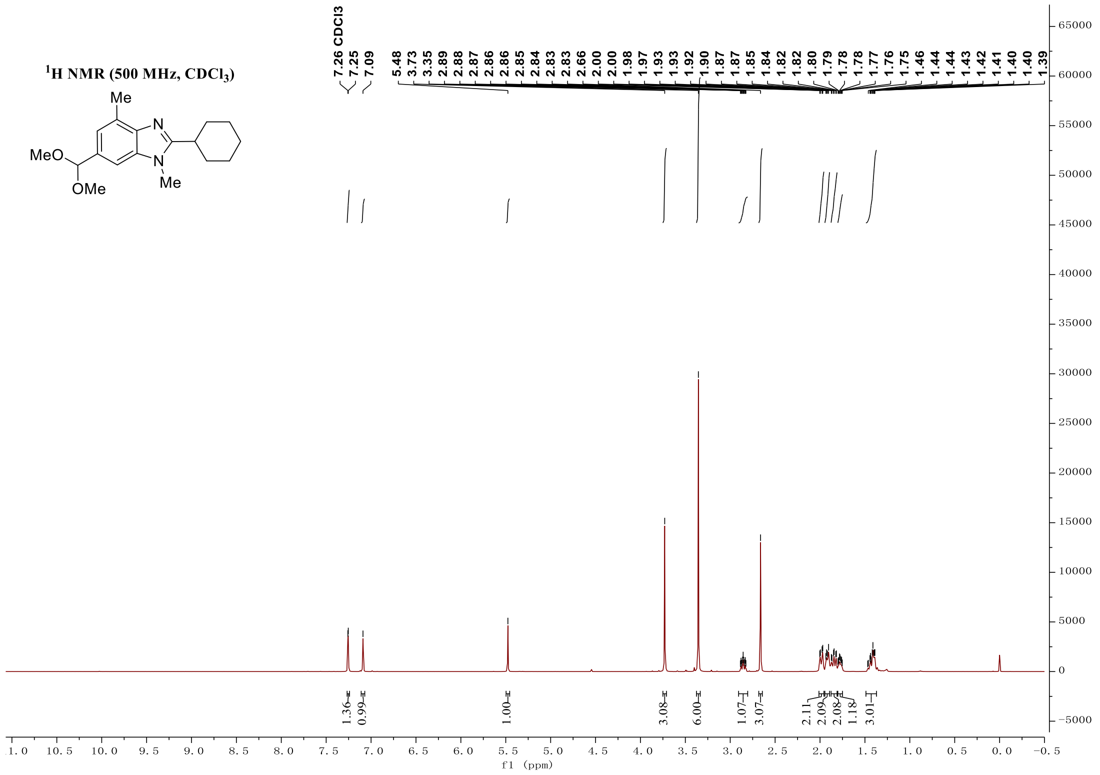

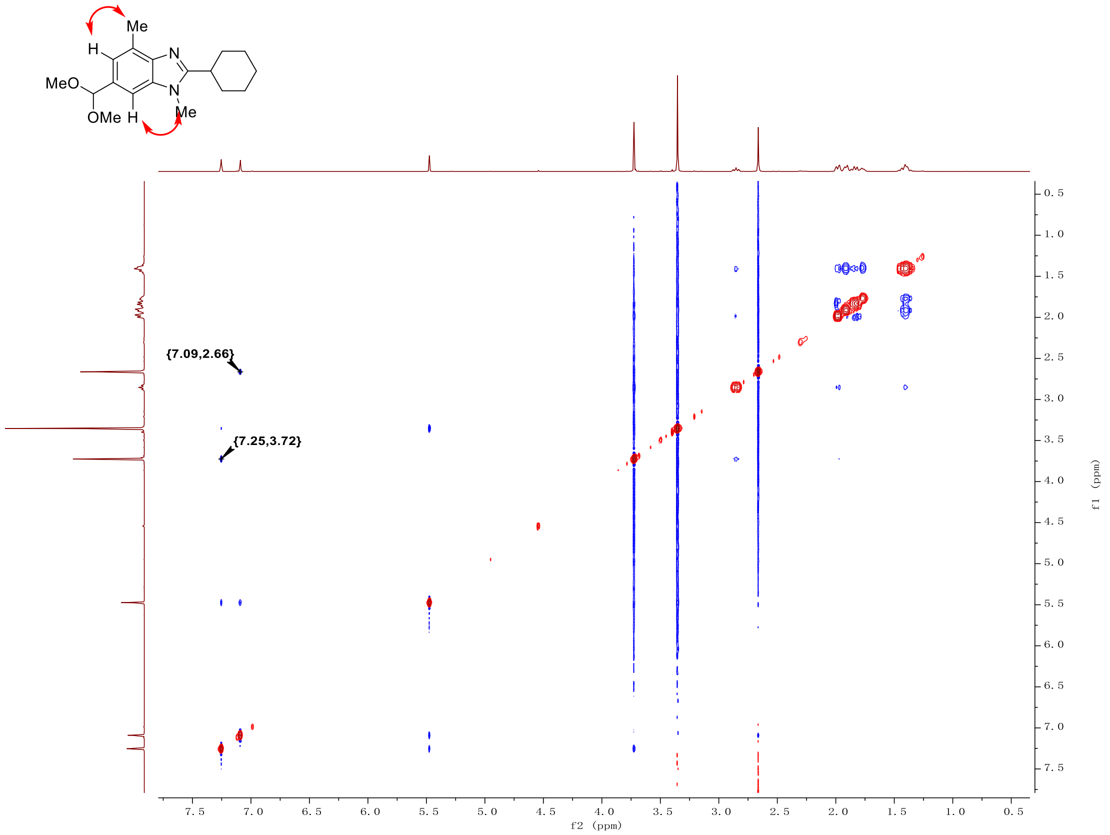

Supplementary Figure 96. ^{1}H NMR and 2D NOESY spectra of compound 6.

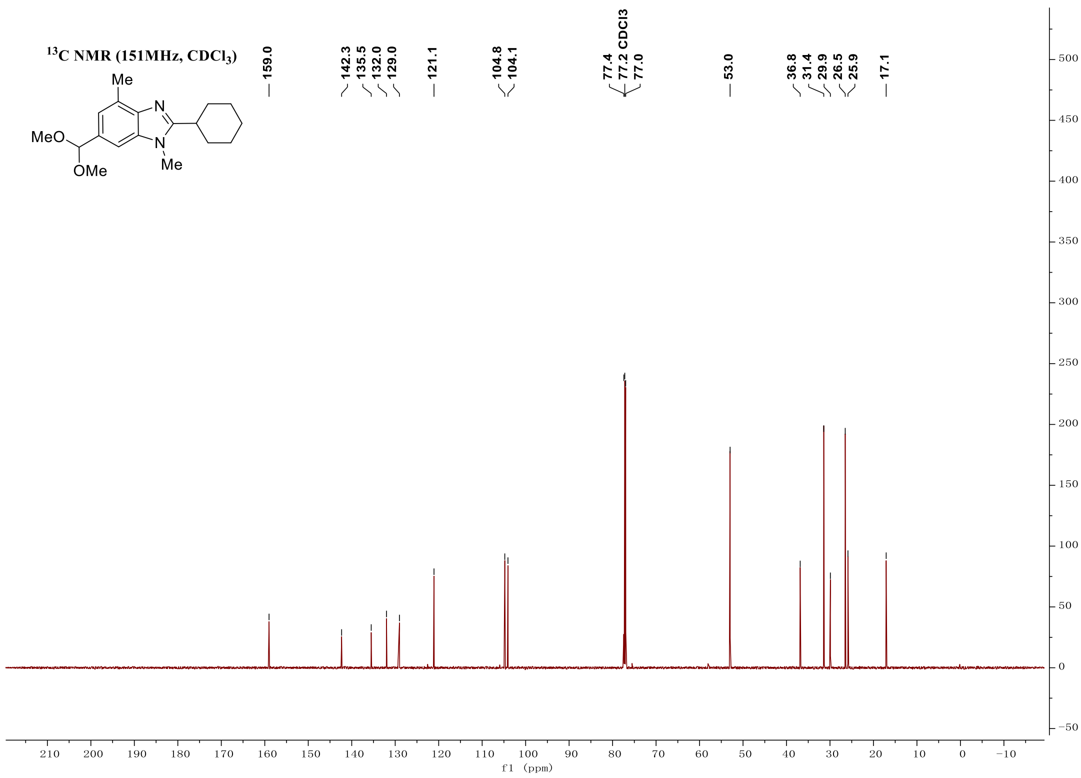

Supplementary Figure 97. ^{13}C NMR spectrum of compound 6.

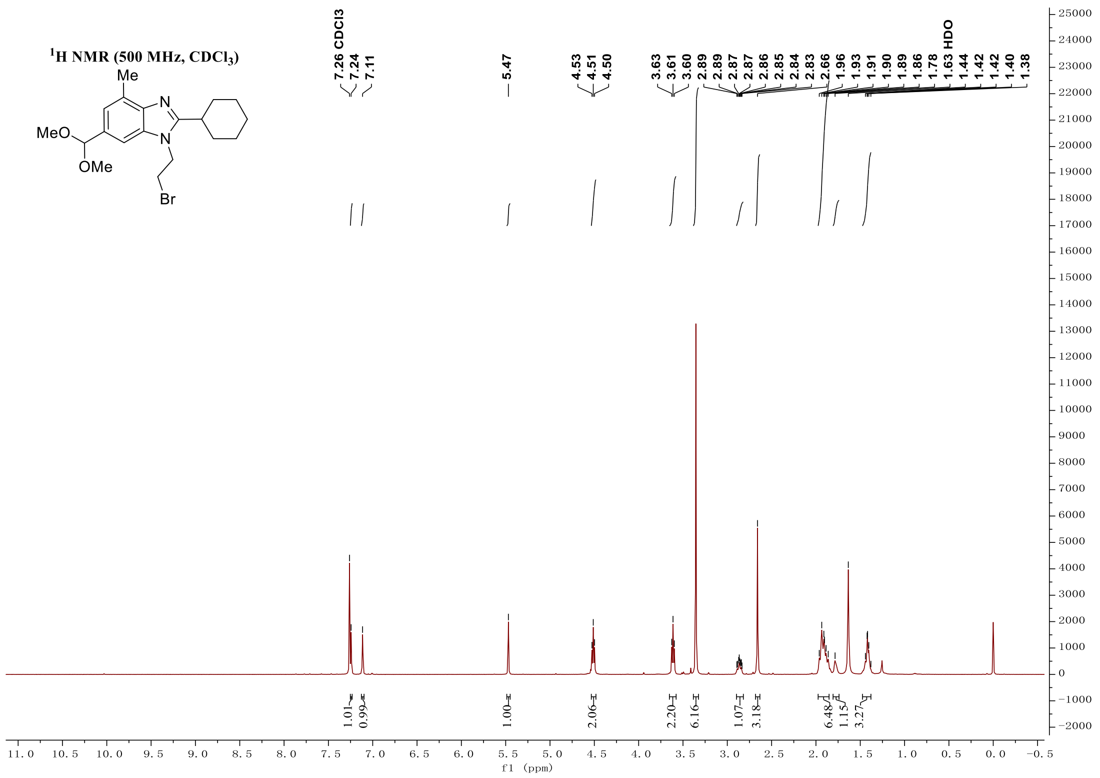

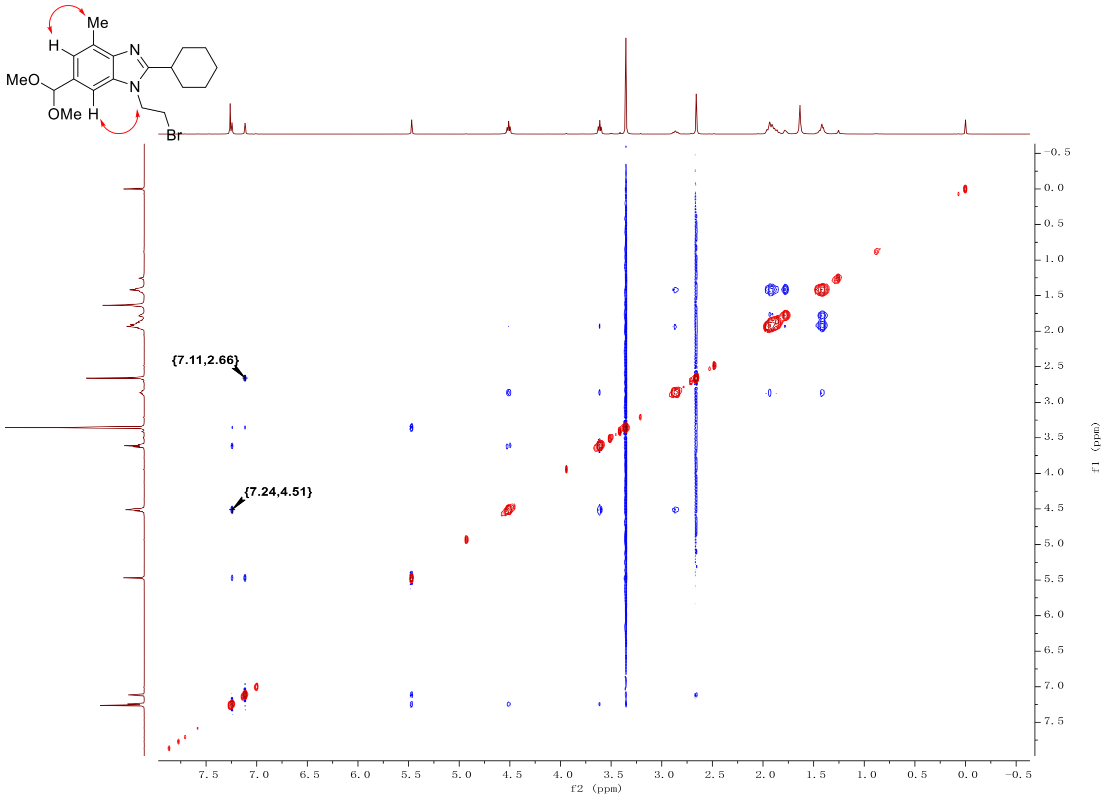

Supplementary Figure 98. **^{1}H NMR and 2D NOESY spectra of compound 7.**

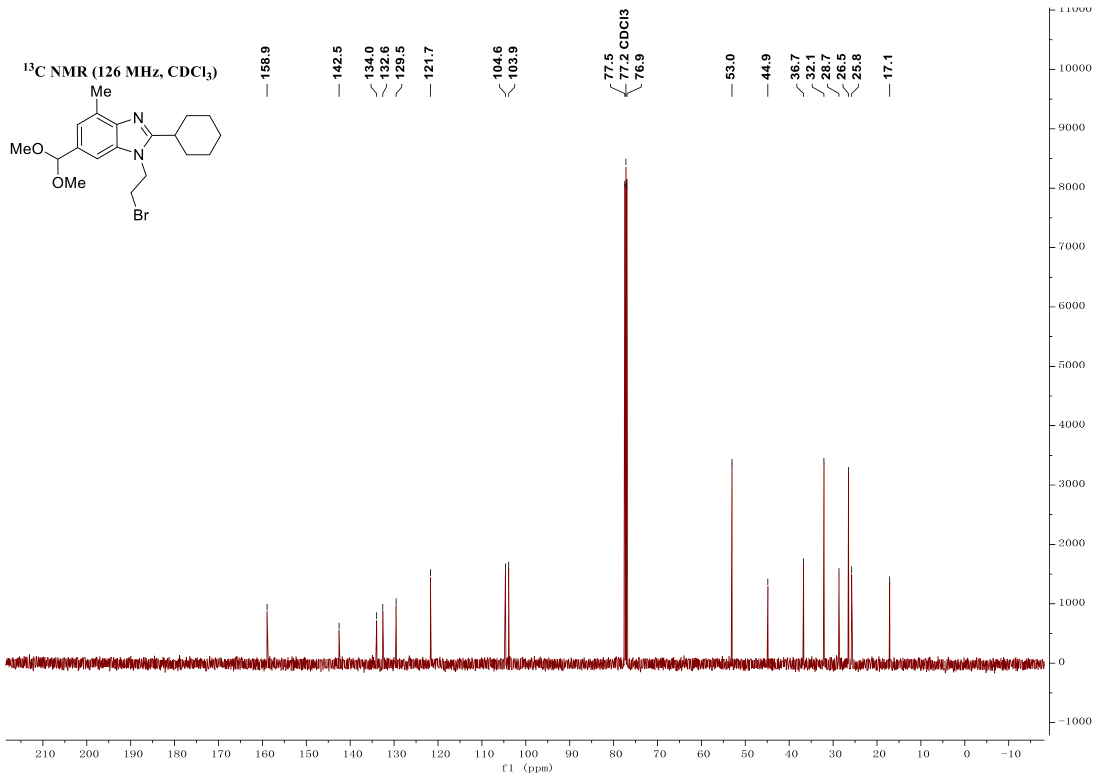

Supplementary Figure 99. ^{13}C NMR spectrum of compound 7.

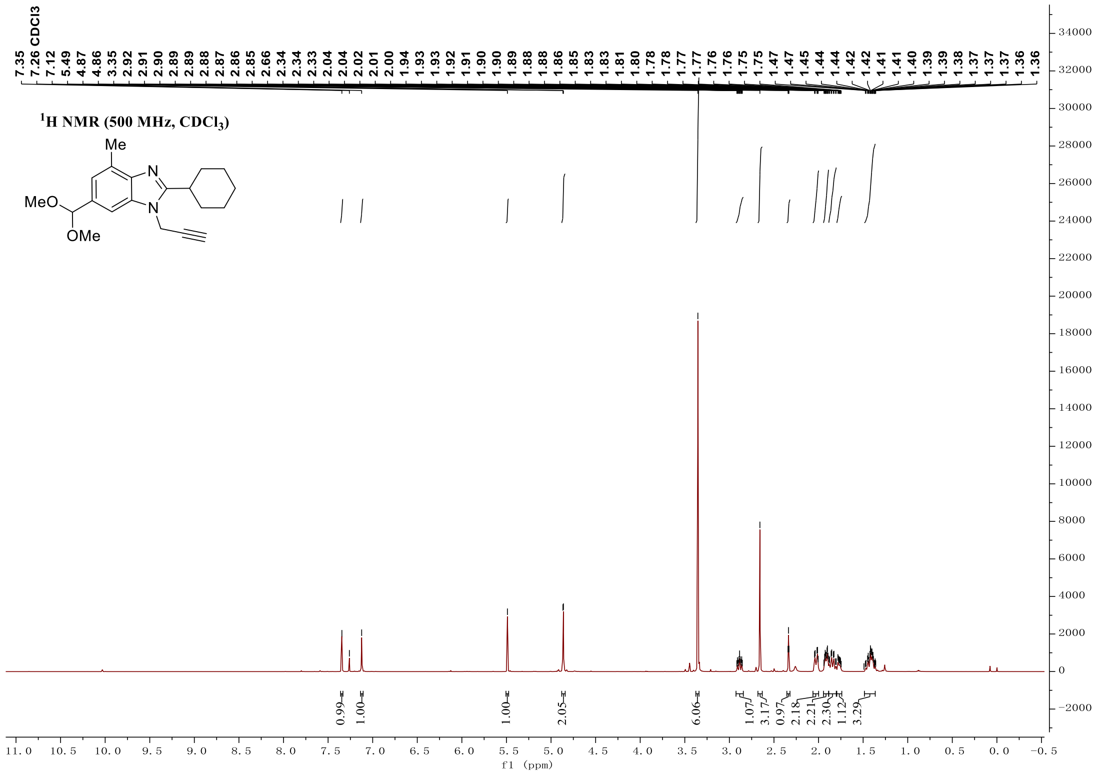

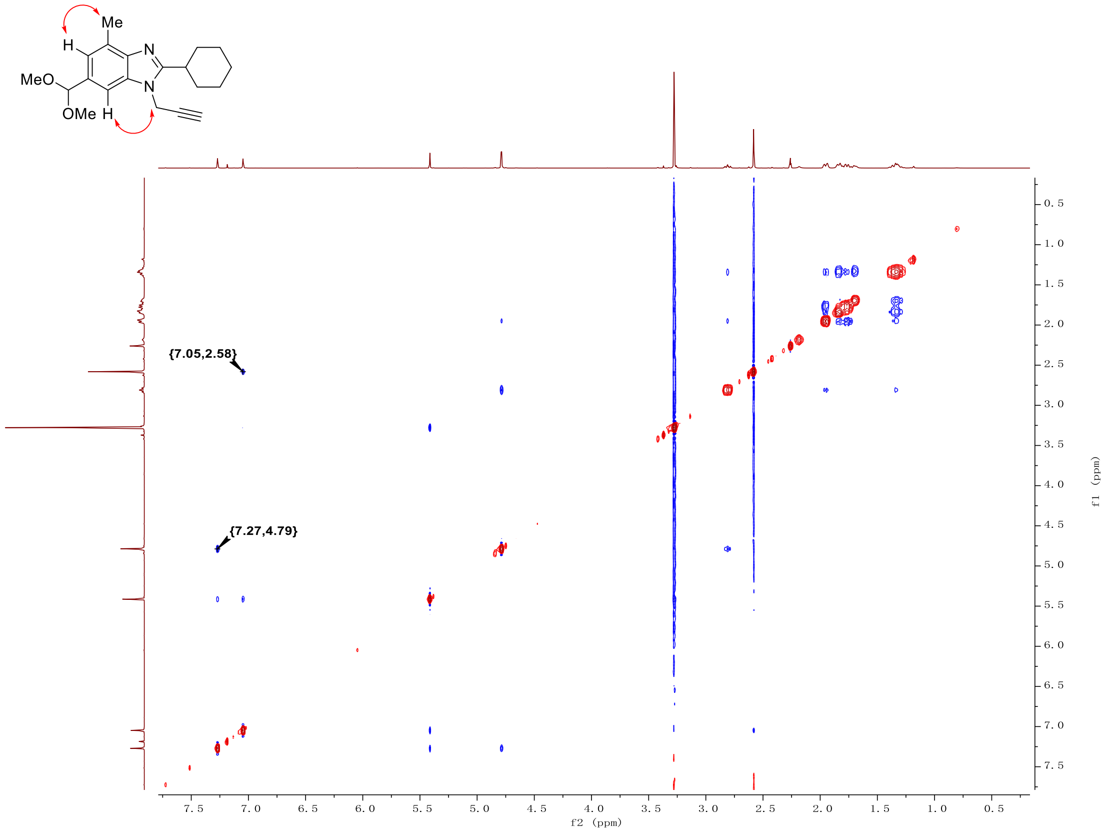

Supplementary Figure 100. ^{1}H NMR and 2D NOESY spectra of compound 8.

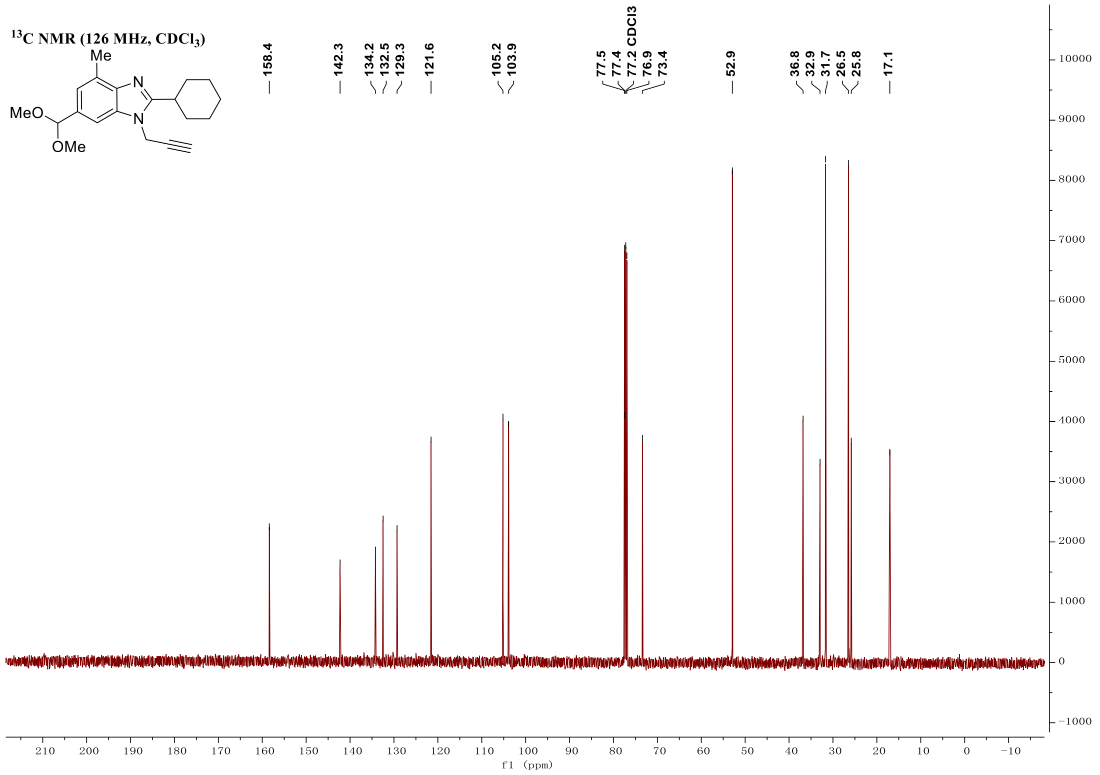

Supplementary Figure 101. ^{13}C NMR spectrum of compound 8.

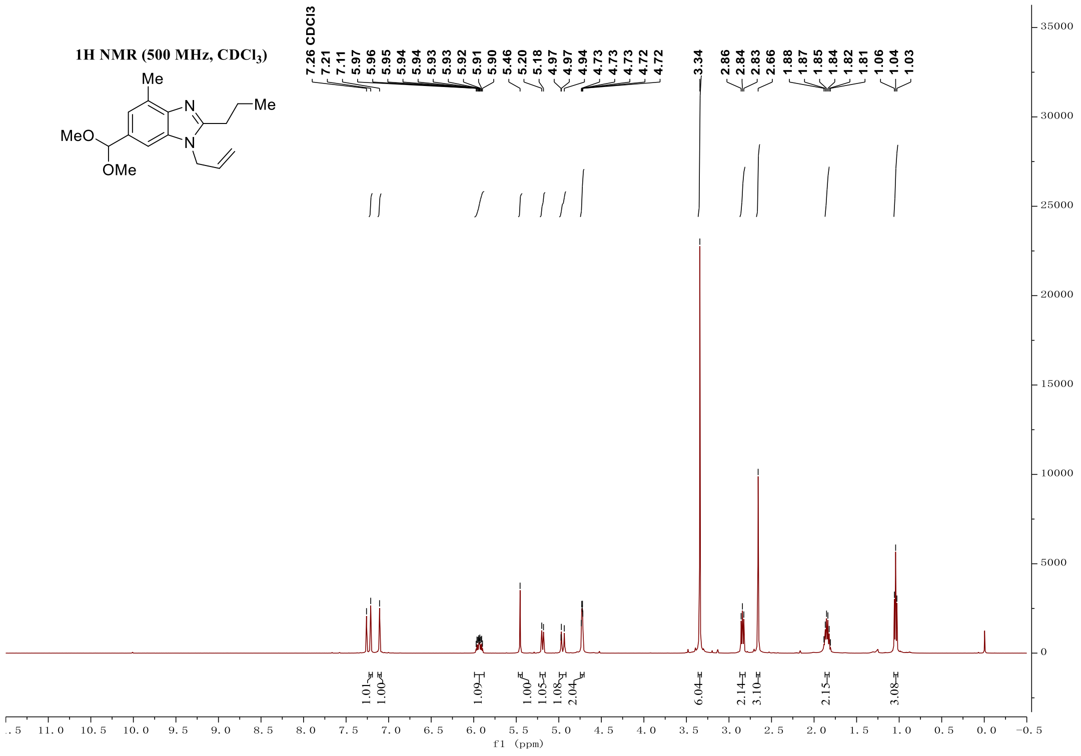

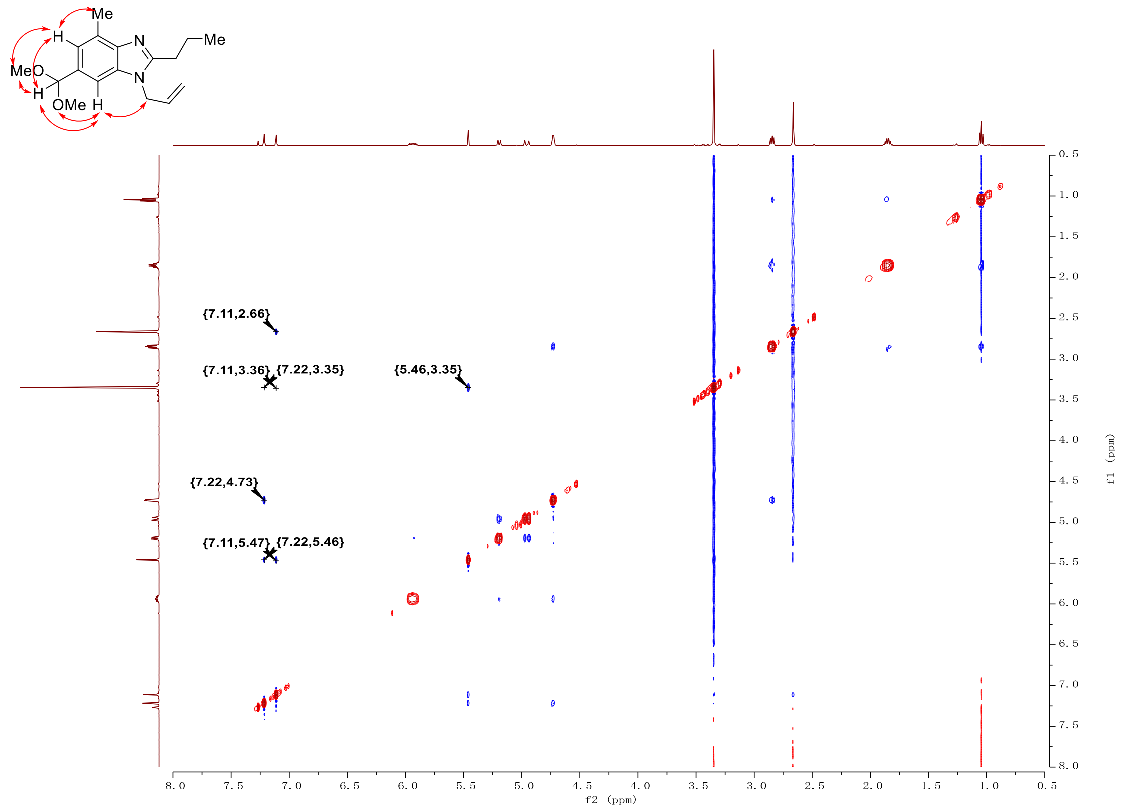

Supplementary Figure 102. ^{1}H NMR and 2D NOESY spectra of compound 9.

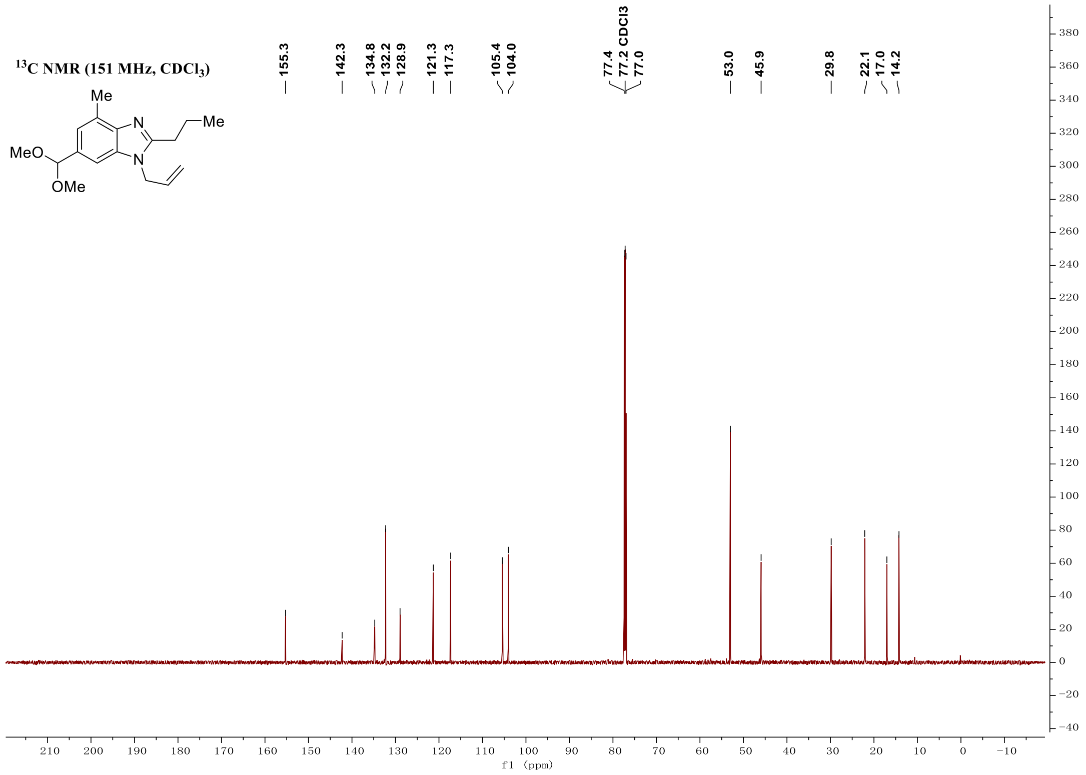

Supplementary Figure 103. ^{13}C NMR spectrum of compound 9.

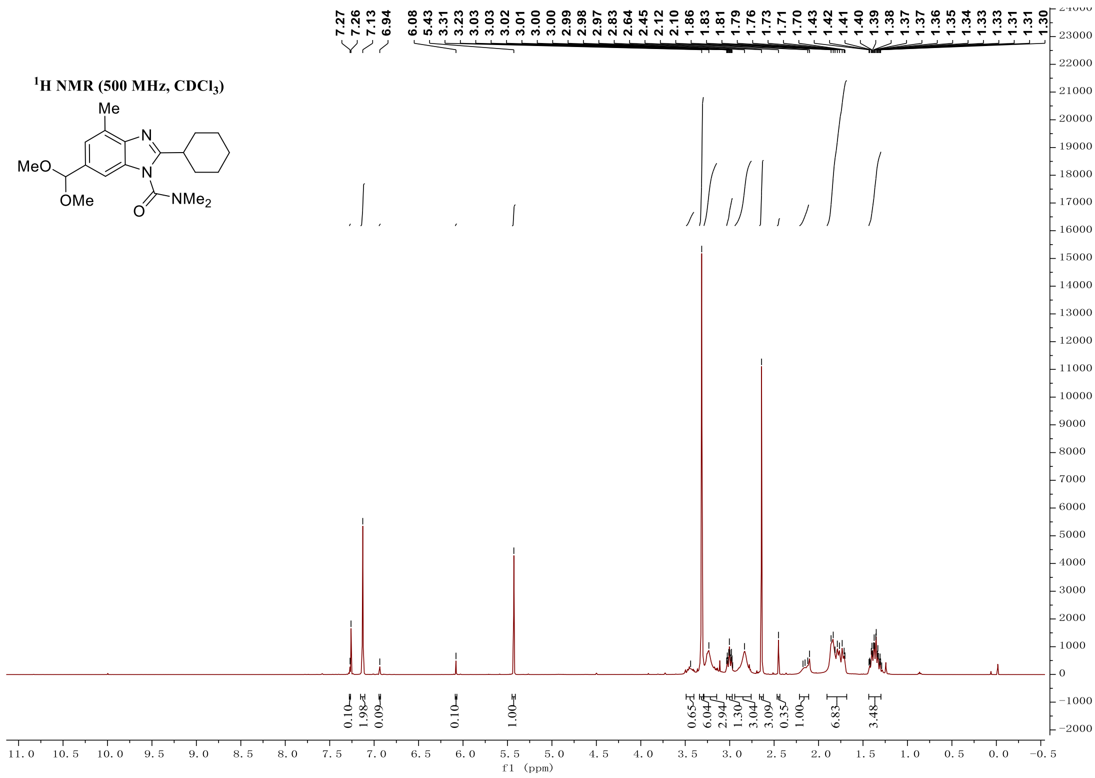

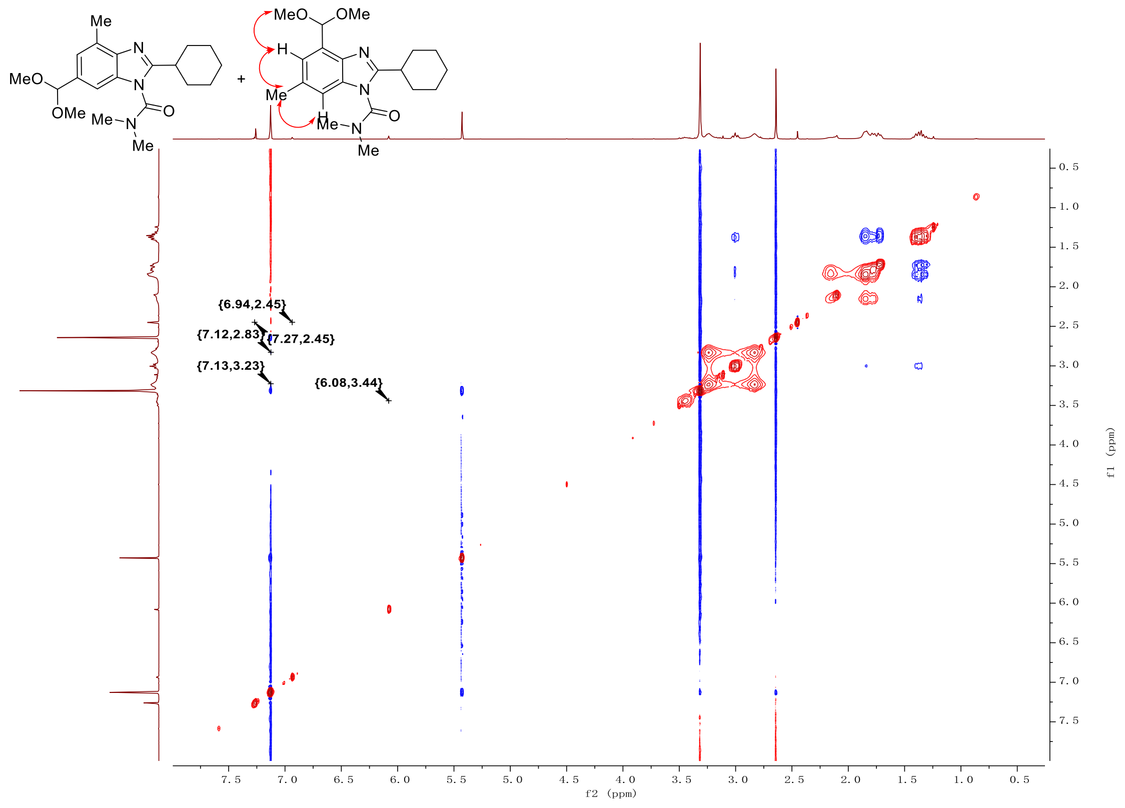

Supplementary Figure 104. ^{1}H NMR and 2D NOESY spectra of compound 10.

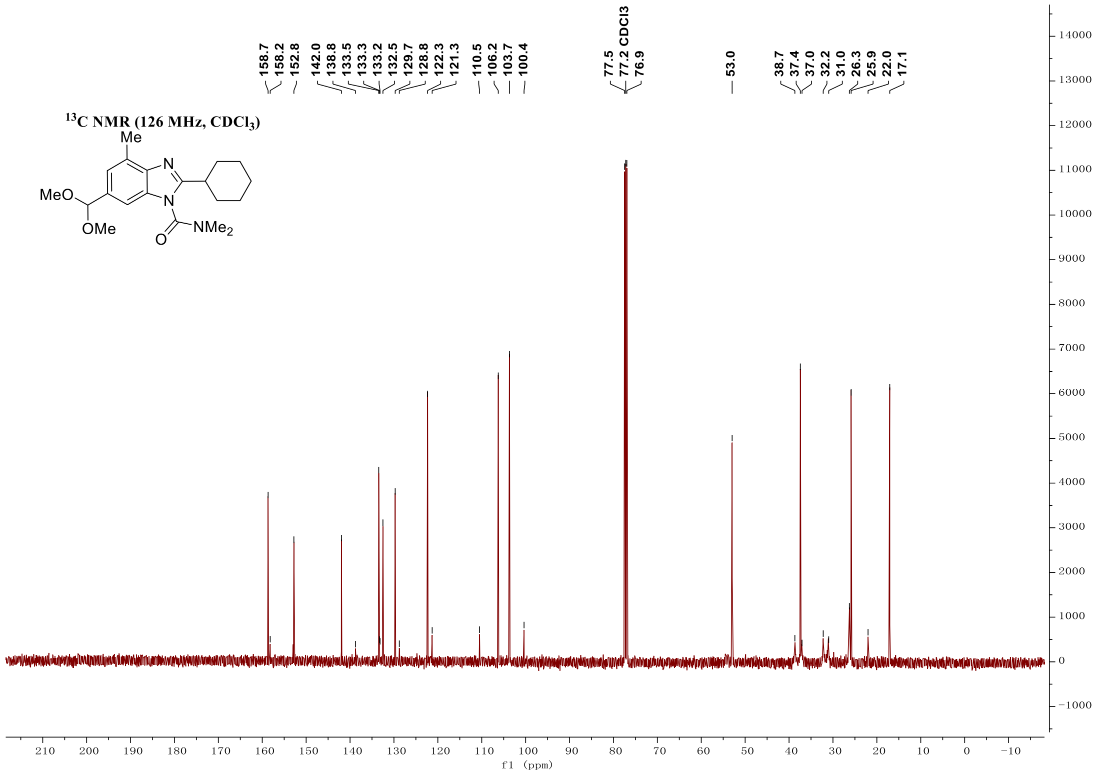

Supplementary Figure 105. ^{13}C NMR spectrum of compound 10.

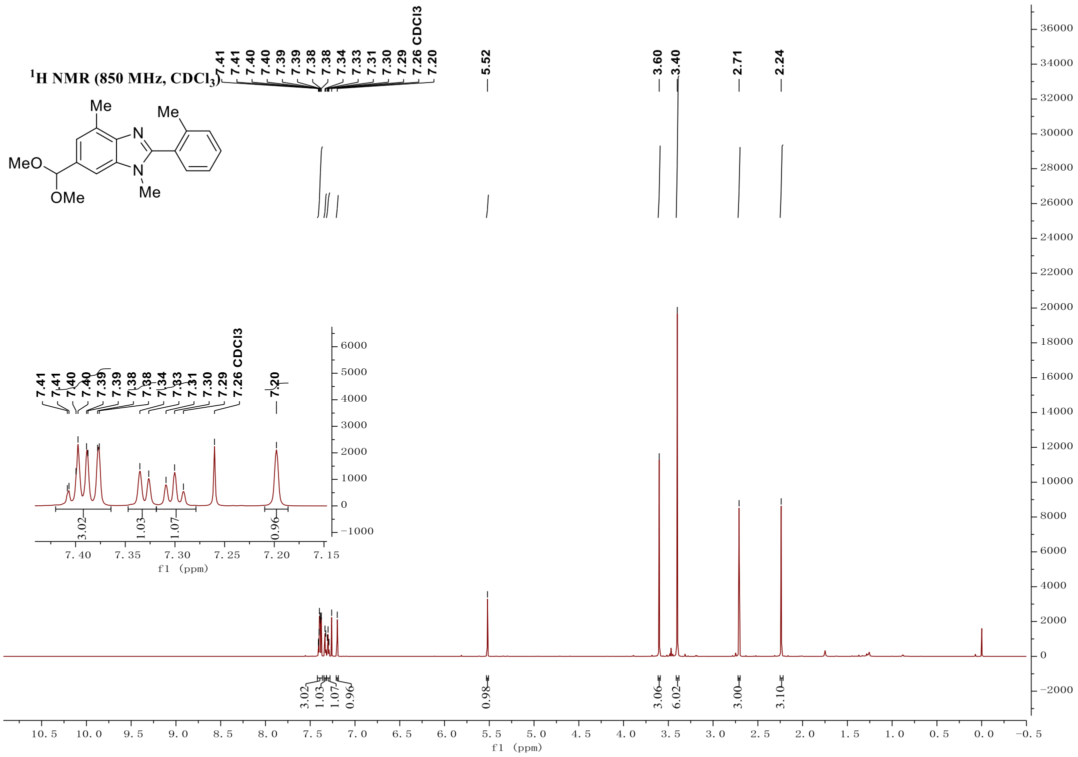

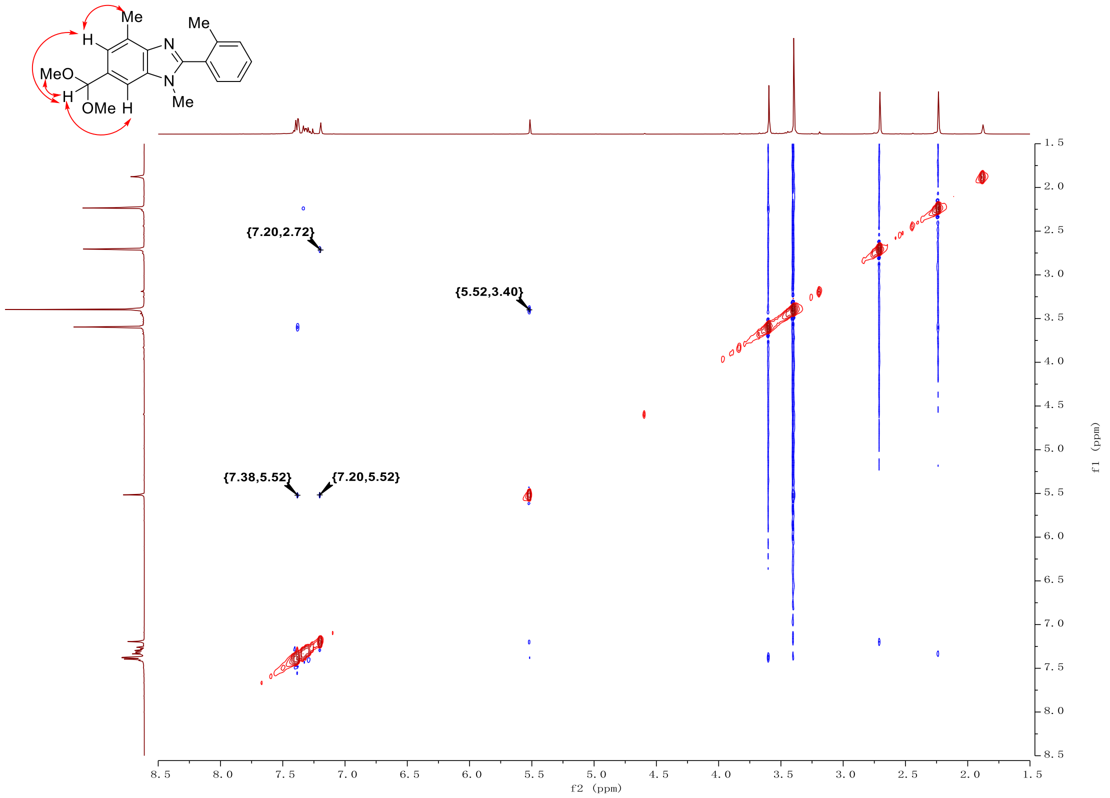

Supplementary Figure 106. ^{1}**H NMR and 2D NOESY spectra of compound 11.**

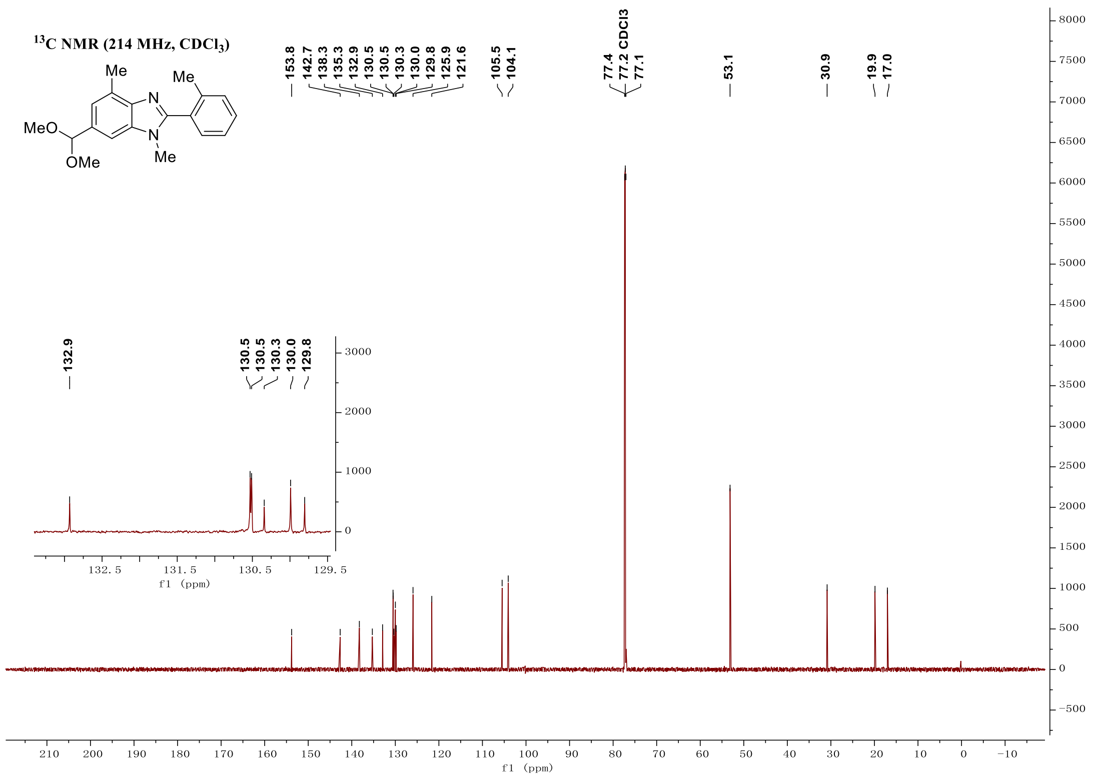

Supplementary Figure 107. ^{13}**C NMR spectrum of compound 11.**

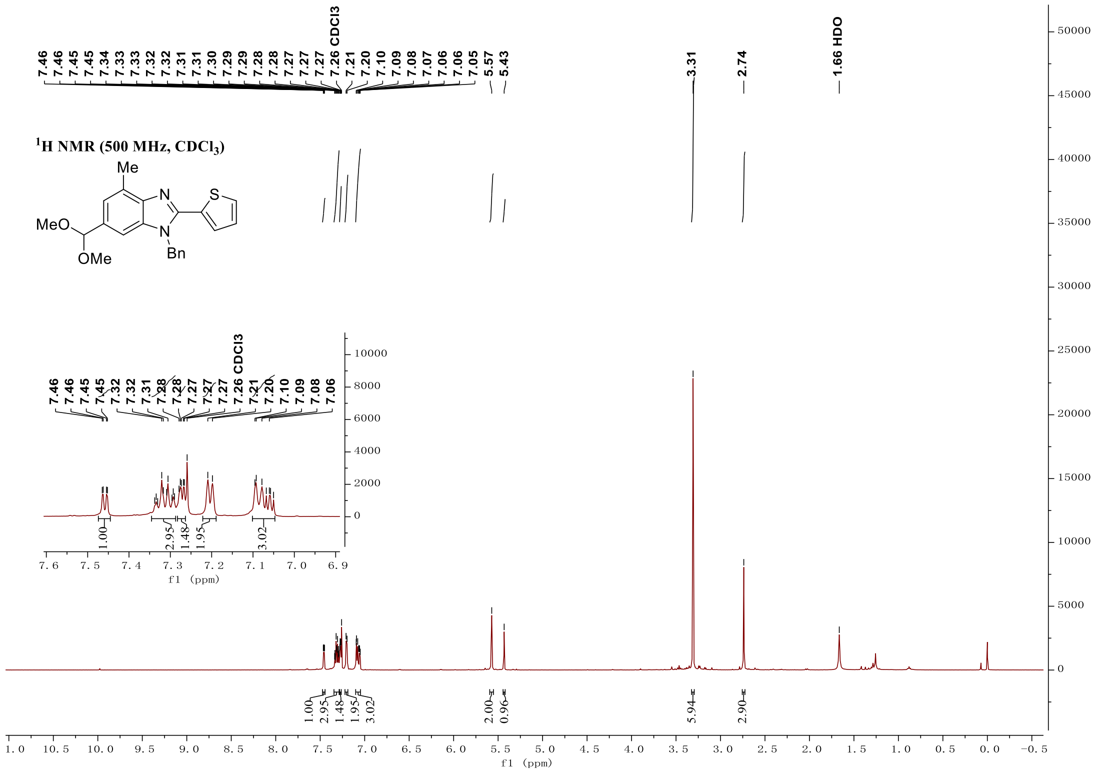

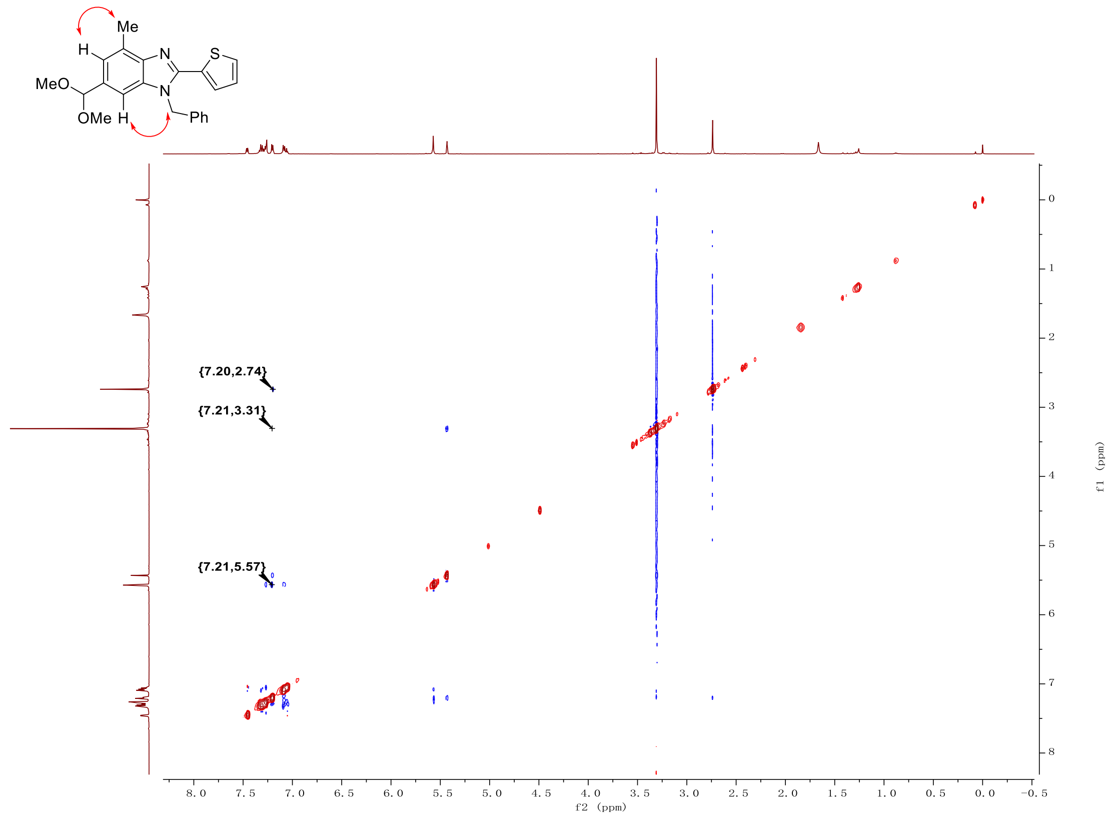

Supplementary Figure 108. ¹H NMR and 2D NOESY spectra of compound 12.

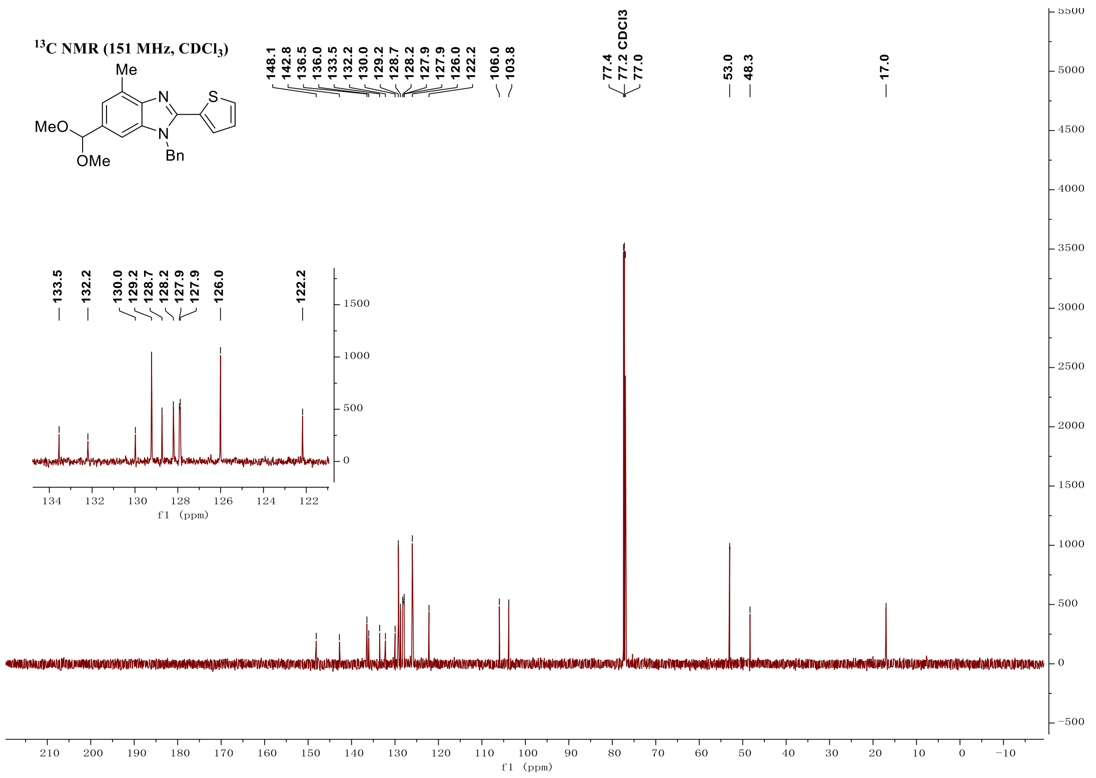

Supplementary Figure 109. ^{13}C NMR spectrum of compound 12.

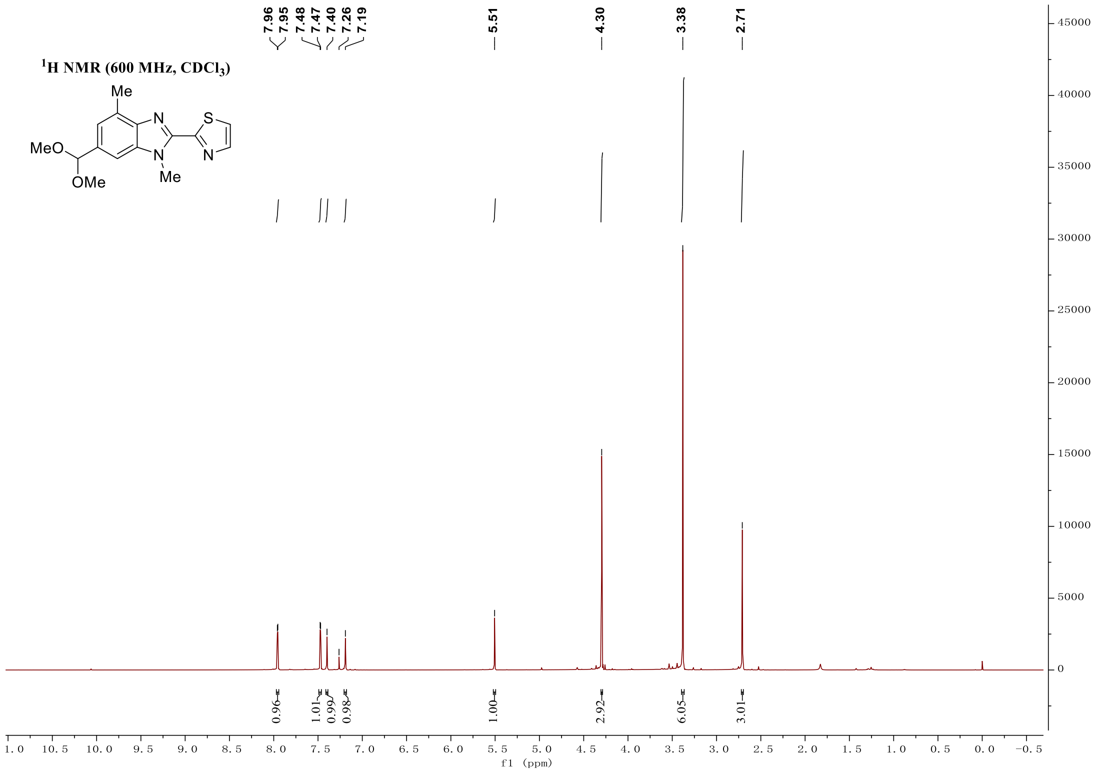

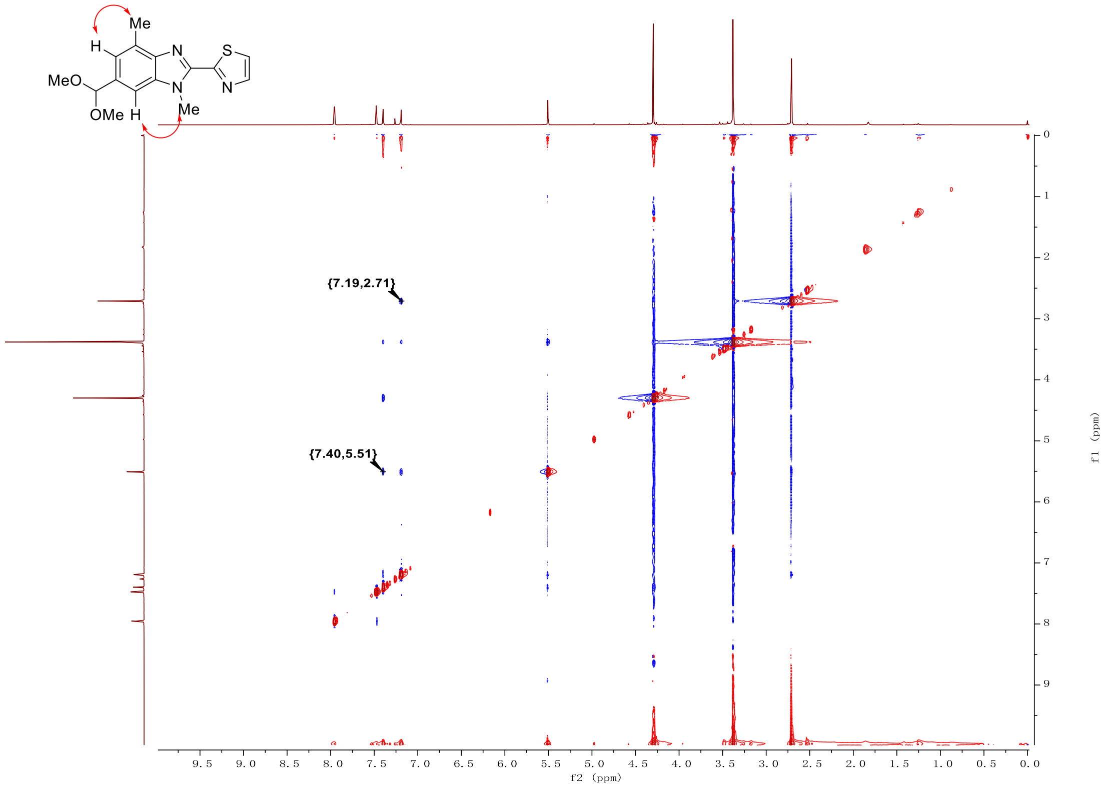

Supplementary Figure 110. ^{1}H NMR and 2D NOESY spectra of compound 13.

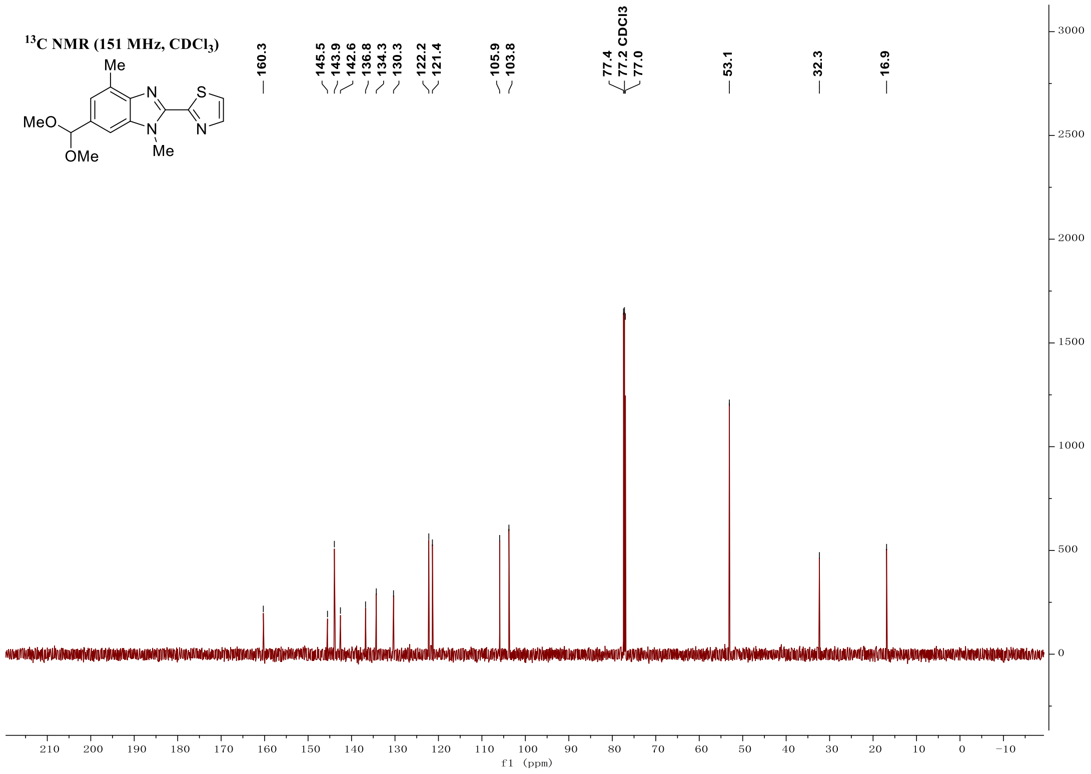

Supplementary Figure 111. ^{13}C NMR spectrum of compound 13.

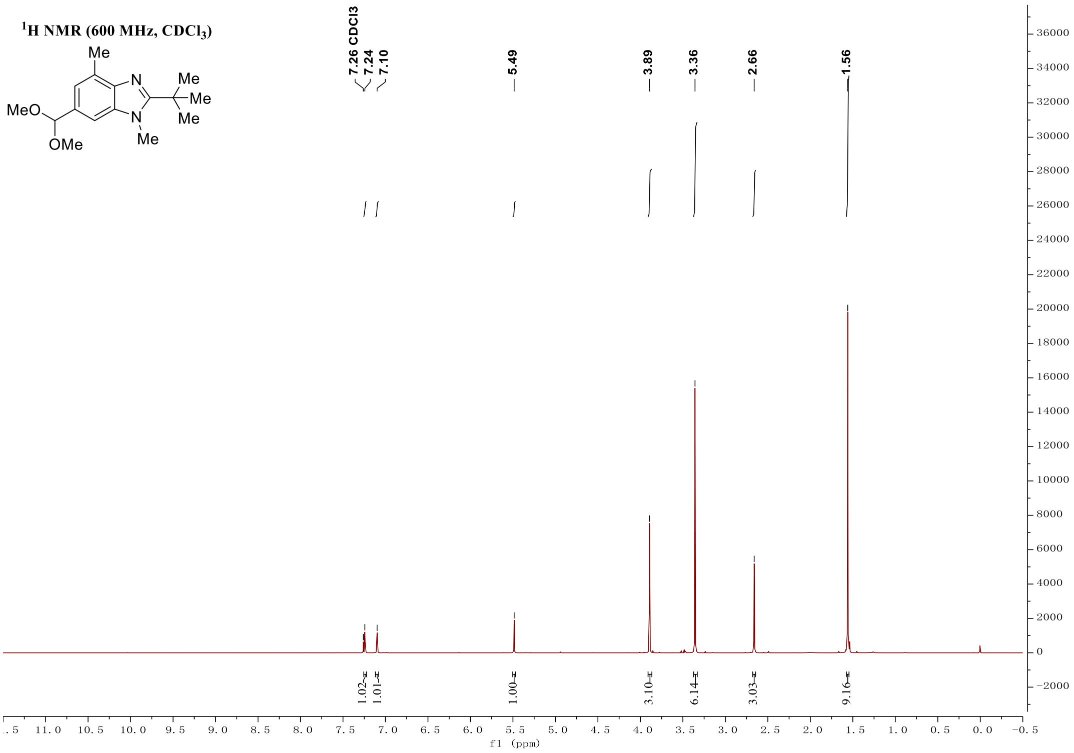

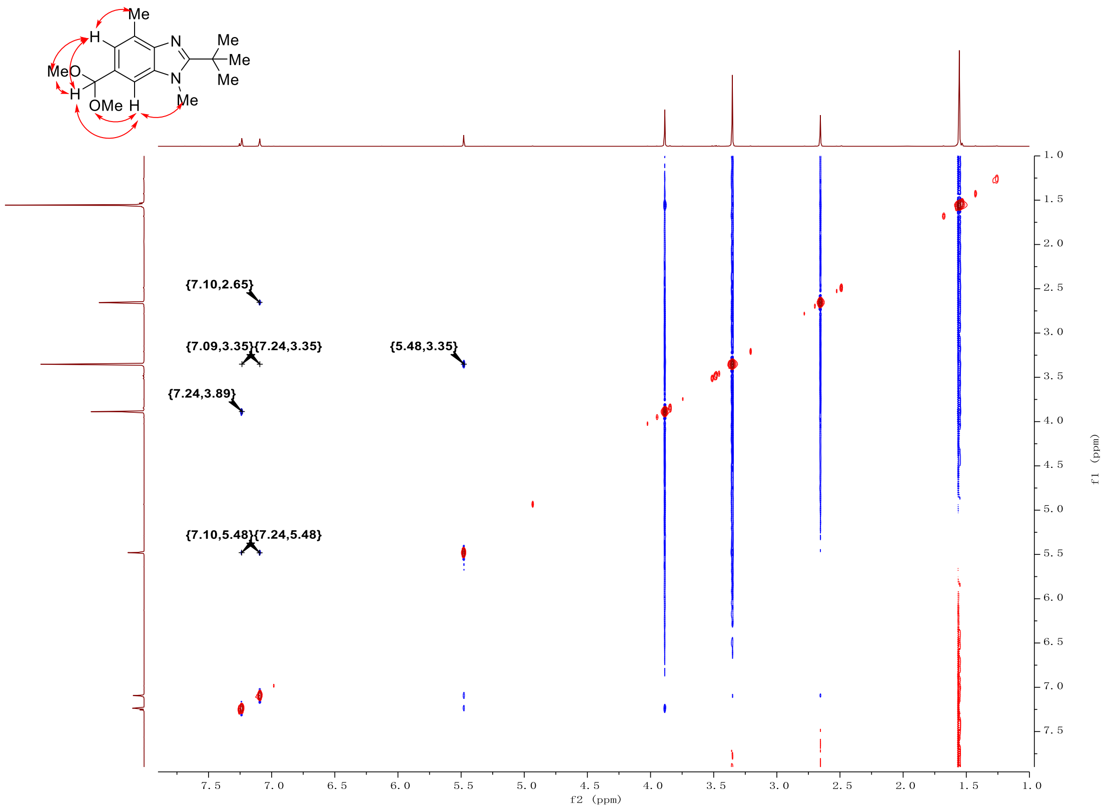

Supplementary Figure 112. ^{1}H NMR and 2D NOESY spectra of compound 14.

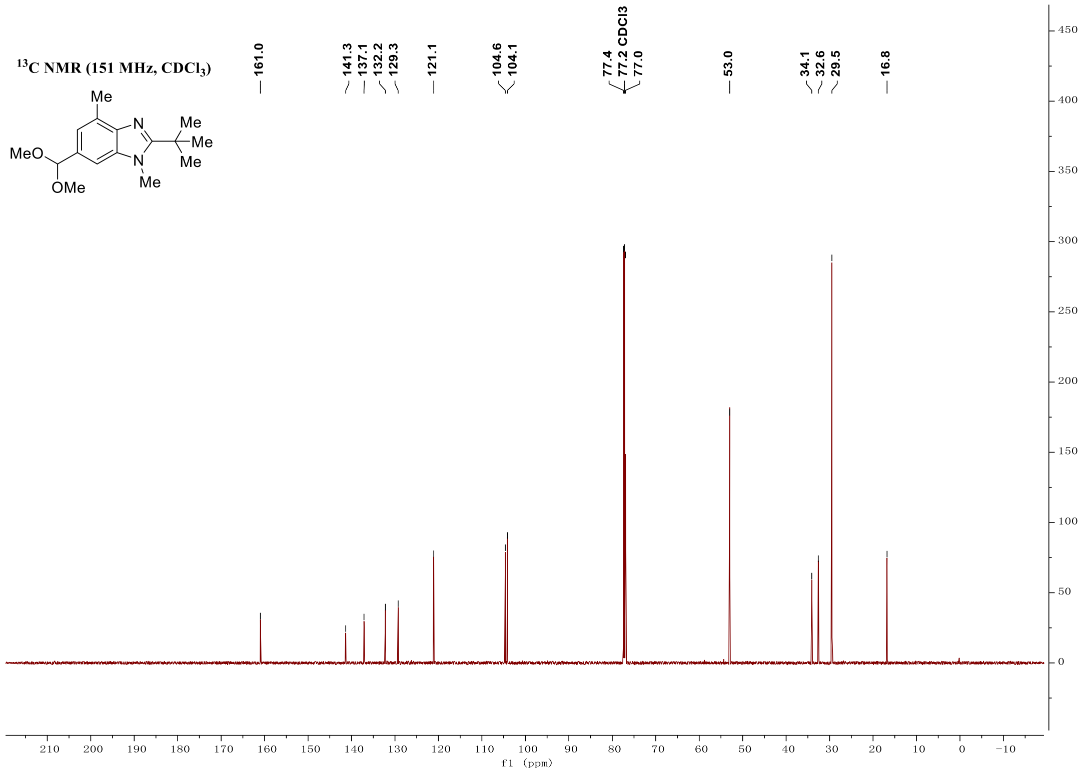

Supplementary Figure 113. ^{13}C NMR spectrum of compound 14.

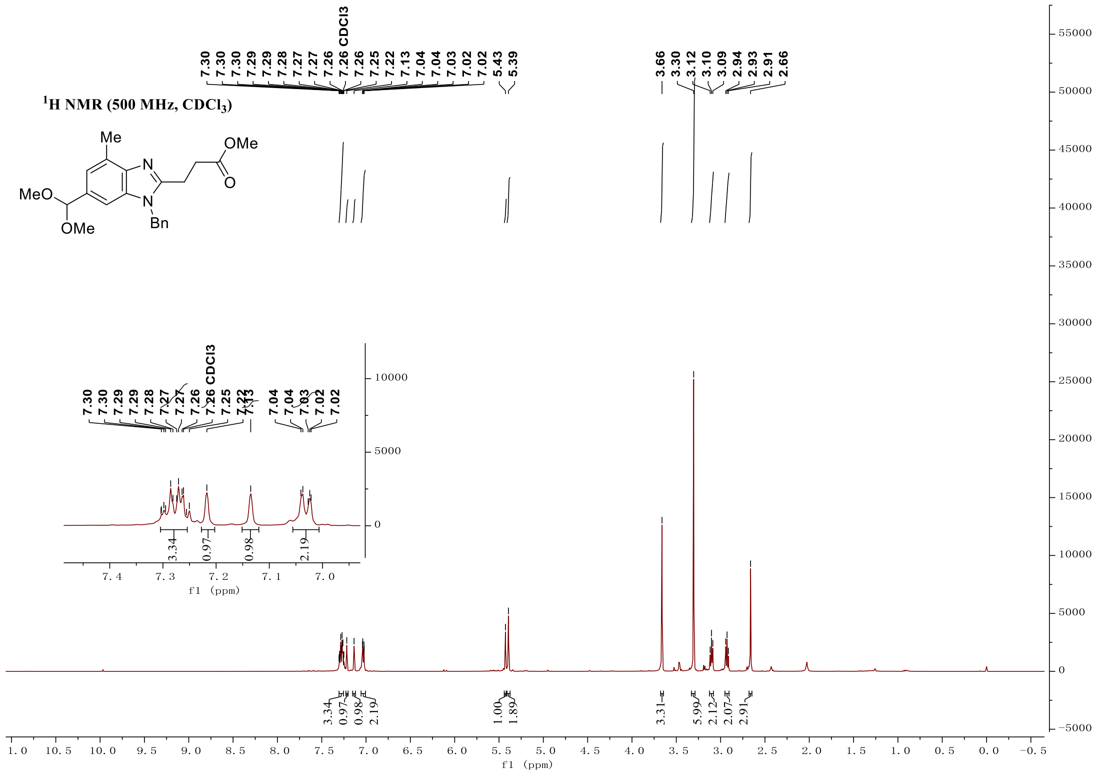

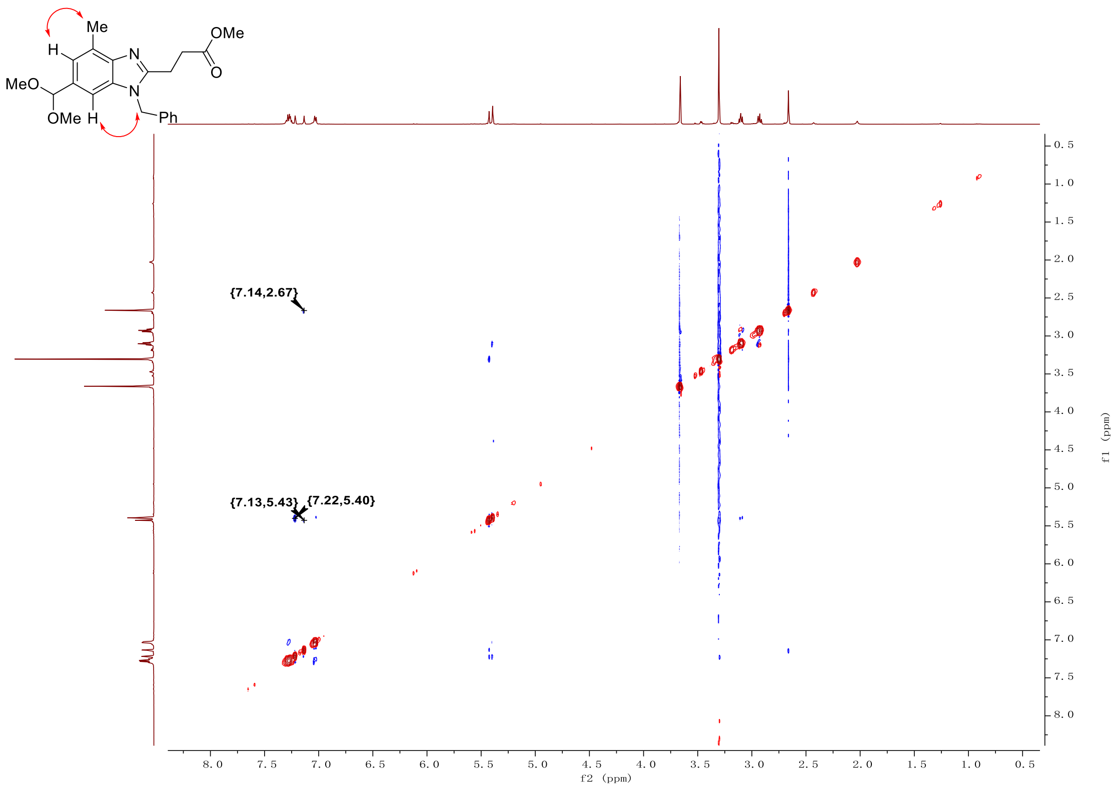

Supplementary Figure 114. H NMR and 2D NOESY spectra of compound 15.

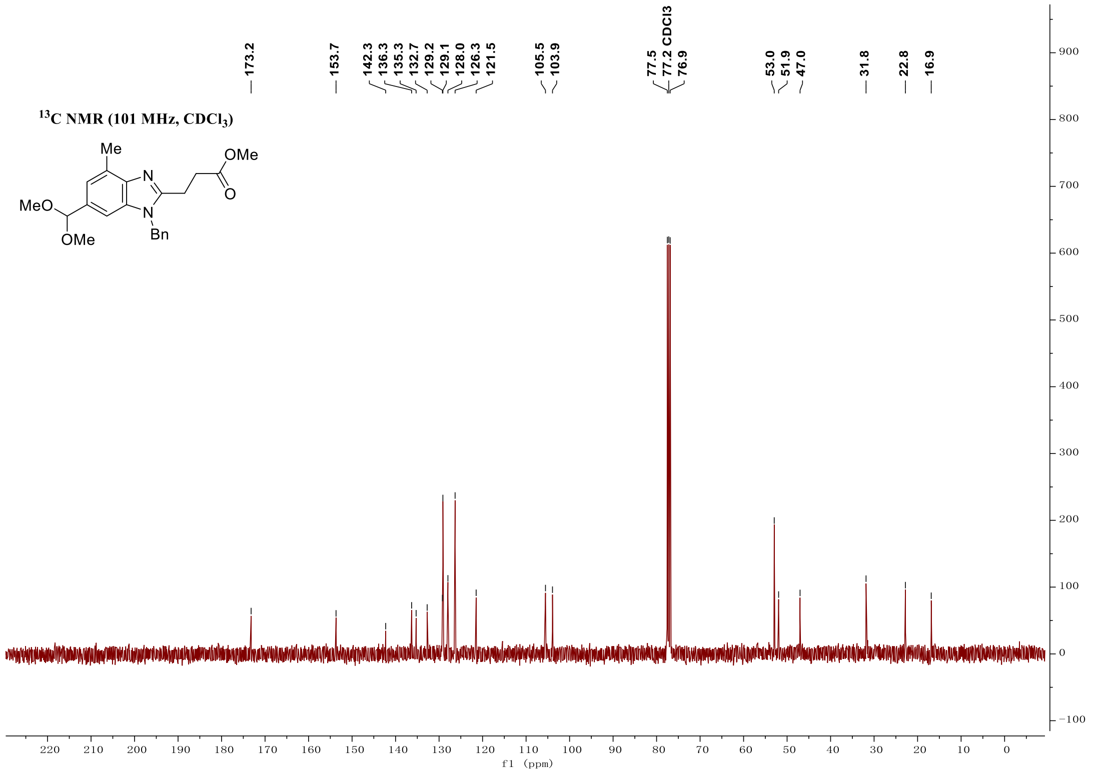

Supplementary Figure 115. ^{13}C NMR spectrum of compound 15.

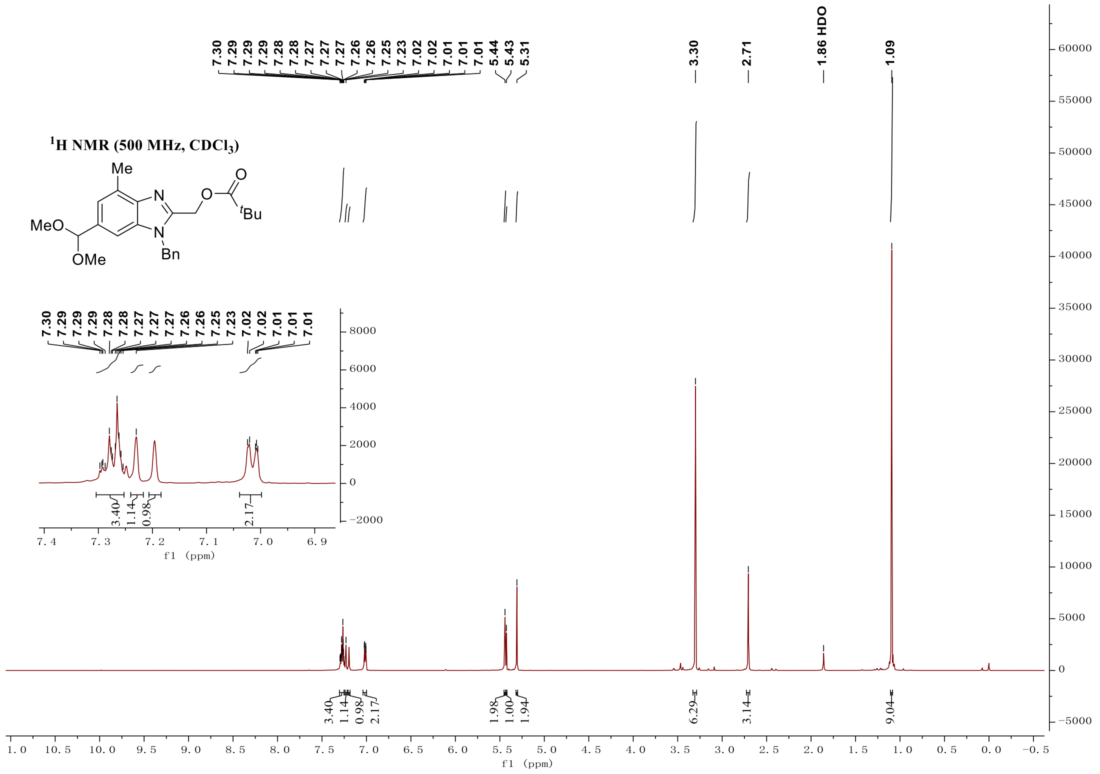

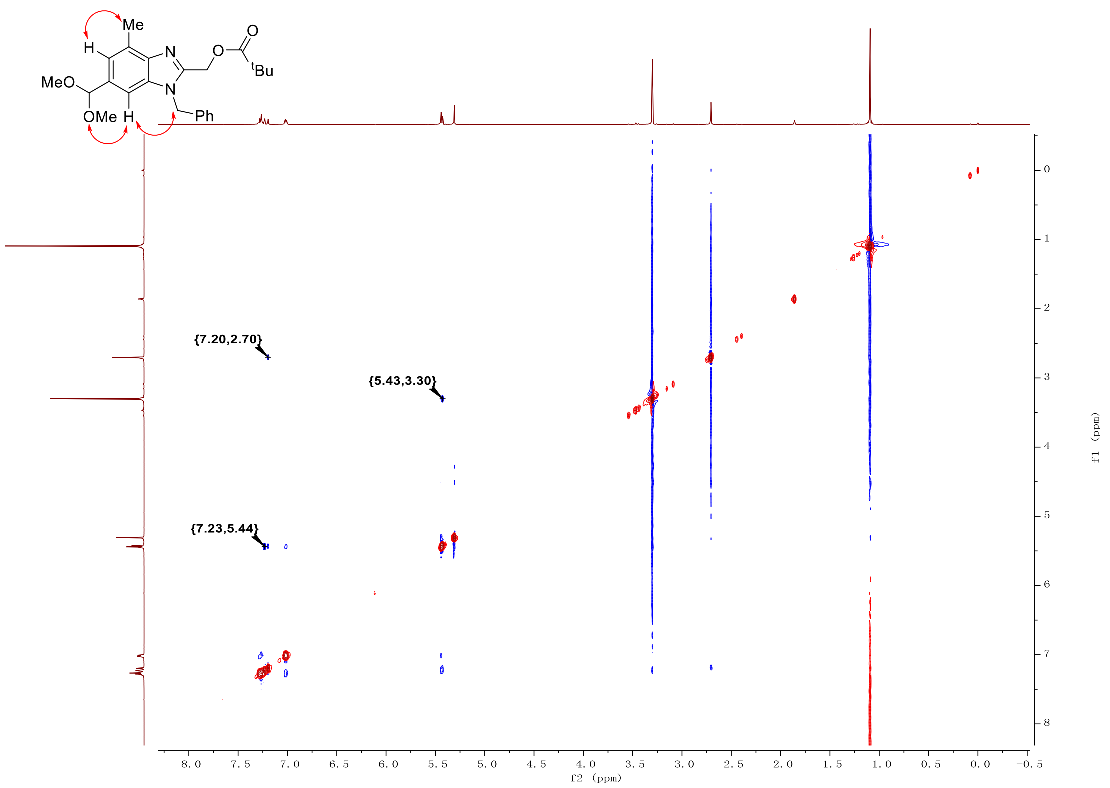

Supplementary Figure 116. ^{1}**H NMR and 2D NOESY spectra of compound 16.**

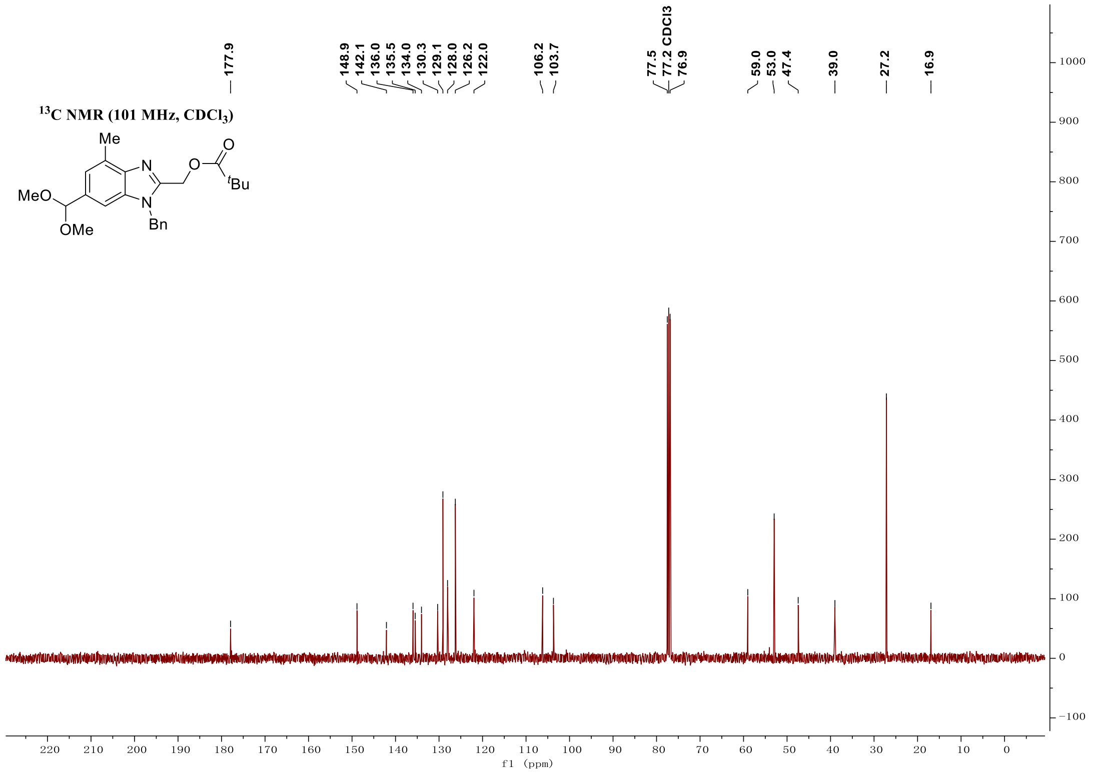

Supplementary Figure 117. ^{13}C NMR spectrum of compound 16.

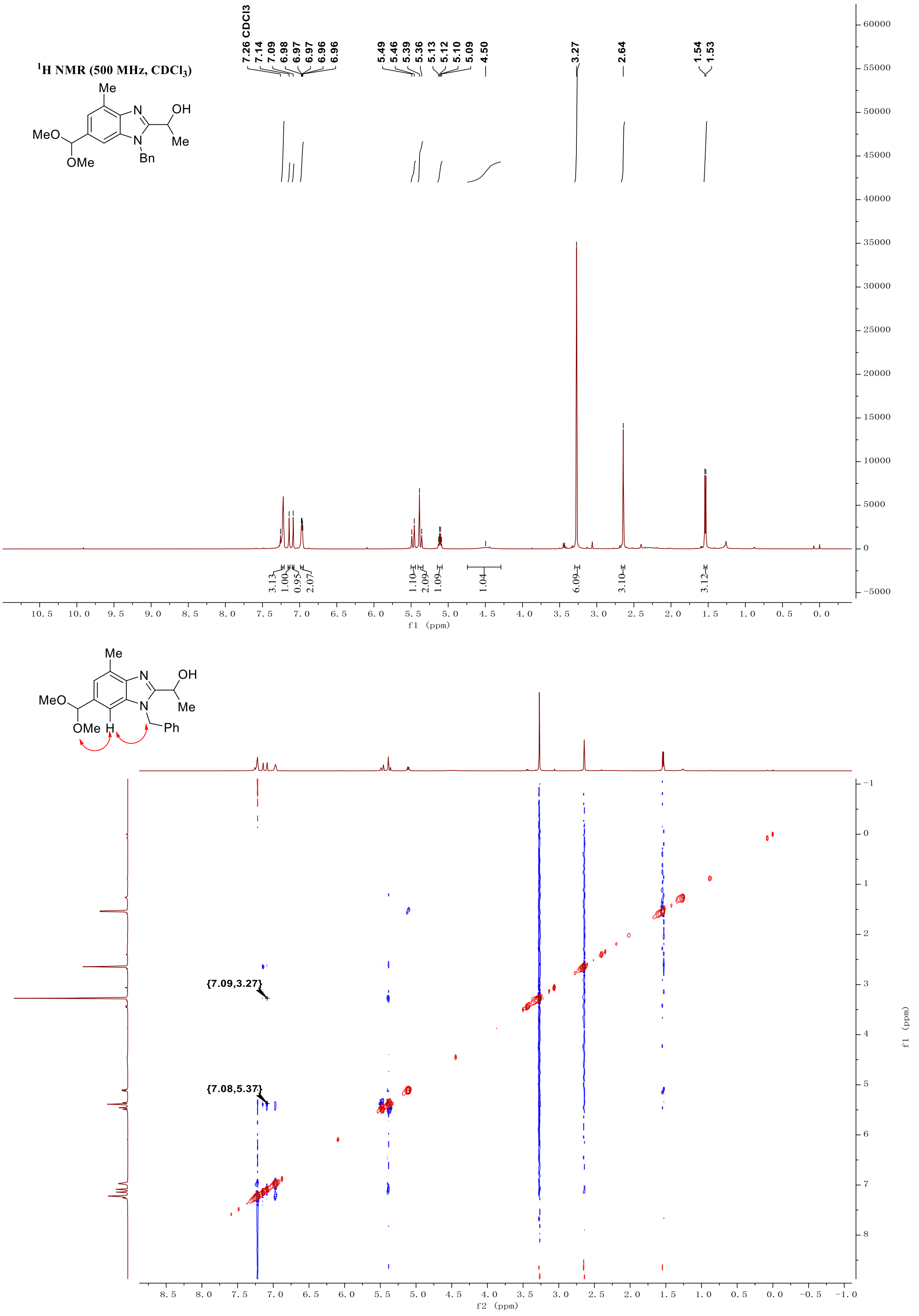

Supplementary Figure 118. ¹H NMR and 2D NOESY spectra of compound 17.

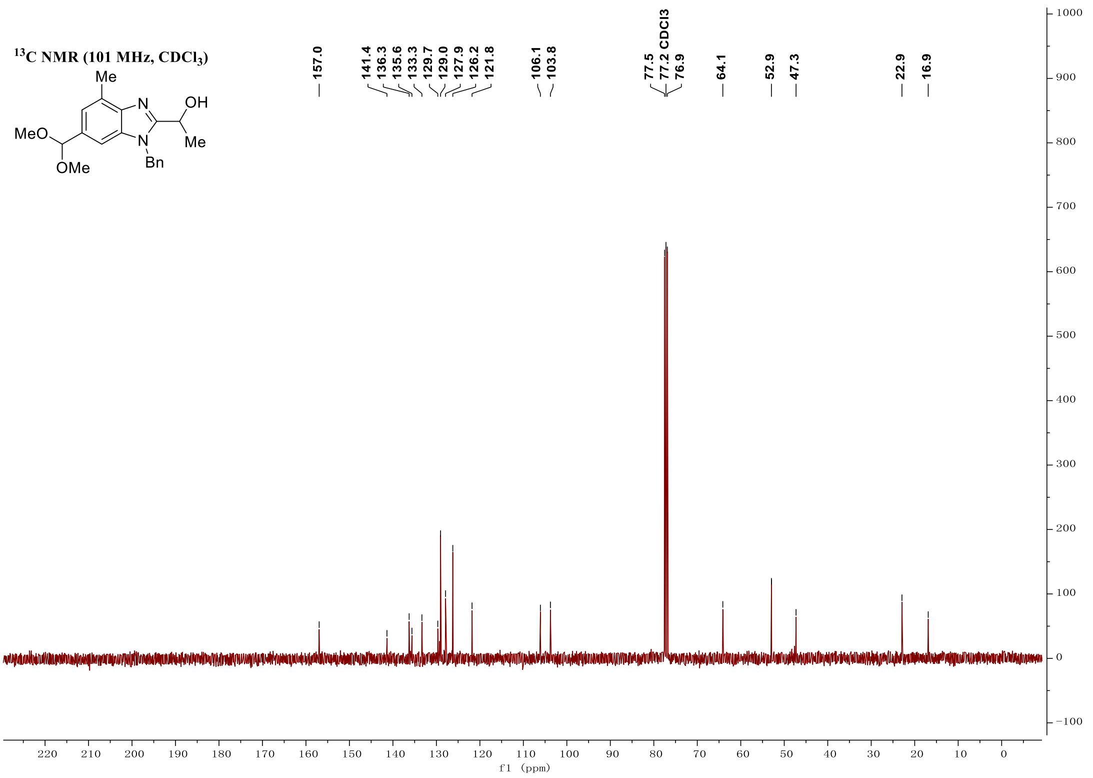

Supplementary Figure 119. ^{13}C NMR spectrum of compound 17.

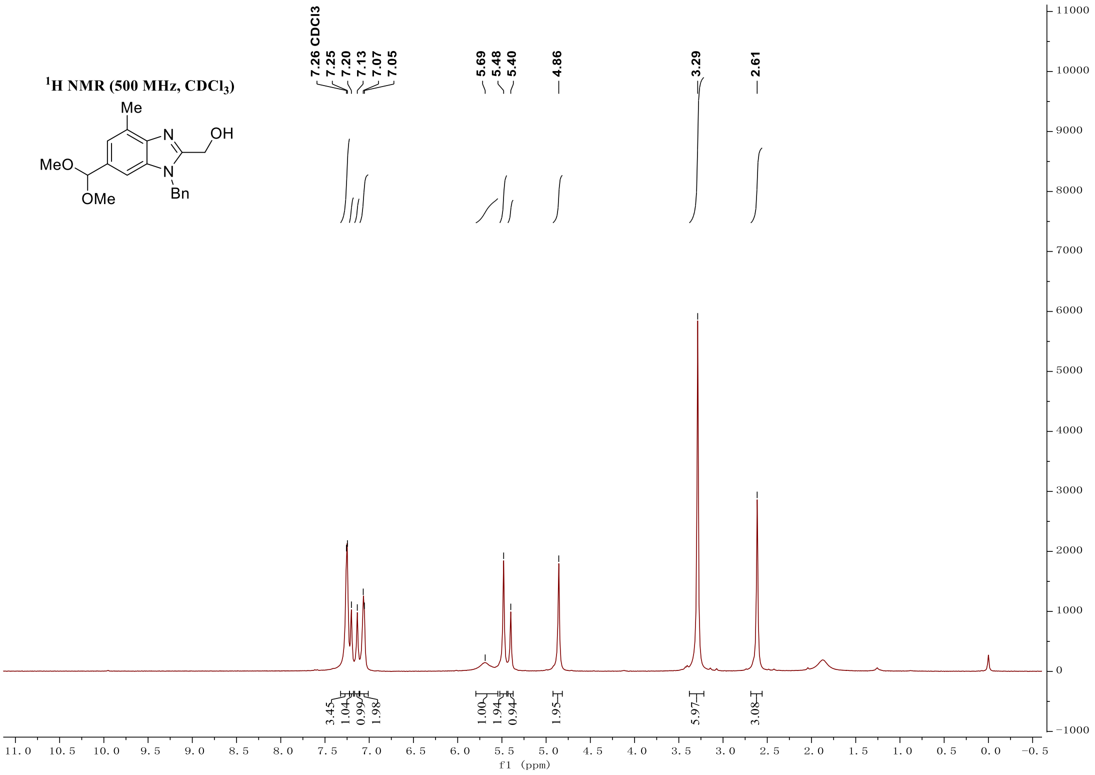

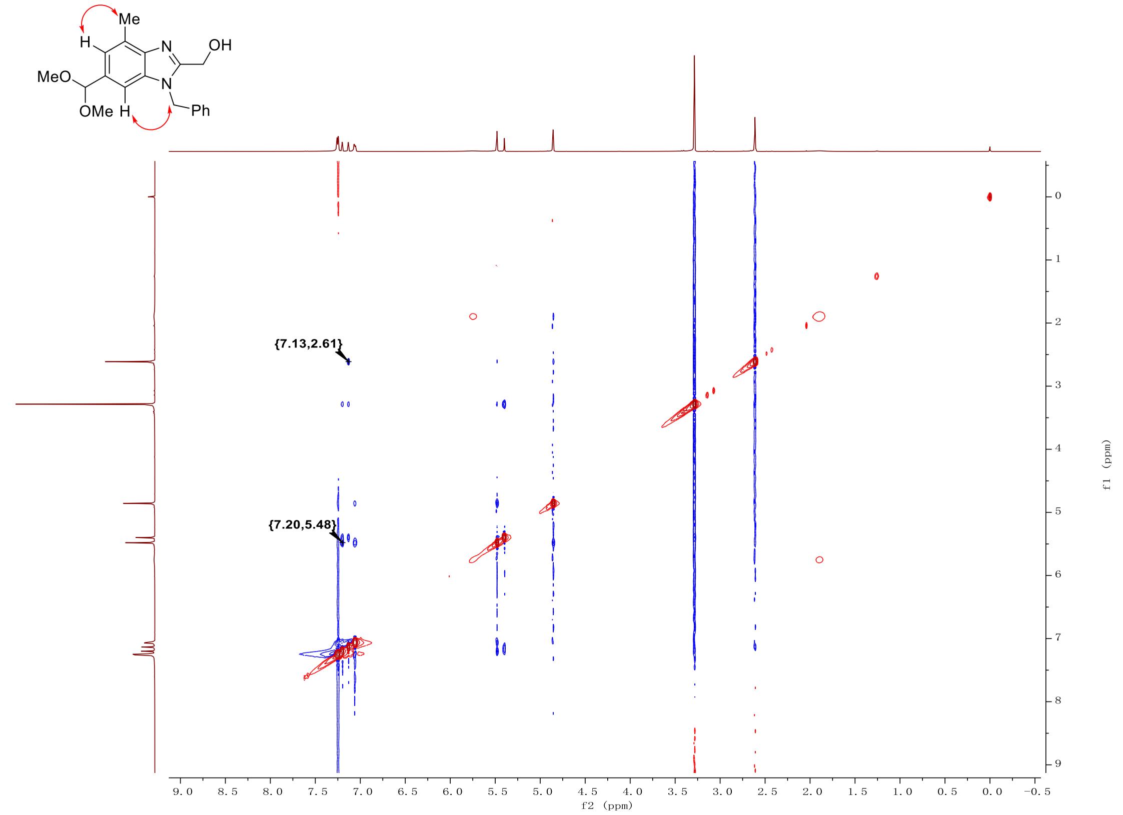

Supplementary Figure 120. ^{1}H NMR and 2D NOESY spectra of compound 18.

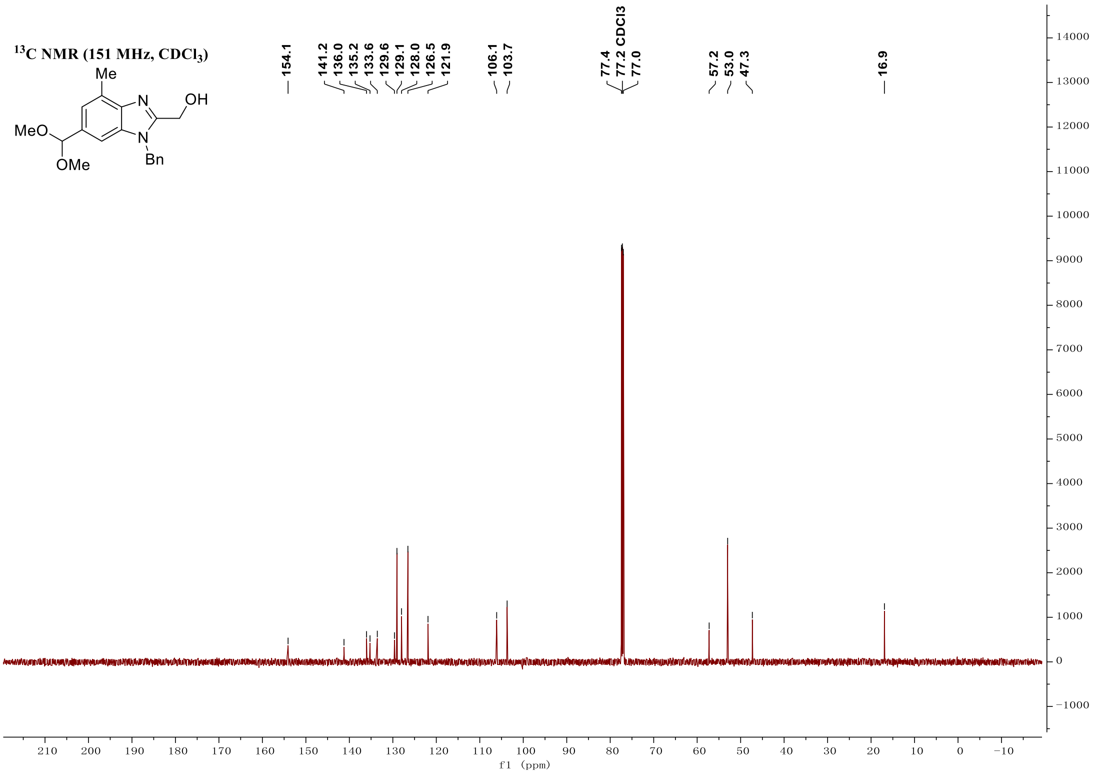

Supplementary Figure 121. ^{13}C NMR spectrum of compound 18.

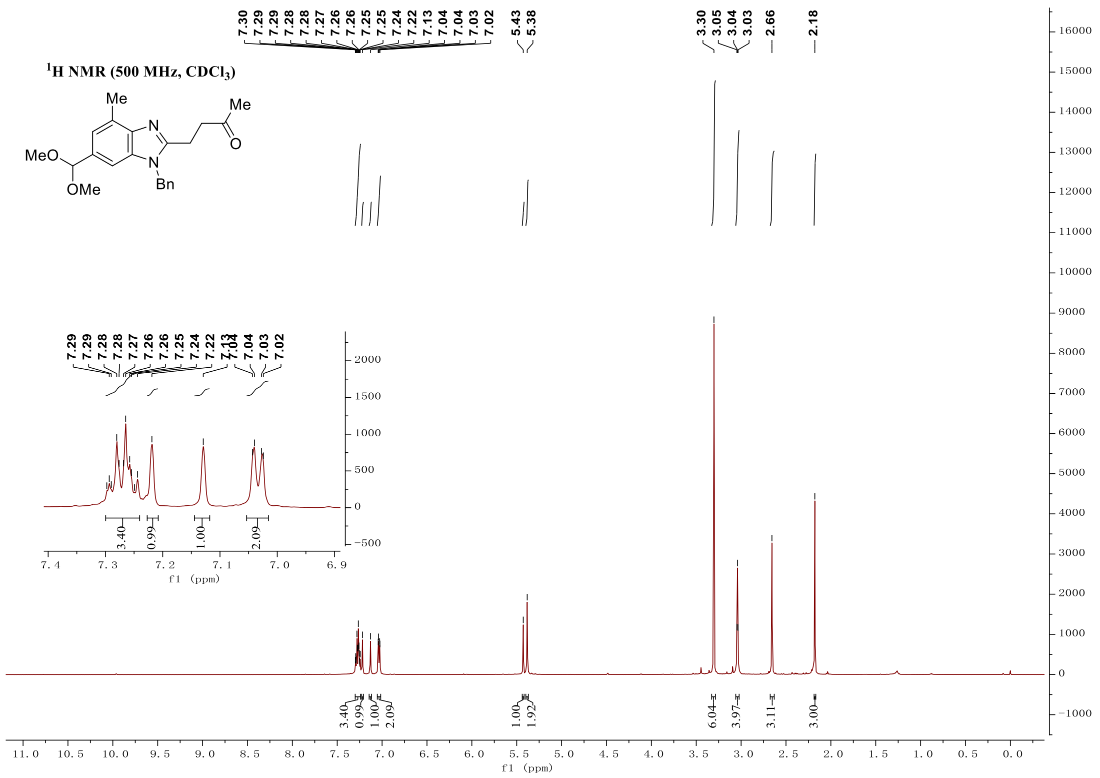

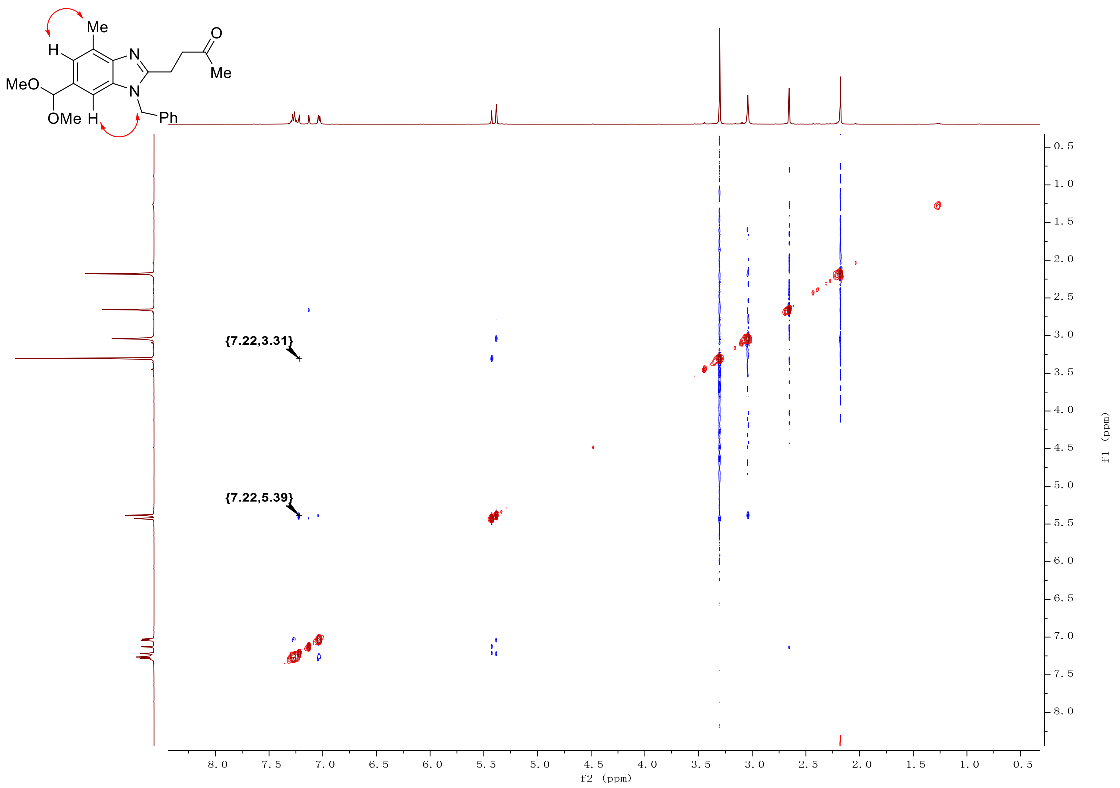

Supplementary Figure 122. ^{1}H NMR and 2D NOESY spectra of compound 19.

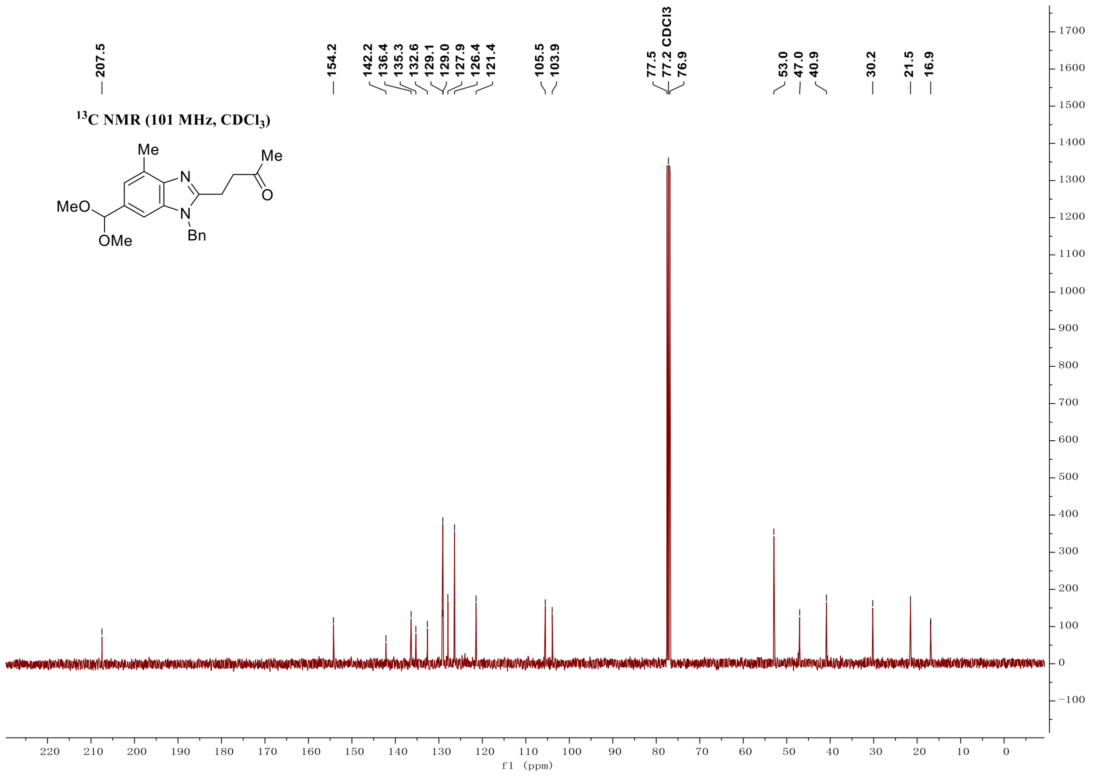

Supplementary Figure 123. ^{13}C NMR spectrum of compound 19.

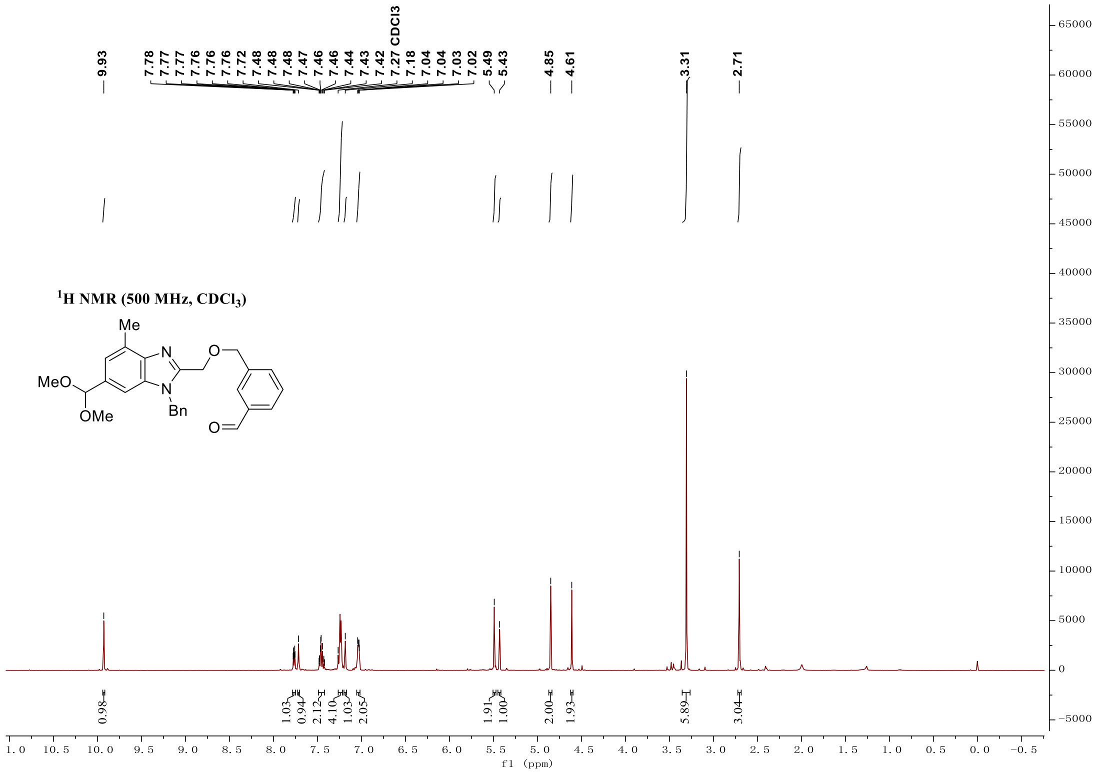

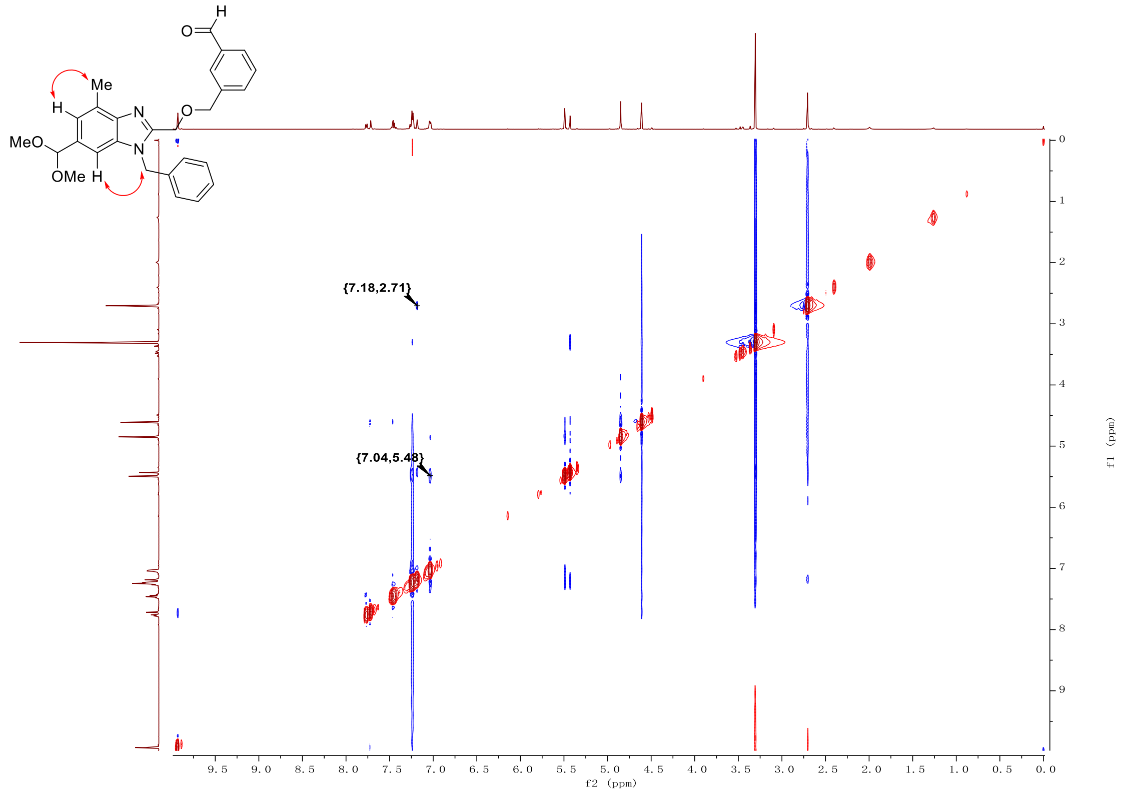

Supplementary Figure 124. ^{1}H NMR and 2D NOESY spectra of compound 20.

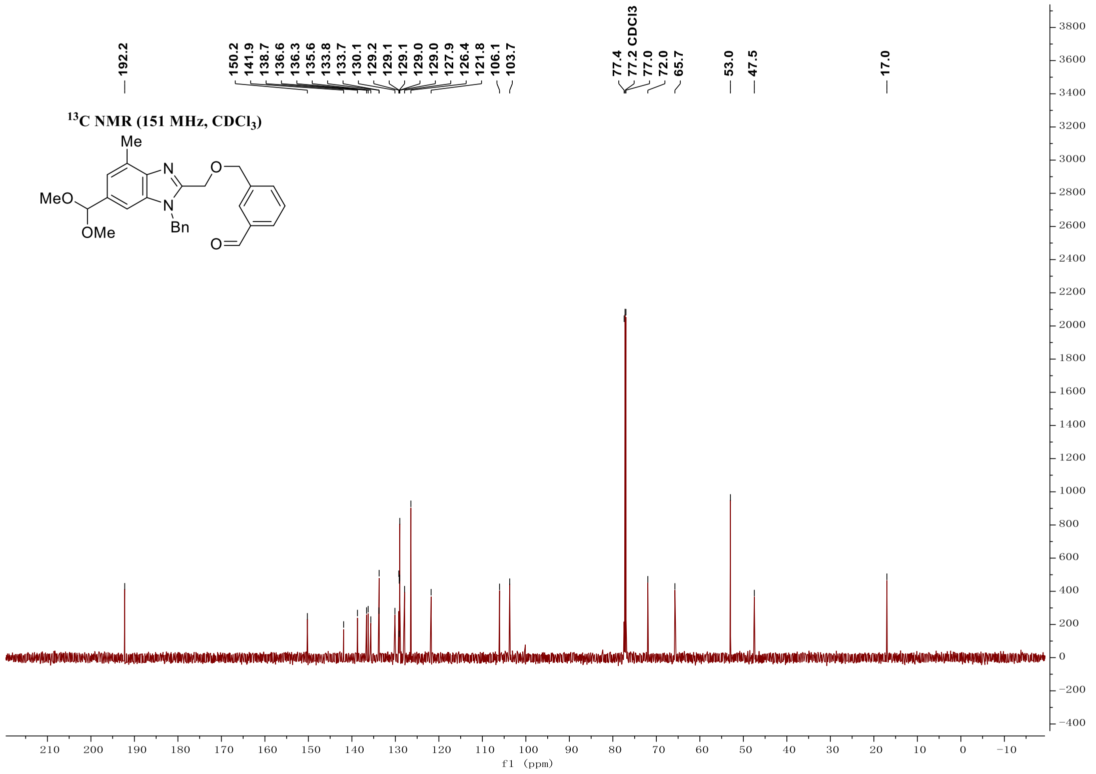

Supplementary Figure 125. ^{13}C NMR spectrum of compound 20.

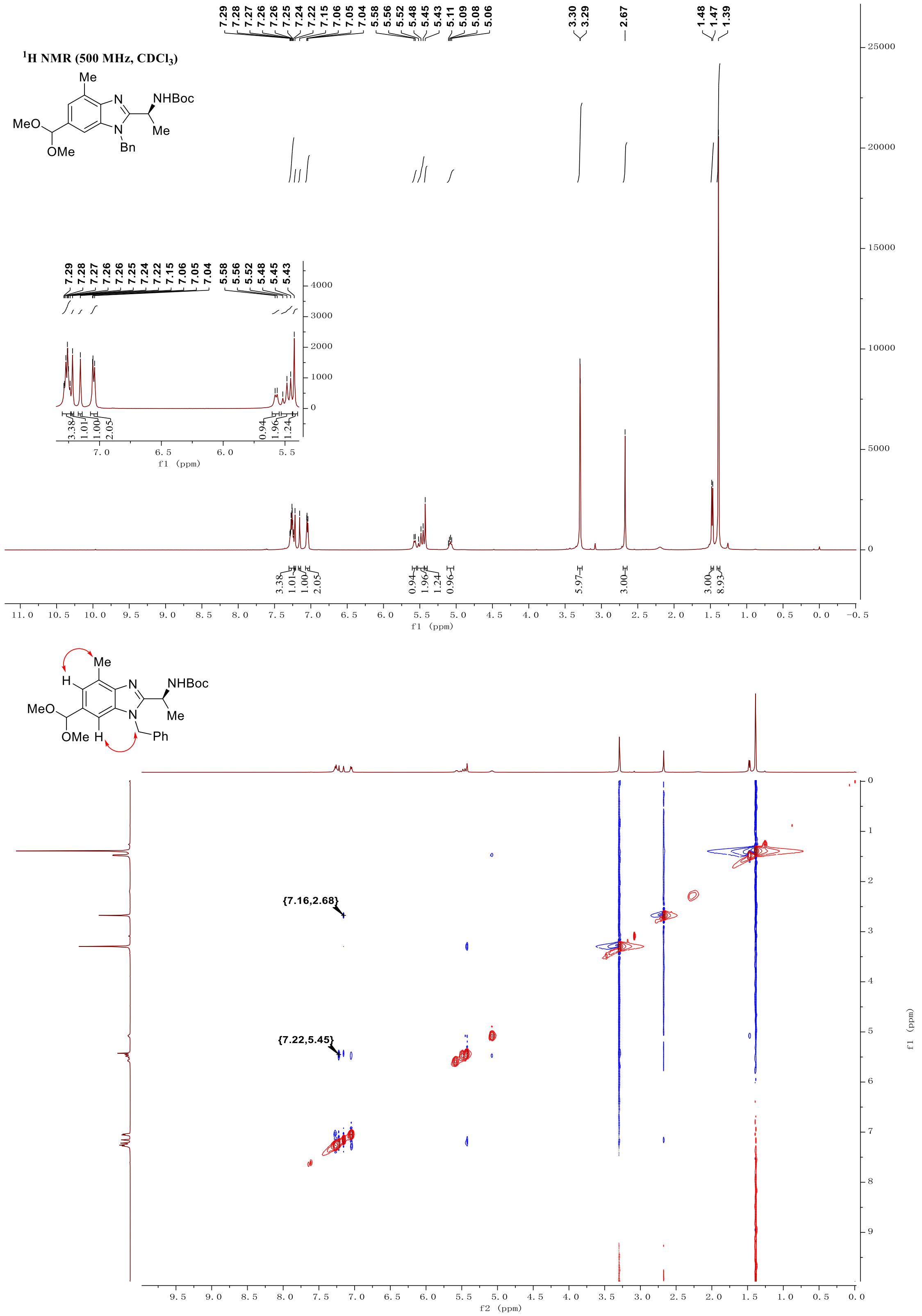

Supplementary Figure 126. ^{1}H NMR and 2D NOESY spectra of compound 21.

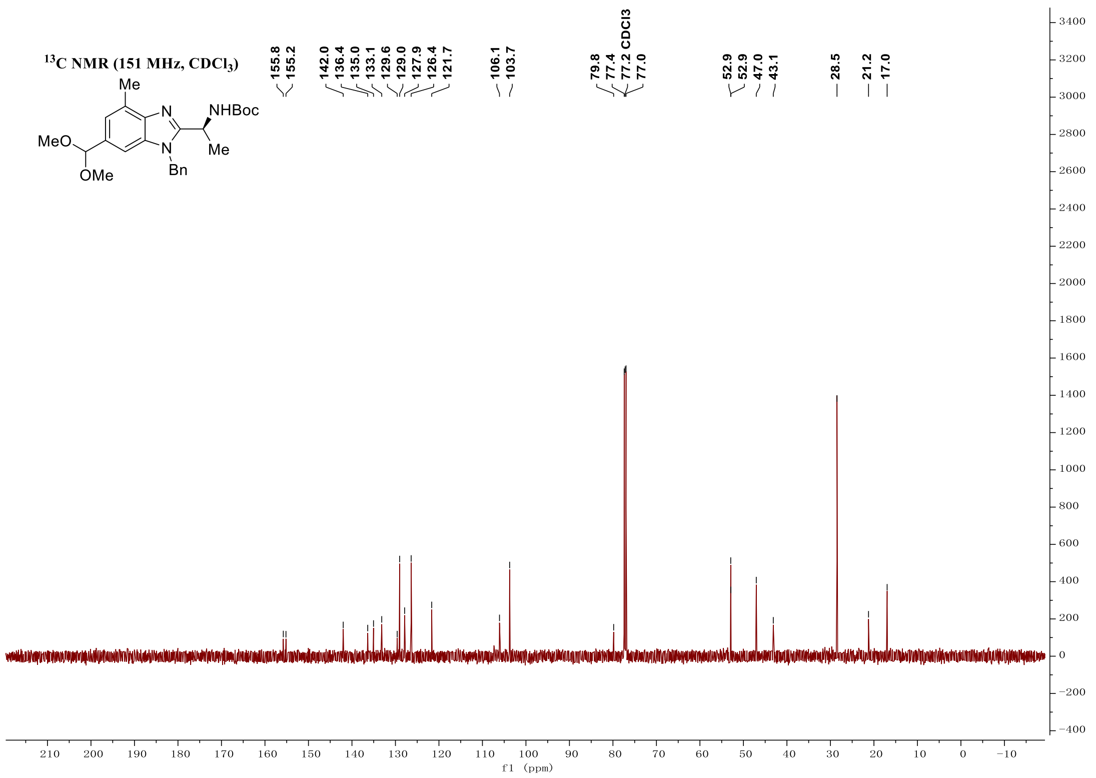

Supplementary Figure 127. ^{13}C NMR spectrum of compound 21.

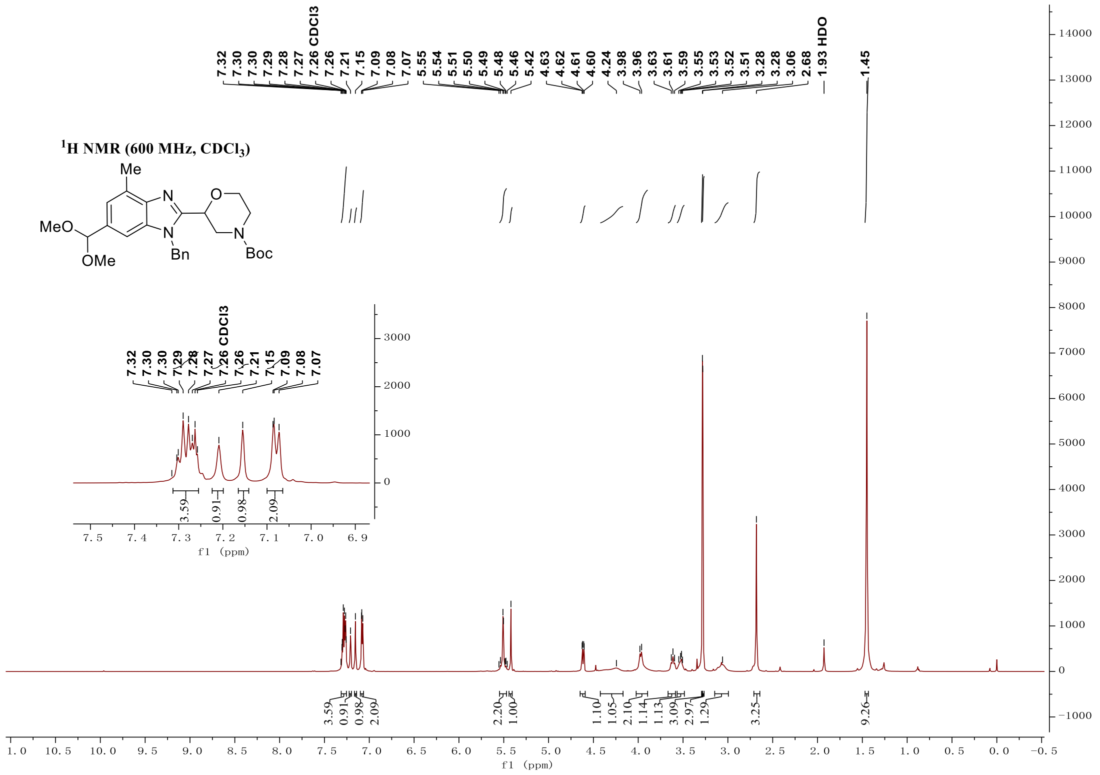

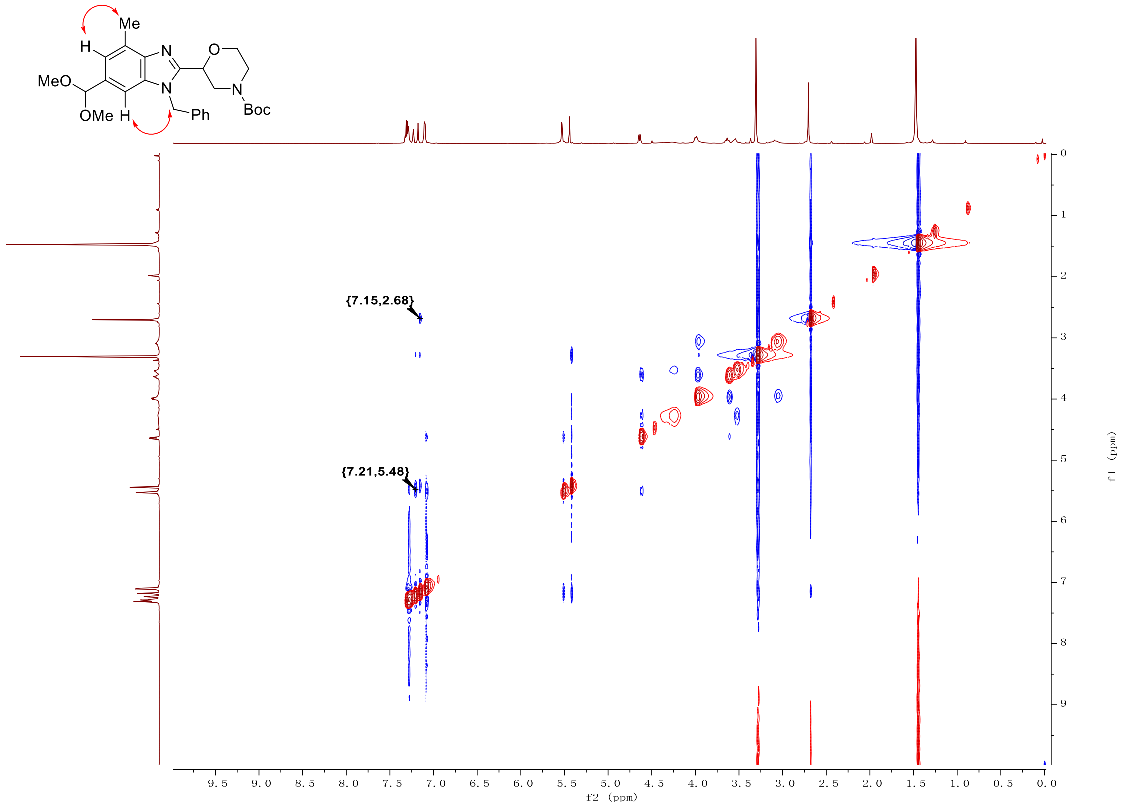

Supplementary Figure 128. ^{1}H NMR and 2D NOESY spectra of compound 22.

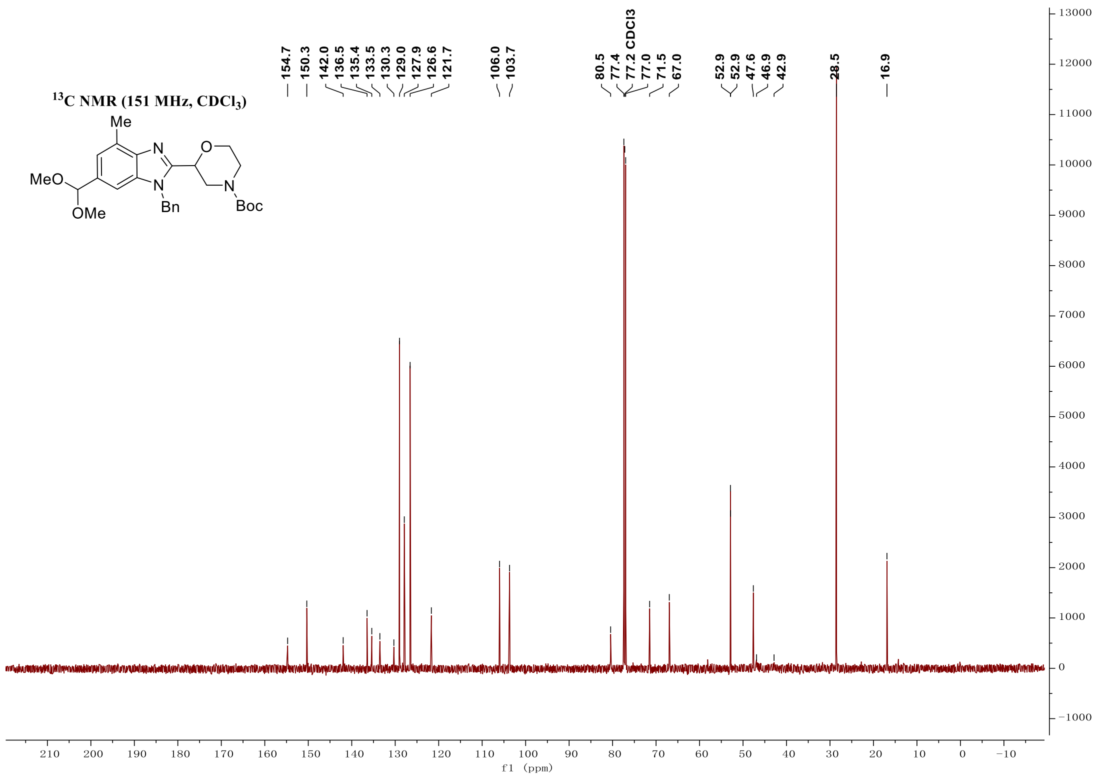

Supplementary Figure 129. ^{13}C NMR spectrum of compound 22.

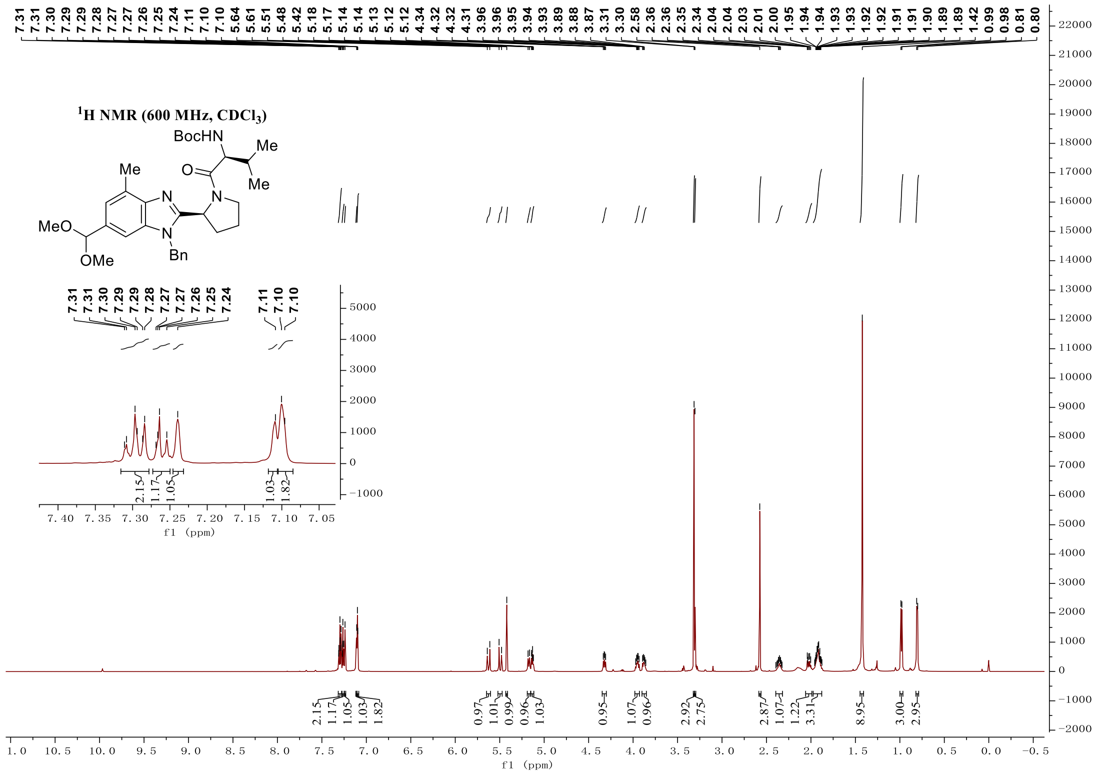

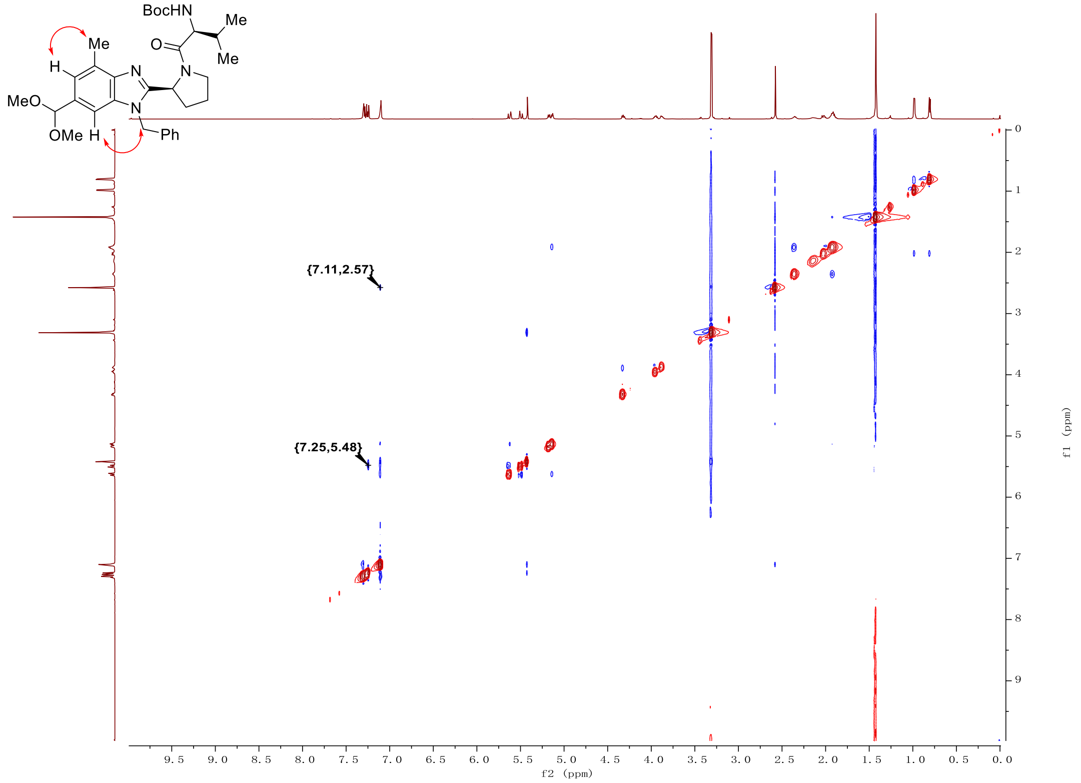

Supplementary Figure 130. ^{1}H NMR and 2D NOESY spectra of compound 23.

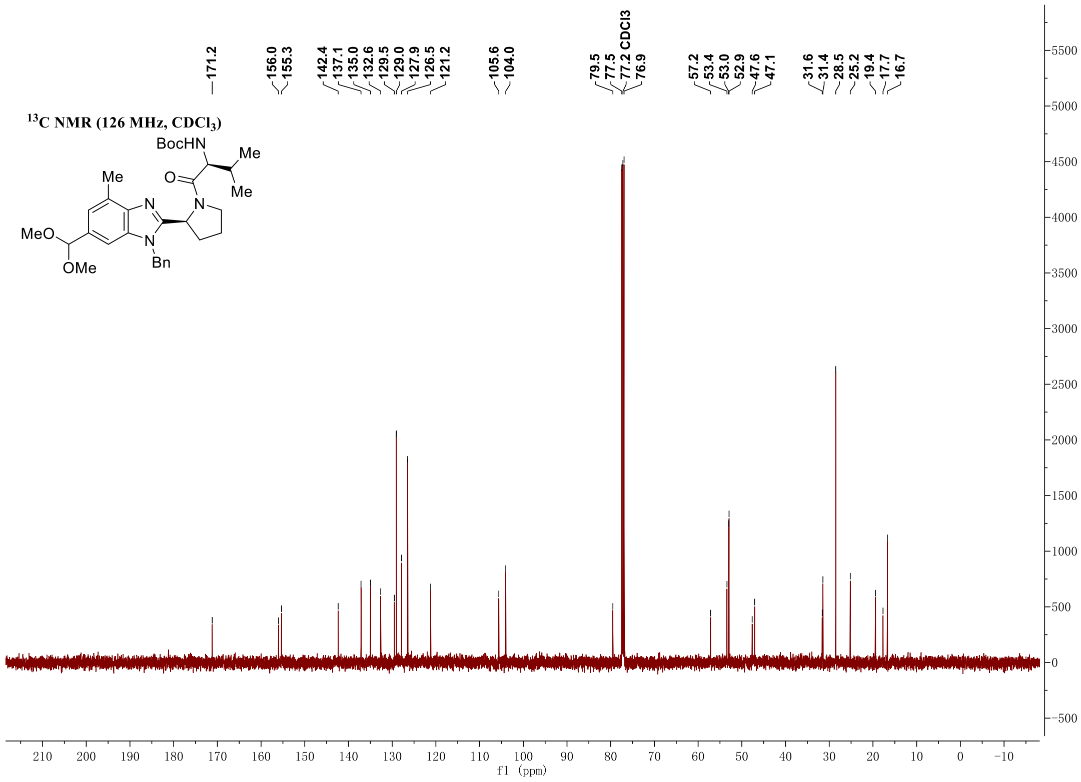

Supplementary Figure 131. ^{13}C NMR spectrum of compound 23.

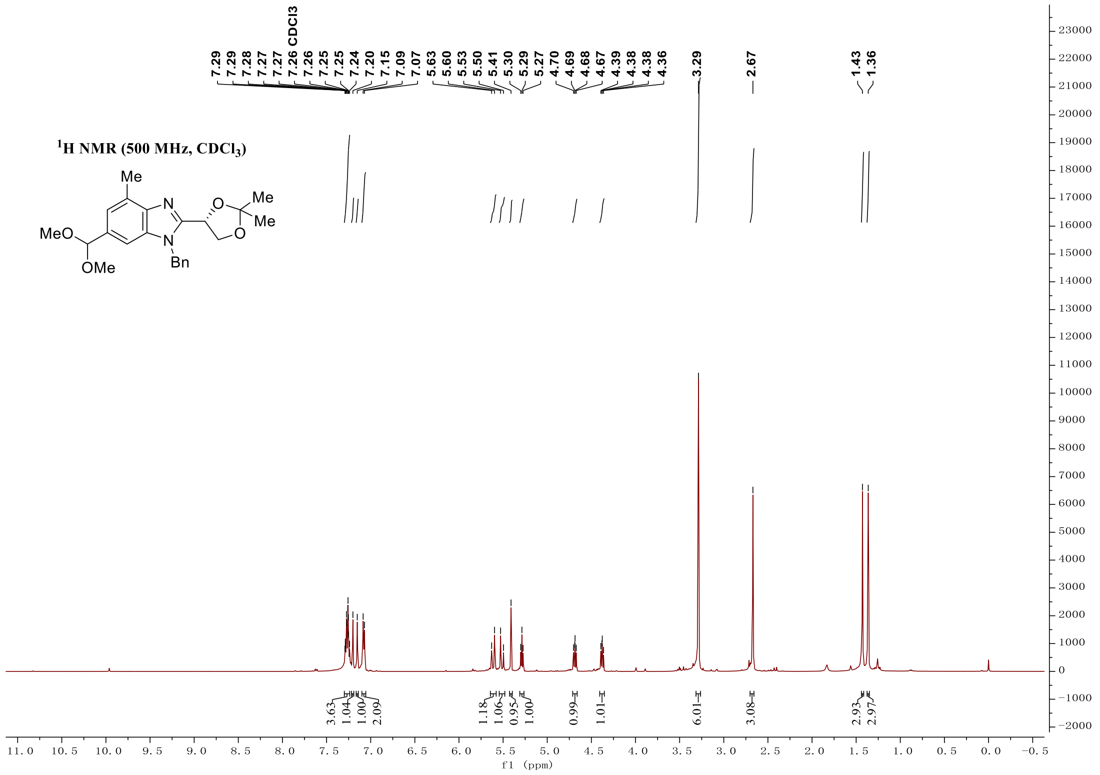

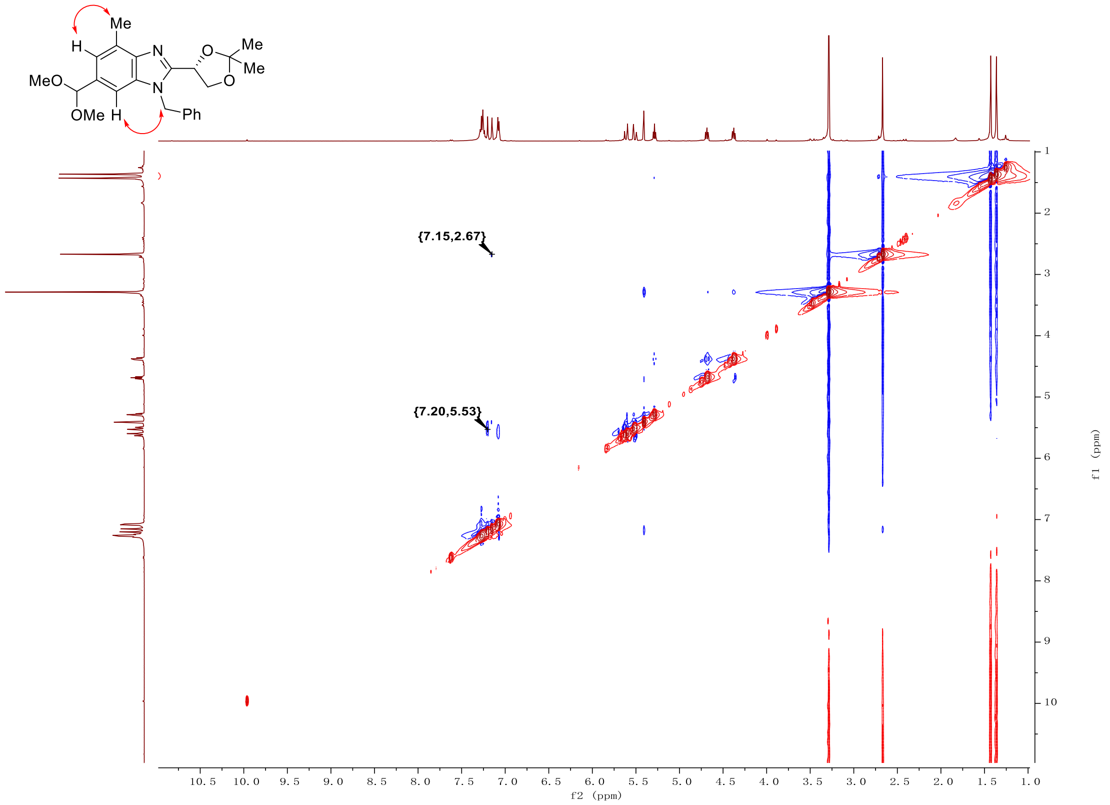

Supplementary Figure 132. ^{1}H NMR and 2D NOESY spectra of compound 24.

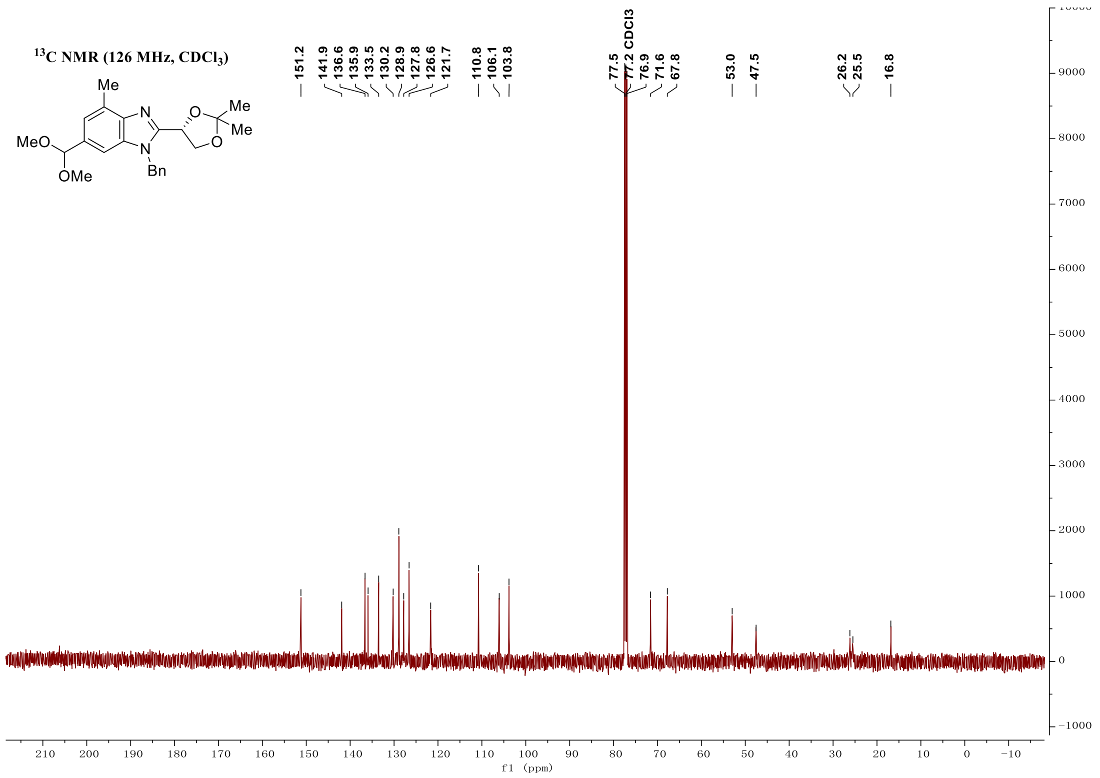

Supplementary Figure 133. ^{13}C NMR spectrum of compound 24.

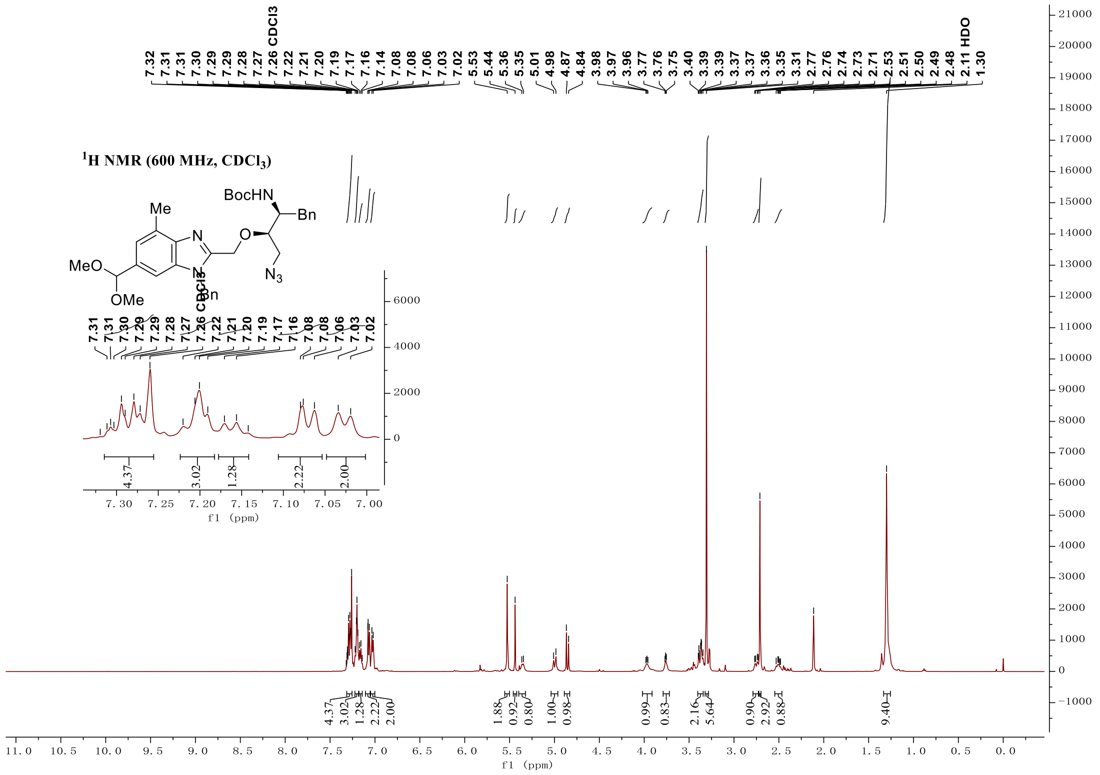

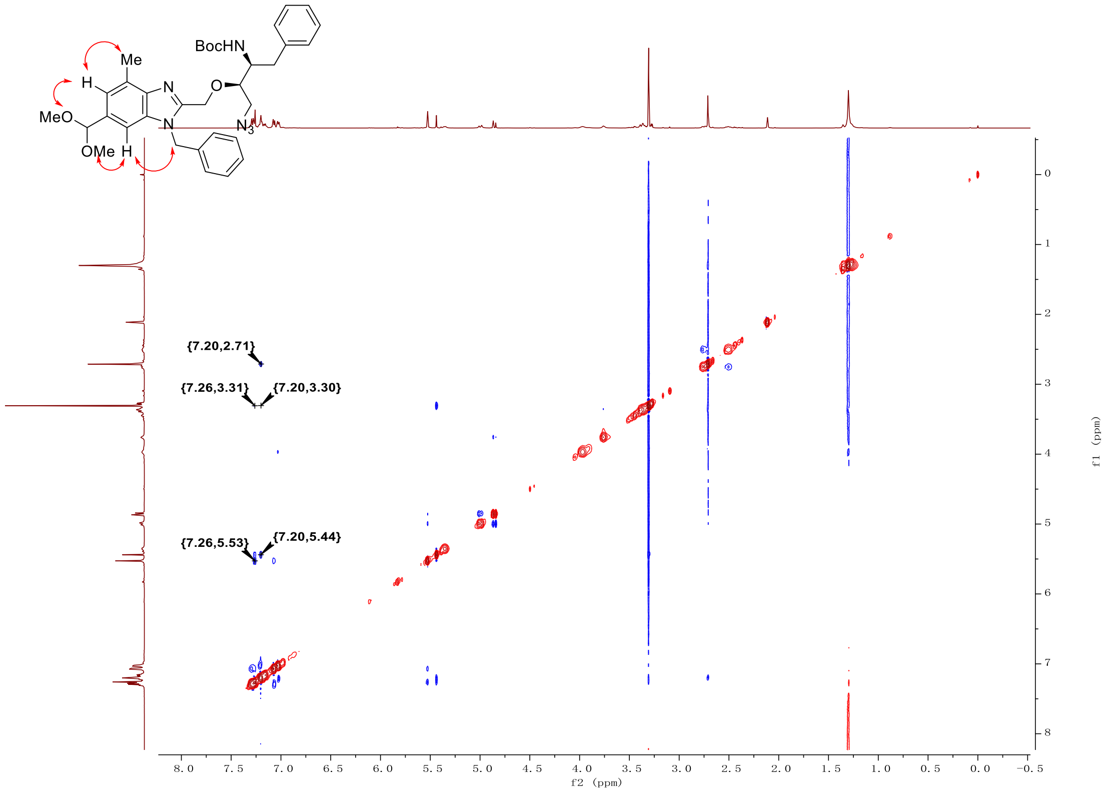

Supplementary Figure 134. ^{1}H NMR and 2D NOESY spectra of compound 25.

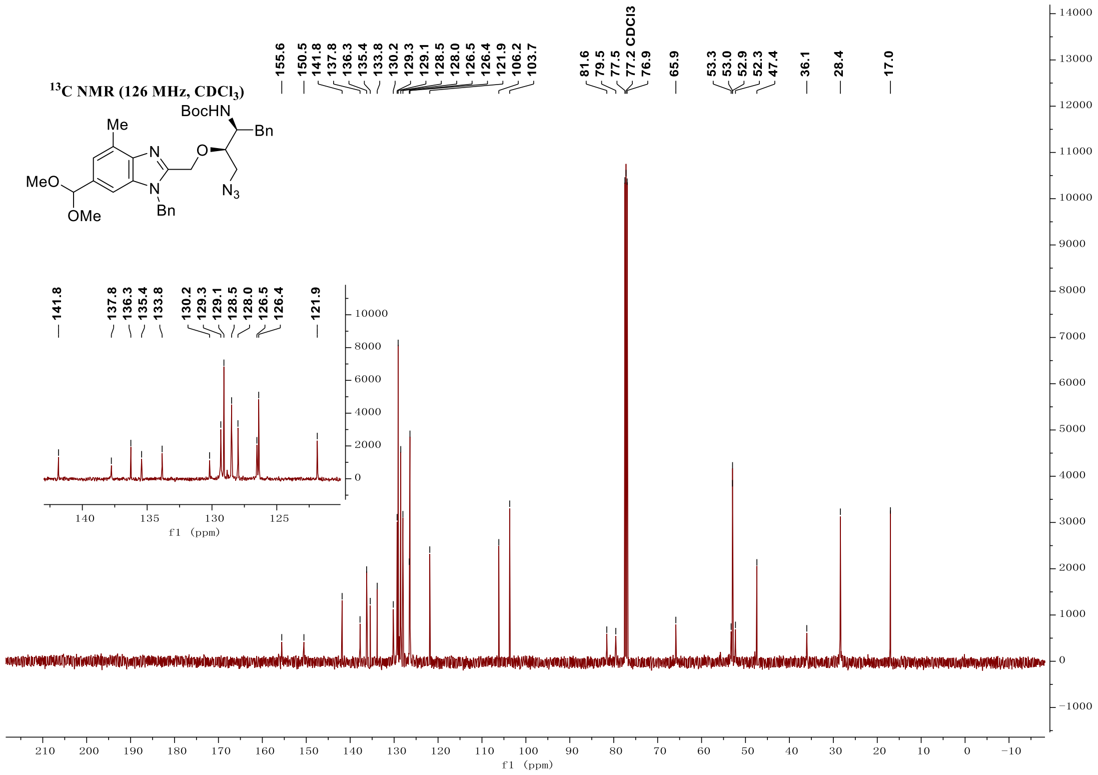

Supplementary Figure 135. ^{13}C NMR spectrum of compound 25.

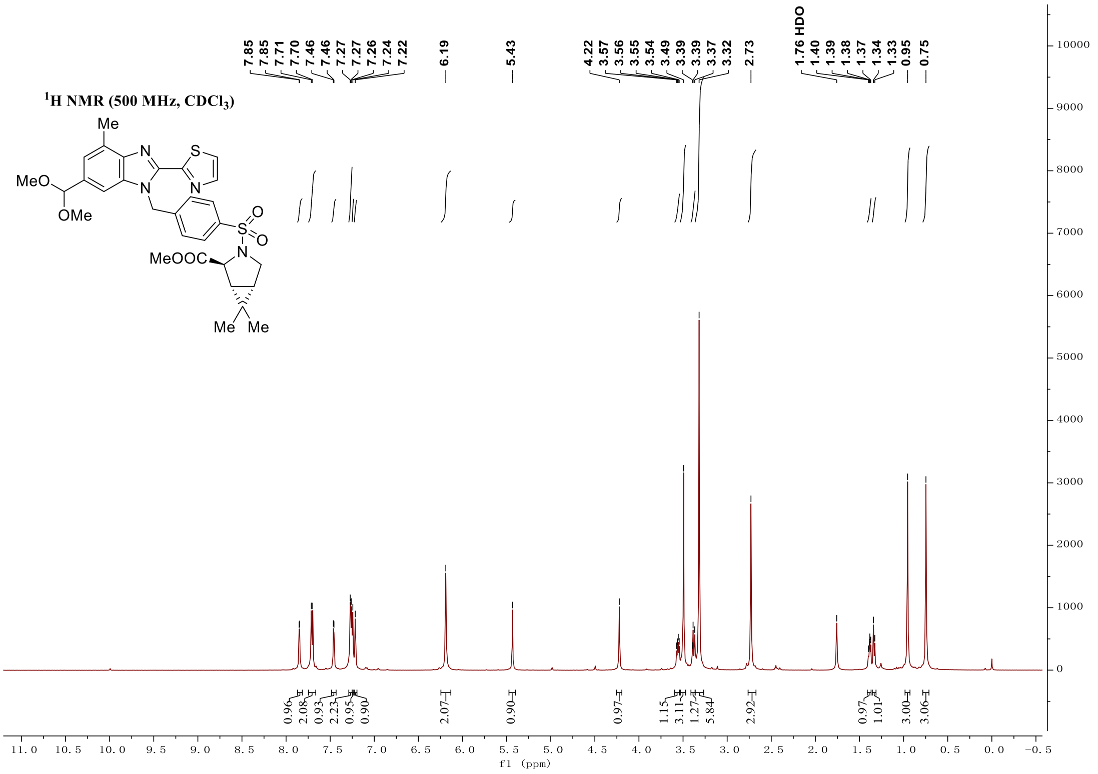

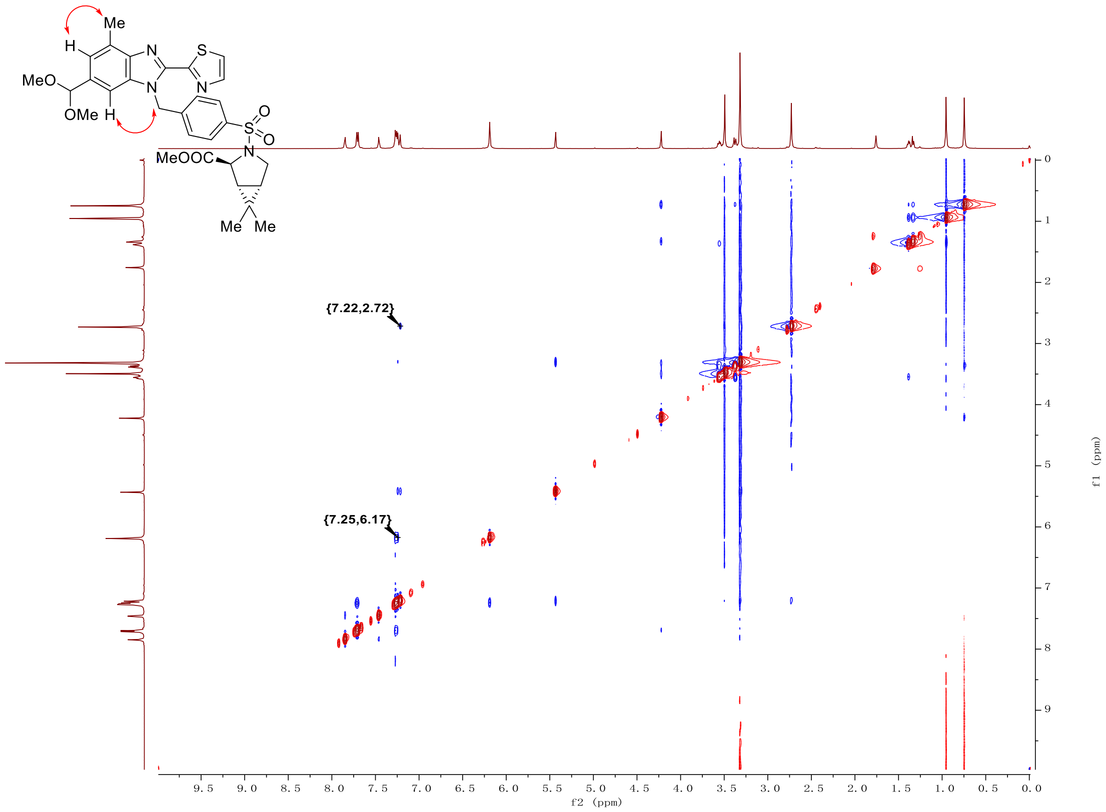

Supplementary Figure 136. ^{1}**H NMR and 2D NOESY spectra of compound 26.**

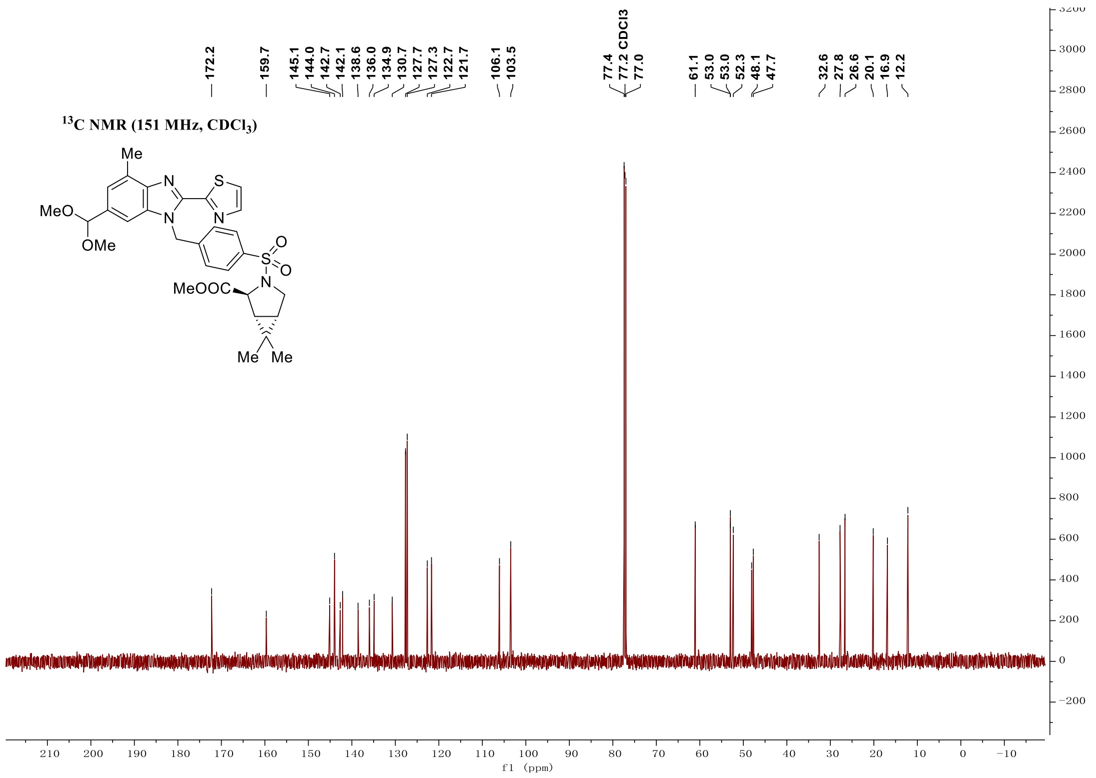

Supplementary Figure 137. ^{13}C NMR spectrum of compound 26.

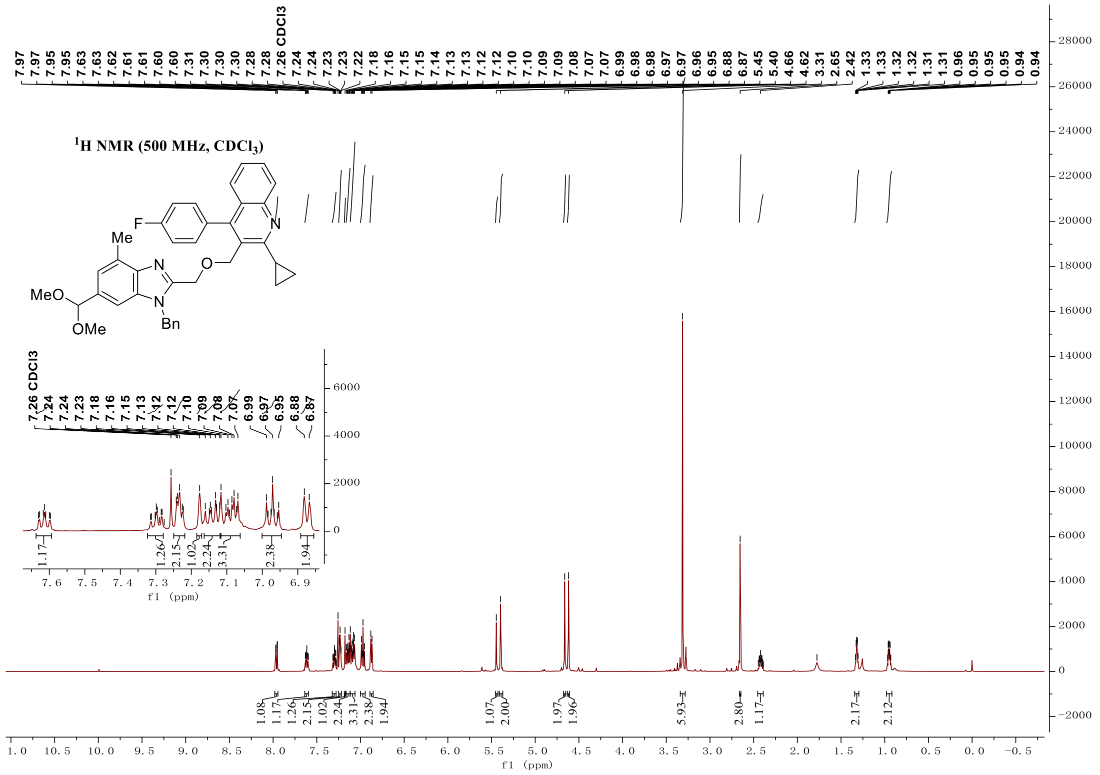

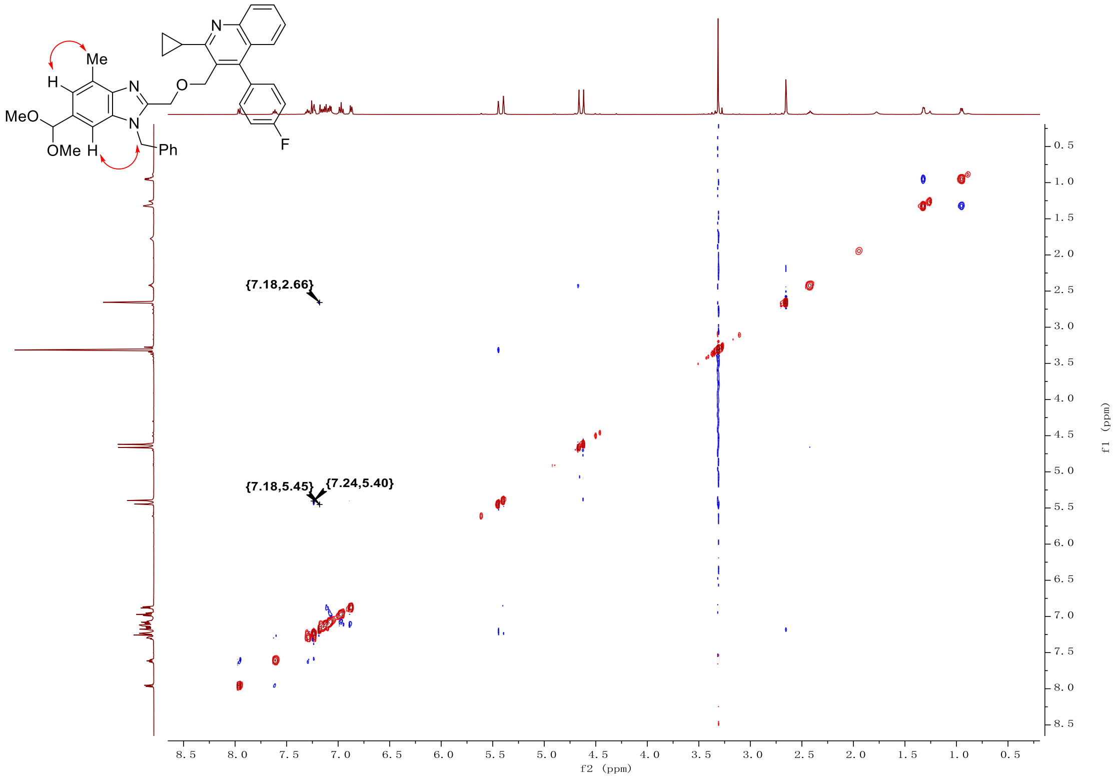

Supplementary Figure 138. ^{1}H NMR and 2D NOESY spectra of compound 27.

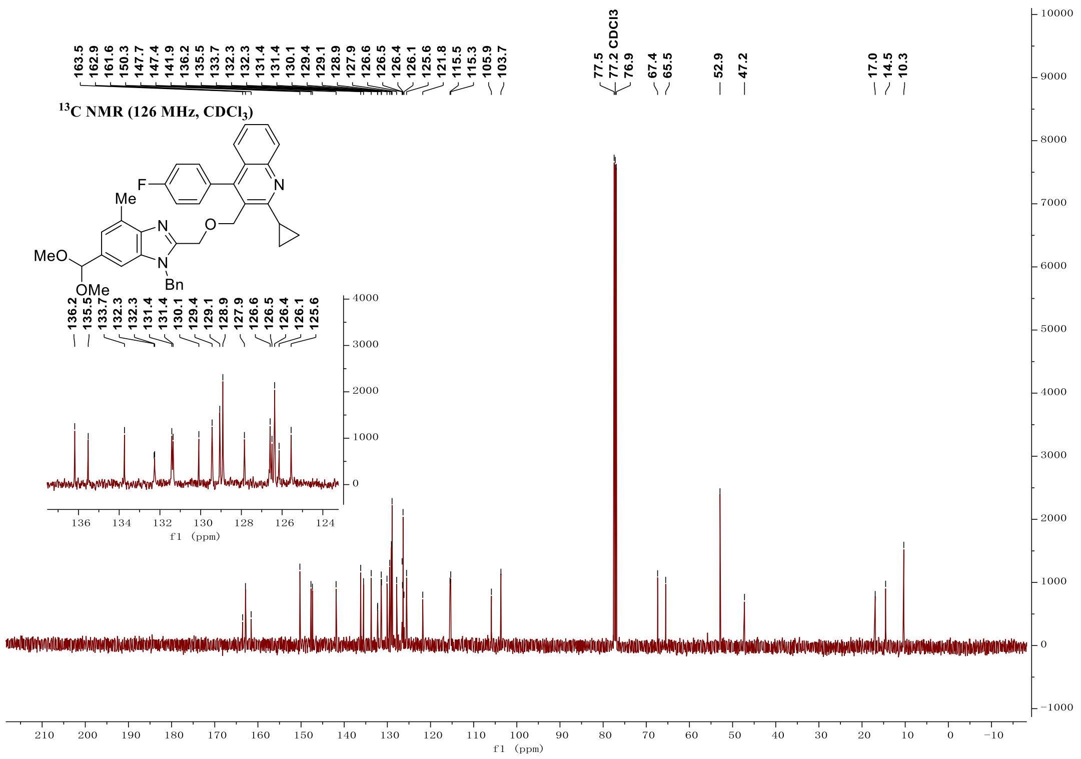

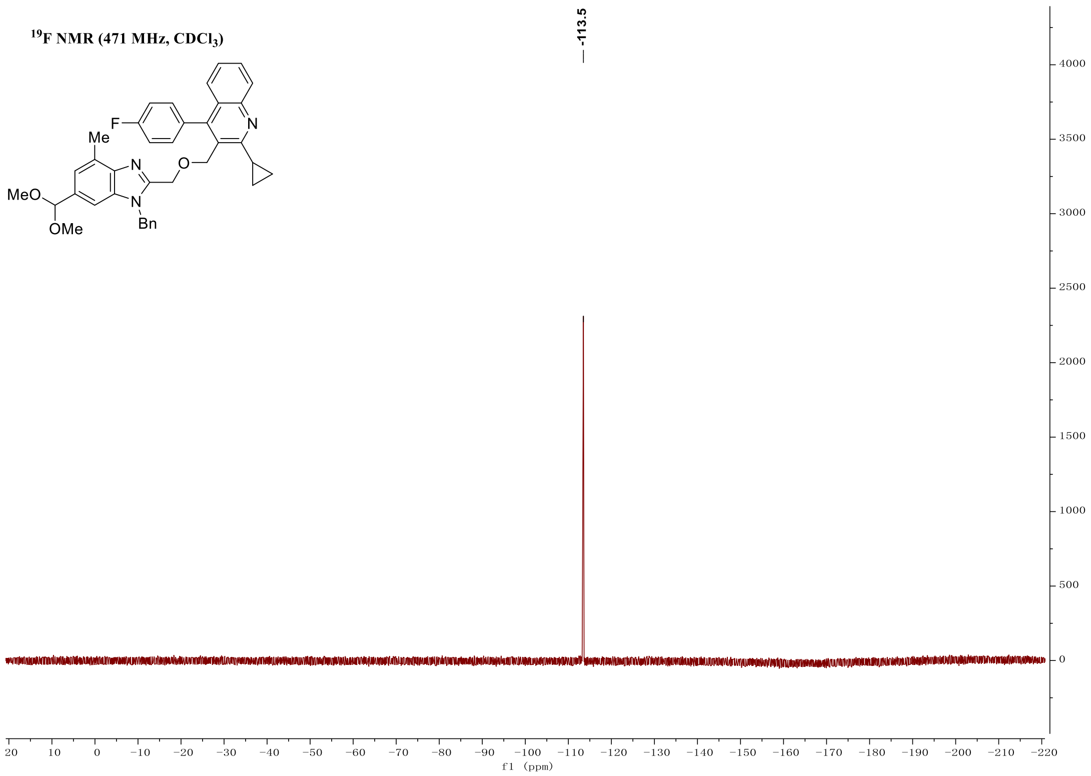

Supplementary Figure 139. ^{13}C **NMR and** ^{19}F **NMR spectra of compound 27.**

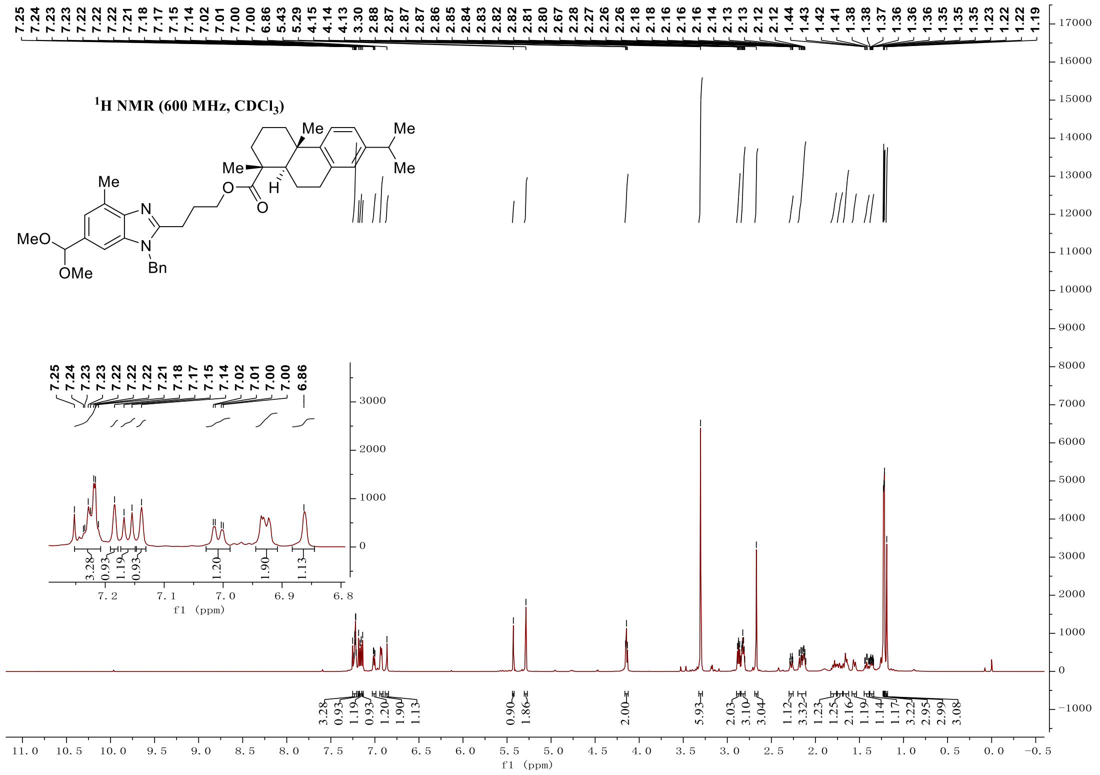

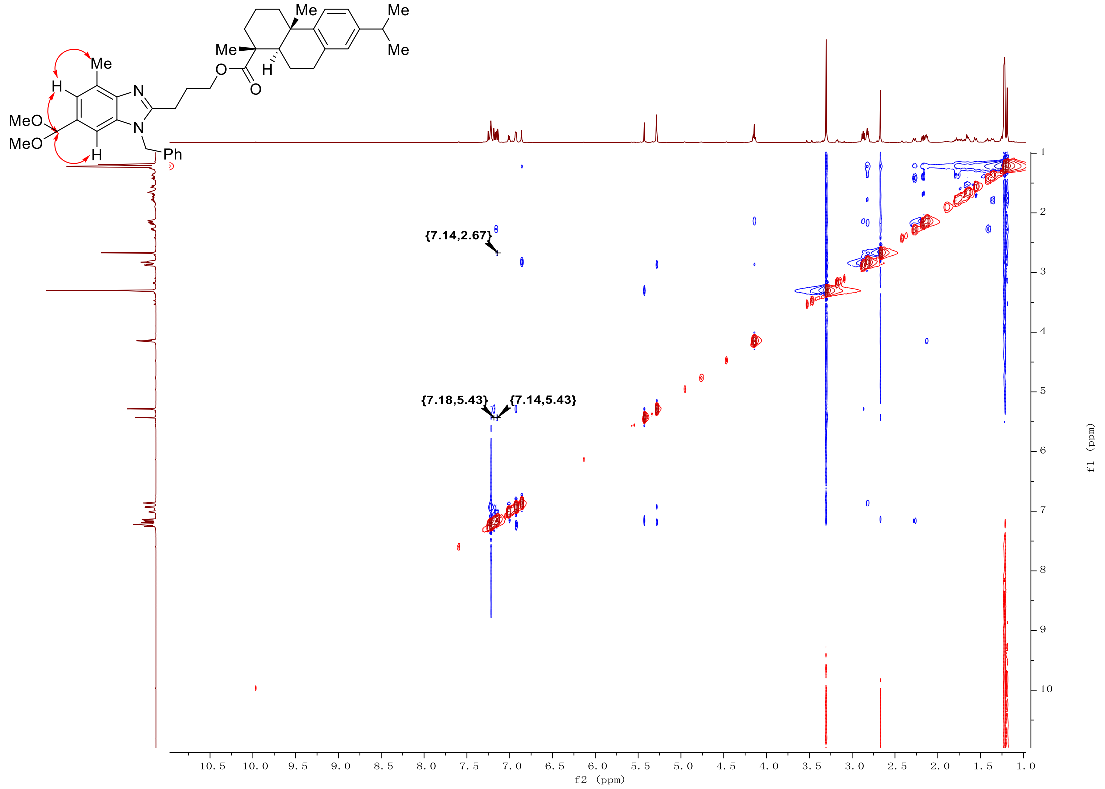

Supplementary Figure 140. ^{1}H NMR and 2D NOESY spectra of compound 28.

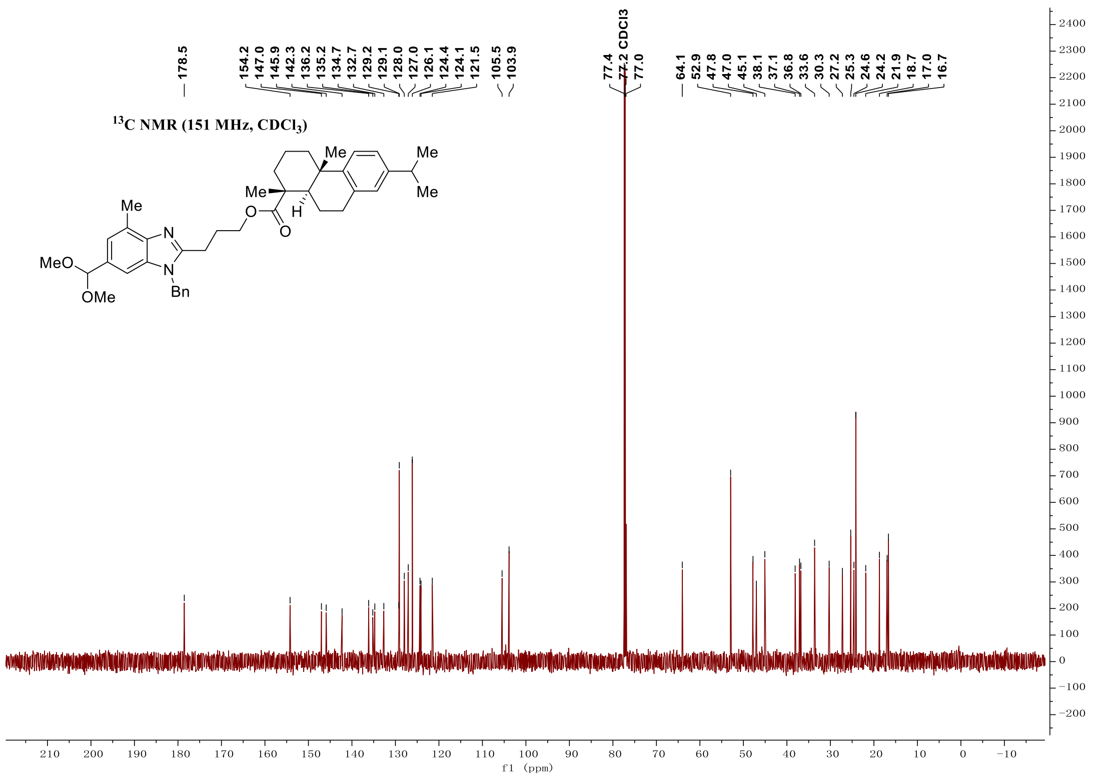

Supplementary Figure 141. ^{13}C NMR spectrum of compound 28.

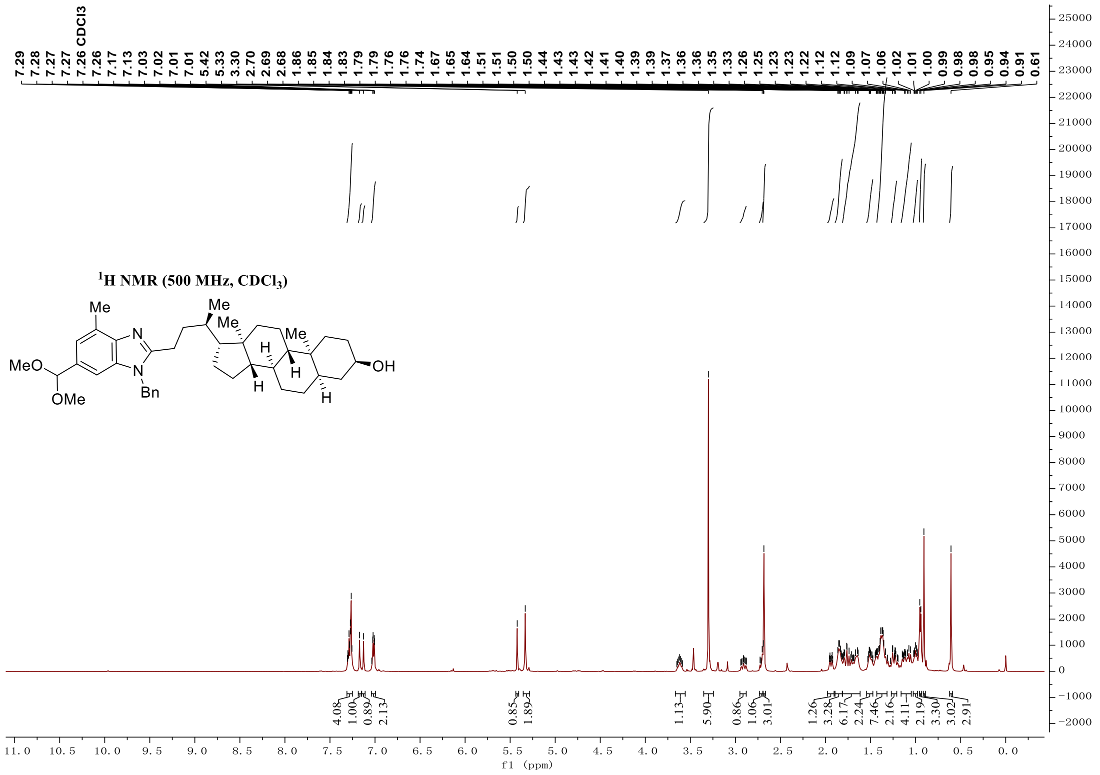

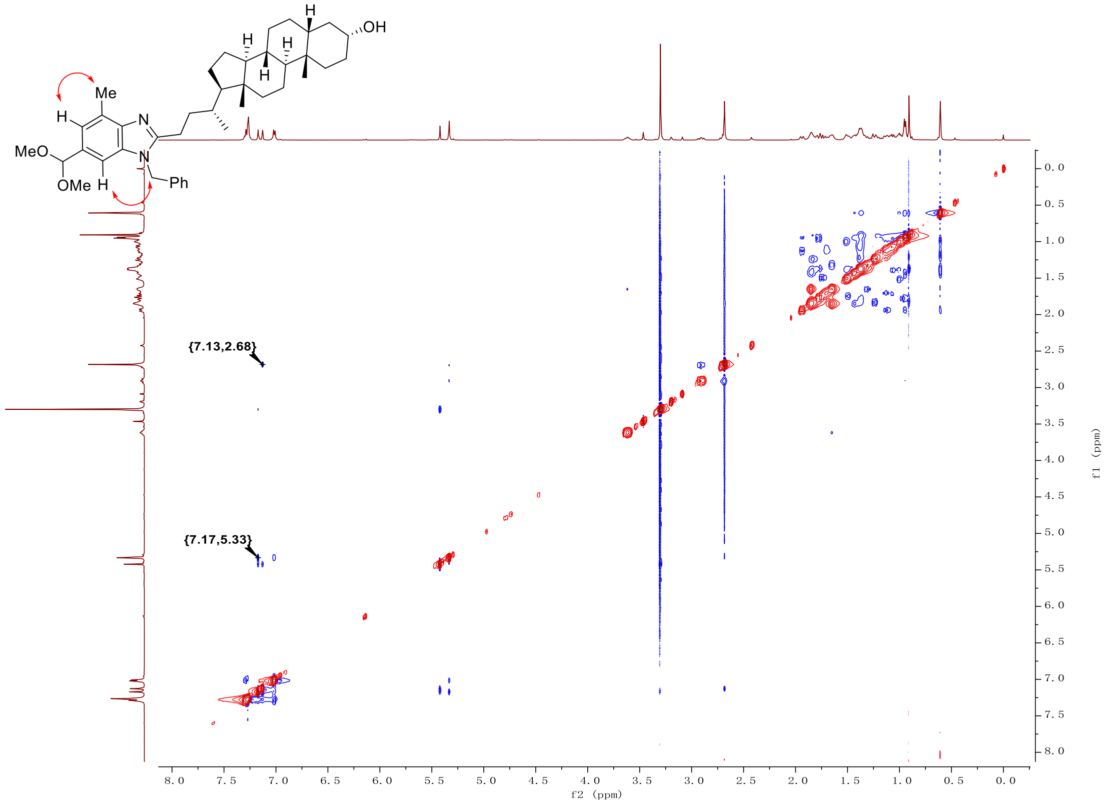

Supplementary Figure 142. ^{1}H NMR and 2D NOESY spectra of compound 29.

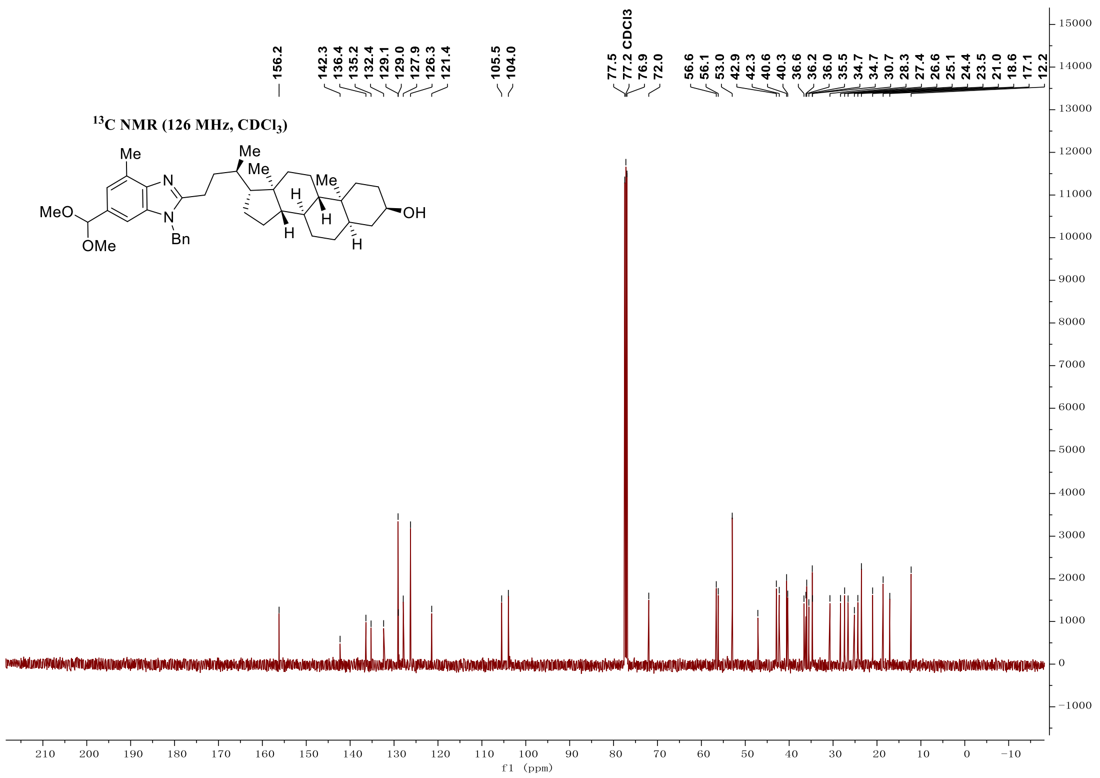

Supplementary Figure 143. ^{13}C NMR spectrum of compound 29.

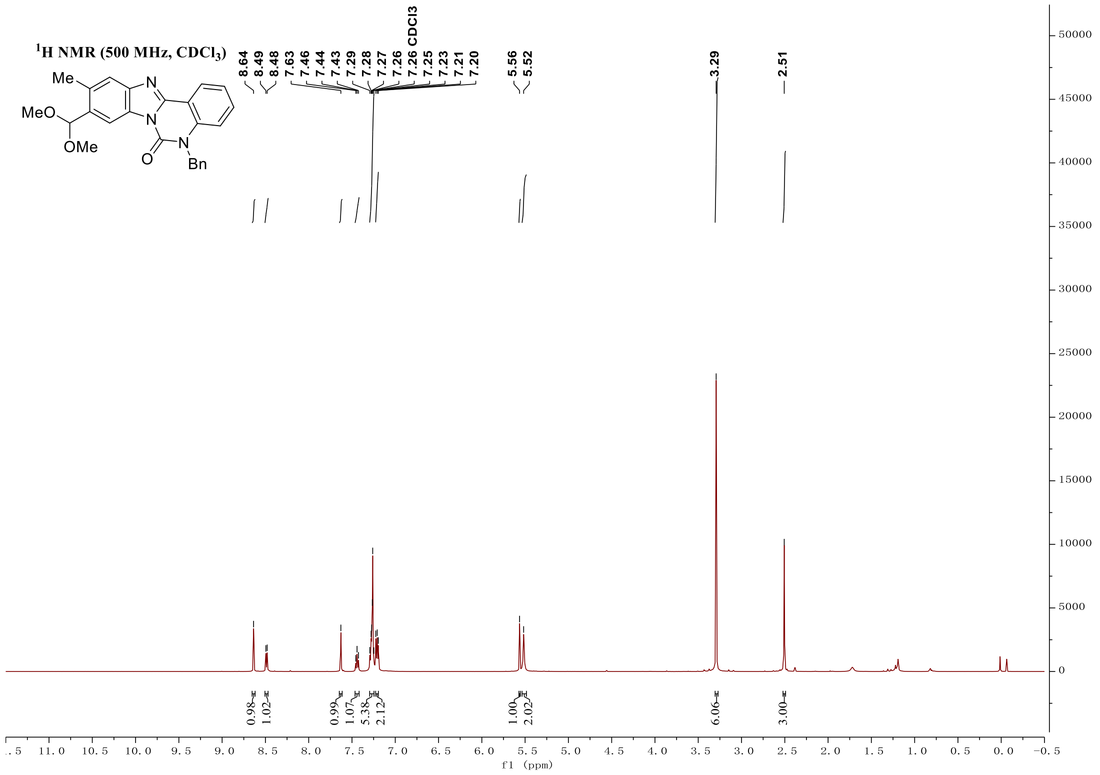

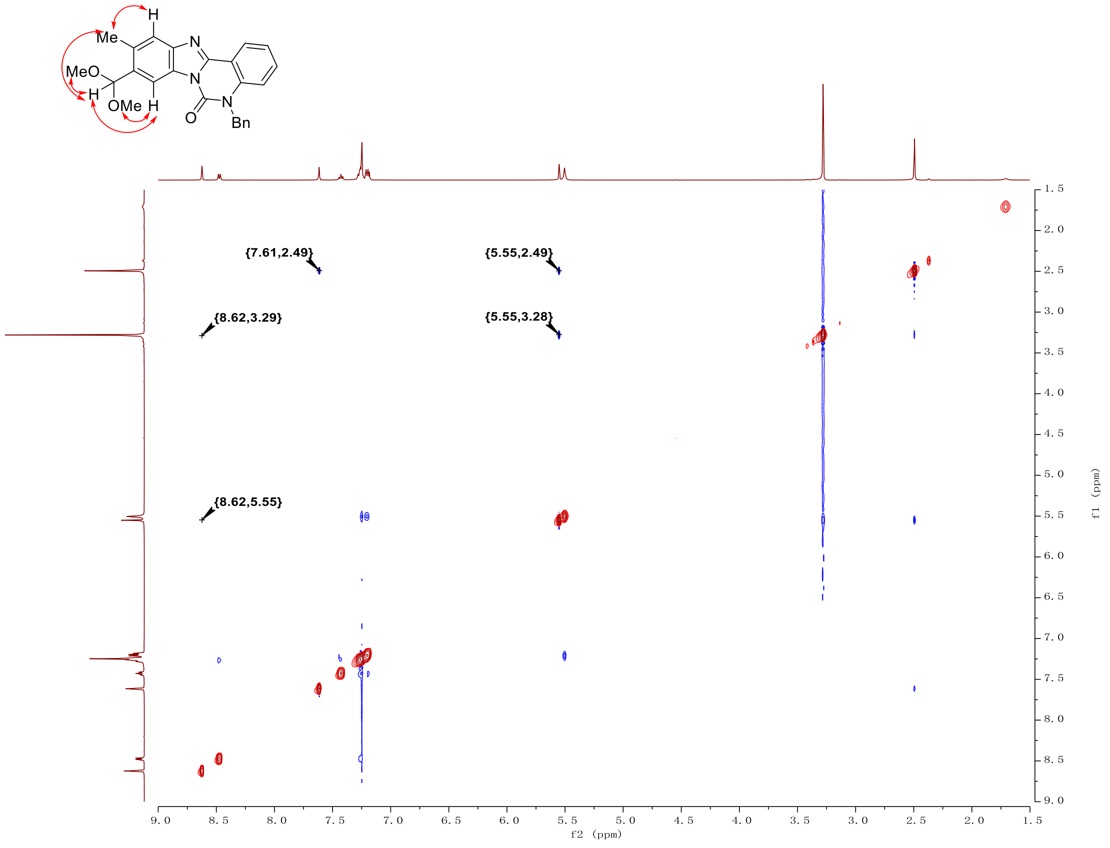

Supplementary Figure 144. ^{1}H NMR and 2D NOESY spectra of compound 30.

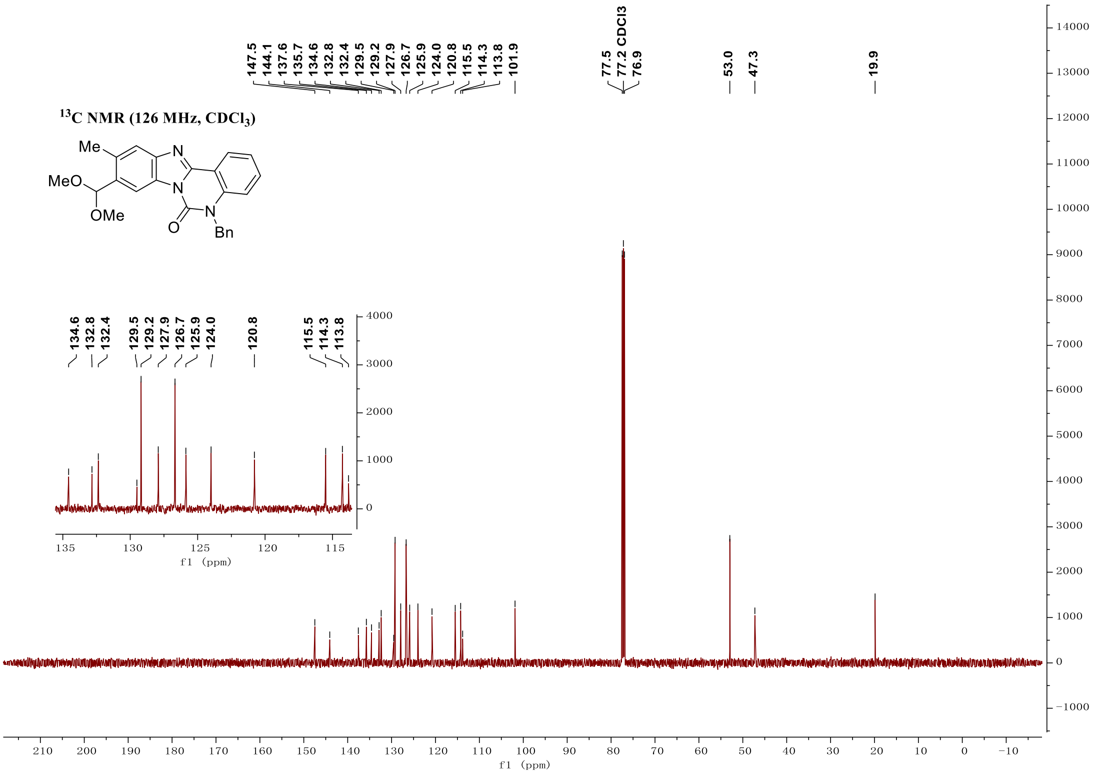

Supplementary Figure 145. ^{13}C NMR spectrum of compound 30.

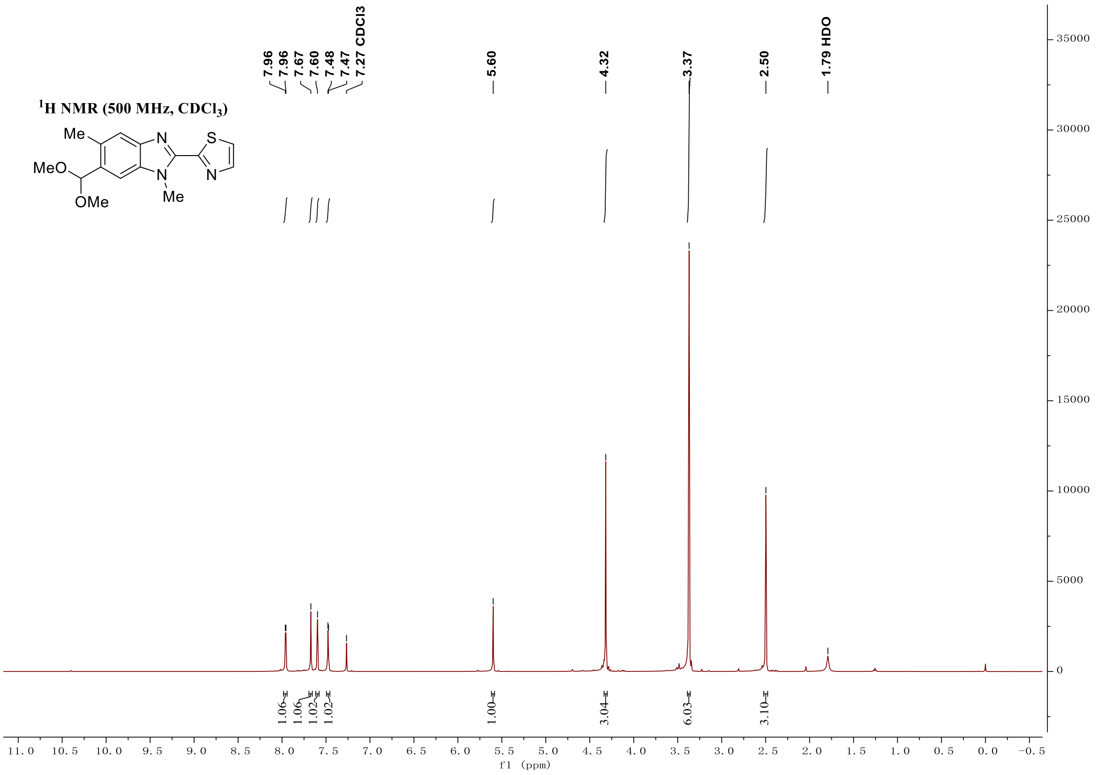

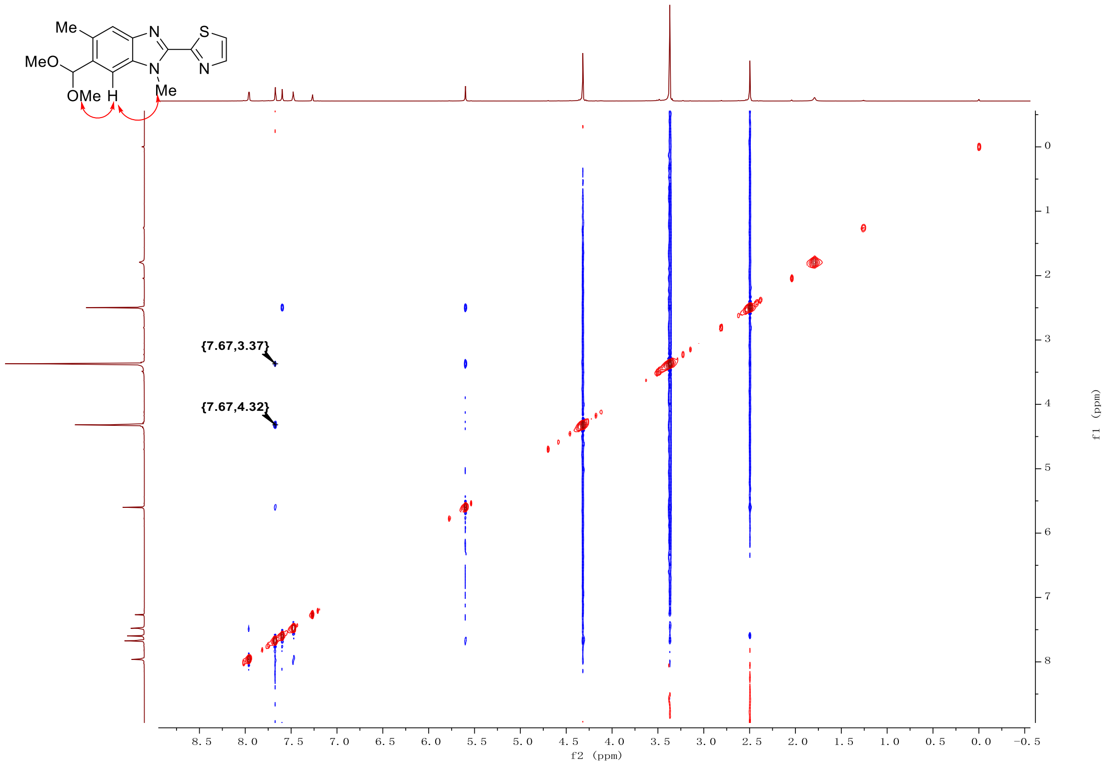

Supplementary Figure 146. ^{1}H NMR and 2D NOESY spectra of compound 31.

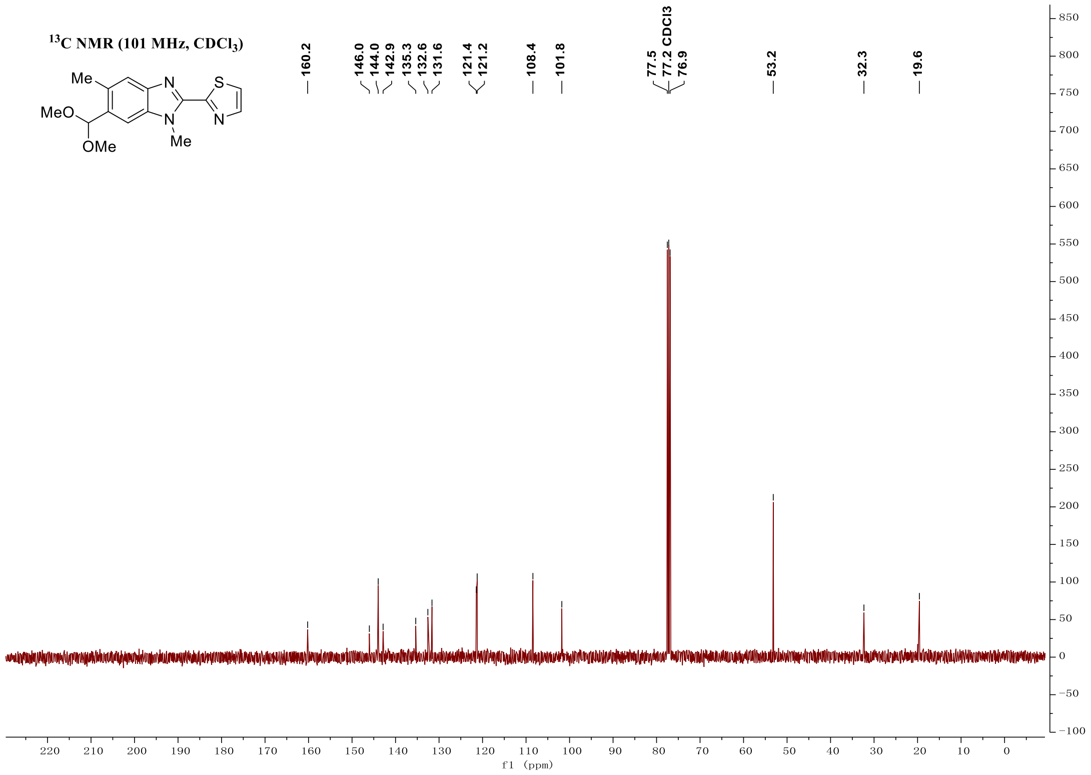

Supplementary Figure 147. ^{13}C NMR spectrum of compound 31.

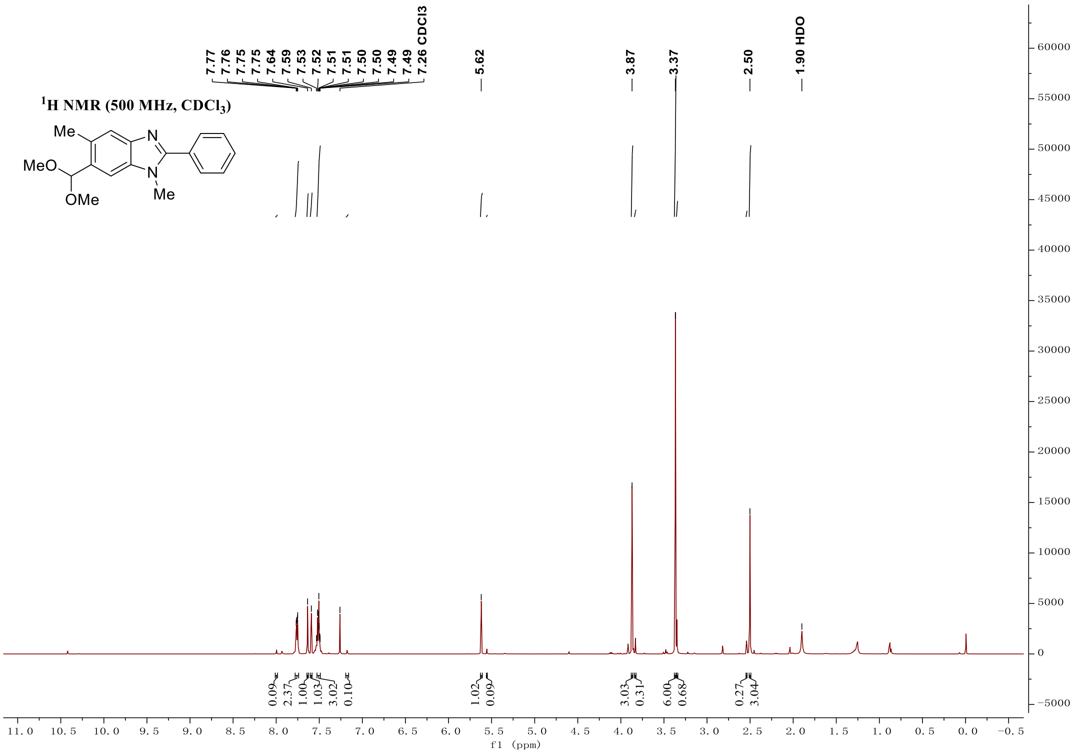

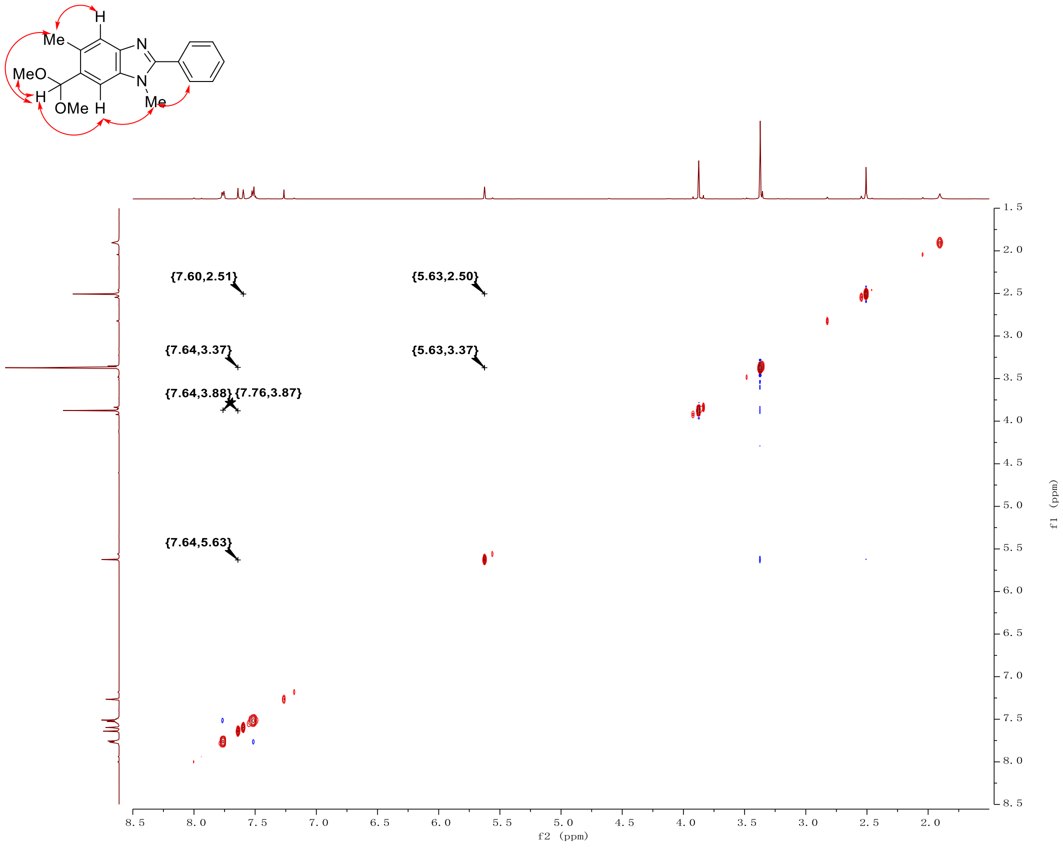

Supplementary Figure 148. ^{1}H NMR and 2D NOESY spectra of compound 32.

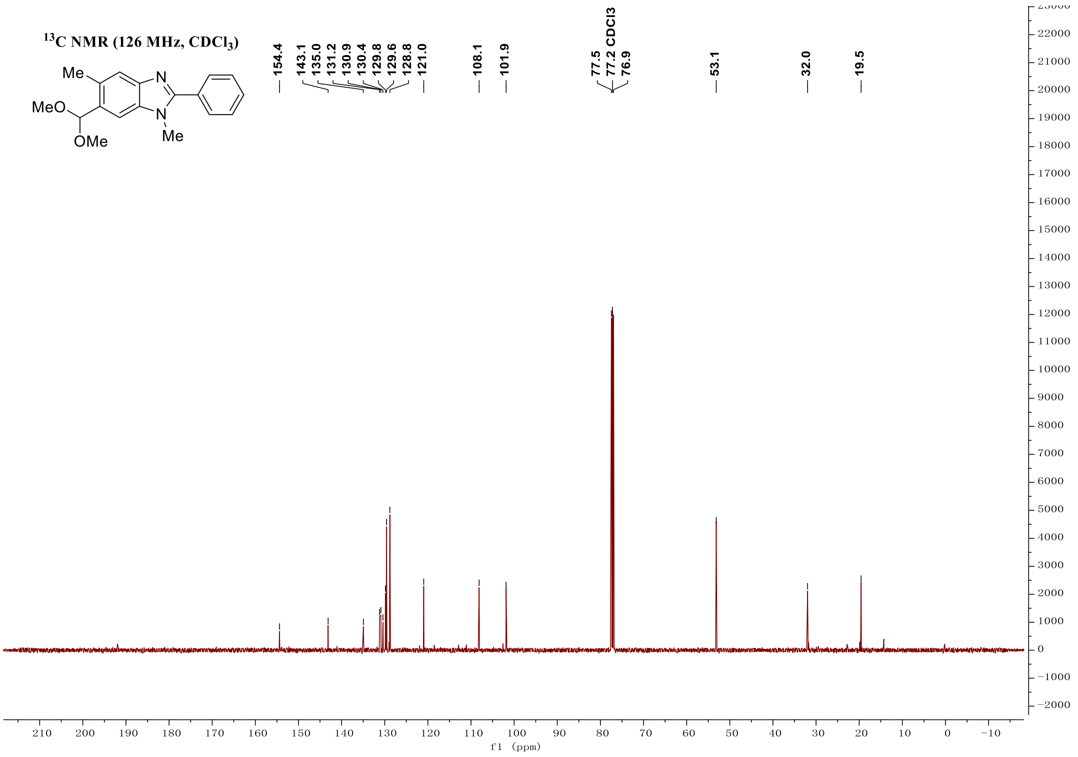

Supplementary Figure 149. ^{13}**C NMR spectrum of compound 32.**

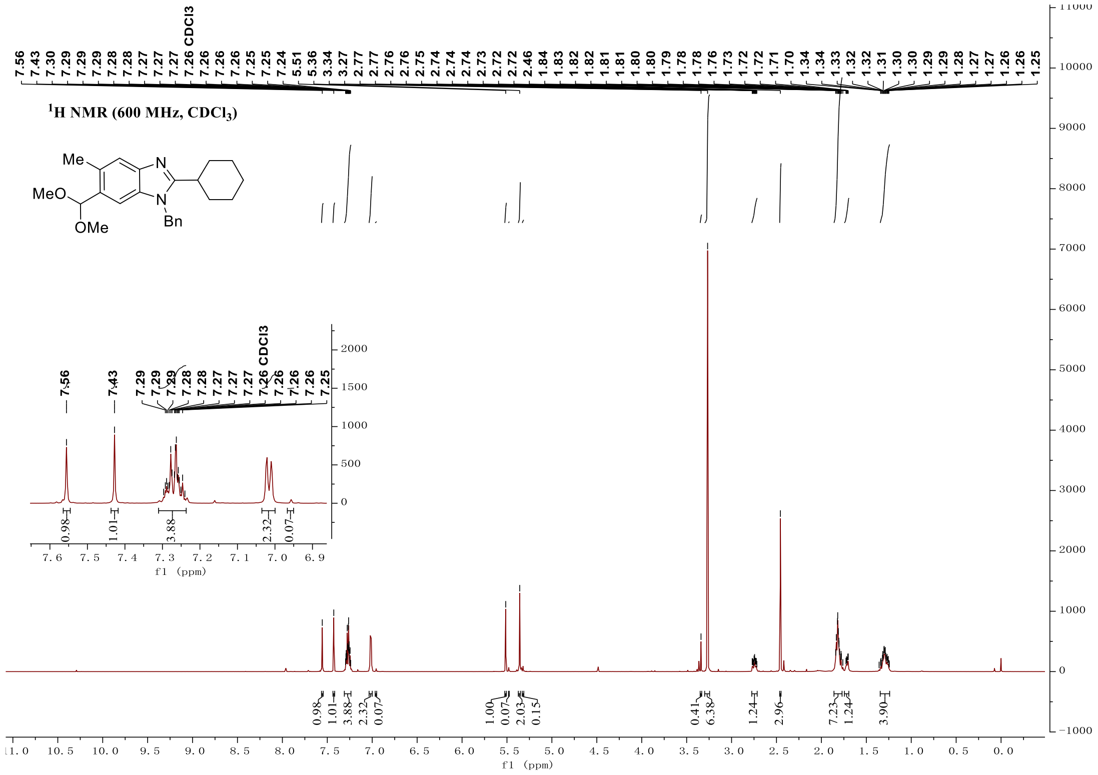

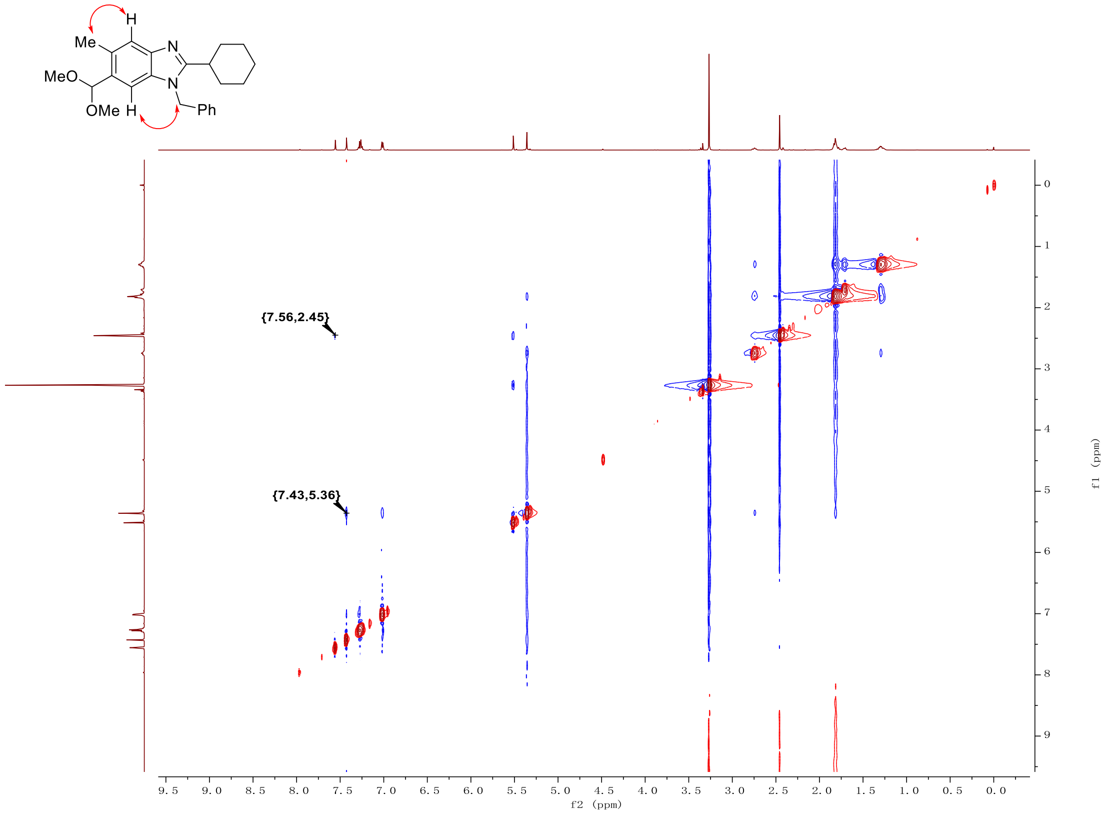

Supplementary Figure 150. ^{1}**H NMR and 2D NOESY spectra of compound 33.**

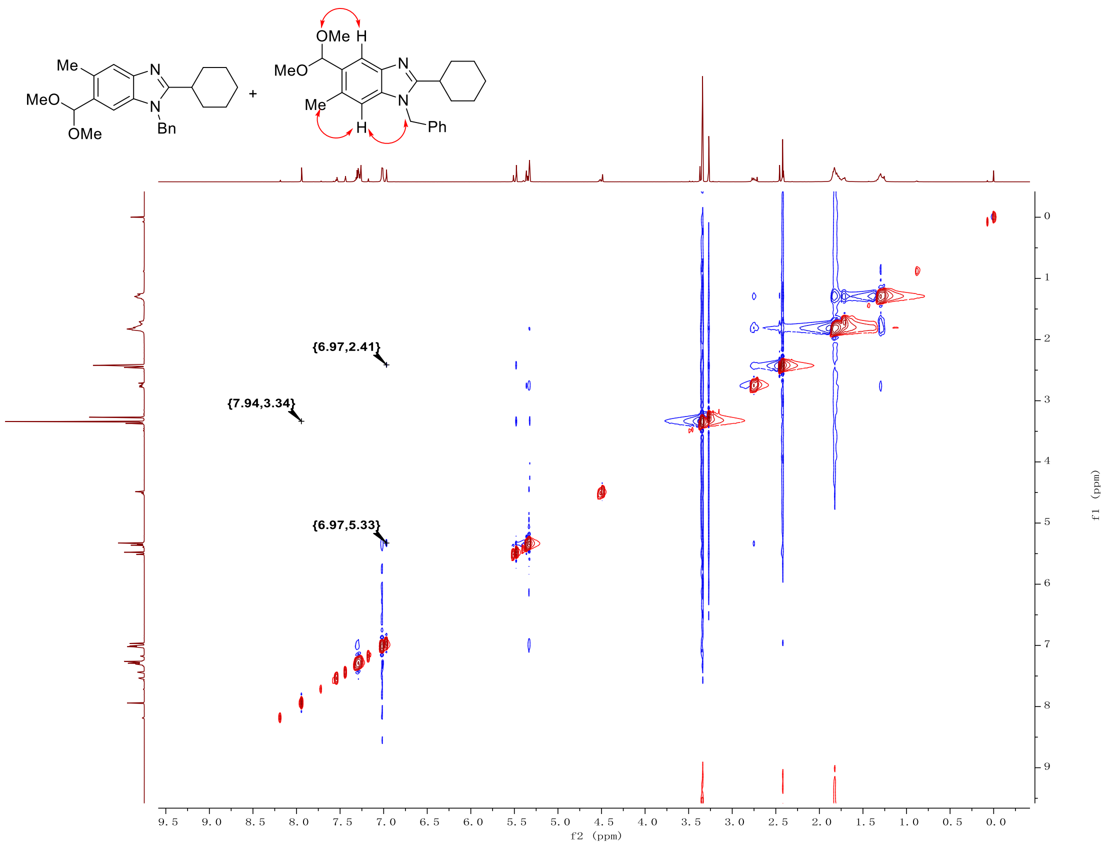

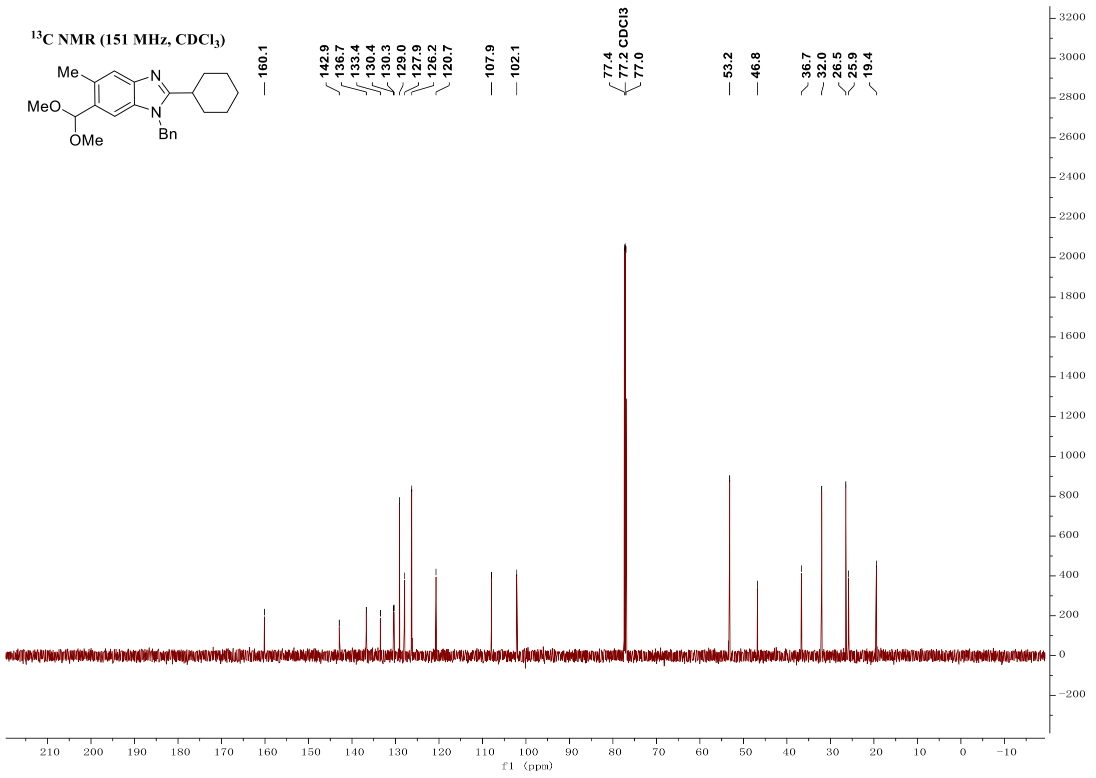

Supplementary Figure 151. 2D NOESY and ^{13}C NMR spectra of compound 33.

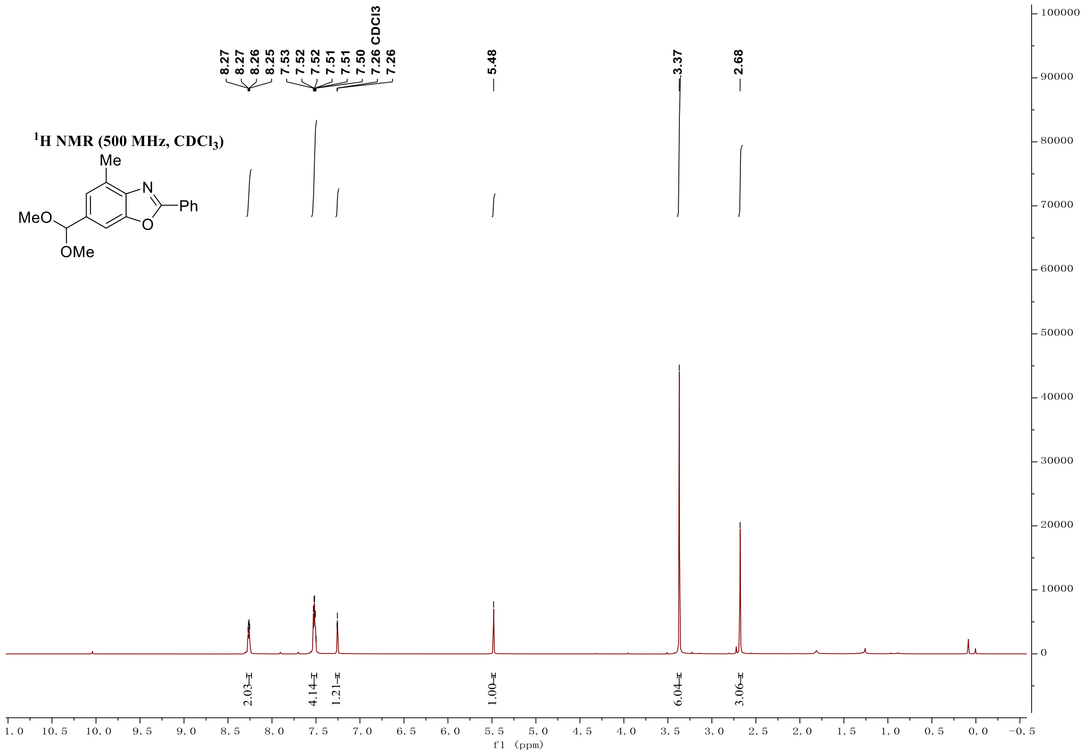

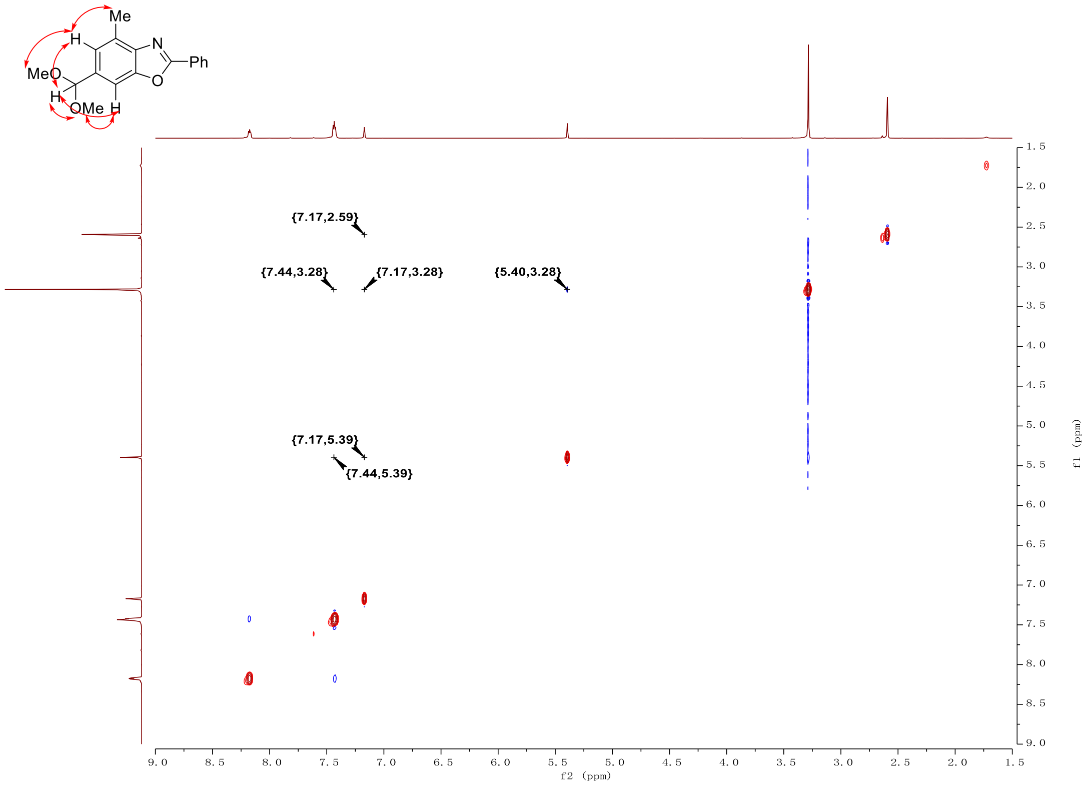

Supplementary Figure 152. ^{1}H NMR and 2D NOESY spectra of compound 34.

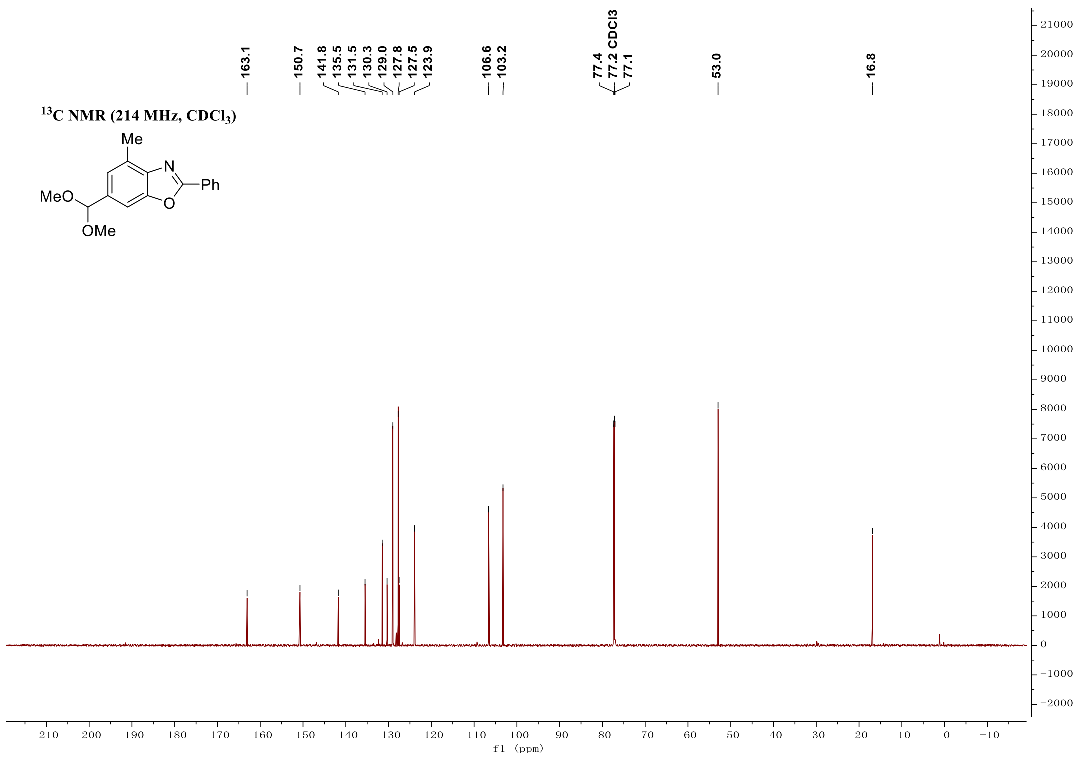

Supplementary Figure 153. ^{13}C NMR spectrum of compound 34.

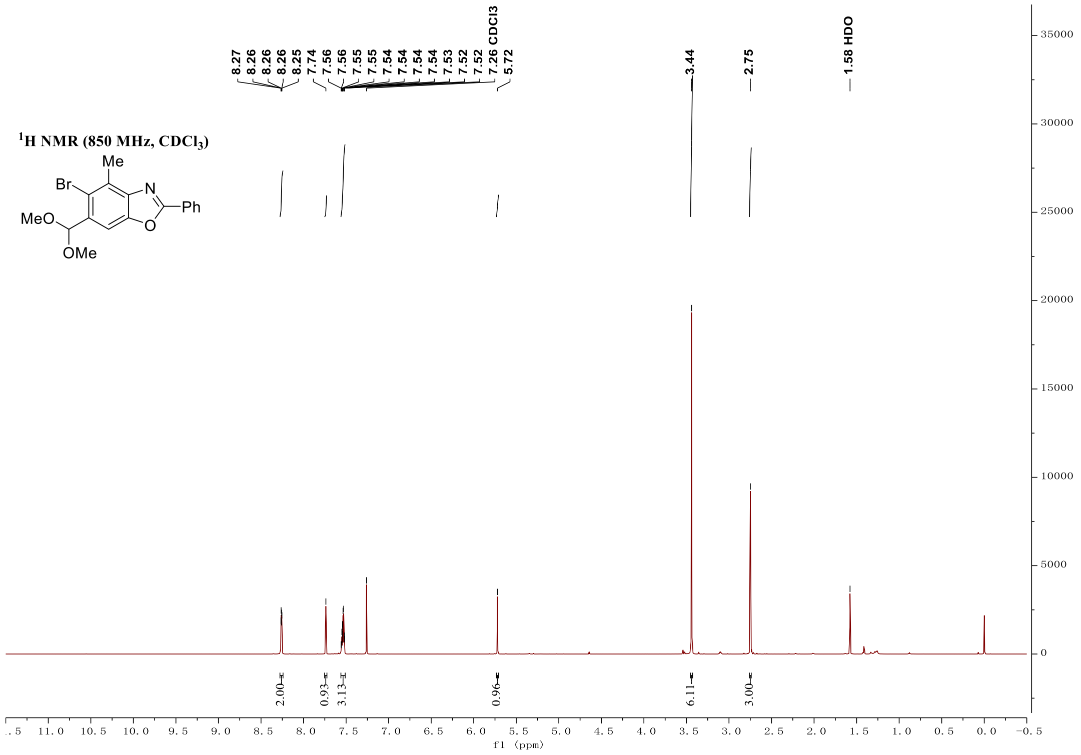

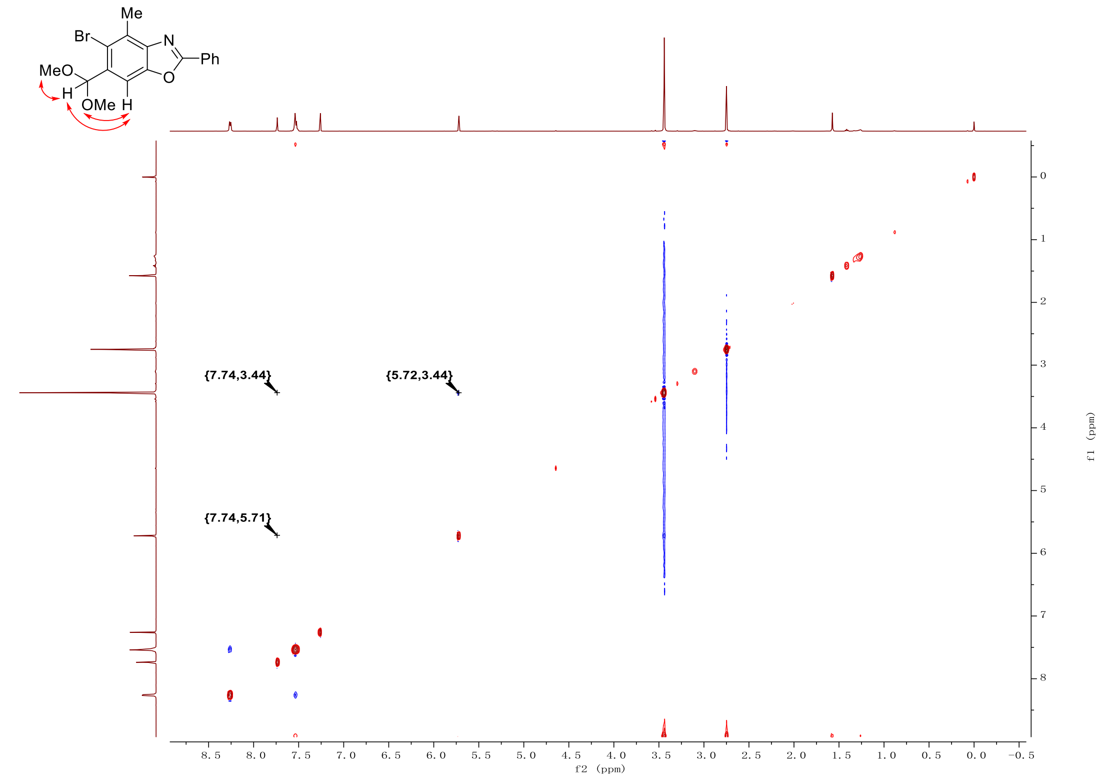

Supplementary Figure 154. ^{1}H NMR and 2D NOESY spectra of compound 35.

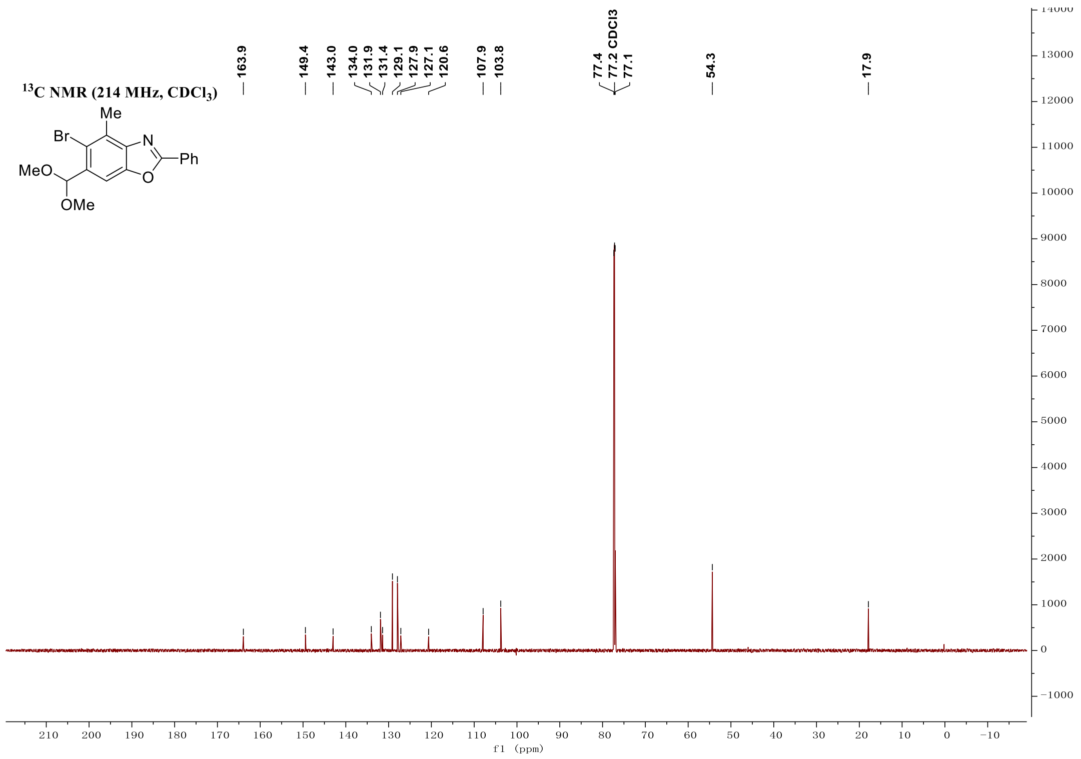

Supplementary Figure 155. ^{13}C NMR spectrum of compound 35.

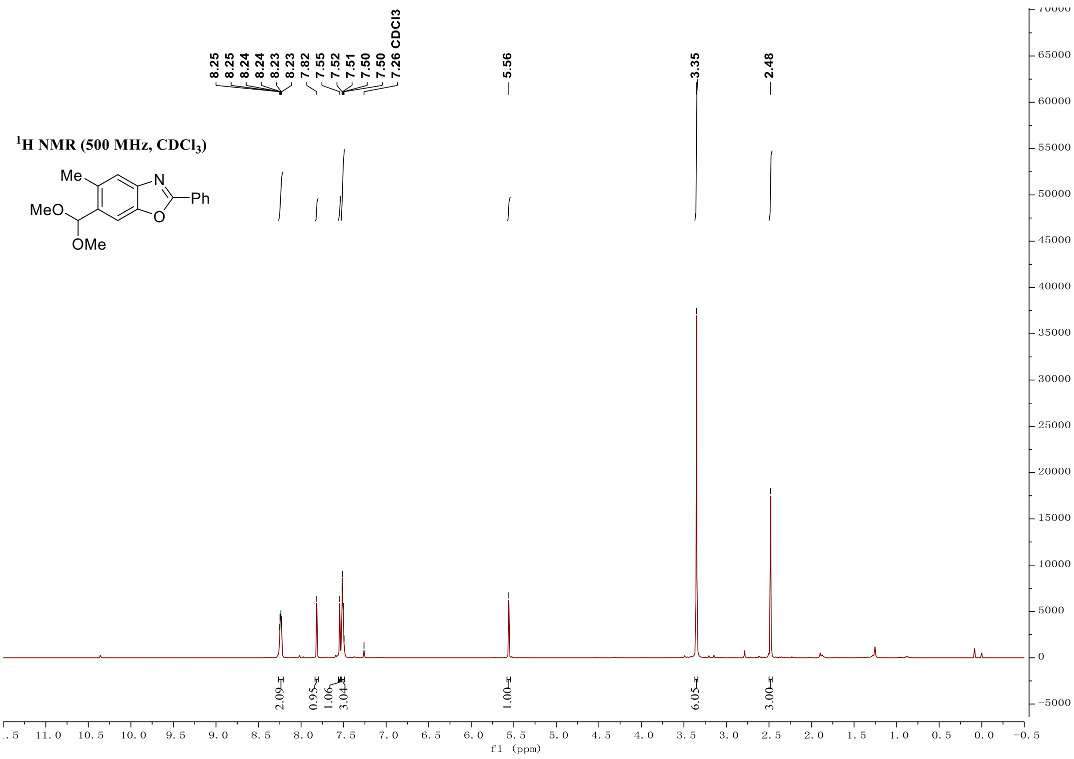

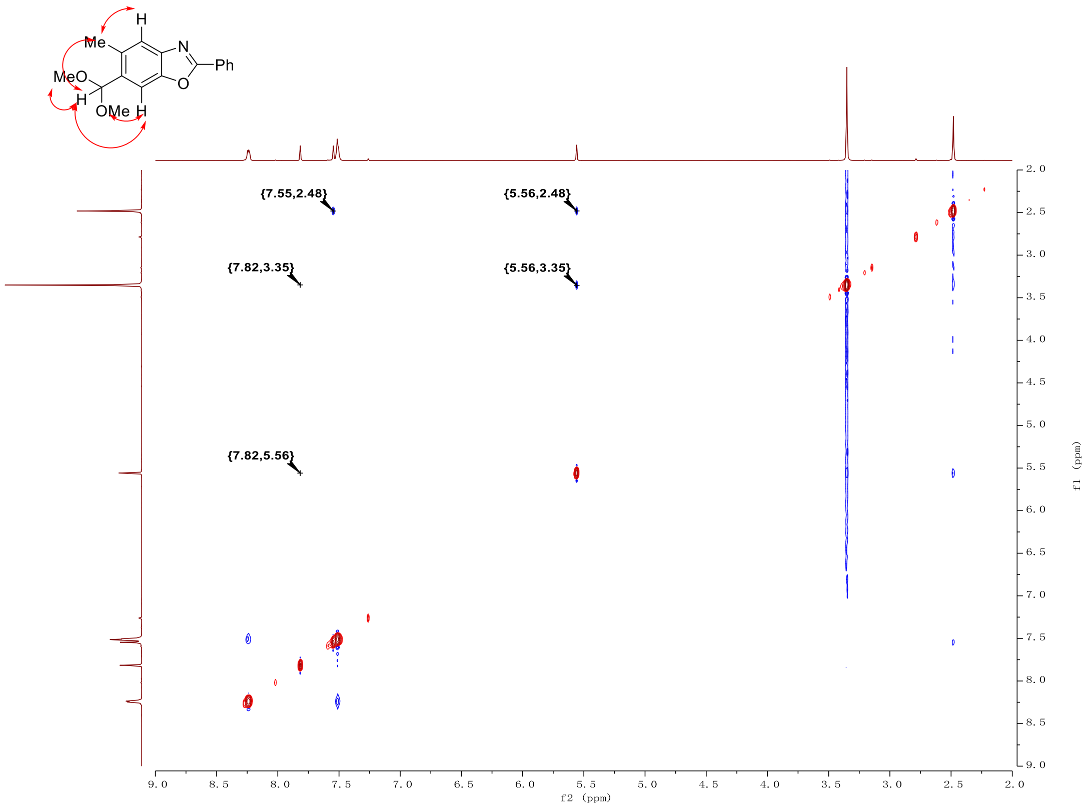

Supplementary Figure 156. ^{1}H NMR and 2D NOESY spectra of compound 36.

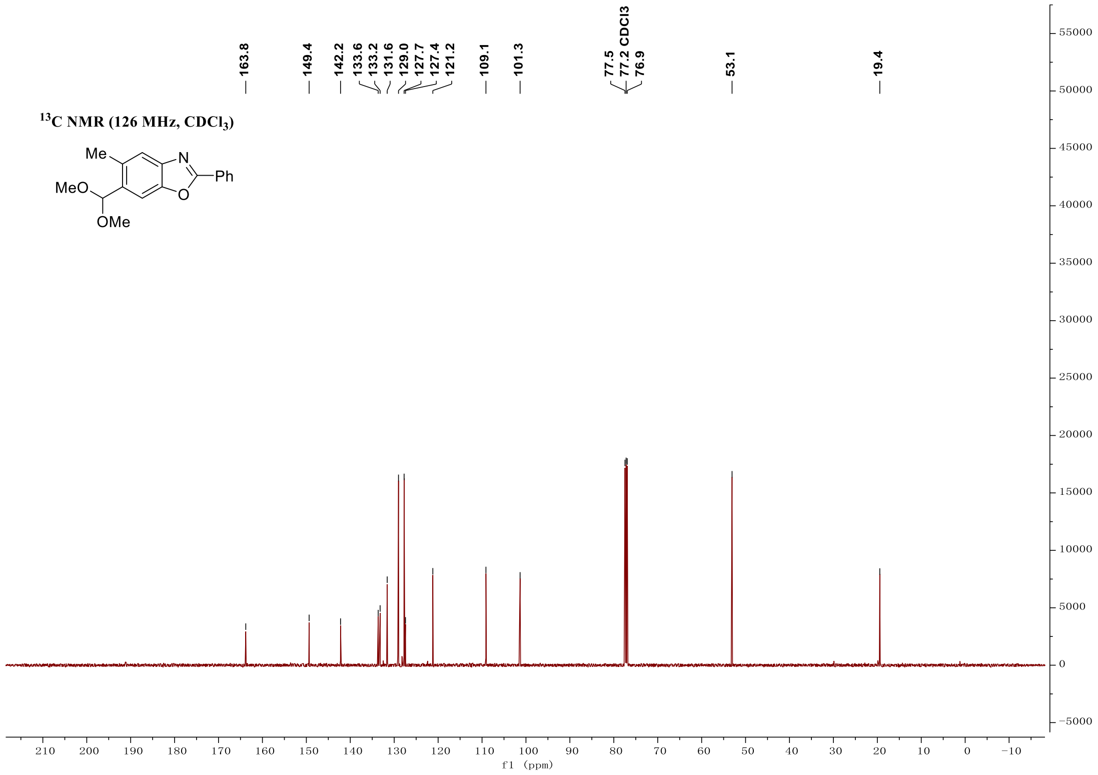

Supplementary Figure 157. ^{13}C NMR spectrum of compound 36.

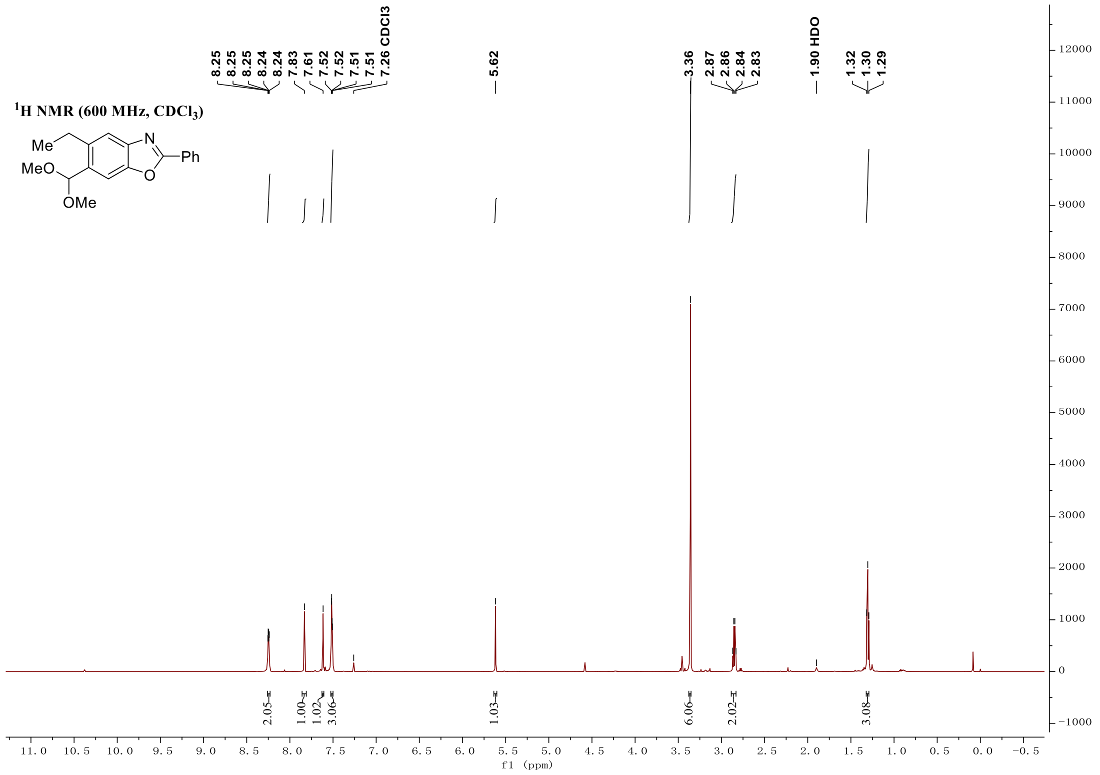

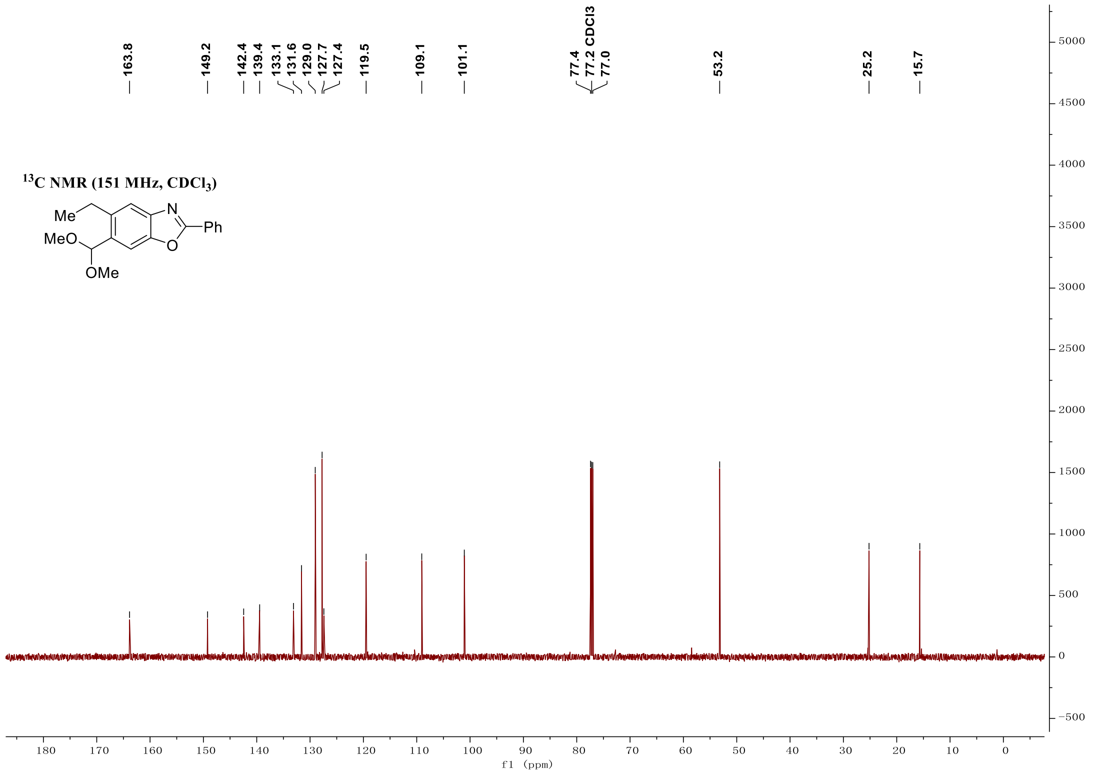

Supplementary Figure 158. ¹H NMR and ¹³C NMR spectra of compound 37.

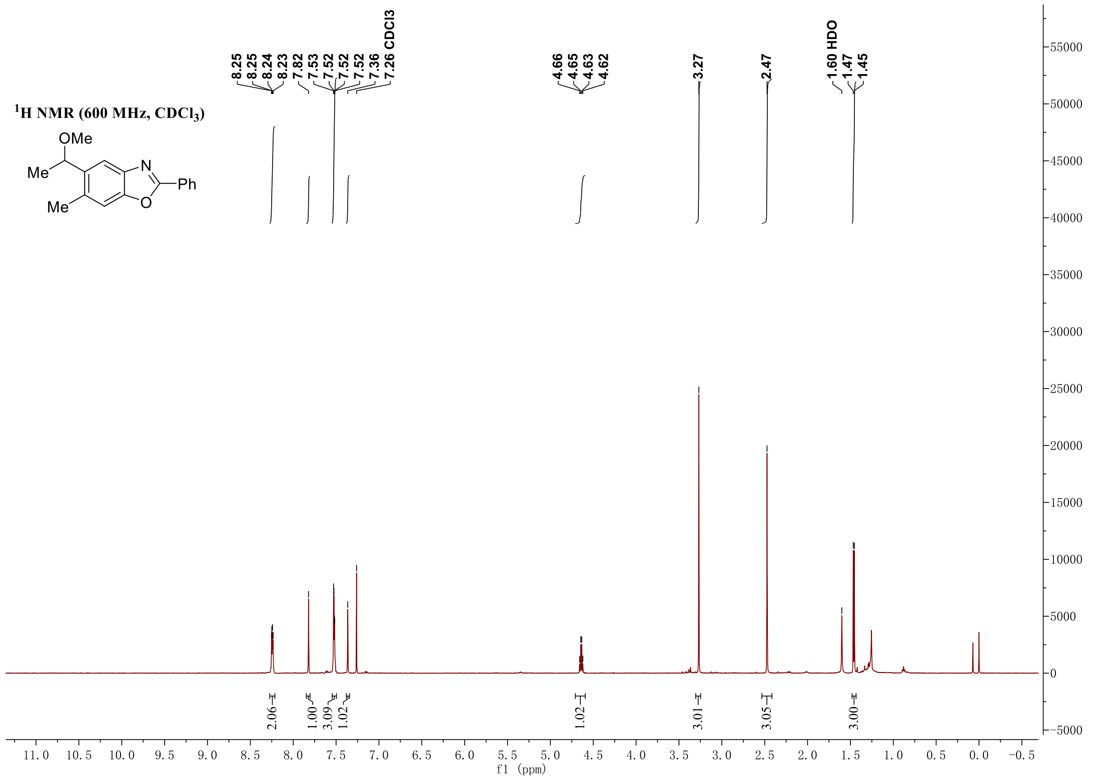

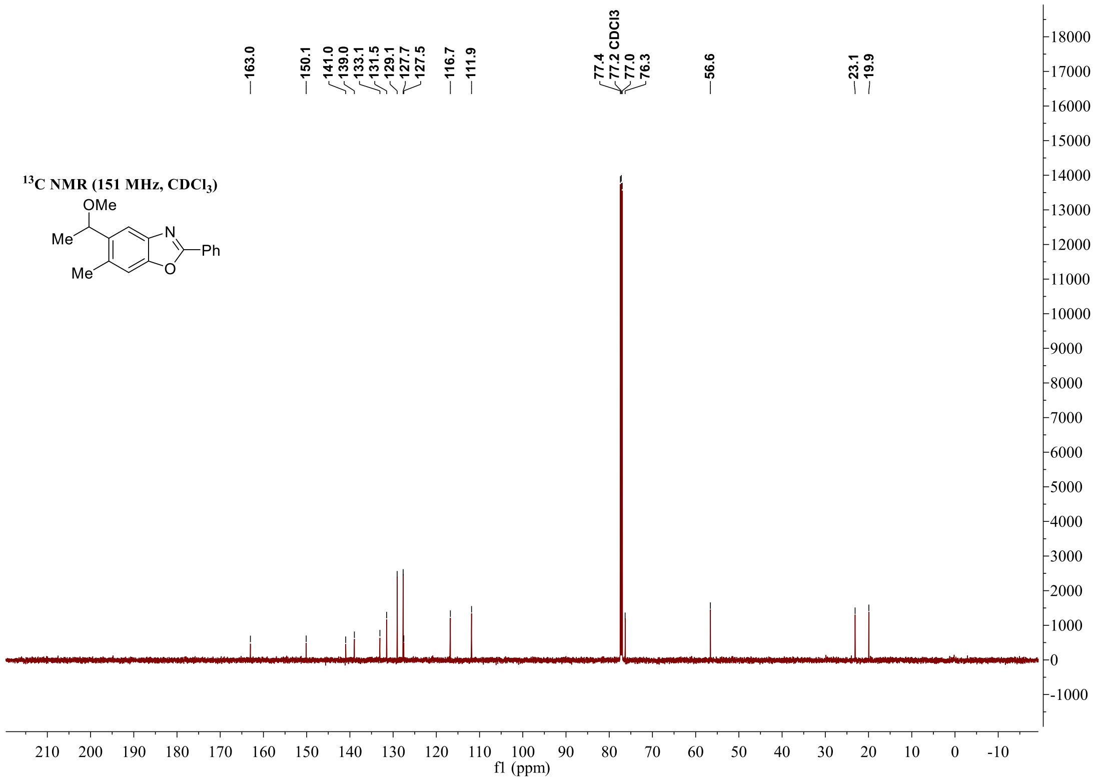

Supplementary Figure 159. ^{1}H NMR and ^{13}C NMR spectra of compound 37'.

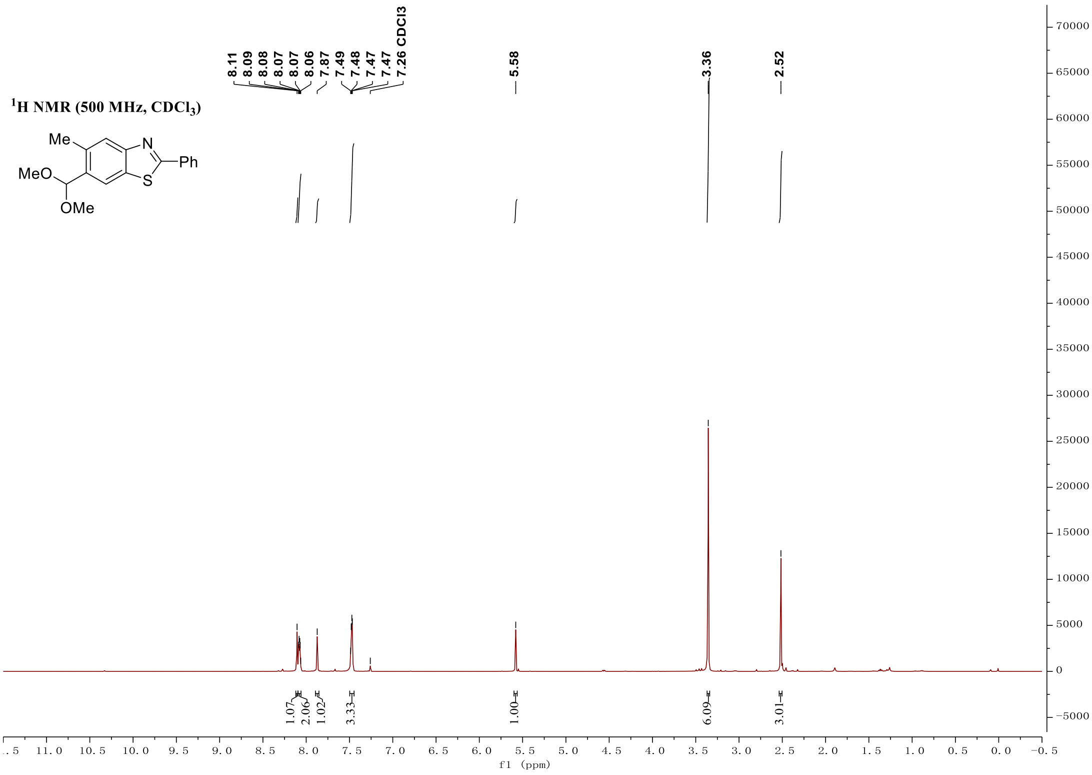

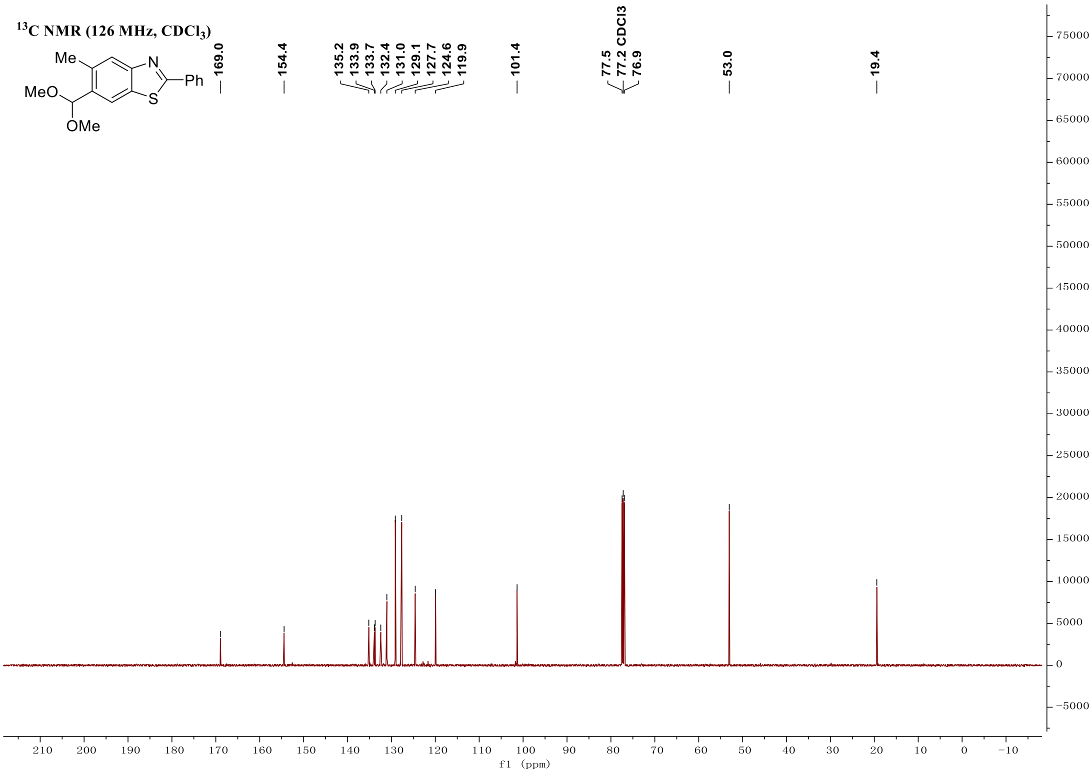

Supplementary Figure 160. ^{1}H NMR and ^{13}C NMR spectra of compound 38.

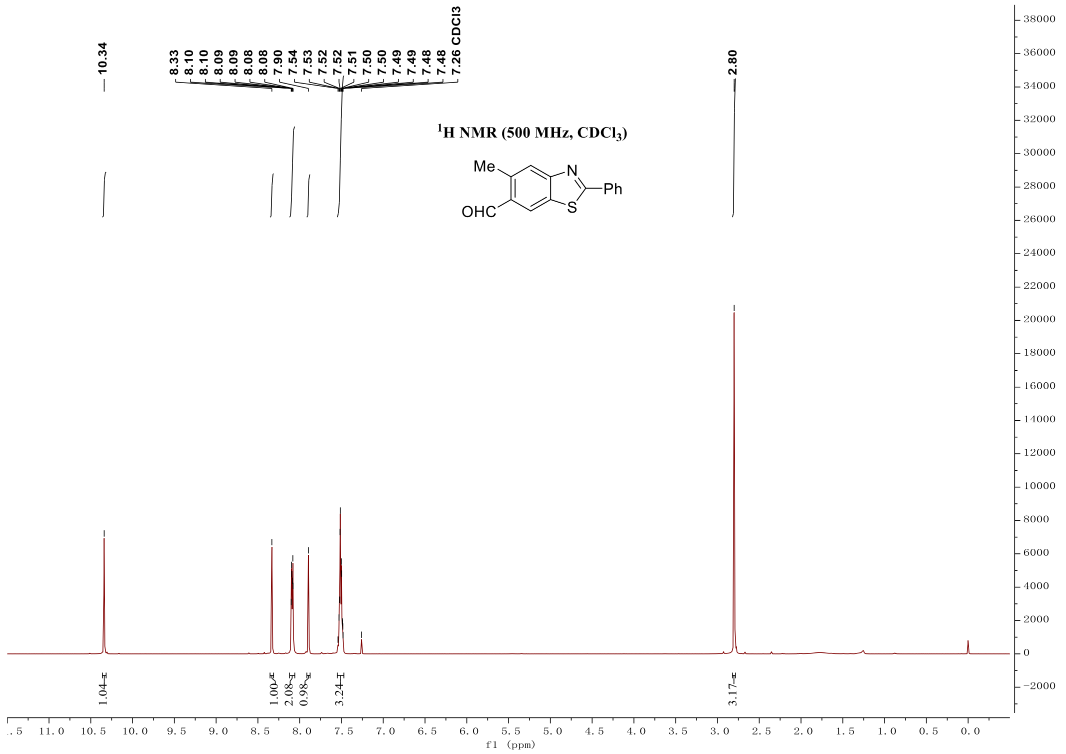

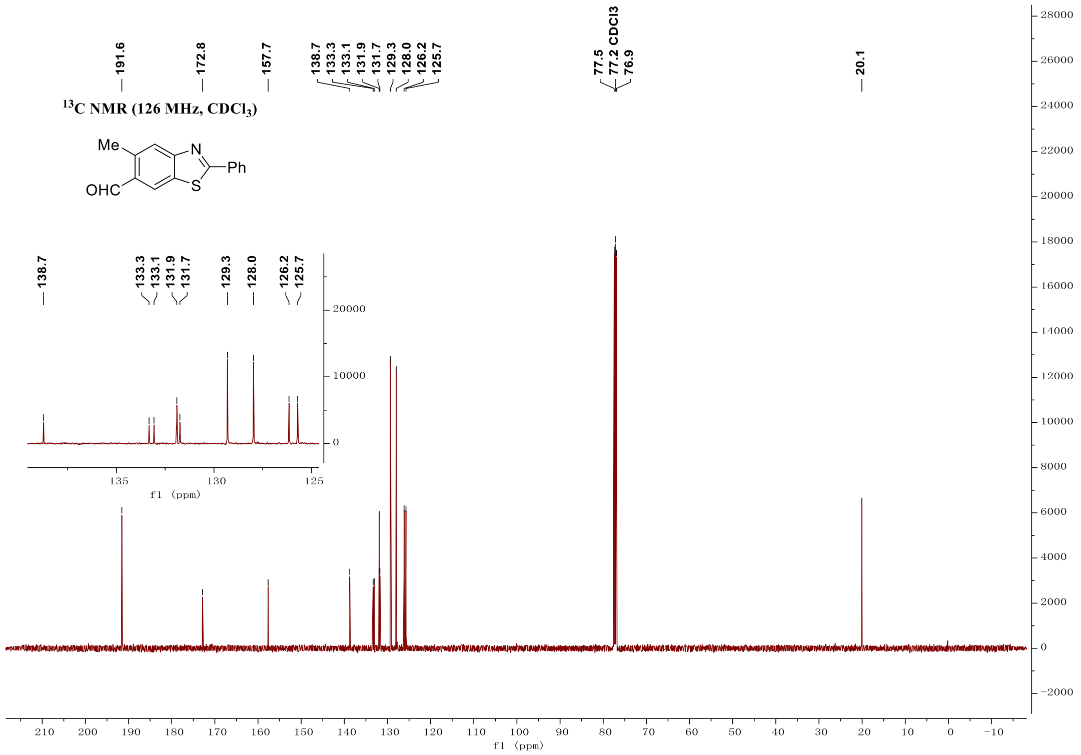

Supplementary Figure 161. ^{1}H NMR and ^{13}C NMR spectra of compound 39.

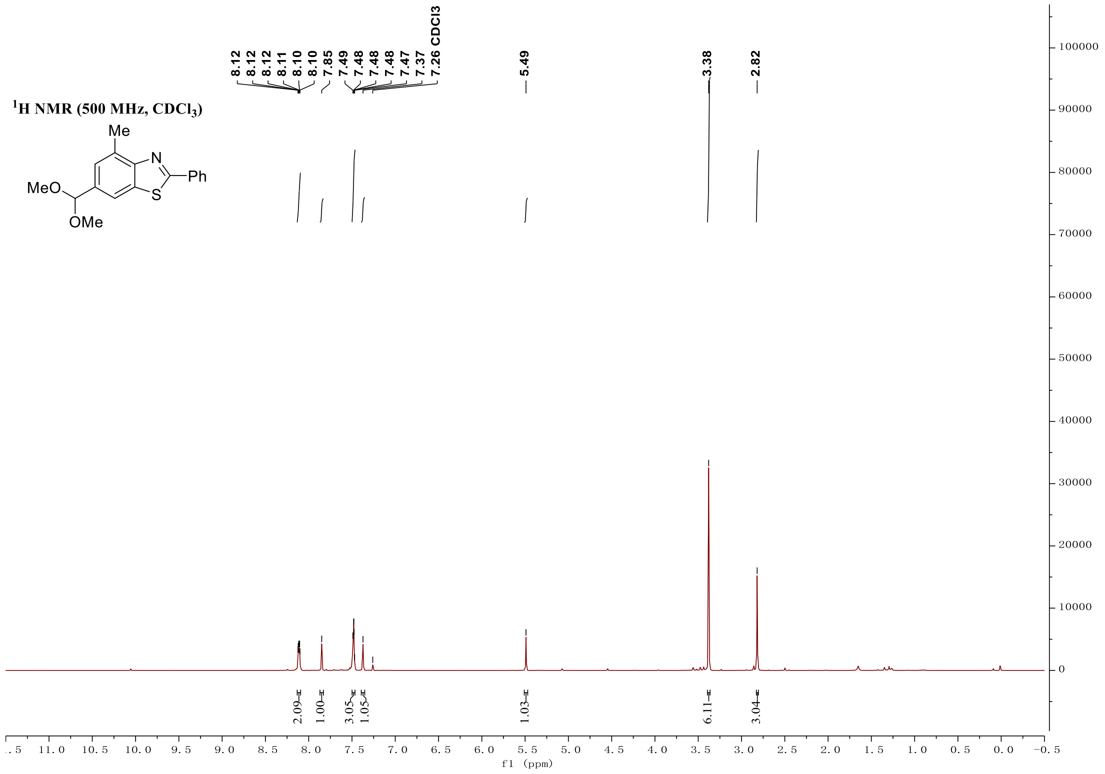

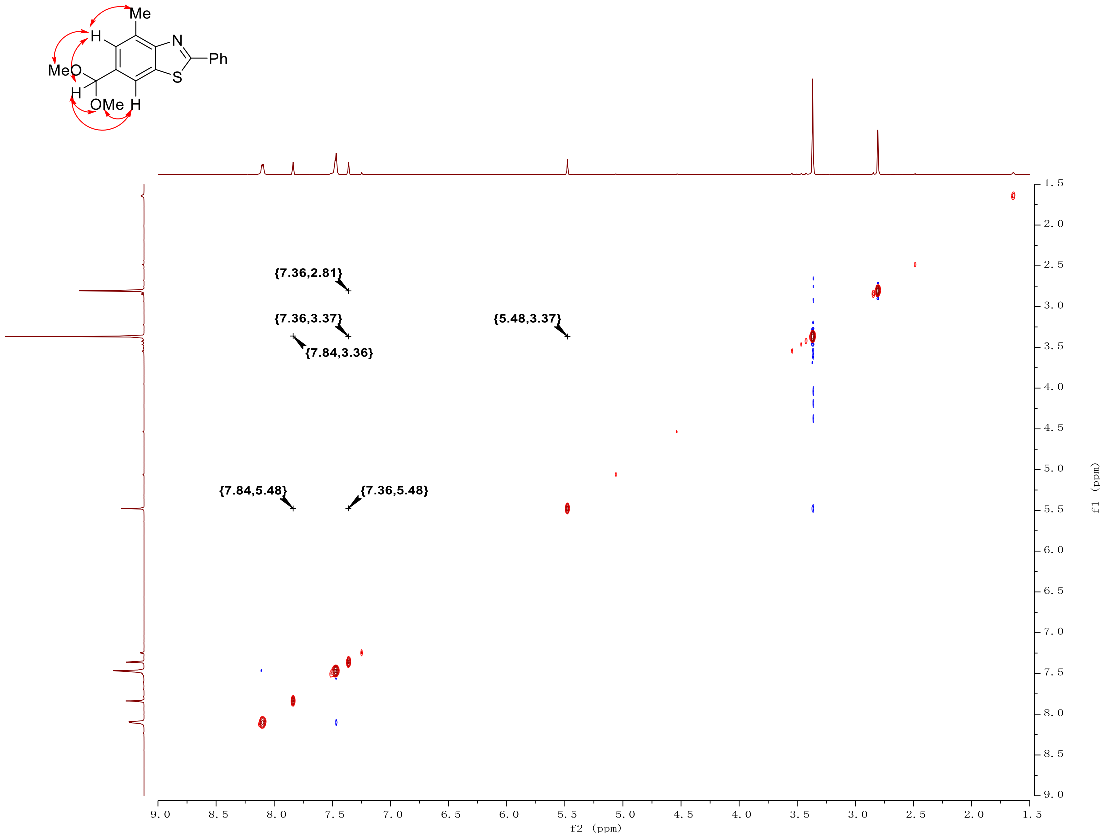

Supplementary Figure 162. ^{1}H NMR and 2D NOESY spectra of compound 40.

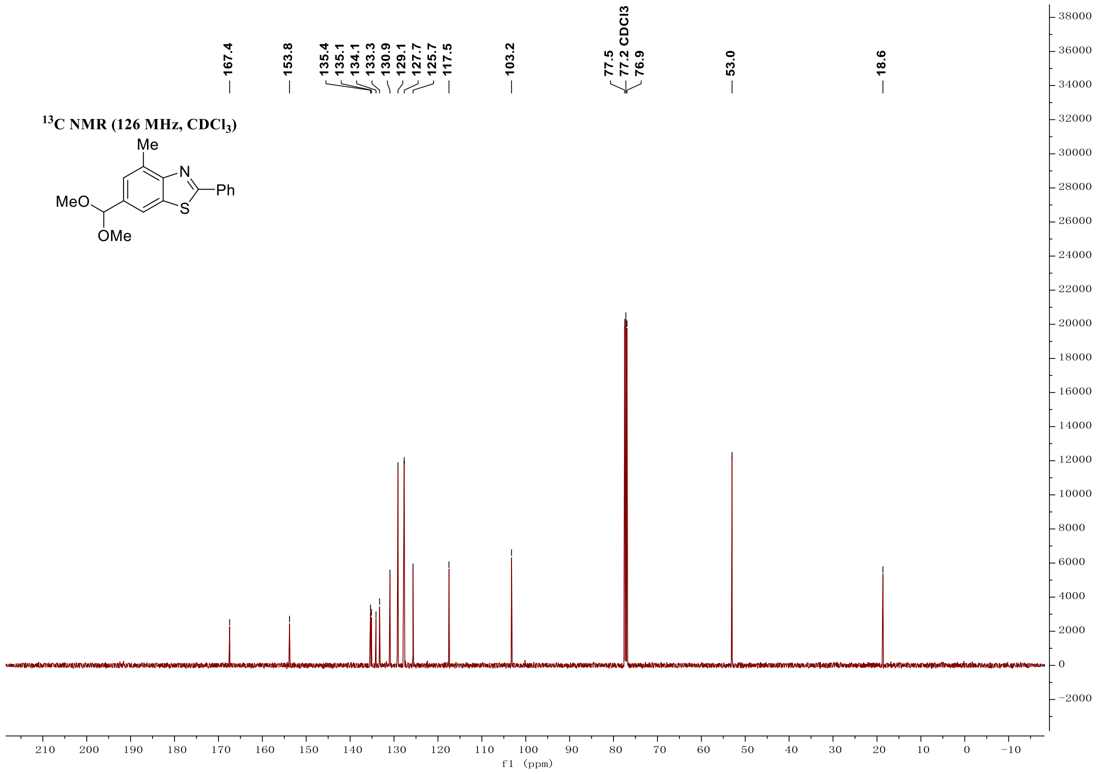

Supplementary Figure 163. ^{13}C NMR spectrum of compound 40.

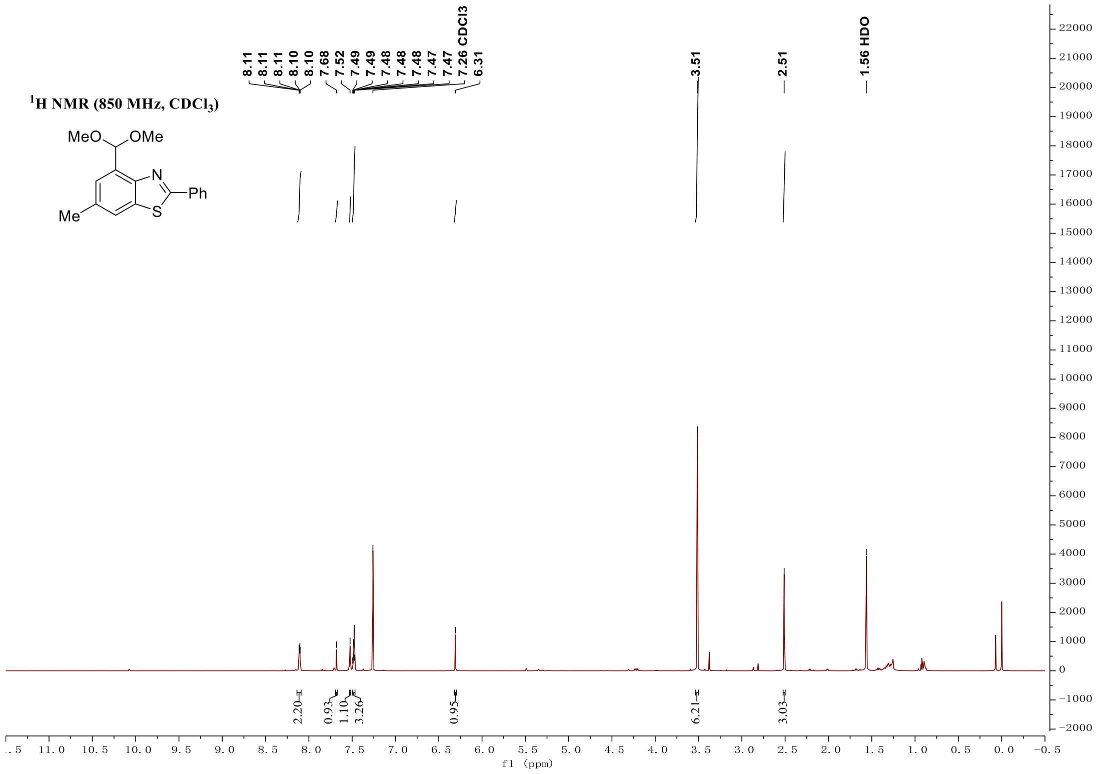

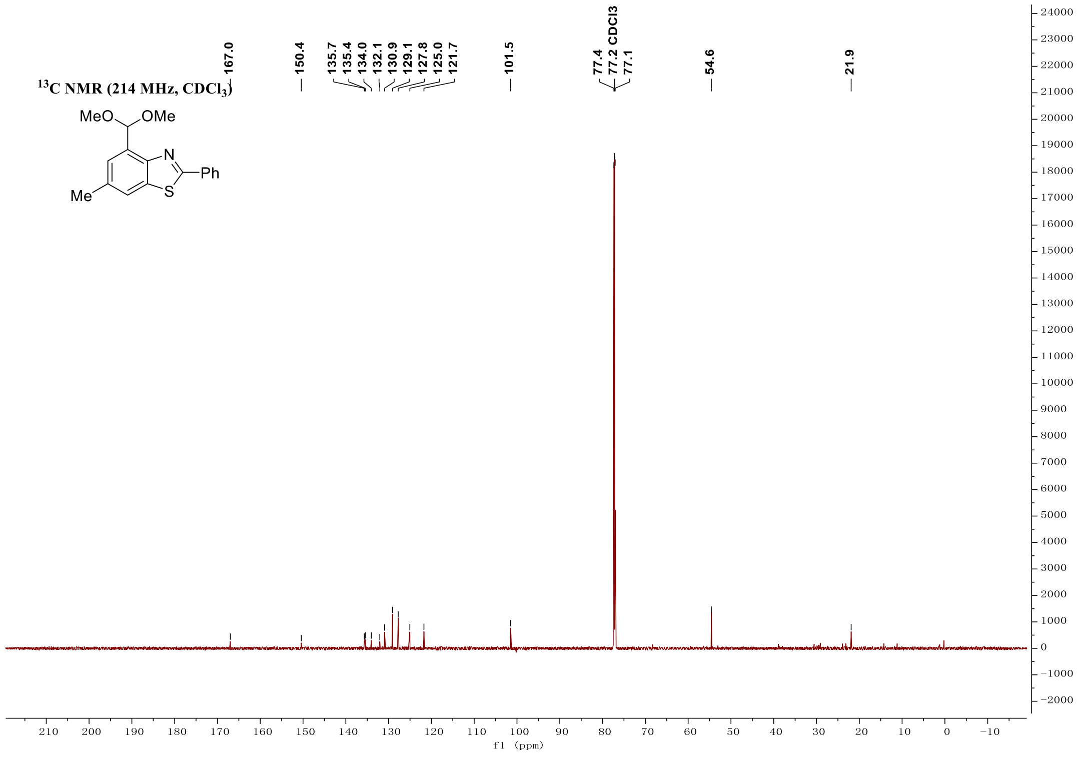

Supplementary Figure 164. ^{1}H NMR and ^{13}C NMR spectra of compound 40'.

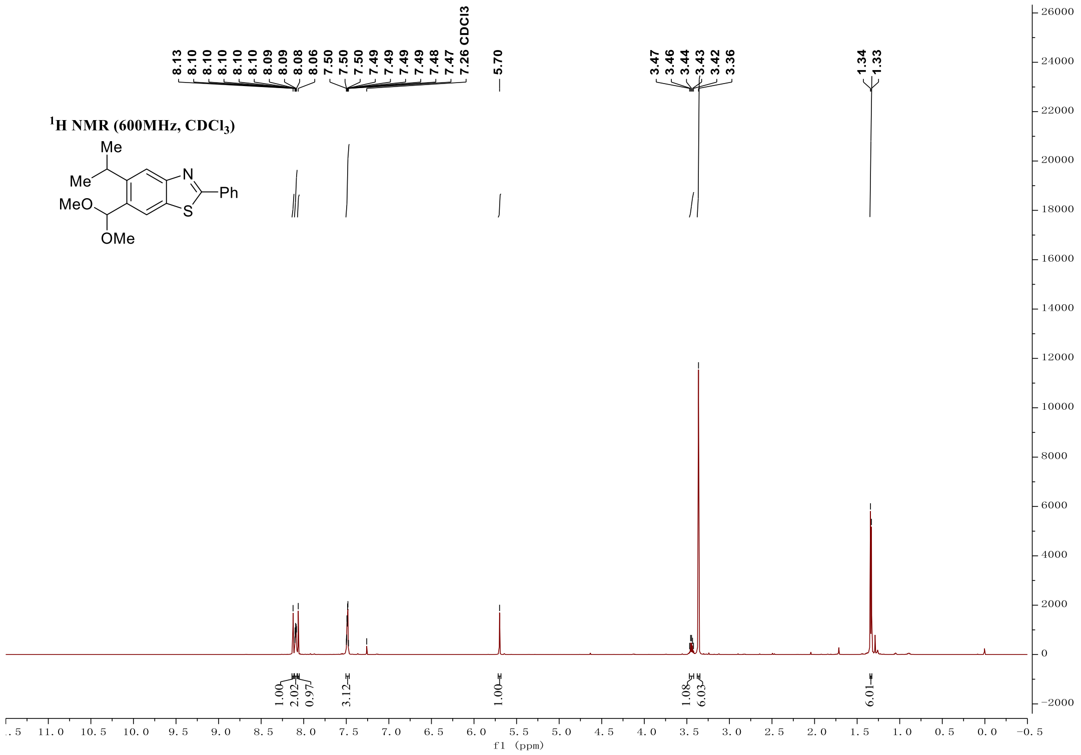

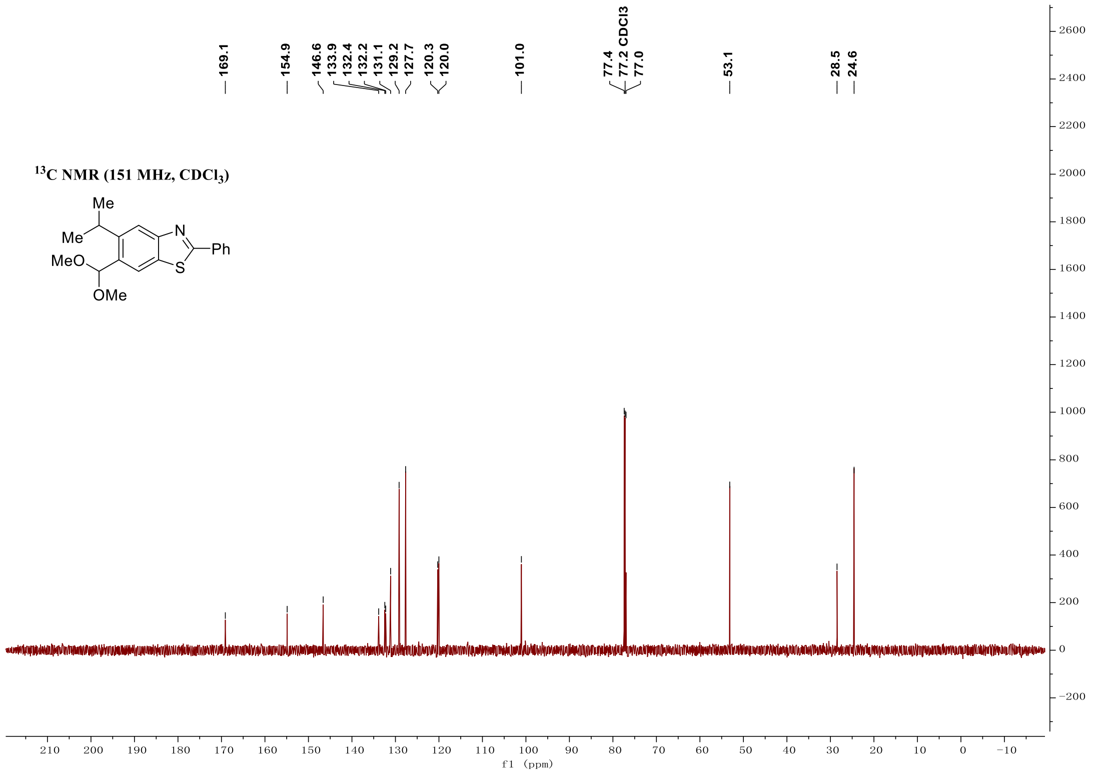

Supplementary Figure 165. ^{1}H NMR and ^{13}C NMR spectra of compound 41.

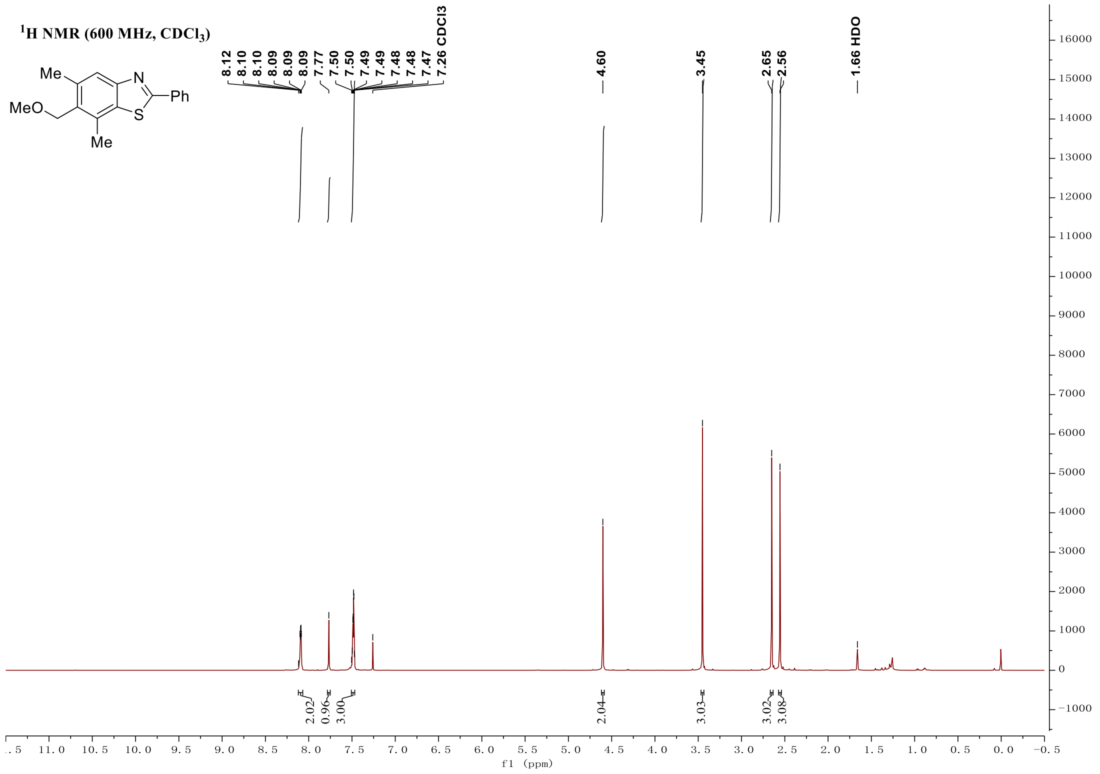

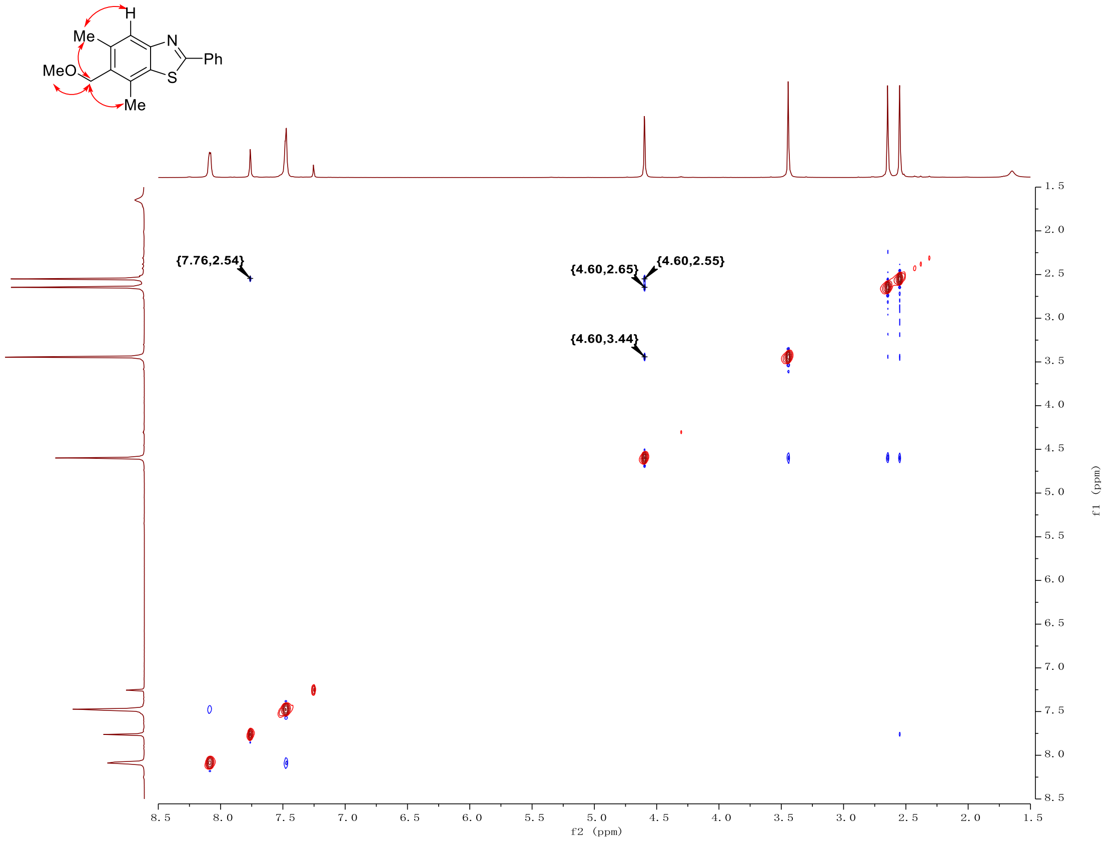

Supplementary Figure 166. ^{1}H NMR and 2D NOESY spectra of compound 42.

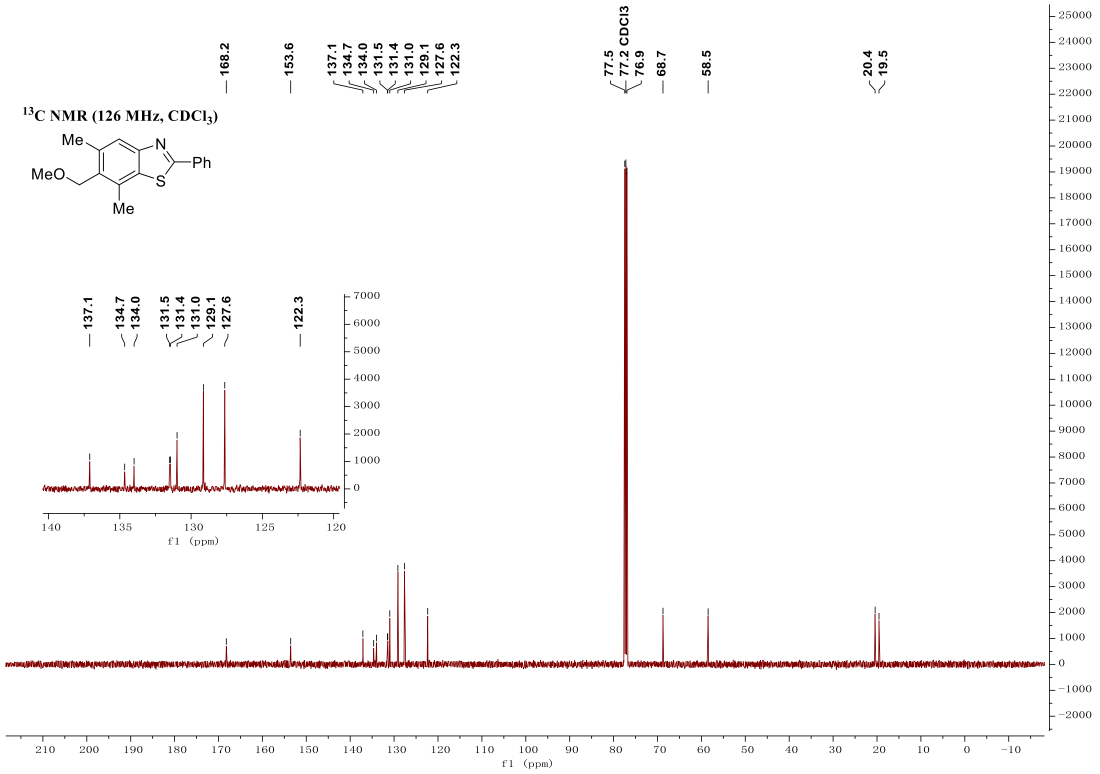

Supplementary Figure 167. ^{13}C NMR spectrum of compound 42.

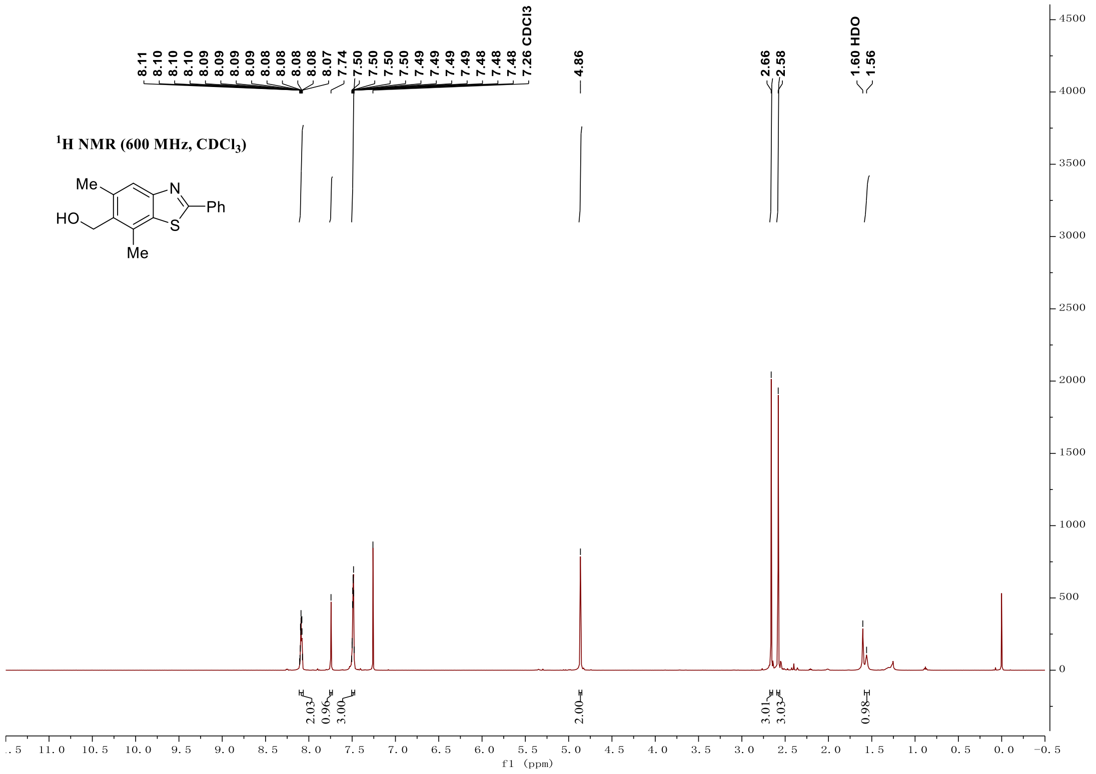

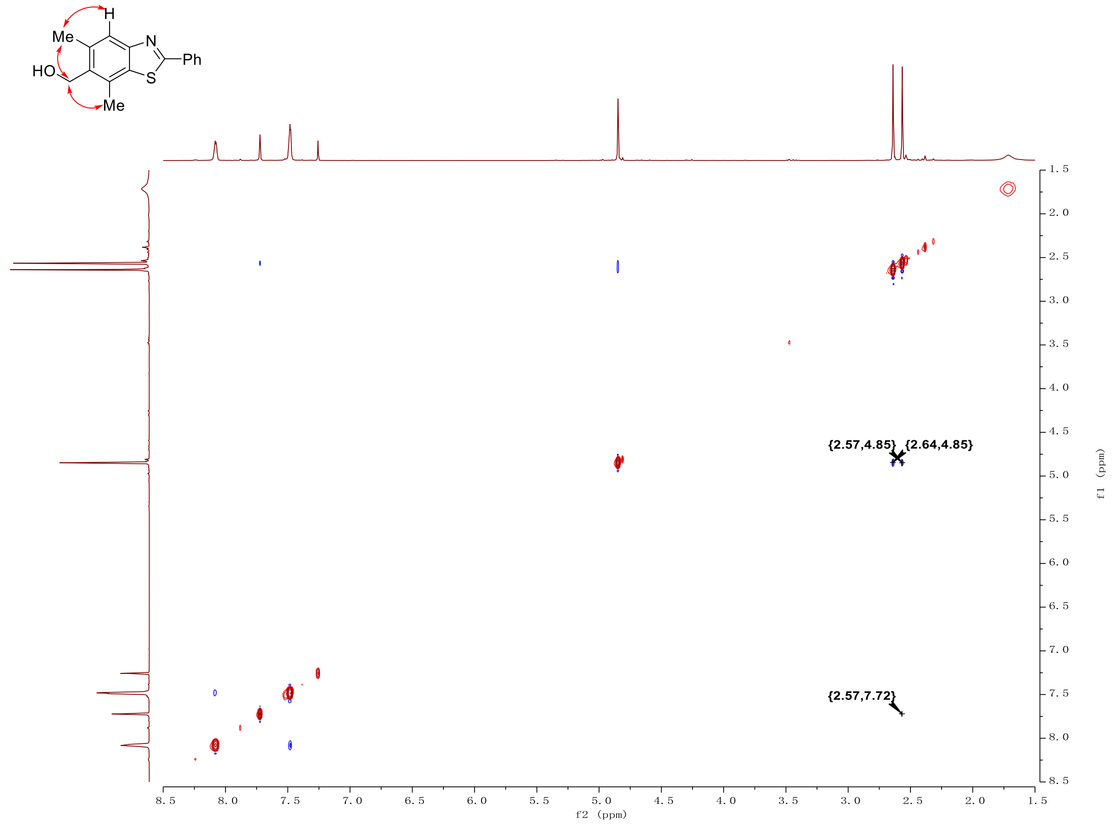

Supplementary Figure 168. ^{1}H NMR and 2D NOESY spectra of compound 43a.

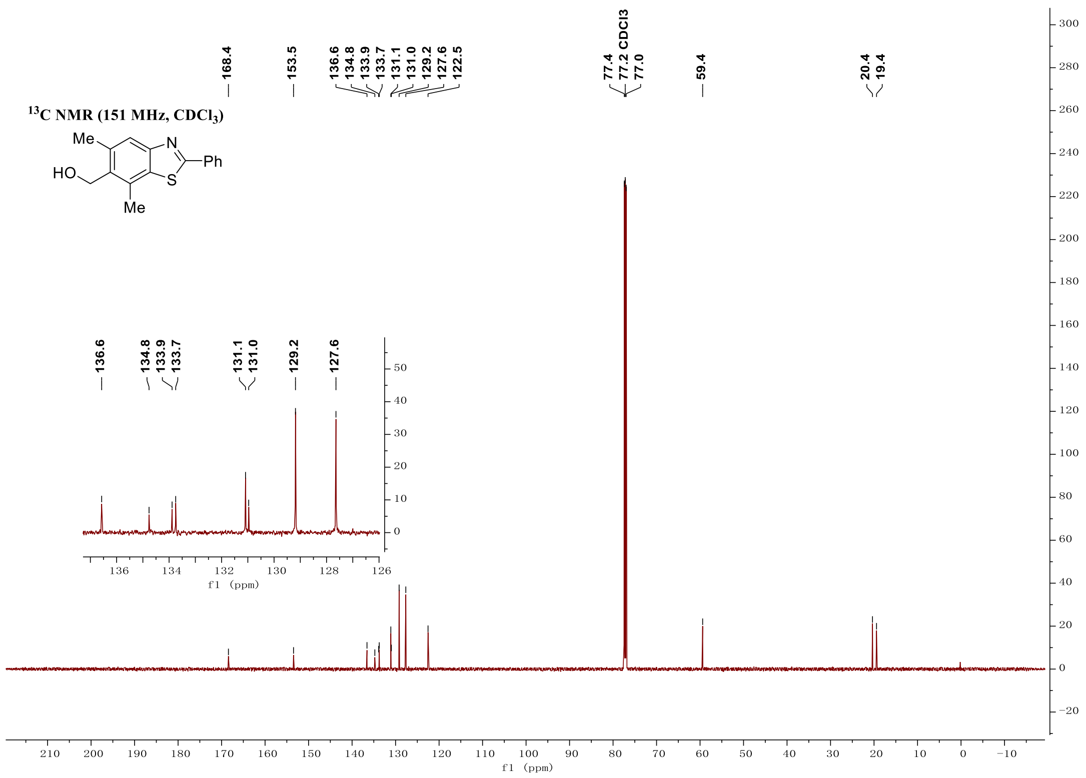

Supplementary Figure 169. ^{13}C **NMR spectrum of compound 43a.**

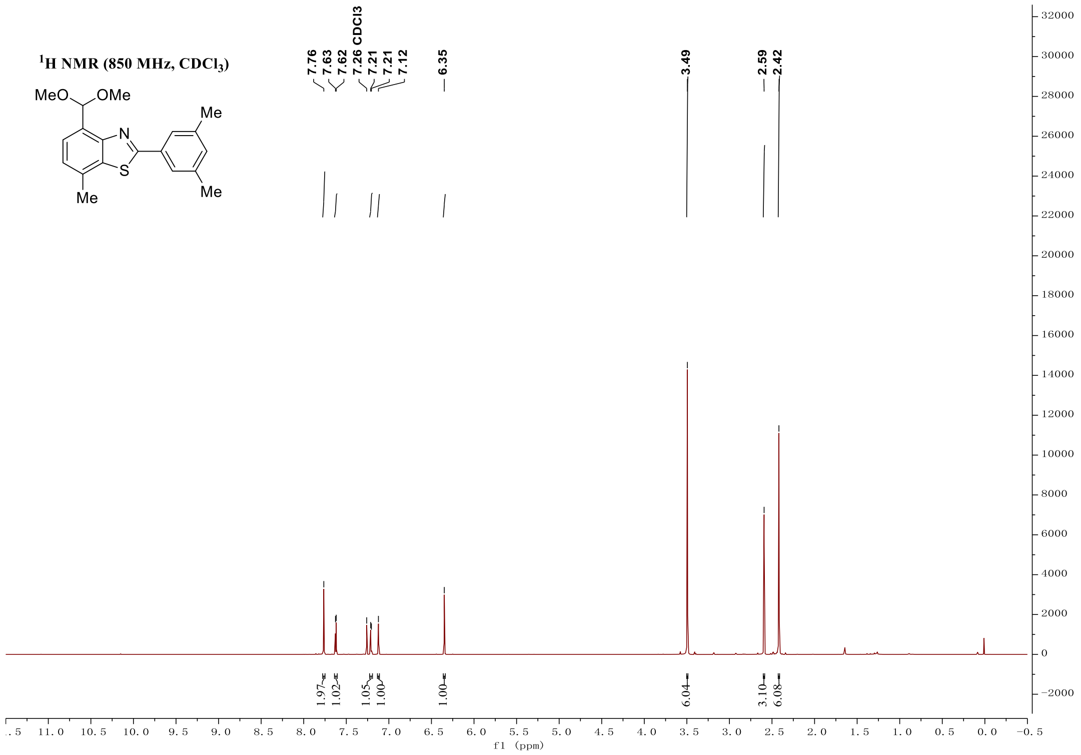

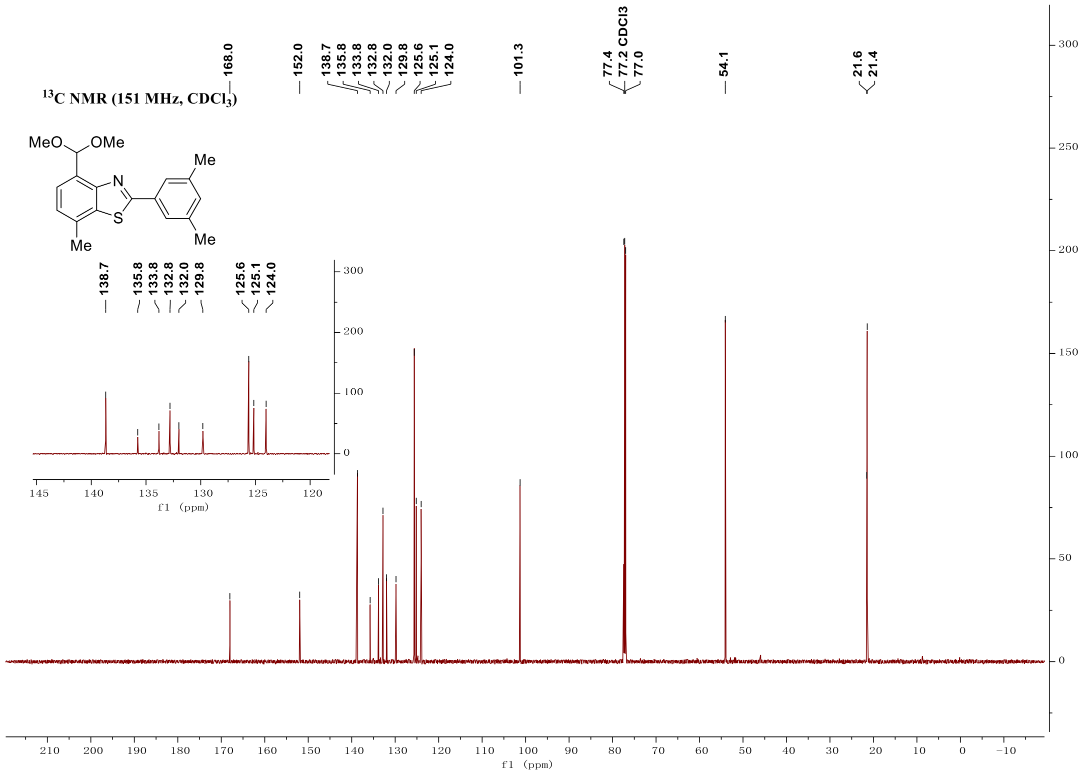

Supplementary Figure 170. ^{1}H NMR and ^{13}C NMR spectra of compound 44.

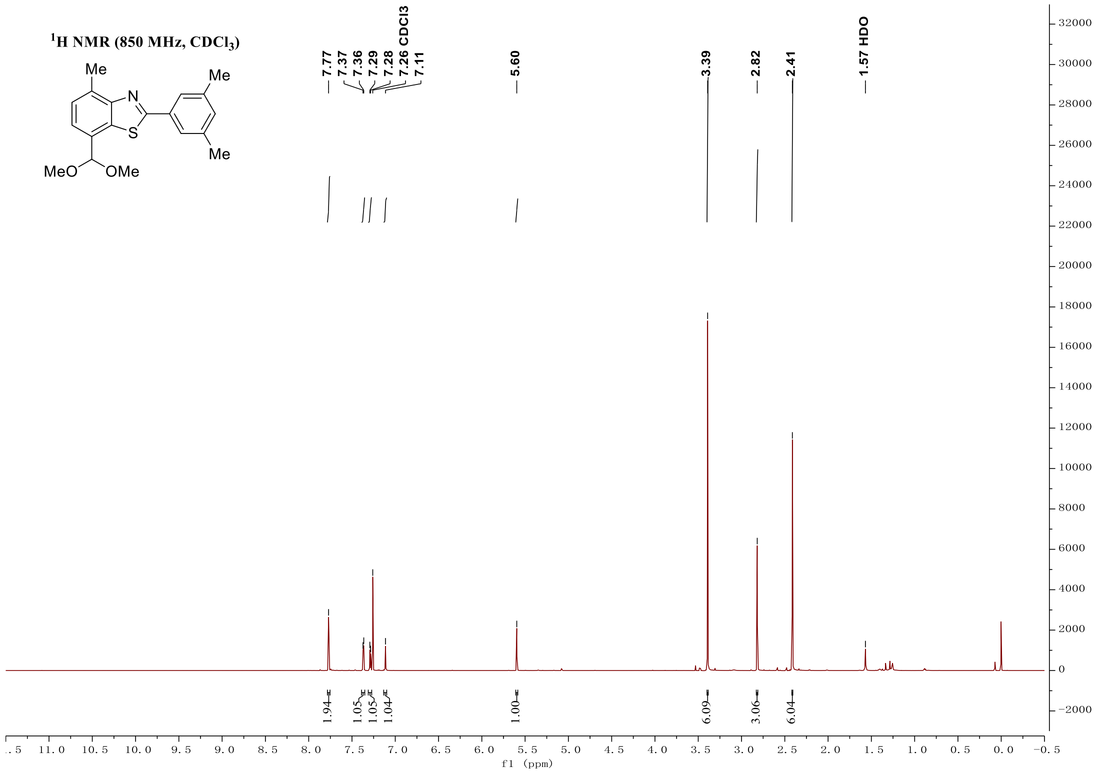

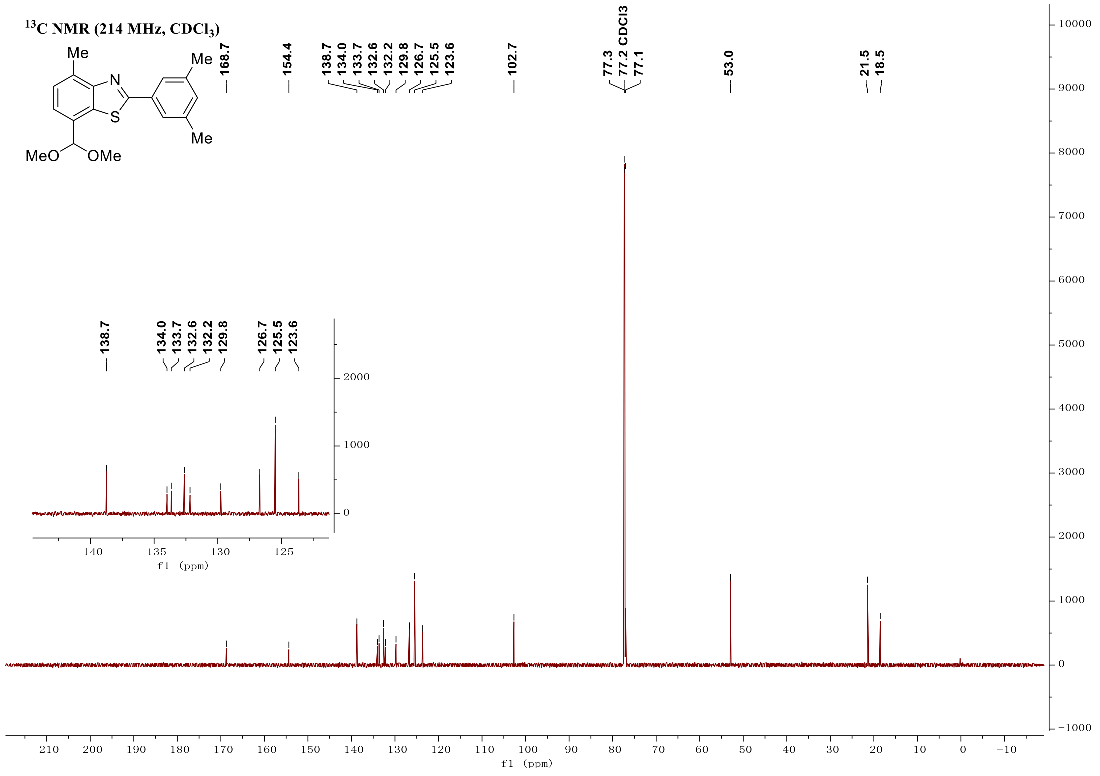

Supplementary Figure 171. ^{1}H NMR and ^{13}C NMR spectra of compound 44'.

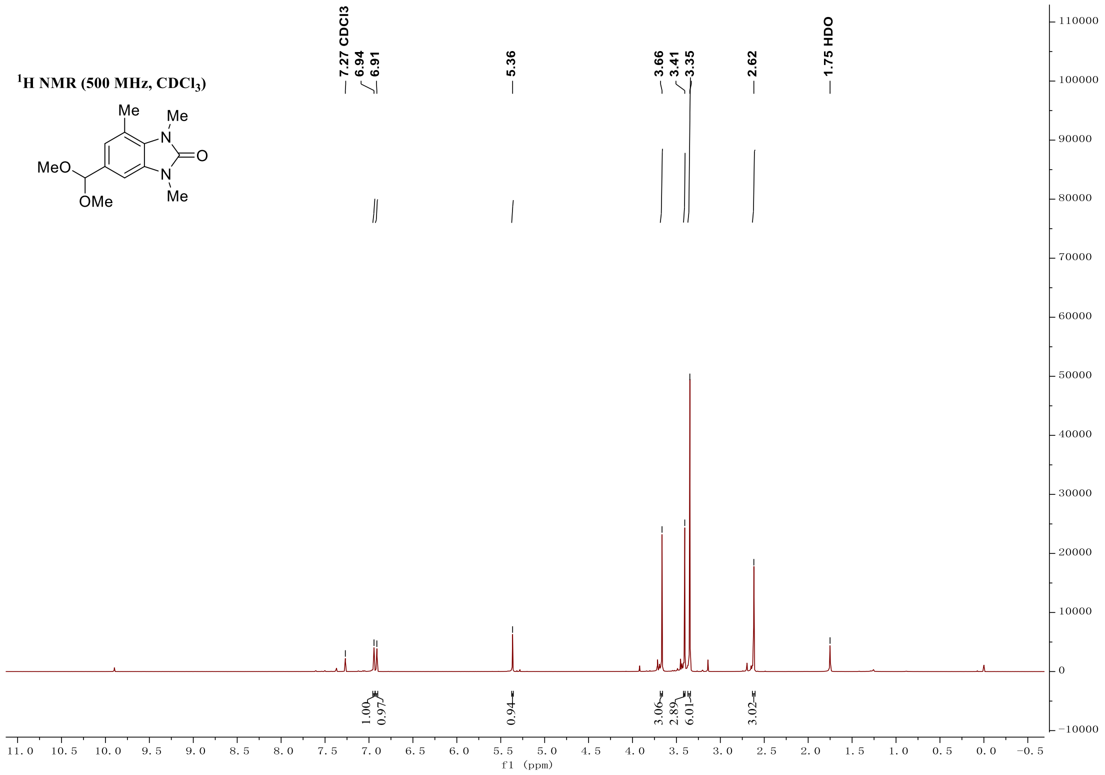

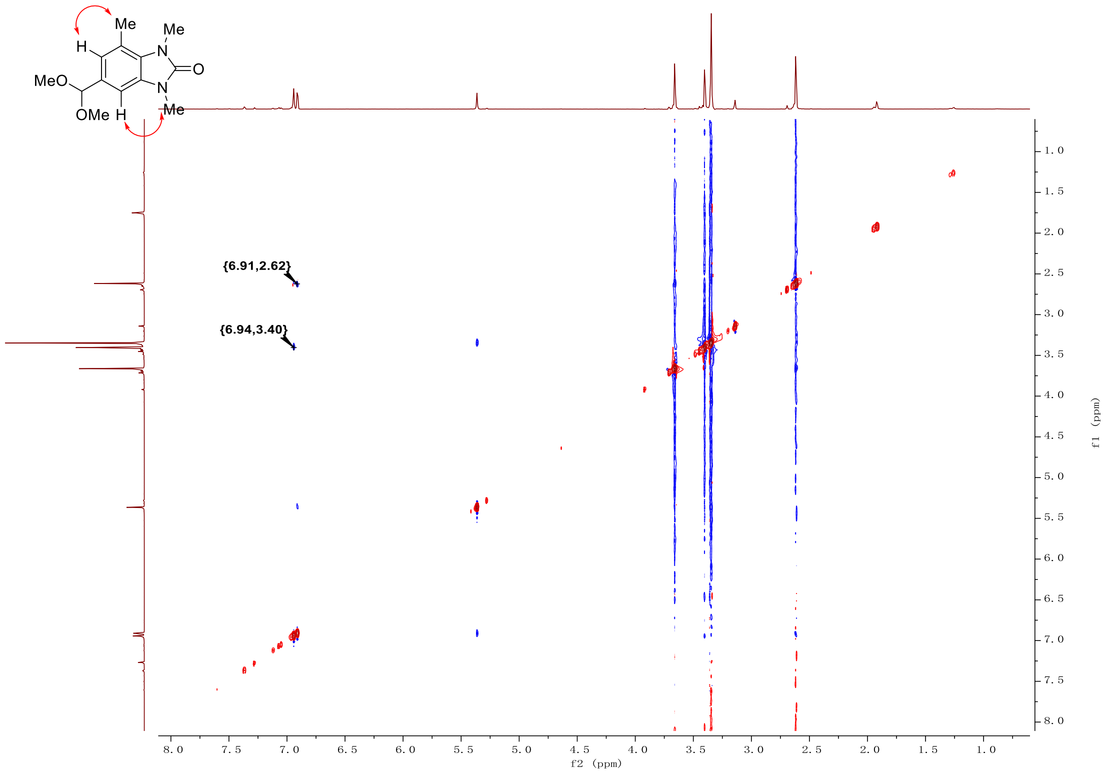

Supplementary Figure 172. ¹H NMR and 2D NOESY spectra of compound 45.

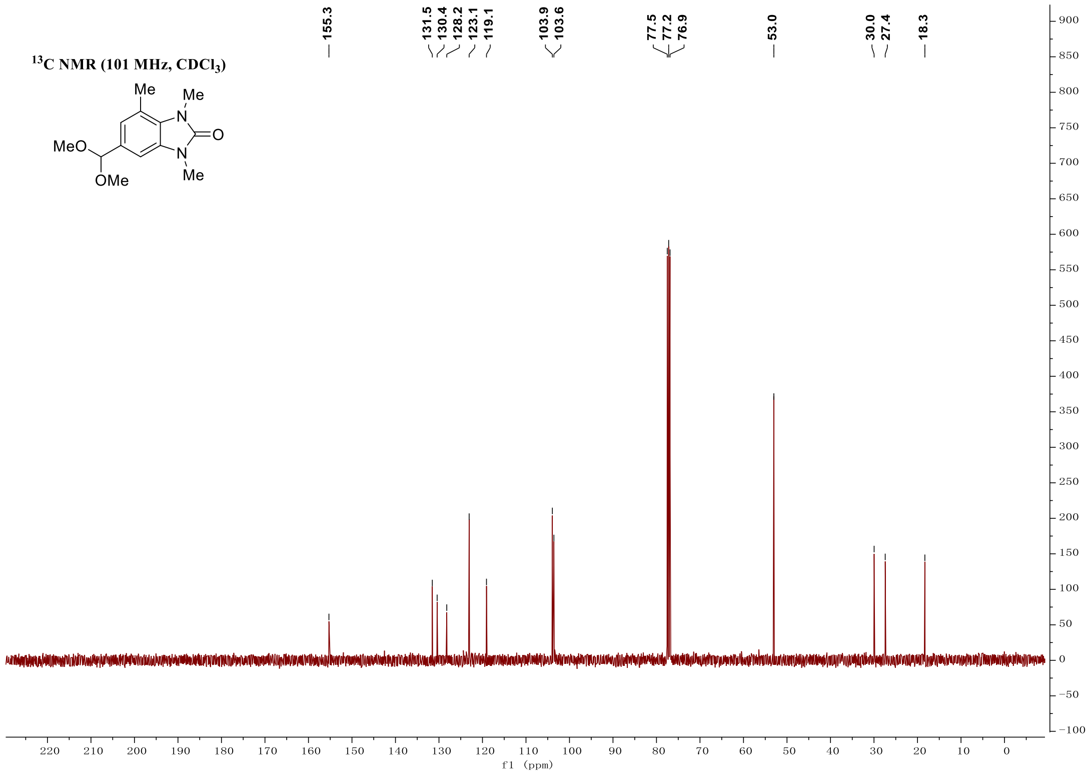

Supplementary Figure 173. ^{13}C NMR spectrum of compound 45.

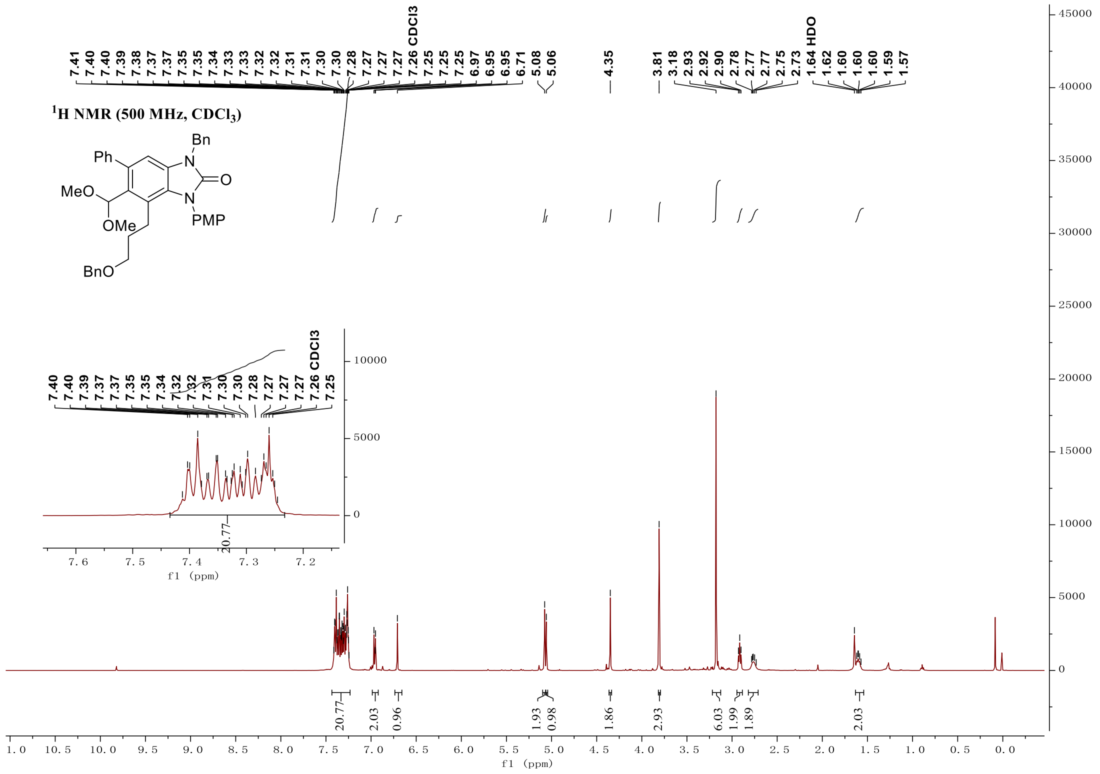

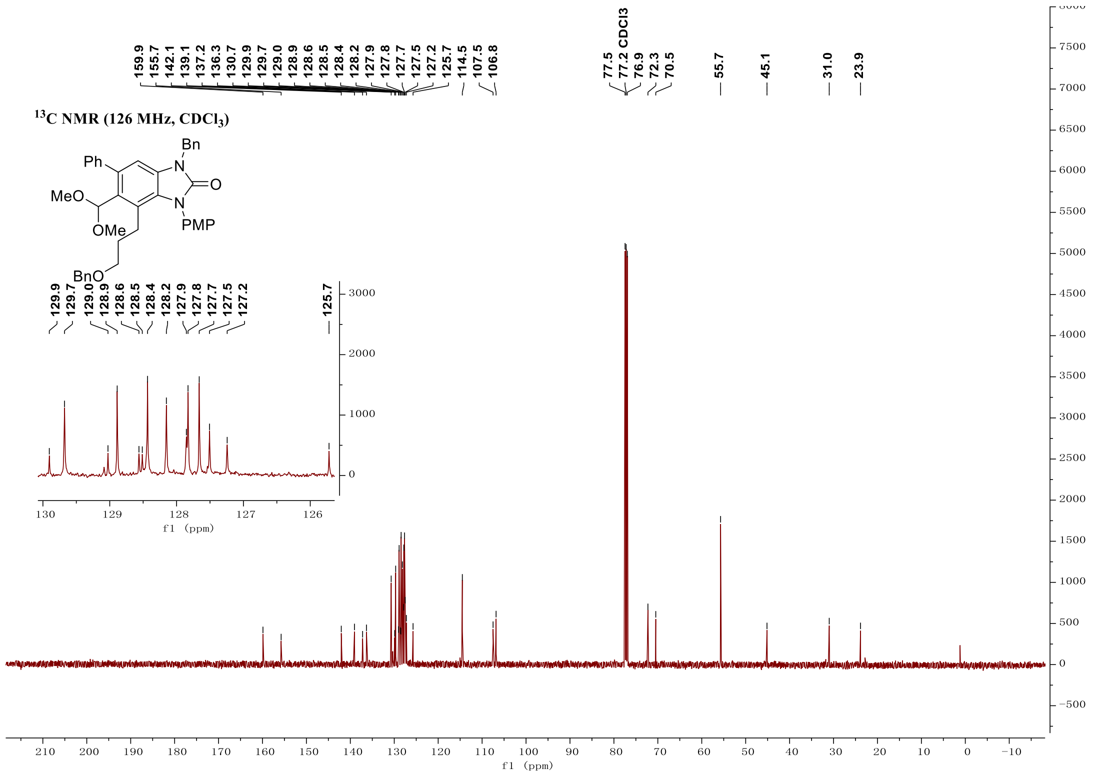

Supplementary Figure 174. ^{1}H NMR and ^{13}C NMR spectra of compound 46.

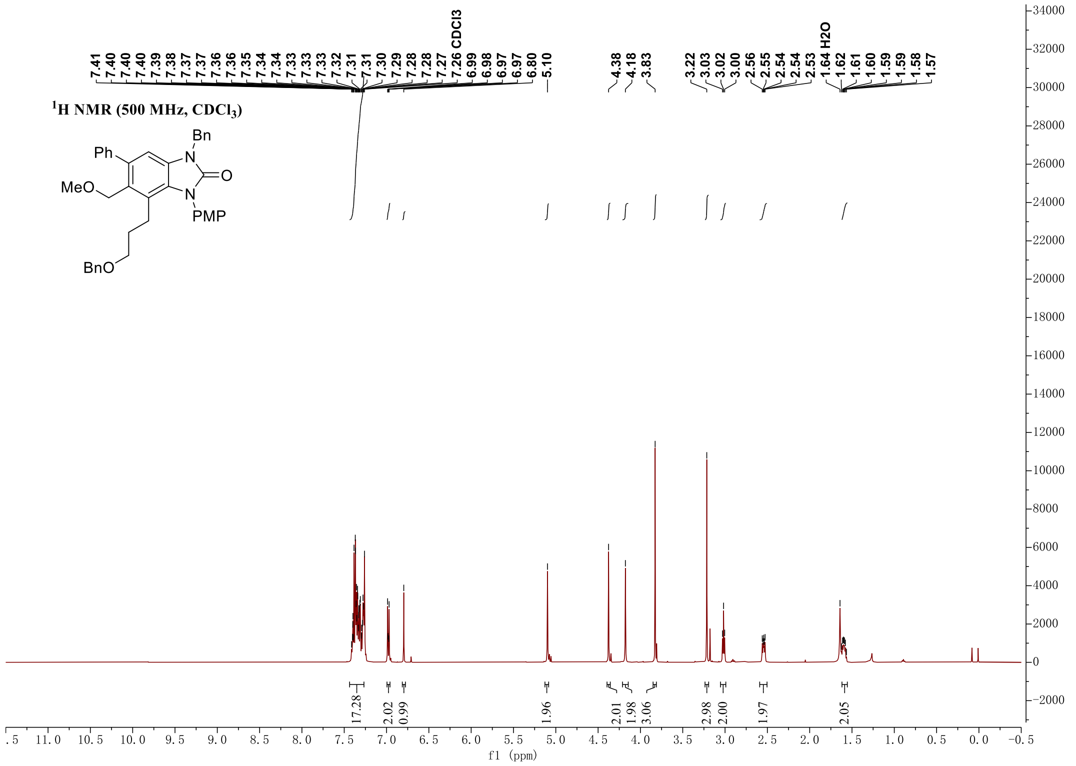

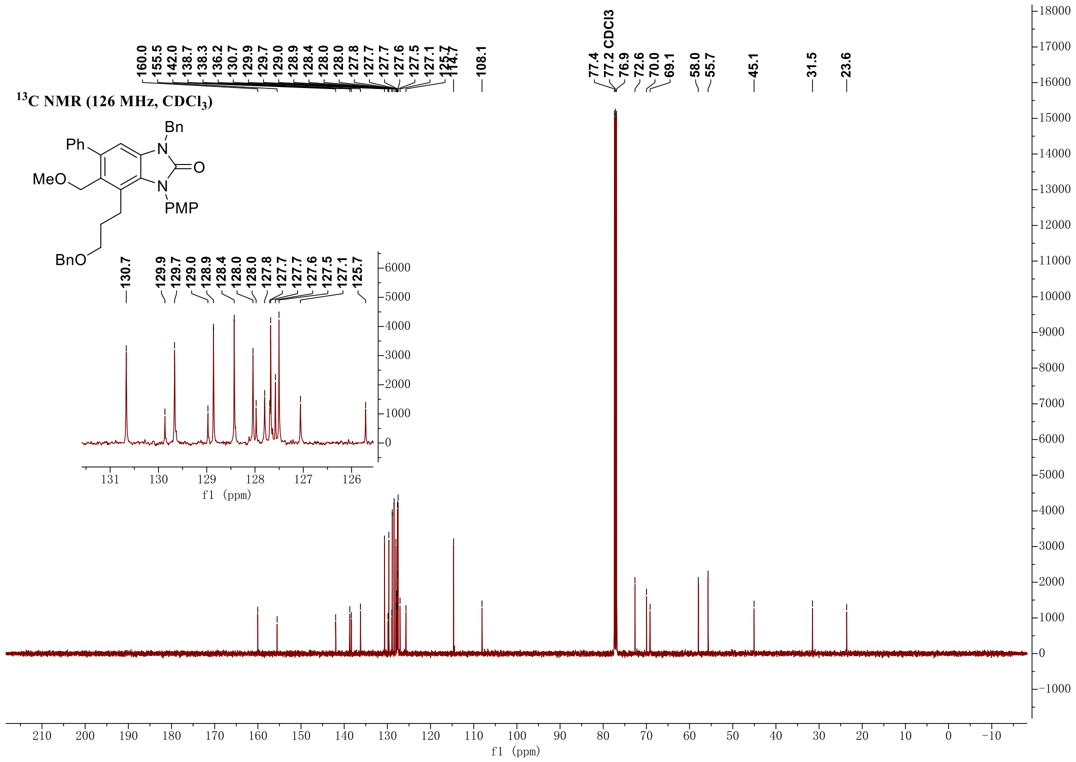

Supplementary Figure 175. ^{1}H NMR and ^{13}C NMR spectra of compound 46'.

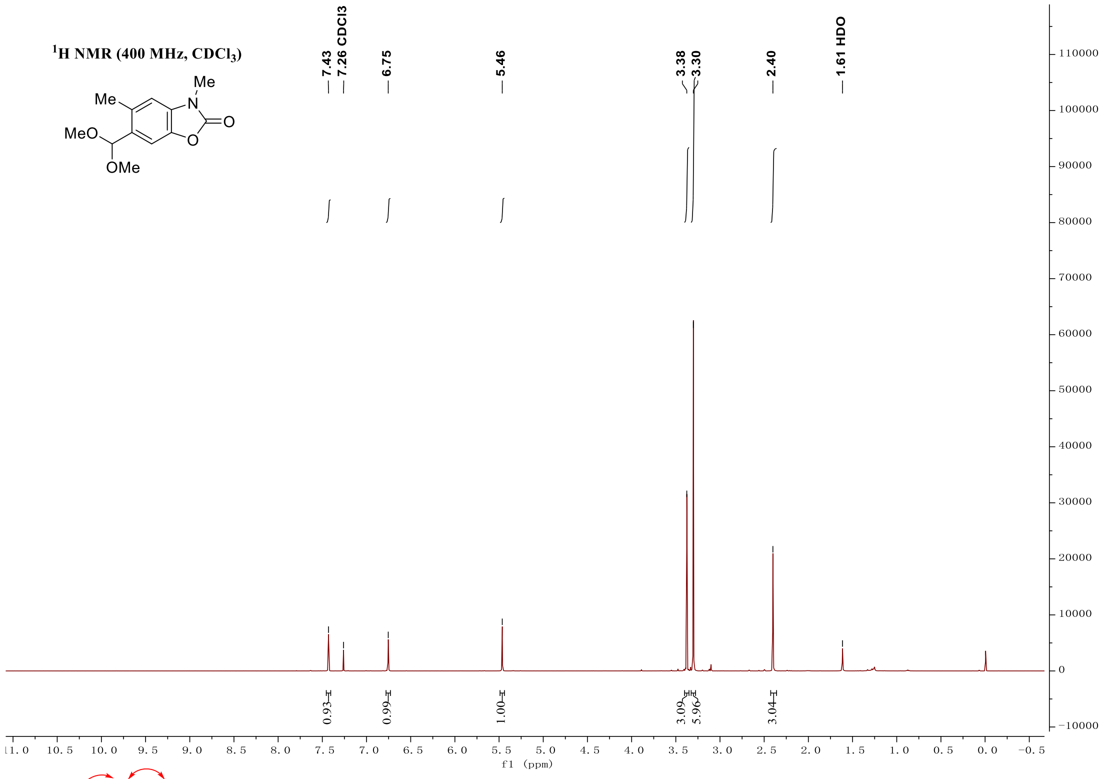

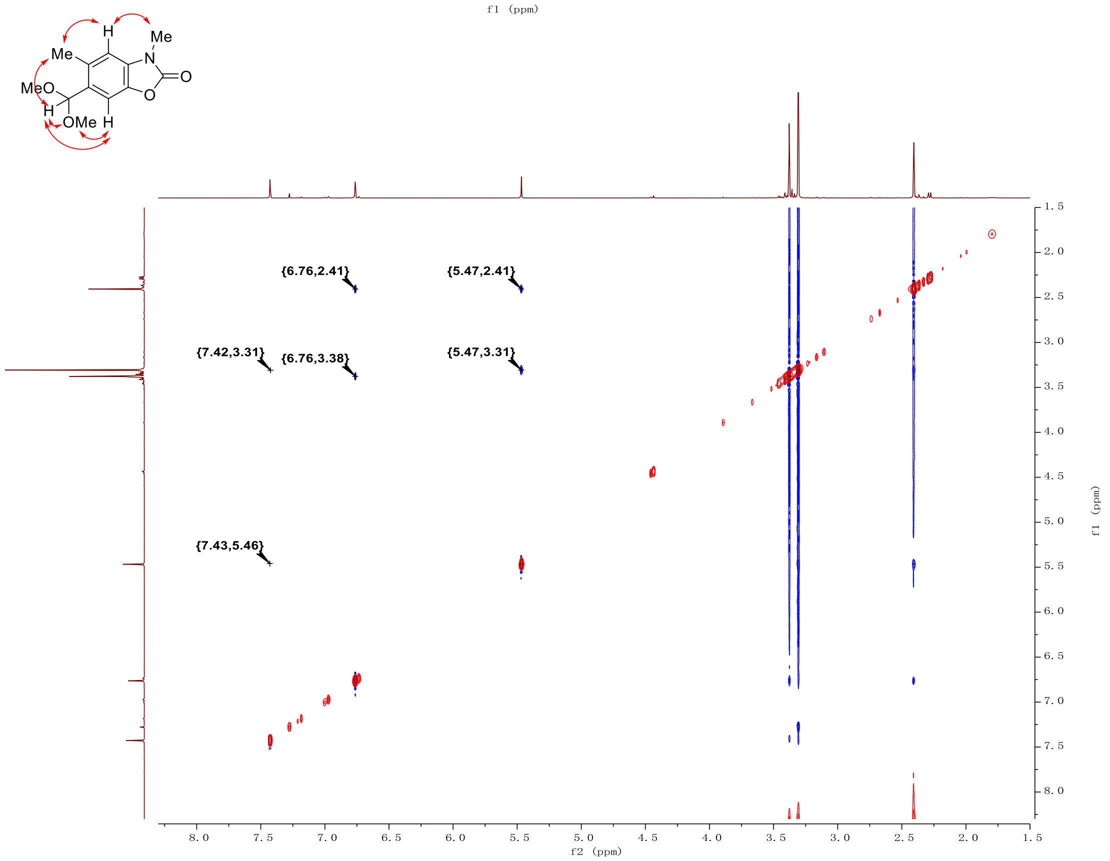

Supplementary Figure 176. ^{1}H NMR and 2D NOESY spectra of compound 47.

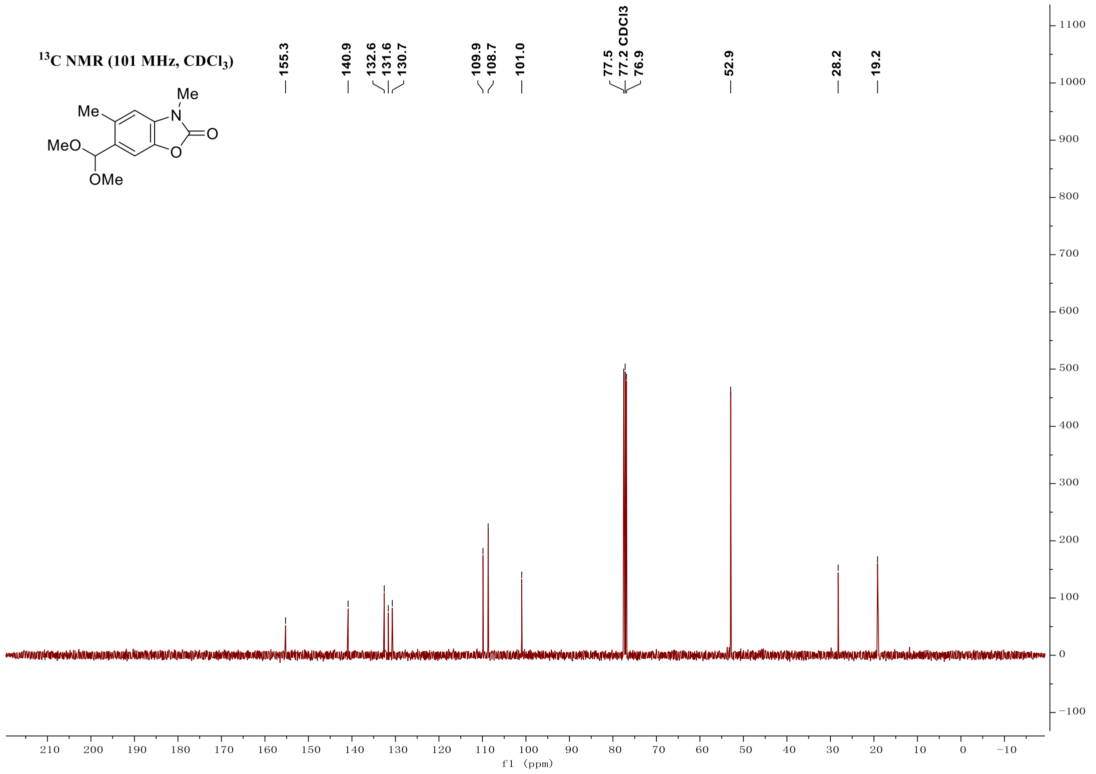

Supplementary Figure 177. ^{13}C NMR spectrum of compound 47.

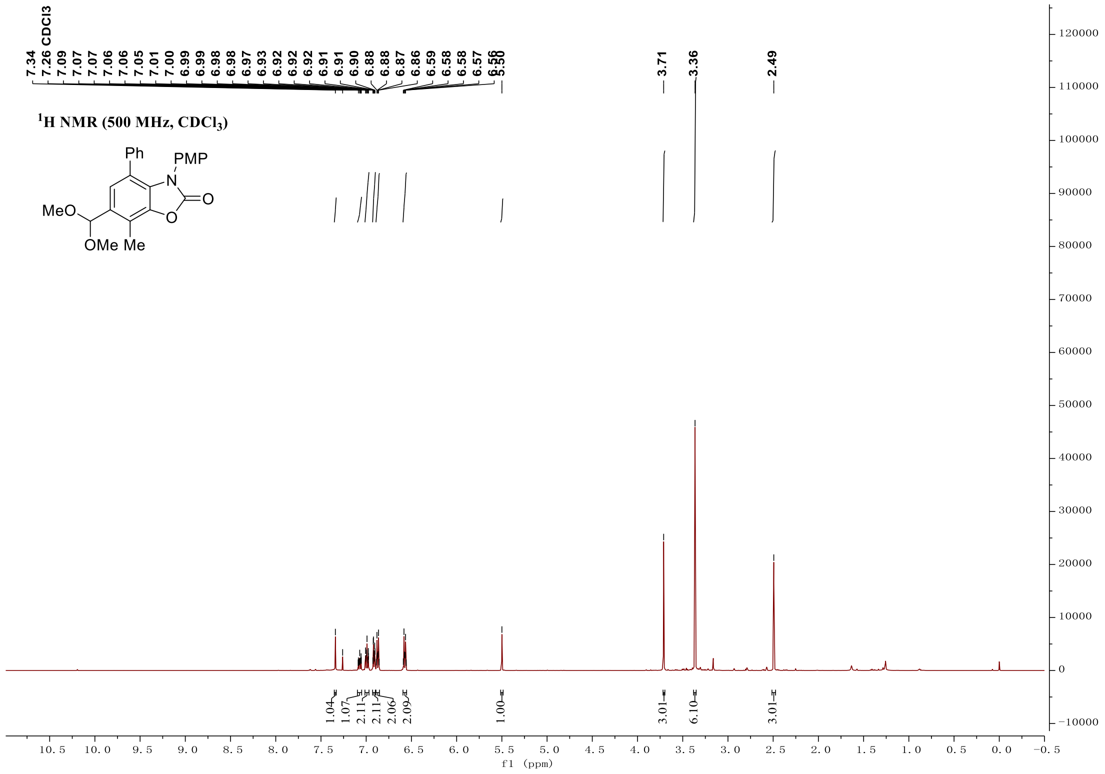

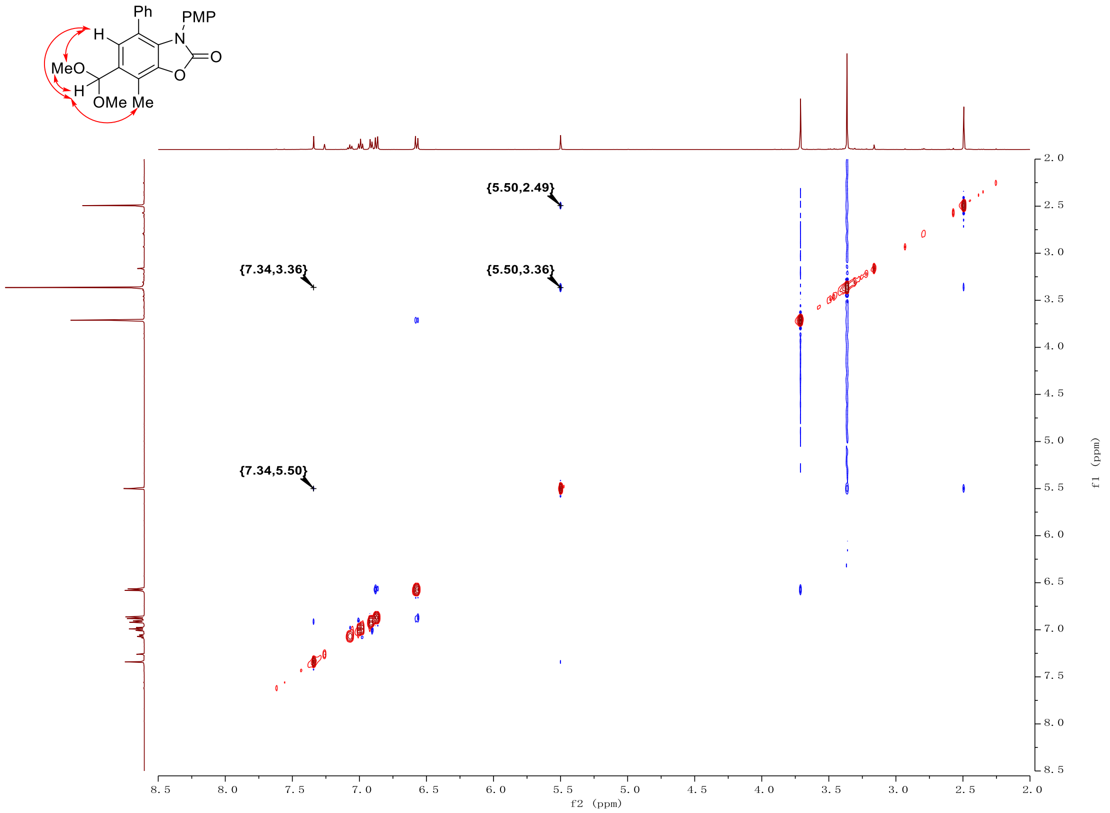

Supplementary Figure 178. ^{1}H NMR and 2D NOESY spectra of compound 48.

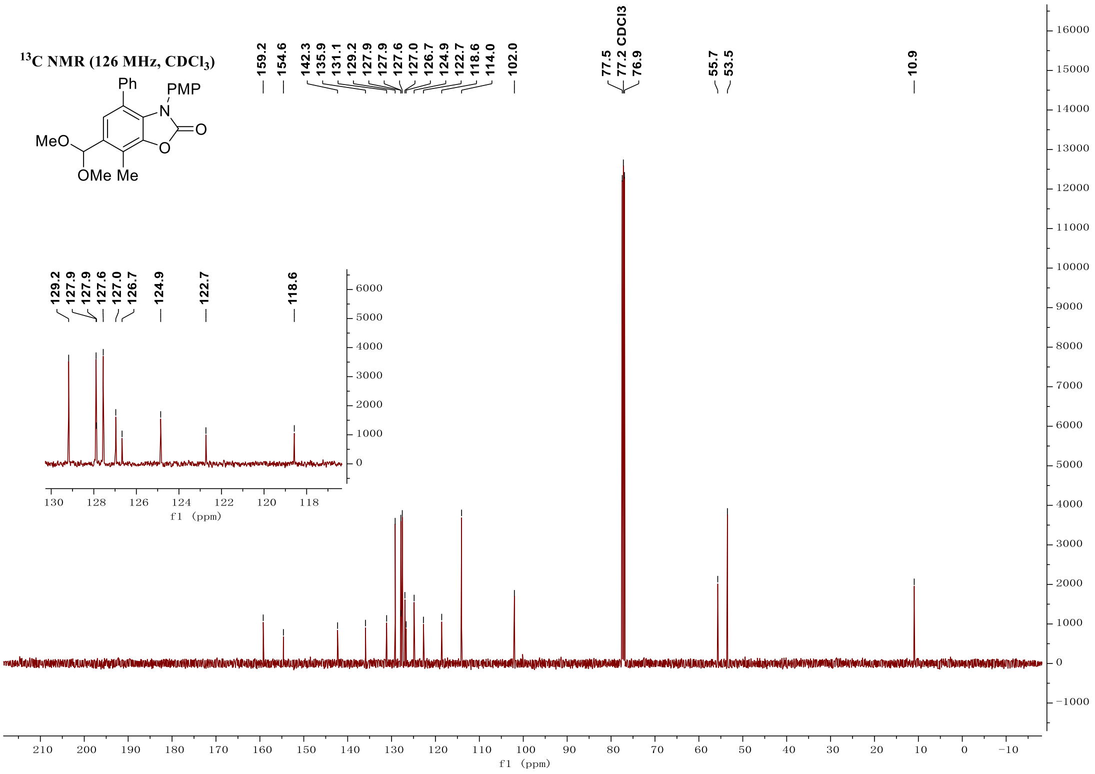

Supplementary Figure 179. ^{13}C NMR spectrum of compound 48.

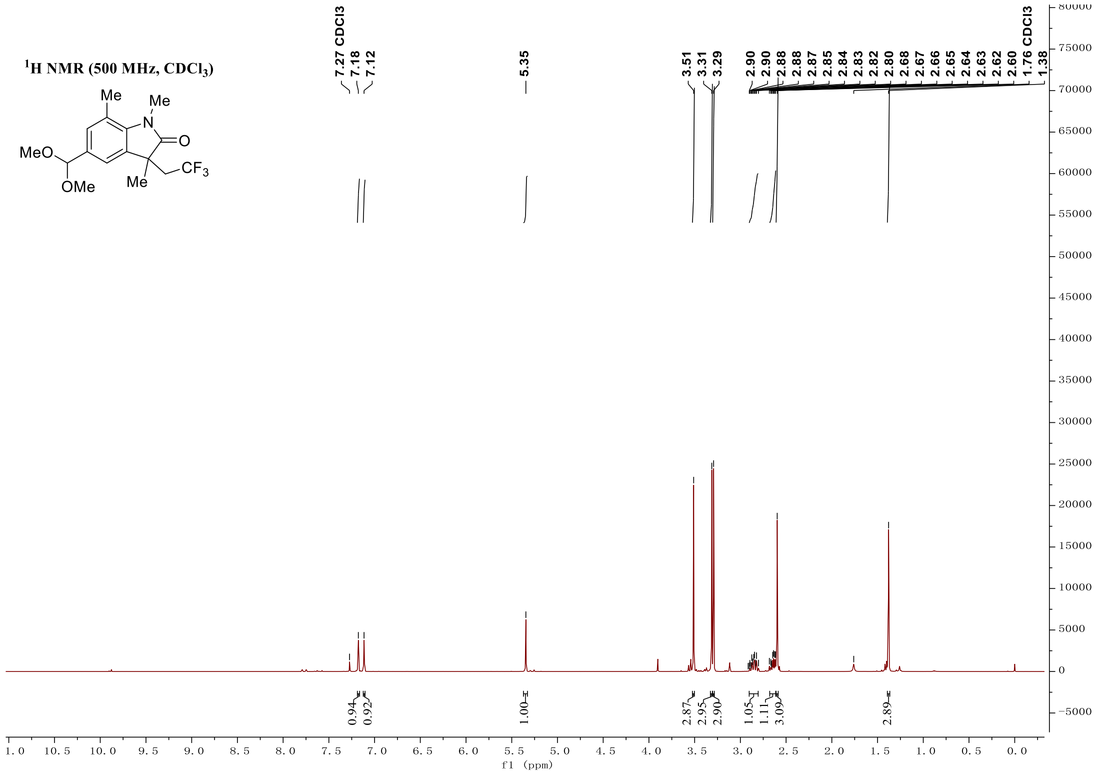

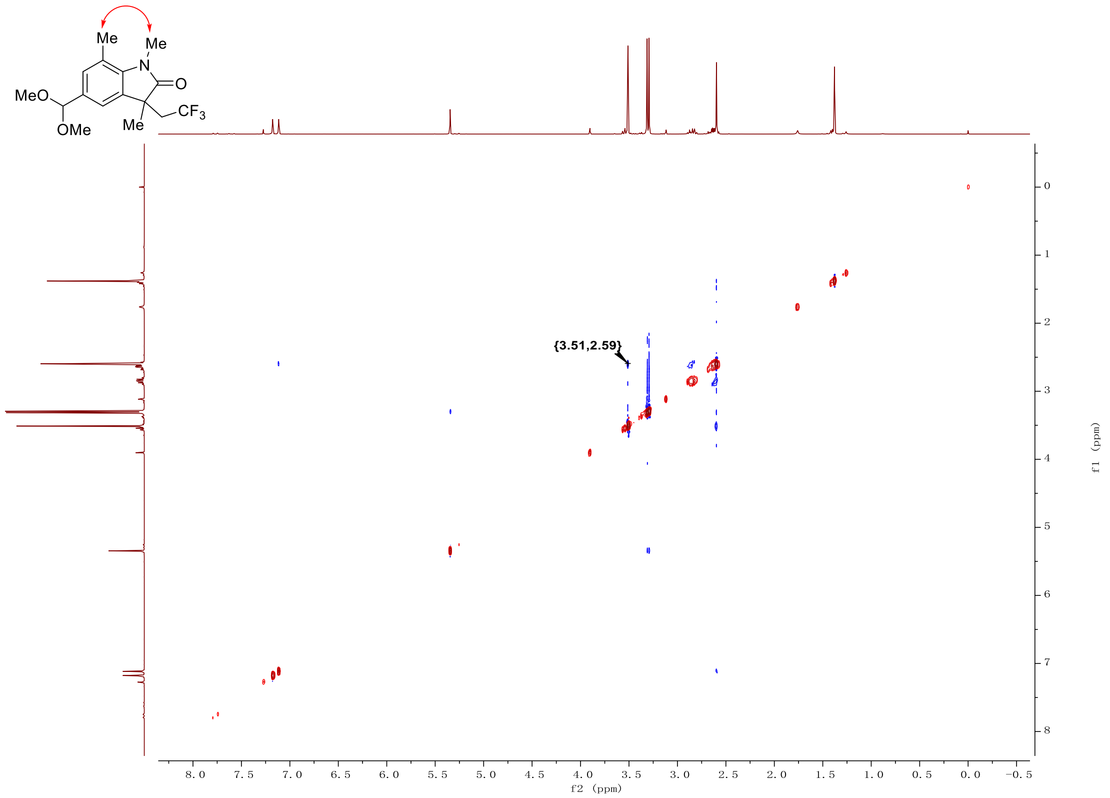

Supplementary Figure 180. ^{1}H NMR and 2D NOESY spectra of compound 49.

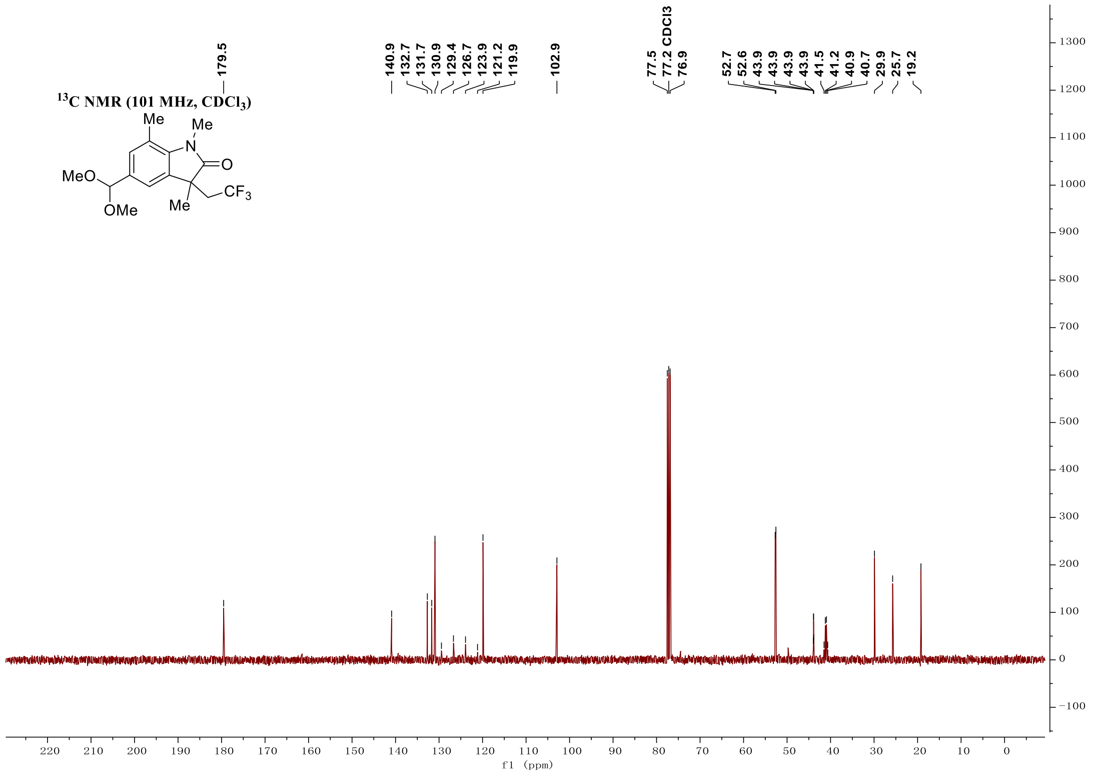

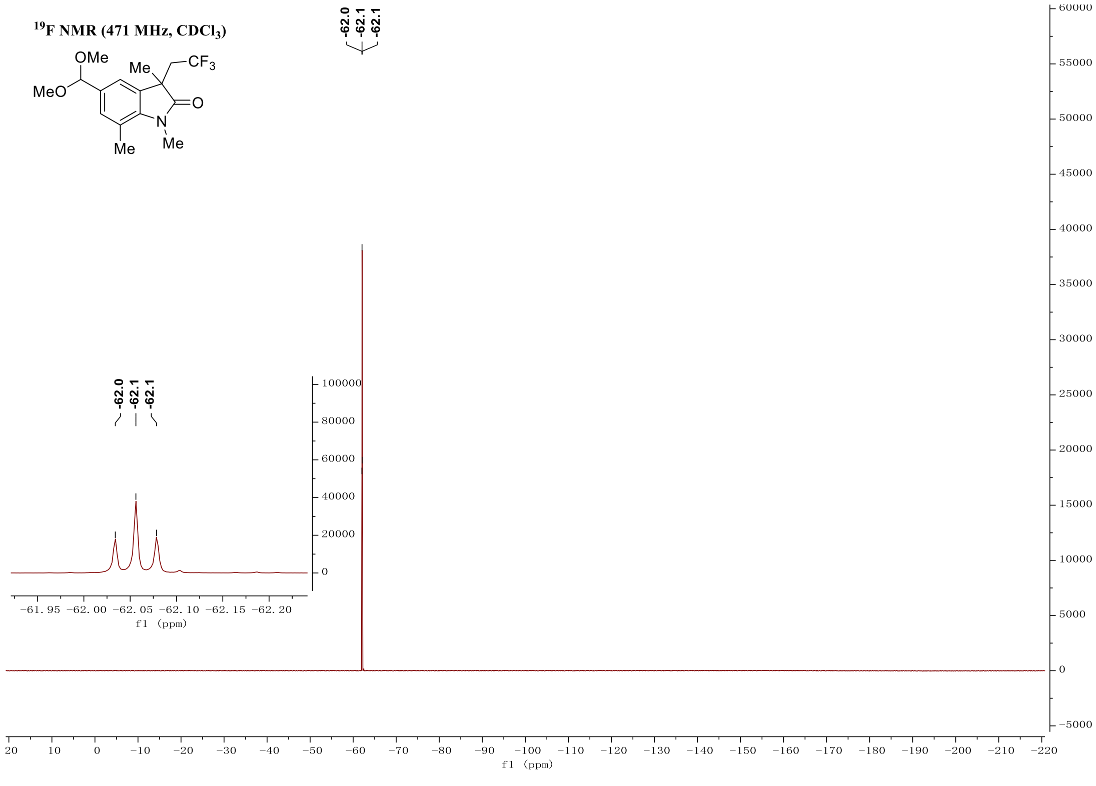

Supplementary Figure 181. ^{13}C **NMR and** ^{19}F **NMR spectra of compound 49.**

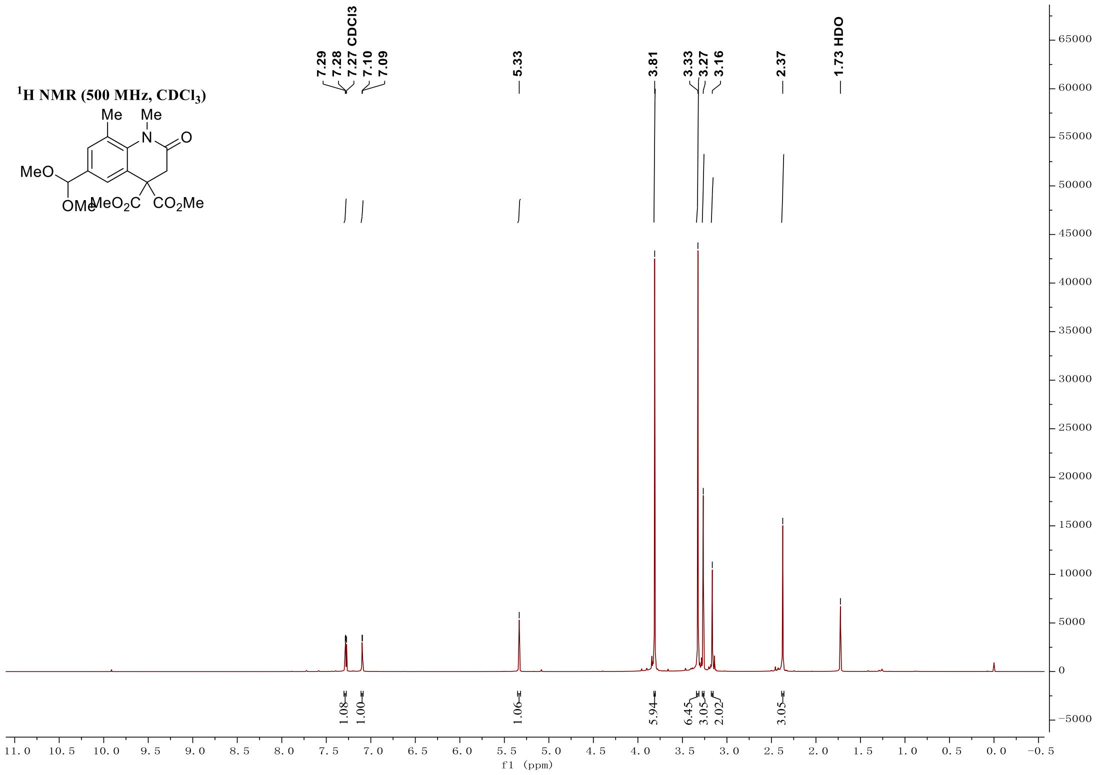

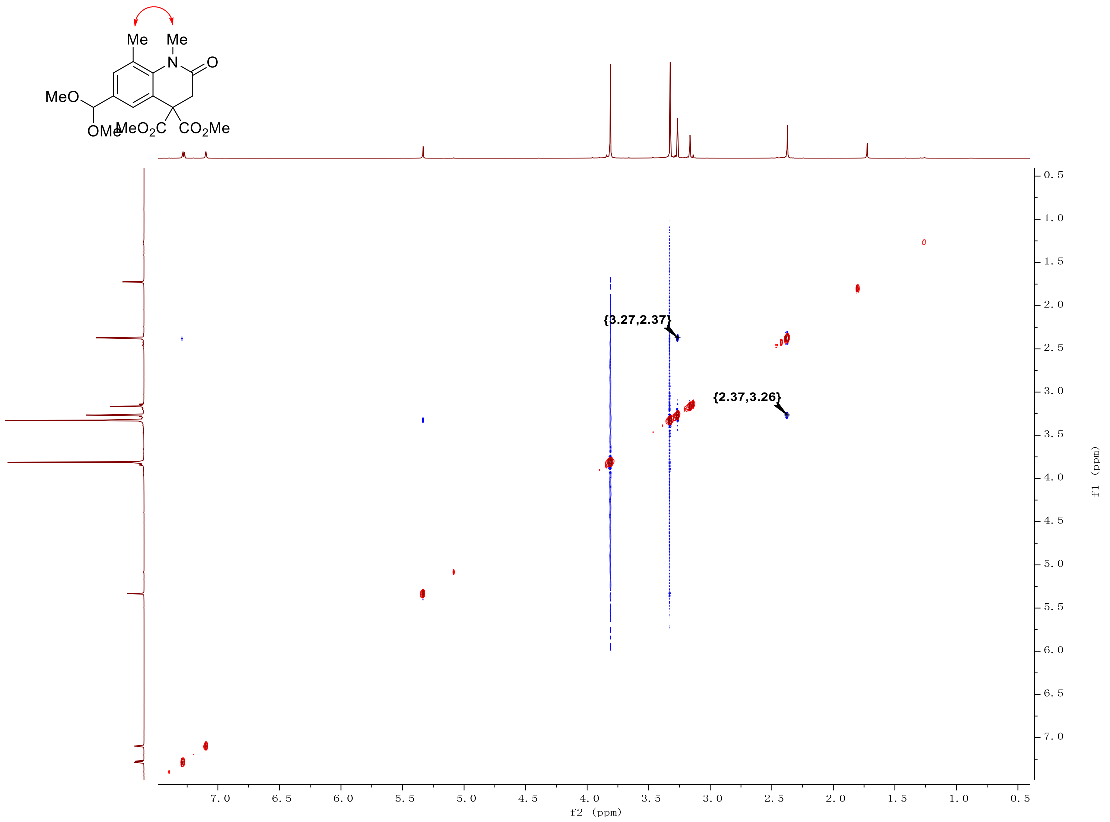

Supplementary Figure 182. ^{1}H NMR and 2D NOESY spectra of compound 50.

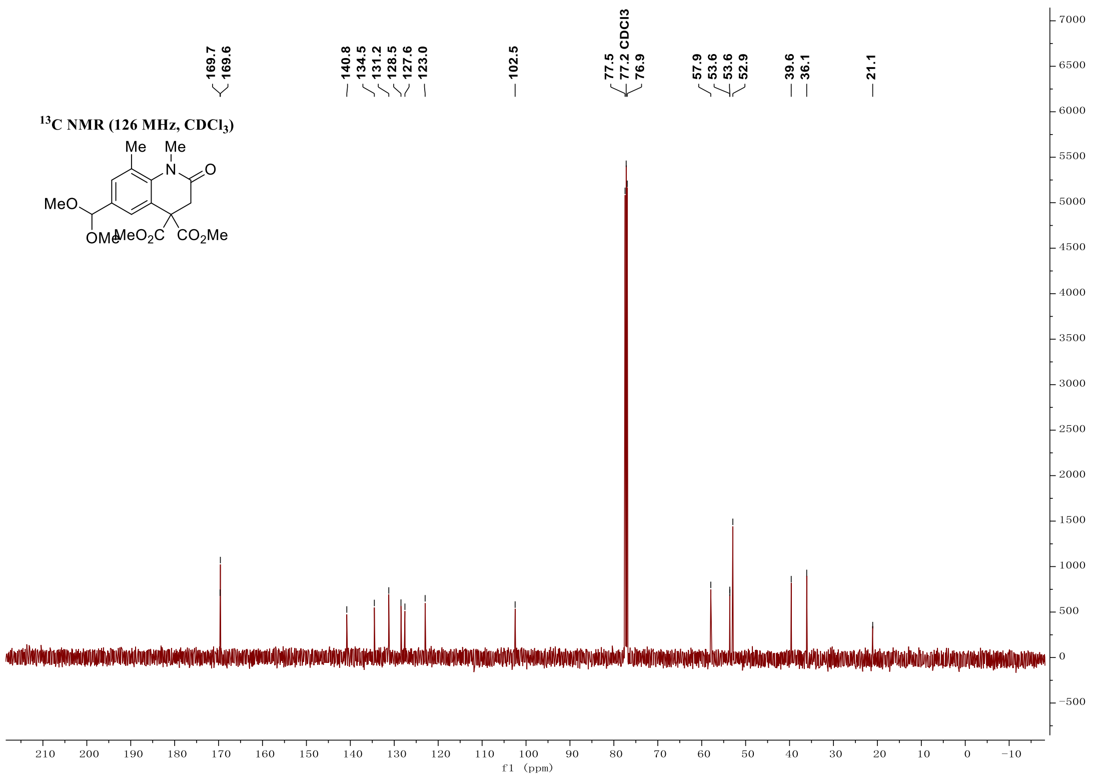

Supplementary Figure 183. ^{13}**C NMR spectrum of compound 50.**

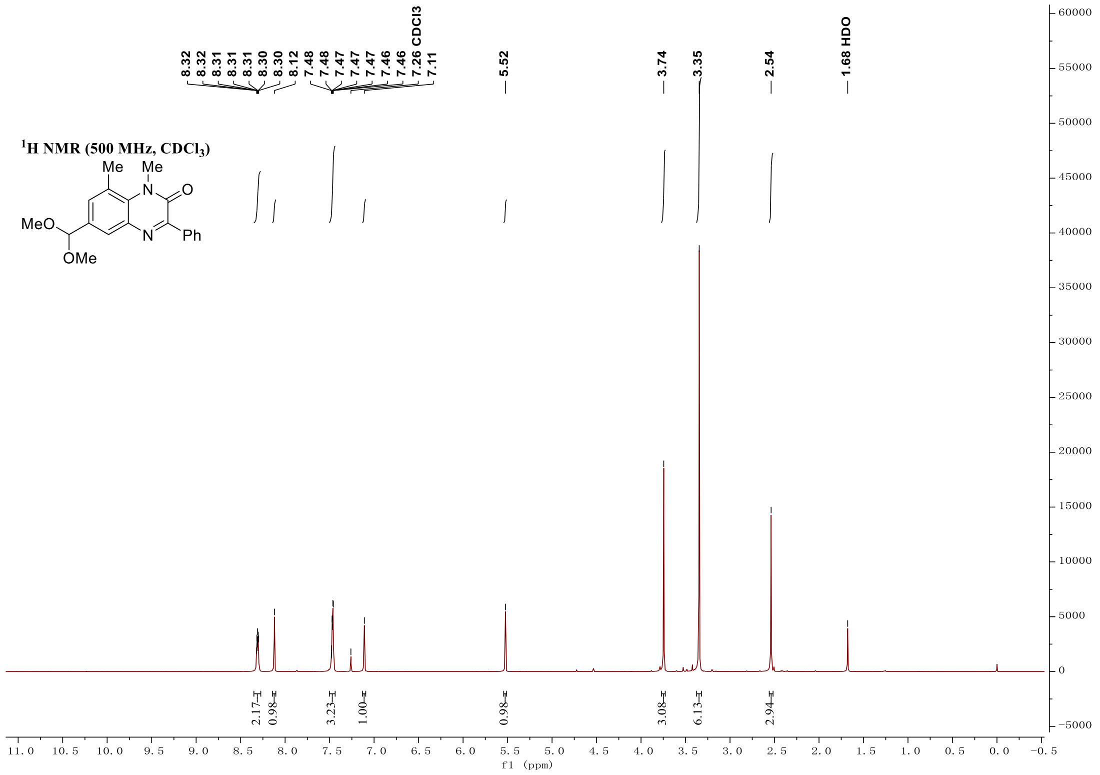

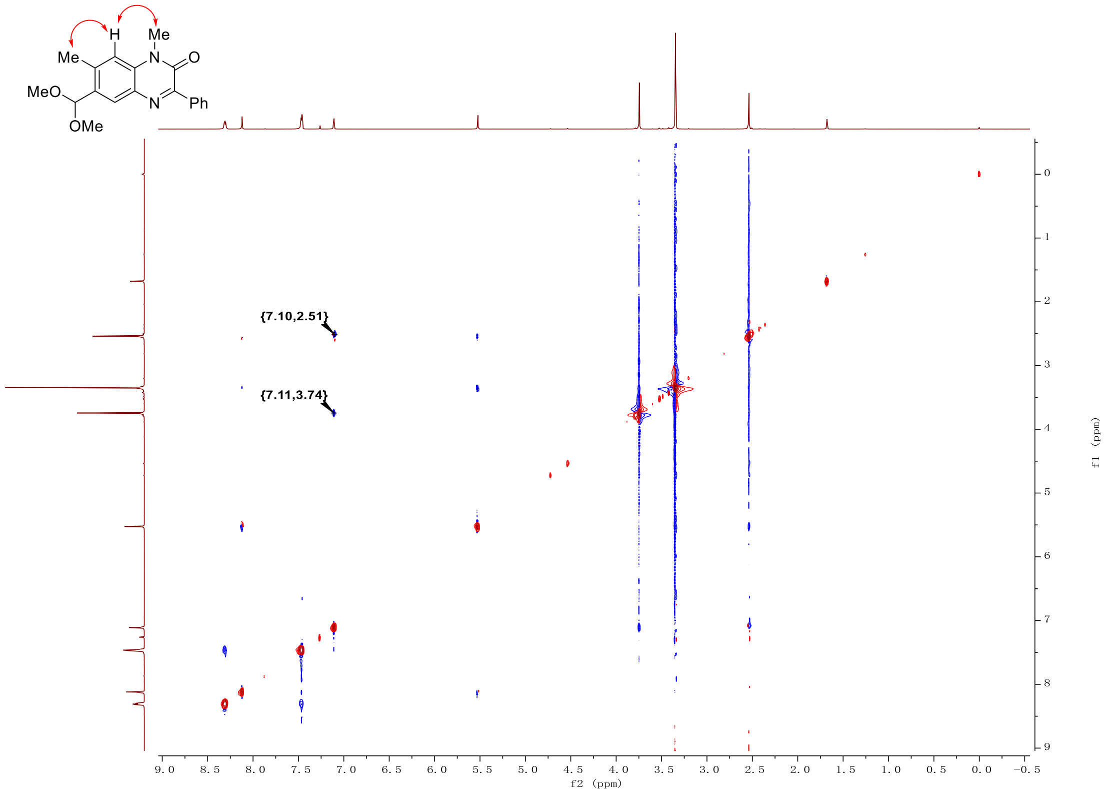

Supplementary Figure 184. ^{1}H NMR and 2D NOESY spectra of compound 51.

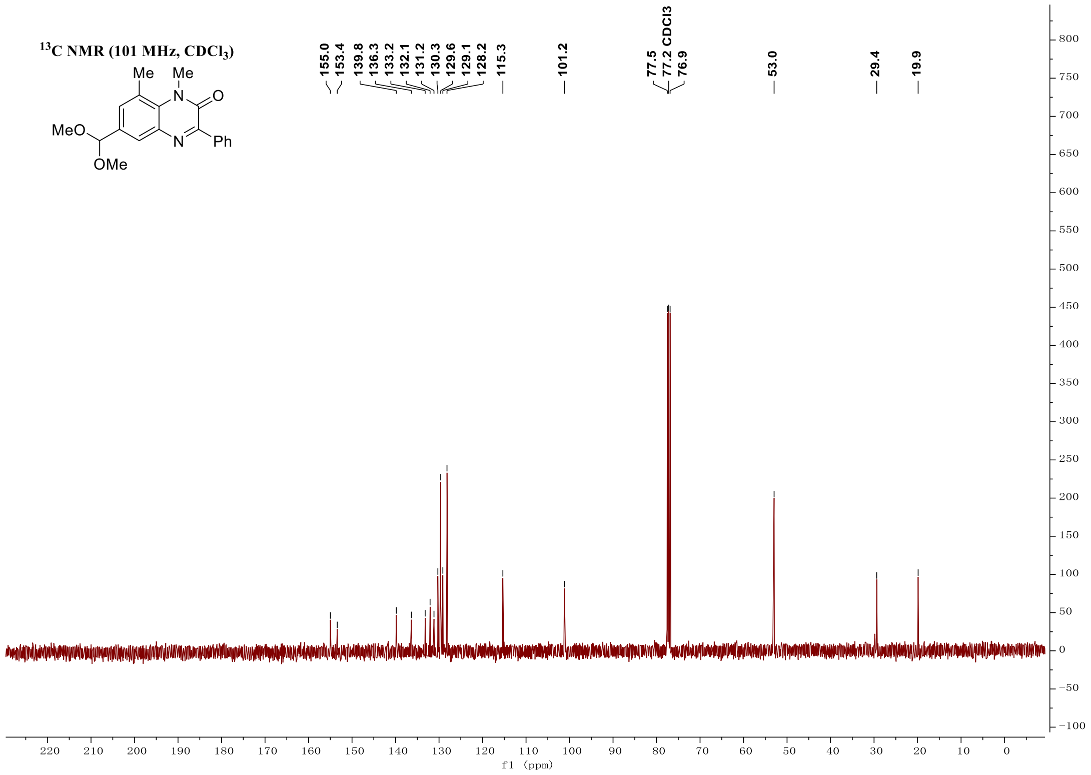

Supplementary Figure 185. ^{13}C NMR spectrum of compound 51.

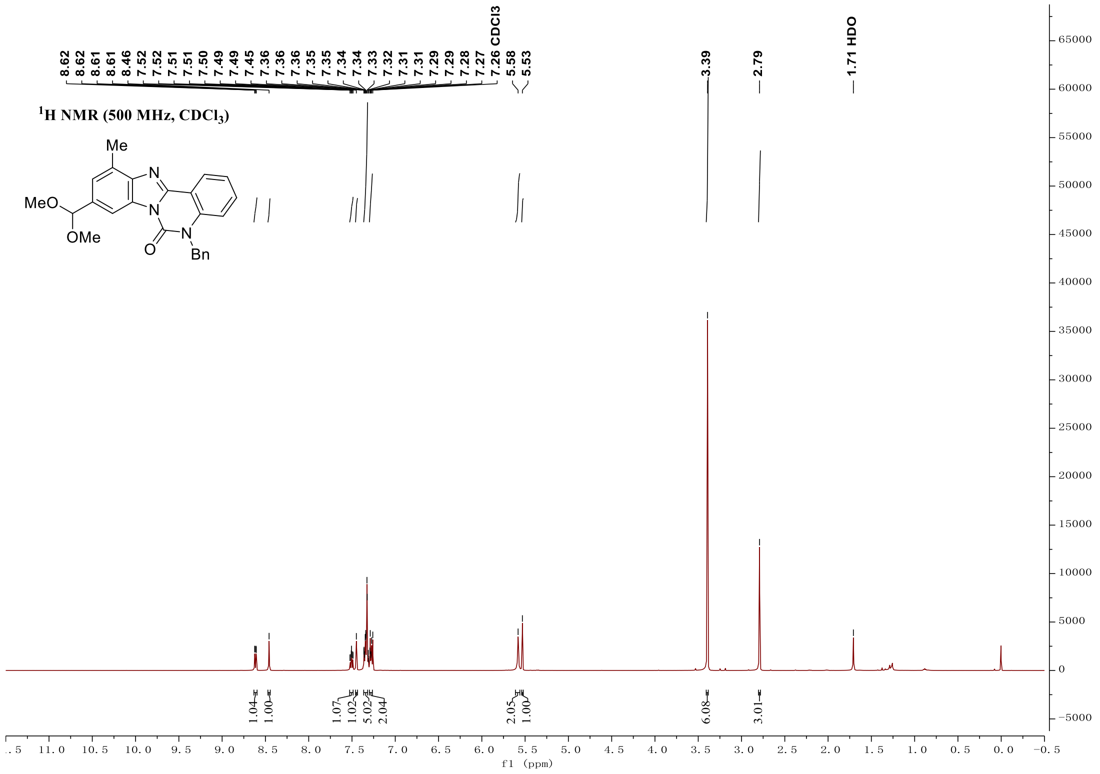

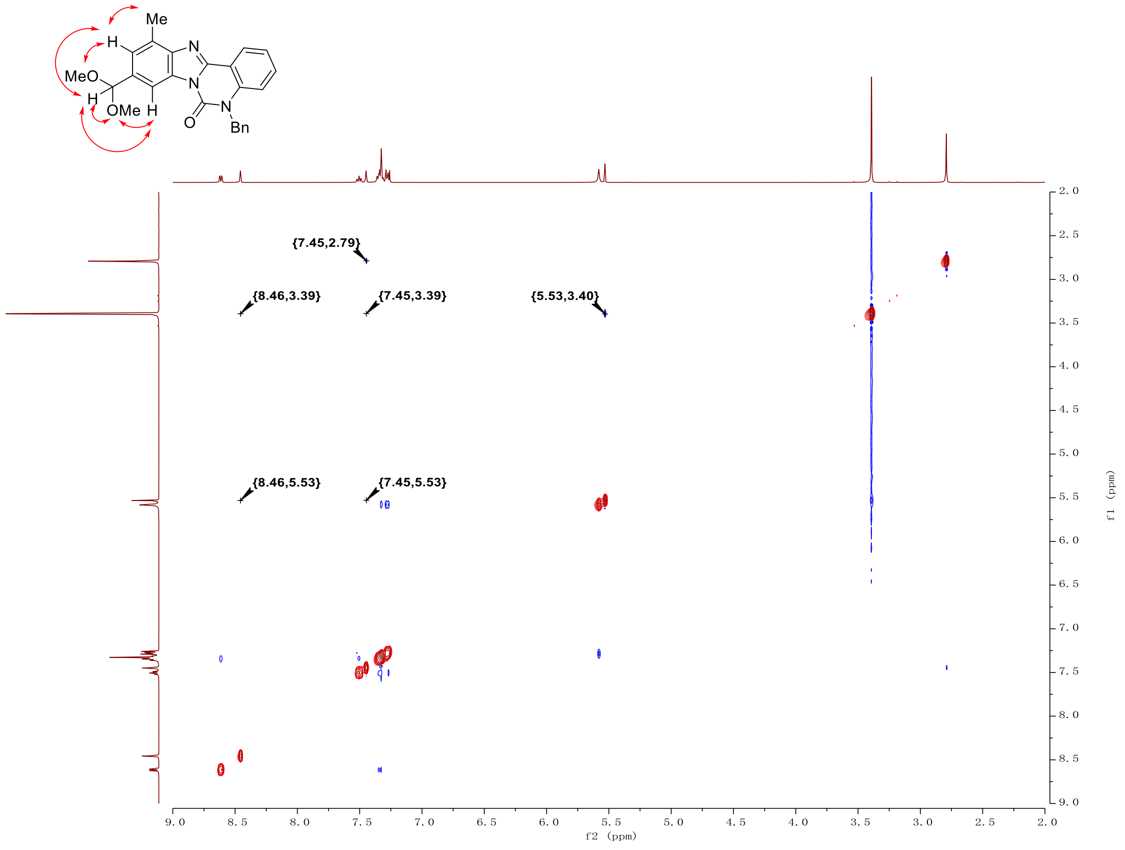

Supplementary Figure 186. ^{1}**H NMR and 2D NOESY spectra of compound 56.**

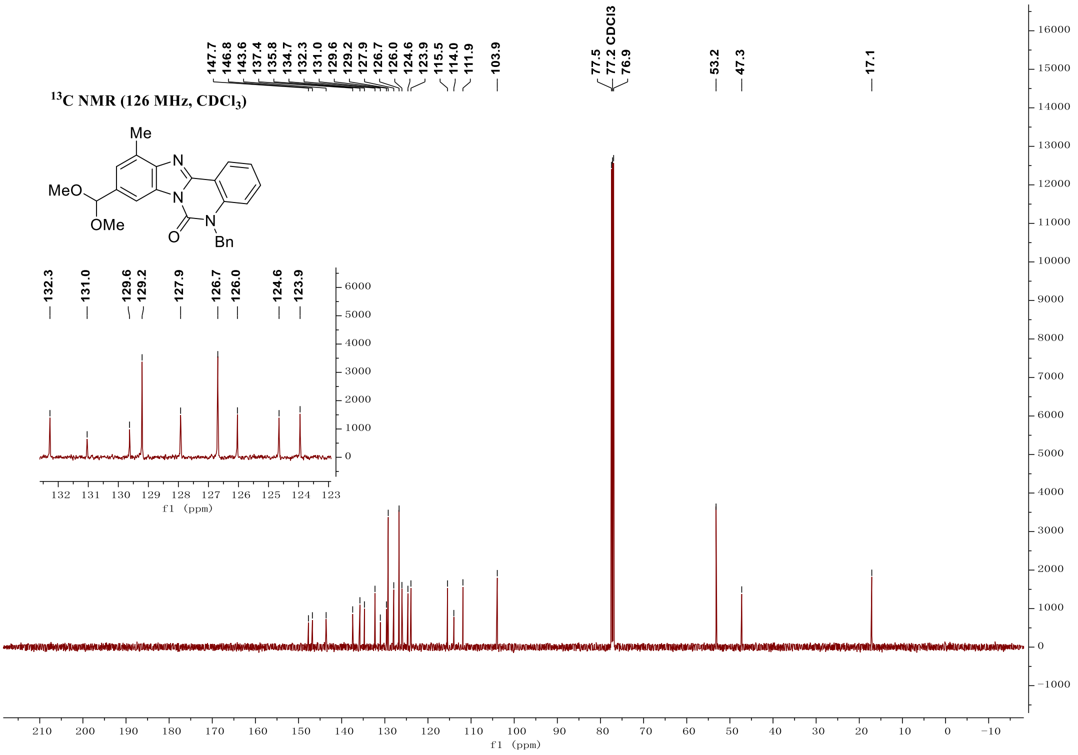

Supplementary Figure 187. ^{13}C **NMR spectrum of compound 56.**

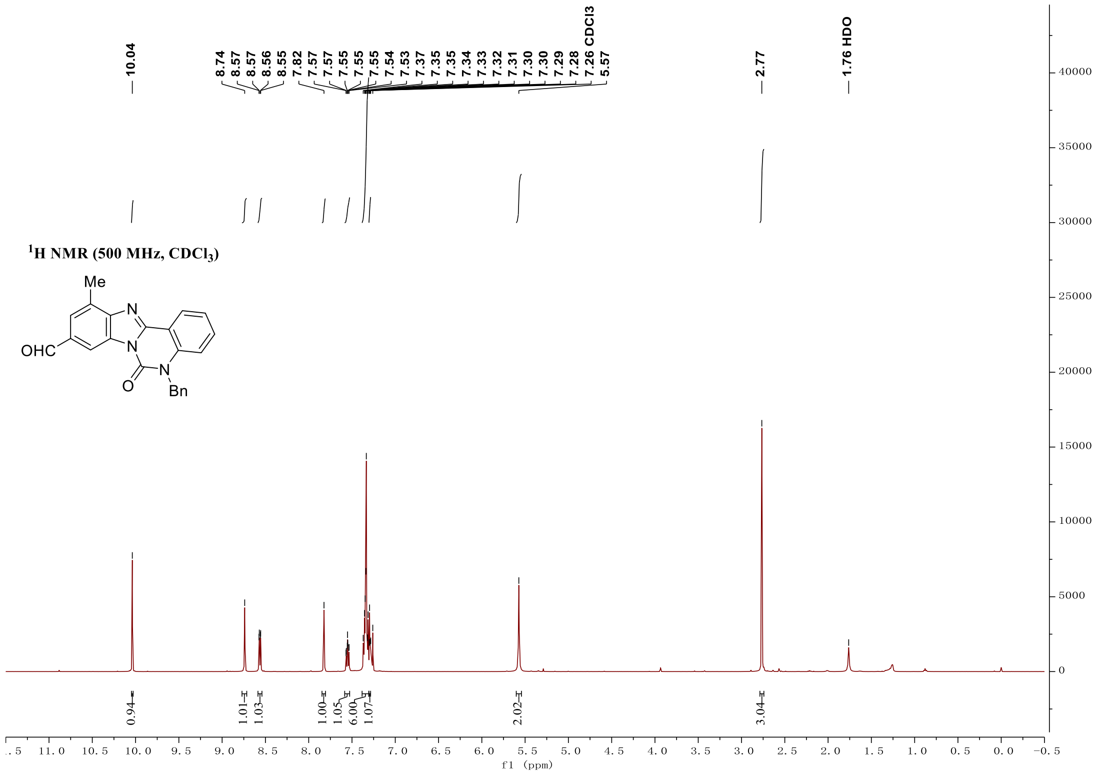

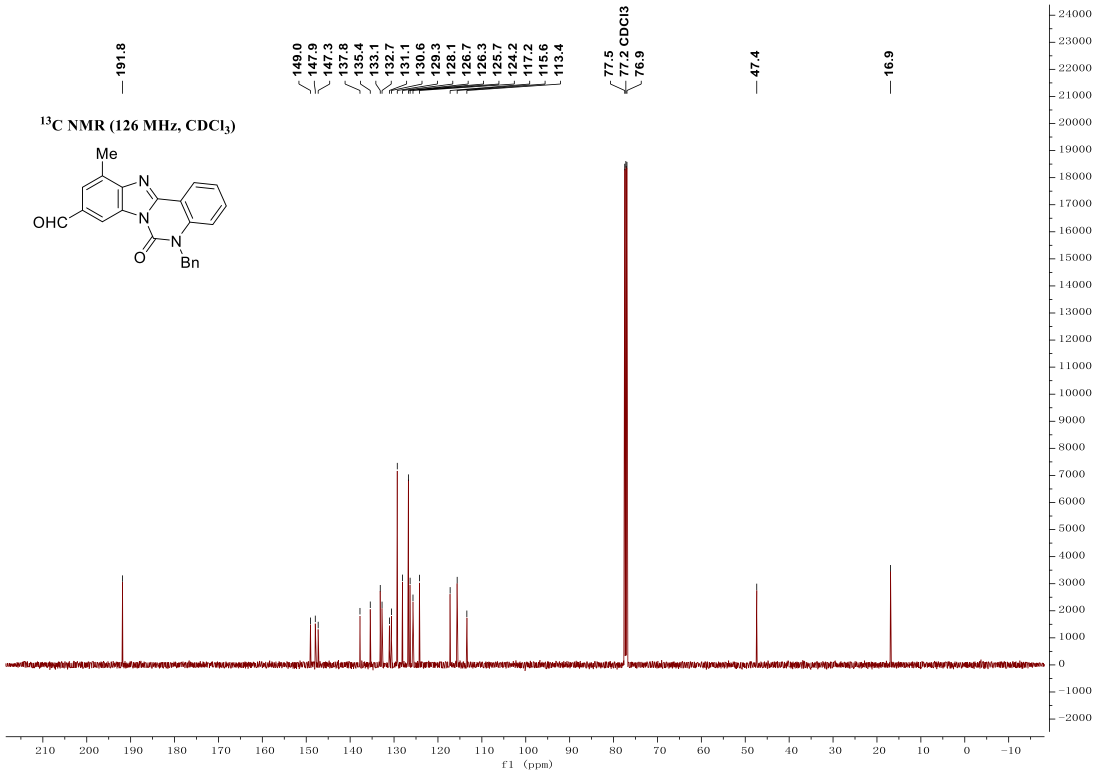

Supplementary Figure 188. ^{1}H NMR and ^{13}C NMR spectra of compound 57.

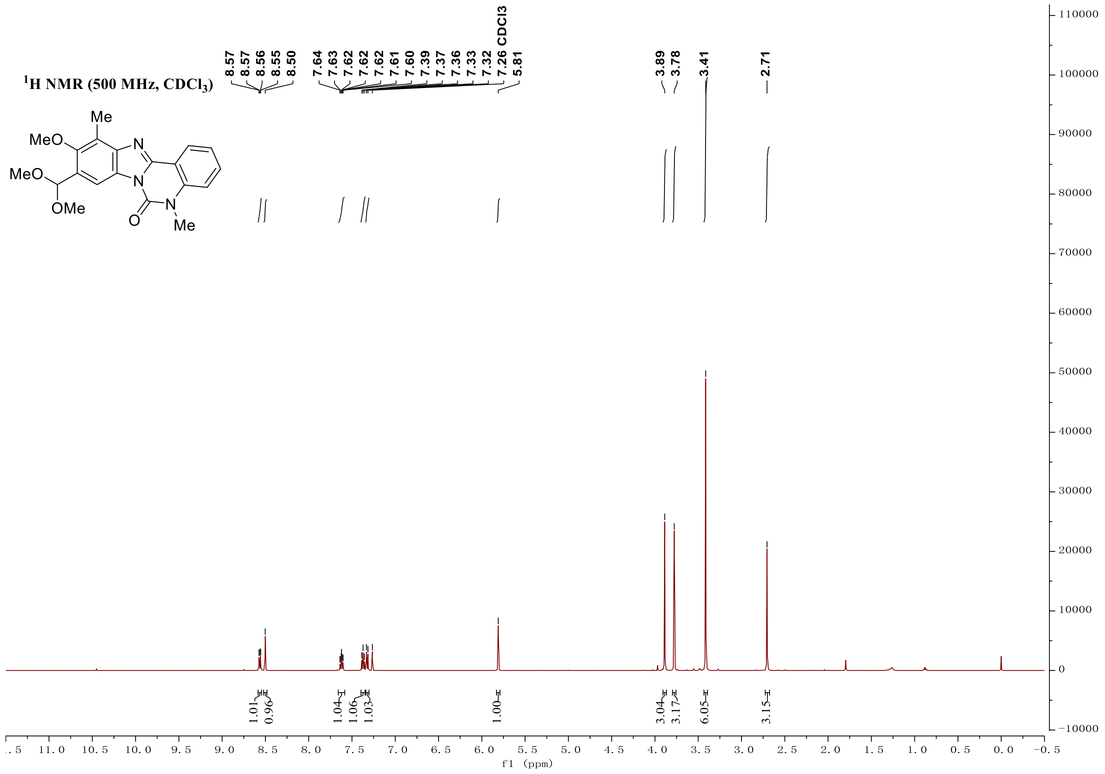

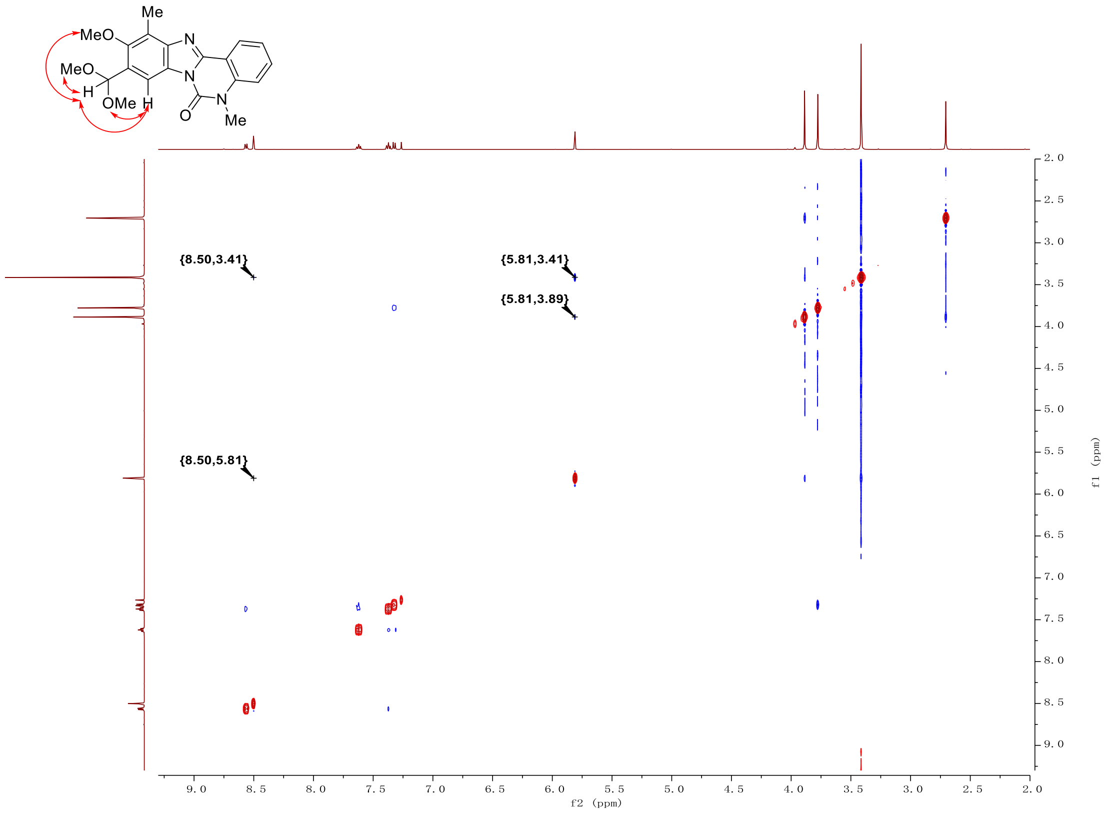

Supplementary Figure 189. ^{1}H NMR and 2D NOESY spectra of compound 58.

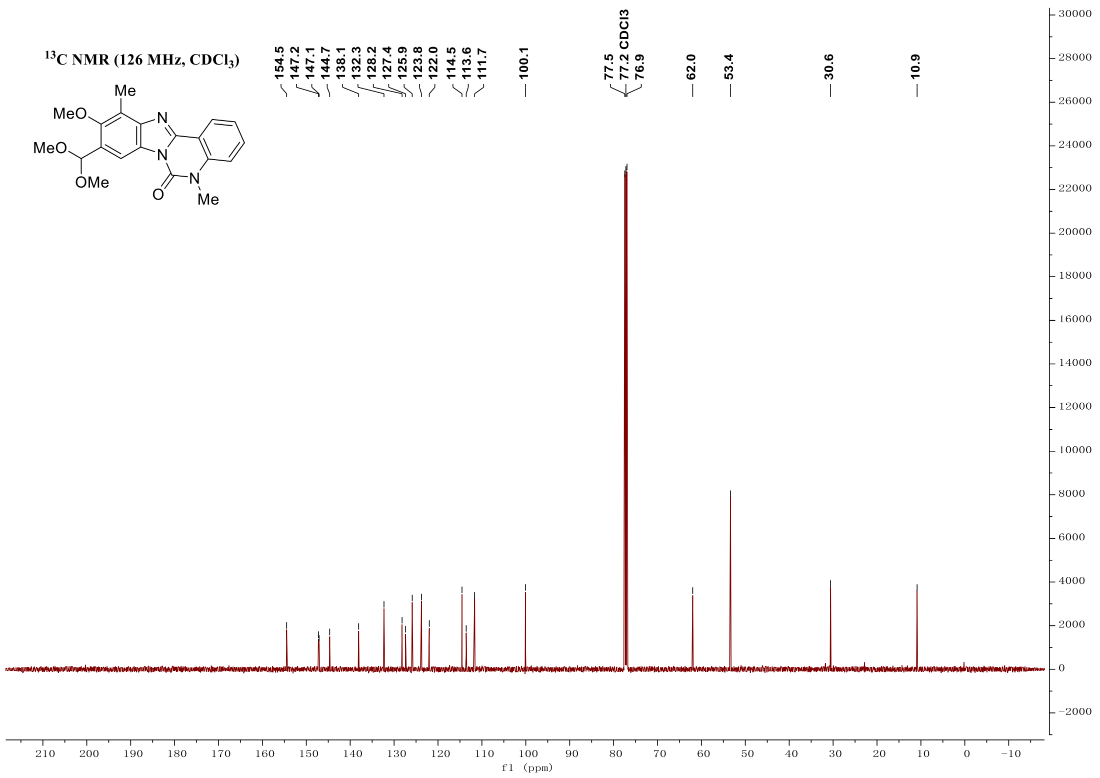

Supplementary Figure 190. ^{13}C NMR spectrum of compound 58.

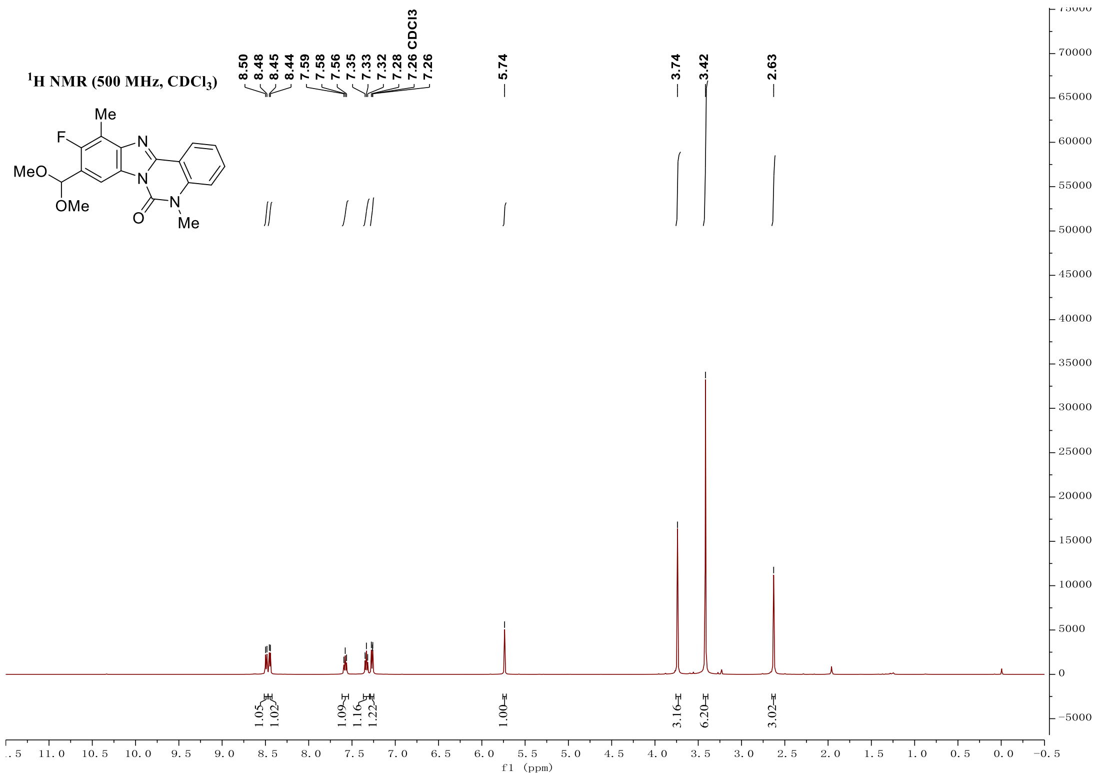

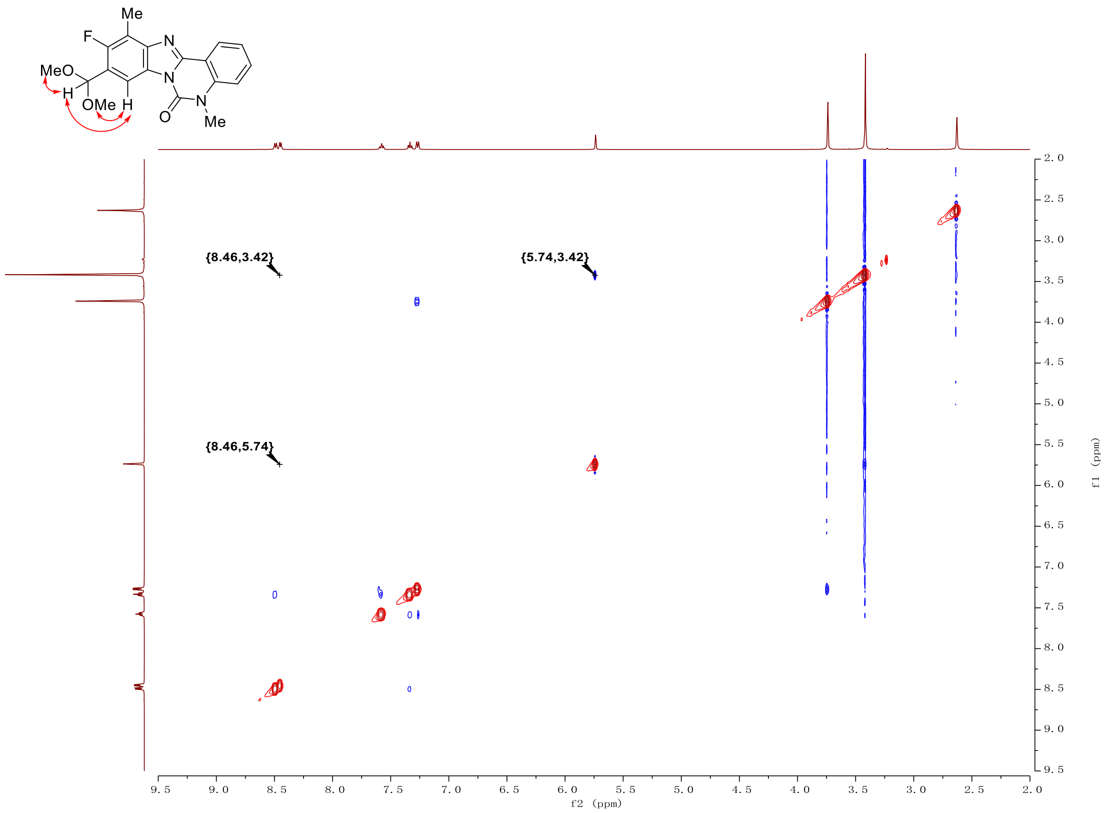

Supplementary Figure 191. ^{1}**H NMR and 2D NOESY spectra of compound 59.**

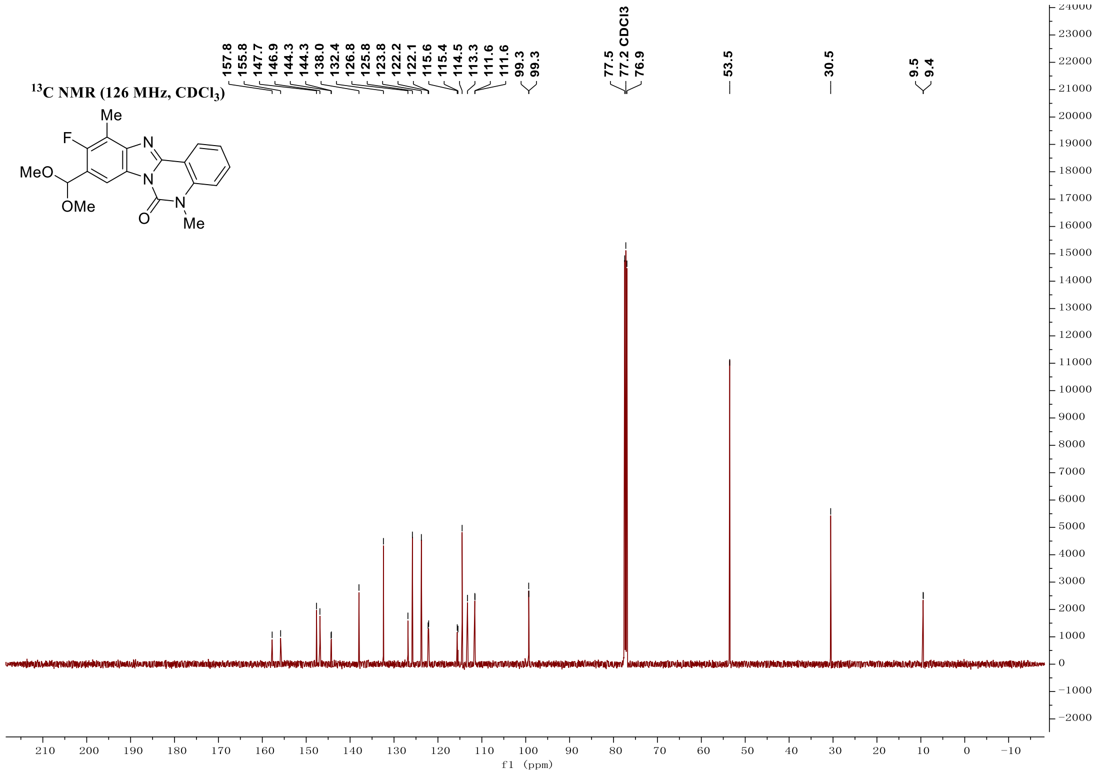

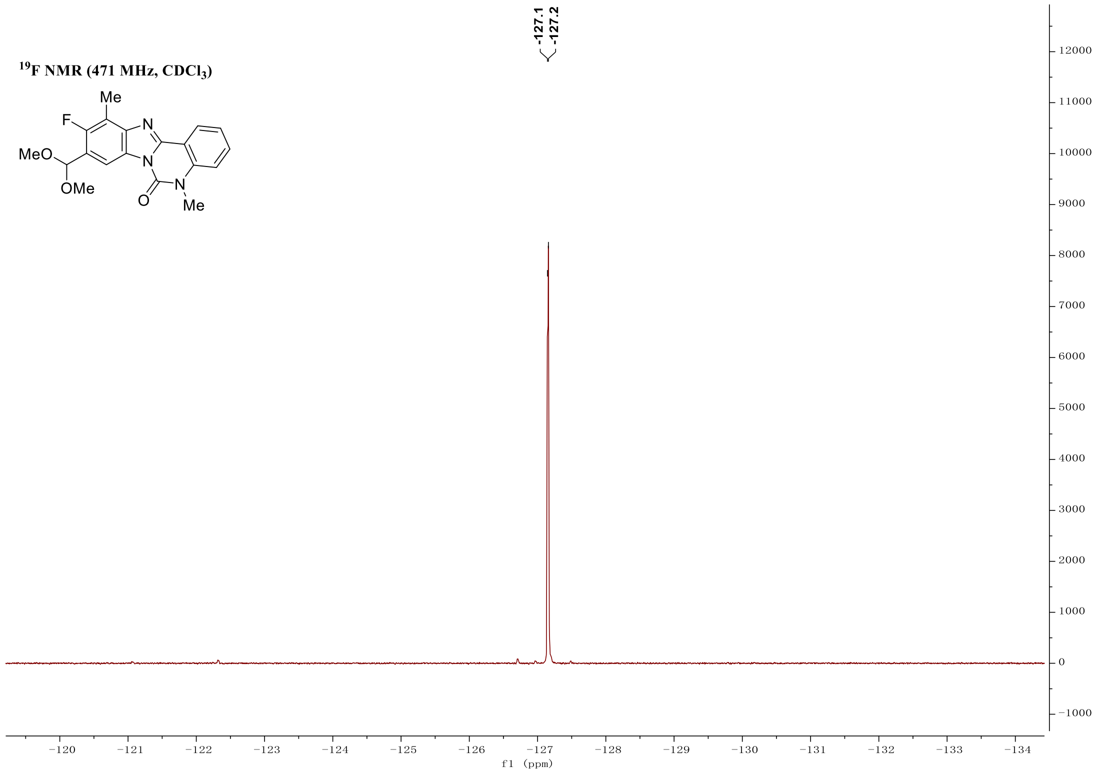

Supplementary Figure 192. ^{13}C NMR and ^{19}F NMR spectra of compound 59.

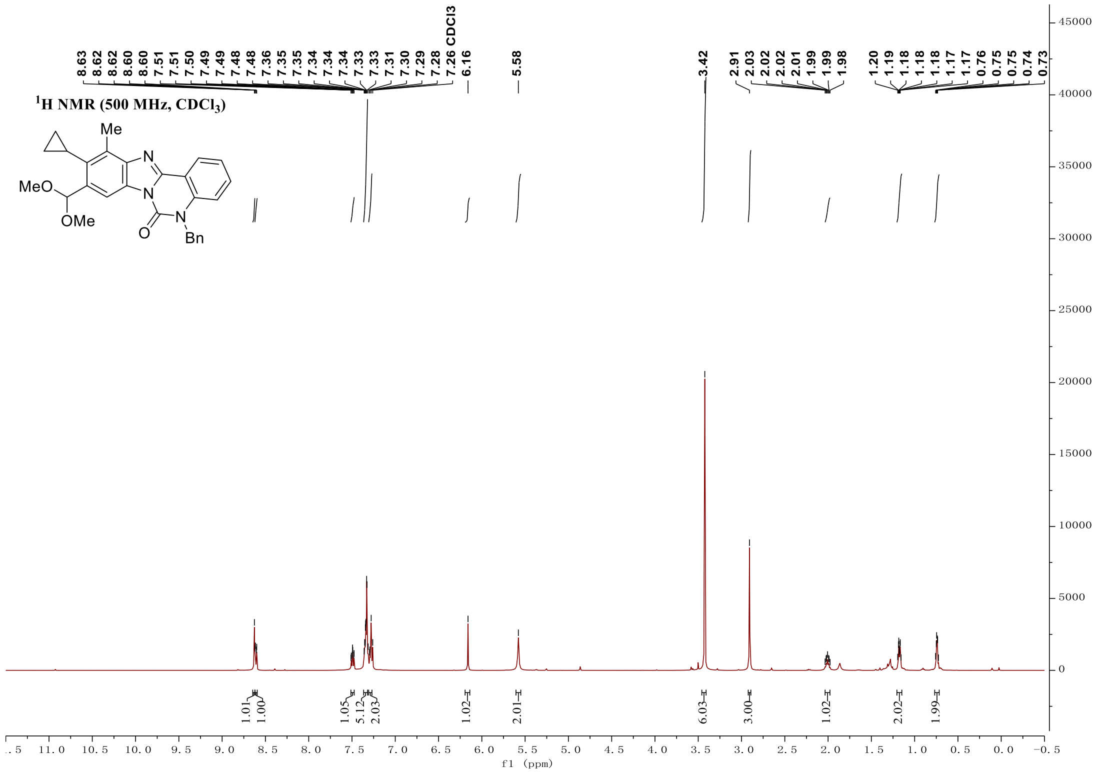

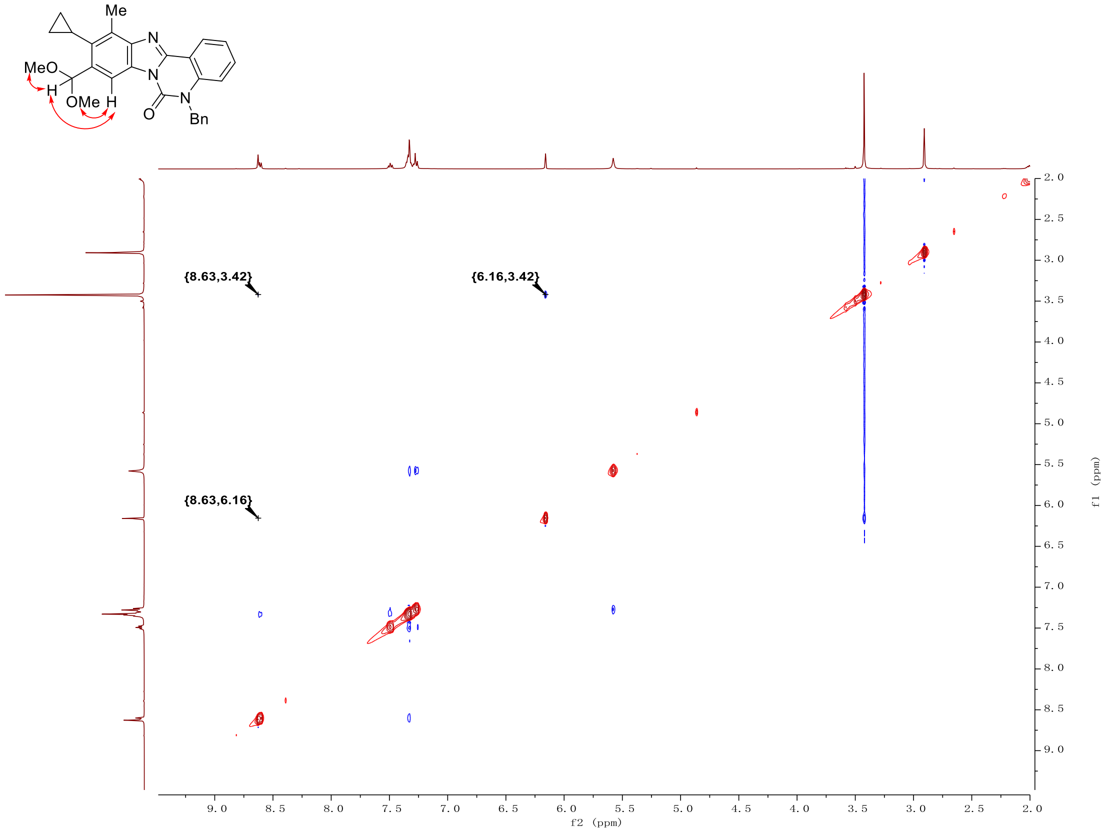

Supplementary Figure 193. ^{1}H NMR and 2D NOESY spectra of compound 60.

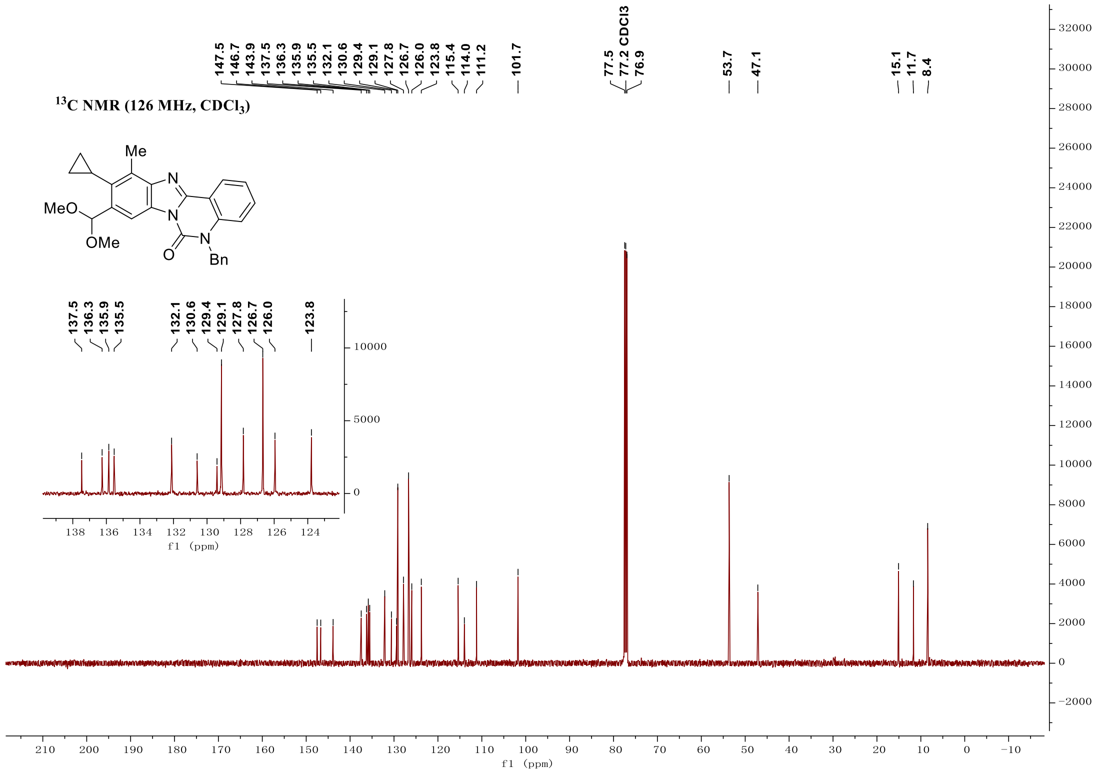

Supplementary Figure 194. ^{13}C **NMR spectrum of compound 60.**

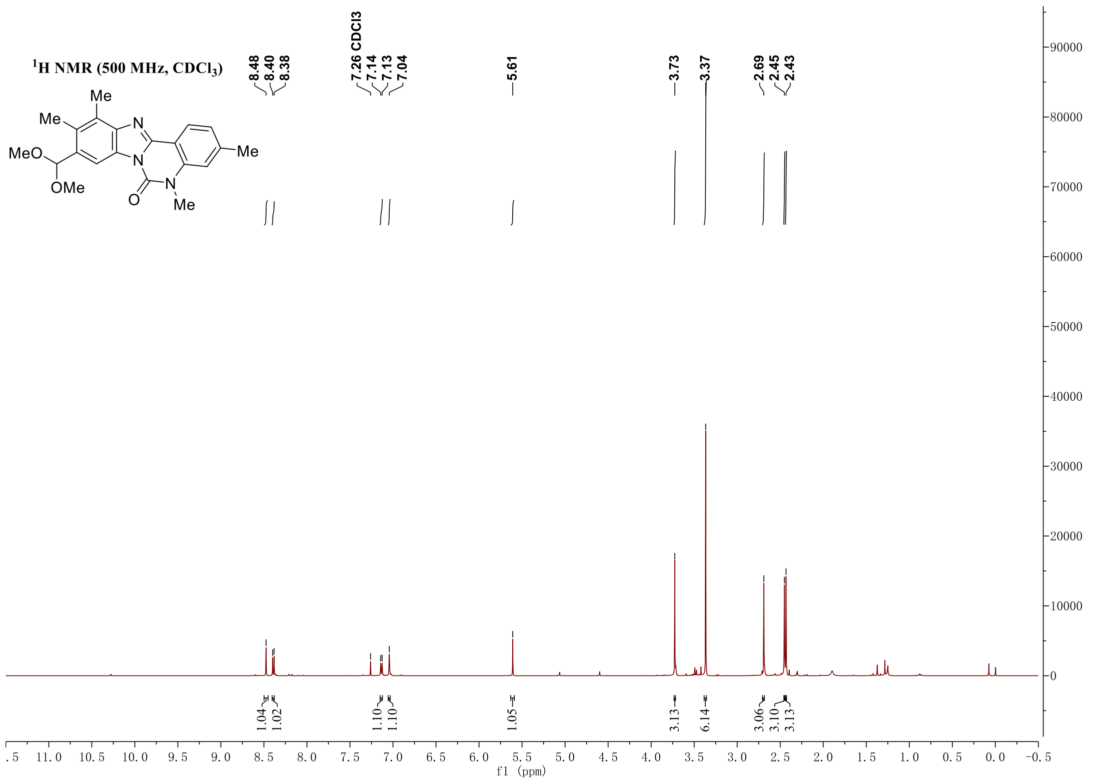

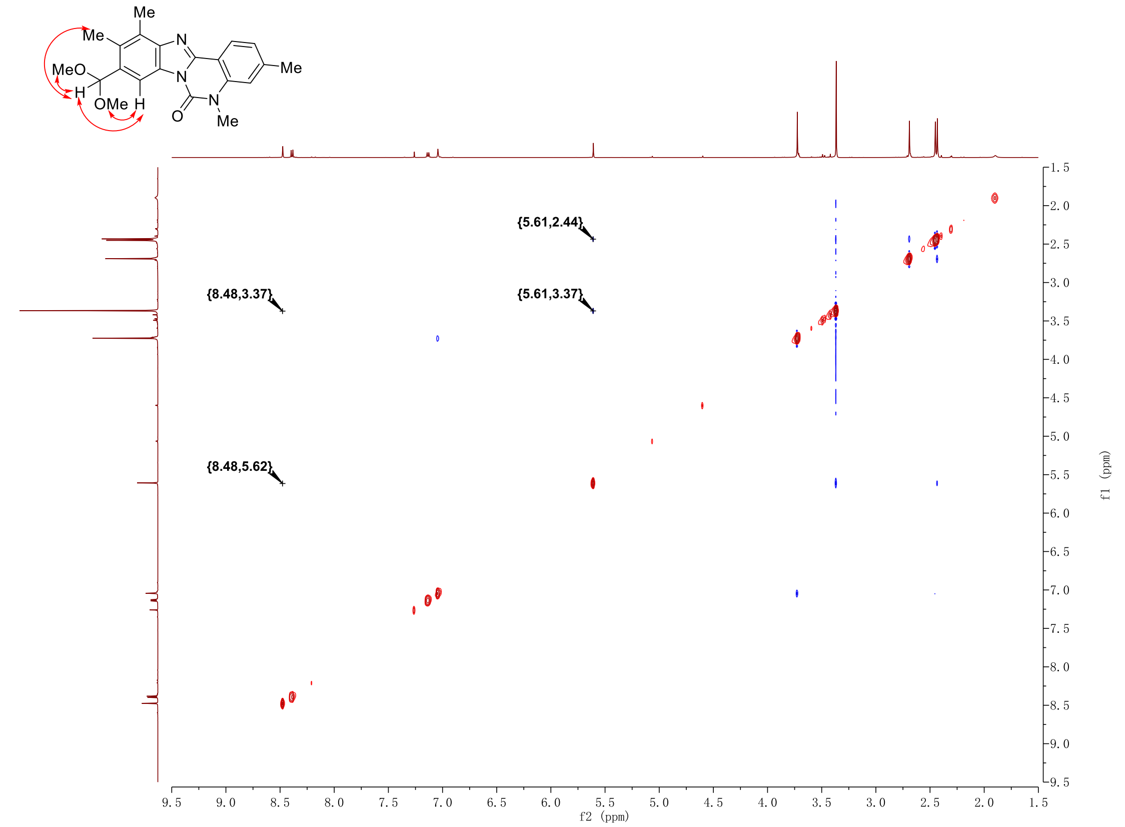

Supplementary Figure 195. ^{1}H NMR and 2D NOESY spectra of compound 61.

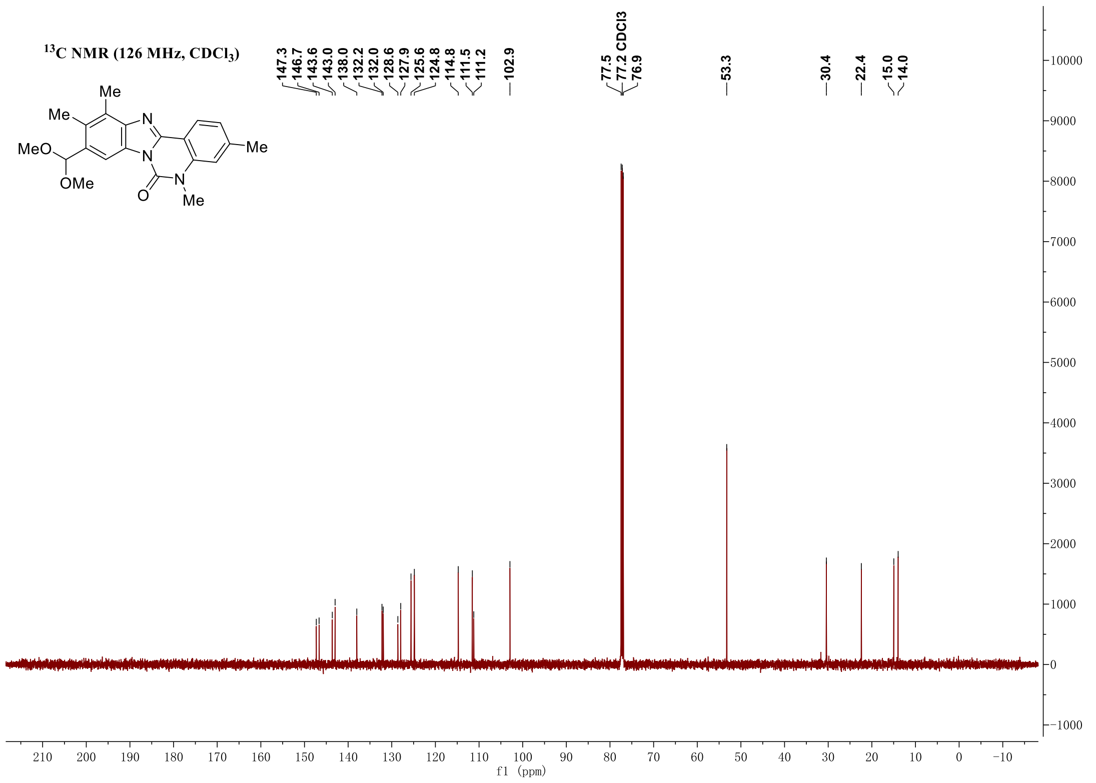

Supplementary Figure 196. ^{13}C **NMR spectrum of compound 61.**

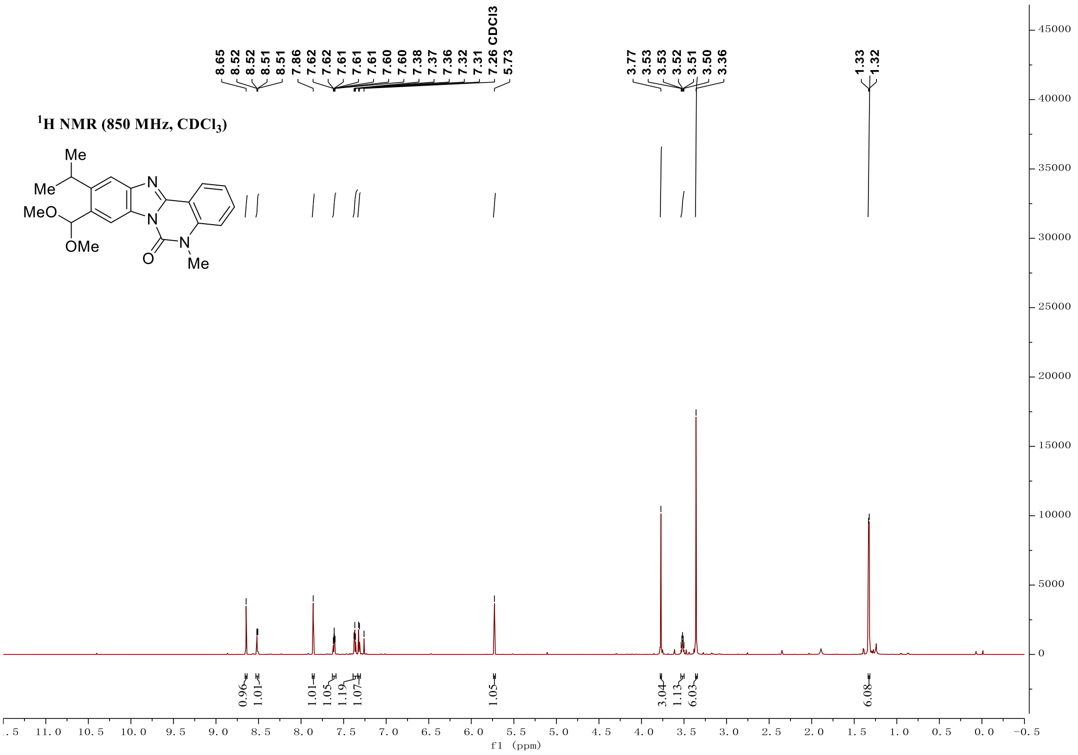

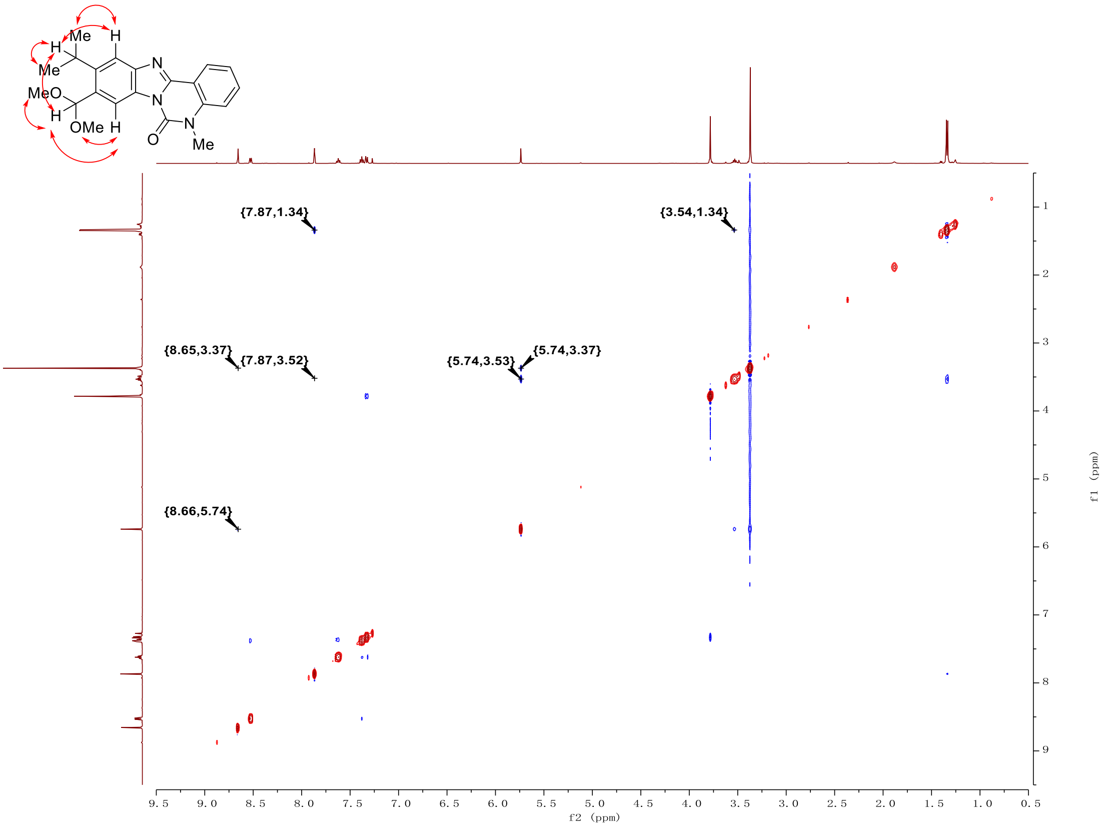

Supplementary Figure 197. ^{1}H NMR and 2D NOESY spectra of compound 62.

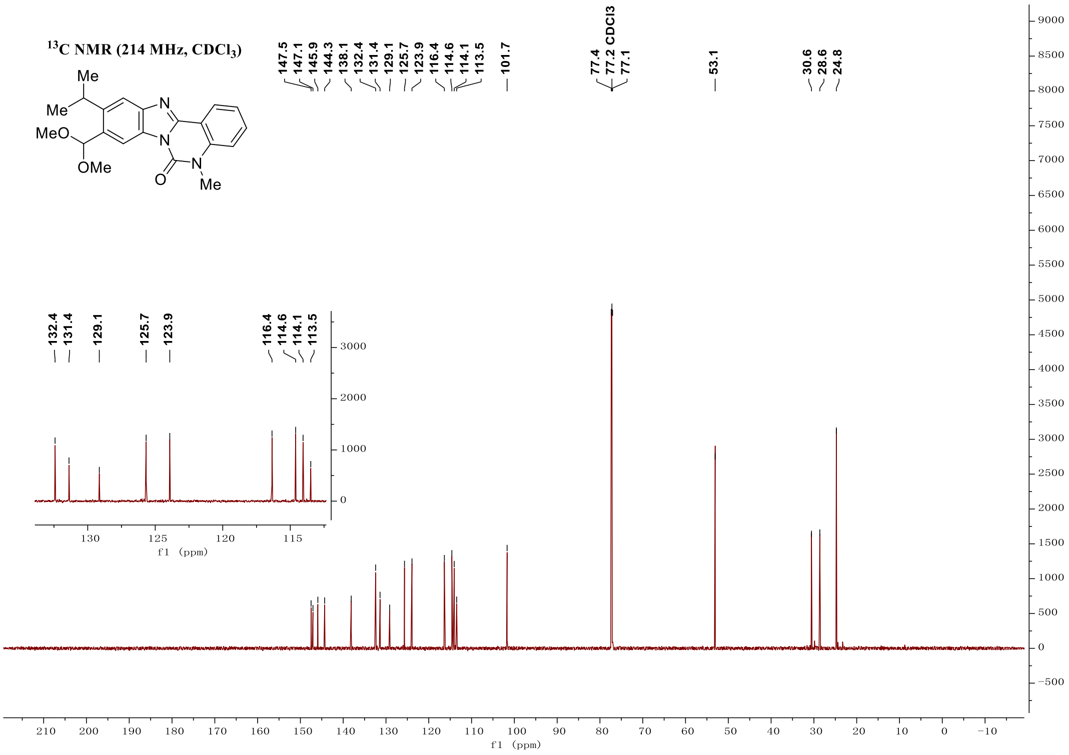

Supplementary Figure 198. ^{13}**C NMR spectrum of compound 62.**

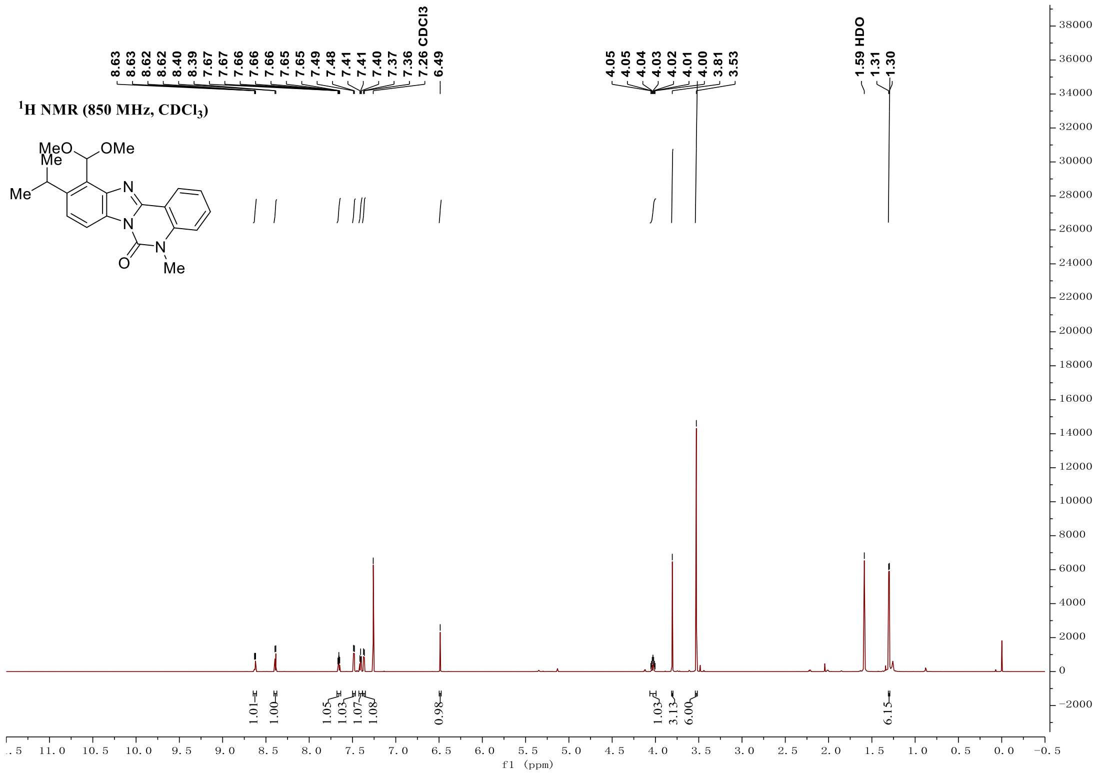

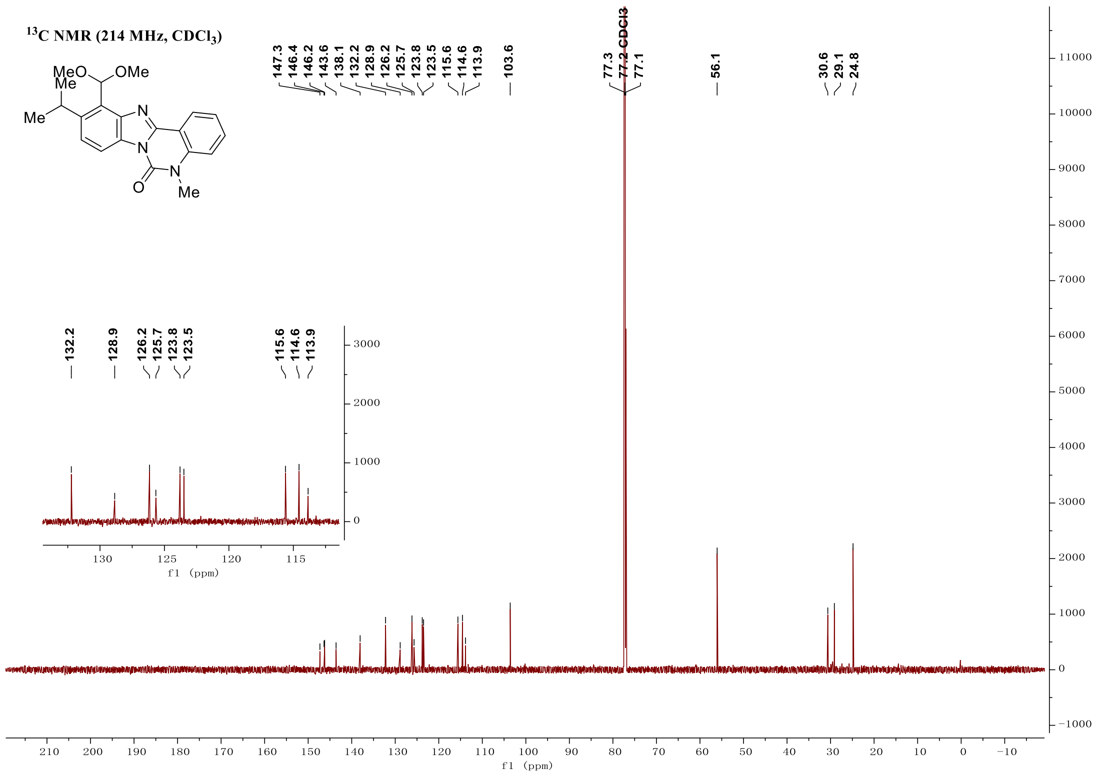

Supplementary Figure 199. ^{1}H NMR and ^{13}C NMR spectra of compound 62'.

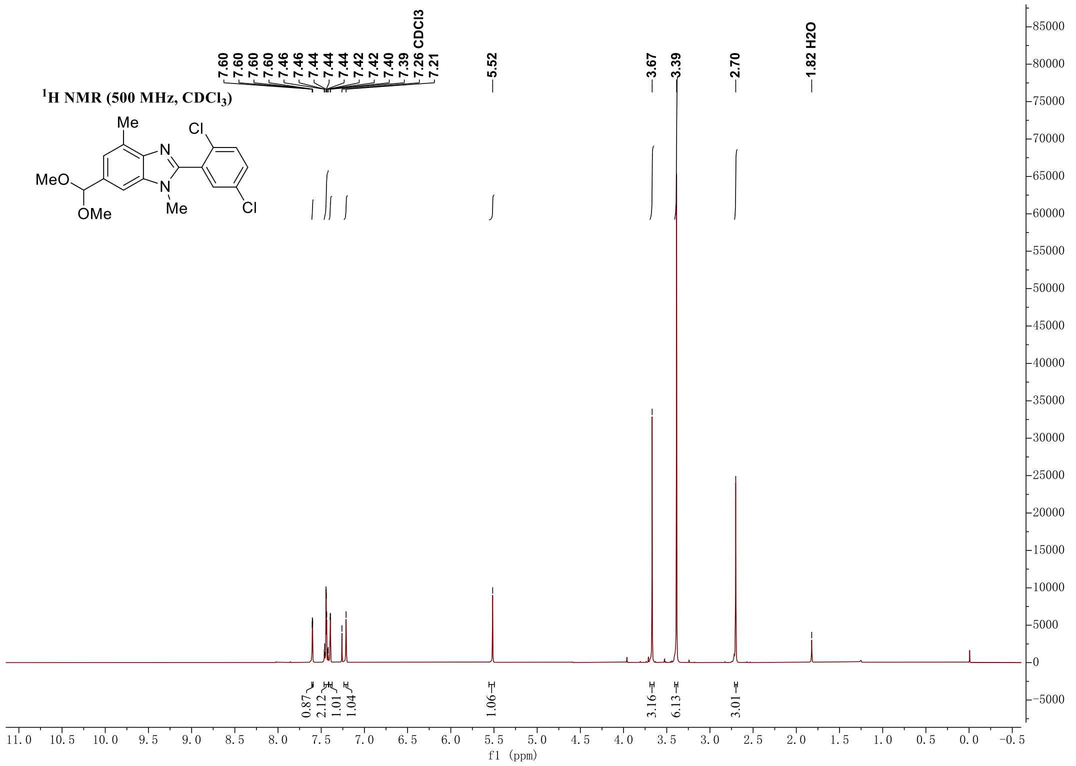

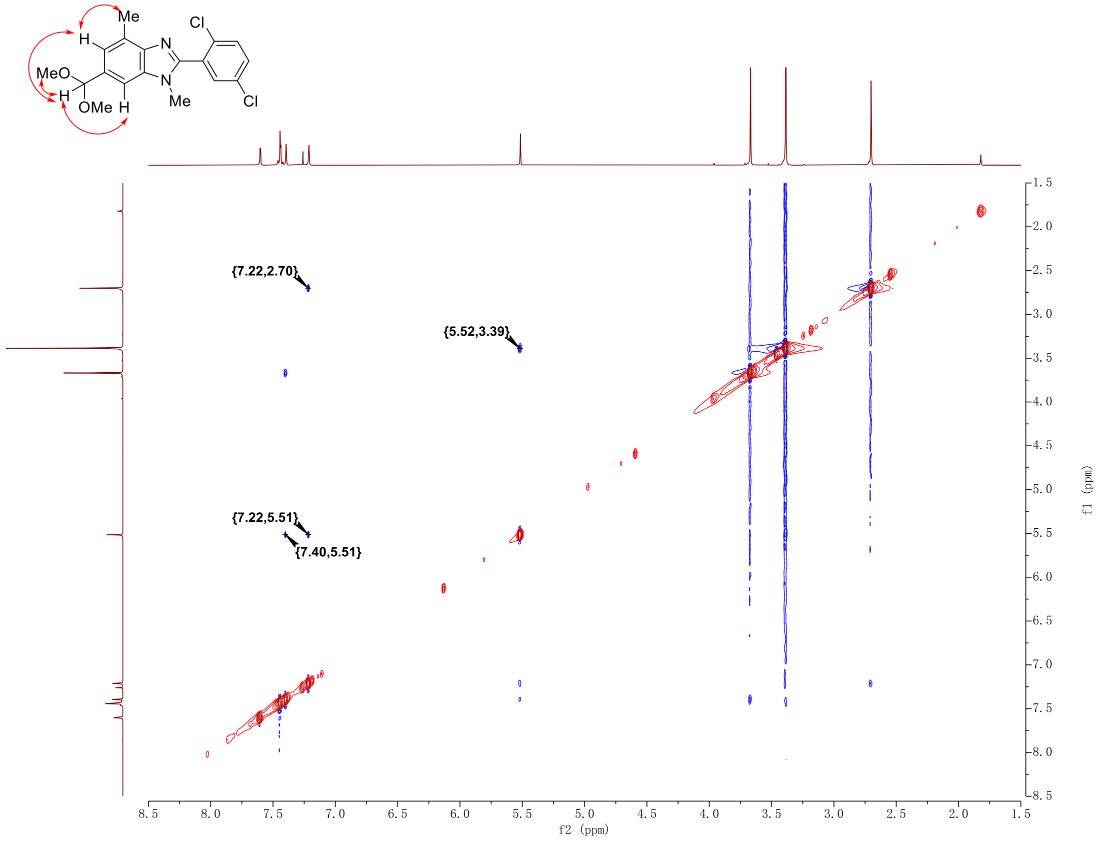

Supplementary Figure 200. ^{1}H NMR and 2D NOESY spectra of compound 63.

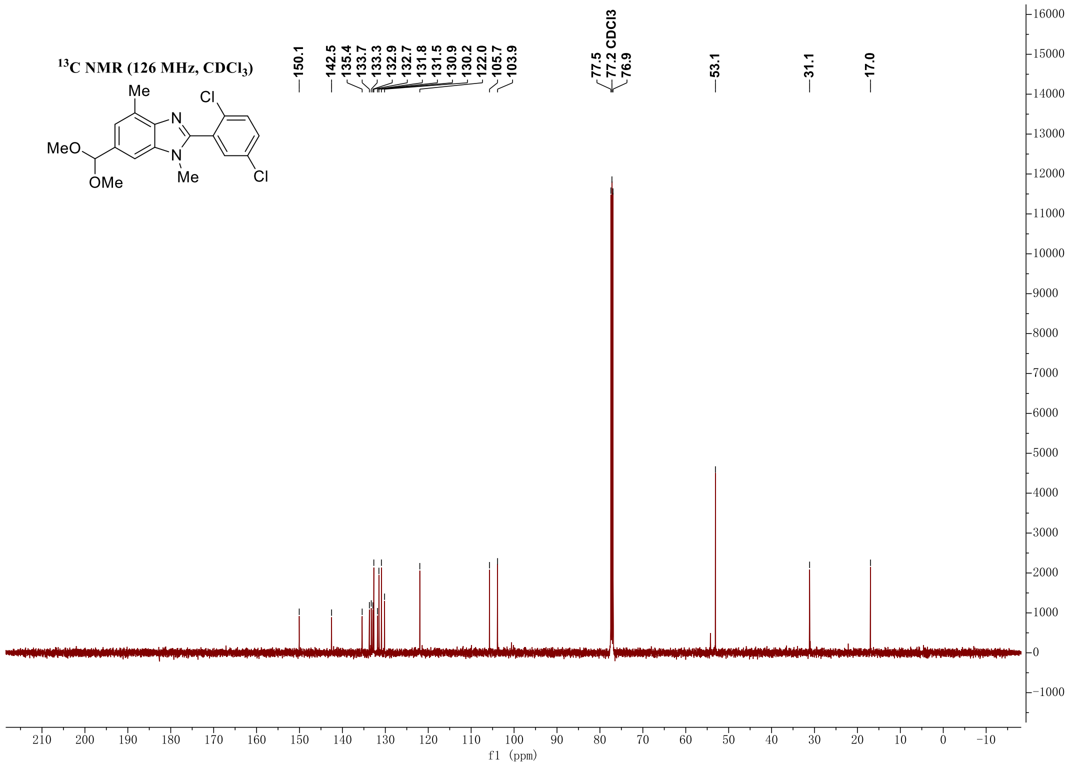

Supplementary Figure 201. ^{13}C NMR spectrum of compound 63.

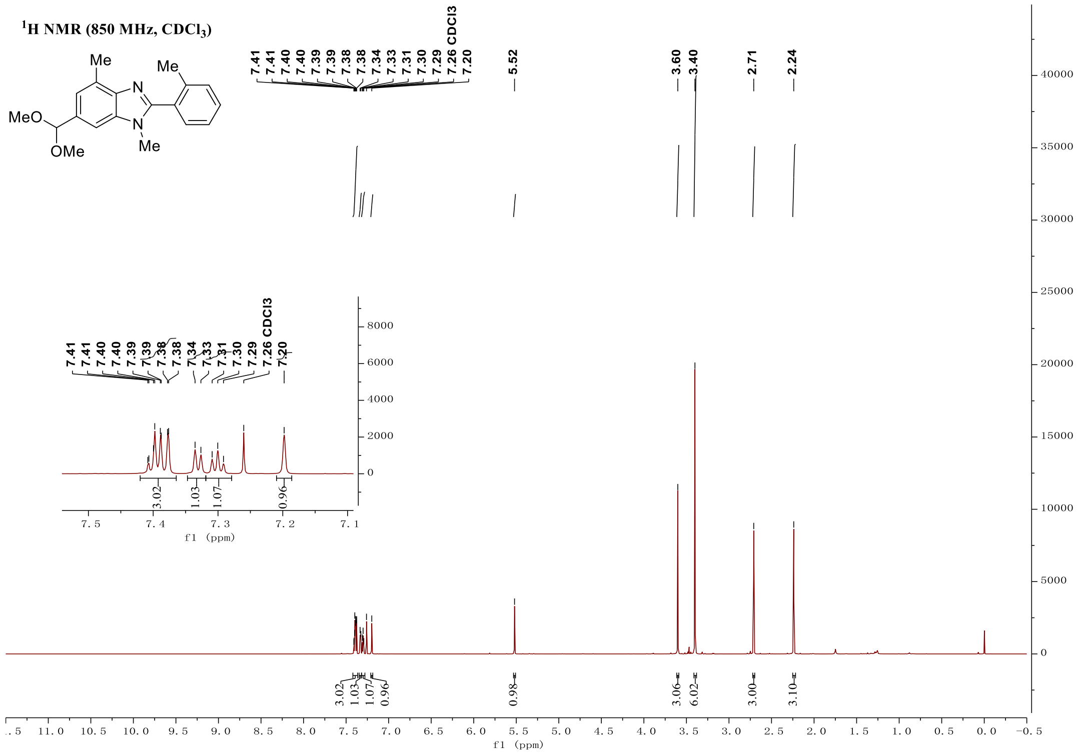

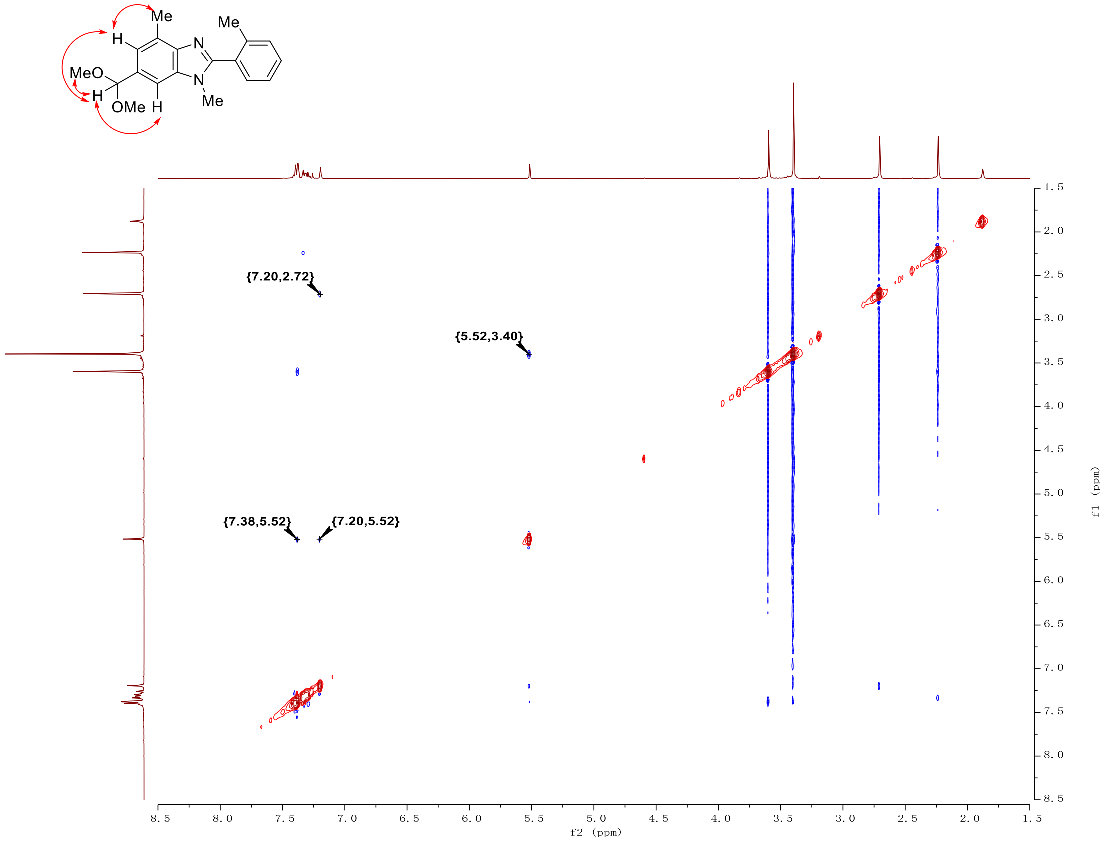

Supplementary Figure 202. ^{1}H NMR and 2D NOESY spectra of compound 64.

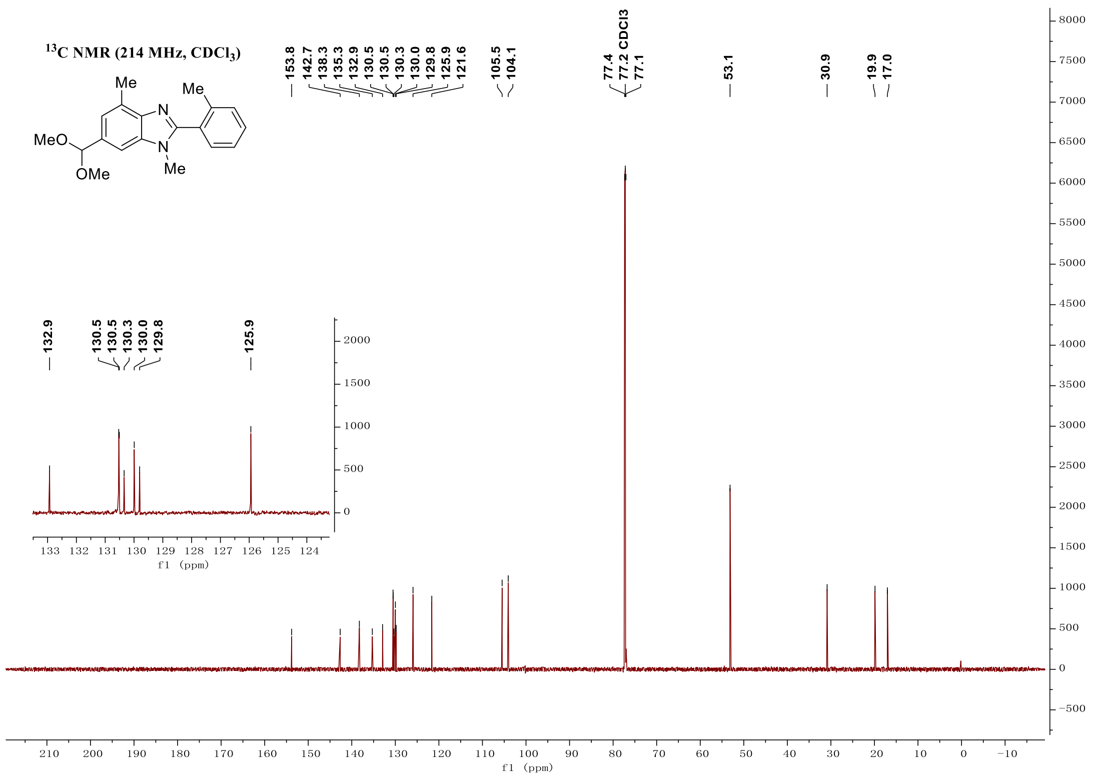

Supplementary Figure 203. ^{13}**C NMR spectrum of compound 64.**

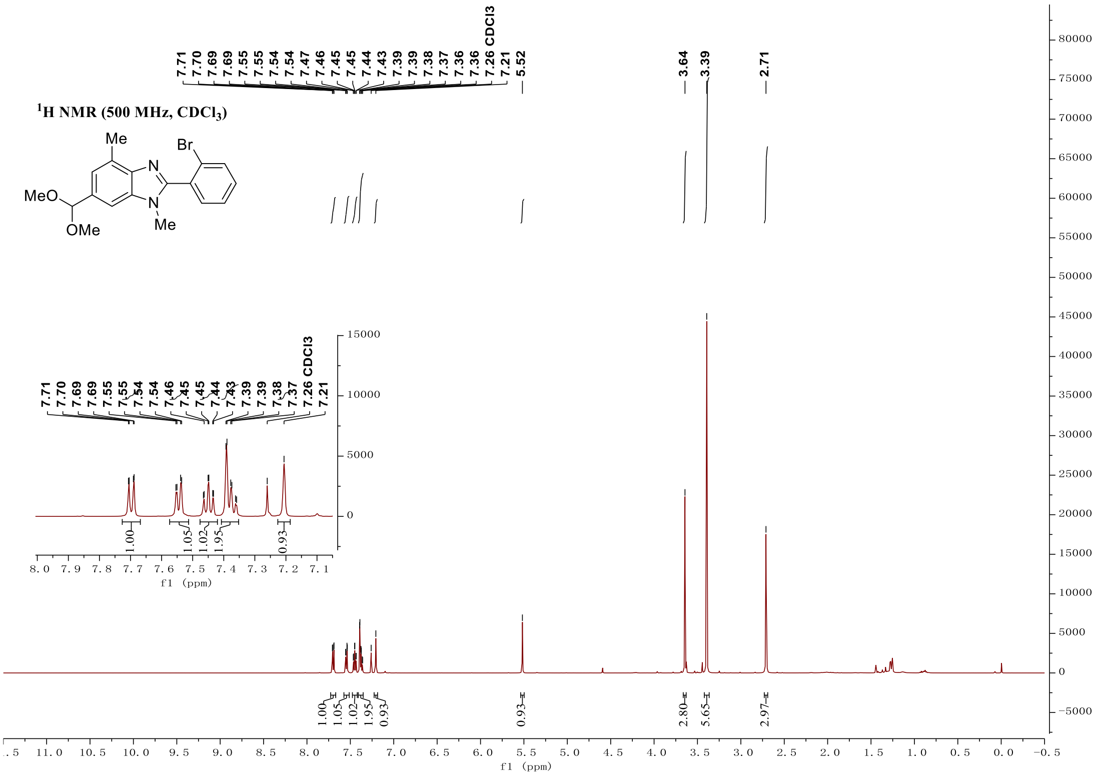

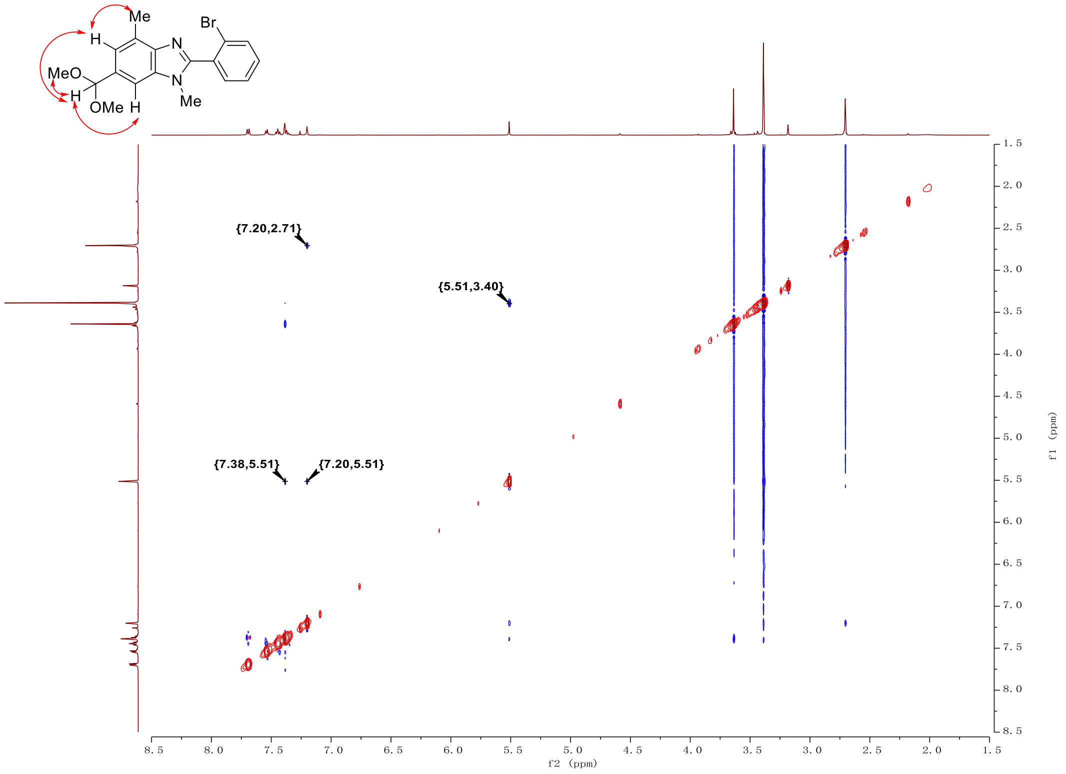

Supplementary Figure 204. ^{1}H NMR and 2D NOESY spectra of compound 65.

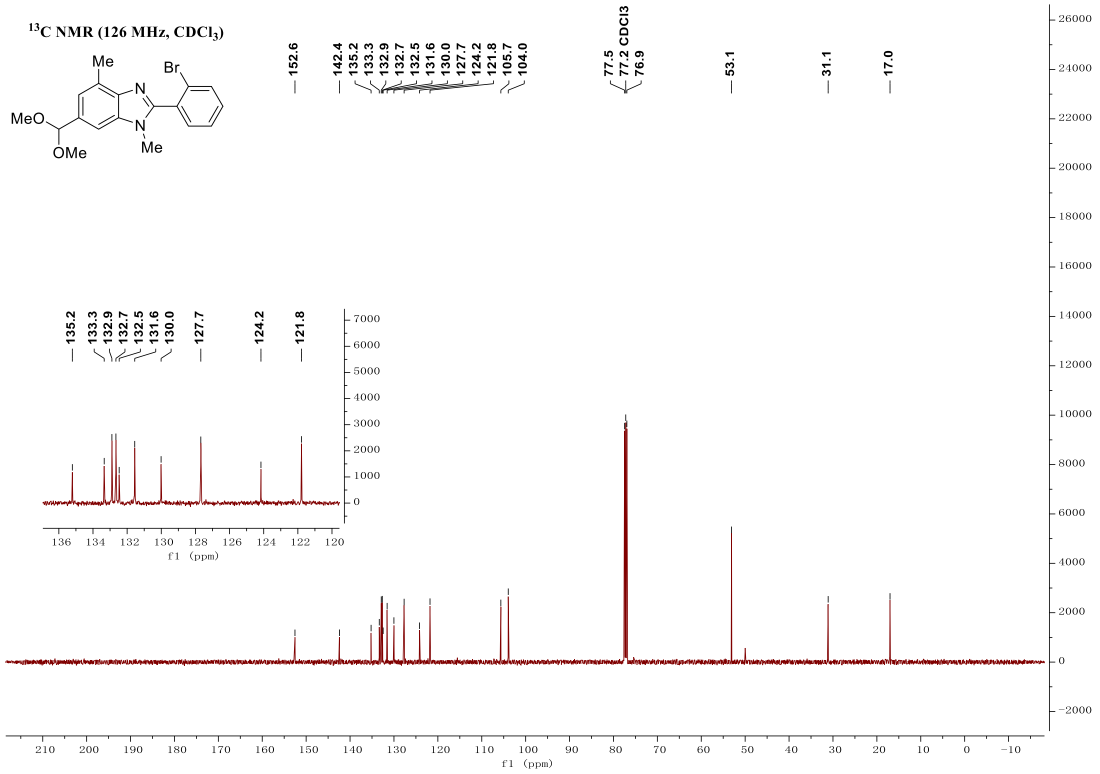

Supplementary Figure 205. ^{13}C NMR spectrum of compound 65.

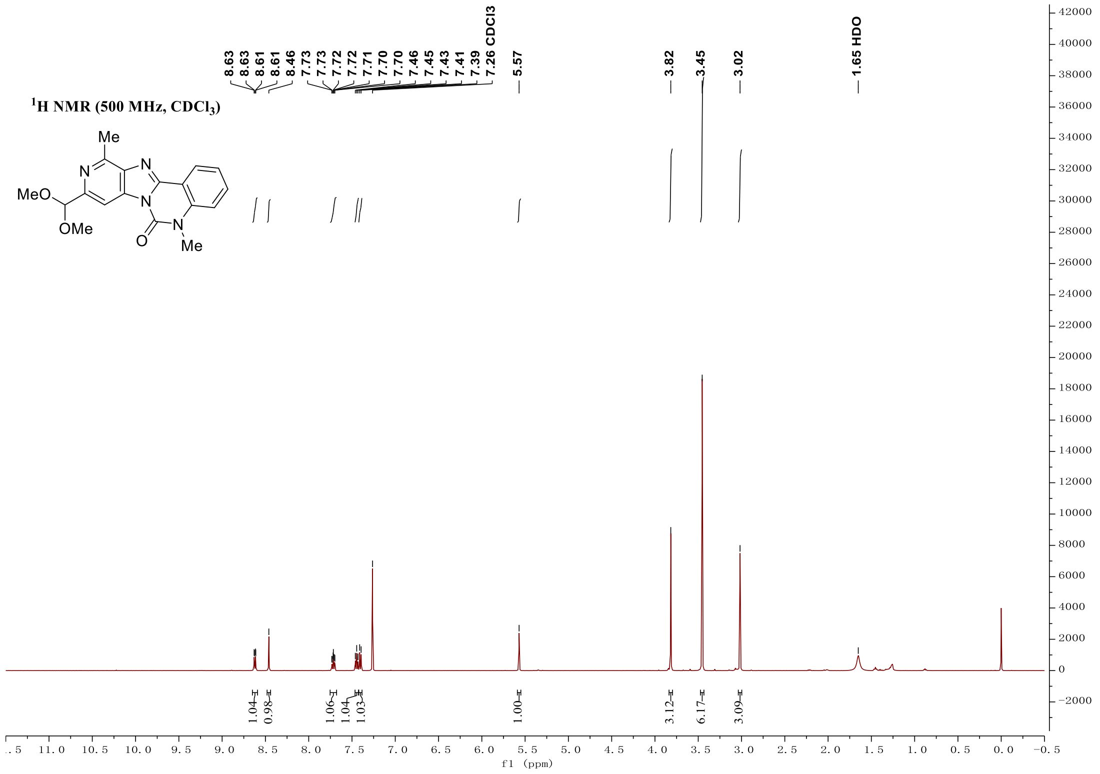

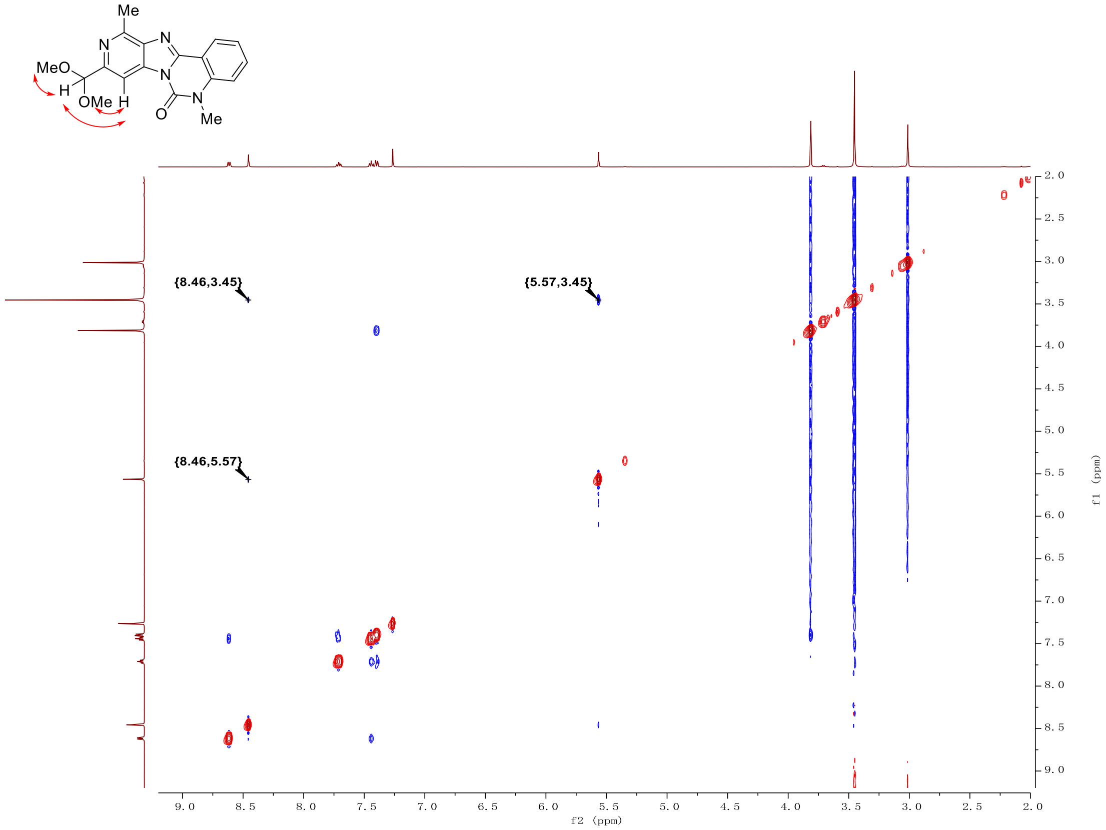

Supplementary Figure 206. ^{1}H NMR and 2D NOESY spectra of compound 66.

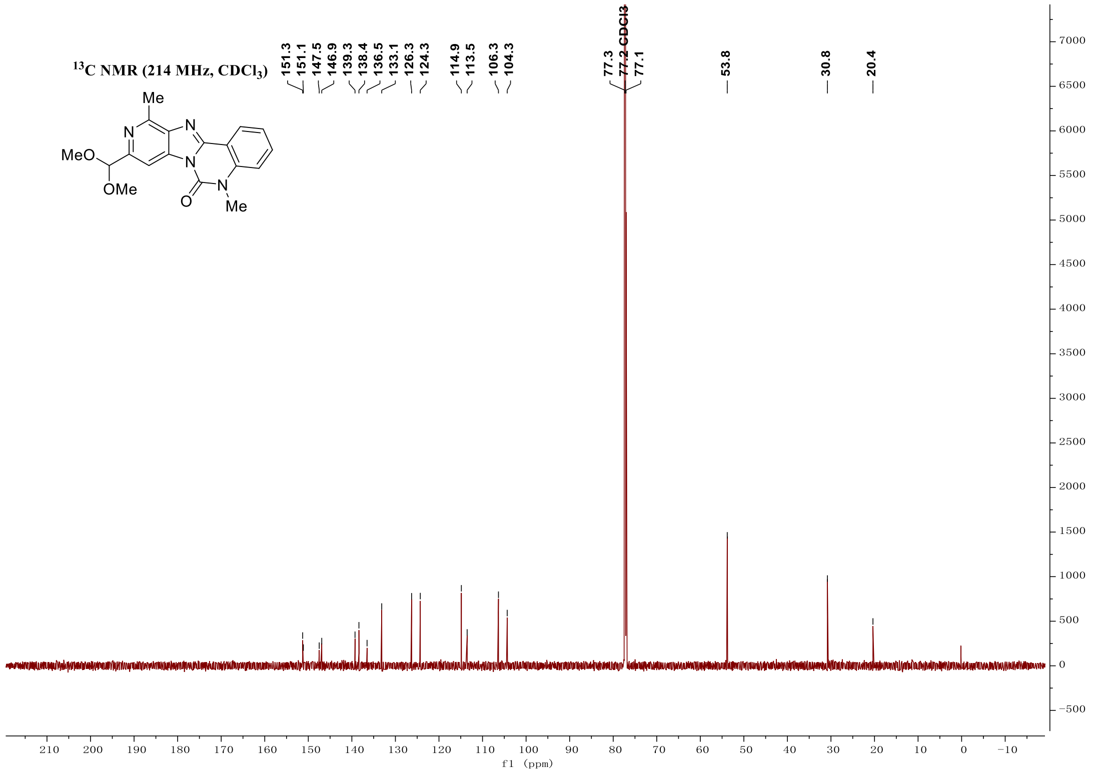

Supplementary Figure 207. ^{13}C NMR spectrum of compound 66.

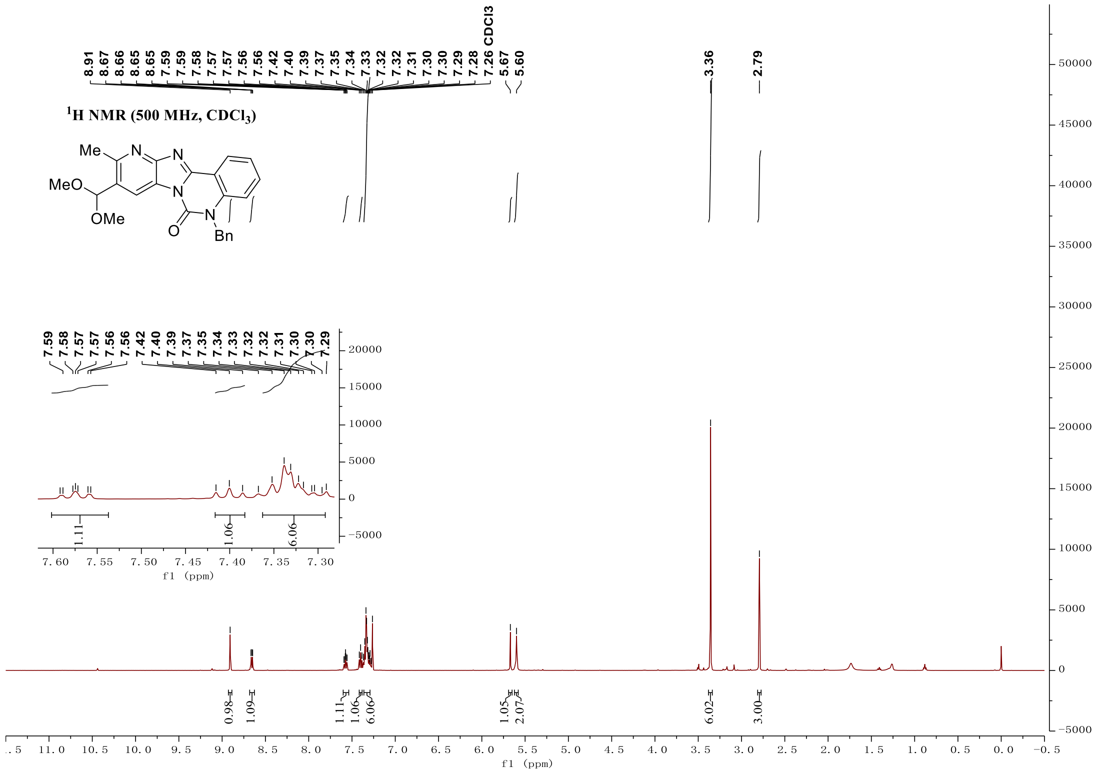

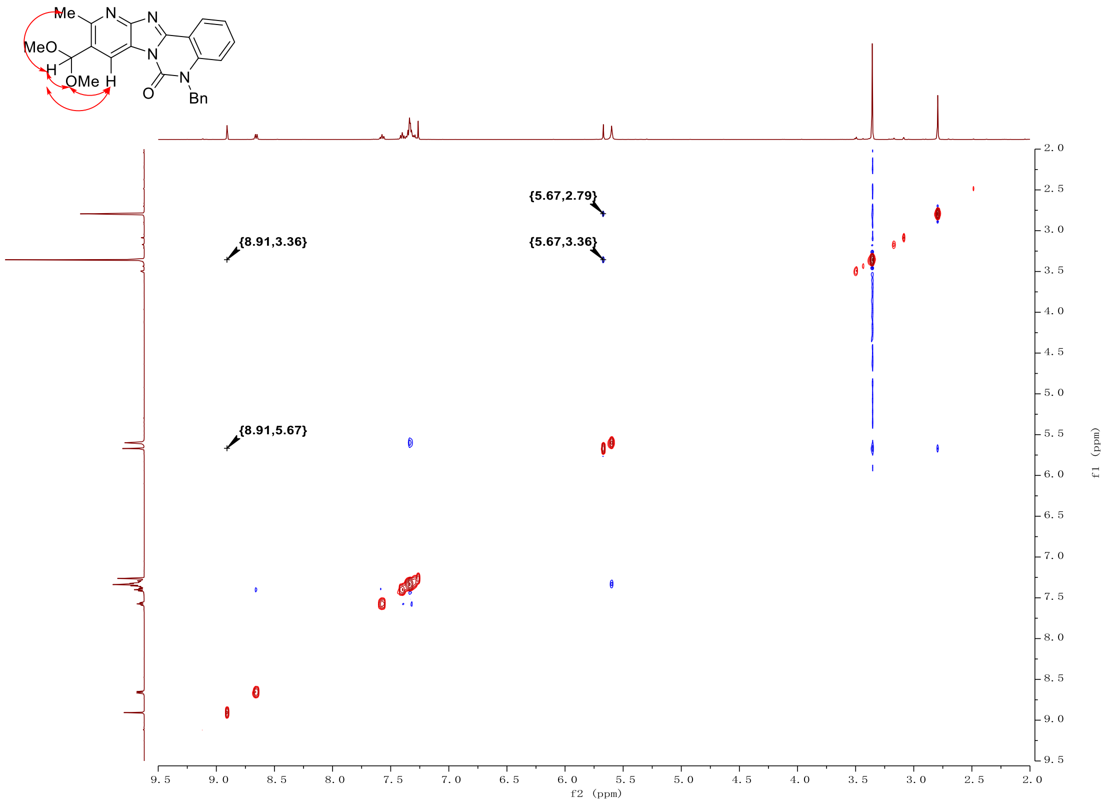

Supplementary Figure 208. ^{1}H NMR and 2D NOESY spectra of compound 67.

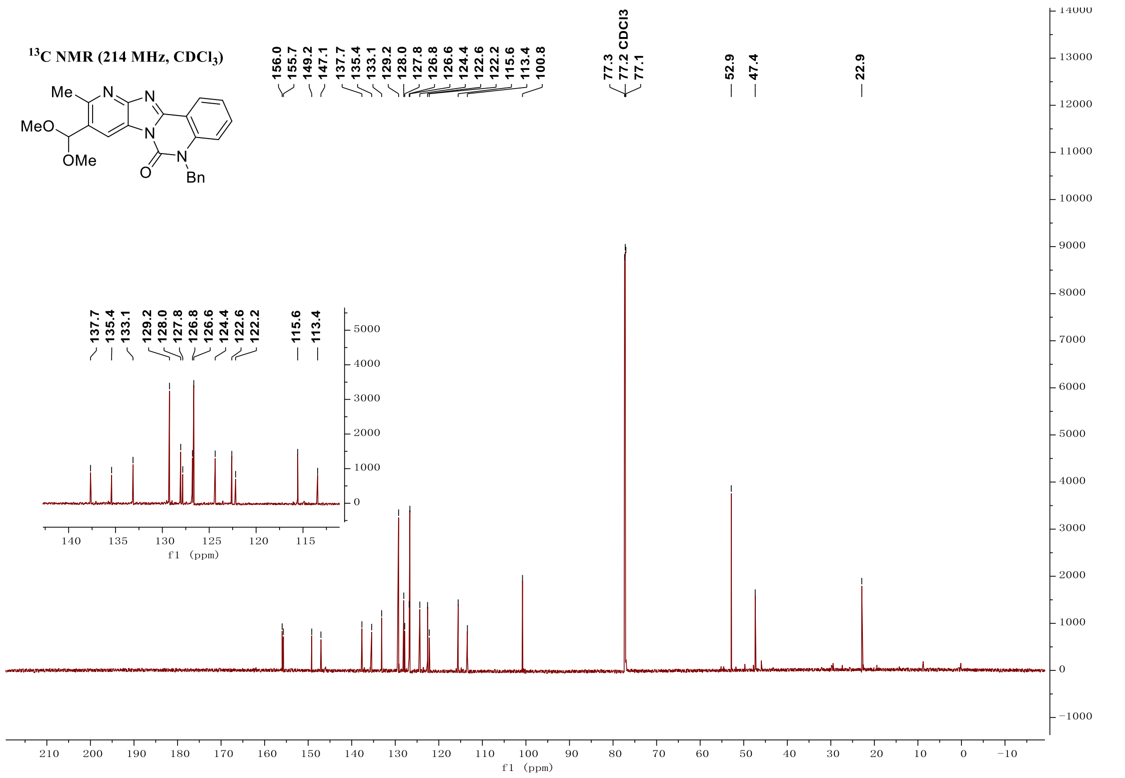

Supplementary Figure 209. ^{13}C **NMR spectrum of compound 67.**

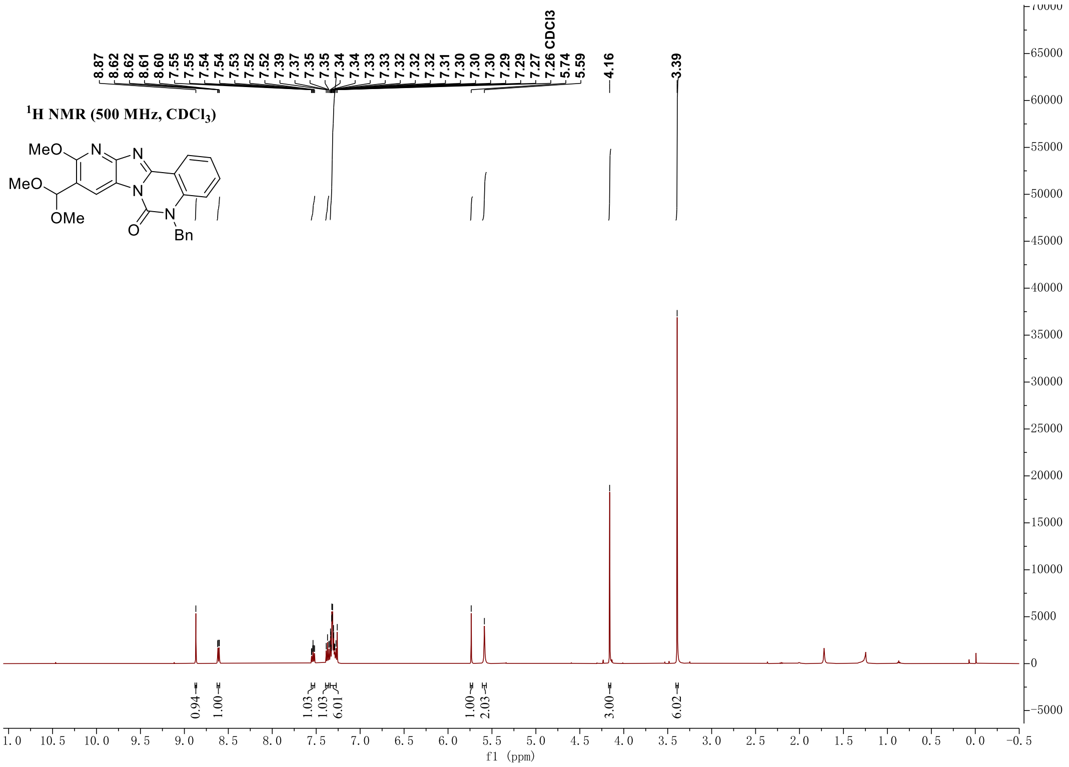

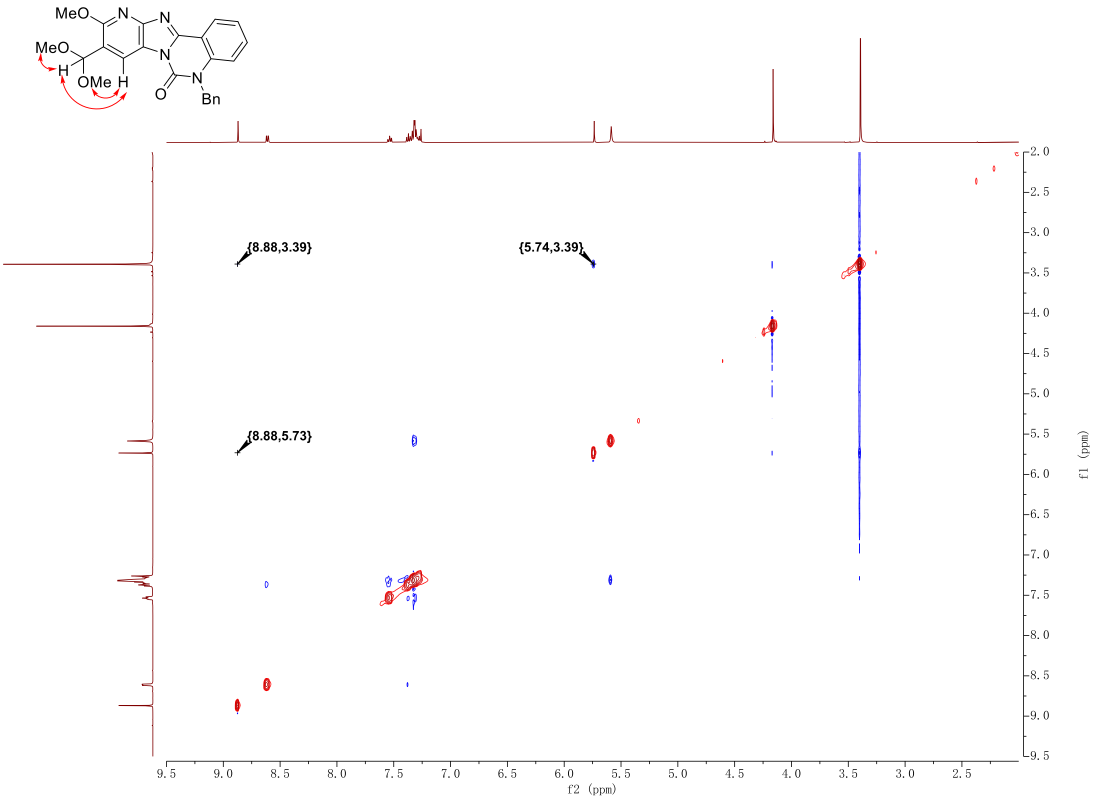

Supplementary Figure 210. ^{1}H NMR and 2D NOESY spectra of compound 68.

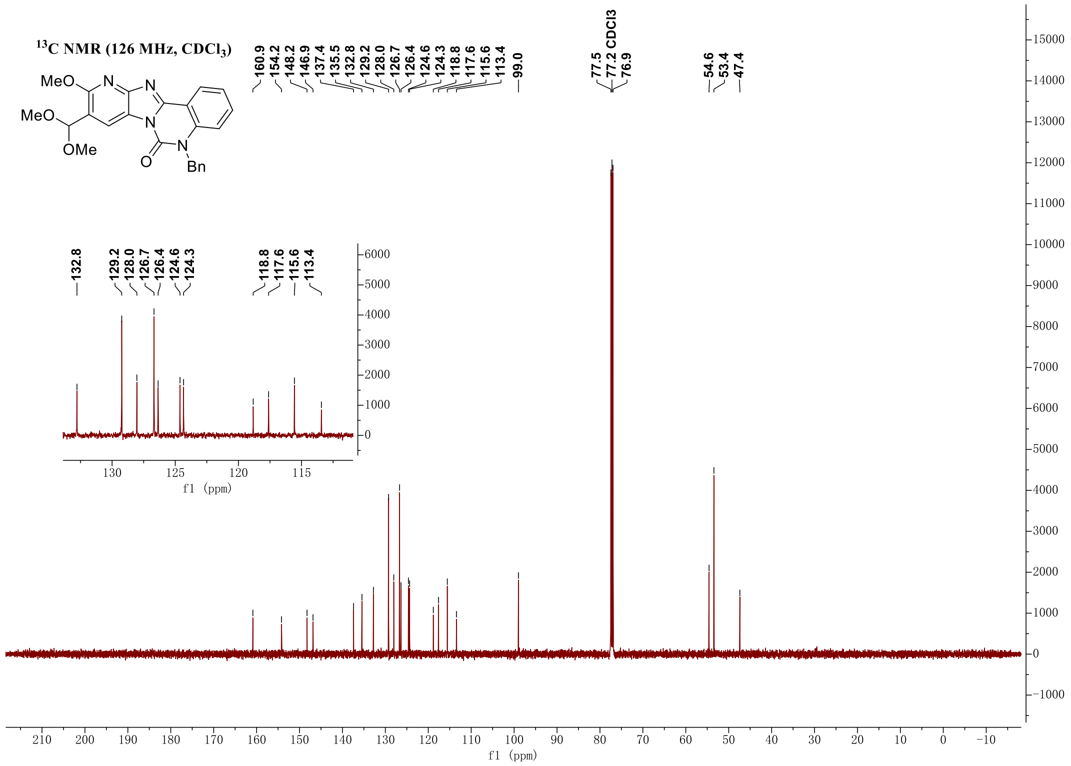

Supplementary Figure 211. ^{13}C NMR spectrum of compound 68.

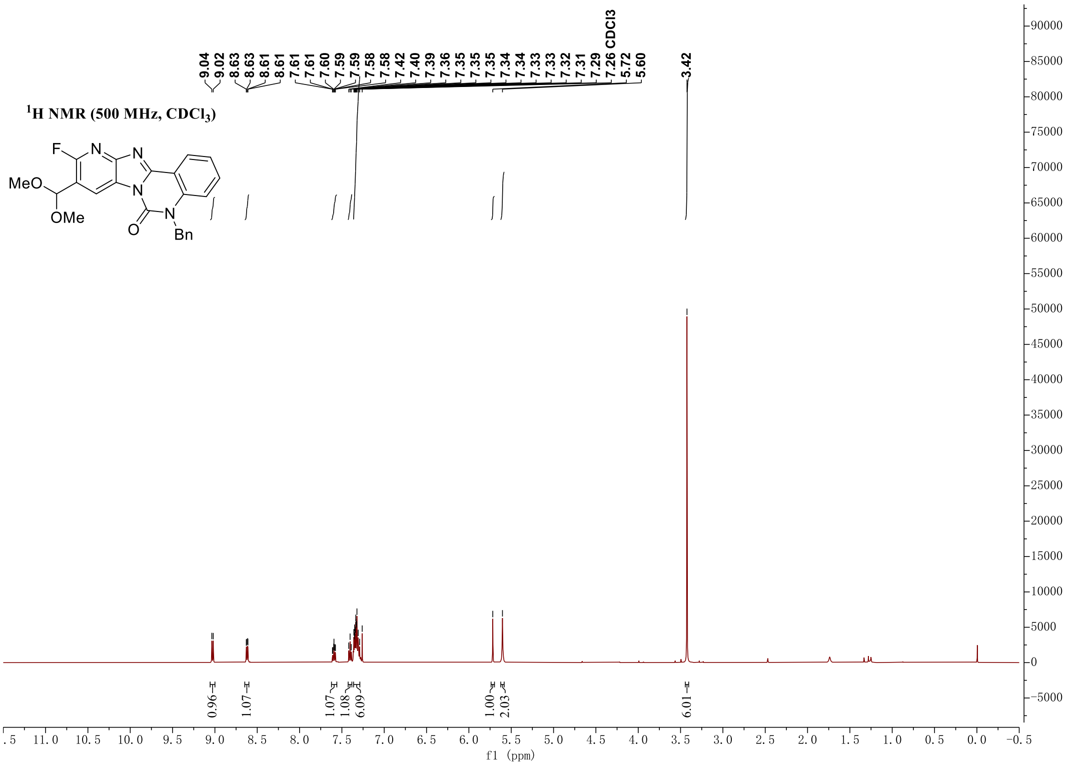

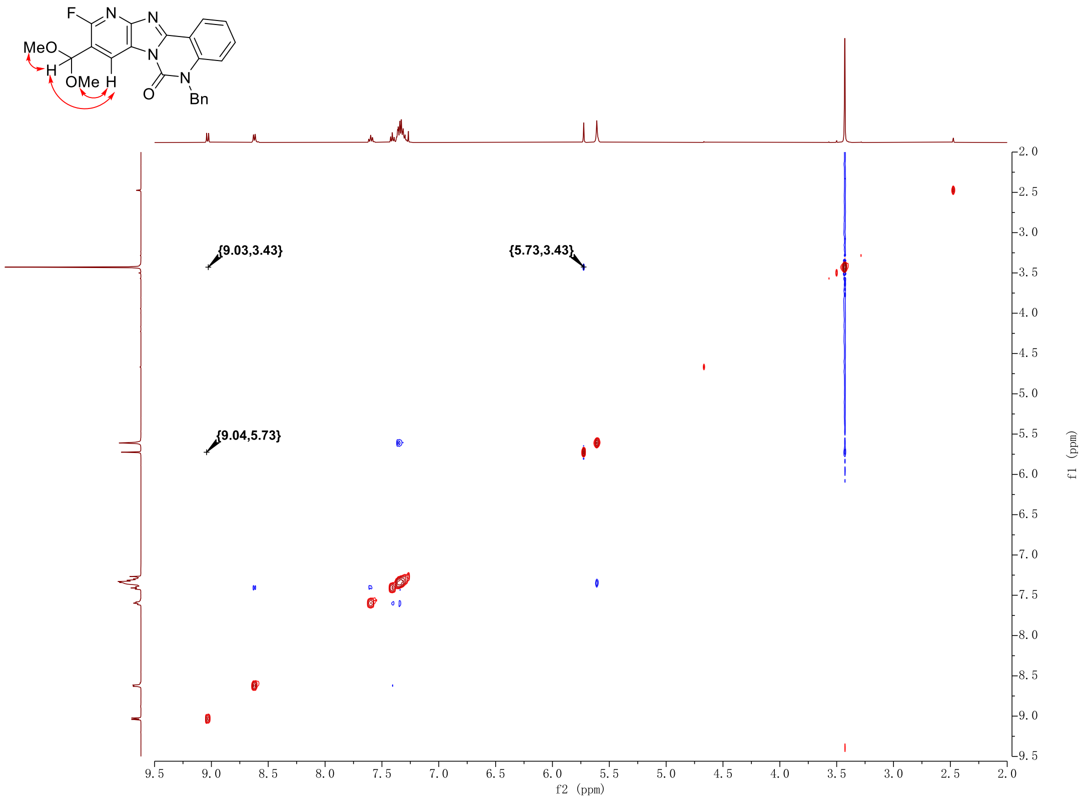

Supplementary Figure 212. ¹H NMR and 2D NOESY spectra of compound 69.

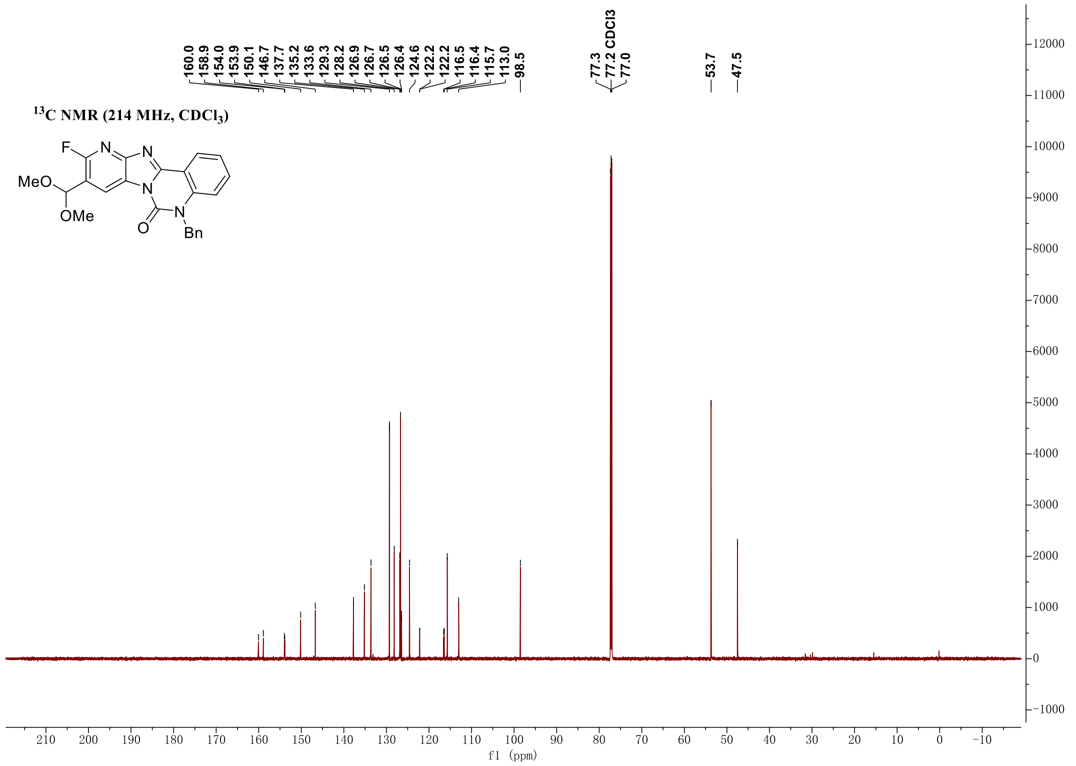

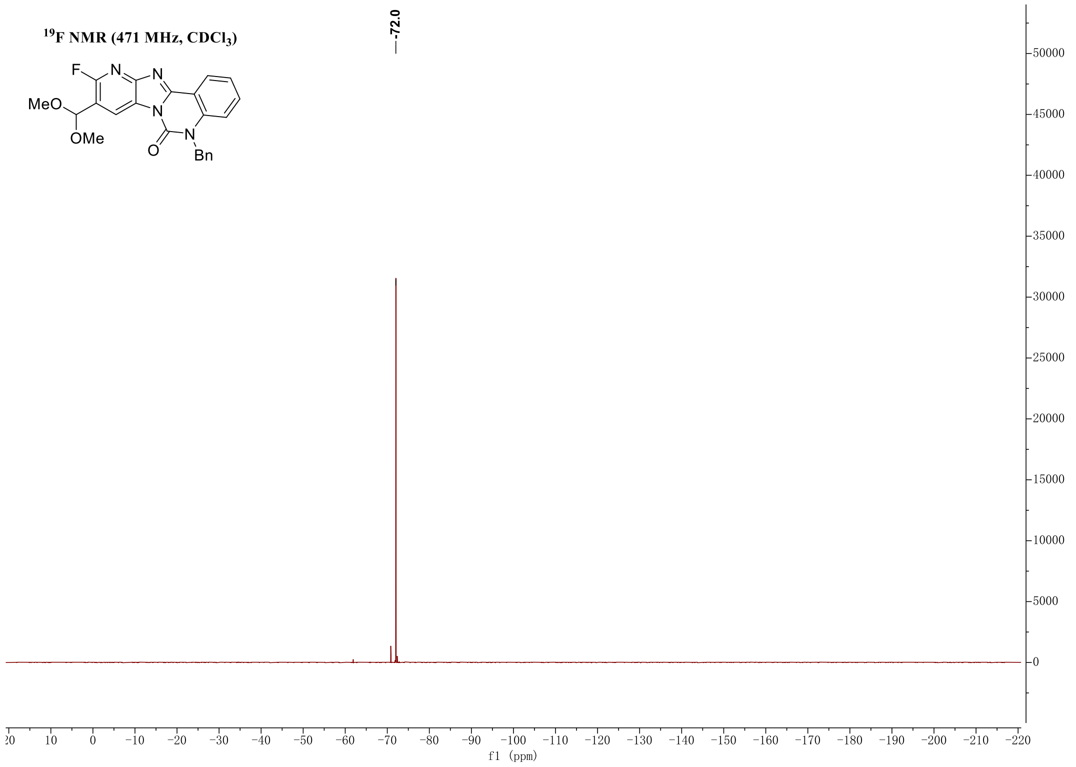

Supplementary Figure 213. ^{13}C **NMR and** ^{19}F **NMR spectra of compound 69.**

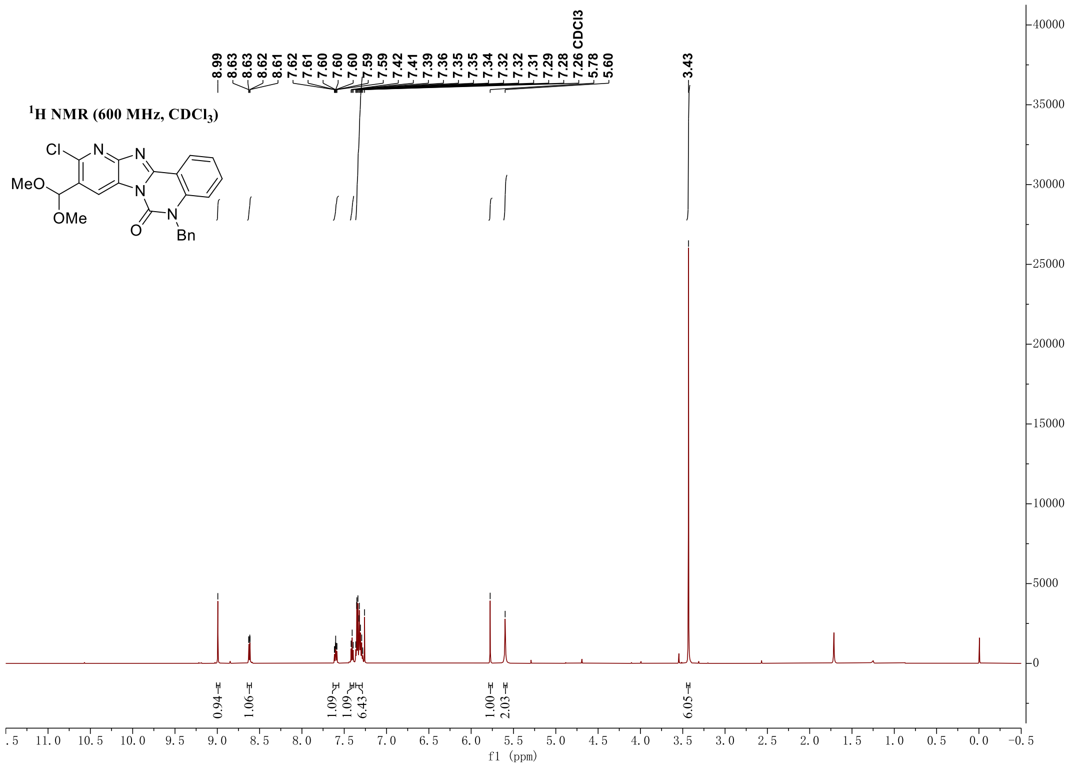

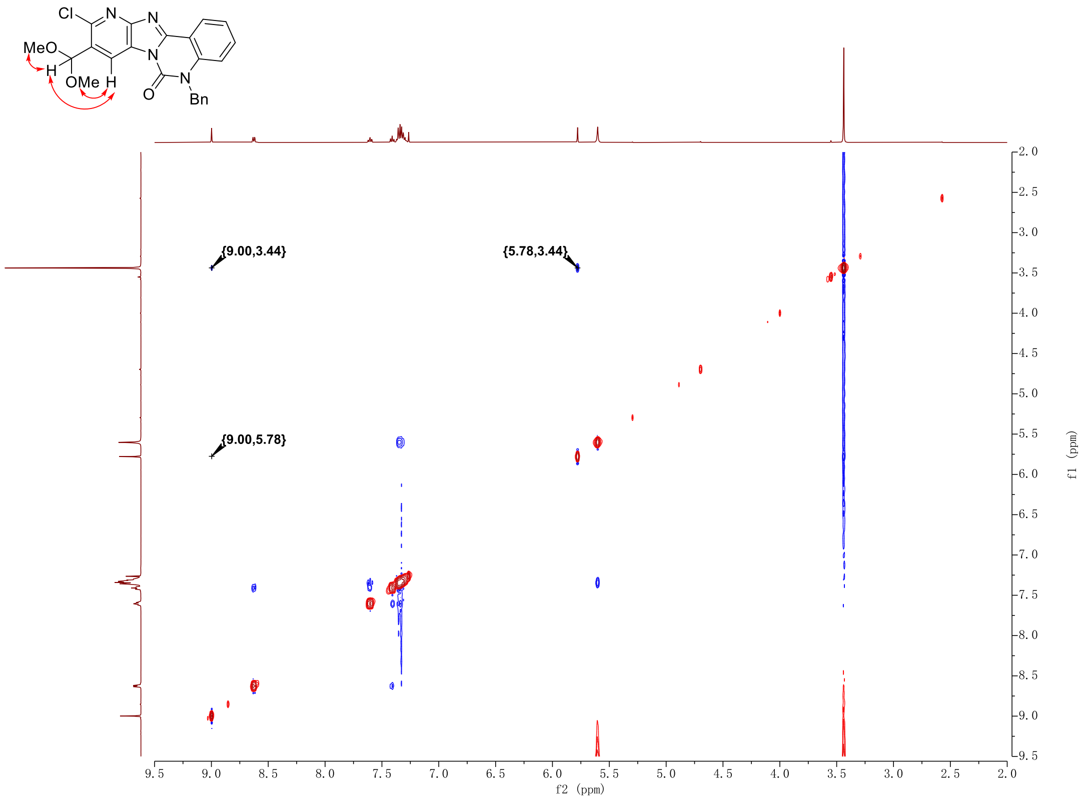

Supplementary Figure 214. ^{1}H NMR and 2D NOESY spectra of compound 70.

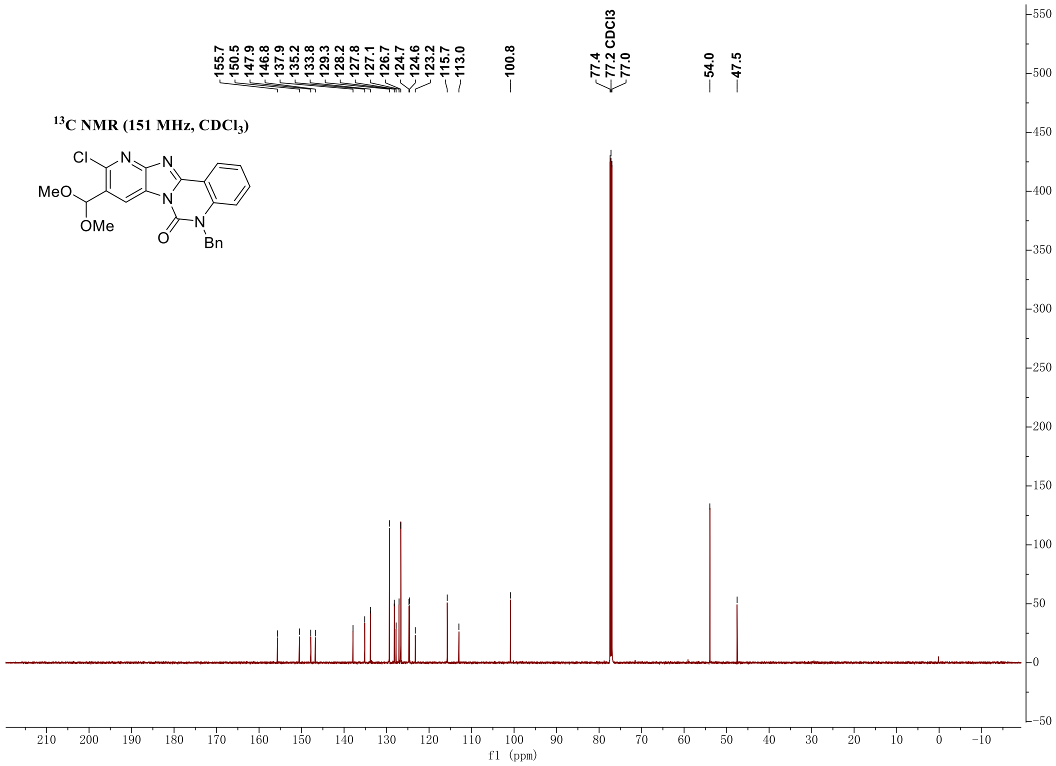

Supplementary Figure 215. ^{13}C NMR spectrum of compound 70.

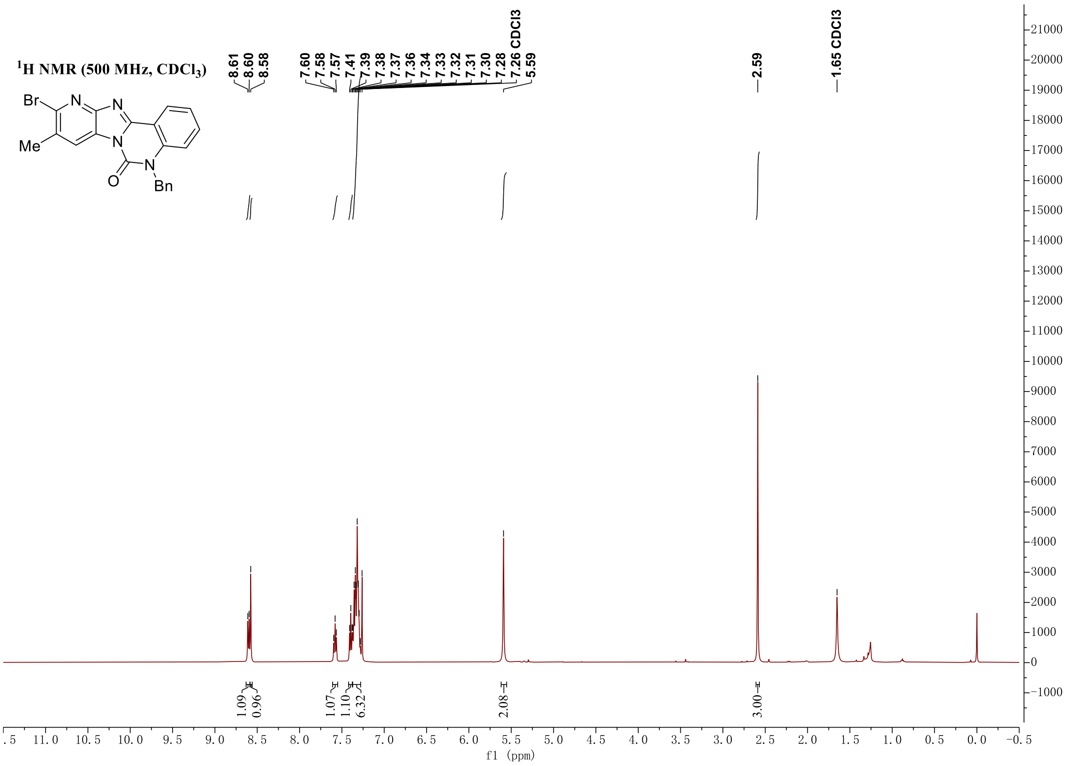

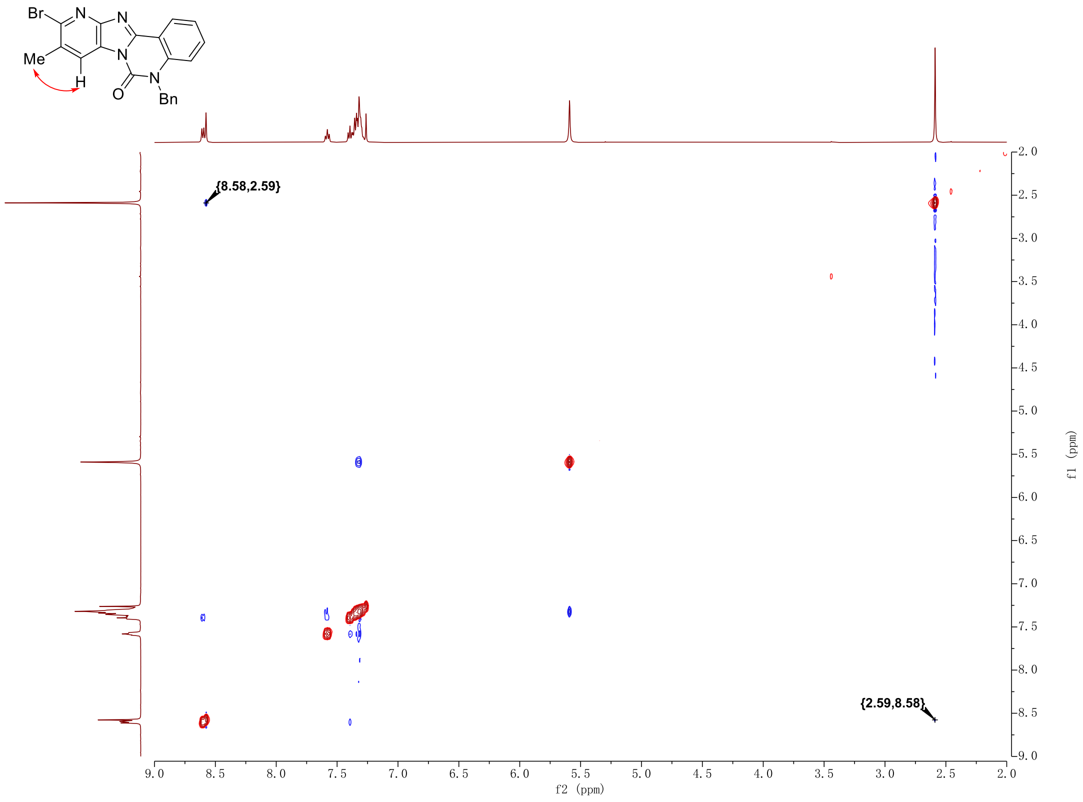

Supplementary Figure 216. ^{1}H NMR and 2D NOESY spectra of compound 71.

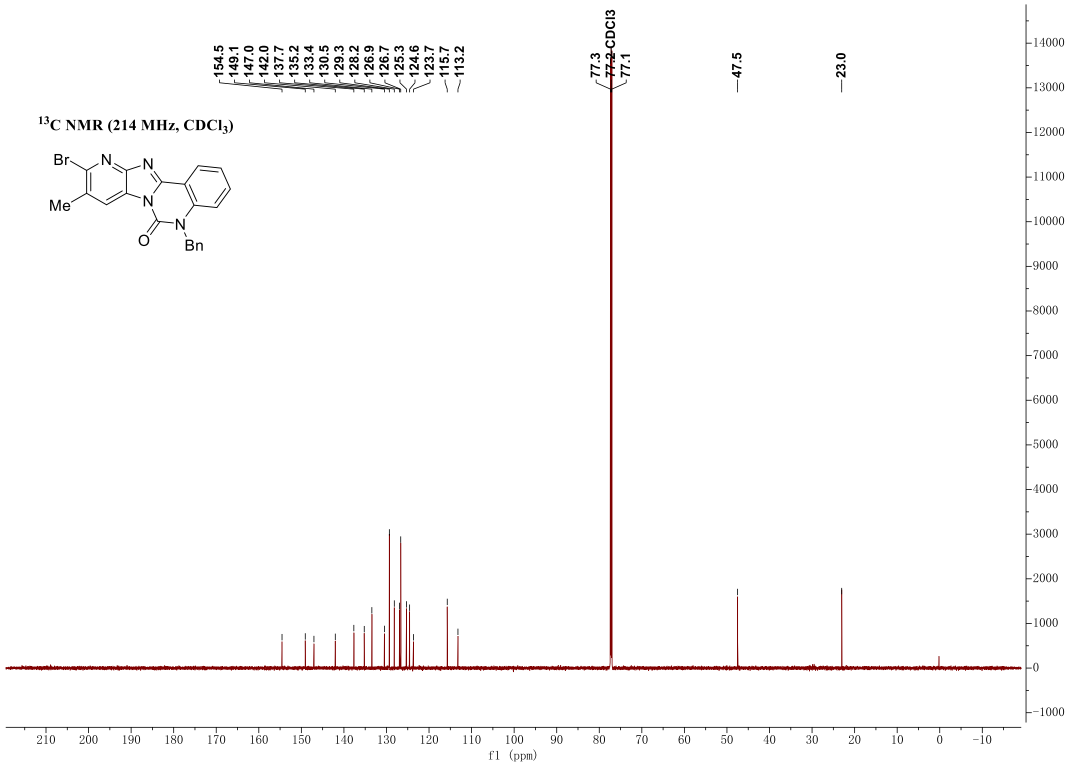

Supplementary Figure 217. ^{13}C NMR spectrum of compound 71.

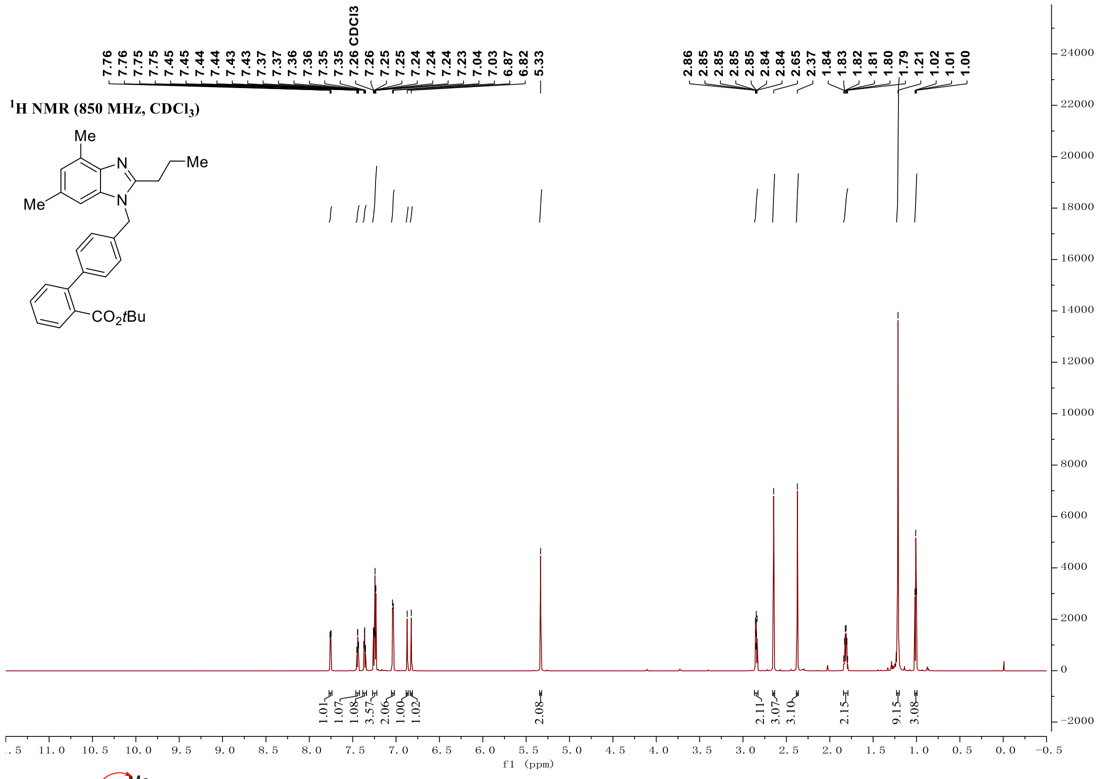

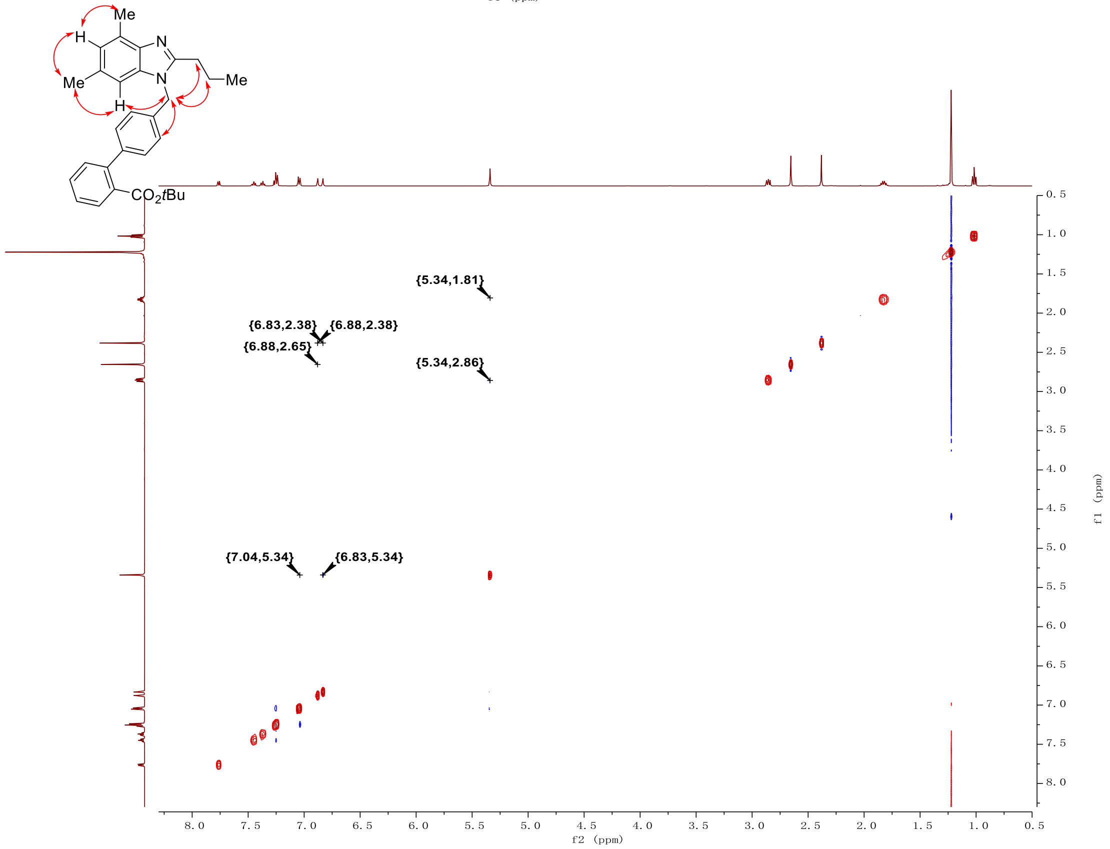

Supplementary Figure 218. ^{1}H NMR and 2D NOESY spectra of compound 75.

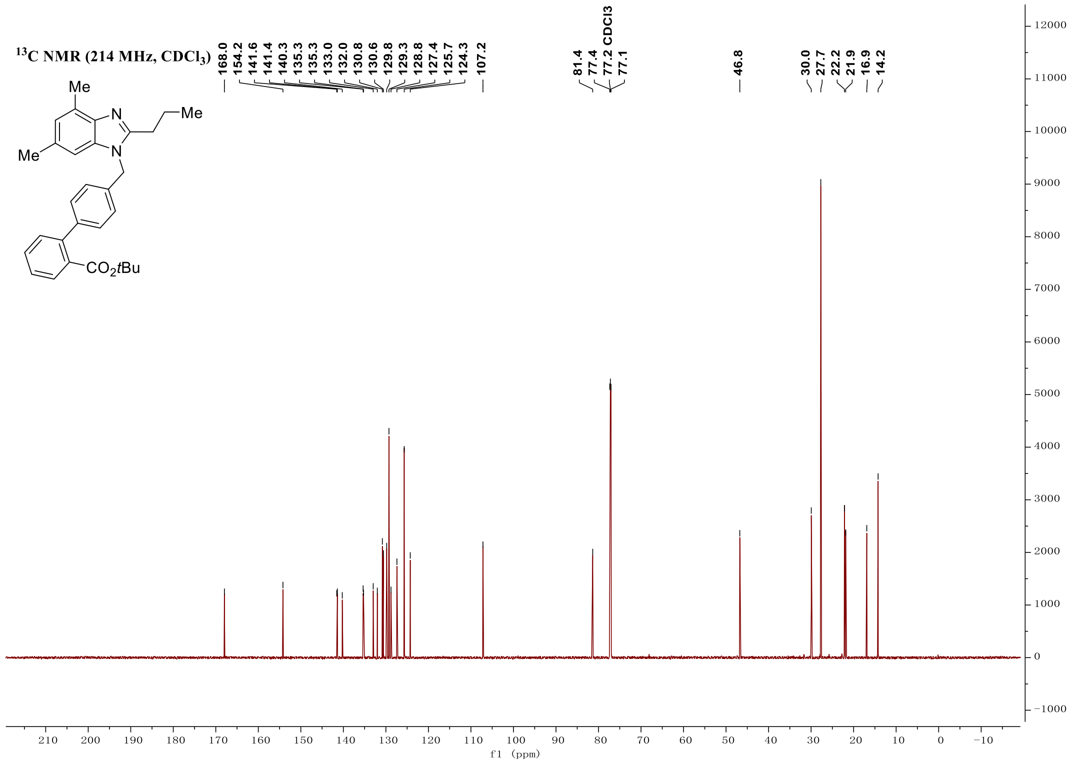

Supplementary Figure 219. ^{13}**C NMR spectrum of compound 75.**

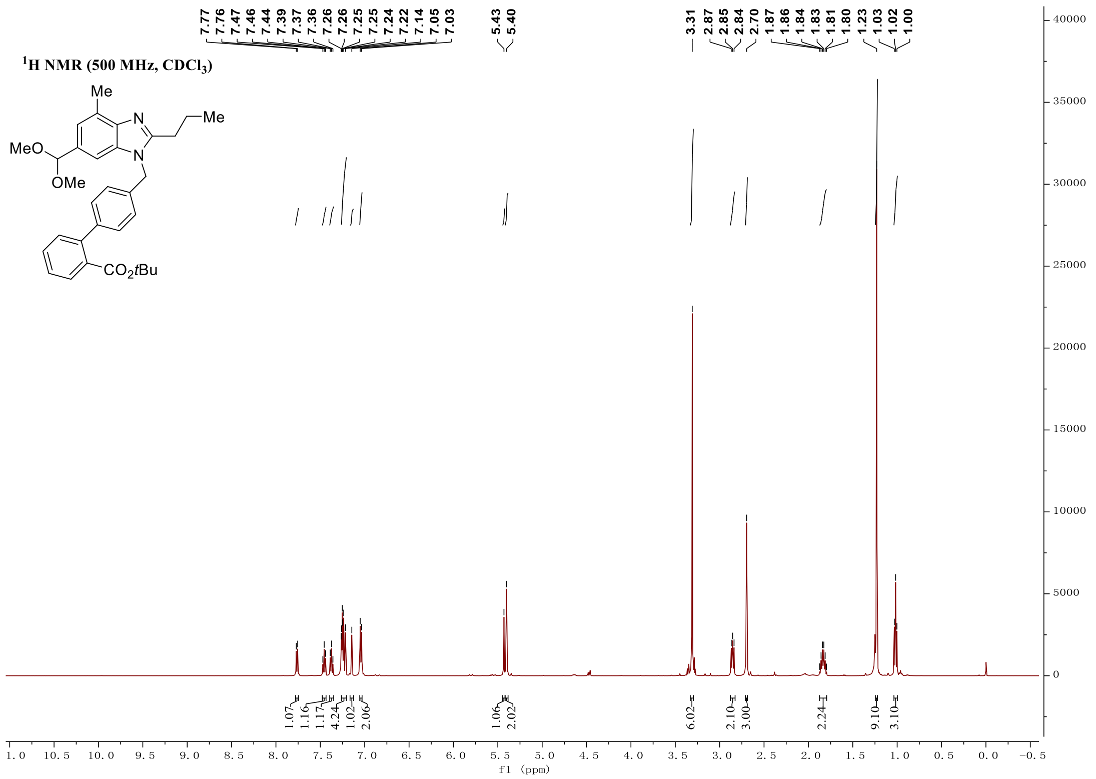

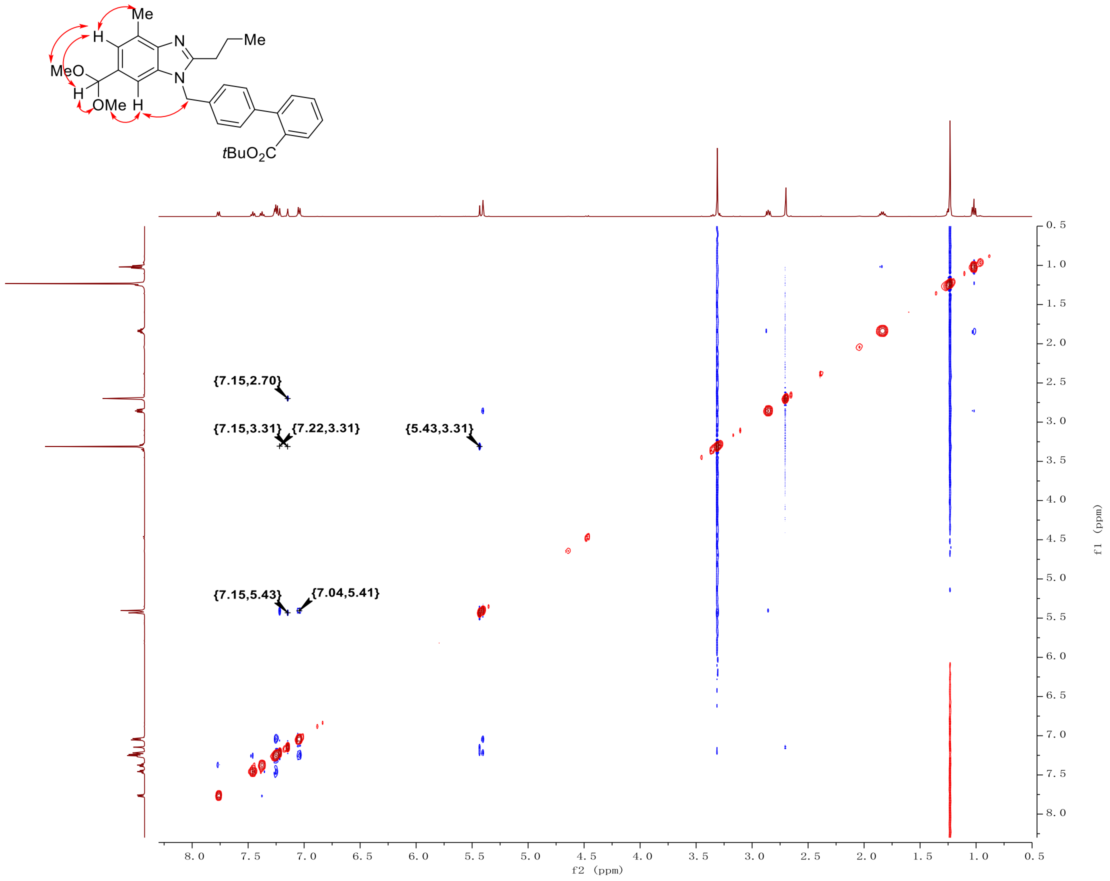

Supplementary Figure 220. ^{1}H NMR and 2D NOESY spectra of compound 76.

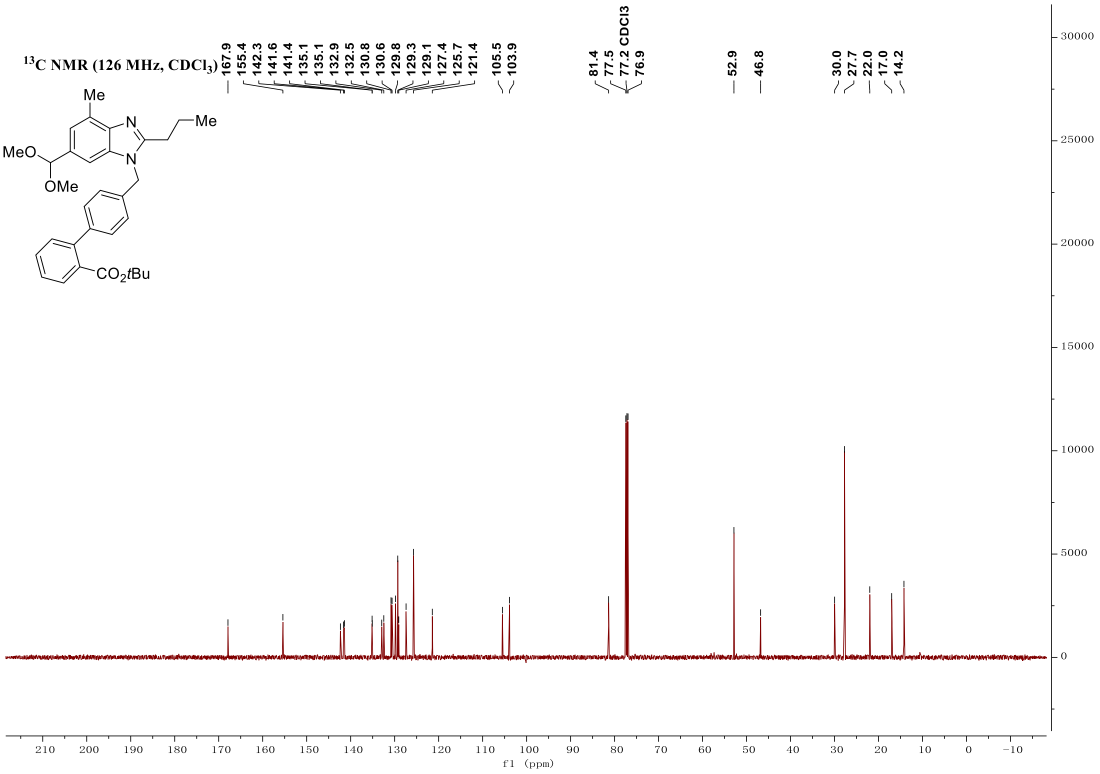

Supplementary Figure 221. ^{13}C NMR spectrum of compound 76.

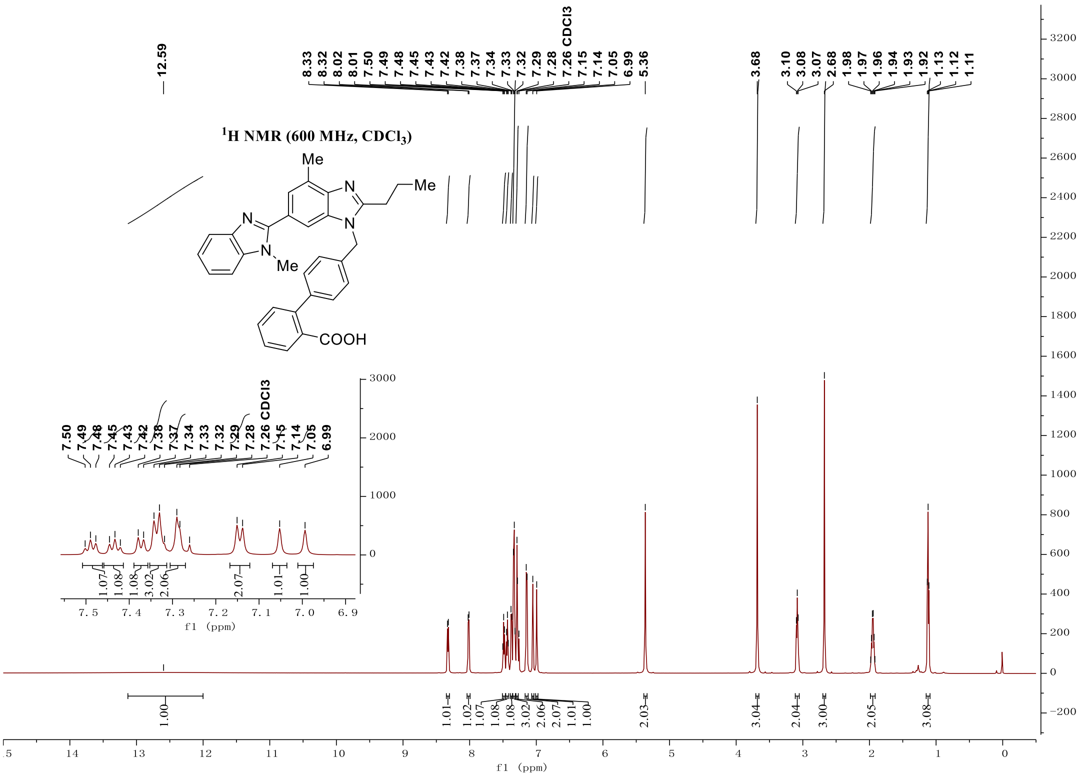

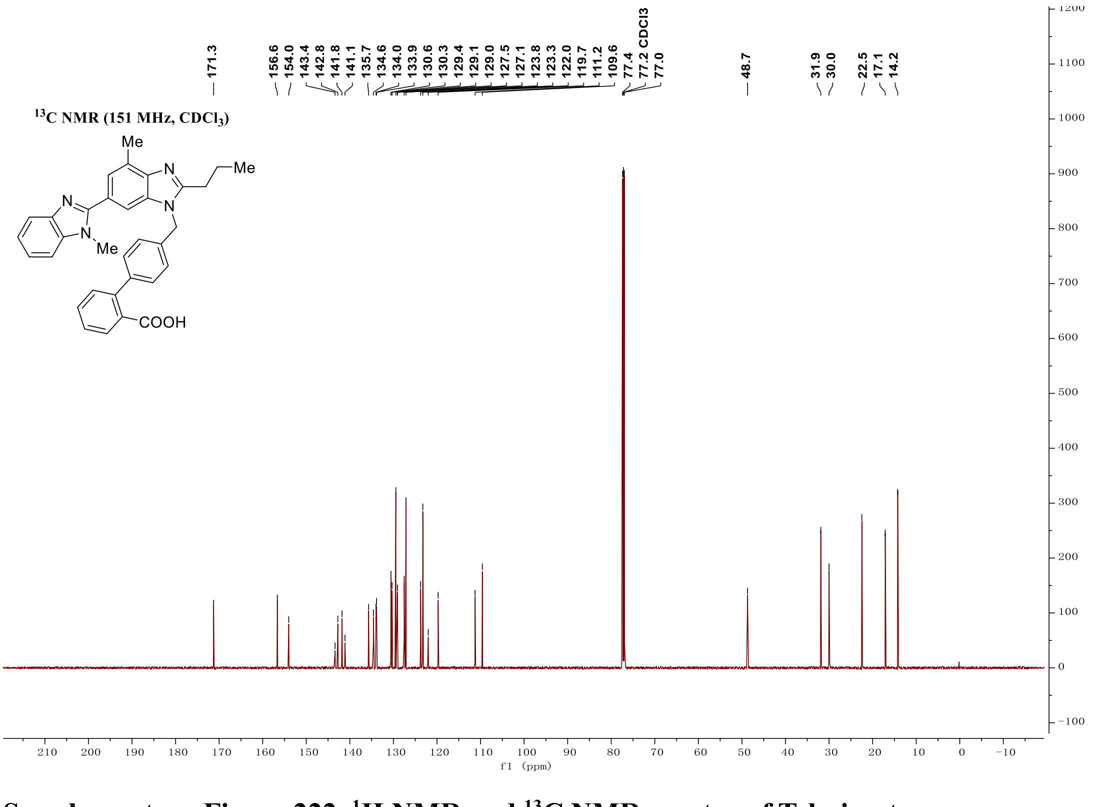

Supplementary Figure 222. ^{1}H NMR and ^{13}C NMR spectra of Telmisartan.

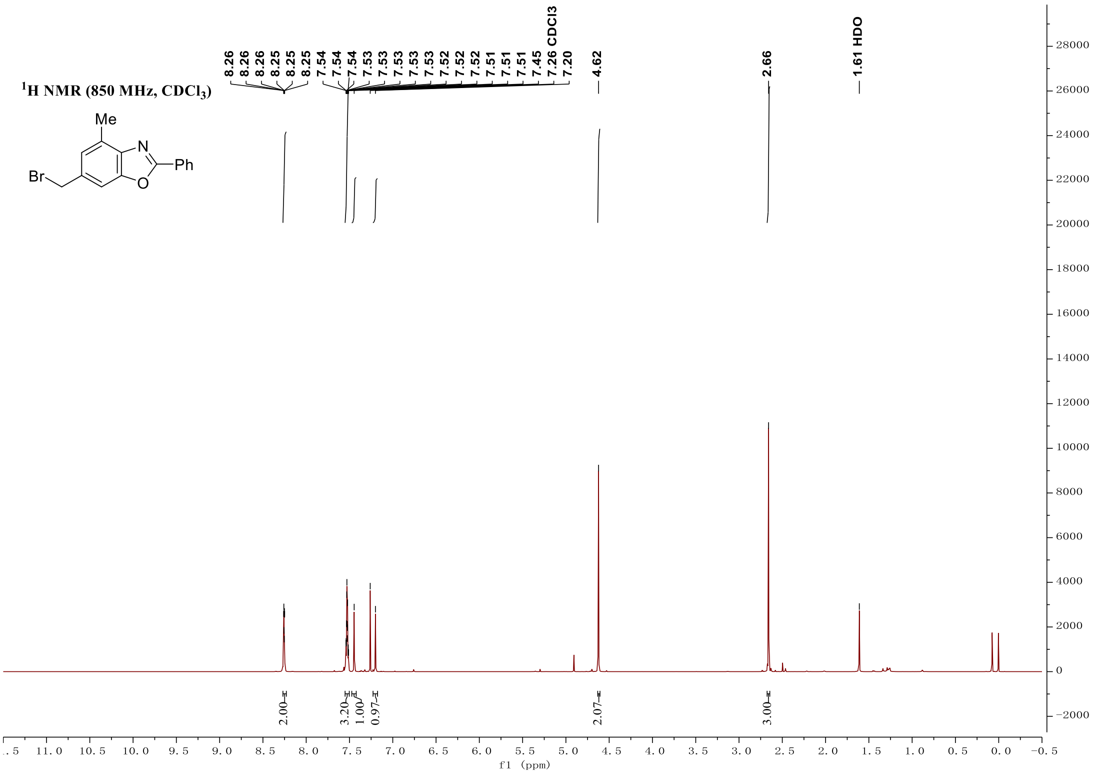

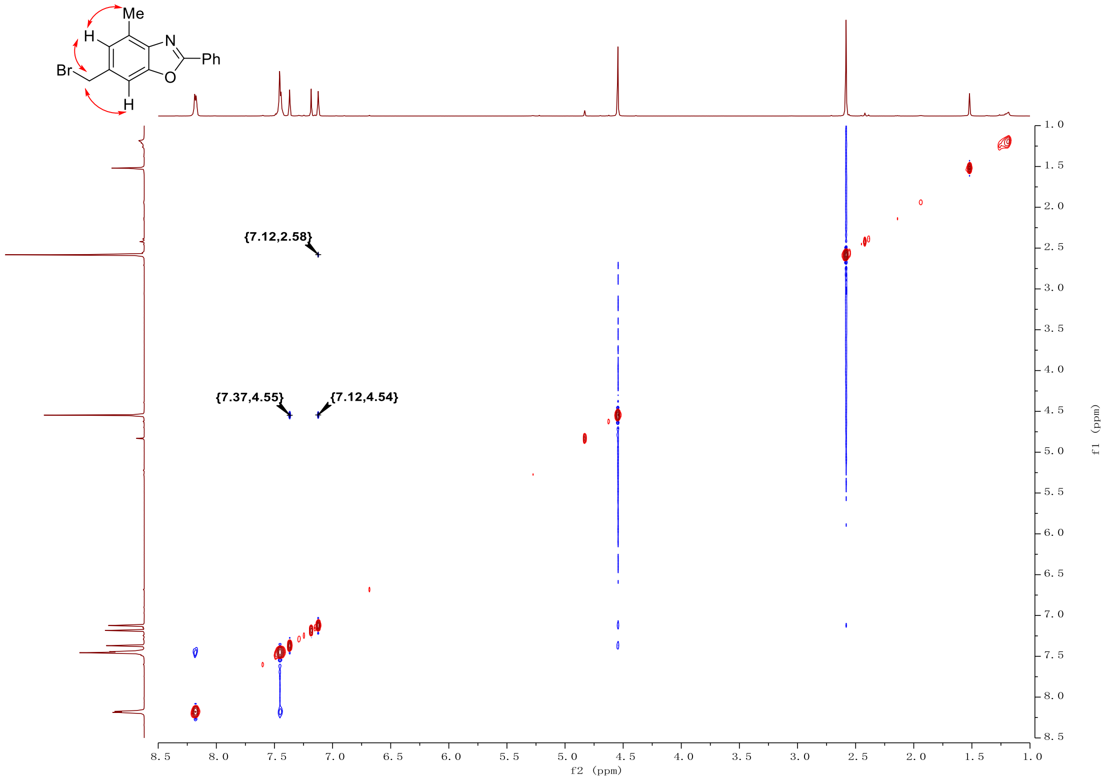

Supplementary Figure 223. ^{1}H NMR and 2D NOESY spectra of compound 82.

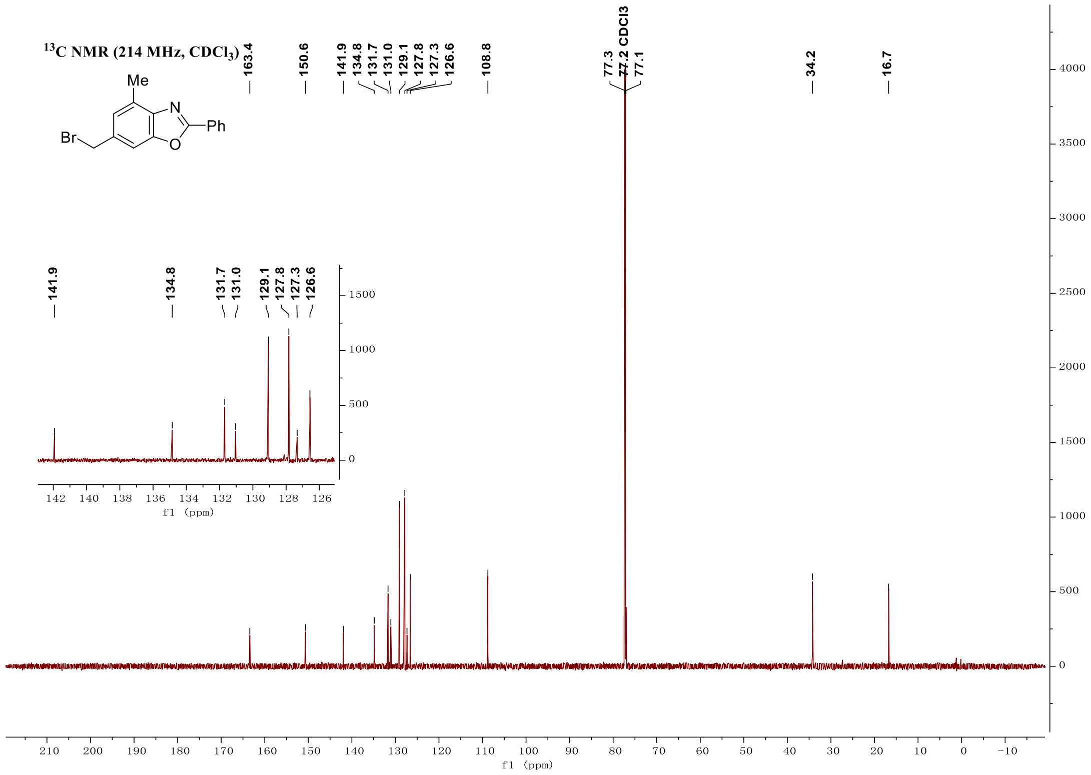

Supplementary Figure 224. ^{13}C NMR spectrum of compound 82.

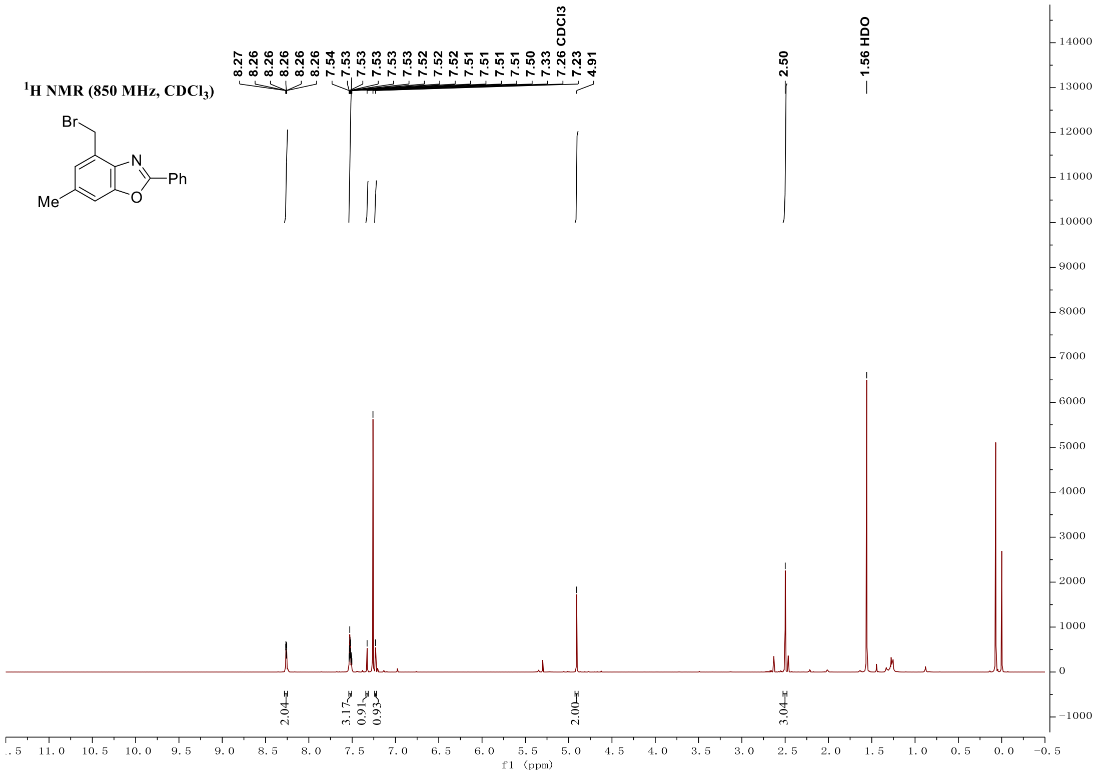

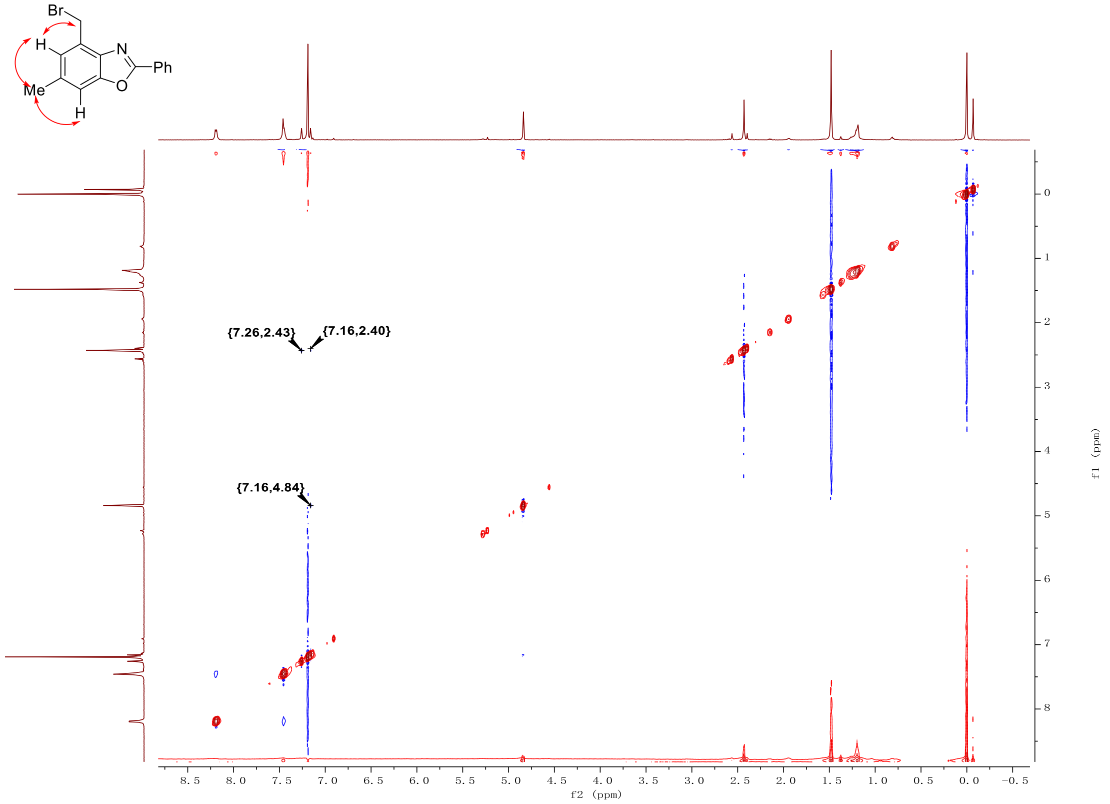

Supplementary Figure 225. ^{1}H NMR and 2D NOESY spectra of compound 83.

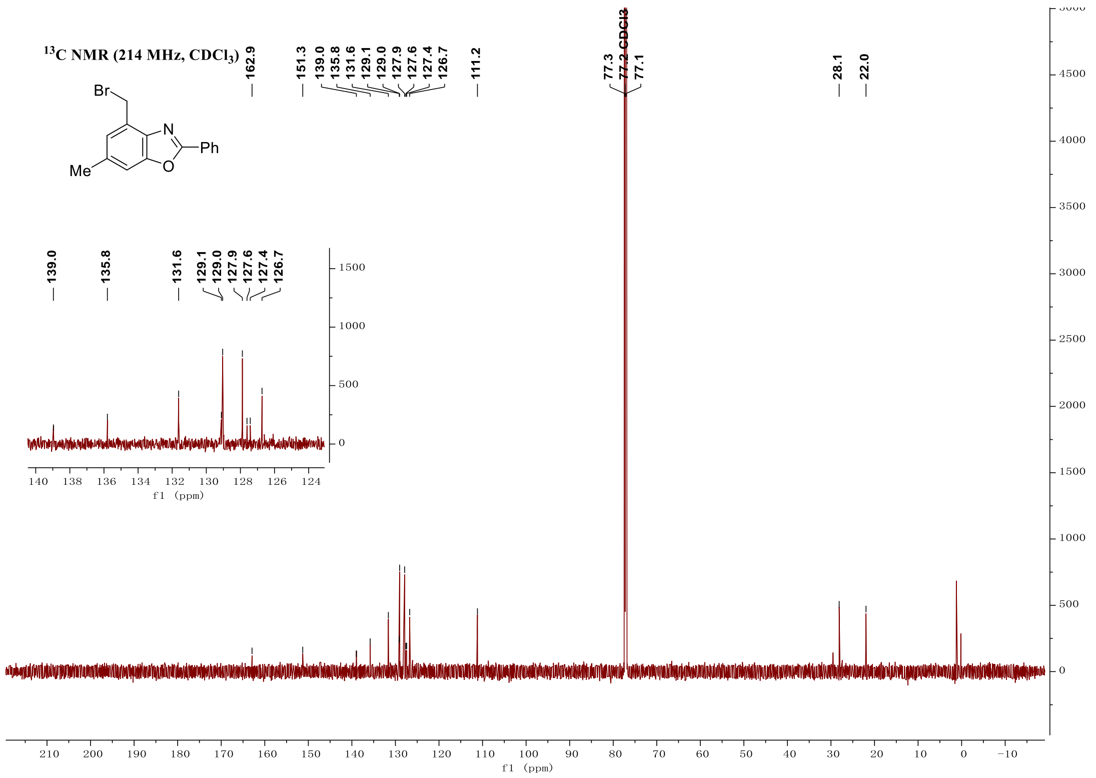

Supplementary Figure 226. ^{13}C **NMR spectrum of compound 83.**

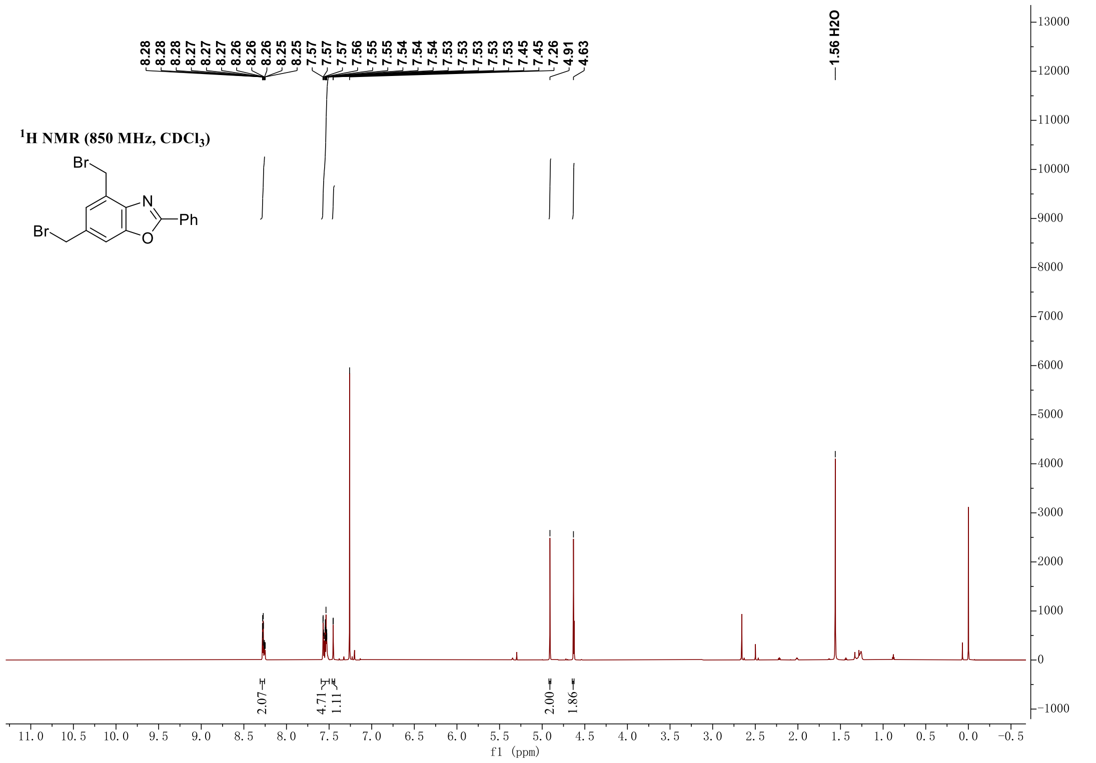

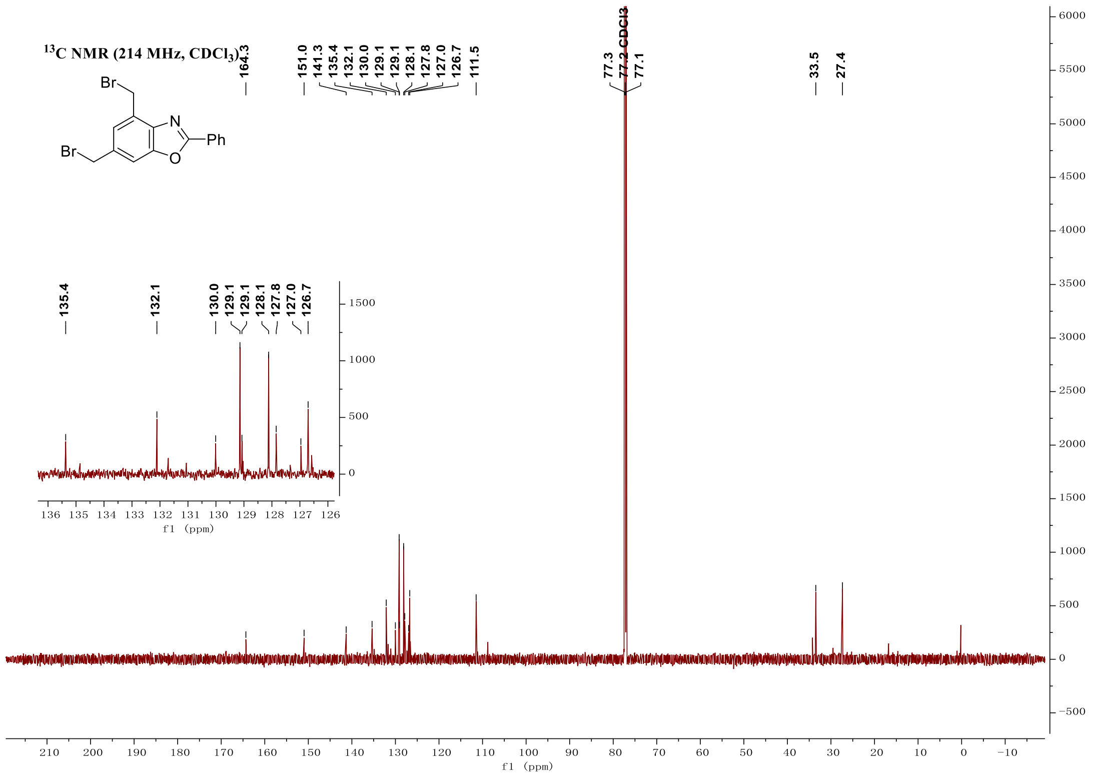

Supplementary Figure 227. ^{1}H NMR and ^{13}C NMR spectra of compound 84.

Supplementary References

1. Mei, C. & Lu, W. Palladium(II)-Catalyzed Oxidative Homo- and Cross-Coupling of Aryl ortho-sp(2) C-H Bonds of Anilides at Room Temperature. *J. Org. Chem.* **83**, 4812-4823 (2018).
2. Morozov, P.G., Kurbatov, S.V., Dolgushin, F.M., Antipin, M.Y. & Olekhnovich, L.P.J.R.C.B. Synthesis and structures of spiro-σ-complexes based on 2-(2-benzylaminophenyl)-5,6-dimethylbenzimidazole. *Russ. Chem. Bul.* **53**, 2075-2079 (2004).
3. Lam, T.*, et al.* Structure-based design of new dihydrofolate reductase antibacterial agents: 7-(benzimidazol-1-yl)-2,4-diaminoquinazolines. *J. Med. Chem.* **57**, 651-668 (2014).
4. Tang, Z., Mai, S., Zhou, Y. & Song, Q. Divergent synthesis of α-aryl ketones/esters via rhodium-catalyzed selective deesterification and decarbonylation of diazo compounds. *Org. Chem. Front.* **5**, 2583-2587 (2018).
5. Alla, S.K., Sadhu, P. & Punniyamurthy, T. Organocatalytic Syntheses of Benzoxazoles and Benzothiazoles using Aryl Iodide and Oxone via C-H Functionalization and C-O/S Bond Formation. *J. Org. Chem.* **79**, 7502-7511 (2014).
6. Sarode, S.A., Bhojane, J.M. & Nagarkar, J.M. An efficient magnetic copper ferrite nanoparticle: for one pot synthesis of 2-substituted benzoxazole via redox reactions. *Tetrahedron Lett.* **56**, 206-210 (2015).
7. Che, X.Z., Jiang, J.J., Xiao, F.H., Huang, H.W. & Deng, G.J. Assembly of 2-Arylbenzothiazoles through Three-Component Oxidative Annulation under Transition-Metal-Free Conditions. *Org. Lett.* **19**, 4576-4579 (2017).
8. Guedes de la Cruz, G.*, et al.* Intensified Microwave-Assisted N-Acylation Procedure – Synthesis and Activity Evaluation of TRPC3 Channel Agonists with a 1,3-Dihydro-2H-benzo[d]imidazol-2-one Core. *Synlett.* **28**, 695-700 (2017).
9. Diao, X.Q., Wang, Y.J., Jiang, Y.W. & Ma, D.W. Assembly of Substituted 1H-Benzimidazoles and 1,3-Dihydrobenzimidazol-2-ones via CuI/L-Proline Catalyzed Coupling of Aqueous Ammonia with 2-Iodoacetanilides and 2-Iodophenylcarbamates. *J. Org. Chem.* **74**, 7974-7977 (2009).
10. Xu, F., Long, H., Song, J.S. & Xu, H.C. De Novo Synthesis of Highly Functionalized Benzimidazolones and Benzoxazolones through an Electrochemical Dehydrogenative Cyclization Cascade. *Angew. Chem., Int. Ed.* **58**, 9017-9021 (2019).
11. Hare, A.A.*, et al.* Optimization of N-benzyl-benzoxazol-2-ones as receptor antagonists of macrophage migration inhibitory factor (MIF). *Bioorg. Med. Chem. Lett.* **20**, 5811-5814 (2010).
12. Lu, K.*, et al.* DMF-Promoted Redox-Neutral Ni-Catalyzed Intramolecular Hydroarylation of Alkene with Simple Arene. *ACS Catal.* **8**, 3913-3917 (2018).
13. Wu, Z.J., Li, S.R., Long, H. & Xu, H.C. Electrochemical dehydrogenative cyclization of 1,3-dicarbonyl compounds. *Chem. Commun.* **54**, 4601-4604 (2018).
14. Yin, K. & Zhang, R.H. Transition-Metal-Free Direct C-H Arylation of Quinoxalin-2(1H)-ones with Diaryliodonium Salts at Room Temperature. *Org. Lett.* **19**, 1530-1533 (2017).
15. Bering, L., Jeyakumar, K. & Antonchick, A.P. Metal-Free C–O Bond Functionalization: Catalytic Intramolecular and Intermolecular Benzylation of Arenes. *Org. Lett.* **20**, 3911-3914 (2018).
16. Cong, X., You, J., Gao, G. & Lan, J. 2-Pyridylmethyl ether: a readily removable and efficient directing group for amino acid ligand accelerated ortho-C–H olefination of phenols.

Chem. Commun. **49**, 662-664 (2013).
17. Chen, C.Y., *et al.* Synthesis of Quinazolines via an Iron-Catalyzed Oxidative Amination of N-H Ketimines. *J. Org. Chem.* **83**, 2395-2401 (2018).
18. Chen, C.Y., *et al.* A Synthesis of 1H-Indazoles via a Cu(OAc)(2)-Catalyzed N-N Bond Formation. *Org. Lett.* **18**, 1690-1693 (2016).
19. Dong, J., Wu, Z., Liu, Z., Liu, P. & Sun, P. Rhodium(III)-Catalyzed Direct Cyanation of Aromatic C–H Bond to Form 2-(Alkylamino)benzonitriles Using N-Nitroso As Directing Group. *J. Org. Chem.* **80**, 12588-12593 (2015).
20. Zhao, H.B., *et al.* Amidinyl Radical Formation through Anodic N-H Bond Cleavage and Its Application in Aromatic C-H Bond Functionalization. *Angew. Chem., Int. Ed.* **56**, 587-590 (2017).
21. Qin, C.X., Chen, X., Hughes, R.A., Williams, S.J. & Woodman, O.L. Understanding the Cardioprotective Effects of Flavonols: Discovery of Relaxant Flavonols without Antioxidant Activity. *J. Med. Chem.* **51**, 1874-1884 (2008).
22. Rajput, S., *et al.* Structure–Activity Relationships of cyclo(l-Tyrosyl-l-tyrosine) Derivatives Binding to Mycobacterium tuberculosis CYP121: Iodinated Analogues Promote Shift to High-Spin Adduct. *J. Med. Chem.* **62**, 9792-9805 (2019).
23. Hu, P., *et al.* Bio-inspired iron-catalyzed oxidation of alkylarenes enables late-stage oxidation of complex methylarenes to arylaldehydes. *Nat. Commun.* **10**, 2425 (2019).
24. Goossen, L.J. & Knauber, T. Concise Synthesis of Telmisartan via Decarboxylative Cross-Coupling. *J. Org. Chem.* **73**, 8631-8634 (2008).
25. Frisch, M. J. et al. Gaussian 09 Revision D.01 (Gaussian Inc. Wallingford CT, 2009).
26. Becke, A. D. Density-functional exchange-energy approximation with correct asymptotic behavior. *Phys. Rev. A* **38**, 3098-3100 (1988).
27 Lee, C., Yang, W. & Parr, R. G. Development of the colle-salvetti correlation-energy formula into a functional of the electron density. *J. Chem. Phys.* **37**, 785-789 (1988).
28. Becke, A. D. Density-functional thermochemistry. 3. The role of exact exchange. *J. Chem. Phys.* **98**, 5648–5652 (1993).
29. Dunning Jr, T. H. Gaussian basis sets for use in correlated molecular calculations. I. The atoms boron through neon and hydrogen. *J. Chem. Phys.* **90**, 1007-1023 (1989).
30. Keith, J. A. & Carter, E. A. Theoretical insights into pyridinium-based photoelectrocatalytic reduction of CO_2. *J. Am. Chem. Soc.* **134**, 7580-7583 (2012).
31. Cossi, M., Rega, N., Scalmani, G. & Barone, V. Energies, structures, and electronic properties of molecules in solution with the C-PCM solvation model. *J. Comput. Chem.* **24**, 669-681 (2003).
32. Lu, T. & Chen, F. Multiwfn: a multifunctional wavefunction analyzer. *J. Comput. Chem.* **33**, 580–592 (2012).
